# Prediction of COVID-19 Waves Using Social Media and Google Search: A Case Study of the US and Canada

**DOI:** 10.3389/fpubh.2021.656635

**Published:** 2021-04-16

**Authors:** Samira Yousefinaghani, Rozita Dara, Samira Mubareka, Shayan Sharif

**Affiliations:** ^1^School of Computer Science, University of Guelph, Guelph, ON, Canada; ^2^Sunnybrook Health Sciences Center, Toronto, ON, Canada; ^3^Department of Pathobiology, University of Guelph, Guelph, ON, Canada

**Keywords:** digital data stream, Twitter, Google Trends, COVID-19, early warning

## Abstract

The ongoing COVID-19 pandemic has posed a severe threat to public health worldwide. In this study, we aimed to evaluate several digital data streams as early warning signals of COVID-19 outbreaks in Canada, the US and their provinces and states. Two types of terms including symptoms and preventive measures were used to filter Twitter and Google Trends data. We visualized and correlated the trends for each source of data against confirmed cases for all provinces and states. Subsequently, we attempted to find anomalies in indicator time-series to understand the lag between the warning signals and real-word outbreak waves. For Canada, we were able to detect a maximum of 83% of initial waves 1 week earlier using Google searches on symptoms. We divided states in the US into two categories: category I if they experienced an initial wave and category II if the states have not experienced the initial wave of the outbreak. For the first category, we found that tweets related to symptoms showed the best prediction performance by predicting 100% of first waves about 2–6 days earlier than other data streams. We were able to only detect up to 6% of second waves in category I. On the other hand, 78% of second waves in states of category II were predictable 1–2 weeks in advance. In addition, we discovered that the most important symptoms in providing early warnings are fever and cough in the US. As the COVID-19 pandemic continues to spread around the world, the work presented here is an initial effort for future COVID-19 outbreaks.

## 1. Introduction

The COVID-19 pandemic caused by SARS-CoV-2 has been spreading rapidly and continuously posing a significant threat to human lives worldwide. Providing early signals ahead of outbreaks is essential for early public health responses. Prediction systems for other diseases have been built to facilitate management in disease emergencies and making rapid policy decisions (1, 2).

Disease monitoring and surveillance are essential to create situational awareness and initiate timely responses. Since the availability of testing is different from country to country, online platforms can help in monitoring disease occurrences. Web-based platforms can serve as sources where users self-report or search their health-related issues. Social media, in particular Twitter, has been taken into consideration for COVID-19 surveillance purposes.

Several studies attempted to track the volume of health-related online content and associated it with official cases or deaths (3, 4). In a recent work by Mackey et al., English Twitter conversations were collected and used in an unsupervised machine learning approach to assess users' self-reports of COVID-19 symptoms, testing, and recovery from disease. The results showed that the volume of tweets regarding “symptoms” and “lack of testing” increased at the same time as a surge in the number of confirmed cases in the United States. Similarly, an overlap between COVID-19 cases and discussions on Twitter and Weibo has been shown (5, 6).

In addition to finding a connection between disease cases/deaths and social media posts, Gharavi et al. (7) utilized social media for early reporting of disease cases. A regression analysis was performed for a number of states in the US, which found a connection between the number of tweets related to “cough” and “fever” and officially reported cases with a 5–19 days lag (7).

Search engines have been analyzed to monitor COVID-19 activities too (8, 9). A study utilized multiple digital data sources, including Google Trends to calculate the probability of exponential growth/decay in COVID-19 activities as early signals of the epidemic in Massachusetts, New York, and California states (4). Another study in the United States found a high correlation between search trends and the number of cases with a 7-day lag (10).

In addition to the US, Google search trends were used to predict COVID-19 incidence in Iran (11) and Colombia (12). The study in Iran used Linear regression and long short-term memory (LSTM) models and found that “hand sanitizer,” “handwashing,” and “antiseptic” were the most effective factors in case predictions.

The present study aimed to examine the potential of online platforms in providing early warnings of first and second waves of COVID-19 outbreaks in the US and Canada for an 8-month period. The main objectives were: (1) to visualize the correlation between digital data sources and COVID-19 official cases; (2) to compare various sources of internet-driven data in terms of their timeliness and precision in providing alert signals of disease waves; and (3) to prioritize COVID-19 symptoms by their values in detecting disease trends.

The first novelty here is utilizing historical and precisely geo-located tweets at provincial/state levels. A growing body of research has been centered around using online content for providing early warning signals of pandemics. The Twitter data used in the existing work of COVID-19 is limited to streaming or standard search APIs that cannot go more than a week back in time (3, 5, 13). Moreover, the above-mentioned studies either had no geographical restrictions on collected tweets (14, 15) or locations have been specified using self-reported information associated with user accounts or tweet contents for a small percentage of tweets (6, 16, 17).

The other novel aspect of the present study lies in comparing the disease predictive value of various data in terms of differences in platforms and keywords. Previous work has explored the correlation between COVID-19 indicator terms of online content and the number of infected individuals (18–20). However, the potential of internet-driven information in providing early warning of COVID-19 outbreaks is still poorly understood.

## 2. Materials and Methods

**Figure 1:**
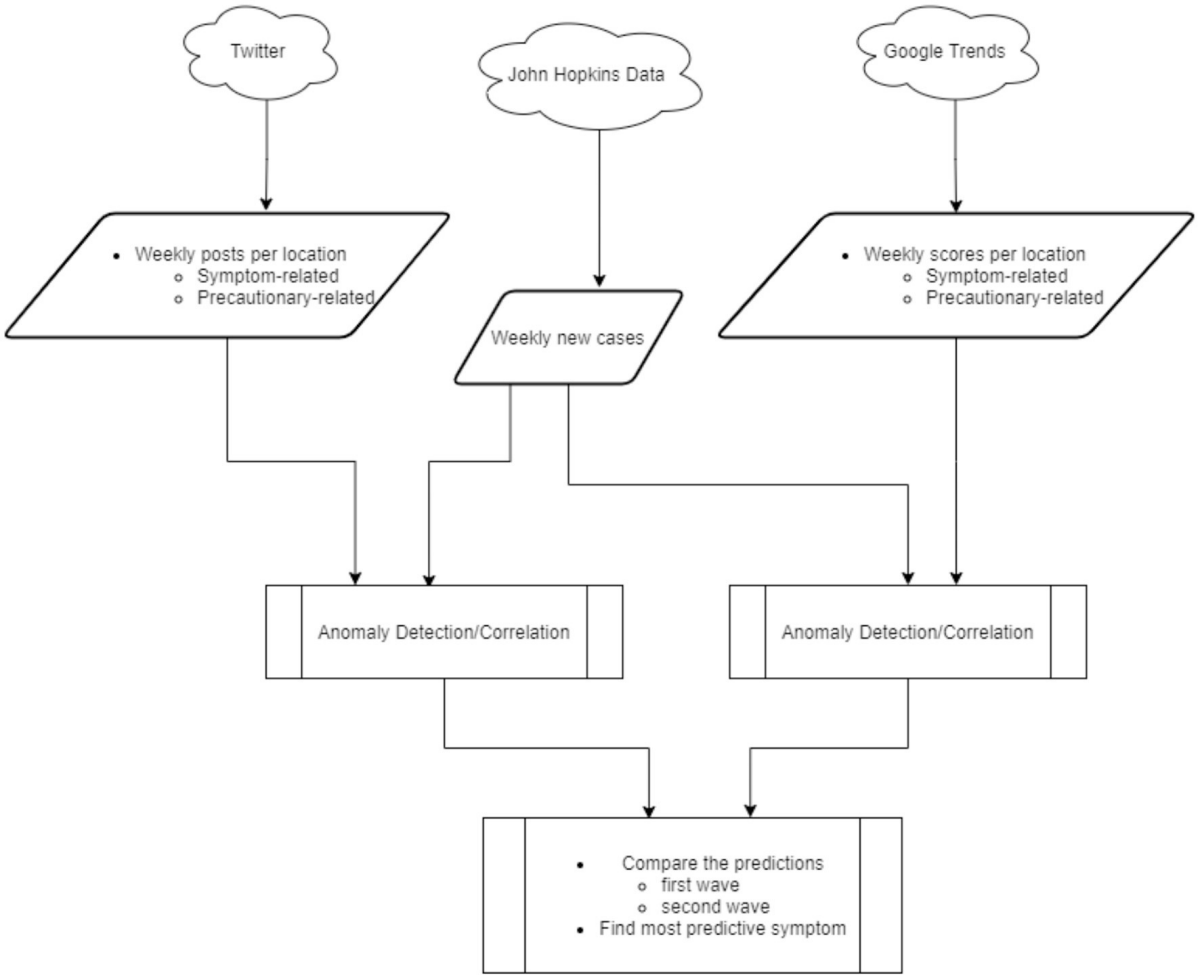
Overall approach.

In this work, we collected Twitter posts and Google search scores related to symptoms and control measures of the COVID-19 outbreak in Canada and the US from January 2020 to September 2020. Subsequently, the weekly time-series of online activities and COVID-19 new cases were employed in anomaly detection and correlation models. Then, we explored and compared the potential of social media and search platforms in providing early warnings of outbreak waves on national and local scales. Furthermore, we compared the ability of COVID-19 symptoms in predicting outbreak waves. An overview of the overall flow of the study is given in Figure 1.

### 2.1. Data Collection

**Ground Truth Data**: We collected the cumulative number of cases and deaths of COVID-19 in Canada and the United States from Johns Hopkins COVID-19 data repository (21). The data included geographical information, such as province, city, latitude, and longitude. The daily number of new cases was calculated from the initial cumulative numbers. Subsequently, the weekly number of new cases was computed for the US and Canada as well as their states/provinces.

**Twitter Data**: Twitter Premium Search application programming interface (API) (22) was used to retrieve tweets containing COVID-19 symptoms and preventive measures posted from the specified geographical locations. A list of keywords that were included in or excluded from the Twitter search query is given in Table 1.

**Table 1:** Twitter query input.

Included symptom keywords	Shortness of breath, cough, fever, sore throat, loss of smell, loss of taste
Included precaution keywords	Face mask, quarantine, wearing mask, wash hand, ovid-19 vaccine, covid-19 vaccine, covid vaccine, corona vaccine, coronavirus vaccine, physical distancing, social distancing
Excluded symptom keywords	Flu, influenza, cold, diabetes, jungle fever, Saturday night fever, fever swamp, baby fever, fever pitch, fever dream, fever 333, dog fever, cat scratch fever, blackouts coastal fever, tattoo fever, Kennel cough, smoke, smoking, allergy, allergies
Excluded precaution keywords	Handle, handling, body wash, hand cream, cold, flu, yogurt, honey, watermelon, cucumber, hair mask

In total, around 300K tweets were collected from January 2020 to September 2020. This included 202K symptom-related and 95K preventive-related tweets. We determined the province/state that each tweet was posted from using the city names returned by Twitter. The provincial/state number of retrieved tweets associated with Canada and the United States for categories of symptom and precaution keywords is given in Tables B1, B2, respectively, in the Supplementary Material.

#### 2.1.1. Google Trends Data

The “Interest_over_time” scores were acquired from Google Trends (23) given national or local locations and the same keywords we used in Twitter search API. We used provinces/states names to pull the data. The scores indicate the popularity of terms over a specified time range and region. Google Trends scores are based on the absolute search volume for a term, relative to the number of searches received by Google. Scores are quantified as indexes, with 100 showing the maximum search interest and zero showing no interest.

### 2.2. Visual Trends

The weekly number of tweets and search scores was plotted against the weekly COVID-19 cases on national and provincial/state scales. Given the line plots, one can visually detect the fist/second-half waves of outbreaks for each province/state and compare the online activities with the reported COVID-19 cases. Further, we plotted the distribution of tweets across various symptoms, which can help gain insight into how specific terms can be connected to official disease cases.

### 2.3. Detection of Pandemic Waves

Anomaly detection in time-series is formulated as identifying outliers or unusual data points relative to some standard or usual signals (24). We applied The Seasonal-Hybrid Extreme Studentized Deviate (SH-ESD) (25, 26) algorithm on the weekly time-series of online activities to eventually identify the onset and peak of COVID-19 waves. SH-ESD algorithm was designed in particular for finding anomalies in the cloud infrastructure (26). The algorithm is built based on the Generalized ESD test and includes a statistical test called Grubb's Test and a time-series decomposition method, known as Seasonal-Trend Decomposition based on Loess (STL). Once decomposition extracts the symmetrically distributed residual component of the observed data, Grubb's Test identifies outliers in a sample of residuals (25, 26).

Weekly time-series of cumulated search scores and the number of tweets were calculated on national and local levels for Canada and the US. Subsequently, we employed an R package “AnomalyDetection,” which uses SH-ESD method and was released by the Twitter engineering team (27). Finally, we compared the lag time between detected anomalies and the onset and peak times of outbreak waves for all provinces/states. The comparison could help understand the potential of online discussions and searches in providing early warnings of outbreak waves. The onset of a wave was defined as a week when the number of new cases jumped to at least 50, and the peak was defined as the week when the number of new cases reached its maximum in the wave. Finally, we calculated average lags and the percentage of correct detections for symptom and precaution related data for each platform in each nation.

To further evaluate the quality of detections, correlation measures between time series of activities in each province/state and corresponding actual COVID-19 cases were calculated using the Pearson correlation coefficients (r) (28). The coefficient of one (r = 1) shows that the two data series are matching and if no correlation exists, the coefficient will be zero (r = 0).

### 2.4. Most Predictive Symptoms

In order to differentiate COVID-19 symptoms for their ability in predicting pandemic trends, we filtered the time-series of tweets by symptoms for each location. Subsequently, the anomaly detection analysis was applied to all symptom-specific time-series similar to the previous section. We compared the detected anomalies from time-series of all keywords with the peaks of waves in each province/state and reported the average measures.

## 3. Results

### 3.1. Visual Trends

**Figure 2:**
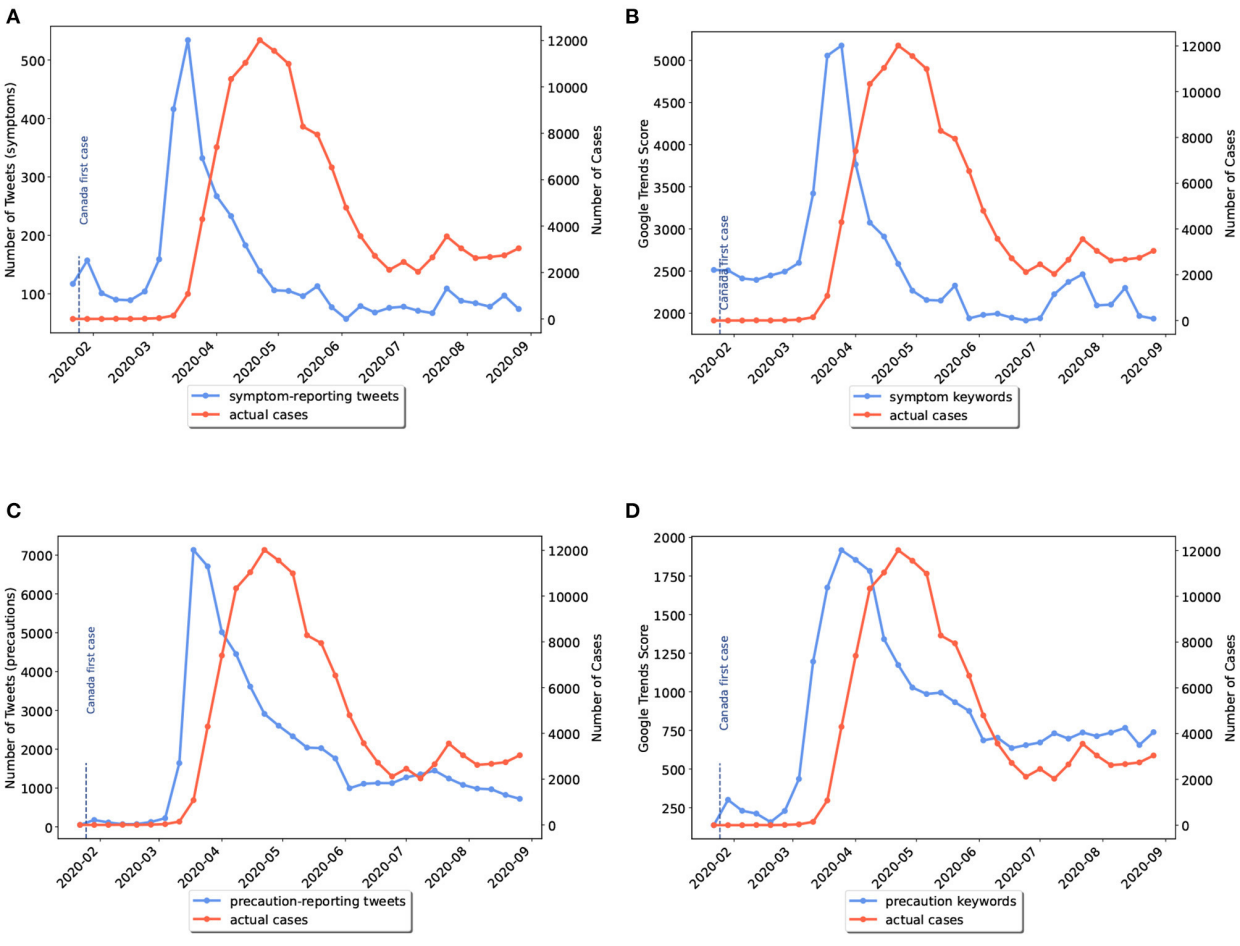
Weekly comparison of online activities and actual number of cases in Canada. **(A)** Symptom-related tweets vs. cases, **(B)** Symptom-related searches vs. cases, **(C)** Precaution-related tweets vs. cases, **(D)** Precaution-related searches vs. cases.

Twitter posts and Google Trends search interests on symptoms and precautionary measures of COVID-19 were plotted weekly against the number of COVID-19 cases. As an example, four curves are given in Figure 2 showing information from Mid-January till September for Canada. Figures 2A,C compare disease cases to the time-series of tweets discussing symptoms and preventive measures, respectively. On the other hand, Figures 2B,D present a comparison between disease cases and Google Trends scores for searches on symptoms and preventive measures, respectively. The online activities are plotted with blue color while the official cases are shown in red color. Additional charts related to other locations are included in the Supplementary Material.

Visually comparing the ability of platforms in giving early warnings in the beginning of the pandemic, Twitter activities on disease symptoms in the majority of states/provinces showed slightly earlier peaks than Google (e.g., Figures A44–A46, A49–A51 in the Supplementary Material). However, comparing the trends for the second waves, Google searches on symptoms showed more noticeable peaks than Twitter (e.g., Figures A8, A10, A12, A17 in the Supplementary Material).

In general, after peak times, when the number of cases started to decrease, people gradually stopped posting or searching about symptoms. This might be due to knowledge saturation which makes the outbreak monitoring more challenging as time passes. On the other hand, trends for precautions kept steady (e.g., Figures 2C,D). The reason behind the steadiness of time-series of precautionary-related data could be due to the impact of news media reporting regulations imposed by governments regardless of the number of cases.

It is worth noting that compared to symptoms, preventive terms were more discussed on Twitter and less searched on Google for all geographical locations. For example, the weekly number of tweets reporting symptoms reached a peak of 500 in Canada while the peak of tweets discussing precautionary measures was 14 times more (Figure 2). On the other hand, the maximum cumulative search score of symptom keywords was more than twice the maximum score of precaution keywords. Thus, we could conclude that internet users tend to post on Twitter to discuss control measures and search their symptoms on Google.

Several states of the US, such as Alabama, Tennessee, Utah, and Texas did not experience the first wave of the pandemic (see **Figure 4**). Nevertheless, online discussions and searches about COVID-19 symptoms and control measures soared in March. Having a closer look at a sample of tweets, we noted only 20% of early tweets for the above-mentioned provinces were regarding self-reporting of symptoms. The rest of the tweets were posted from users being anxious about or scared of COVID-19 symptoms. Similarly, internet users likely would search the related terms on Google when they are afraid of pandemic news about other states.

### 3.2. Detection of COVID-19 Waves

**Figure 3:**
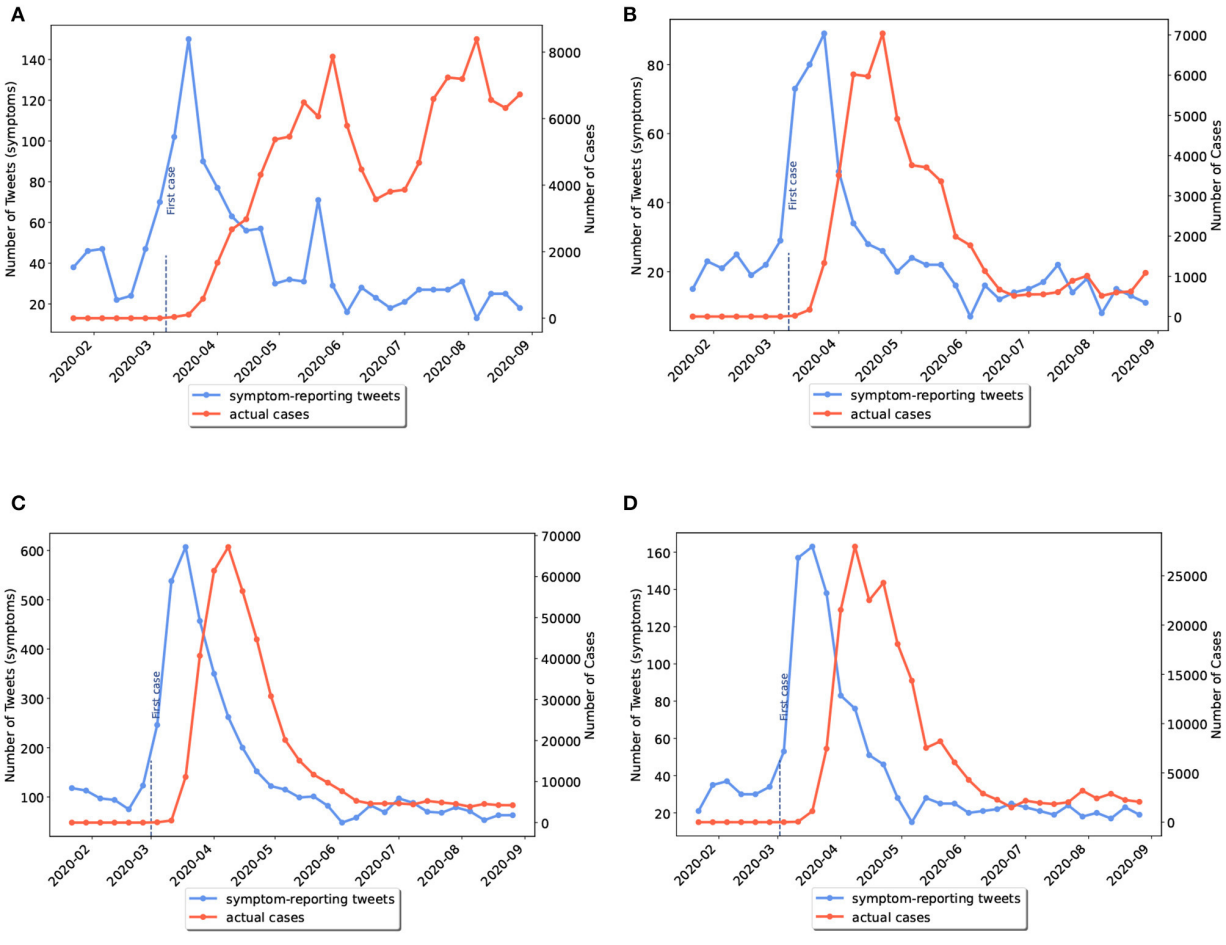
Weekly comparison of symptom-related tweets and the actual number of cases in provinces with first wave of disease (category I). **(A)** Virginia, **(B)** Connecticut, **(C)** New York, **(D)** New Jersey.

**Figure 4:**
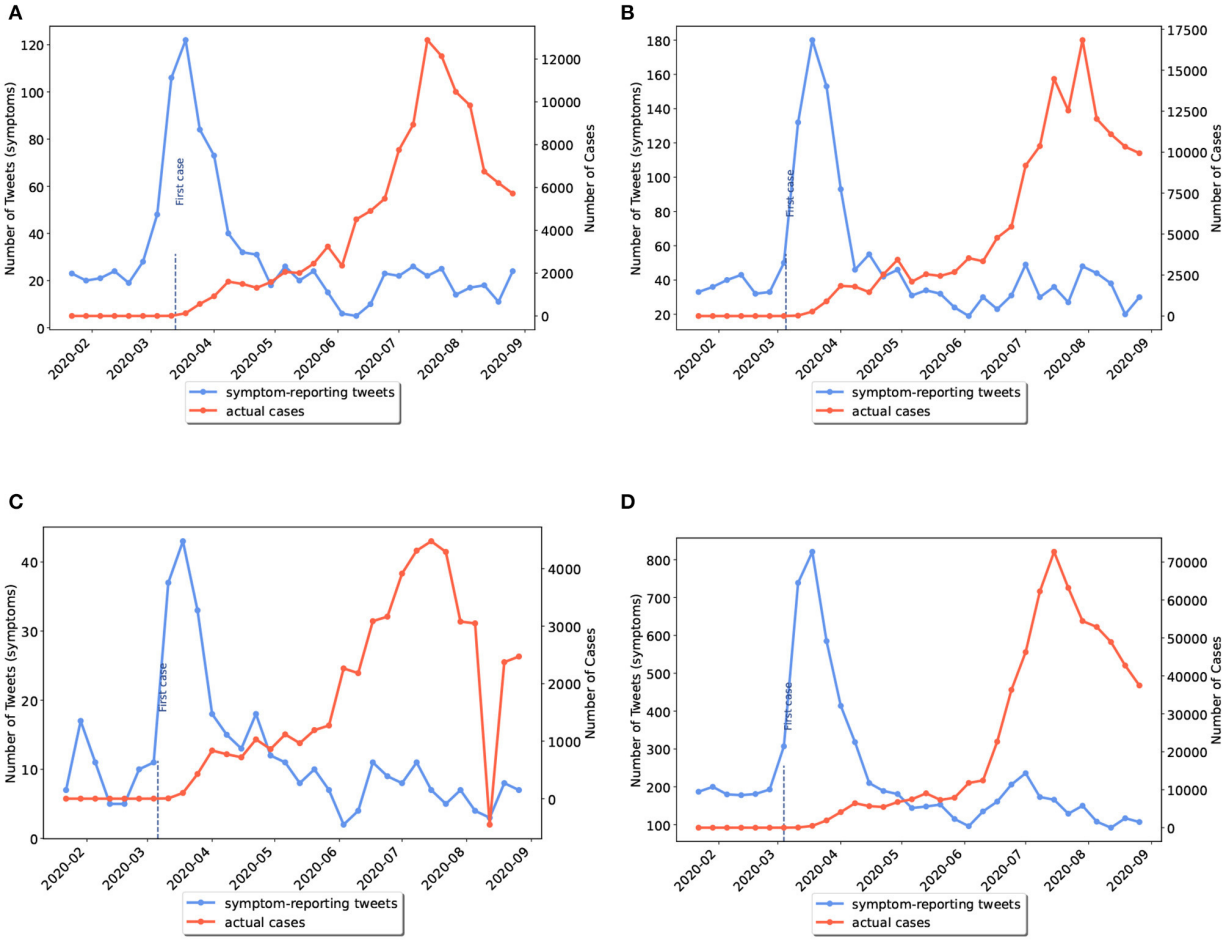
Weekly comparison of symptom-related tweets and the actual number of cases in states with only the second wave of disease (category II). **(A)** Alabama, **(B)** Tennessee, **(C)** Utah, **(D)** Texas.

As previously mentioned, some US states had not experienced the first wave of disease. We grouped the US states into two categories: (I) states that had experienced a peak of disease wave before June 2020; (II) states that had experienced a wave peak only after June 2020, which included Alaska, North Carolina, Utah, Alabama, Tennessee, California, Arizona, and Texas. In the latter category, waves actually started before June but reached their peak in the second half of the study period. Examples of the first and second categories are given in Figures 3, 4, respectively.

After applying anomaly detections on the time-series of different sources of internet data for the provinces/states in Canada and the US, we presented the outcomes in Tables 2, 3, respectively. We quantified the average number of weeks that each source of data can provide anomalies before the start and peak of waves. As mentioned in section 2, the onset of a wave was defined as the point when new cases reached at least 50 and the peak as the point when cases got to their maximum. Similarly, we calculated the percentage of waves in provinces/states of these countries that can be detected earlier given a specific source of data (e.g., Twitter or Google Trends). In Table 3, we presented separate prediction outcomes for the previously mentioned categories of the US states.

**Table 2:** The average prediction value of Canadian provinces (with an early wave).

**Source**	**Start1**	**Peak1**
**Twitter**		
Symptoms (week lags)	1.19	4.3
Symptoms (detection percentage)	50%	50%
Precautions (week lags)	0.4	2.8
Precautions (detection percentage)	83%	83%
**Google Trends**		
Symptoms (week lags)	1.2	3.1
Symptoms (detection percentage)	83%	83%
Precautions (week lags)	1.2	3.2
Precautions (detection percentage)	75%	75%

**Table 3:** The average prediction value of the US states.

**Provinces**	**Source**	**Start1**	**Peak1**	**Start2**	**Peak2**
**Twitter**					
Category I	Symptoms (week lags)	**1.83**	5	6	–
	Symptoms (detection percentage)	**100%**	81%	3.2%	0%
	Precautions (week lags)	0.94	4.39	2	3.42
	Precautions (detection percentage)	97%	89%	6%	22%
Category II	Symptoms (week lags)	–	–	1.86	–
	Symptoms (detection percentage)	–	–	**78%**	0%
	Precautions (week lags)	–	–	1.14	2.5
	Precautions (detection percentage)	–	–	**78%**	44%
**Google Trends**					
Category I	Symptoms (week lags)	1.54	4.75	7	3.87
	Symptoms (detection percentage)	**100%**	86%	3%	26%
	Precautions (week lags)	1.4	4.75	6	1.75
	Precautions (detection percentage)	**100%**	86%	3%	13%
Category II	Symptoms (week lags)	–	–	1	1.75
	Symptoms (detection percentage)	–	–	**78%**	44%
	Precautions (week lags)	–	–	1.14	4
	Precautions (detection percentage)	–	–	**78%**	11%

*The bold values are the best obtained results*.

Table 2 shows that except for the precaution-related tweets, the rest of the sources acted the same in the detection time of onsets of waves. The symptom-related tweets showed anomalies 4.3 weeks before the waves peak, which is about 1 week earlier than other sources of data (i.e., 3 weeks). However, the percentage of detection was only 50% which was less compared to the rest of the sources.

Overall, the result presented here demonstrated that Google Trends performed better in terms of the number of early warning weeks and the percentage of correct predictions. Utilizing Google Trends enabled us to identify starts and peaks of waves in Canada in average for about 1 and 3 weeks earlier, respectively. In terms of detection percentages, the symptom-related searches with a detection percentage of 83% outperformed the precautionary-based searches with a detection percentage of 75%.

Additionally, we observed a strong and statistically significant (*p*-value < 0.05) correlation between the Twitter/Google activities and the number of cases of the disease in Canada. Table 4 shows correlations of above 75% with lags of 3–5 weeks for all sources of data.

**Table 4:** Correlation coefficients (r) of weekly online activities and COVID-19 cases.

**Location**	**TW symptoms**	**TW precautions**	**GT symptoms**	**GT precautions**
Canada	0.85 (lag = 5)	0.93 (lag = 3)	0.75 (lag = 5)	0.84 (lag = 3)
Massachusetts	0.94 (lag = 5)	0.9 (lag = 3)	0.66 (lag = 5)	0.86 (lag = 4)
Michigan	0.7 (lag = 3)	0.81 (lag = 2)	0.38 (lag = 4)	0.87 (lag = 3)
New Jersey	0.95 (lag = 4)	0.87 (lag = 2)	0.7 (lag = 5)	0.9 (lag = 3)
New York	0.97 (lag = 3)	0.86 (lag = 2)	0.72 (lag = 4)	0.91 (lag = 3)
Vermont	0.9 (lag = 2)	0.83 (lag = 1)	0.63 (lag = 3)	0.88 (lag = 2)

During the time period covered in the present study, the majority of Canadian provinces had not encountered a major second wave except for British Columbia and Manitoba. The online content generated in these two provinces did not show strong correlations with the actual number of disease cases. Moreover, the analysis used in the present study was not capable of detecting the second waves in Canada.

Similar to Canada, the anomaly detection results for the US is given in Table 3. Comparing the ability of Twitter and Google in detecting the start of the first waves, symptom related posts and searches as well as precaution-related searches were capable of detecting 100% of first waves. However, symptom-related tweets could detect the start of first waves about 2–3 days earlier than Google trends and about 6 days earlier than tweets related to precautions. The lag time of symptom-related searches (e.g., 1.54 weeks) matched with the findings of a previous study in China (29). The Baidu searches on symptoms could detect the increase in the number of COVID-19 cases for 6–9 days earlier.

The results revealed that Twitter and Google Trends performed better in detecting the onset of second waves in category II than category I states. Posts and searches identified the start of second waves in 78% of provinces in category II states while the detection percentage for the second wave for the category I states was up to 6%. With regard to time, symptom-related tweets identified the start of second waves in category II about 5 days earlier than other sources.

Overall, higher percentages of detections in early waves than late waves were observed. This could be due to social media users being exhausted and less motivated to post or search on the internet as their level of concern had decreased over time. This is referred to as “pandemic fatigue” in psychology (30).

Furthermore, we observed statistically significant (*p*-value < 0.05) and strong correlations between online data and disease cases for the US states of category I. A sample of locations is given in Table 4 and the rest can be found in Table B3 in the Supplementary Material. In general, lags in the second and fourth columns (i.e., symptoms) are higher than the third and fifth columns (i.e., precautions). The same pattern was found in the anomaly detection results in Table 3. On the other hand, precaution-related series showed stronger correlations than symptom-related series.

The findings of a study in Taiwan (9) in the early stages of COVID-19 outbreak are consistent with our result (the fifth column) in Table 4. Authors found that Google searches on “hand washing” and “face mask” increased 1–3 days prior to the increase in COVID-19 cases. However, our findings in the fourth column (GT symptoms) did not match with the findings in Italy, Spain, UK, USA, Germany, France, Iran, and The Netherlands (8). In comparison with the moderate correlations presented in the fourth column, Walker et al. discovered a strong correlation between the number of searches for “loss of smell”-related information and the number of COVID-19 cases.

### 3.3. Prediction Values of Symptoms

**Figure 5:**
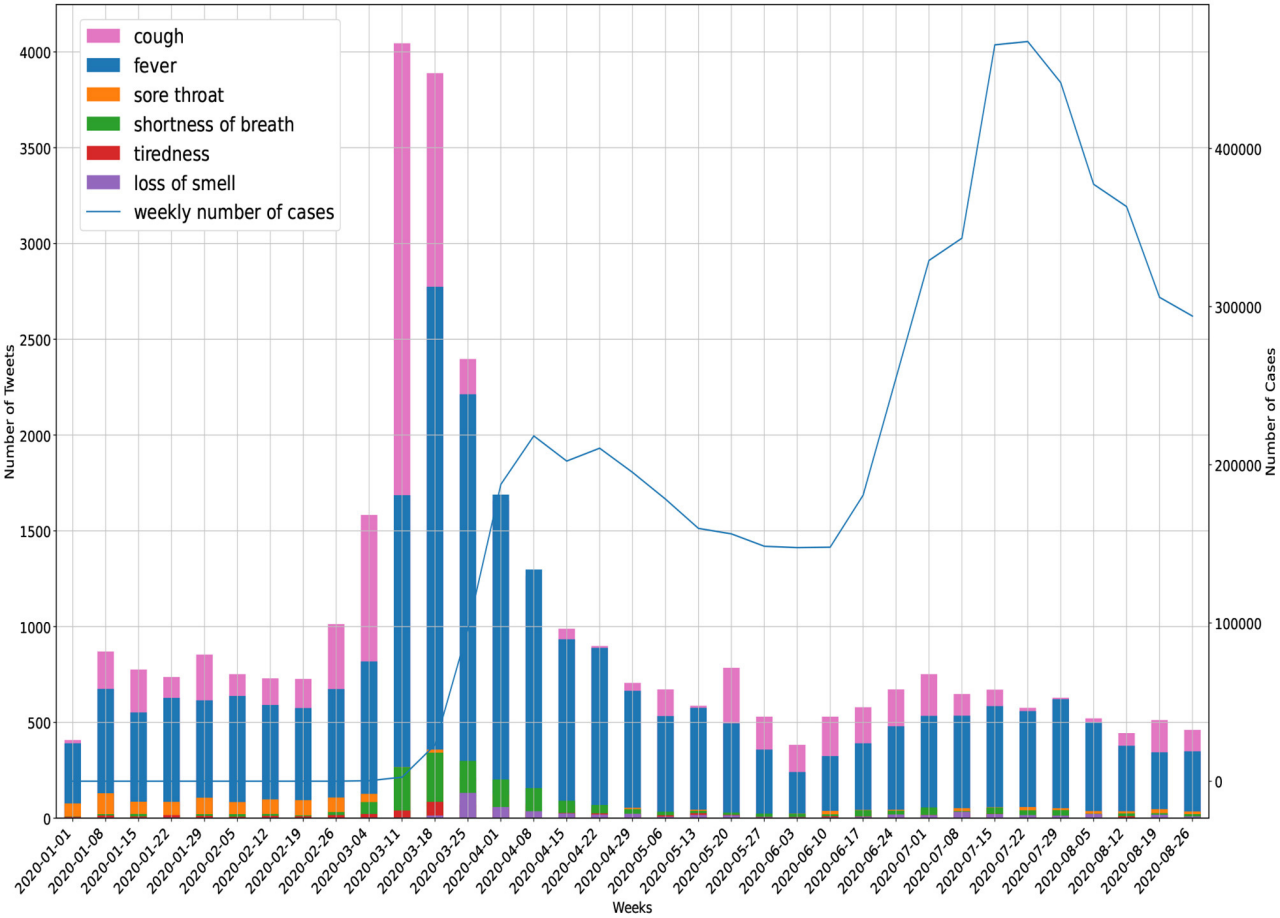
Symptom reporting in Twitter (United States).

The progression of tweets related to COVID-19 symptoms during the course of the present study is given in Figure 5. Furthermore, a quantitative analysis was performed to find anomalies in symptom-specific time-series of tweets for all US states (see Table 5).

**Table 5:** The average prediction values of the US states (detection of early waves).

**Source**	**Peak1 (Twitter)**	**Peak1 (Google Trends)**
Fever (week lags)	4.45	4.03
Fever (detection percentage)	53%	58%
Cough (week lags)	5.2	4.2
Cough (detection percentage)	55%	44%
Tiredness (week lags)	5.2	–
Tiredness (detection percentage)	20%	0%
Shortness of breath (week lags)	4.38	4.27
Shortness of breath (detection percentage)	29%	24%
Loss of smell (week lags)	3.33	–
Loss of smell (detection percentage)	7%	0%
Sore throat (week lags)	4.29	4.24
Sore throat (detection percentage)	38%	55%

Manually, we looked at a sample of tweets (1K) for the peak time of symptoms (4 March–25 March) in Figure 5 and categorized them. We noted that more than 50% of tweets were about self-reporting of symptoms. For example, users reported their symptoms in tweets, such as “I haven't coughed this much in my life. It's a really violent dry cough. My chest hurts.” and “My sweet daughter has a high fever for 3 days.” The next major category (25%) was the educational tweets, such as “Limit the spread of illnesses like #COVID19: sneeze or cough into a tissue or your elbow, and dispose of used tissues.” In the last category (20%) we found comic feeds like “waiting until my roommates asleep to cough.”

The volume of tweets related to “sore throat” was high at the beginning of the study period and then decreased. The sample tweets in the first 2 months showed that the majority of discussions were around “sore throat” due to the cold season. After that tweets reporting “tiredness” and “shortness of breath” started to grow. Also, it is visually clear that “cough” and “fever” were better trend indicators of official cases compared with other symptoms.

The quantitative results in Table 5 are consistent with the visual implications above. We were able to predict first waves of the pandemic in more than half of the US states using tweets regarding “fever” and “cough.” Tweets related to all symptoms predicted the peaks of the first wave with an average within the range of 3.3–5.2 weeks earlier than official peaks of cases. Terminologies, such as “tiredness” and “loss of smell” showed the lowest percentage of detections (i.e., up to 20%) among all symptoms.

## 4. Discussion

We aimed to perform a comparative study to understand the potential of Twitter activities and Google searches to be used in early warning systems of COVID-19 pandemic in Canada and the US. Time-series of Twitter posts and Google search scores on several symptoms and precautionary terms were compared with the actual cases qualitatively and quantitatively. Subsequently, we assessed the prediction values of different sources of data in providing early warnings of pandemic waves. Additionally, we made an effort to prioritize symptoms based on their predictive values.

The qualitative results indicated that overall, in the beginning of the pandemic, Twitter posts related to symptoms showed earlier trends compared to Google searches. However, during the second half of the study period (e.g., June–August), Google searches of symptoms could show more noticeable trends. Furthermore, we observed fixed trends of the precautionary time-series after the first waves, which might be due to news media influencing internet users. Visual observations also indicated that internet users tend to discuss preventive measures of COVID-19 on Twitter and search disease symptoms on Google.

Pearson correlation analysis demonstrated an overall strong correlation between official cases and the relevant posts and searches related to Canada. Except for British Columbia and Manitoba, other Canadian provinces have shown correlation coefficients of above 75% with a lag of 3–5 weeks. We did not observe a strong correlation for British Columbia and Manitoba as they did not experience major early waves of disease. Anomaly detections in time-series of Canada revealed that symptom-related Google searches showed the best performance in predicting the onset and peak of first waves about 1 and 3 weeks earlier, respectively.

Although several states in the US did not experience the early waves, the online activities started to grow in March. Similar findings were reported by other studies (31). Increasing activities in social media in the absence of outbreaks is likely due to the panic of the pandemic in other states. We divided states into categories I and II for those with and without early waves, respectively. We observed strong correlations for states in category I. In particular, symptom-related tweets showed the highest correlations. Previous studies have also shown strong (27 days lag) but state different correlations for the US states (32). Additionally, we found that correlation lags for posts and searches of symptoms were higher compared to preventive measures.

The prediction of the first waves in the present study outperforms the detection of second waves. This was aligned with the correlation results being weak for the locations with only the second waves. In other words, the correlations faded as the pandemic proceeded in weeks. This could be due to two following reasons: (1) public began to feel exhausted with the pandemic and were less likely to follow public health practices and (2) COVID-19 related subjects, such as symptoms and remedies became well-known among the public. Thus, the approach presented in the paper is more suitable for the initial wave of an outbreak as it reflects the public's anxiety or curiosity and desire to learn about disease symptoms and control measures. Based on the results presented in this paper, it is expected that there will be less engagement through social media in the second and future waves of the outbreak. However, if new symptoms and variants of the virus appear or if public health imposes new control measures in the future, then the proposed approach might be appropriate for the second and future waves.

The analyses on symptom-specific time-series of the US demonstrated that tweets related to “fever” and “cough” had the highest performance in predicting the first waves, which is aligned with the study by Gharavi et al. (7). On the other hand, tweets related to “tiredness” and “loss of smell” could only predict up to 20% of the waves. These results were in contrast with previous studies (8, 33). Walker et al. showed a strong correlation between the frequency of Google search results related to “loss of smell” and the onset of COVID-19 infection in several countries. Similarly, Asseo et al. revealed a correlation between Google searches for “loss of taste” and “loss of smell” symptoms with the number of cases. However, the correlation was found only for a short period of time when people were surprised by new cases and media coverage. A reason behind the differences in findings could be the fact that Walker et al. and Asseo et al. have used Google searches while our study and the study by Gharavi et al. have used Twitter posts.

The scope of present study is larger in terms of using data with a longer duration and geographical extent compared with a previous studies of the US (4, 7, 10). While we studied all the US states from January 2020 to September 2020, Gharavi et al. performed an analysis for a duration up to April 2020 for the six most affected states of the US. Compared to only “fever” and “cough” terms that were analyzed by Gharavi et al., we employed a wider range of symptoms and the Twitter posts have been filtered during the data collection to avoid irrelevant content.

Despite the strengths of the approach taken in this study and many other existing work, the number of confirmed cases used here might be an underestimate of the actual number of cases due to the lack of testing kits in the beginning of the pandemic (34). Initially, regions had travel-based, symptom-based, or contact-based testing policies that might have not been identified. In the future, it is of interest to indicate whether social media is a better indicator of new cases after regions had open testing for everyone. Moreover, tweets are generated by individuals who are capable of accessing and using social media and search engines. Therefore, it is possible that there may be a bias in favor of certain age groups or individuals belonging to certain socioeconomic groups.

Here, we assumed equal weights for counting tweets. However, engagement metrics, such as re-tweets, replies, follows, favorites and links can be used to assign an important weight to each tweet. Future studies therefore might calculate the weighted sum of tweets in building time-series. Additionally, future work can analyze social media and search signals collectively. Fusion approaches could be used to integrate evidence from several sources, which might lead to more precise predictions.

## Data Availability Statement

The raw data supporting the conclusions of this article will be made available by the authors, without undue reservation.

## Author Contributions

SY: writing–original draft preparation, investigation, methodology, software, data curation, visualization, and validation. RD: supervision, investigation, conceptualization, writing, reviewing, and editing, and validation. SM: writing, reviewing and editing, and investigation. SS: supervision, conceptualization, writing, reviewing, and editing. All authors contributed to the article and approved the submitted version.

## Conflict of Interest

The authors declare that the research was conducted in the absence of any commercial or financial relationships that could be construed as a potential conflict of interest.
