# Supplementary material for: Prediction of COVID-19 Waves Using Social Media and Google Search: A Case Study of the US and Canada
Source: Front Public Health. 2021 Apr 16;9:656635. doi: 10.3389/fpubh.2021.656635 (PMC8085269; doi:10.3389/fpubh.2021.656635)
Supplement: Supplementary file 1 [file Data_Sheet_2.PDF]

A Figures

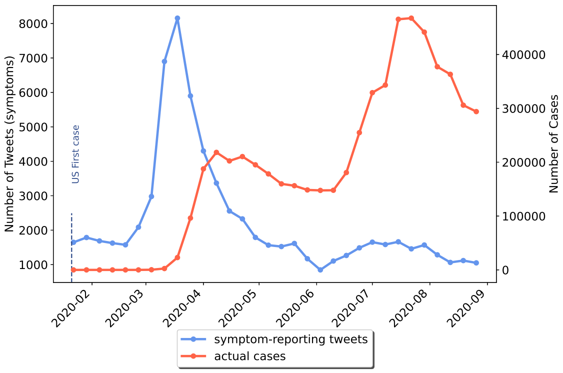

(a) Symptom-related tweets vs. cases

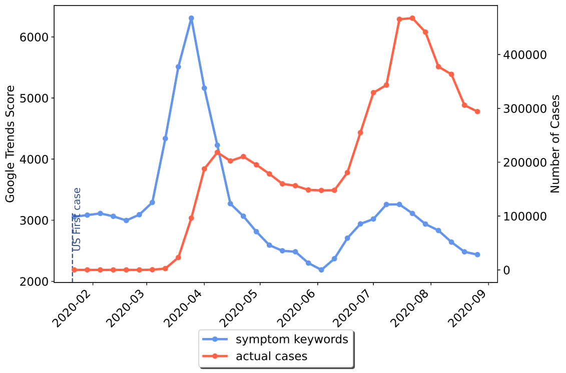

(b) Symptom-related searches vs. cases

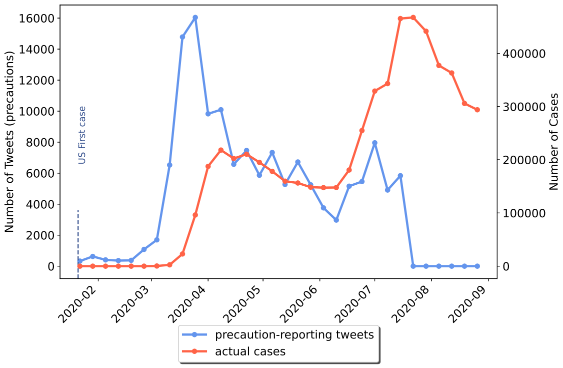

(c) Precaution-related tweets vs. cases

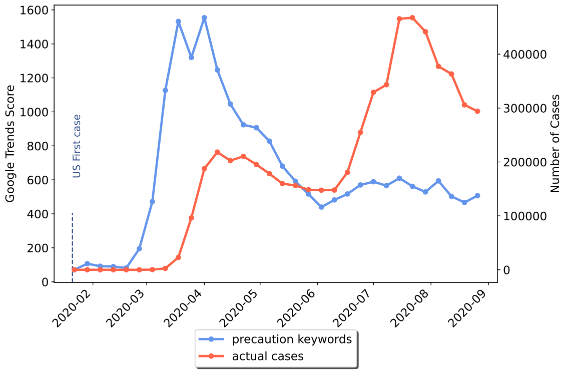

(d) Precaution-related searches vs. cases

Figure A1: Weekly comparison of online activities and actual number of cases in the United States

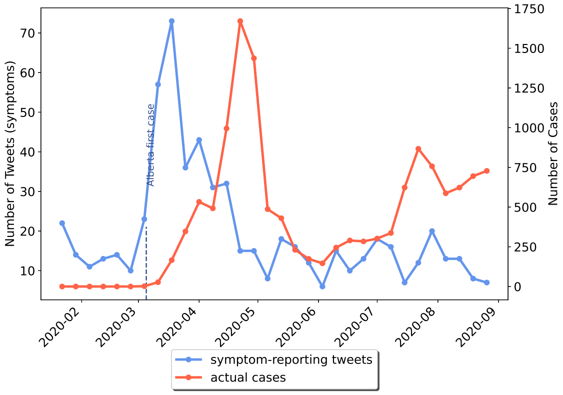

(a) Symptom-related tweets vs. cases

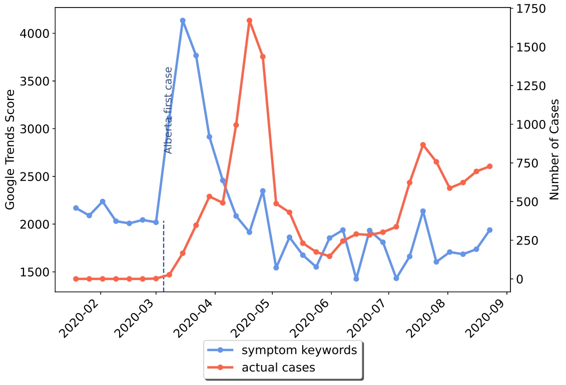

(b) Symptom-related searches vs. cases

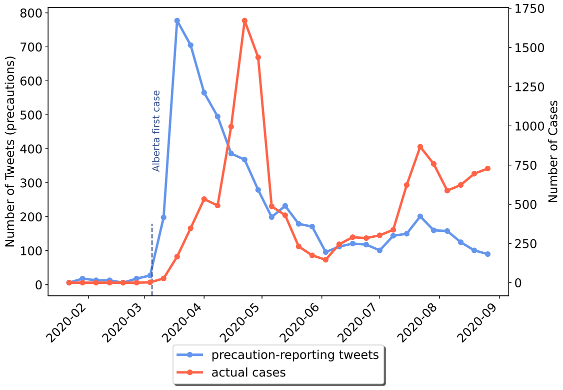

(c) Precaution-related tweets vs. cases

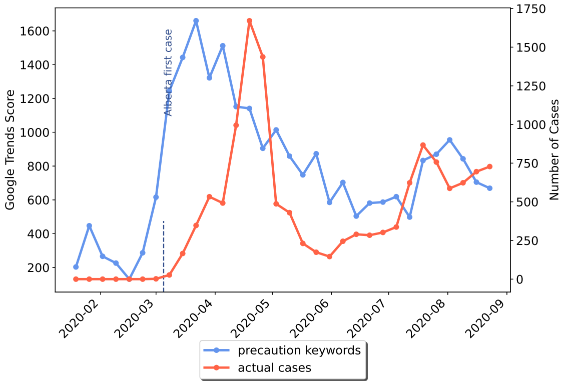

(d) Precaution-related searches vs. cases

Figure A2: Weekly comparison of online activities and actual number of cases in Alberta

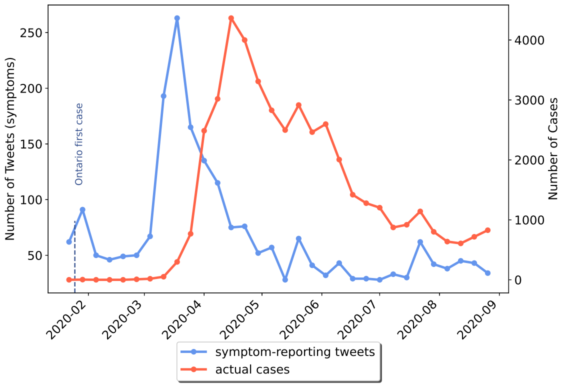

(a) Symptom-related tweets vs. cases

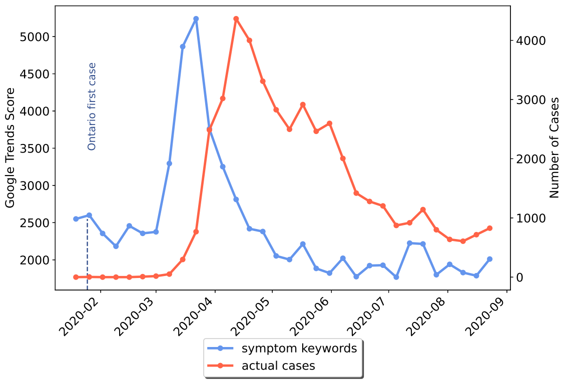

(b) Symptom-related searches vs. cases

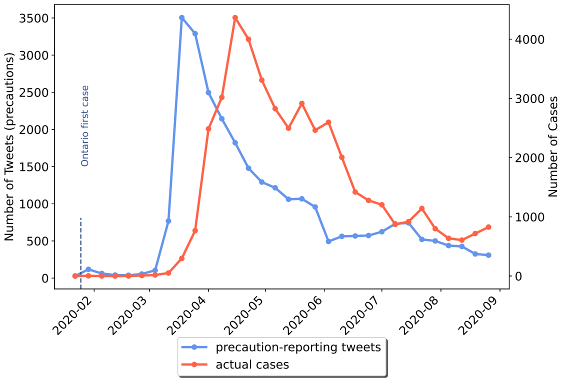

(c) Precaution-related tweets vs. cases

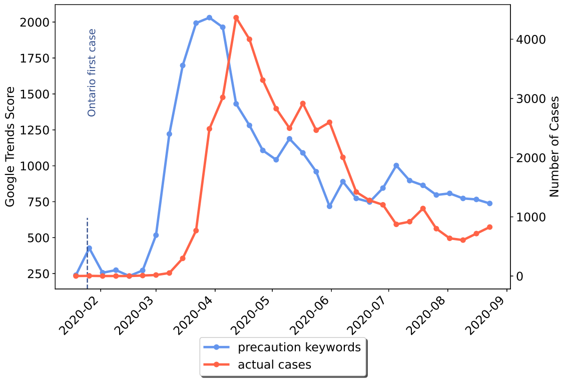

(d) Precaution-related searches vs. cases

Figure A3: Weekly comparison of online activities and actual number of cases in Ontario

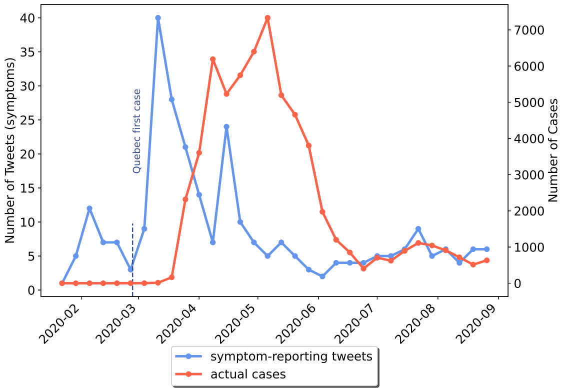

(a) Symptom-related tweets vs. cases

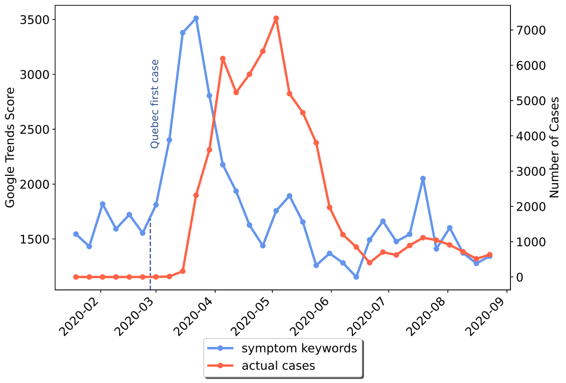

(b) Symptom-related searches vs. cases

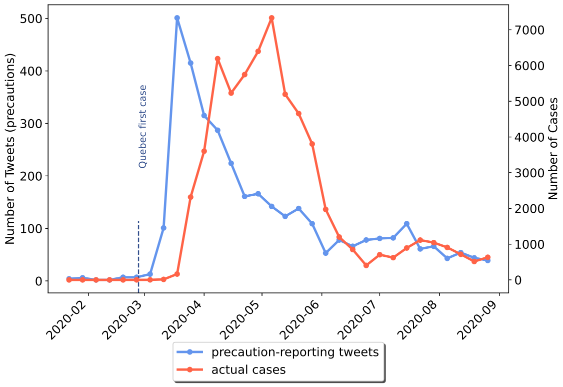

(c) Precaution-related tweets vs. cases

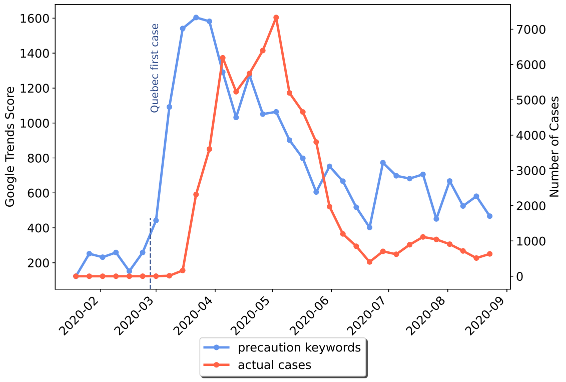

(d) Precaution-related searches vs. cases

Figure A4: Weekly comparison of online activities and actual number of cases in Quebec

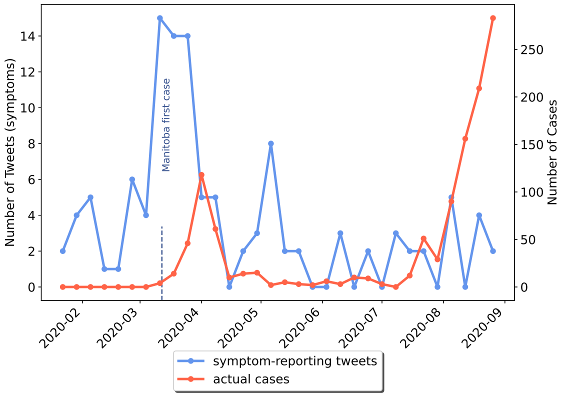

(a) Symptom-related tweets vs. cases

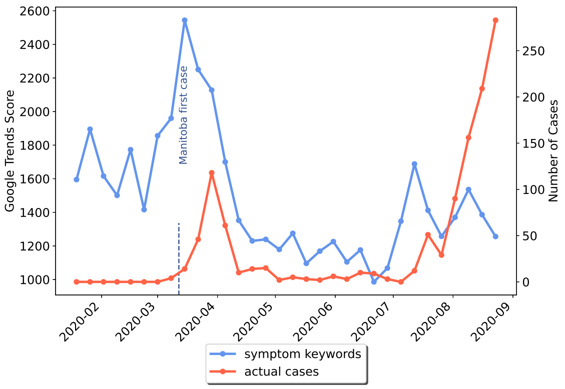

(b) Symptom-related searches vs. cases

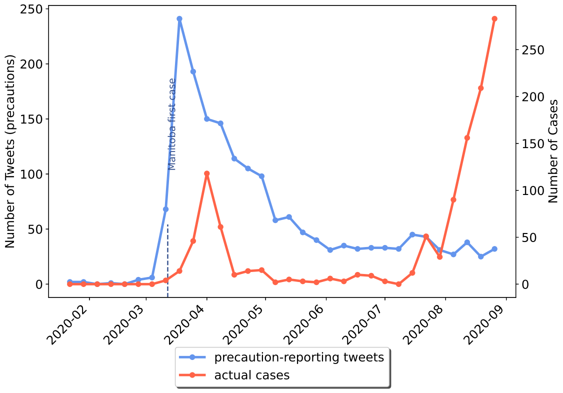

(c) Precaution-related tweets vs. cases

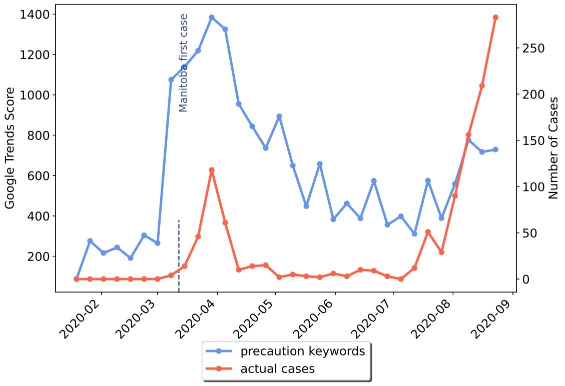

(d) Precaution-related searches vs. cases

Figure A5: Weekly comparison of online activities and actual number of cases in Manitoba

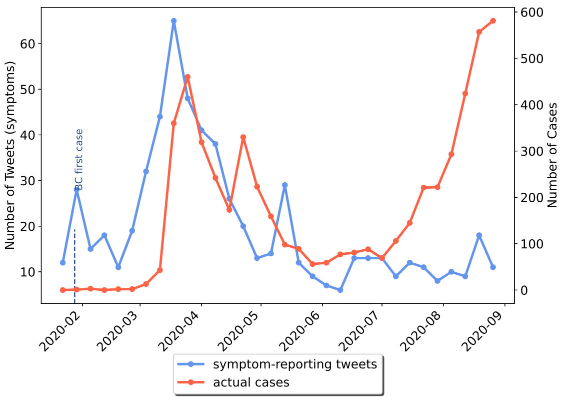

(a) Symptom-related tweets vs. cases

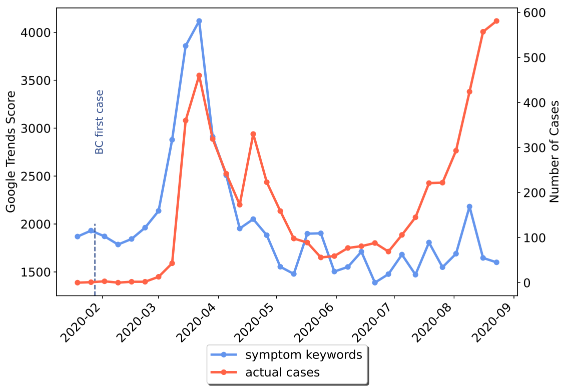

(b) Symptom-related searches vs. cases

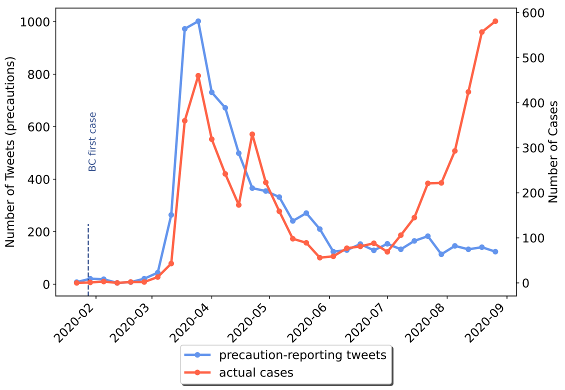

(c) Precaution-related tweets vs. cases

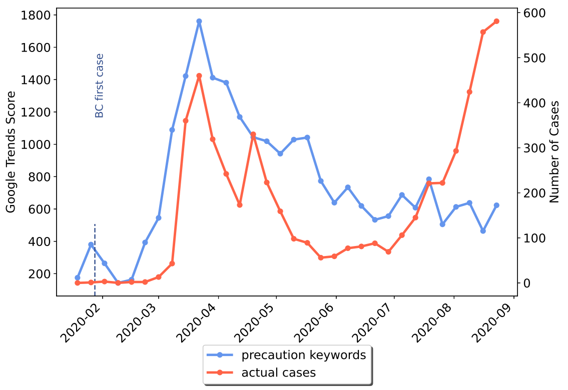

(d) Precaution-related searches vs. cases

Figure A6: Weekly comparison of online activities and actual number of cases in British Columbia

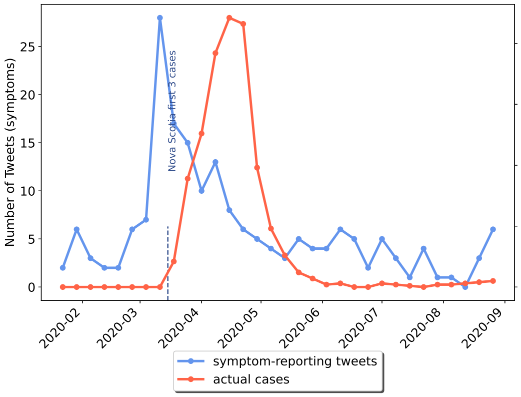

(a) Symptom-related tweets vs. cases

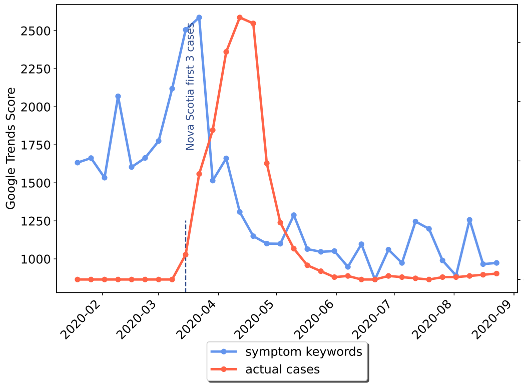

(b) Symptom-related searches vs. cases

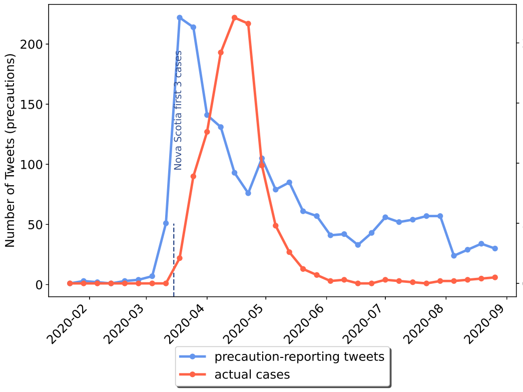

(c) Precaution-related tweets vs. cases

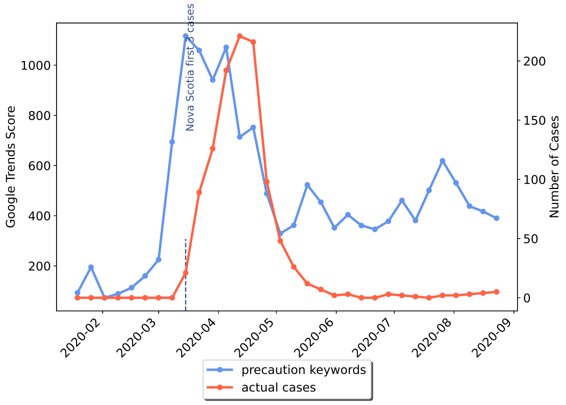

(d) Precaution-related searches vs. cases

Figure A7: Weekly comparison of online activities and actual number of cases in Nova Scotia

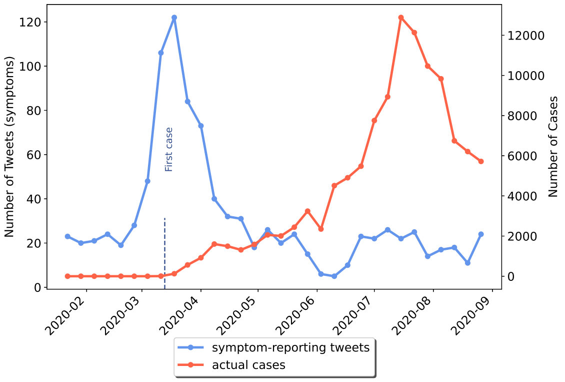

(a) Symptom-related tweets vs. cases

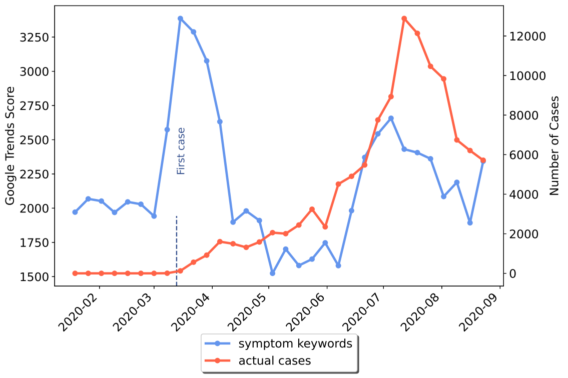

(b) Symptom-related searches vs. cases

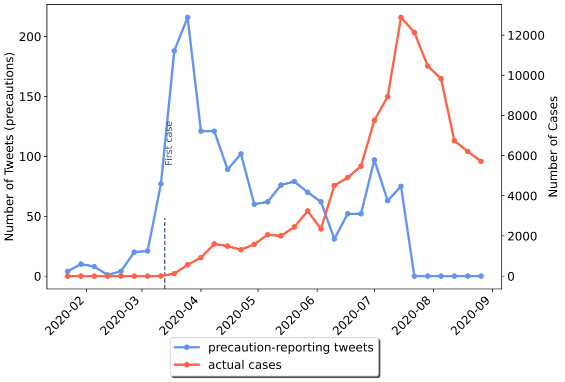

(c) Precaution-related tweets vs. cases

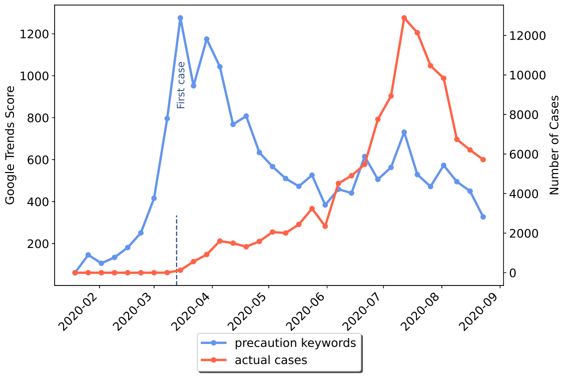

(d) Precaution-related searches vs. cases

Figure A8: Weekly comparison of online activities and actual number of cases in Alabama

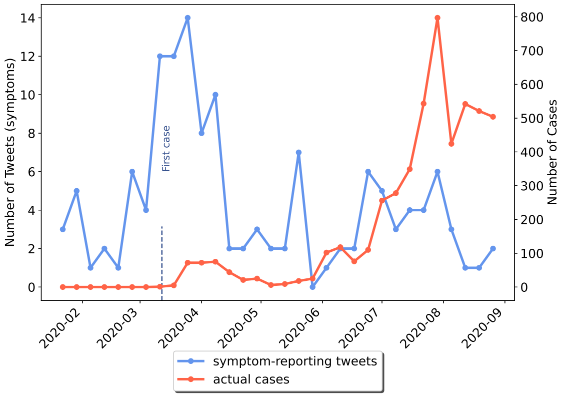

(a) Symptom-related tweets vs. cases

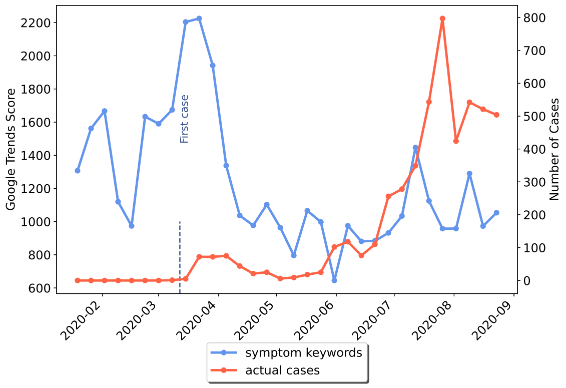

(b) Symptom-related searches vs. cases

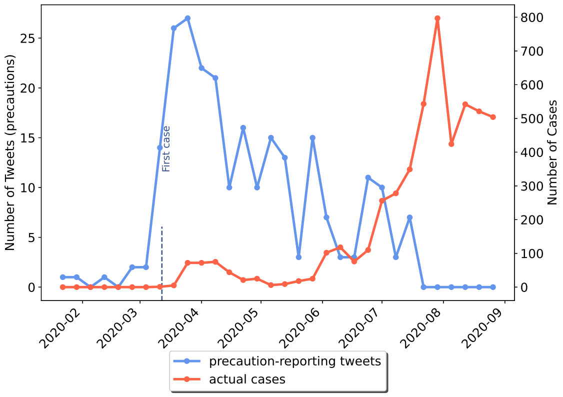

(c) Precaution-related tweets vs. cases

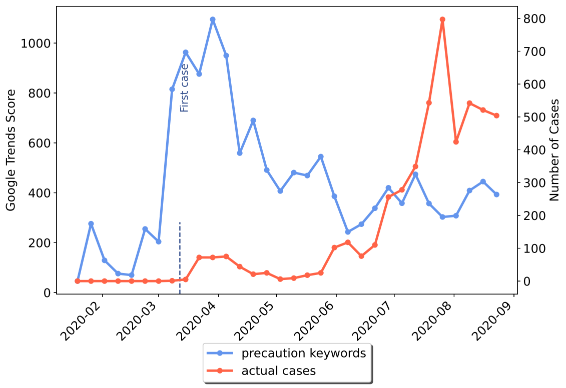

(d) Precaution-related searches vs. cases

Figure A9: Weekly comparison of online activities and actual number of cases in Alaska

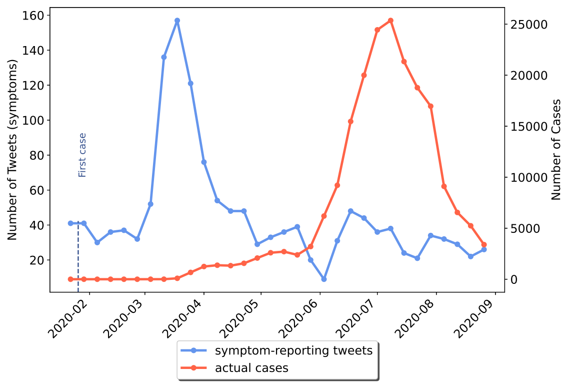

(a) Symptom-related tweets vs. cases

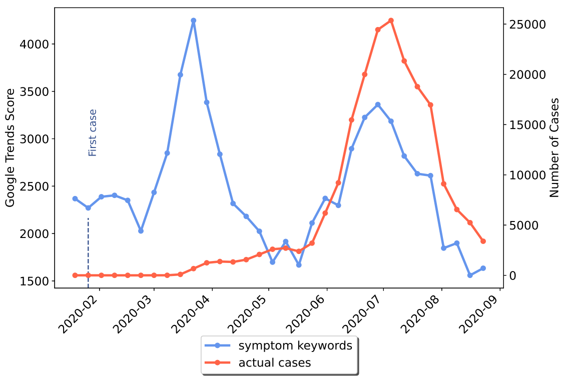

(b) Symptom-related searches vs. cases

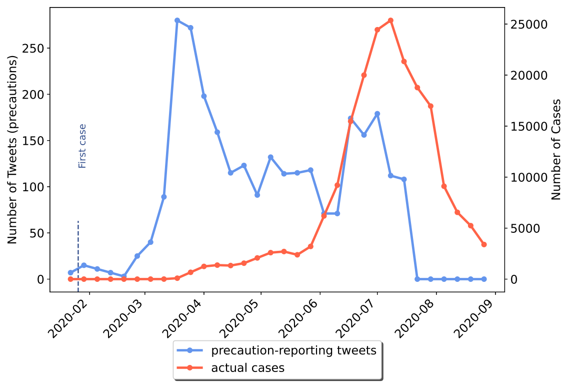

(c) Precaution-related tweets vs. cases

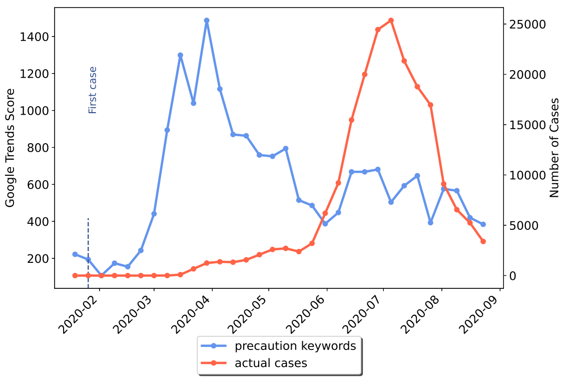

(d) Precaution-related searches vs. cases

Figure A10: Weekly comparison of online activities and actual number of cases in Arizona

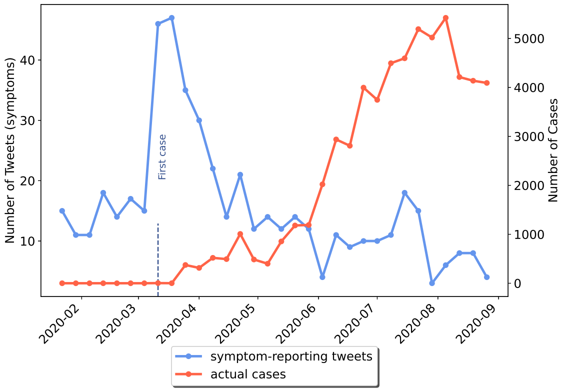

(a) Symptom-related tweets vs. cases

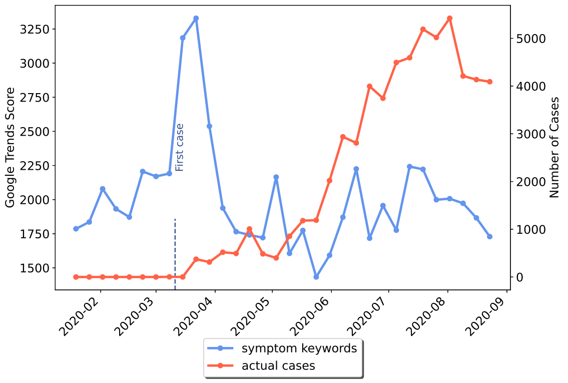

(b) Symptom-related searches vs. cases

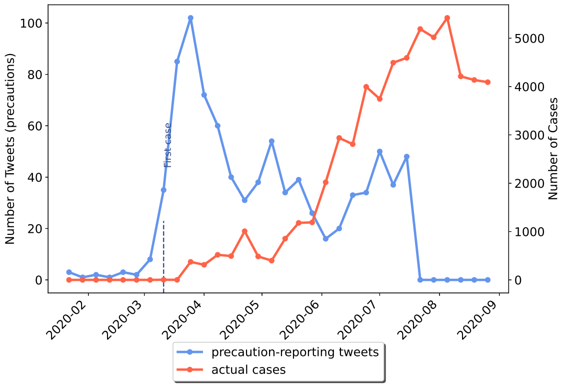

(c) Precaution-related tweets vs. cases

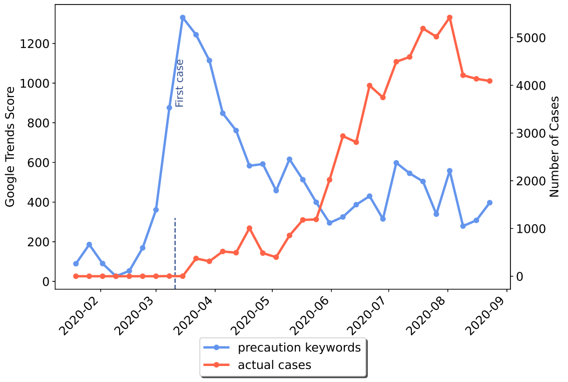

(d) Precaution-related searches vs. cases

Figure A11: Weekly comparison of online activities and actual number of cases in Arkansas

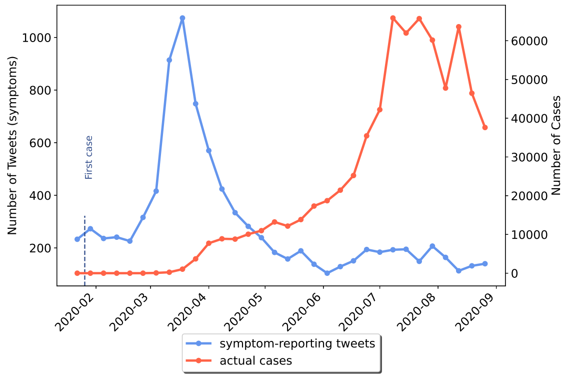

(a) Symptom-related tweets vs. cases

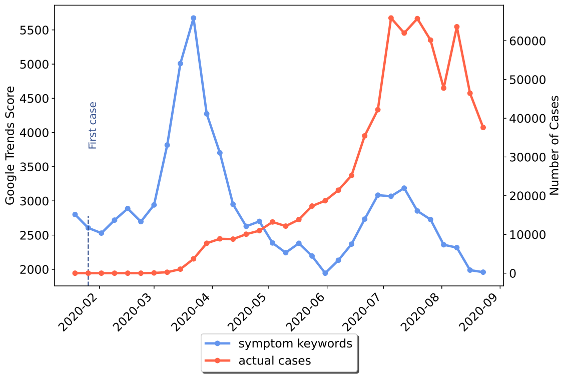

(b) Symptom-related searches vs. cases

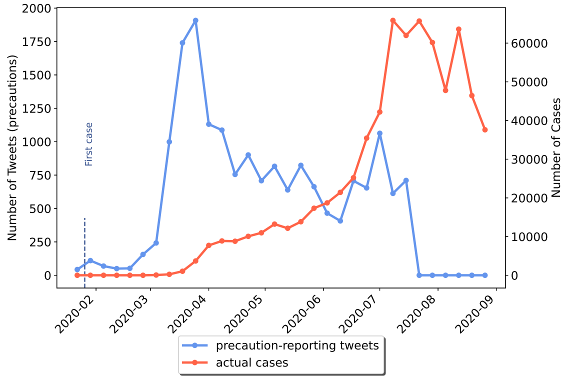

(c) Precaution-related tweets vs. cases

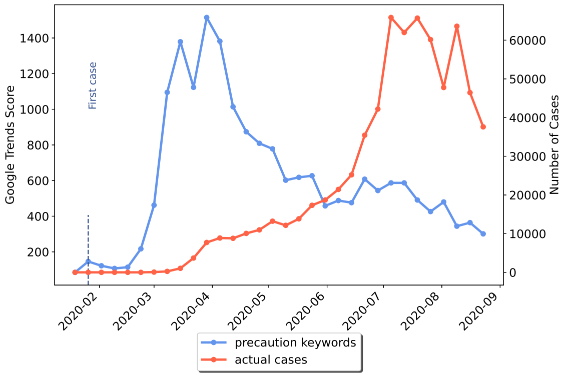

(d) Precaution-related searches vs. cases

Figure A12: Weekly comparison of online activities and actual number of cases in California

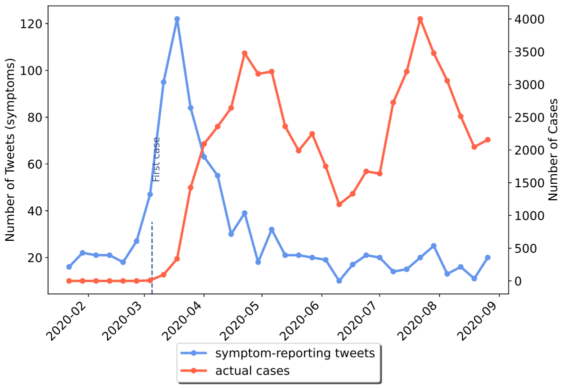

(a) Symptom-related tweets vs. cases

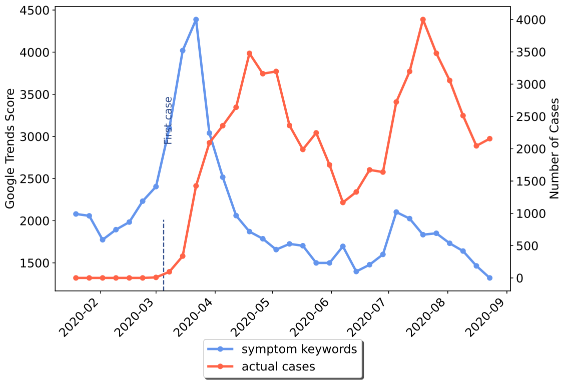

(b) Symptom-related searches vs. cases

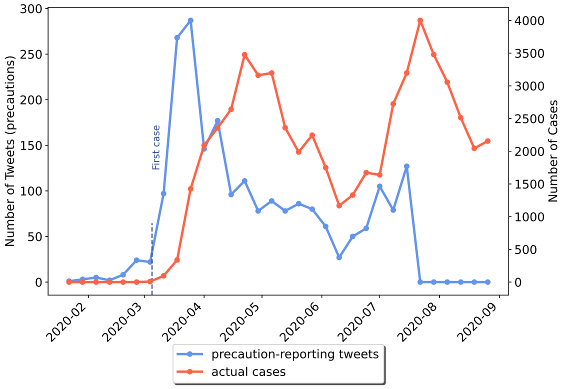

(c) Precaution-related tweets vs. cases

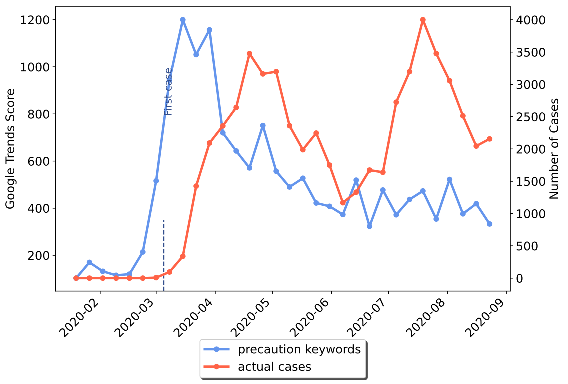

(d) Precaution-related searches vs. cases

Figure A13: Weekly comparison of online activities and actual number of cases in Colorado

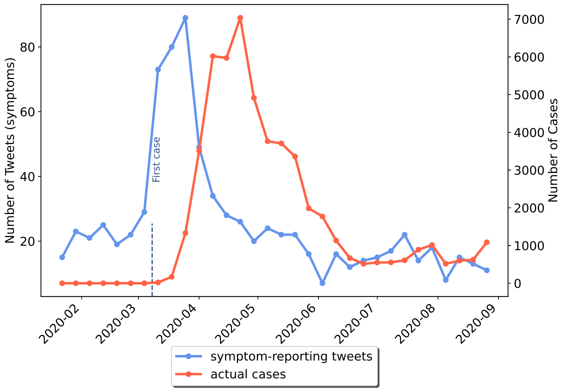

(a) Symptom-related tweets vs. cases

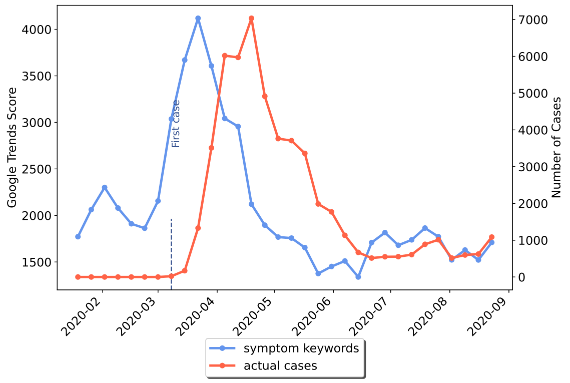

(b) Symptom-related searches vs. cases

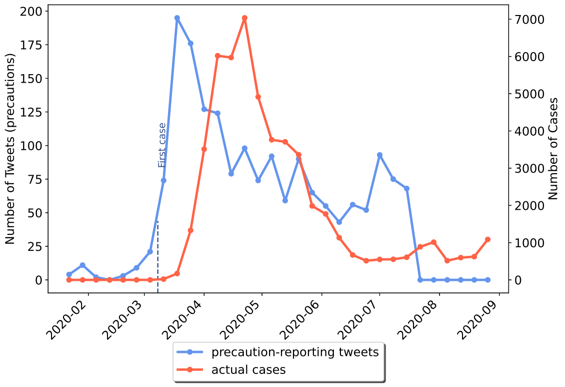

(c) Precaution-related tweets vs. cases

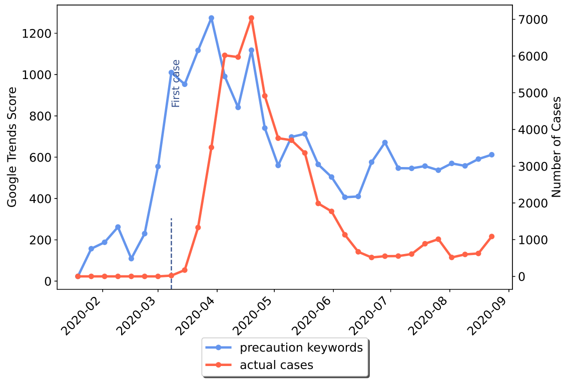

(d) Precaution-related searches vs. cases

Figure A14: Weekly comparison of online activities and actual number of cases in Connecticut

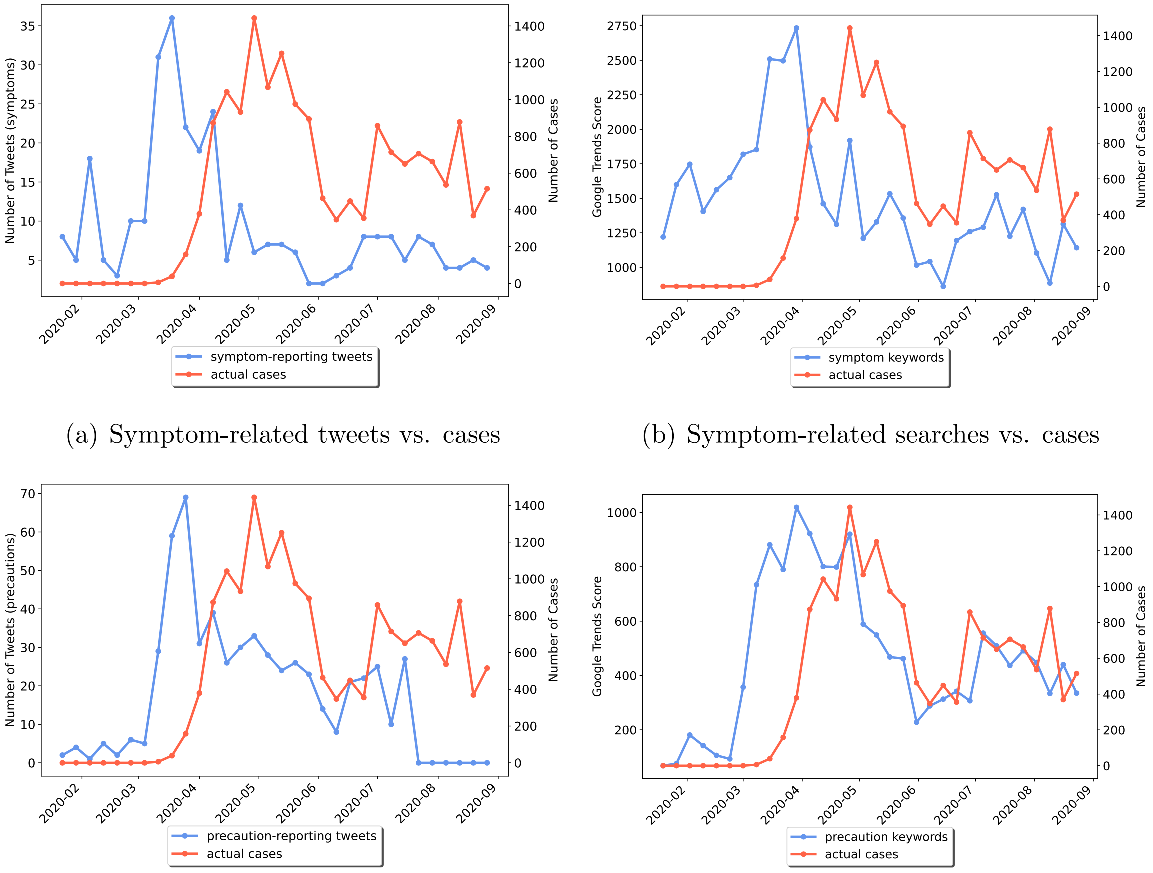

(a) Symptom-related tweets vs. cases

(b) Symptom-related searches vs. cases

(c) Precaution-related tweets vs. cases

(d) Precaution-related searches vs. cases

Figure A15: Weekly comparison of online activities and actual number of cases in Delaware

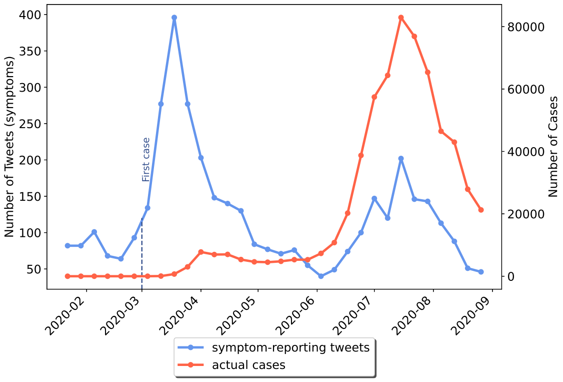

(a) Symptom-related tweets vs. cases

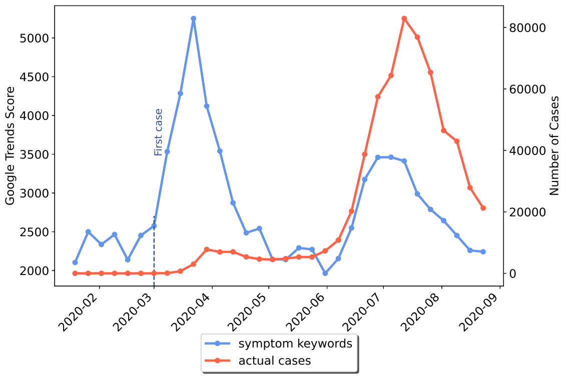

(b) Symptom-related searches vs. cases

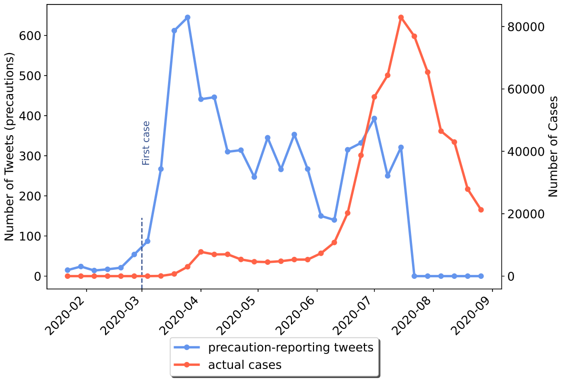

(c) Precaution-related tweets vs. cases

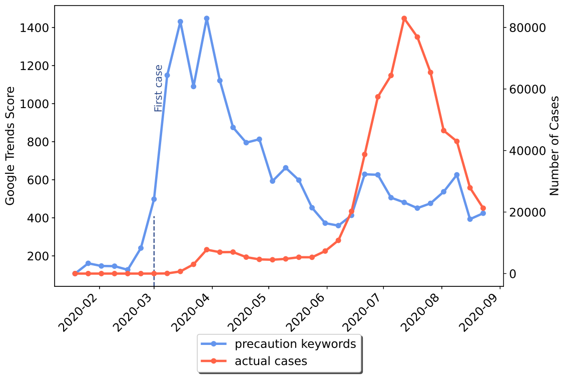

(d) Precaution-related searches vs. cases

Figure A16: Weekly comparison of online activities and actual number of cases in Florida

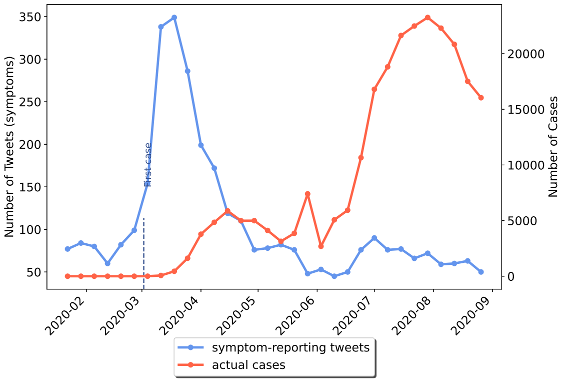

(a) Symptom-related tweets vs. cases

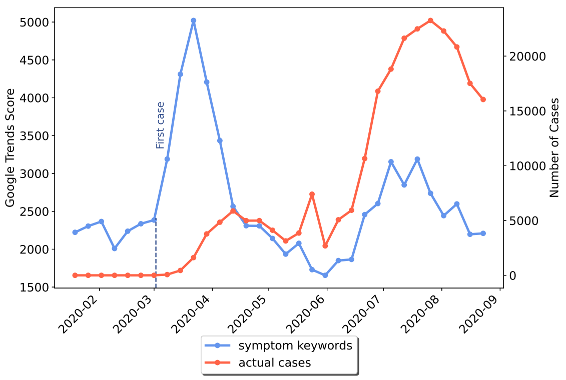

(b) Symptom-related searches vs. cases

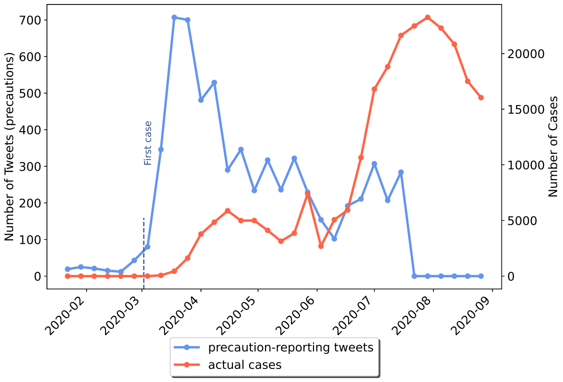

(c) Precaution-related tweets vs. cases

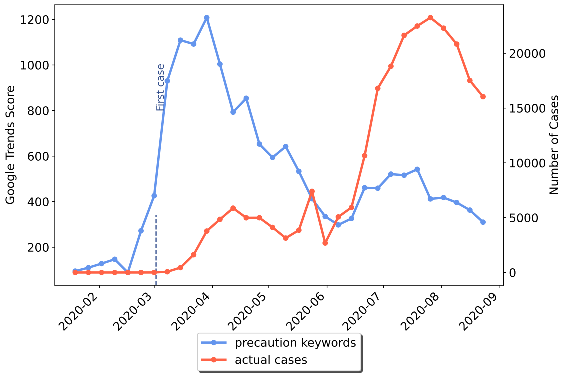

(d) Precaution-related searches vs. cases

Figure A17: Weekly comparison of online activities and actual number of cases in Georgia

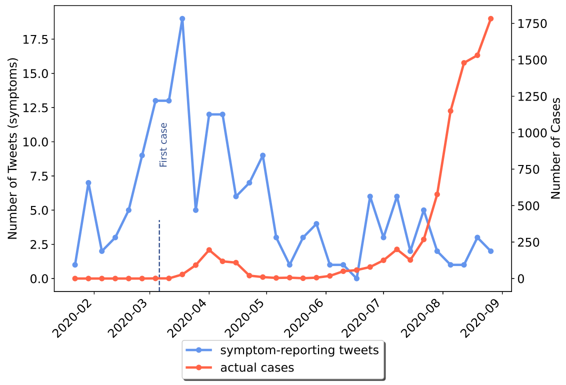

(a) Symptom-related tweets vs. cases

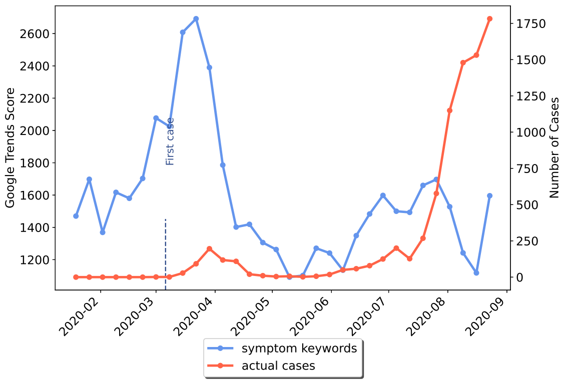

(b) Symptom-related searches vs. cases

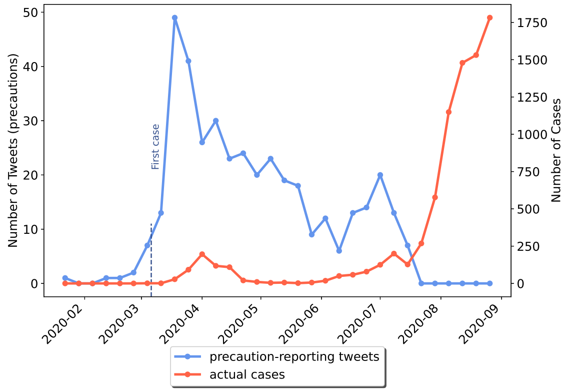

(c) Precaution-related tweets vs. cases

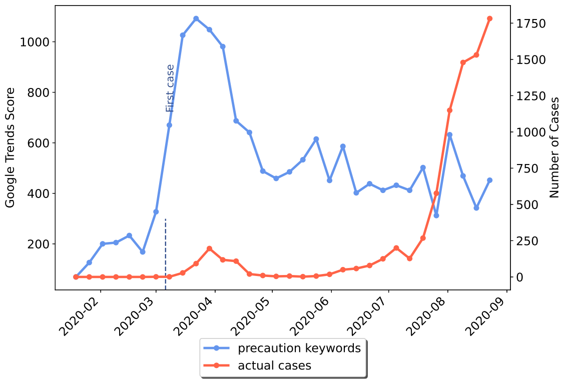

(d) Precaution-related searches vs. cases

Figure A18: Weekly comparison of online activities and actual number of cases in Hawaii

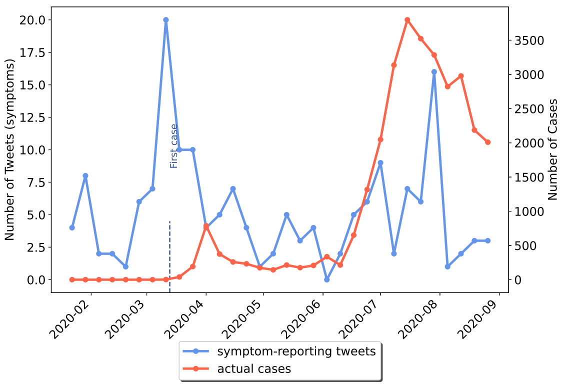

(a) Symptom-related tweets vs. cases

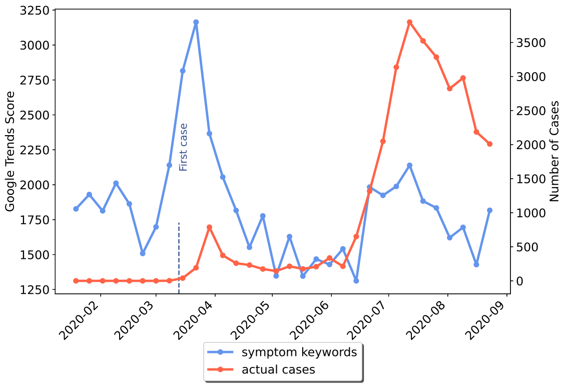

(b) Symptom-related searches vs. cases

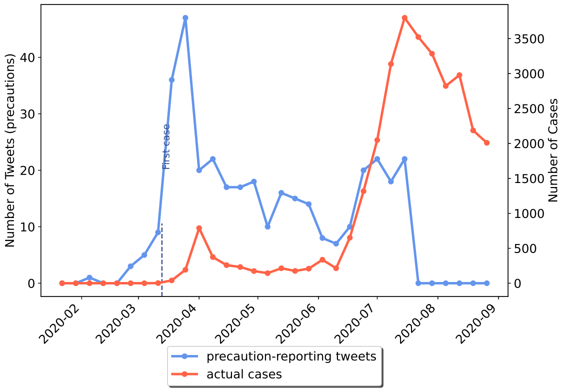

(c) Precaution-related tweets vs. cases

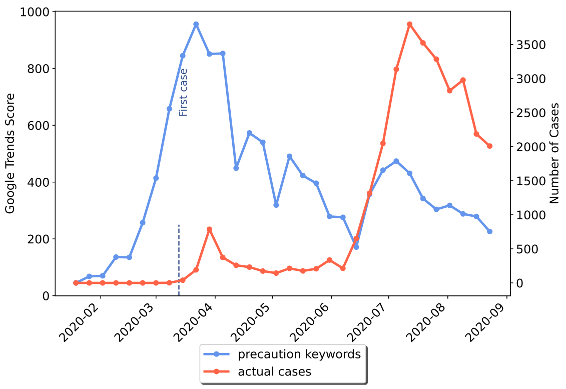

(d) Precaution-related searches vs. cases

Figure A19: Weekly comparison of online activities and actual number of cases in Idaho

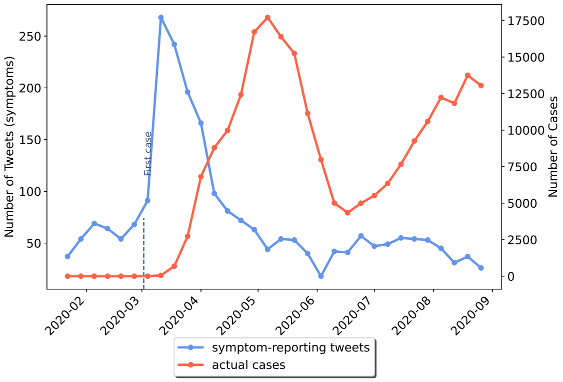

(a) Symptom-related tweets vs. cases

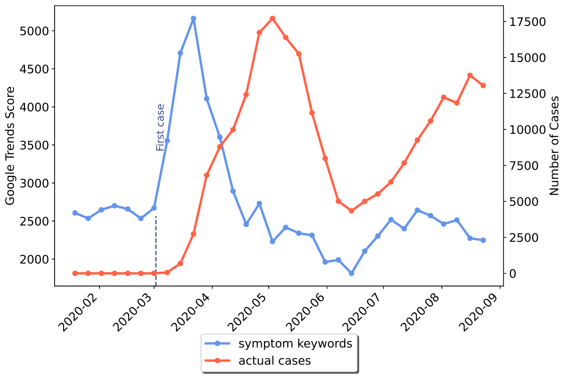

(b) Symptom-related searches vs. cases

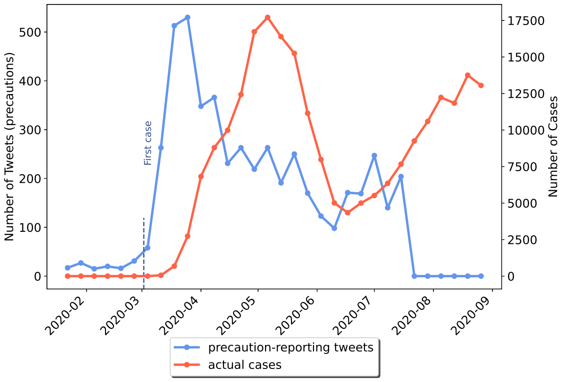

(c) Precaution-related tweets vs. cases

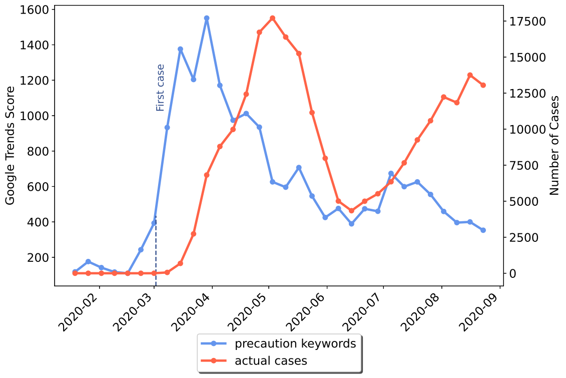

(d) Precaution-related searches vs. cases

Figure A20: Weekly comparison of online activities and actual number of cases in Illinois

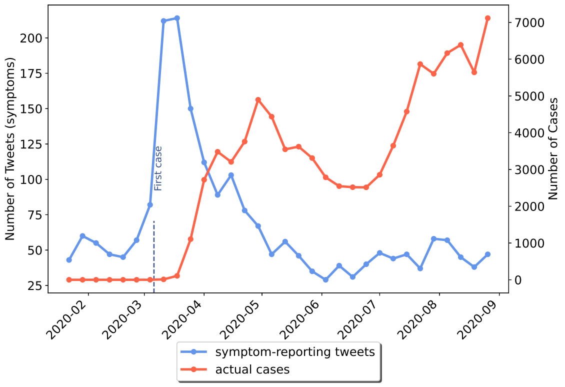

(a) Symptom-related tweets vs. cases

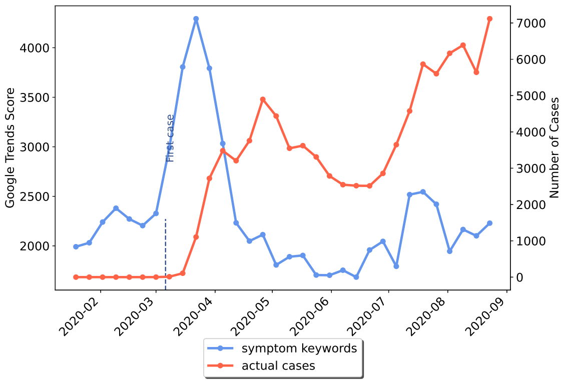

(b) Symptom-related searches vs. cases

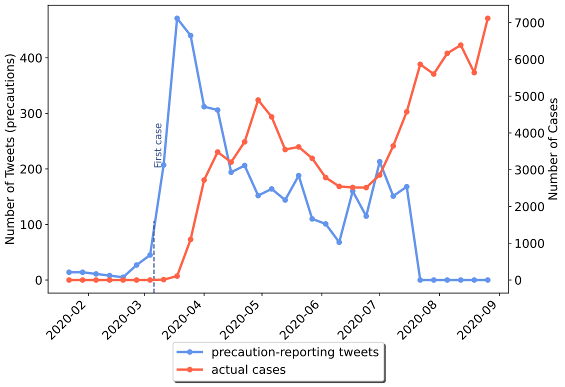

(c) Precaution-related tweets vs. cases

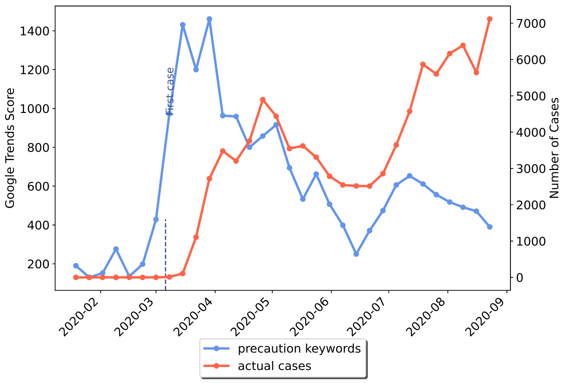

(d) Precaution-related searches vs. cases

Figure A21: Weekly comparison of online activities and actual number of cases in Indiana

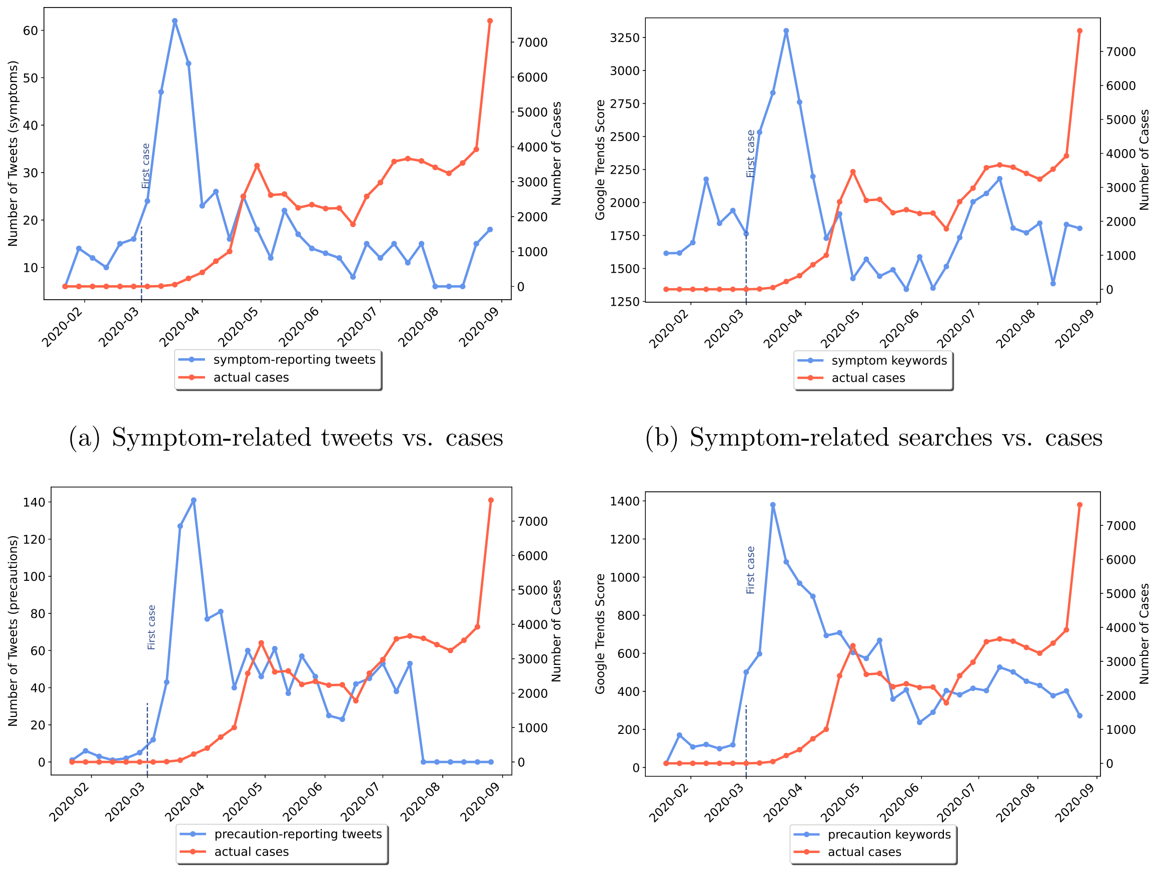

(a) Symptom-related tweets vs. cases

(b) Symptom-related searches vs. cases

(c) Precaution-related tweets vs. cases

(d) Precaution-related searches vs. cases

Figure A22: Weekly comparison of online activities and actual number of cases in Iowa

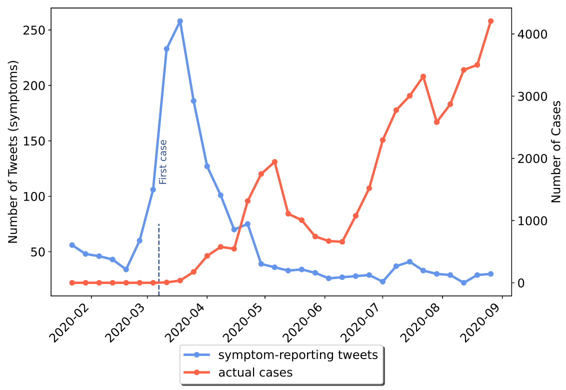

(a) Symptom-related tweets vs. cases

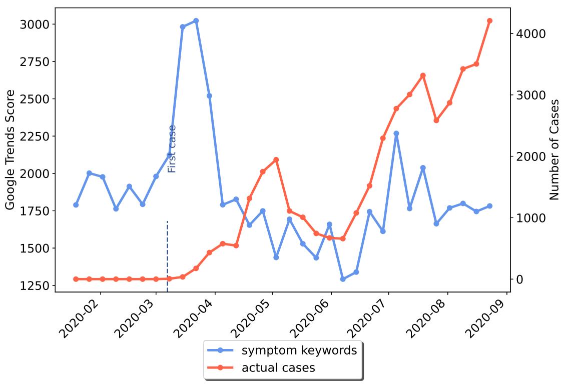

(b) Symptom-related searches vs. cases

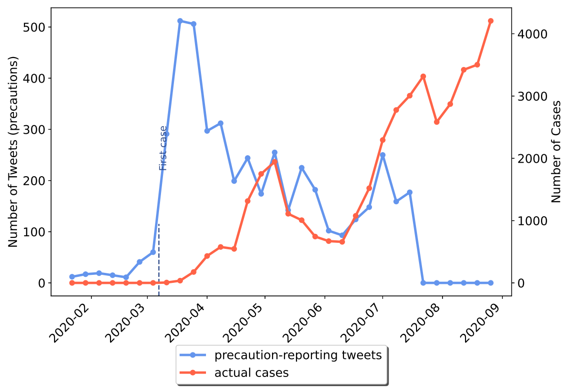

(c) Precaution-related tweets vs. cases

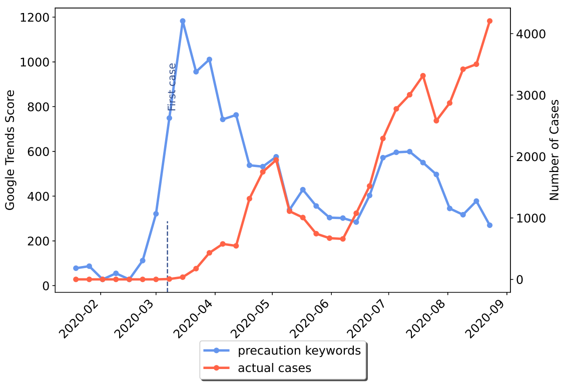

(d) Precaution-related searches vs. cases

Figure A23: Weekly comparison of online activities and actual number of cases in Kansas

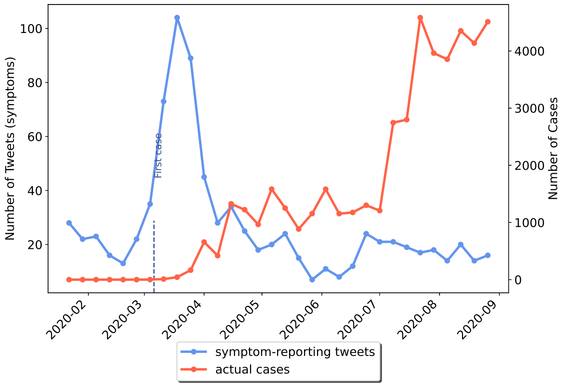

(a) Symptom-related tweets vs. cases

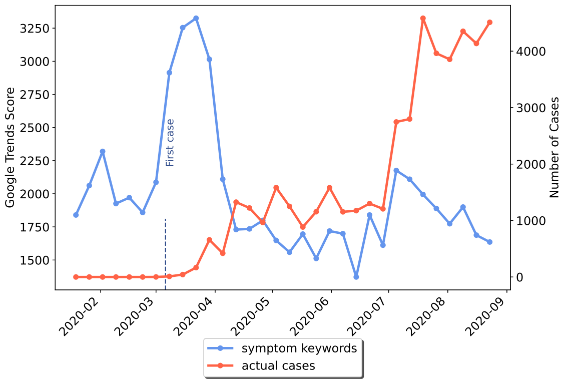

(b) Symptom-related searches vs. cases

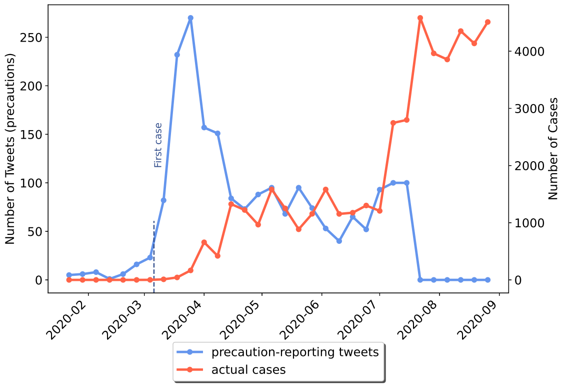

(c) Precaution-related tweets vs. cases

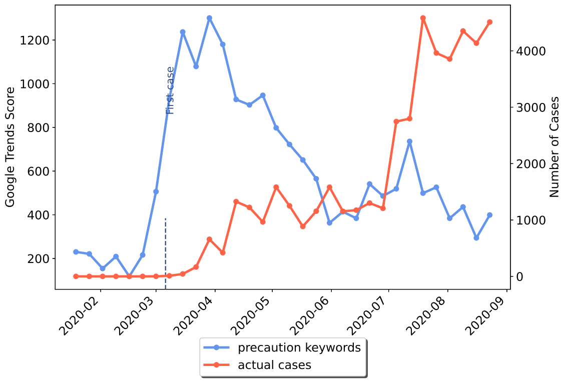

(d) Precaution-related searches vs. cases

Figure A24: Weekly comparison of online activities and actual number of cases in Kentucky

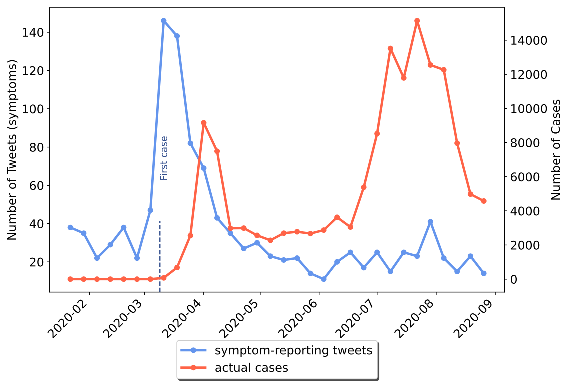

(a) Symptom-related tweets vs. cases

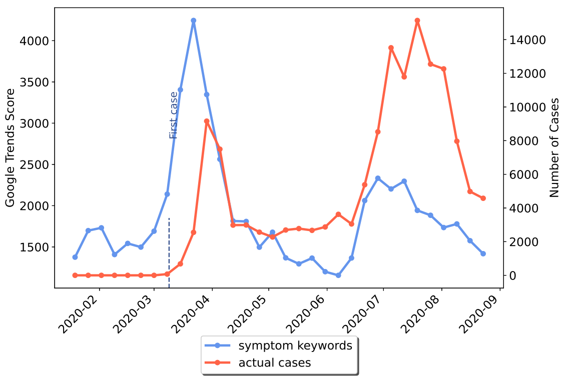

(b) Symptom-related searches vs. cases

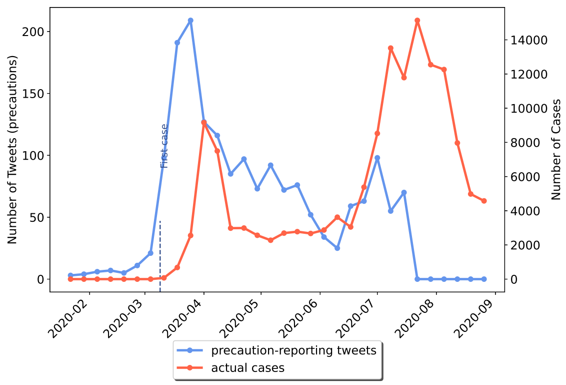

(c) Precaution-related tweets vs. cases

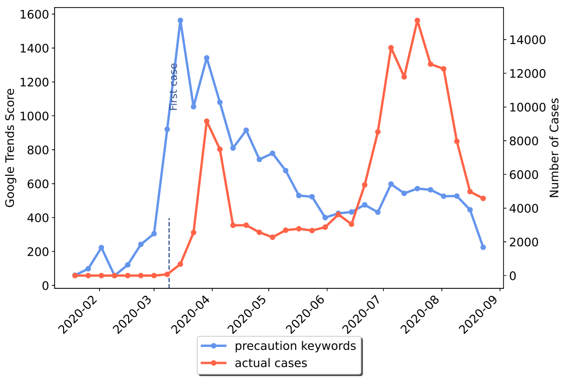

(d) Precaution-related searches vs. cases

Figure A25: Weekly comparison of online activities and actual number of cases in Louisiana

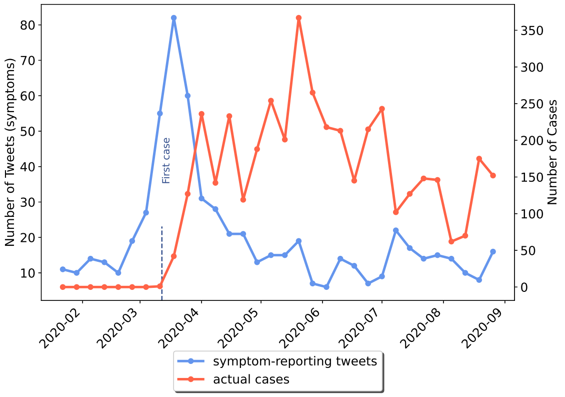

(a) Symptom-related tweets vs. cases

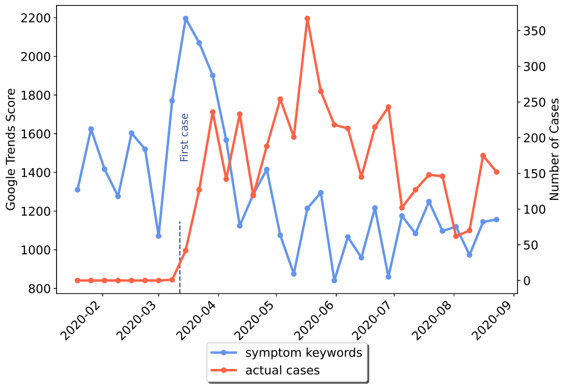

(b) Symptom-related searches vs. cases

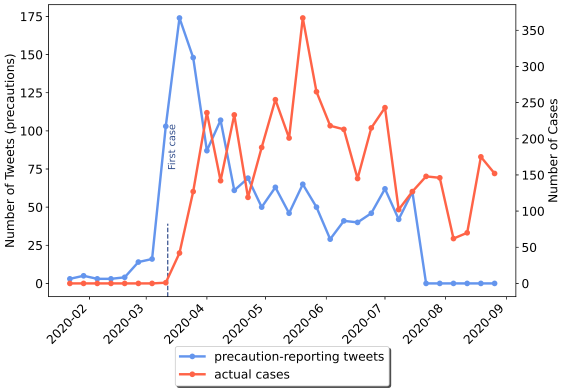

(c) Precaution-related tweets vs. cases

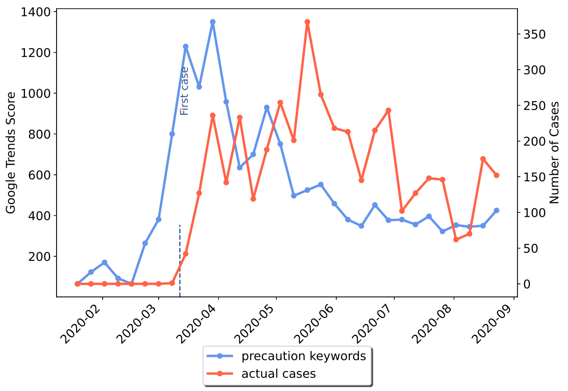

(d) Precaution-related searches vs. cases

Figure A26: Weekly comparison of online activities and actual number of cases in Maine

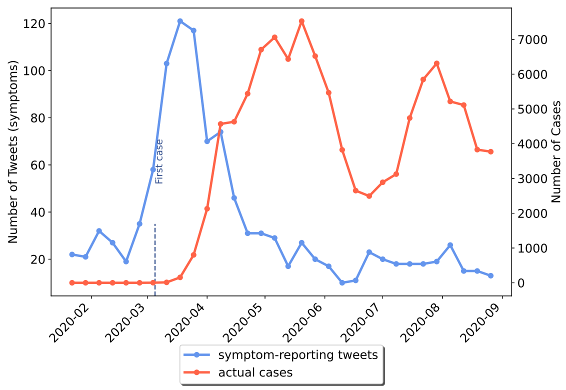

(a) Symptom-related tweets vs. cases

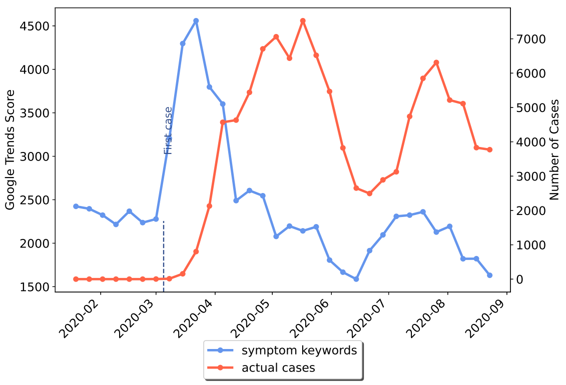

(b) Symptom-related searches vs. cases

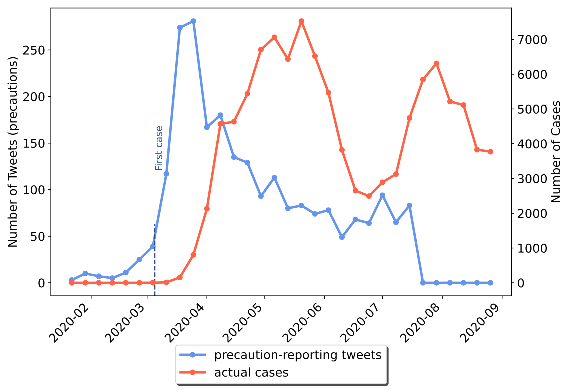

(c) Precaution-related tweets vs. cases

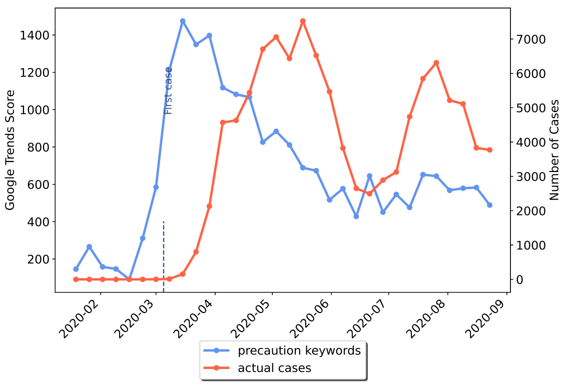

(d) Precaution-related searches vs. cases

Figure A27: Weekly comparison of online activities and actual number of cases in Maryland

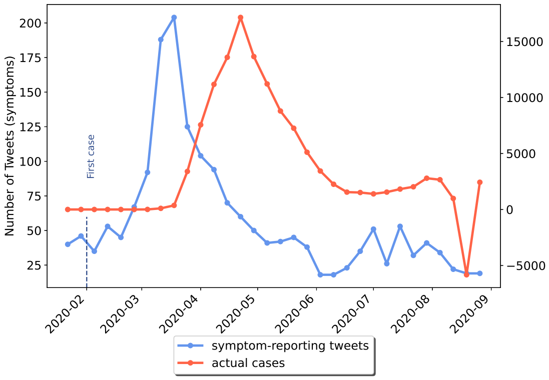

(a) Symptom-related tweets vs. cases

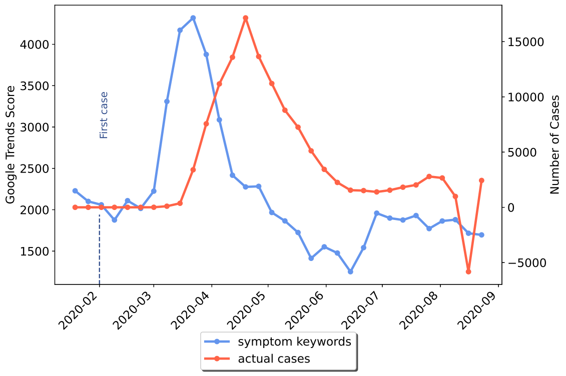

(b) Symptom-related searches vs. cases

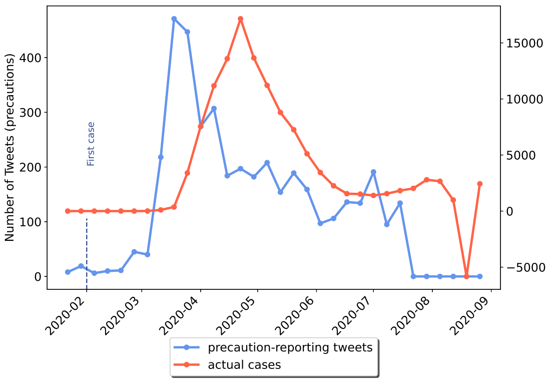

(c) Precaution-related tweets vs. cases

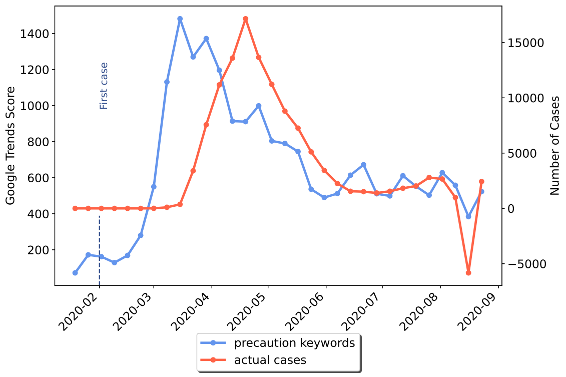

(d) Precaution-related searches vs. cases

Figure A28: Weekly comparison of online activities and actual number of cases in Massachusetts

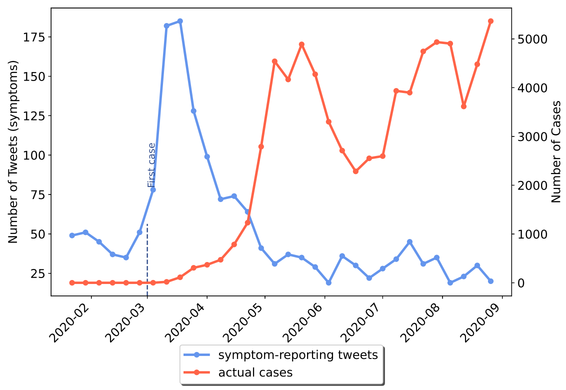

(a) Symptom-related tweets vs. cases

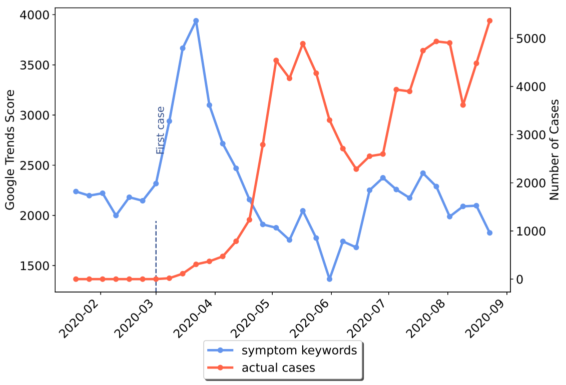

(b) Symptom-related searches vs. cases

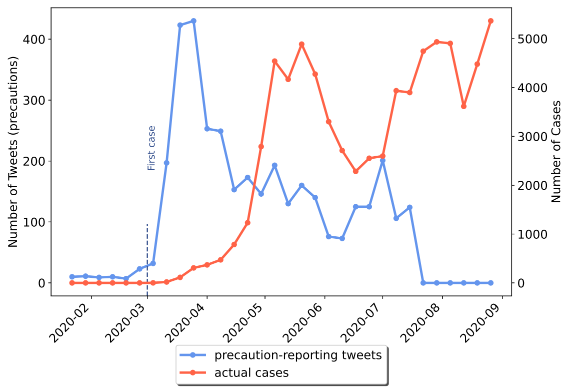

(c) Precaution-related tweets vs. cases

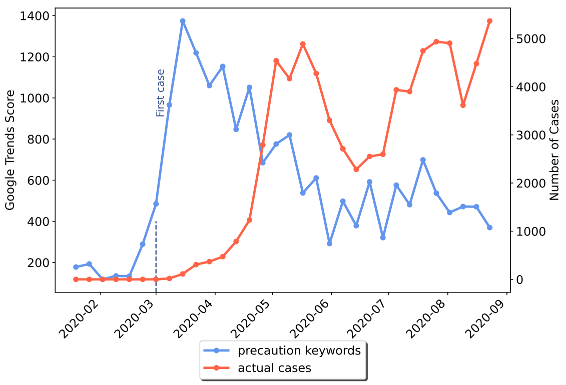

(d) Precaution-related searches vs. cases

Figure A29: Weekly comparison of online activities and actual number of cases in Minnesota

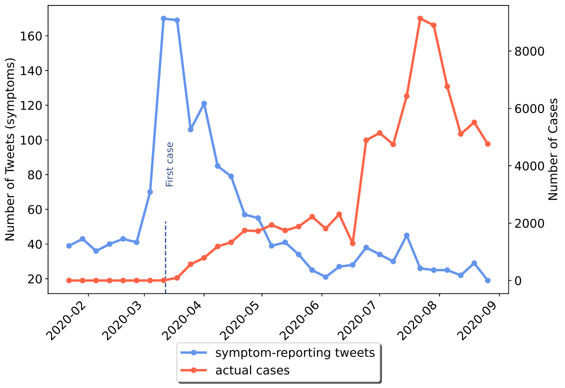

(a) Symptom-related tweets vs. cases

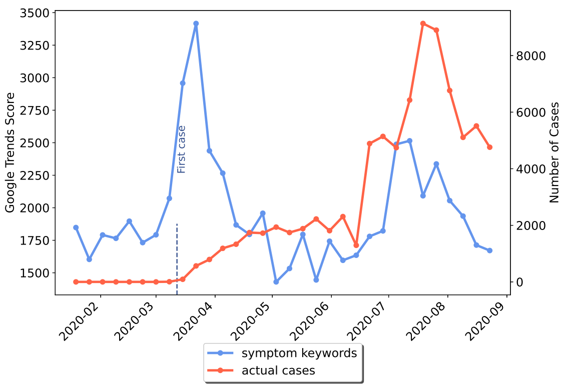

(b) Symptom-related searches vs. cases

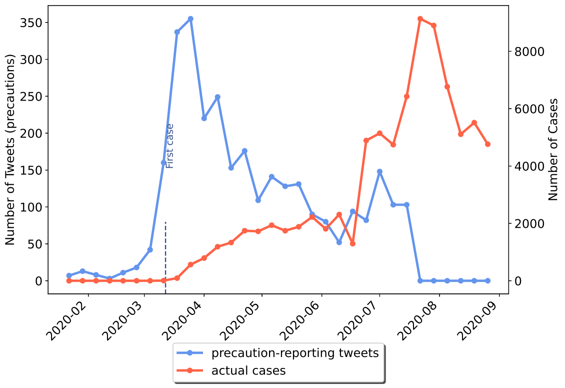

(c) Precaution-related tweets vs. cases

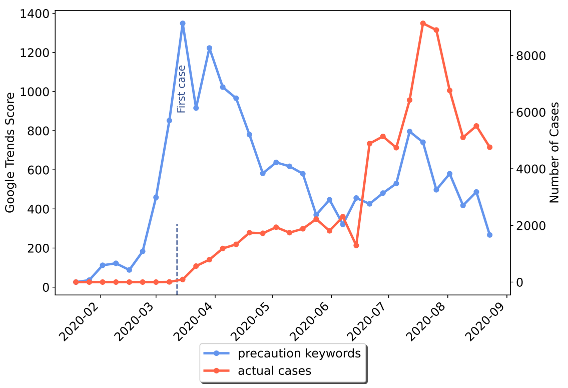

(d) Precaution-related searches vs. cases

Figure A30: Weekly comparison of online activities and actual number of cases in Mississippi

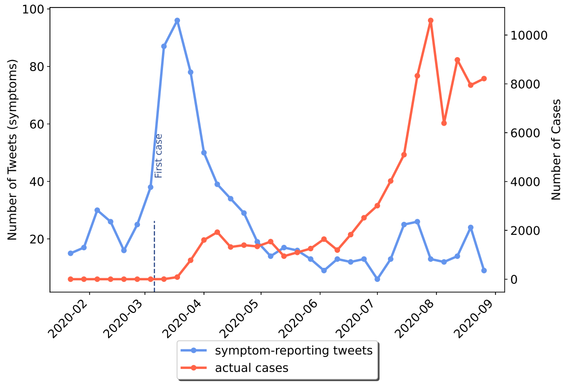

(a) Symptom-related tweets vs. cases

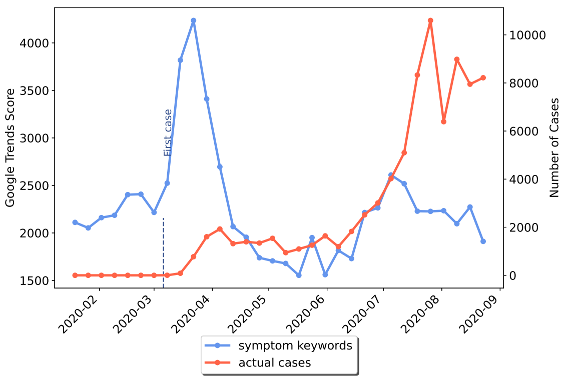

(b) Symptom-related searches vs. cases

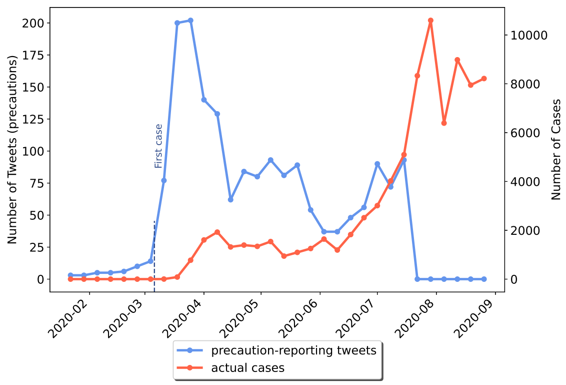

(c) Precaution-related tweets vs. cases

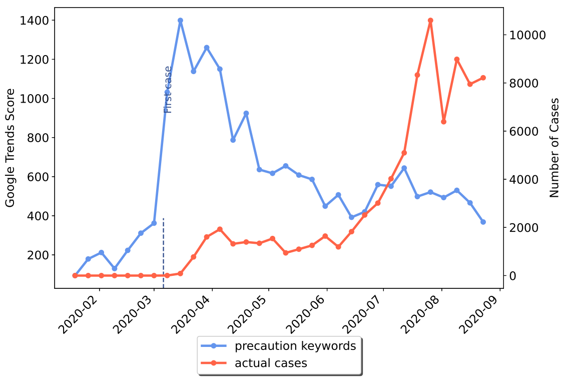

(d) Precaution-related searches vs. cases

Figure A31: Weekly comparison of online activities and actual number of cases in Missouri

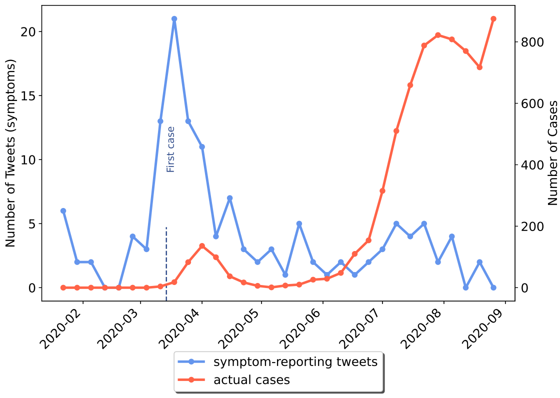

(a) Symptom-related tweets vs. cases

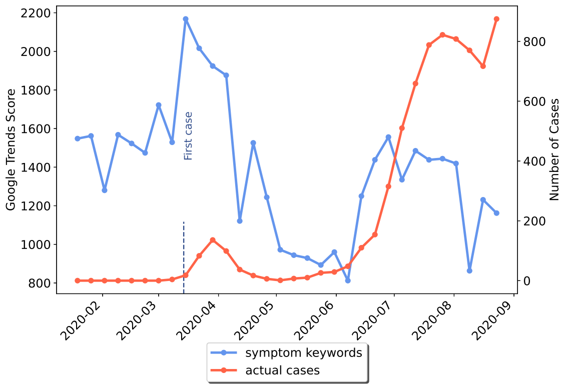

(b) Symptom-related searches vs. cases

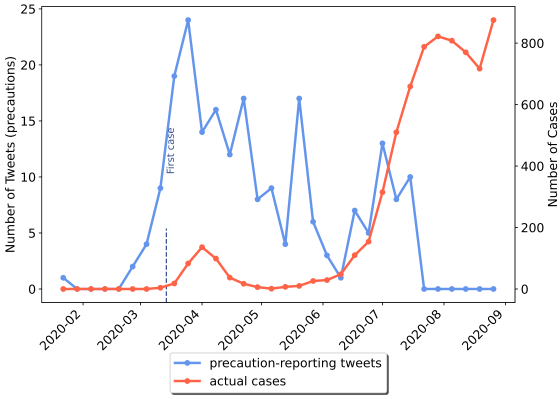

(c) Precaution-related tweets vs. cases

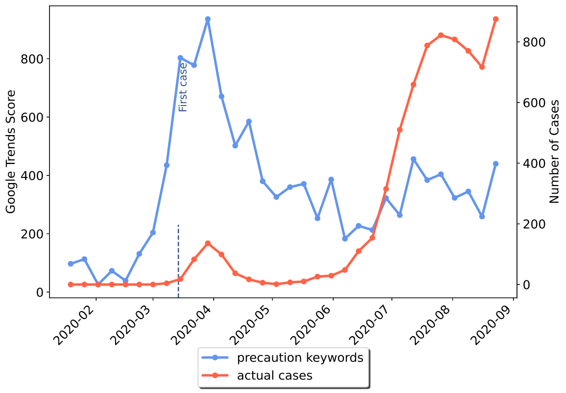

(d) Precaution-related searches vs. cases

Figure A32: Weekly comparison of online activities and actual number of cases in Montana

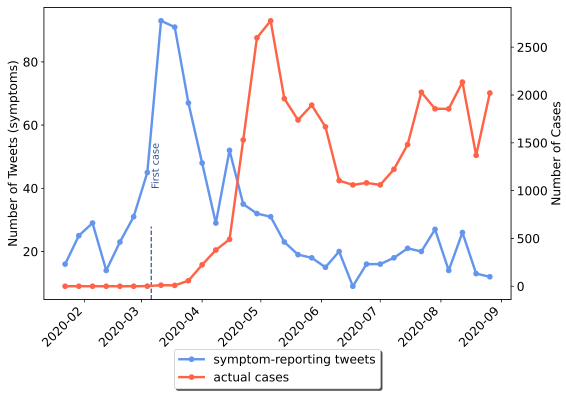

(a) Symptom-related tweets vs. cases

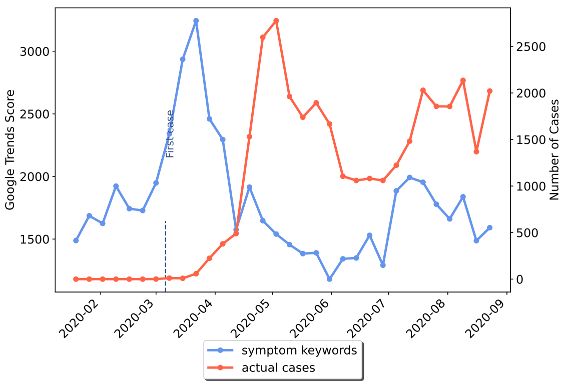

(b) Symptom-related searches vs. cases

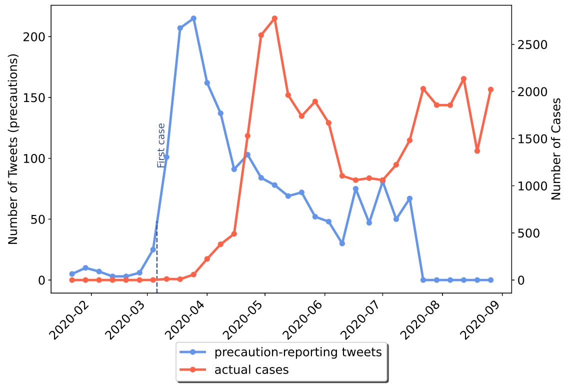

(c) Precaution-related tweets vs. cases

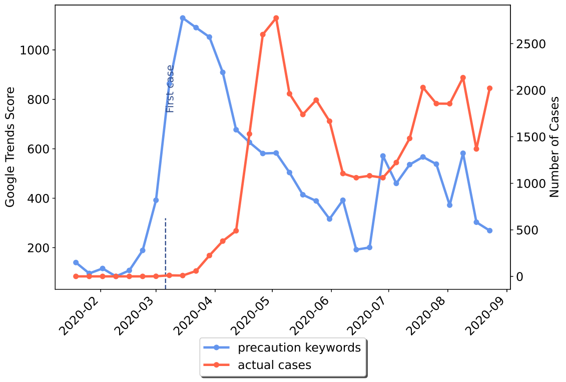

(d) Precaution-related searches vs. cases

Figure A33: Weekly comparison of online activities and actual number of cases in Nebraska

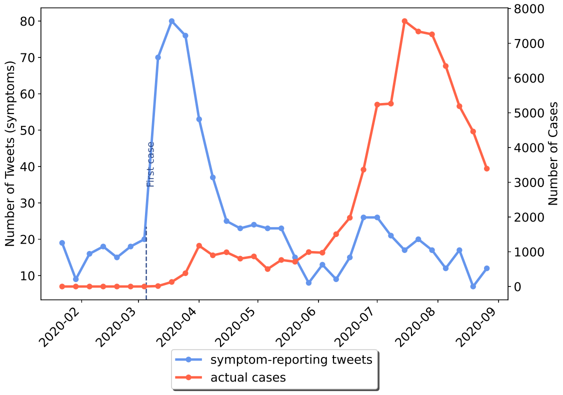

(a) Symptom-related tweets vs. cases

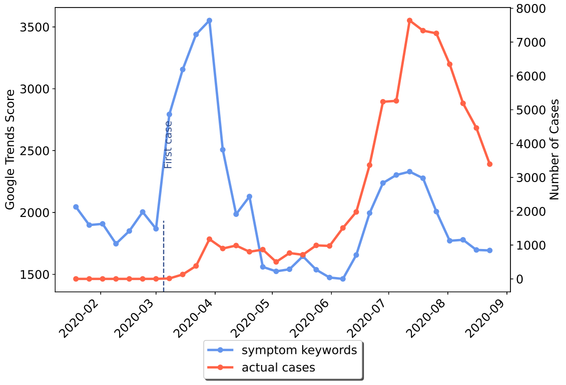

(b) Symptom-related searches vs. cases

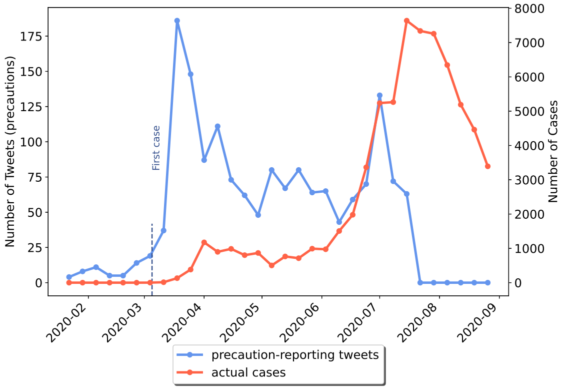

(c) Precaution-related tweets vs. cases

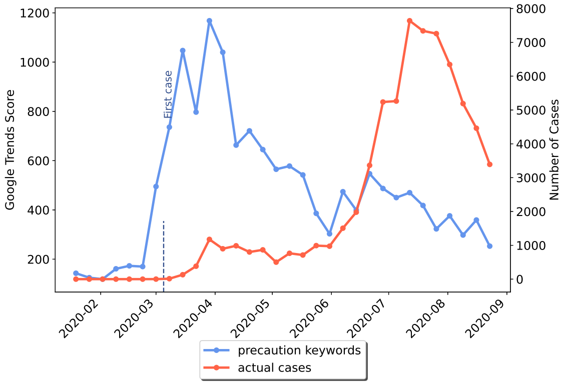

(d) Precaution-related searches vs. cases

Figure A34: Weekly comparison of online activities and actual number of cases in Nevada

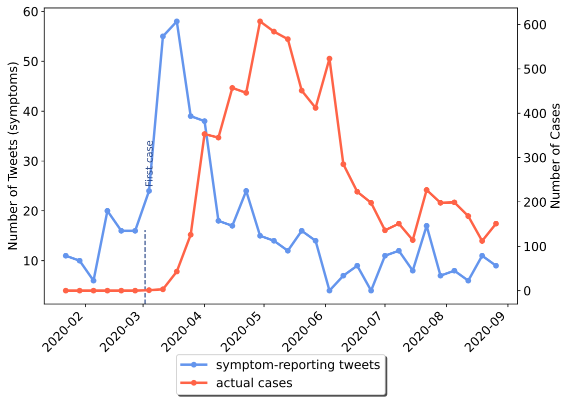

(a) Symptom-related tweets vs. cases

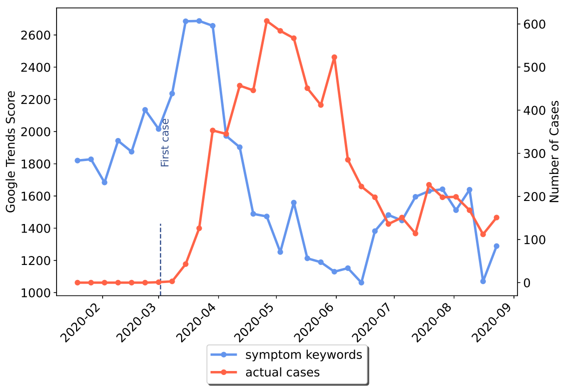

(b) Symptom-related searches vs. cases

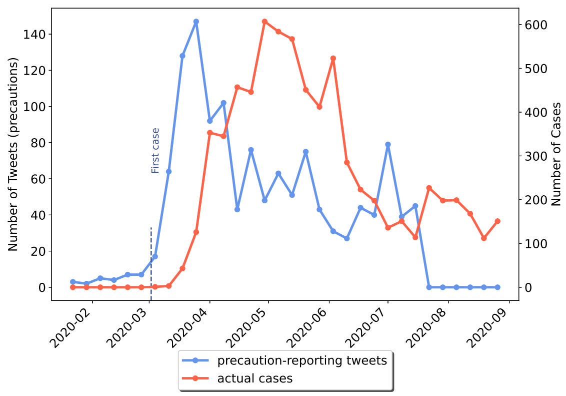

(c) Precaution-related tweets vs. cases

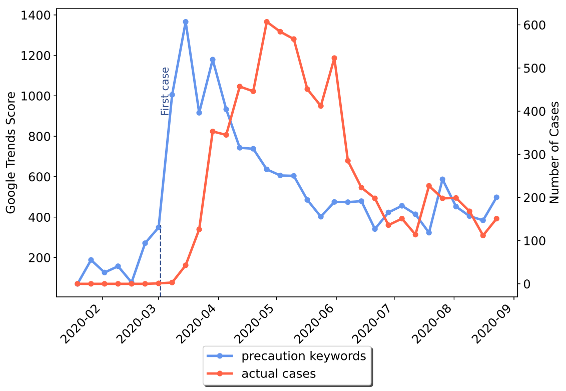

(d) Precaution-related searches vs. cases

Figure A35: Weekly comparison of online activities and actual number of cases in New Hampshire

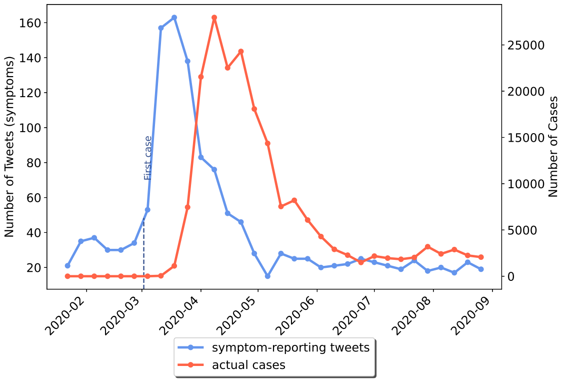

(a) Symptom-related tweets vs. cases

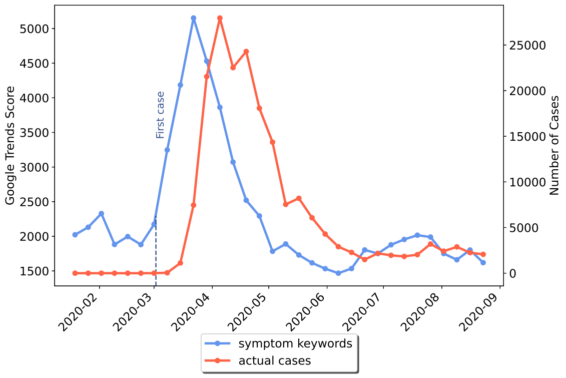

(b) Symptom-related searches vs. cases

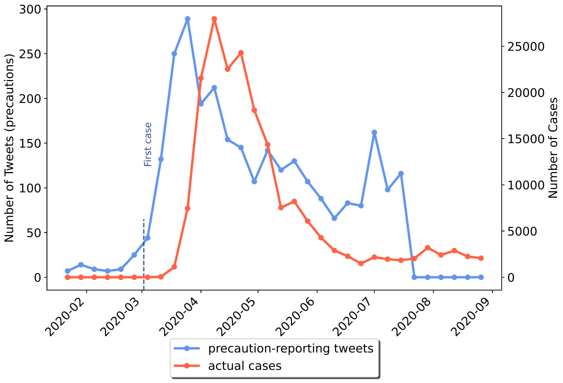

(c) Precaution-related tweets vs. cases

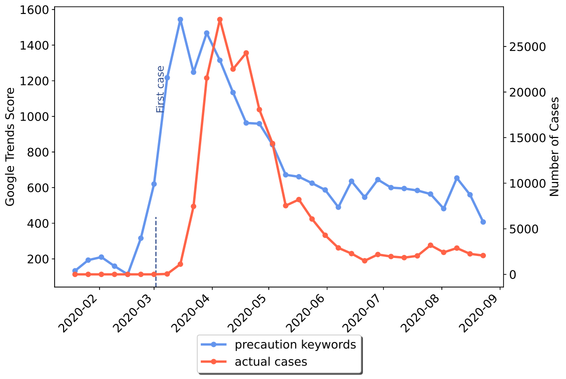

(d) Precaution-related searches vs. cases

Figure A36: Weekly comparison of online activities and actual number of cases in New Jersey

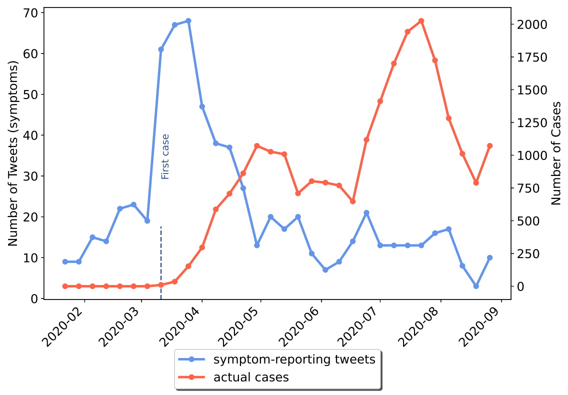

(a) Symptom-related tweets vs. cases

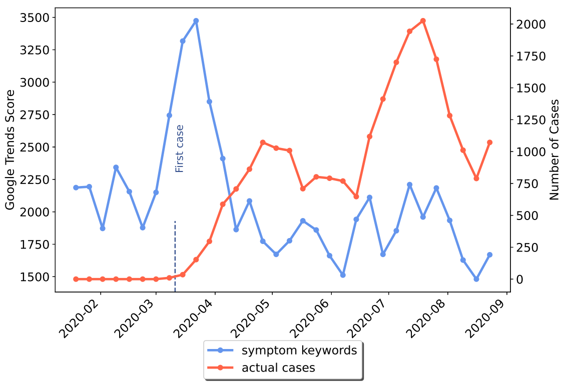

(b) Symptom-related searches vs. cases

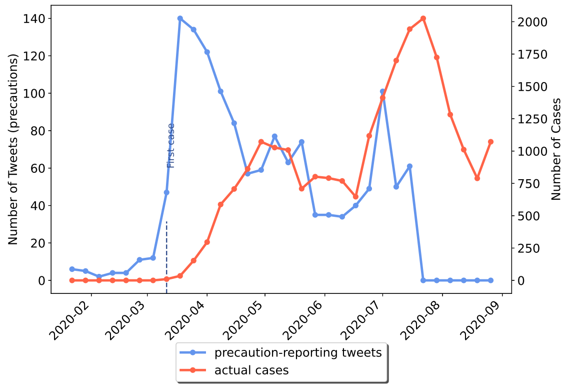

(c) Precaution-related tweets vs. cases

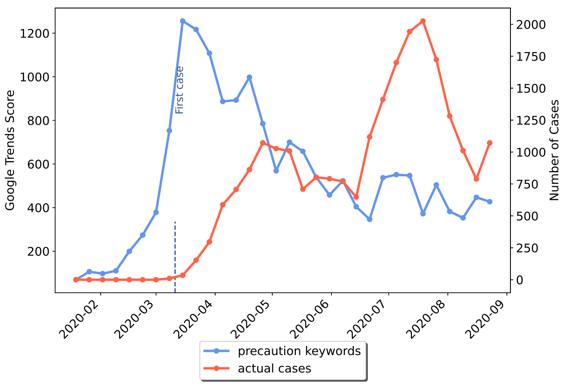

(d) Precaution-related searches vs. cases

Figure A37: Weekly comparison of online activities and actual number of cases in New Mexico

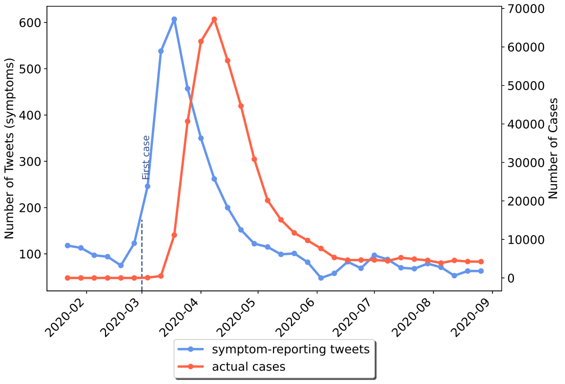

(a) Symptom-related tweets vs. cases

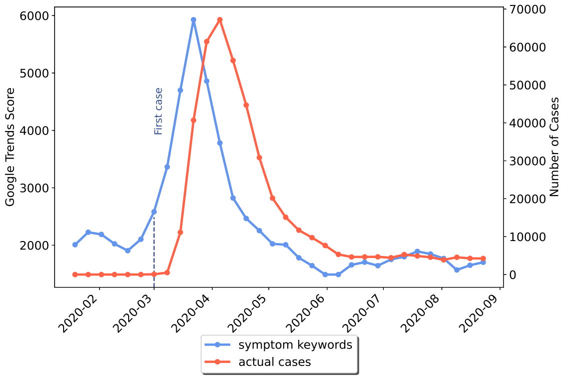

(b) Symptom-related searches vs. cases

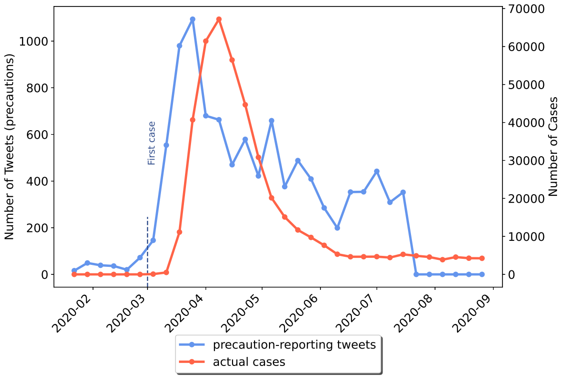

(c) Precaution-related tweets vs. cases

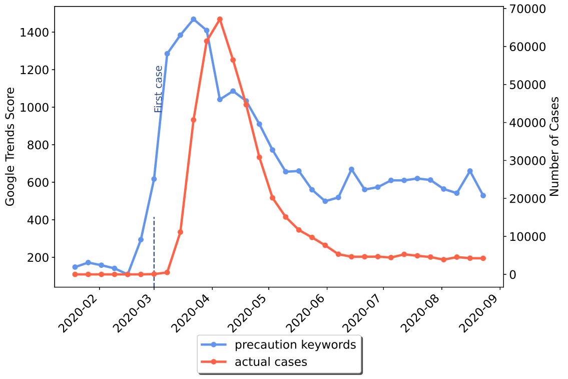

(d) Precaution-related searches vs. cases

Figure A38: Weekly comparison of online activities and actual number of cases in New York

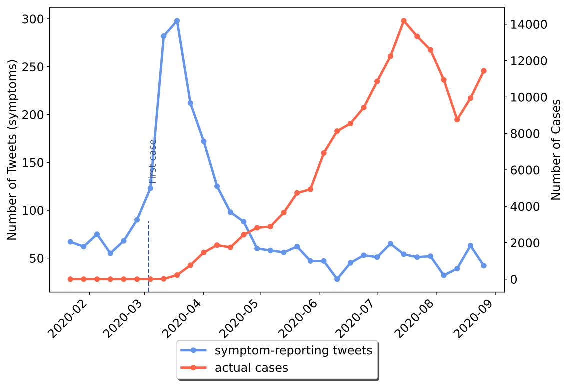

(a) Symptom-related tweets vs. cases

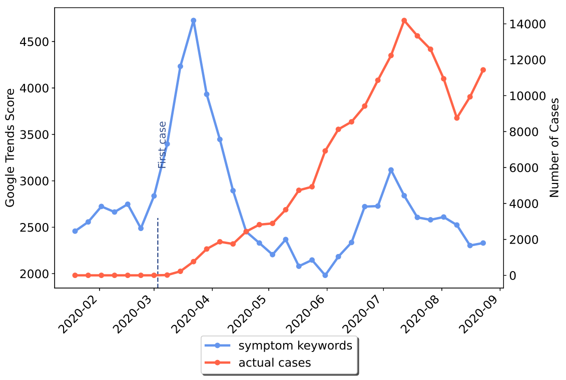

(b) Symptom-related searches vs. cases

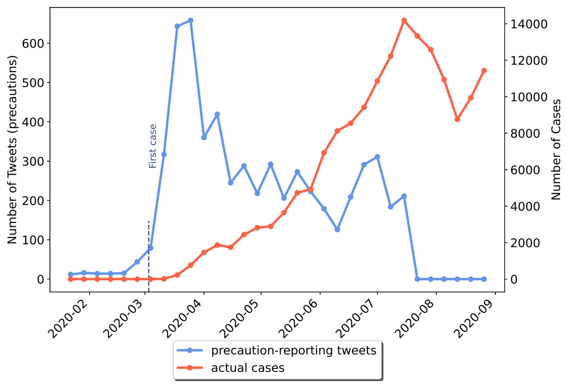

(c) Precaution-related tweets vs. cases

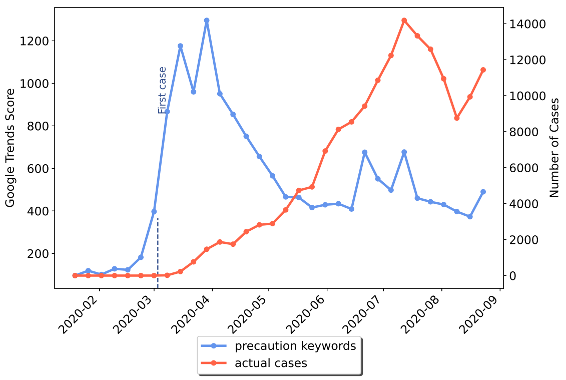

(d) Precaution-related searches vs. cases

Figure A39: Weekly comparison of online activities and actual number of cases in North Carolina

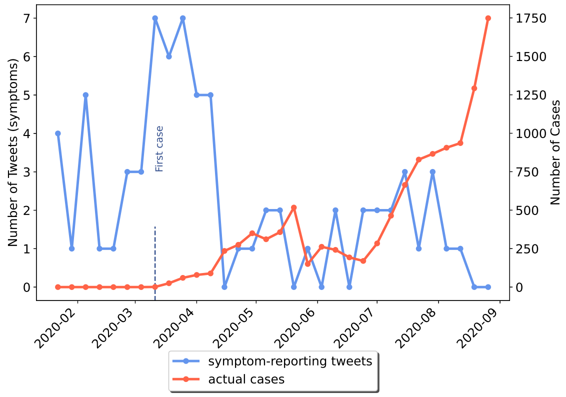

(a) Symptom-related tweets vs. cases

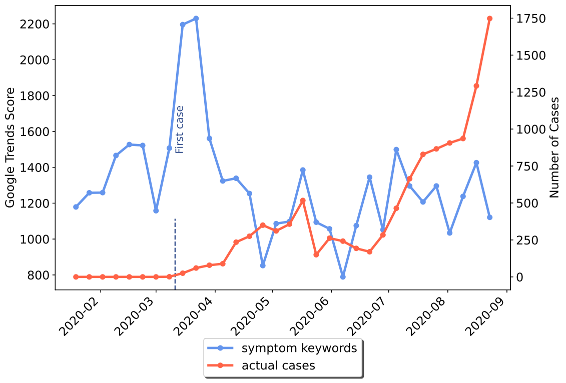

(b) Symptom-related searches vs. cases

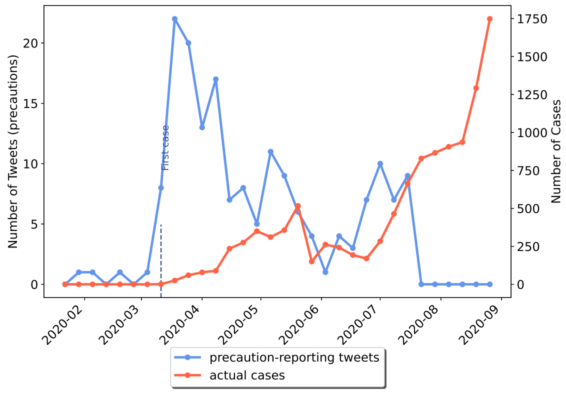

(c) Precaution-related tweets vs. cases

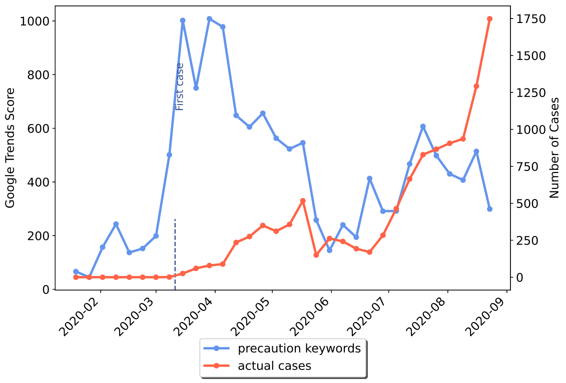

(d) Precaution-related searches vs. cases

Figure A40: Weekly comparison of online activities and actual number of cases in North Dakota

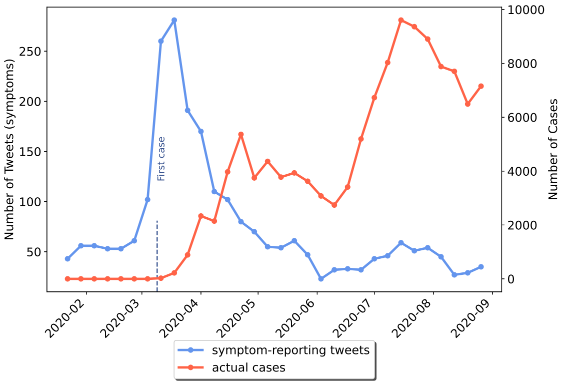

(a) Symptom-related tweets vs. cases

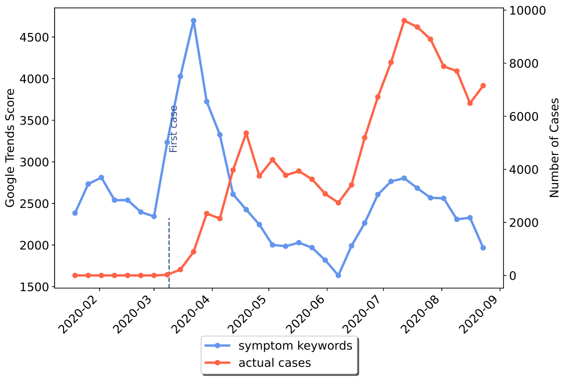

(b) Symptom-related searches vs. cases

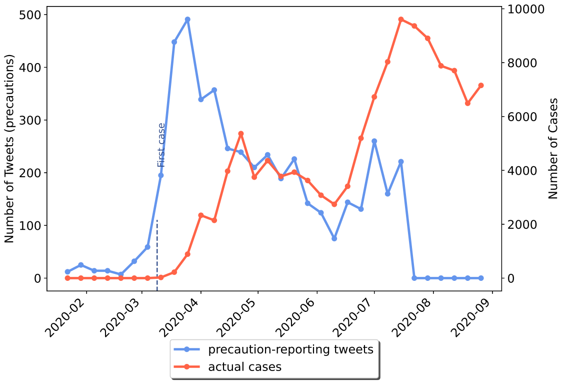

(c) Precaution-related tweets vs. cases

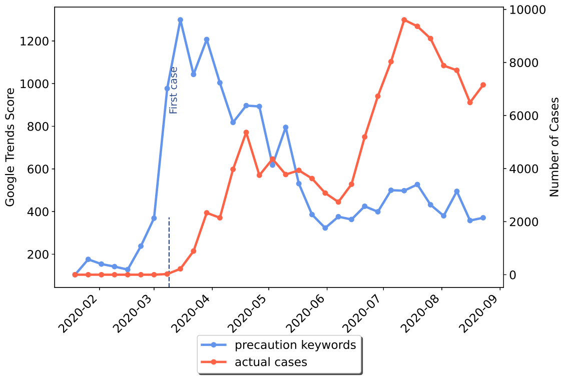

(d) Precaution-related searches vs. cases

Figure A41: Weekly comparison of online activities and actual number of cases in Ohio

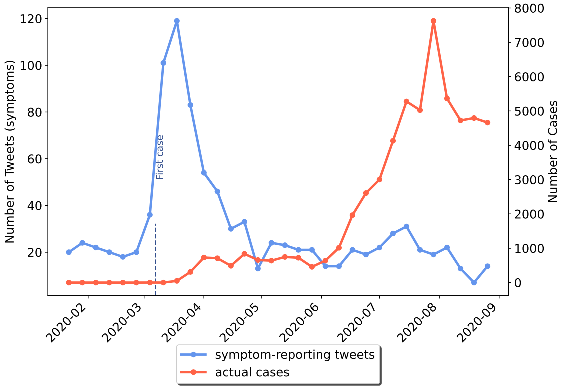

(a) Symptom-related tweets vs. cases

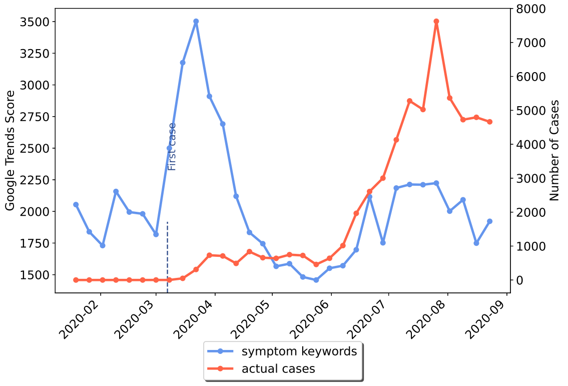

(b) Symptom-related searches vs. cases

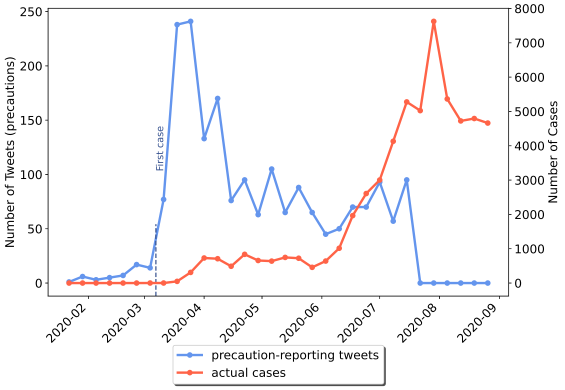

(c) Precaution-related tweets vs. cases

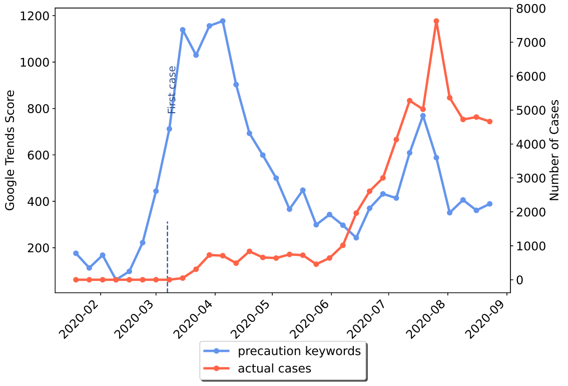

(d) Precaution-related searches vs. cases

Figure A42: Weekly comparison of online activities and actual number of cases in Oklahoma

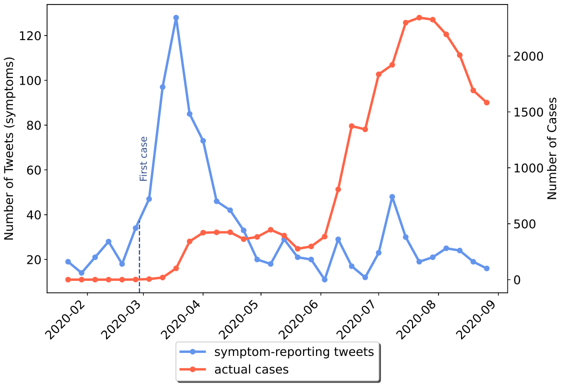

(a) Symptom-related tweets vs. cases

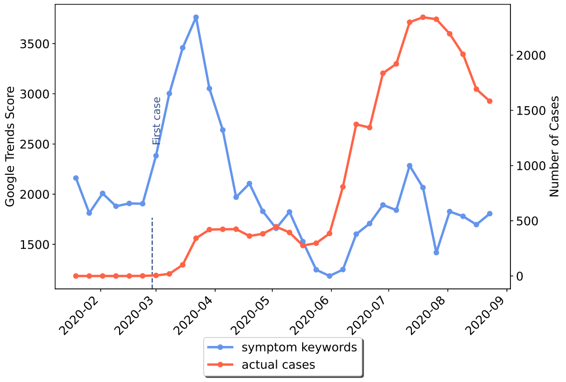

(b) Symptom-related searches vs. cases

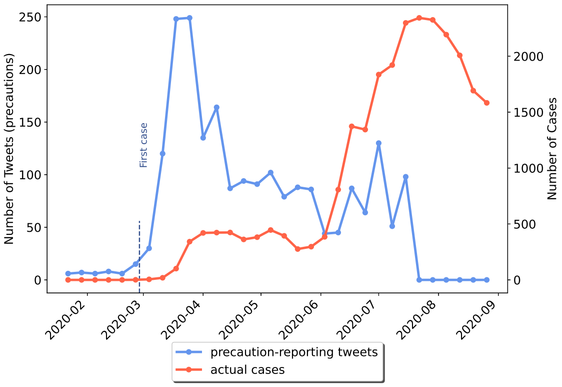

(c) Precaution-related tweets vs. cases

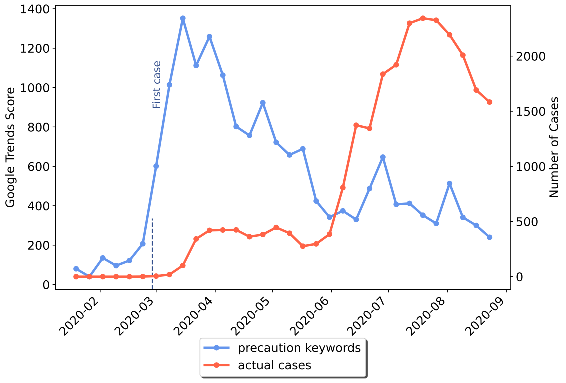

(d) Precaution-related searches vs. cases

Figure A43: Weekly comparison of online activities and actual number of cases in Oregon

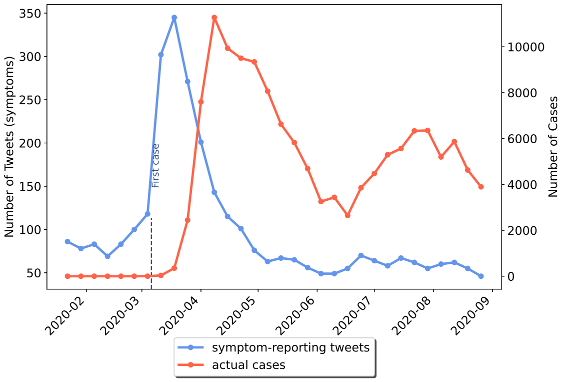

(a) Symptom-related tweets vs. cases

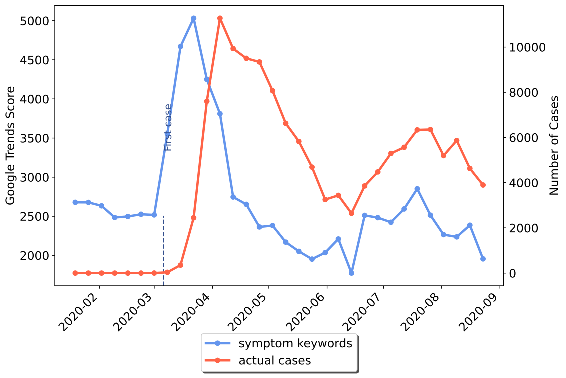

(b) Symptom-related searches vs. cases

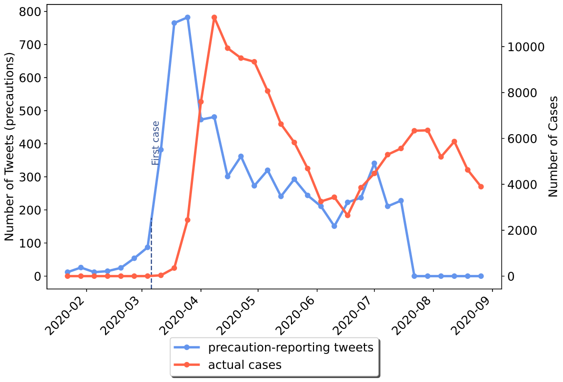

(c) Precaution-related tweets vs. cases

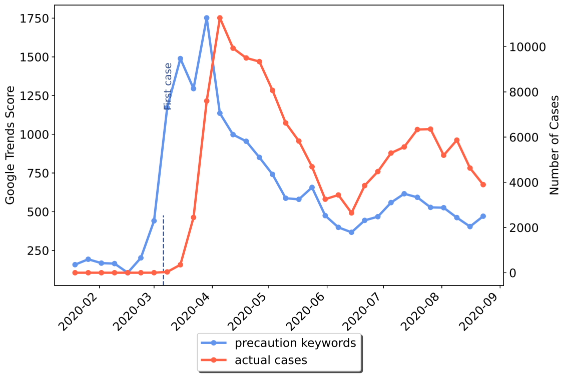

(d) Precaution-related searches vs. cases

Figure A44: Weekly comparison of online activities and actual number of cases in Pennsylvania

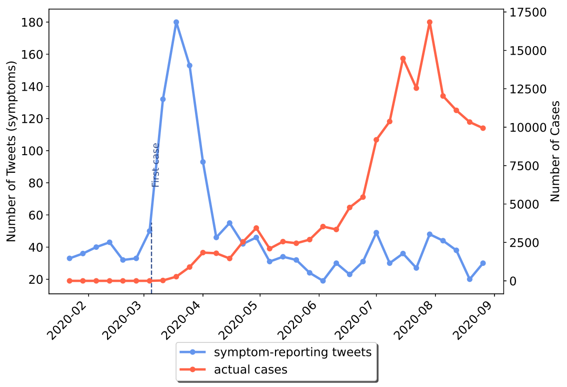

(a) Symptom-related tweets vs. cases

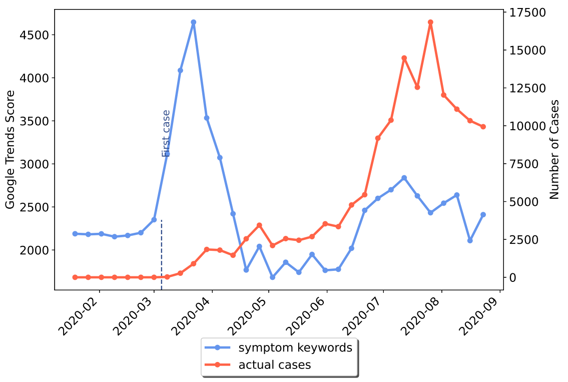

(b) Symptom-related searches vs. cases

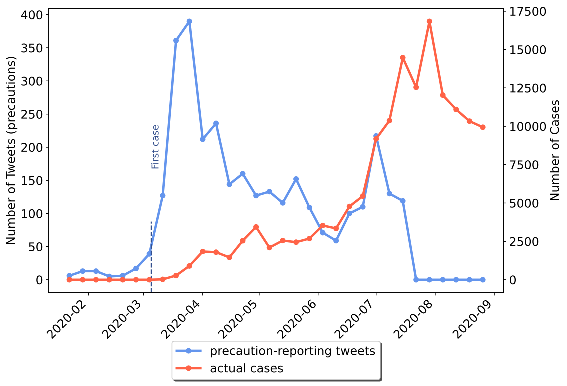

(c) Precaution-related tweets vs. cases

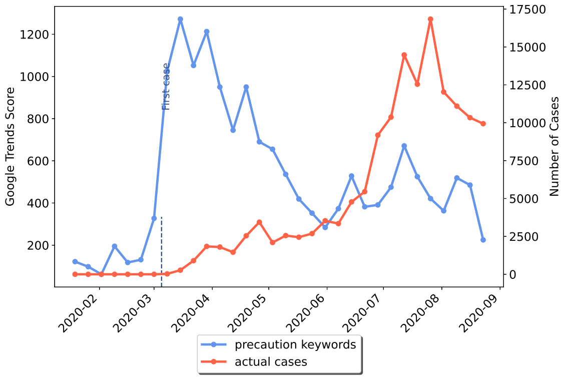

(d) Precaution-related searches vs. cases

Figure A45: Weekly comparison of online activities and actual number of cases in Tennessee

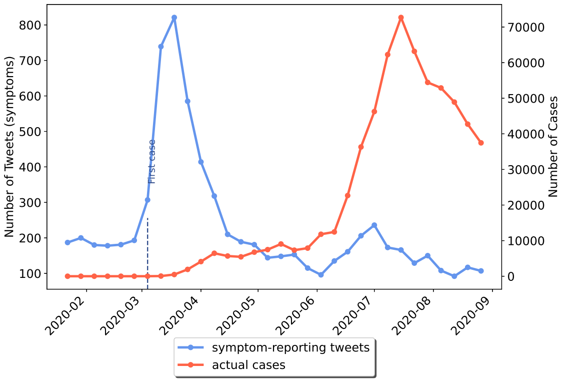

(a) Symptom-related tweets vs. cases

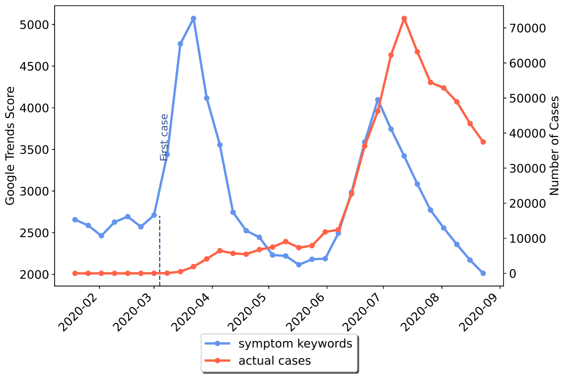

(b) Symptom-related searches vs. cases

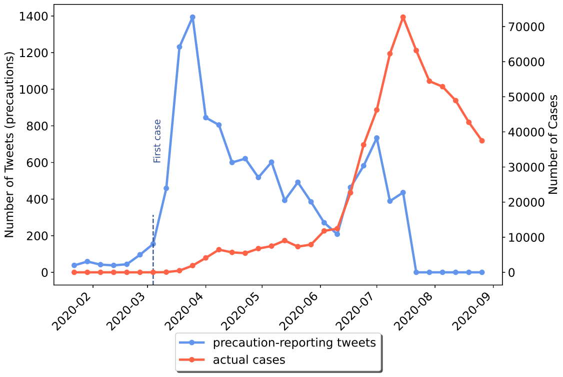

(c) Precaution-related tweets vs. cases

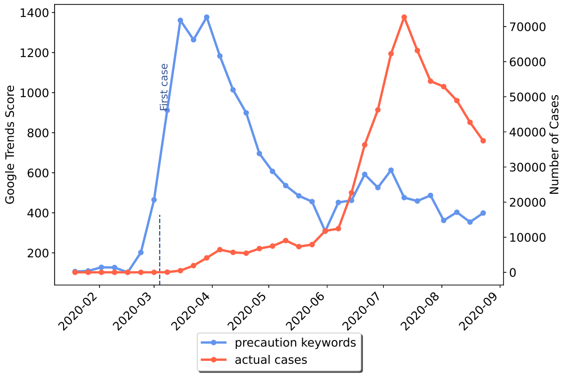

(d) Precaution-related searches vs. cases

Figure A46: Weekly comparison of online activities and actual number of cases in Texas

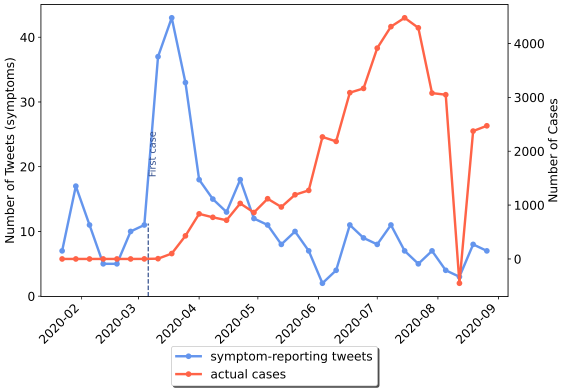

(a) Symptom-related tweets vs. cases

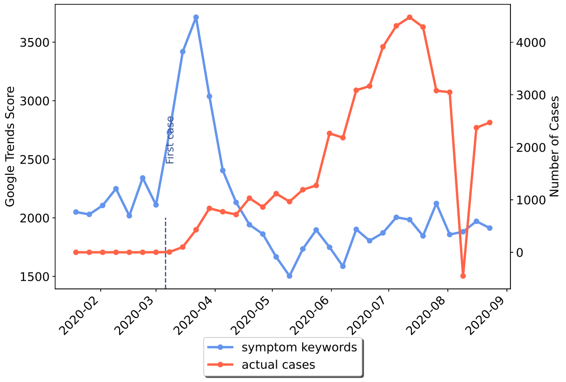

(b) Symptom-related searches vs. cases

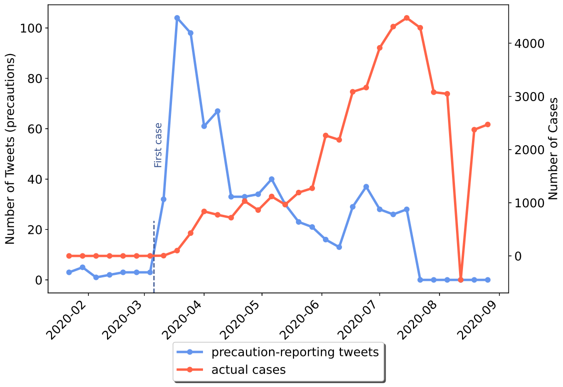

(c) Precaution-related tweets vs. cases

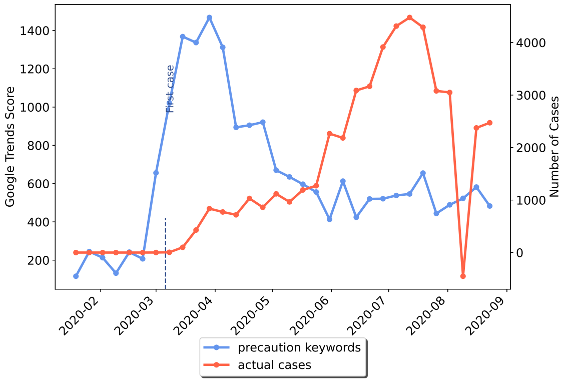

(d) Precaution-related searches vs. cases

Figure A47: Weekly comparison of online activities and actual number of cases in Utah

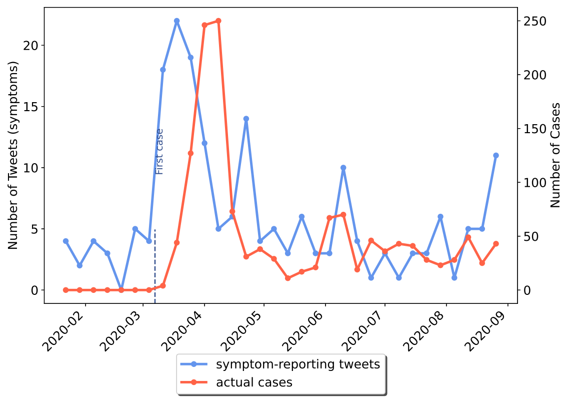

(a) Symptom-related tweets vs. cases

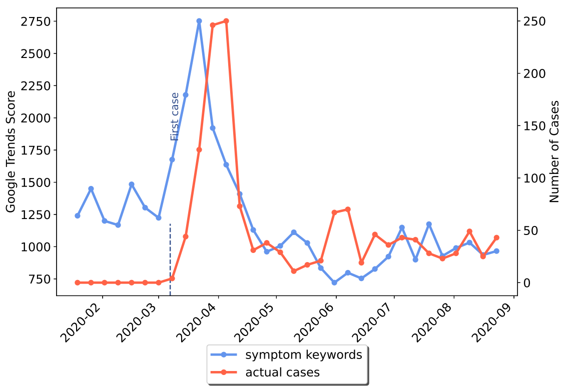

(b) Symptom-related searches vs. cases

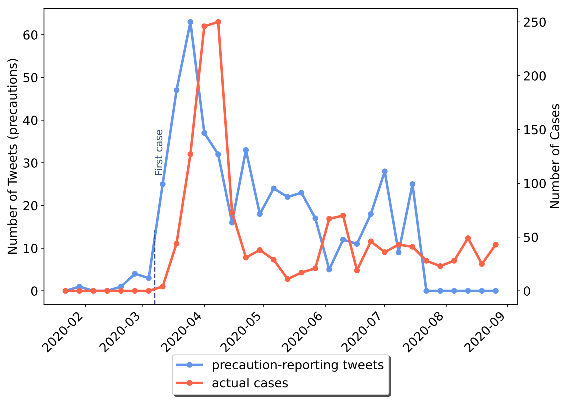

(c) Precaution-related tweets vs. cases

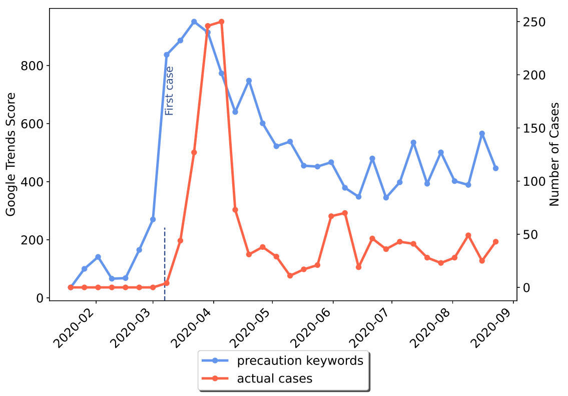

(d) Precaution-related searches vs. cases

Figure A48: Weekly comparison of online activities and actual number of cases in Vermont

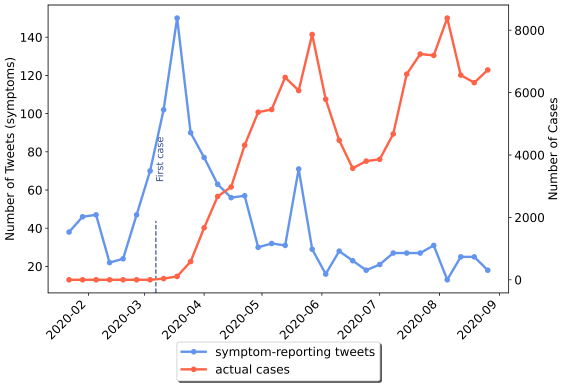

(a) Symptom-related tweets vs. cases

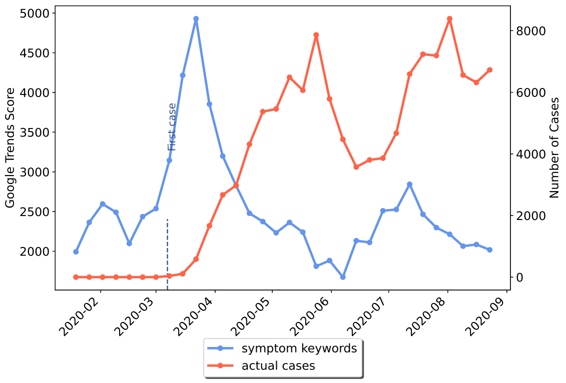

(b) Symptom-related searches vs. cases

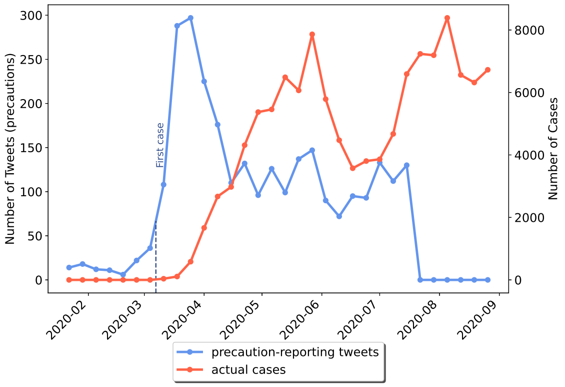

(c) Precaution-related tweets vs. cases

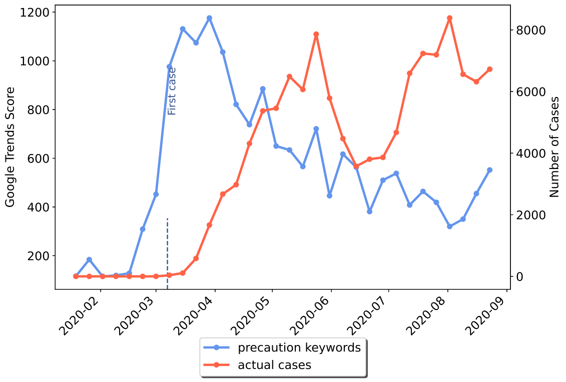

(d) Precaution-related searches vs. cases

Figure A49: Weekly comparison of online activities and actual number of cases in Virginia

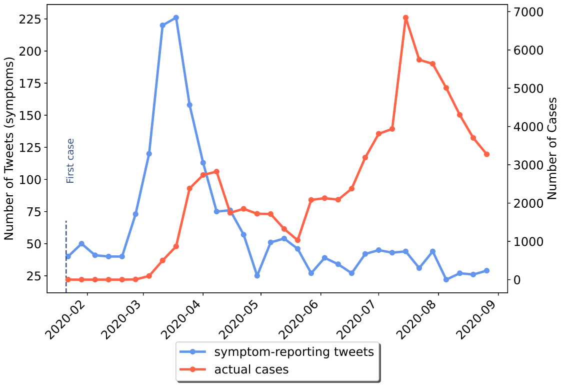

(a) Symptom-related tweets vs. cases

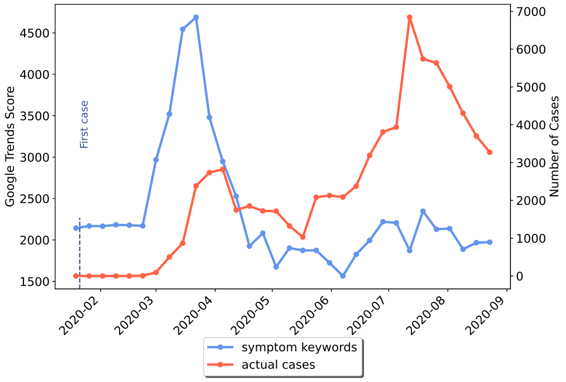

(b) Symptom-related searches vs. cases

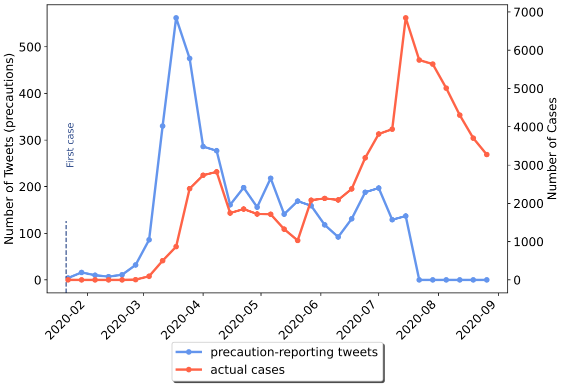

(c) Precaution-related tweets vs. cases

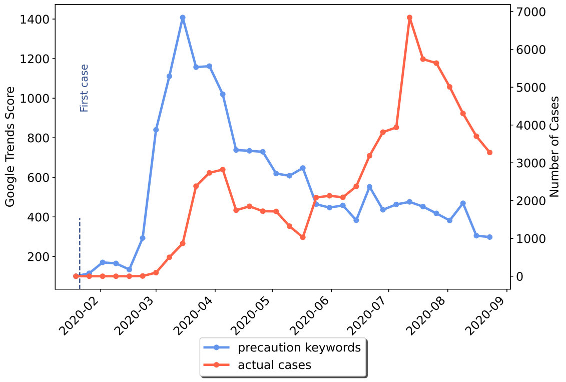

(d) Precaution-related searches vs. cases

Figure A50: Weekly comparison of online activities and actual number of cases in Washington

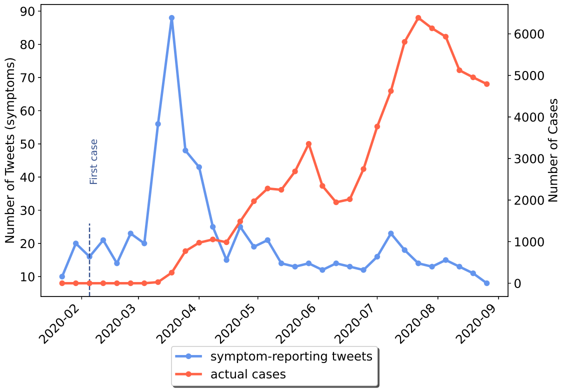

(a) Symptom-related tweets vs. cases

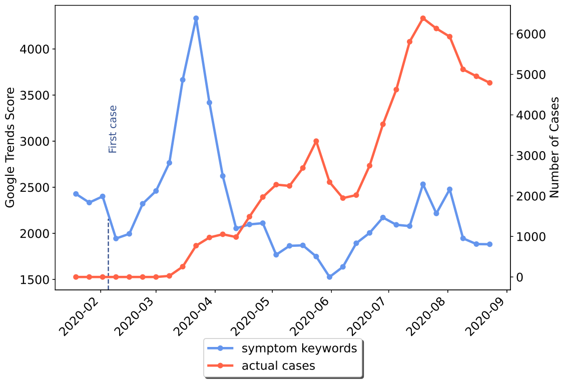

(b) Symptom-related searches vs. cases

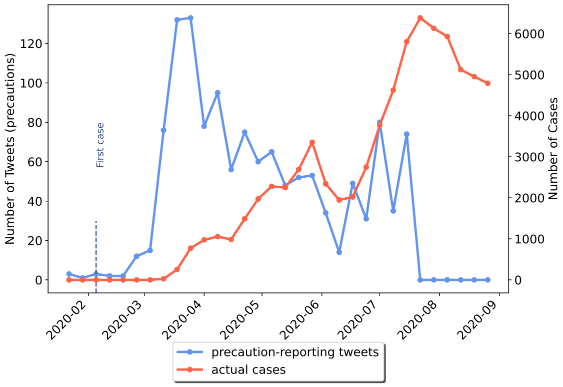

(c) Precaution-related tweets vs. cases

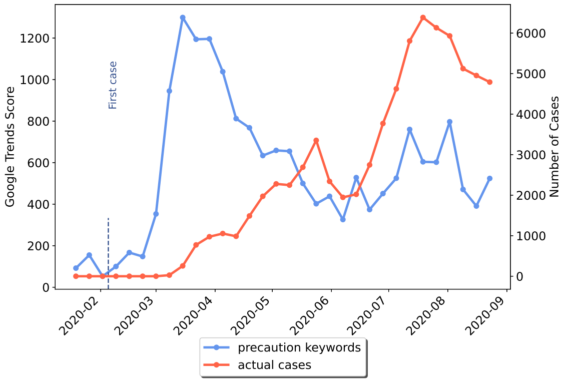

(d) Precaution-related searches vs. cases

Figure A51: Weekly comparison of online activities and actual number of cases in Wisconsin

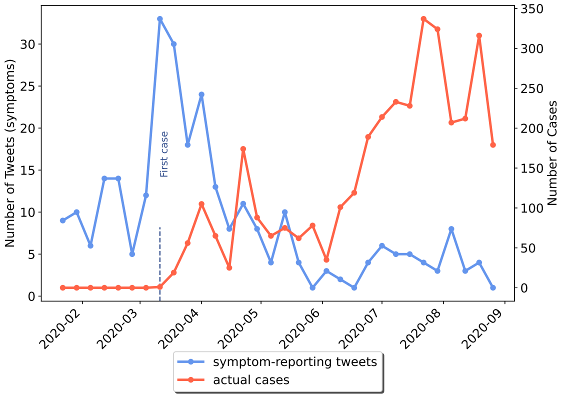

(a) Symptom-related tweets vs. cases

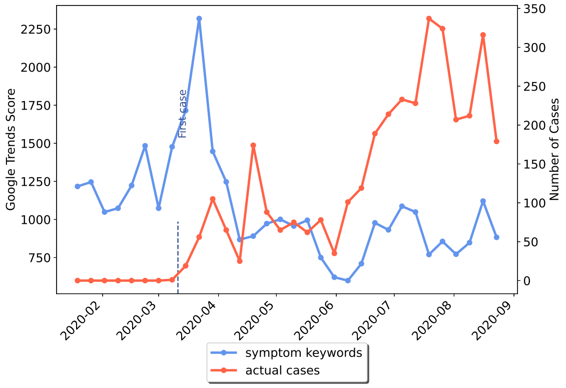

(b) Symptom-related searches vs. cases

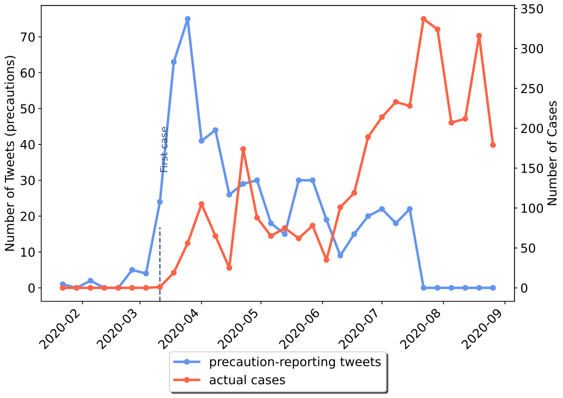

(c) Precaution-related tweets vs. cases

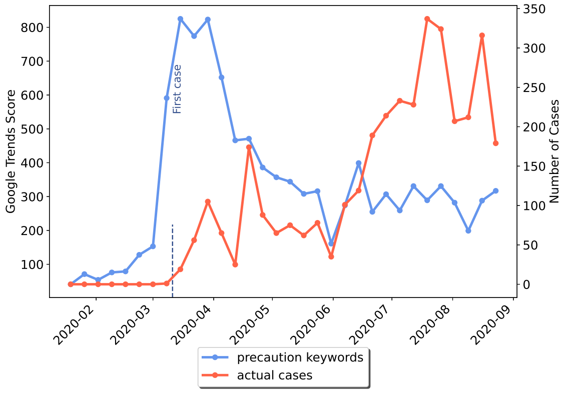

(d) Precaution-related searches vs. cases

Figure A52: Weekly comparison of online activities and actual number of cases in Wyoming

B Data Description

Table B1: Provincial number of tweets (CA)

Location	precautions	symptoms
Alberta	6590	738
British Columbia	8152	739
Manitoba	1837	145
New Brunswick	407	25
Newfoundland and Labrador	708	66
Northwest Territories	51	6
Nova Scotia	1969	214
Ontario	29299	2537
Prince Edward Island	120	17
Quebec	3709	315
Saskatchewan	915	123
Yukon	63	6

Table B2: Major provincial number of tweets (US)

Location	precautions	symptoms
Alabama	1040	681
Alaska	244	158
Arizona	2703	1606
Arkansas	687	425
California	15193	10777
Colorado	1975	1026
Connecticut	921	498
Delaware	127	64
Florida	6021	4040
Georgia	3264	2263
Hawaii	398	200
Idaho	333	183
Illinois	3794	2155
Indiana	1296	838
Iowa	802	483
Kansas	754	464
Kentucky	1157	529
Louisiana	1491	1246
Maine	434	223
Maryland	2016	1153
Massachusetts	2668	1306
Michigan	1679	1082
Minnesota	1288	672
Mississippi	587	389
Missouri	1409	789
Montana	206	141
Nebraska	866	409
Nevada	711	421
New Hampshire	342	137
New Jersey	1949	1107
New Mexico	1413	808
New York	9355	5144
North Carolina	3643	2038
North Dakota	175	84
Ohio	3385	2032
Oklahoma	1959	1118
Oregon	1894	1137
Pennsylvania	4336	2472
Rhode Island	546	279
South Carolina	1284	784
South Dakota	306	180
Tennessee	3007	1718
Texas	11950	8838
Utah	776	448
Vermont	477	222
Virginia	2808	1661
Washington	4308	2301
West Virginia	630	347
Wisconsin	1285	796
Wyoming	563	332

Table B3: Correlation coefficients (r) of weekly online activities and COVID-19 cases

Location	TW Symptoms	TW Precautions	GT Symptoms	GT Precautions
Canada	0.85 (lag=5)	0.93 (lag=3)	0.75 (lag=5)	0.84 (lag=3)
Alberta	0.67 (lag=5)	0.77 (lag=3)	-	0.53 (lag=4)
Quebec	0.85 (lag=5)	0.89 (lag=3)	0.7 (lag=3)	0.46 (lag=3)
Ontario	0.77 (lag=5)	0.95 (lag=3)	-	0.59 (lag=3)
Newfoundland	0.62 (lag=1)	0.88 (lag=1)	0.54 (lag=2)	0.67 (lag=1)
Nova Scotia	0.94 (lag=4)	0.84 (lag=2)	0.5 (lag=5)	0.6 (lag=3)
Colorado	-	0.68 (lag=4)	-	0.64 (lag=4)
Connecticut	0.92 (lag=4)	0.89 (lag=3)	0.66 (lag=5)	0.9 (lag=4)
Delaware	0.62 (lag=5)	0.85 (lag=4)	0.38 (lag=6)	0.83 (lag=5)
Illinois	0.65 (lag=6)	0.8 (lag=5)	-	0.68 (lag=5)
Indiana	-	0.41 (lag=3)	-	0.42 (lag=5)
Maine	0.41 (lag=6)	0.62 (lag=3)	-	0.57 (lag=4)
Maryland	0.44 (lag=5)	0.73 (lag=4)	-	0.6 (lag=4)
Massachusetts	0.94 (lag=5)	0.9 (lag=3)	0.66 (lag=5)	0.86 (lag=4)
Michigan	0.7 (lag=3)	0.81 (lag=2)	0.38 (lag=4)	0.87 (lag=3)
New Hampshire	0.74 (lag=5)	0.85 (lag=4)	0.46 (lag=6)	0.8 (lag=5)
New Jersey	0.95 (lag=4)	0.87 (lag=2)	0.7 (lag=5)	0.9 (lag=3)
New York	0.97 (lag=3)	0.86 (lag=2)	0.72 (lag=4)	0.91 (lag=3)
Pennsylvania	0.68 (lag=4)	0.88 (lag=3)	0.36 (lag=5)	0.89 (lag=4)
Rhode Island	0.87 (lag=5)	0.9 (lag=4)	0.56 (lag=6)	0.84 (lag=4)
Vermont	0.9 (lag=2)	0.83 (lag=1)	0.63 (lag=3)	0.88 (lag=2)
