# Supplementary figures and images for: Prediction of COVID-19 Waves Using Social Media and Google Search: A Case Study of the US and Canada (part 2 of 2)
Source: Front Public Health. 2021 Apr 16;9:656635. doi: 10.3389/fpubh.2021.656635 (PMC8085269; doi:10.3389/fpubh.2021.656635)

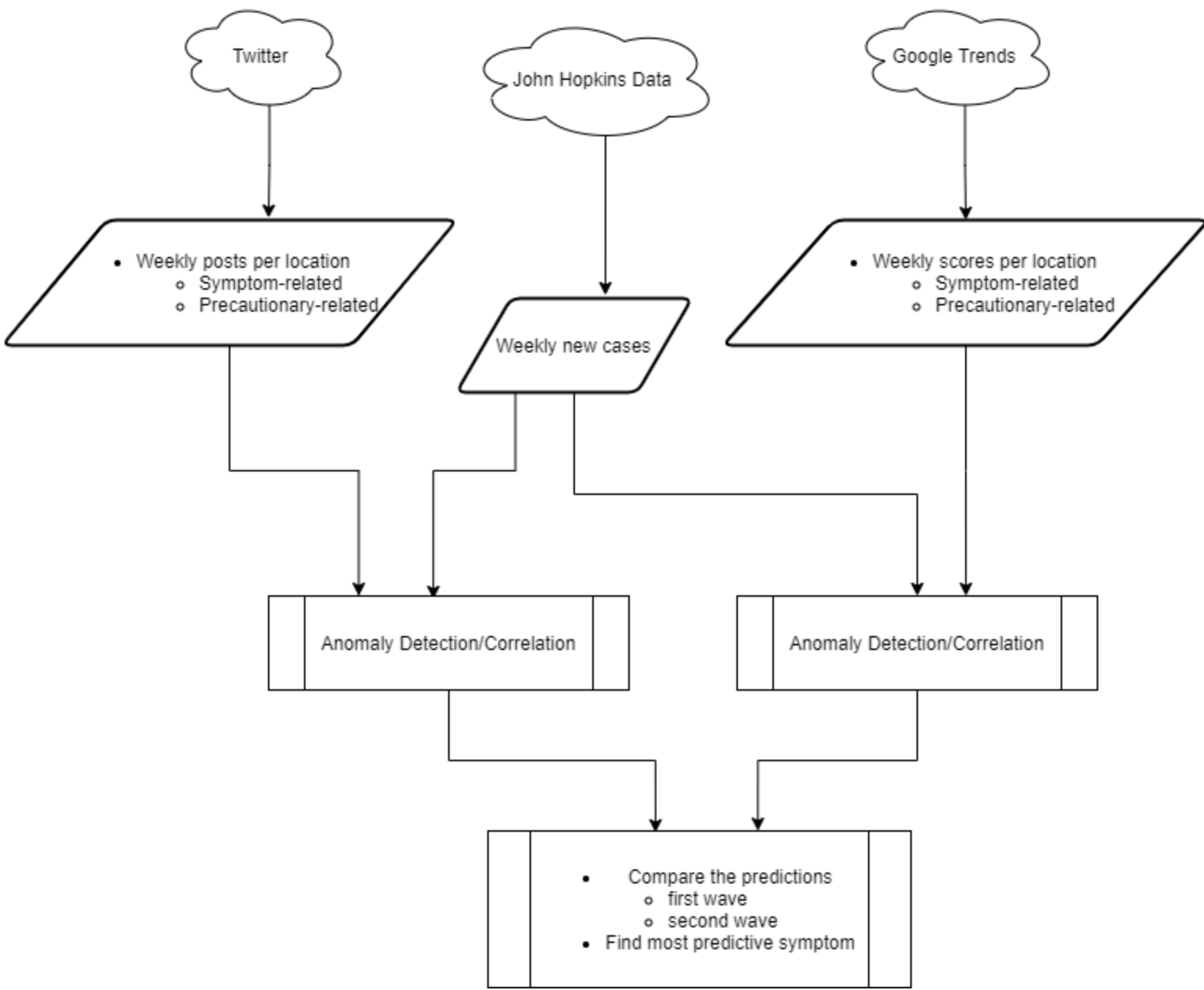

Supplement: Supplementary file 2 [file Data_Sheet_1.ZIP › figures/methodology_flow-eps-converted-to.pdf]

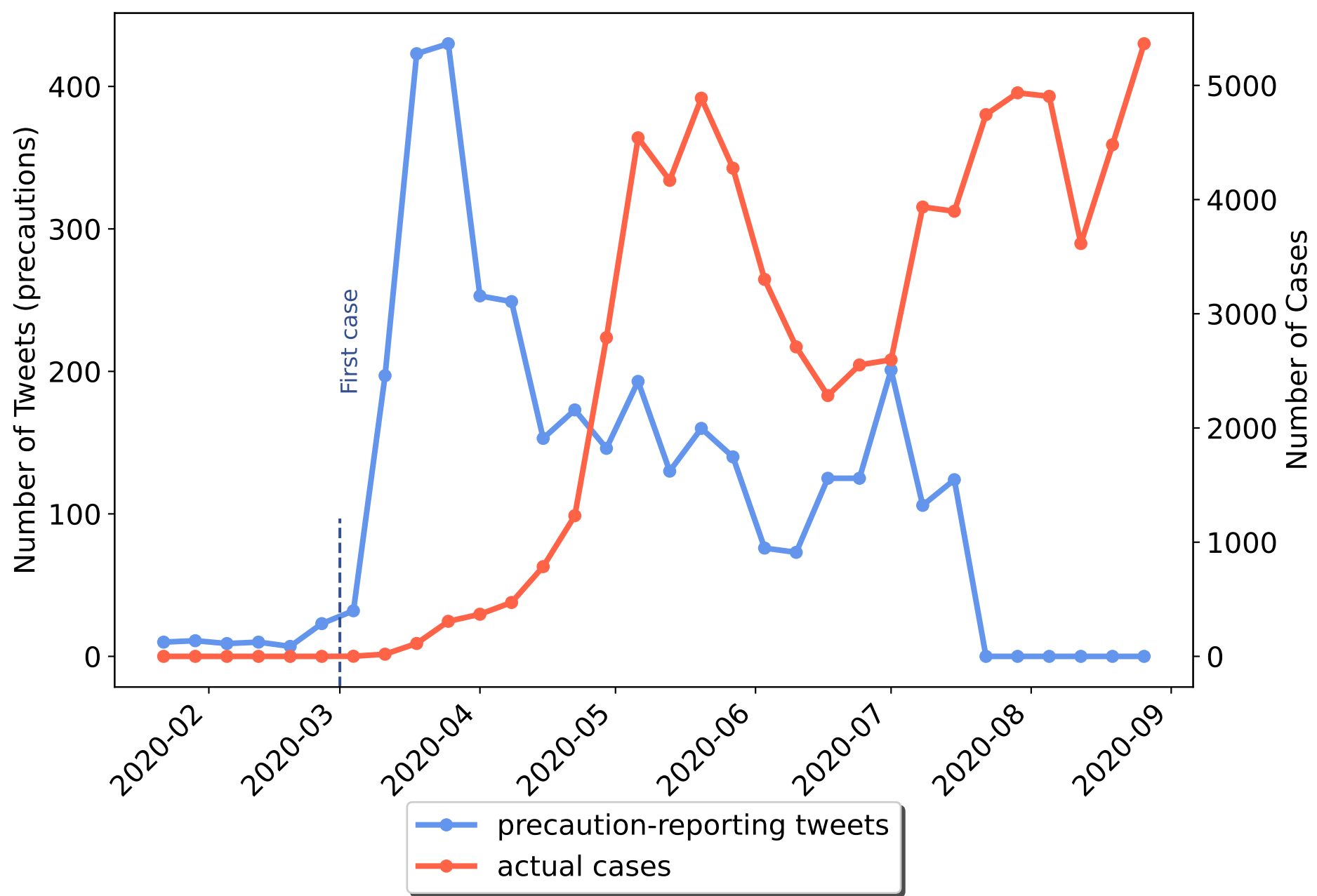

Supplement: Supplementary file 2 [file Data_Sheet_1.ZIP › figures/Minnesota_precaution_twitter-eps-converted-to.pdf]

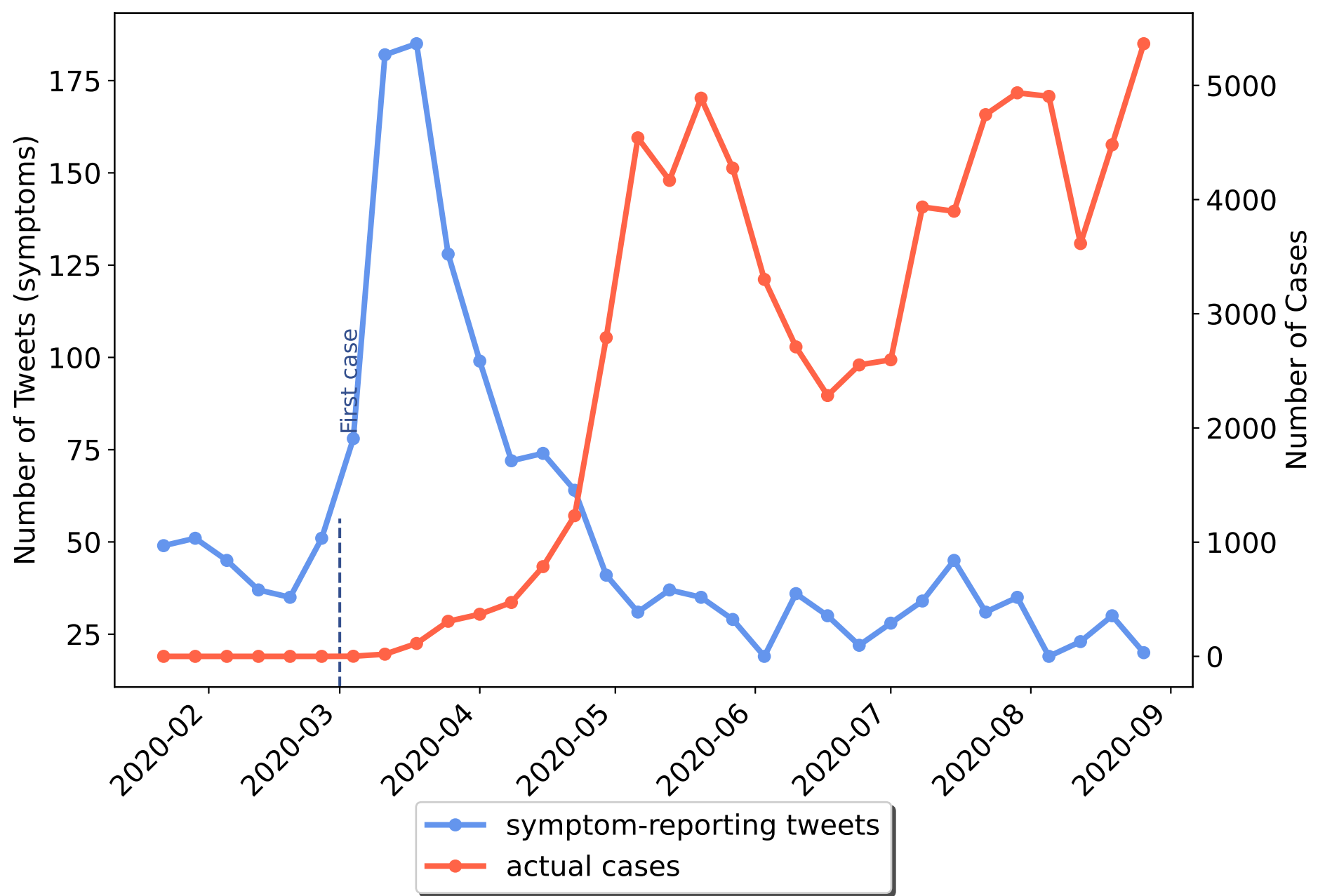

Supplement: Supplementary file 2 [file Data_Sheet_1.ZIP › figures/Minnesota_symptom_twitter-eps-converted-to.pdf]

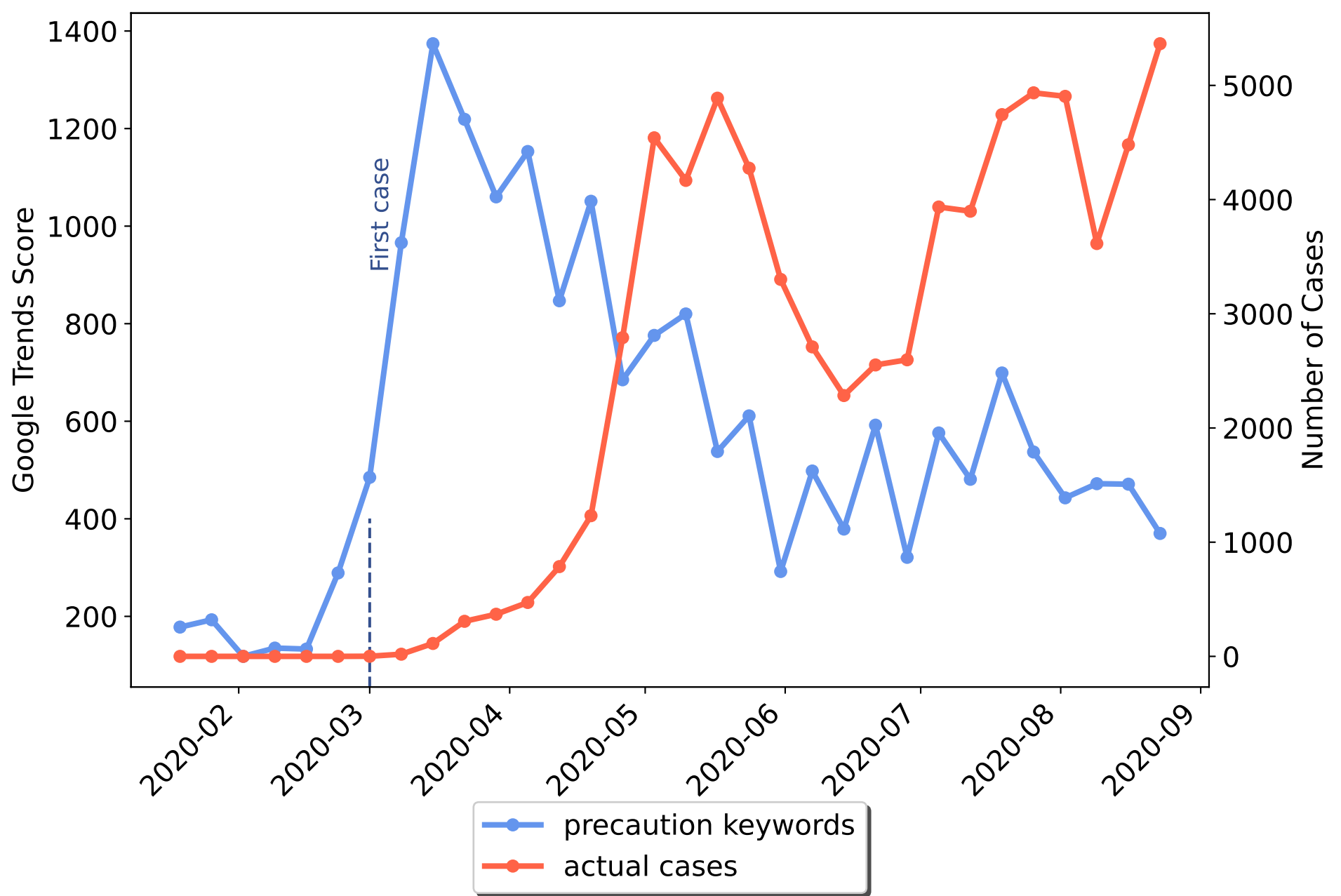

Supplement: Supplementary file 2 [file Data_Sheet_1.ZIP › figures/Minnesota_totalprecaution_GT-eps-converted-to.pdf]

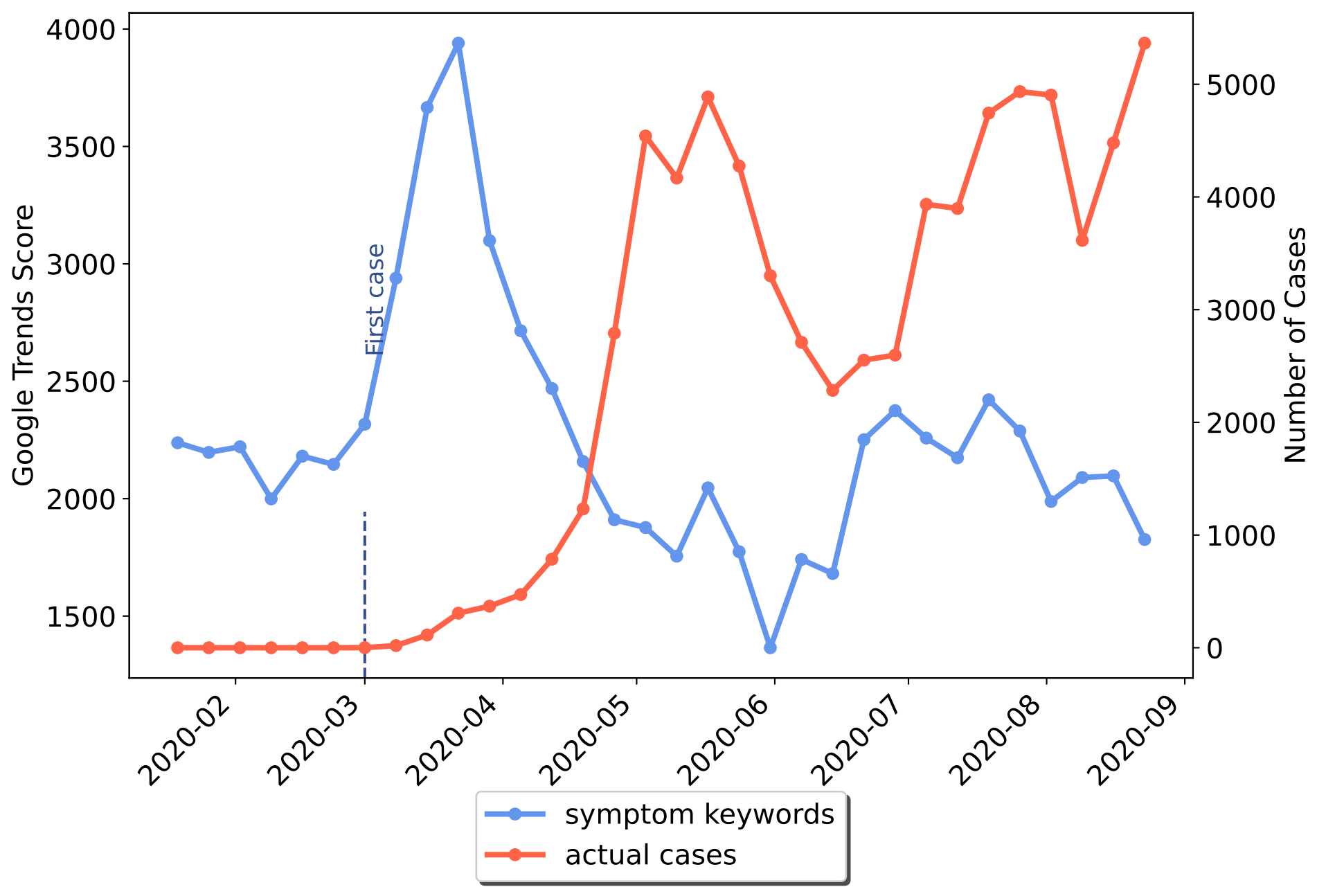

Supplement: Supplementary file 2 [file Data_Sheet_1.ZIP › figures/Minnesota_totalsymptom_GT-eps-converted-to.pdf]

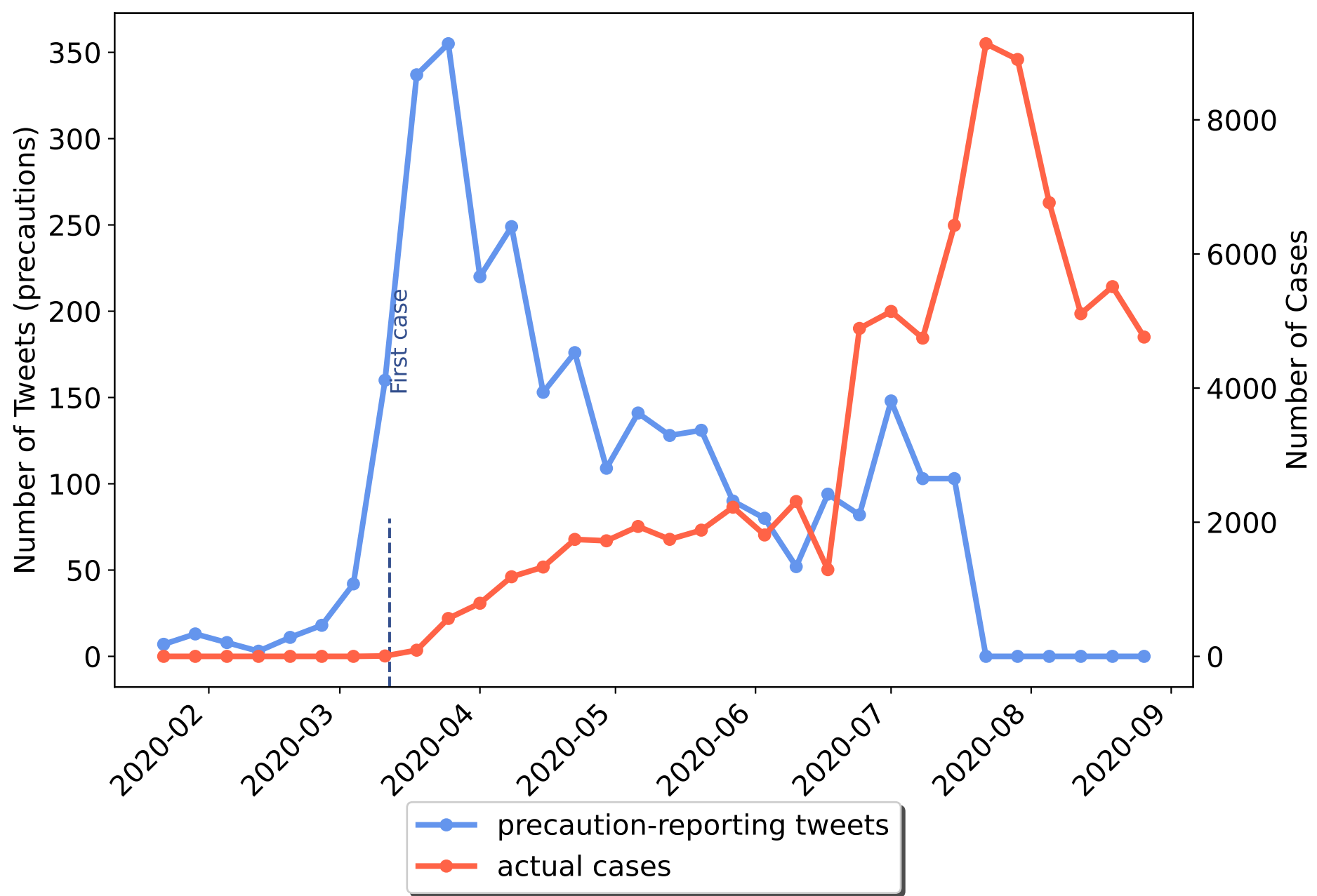

Supplement: Supplementary file 2 [file Data_Sheet_1.ZIP › figures/Mississippi_precaution_twitter-eps-converted-to.pdf]

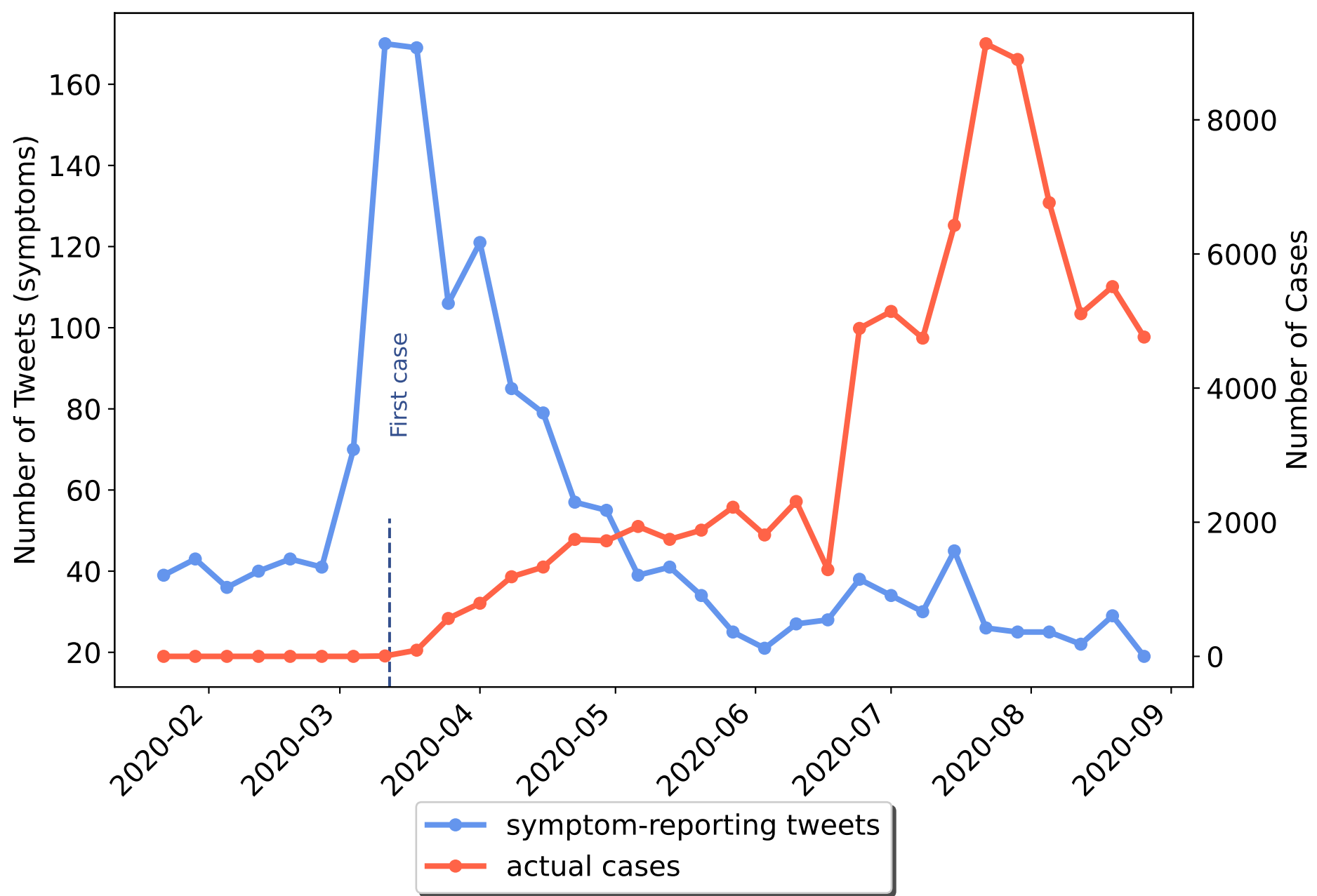

Supplement: Supplementary file 2 [file Data_Sheet_1.ZIP › figures/Mississippi_symptom_twitter-eps-converted-to.pdf]

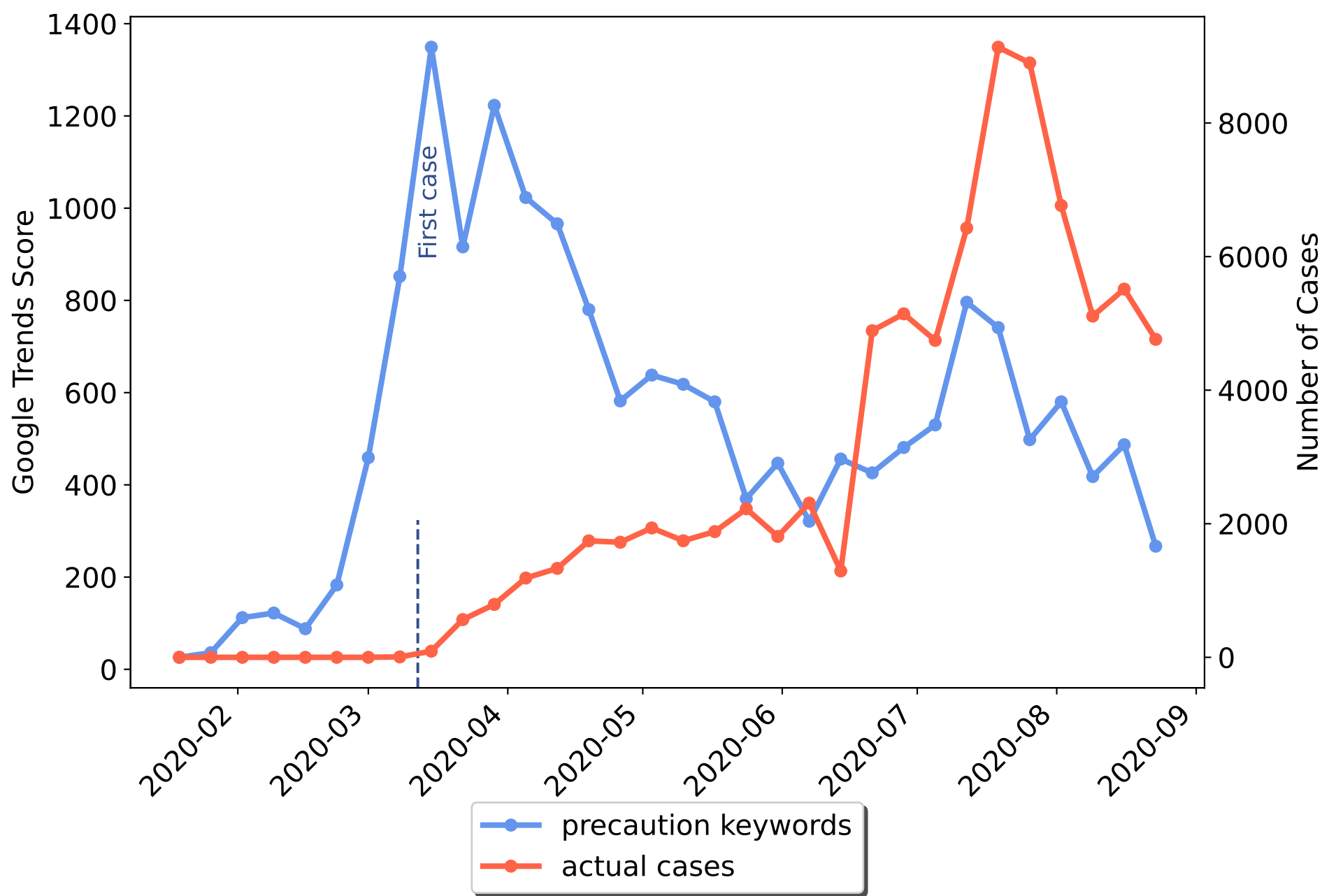

Supplement: Supplementary file 2 [file Data_Sheet_1.ZIP › figures/Mississippi_totalprecaution_GT-eps-converted-to.pdf]

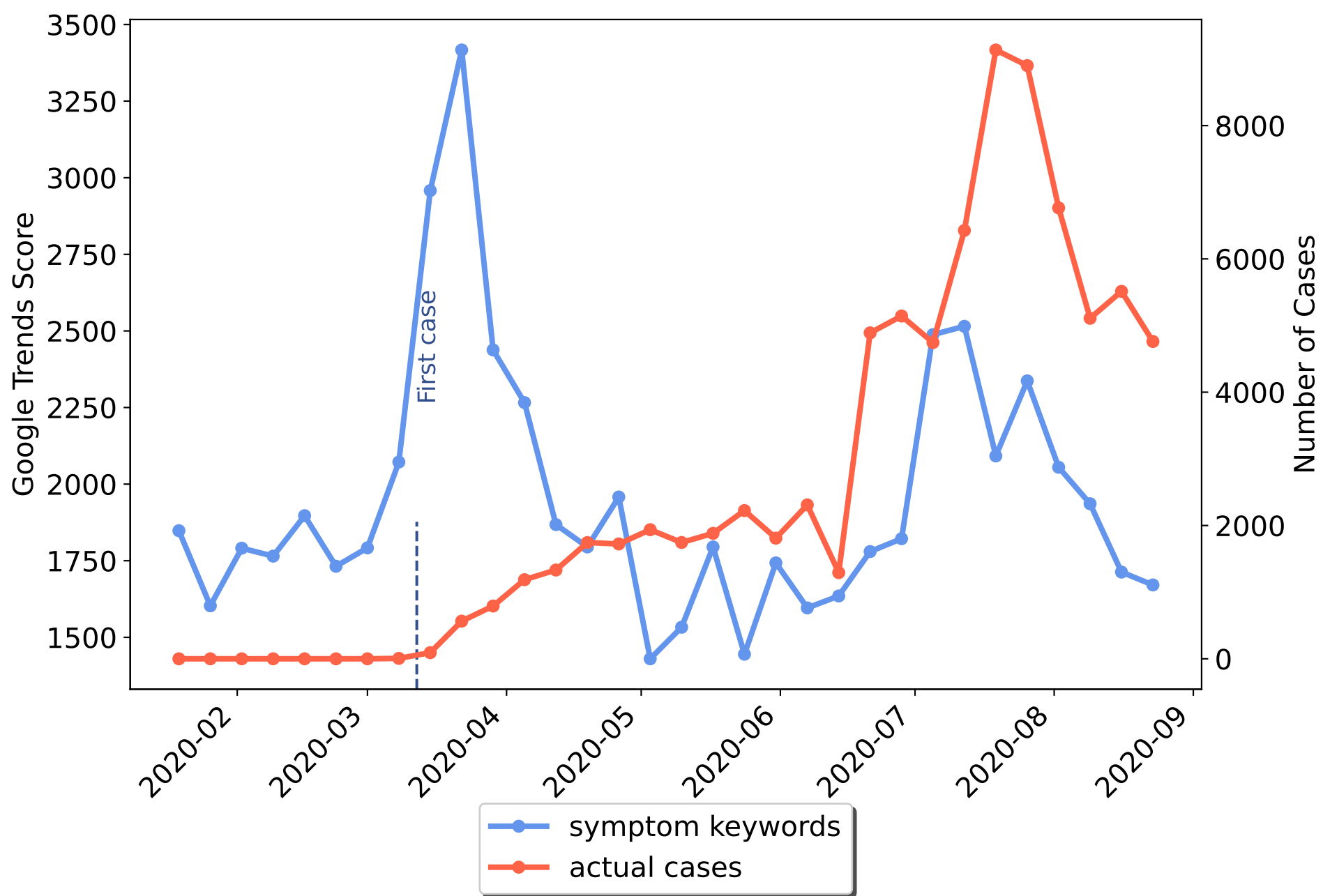

Supplement: Supplementary file 2 [file Data_Sheet_1.ZIP › figures/Mississippi_totalsymptom_GT-eps-converted-to.pdf]

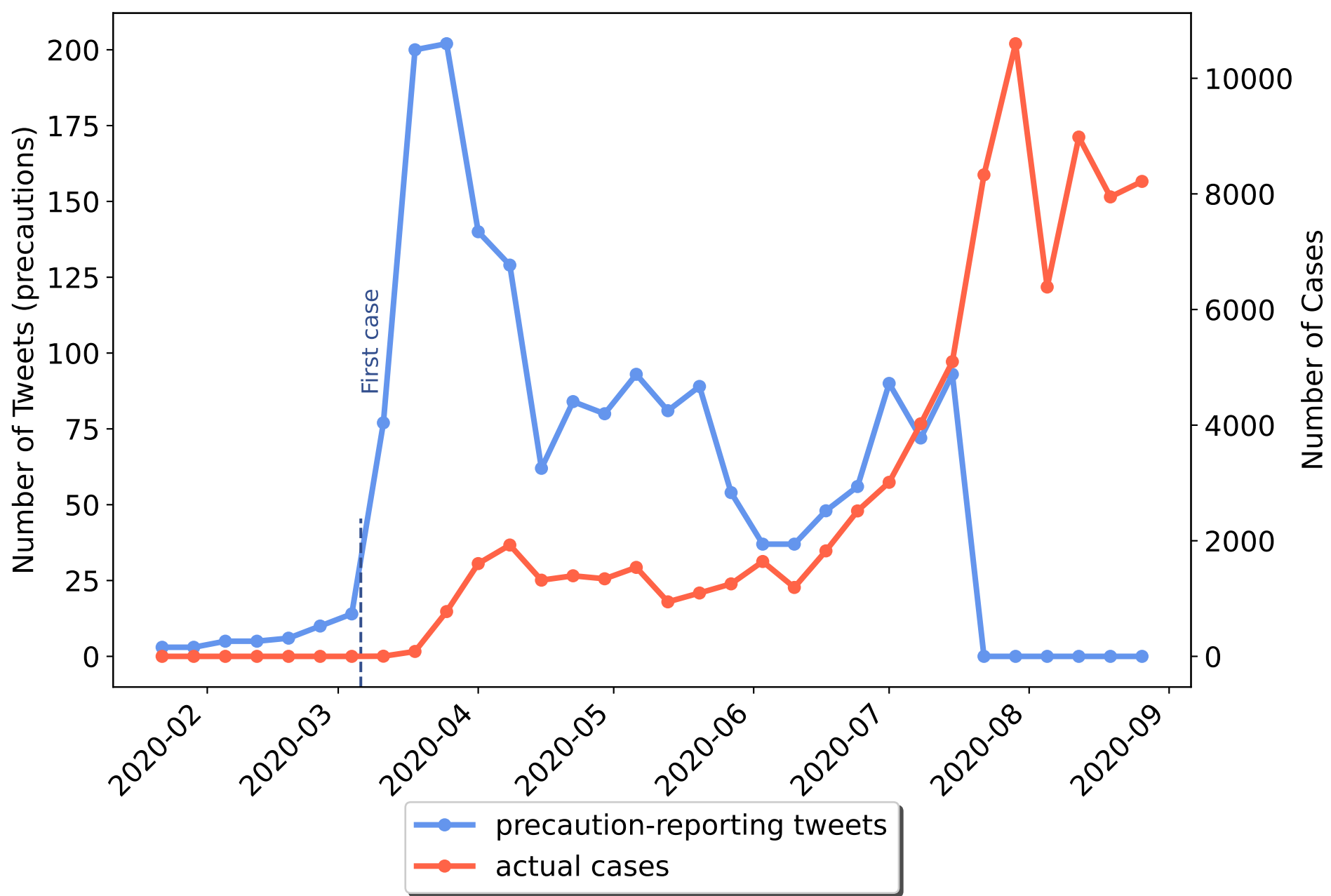

Supplement: Supplementary file 2 [file Data_Sheet_1.ZIP › figures/Missouri_precaution_twitter-eps-converted-to.pdf]

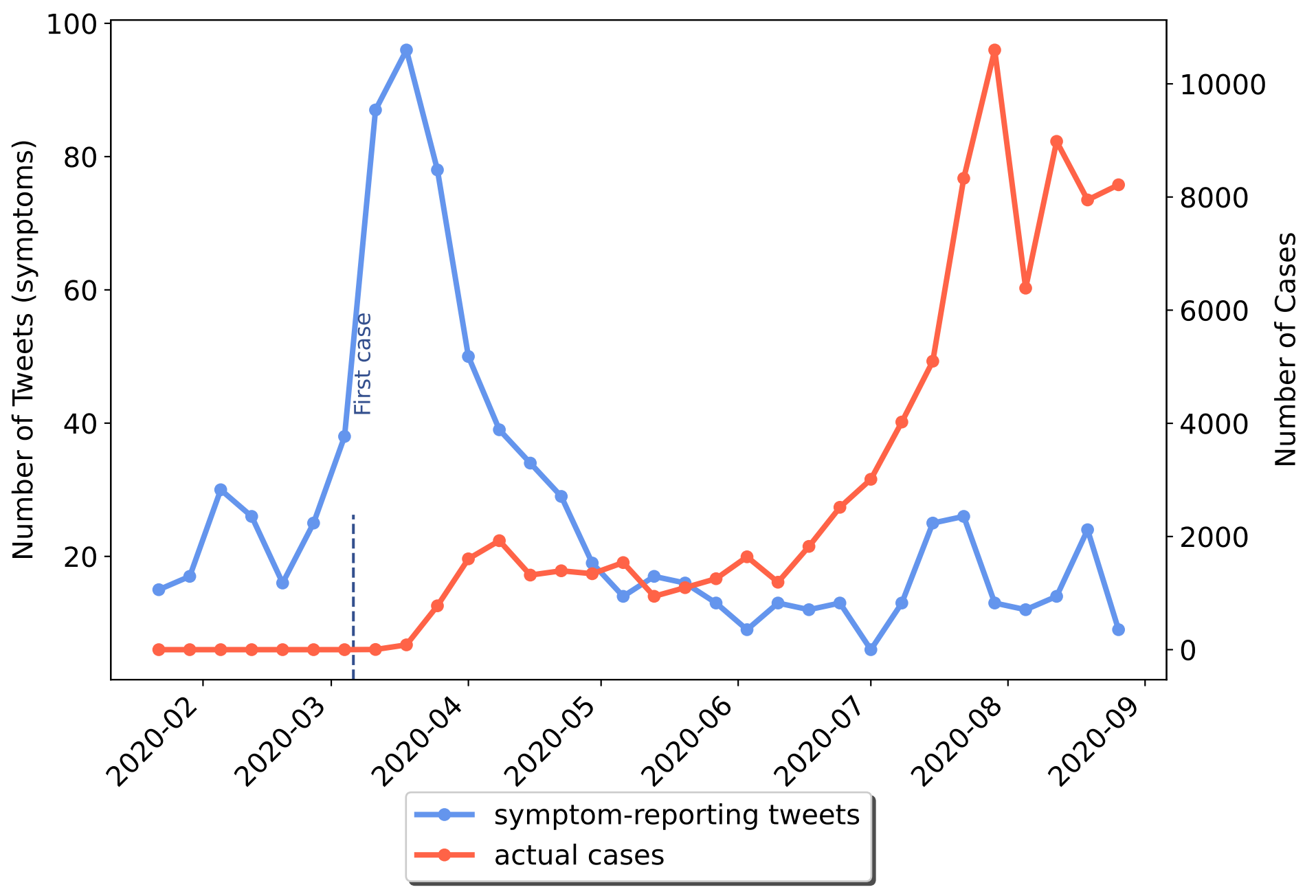

Supplement: Supplementary file 2 [file Data_Sheet_1.ZIP › figures/Missouri_symptom_twitter-eps-converted-to.pdf]

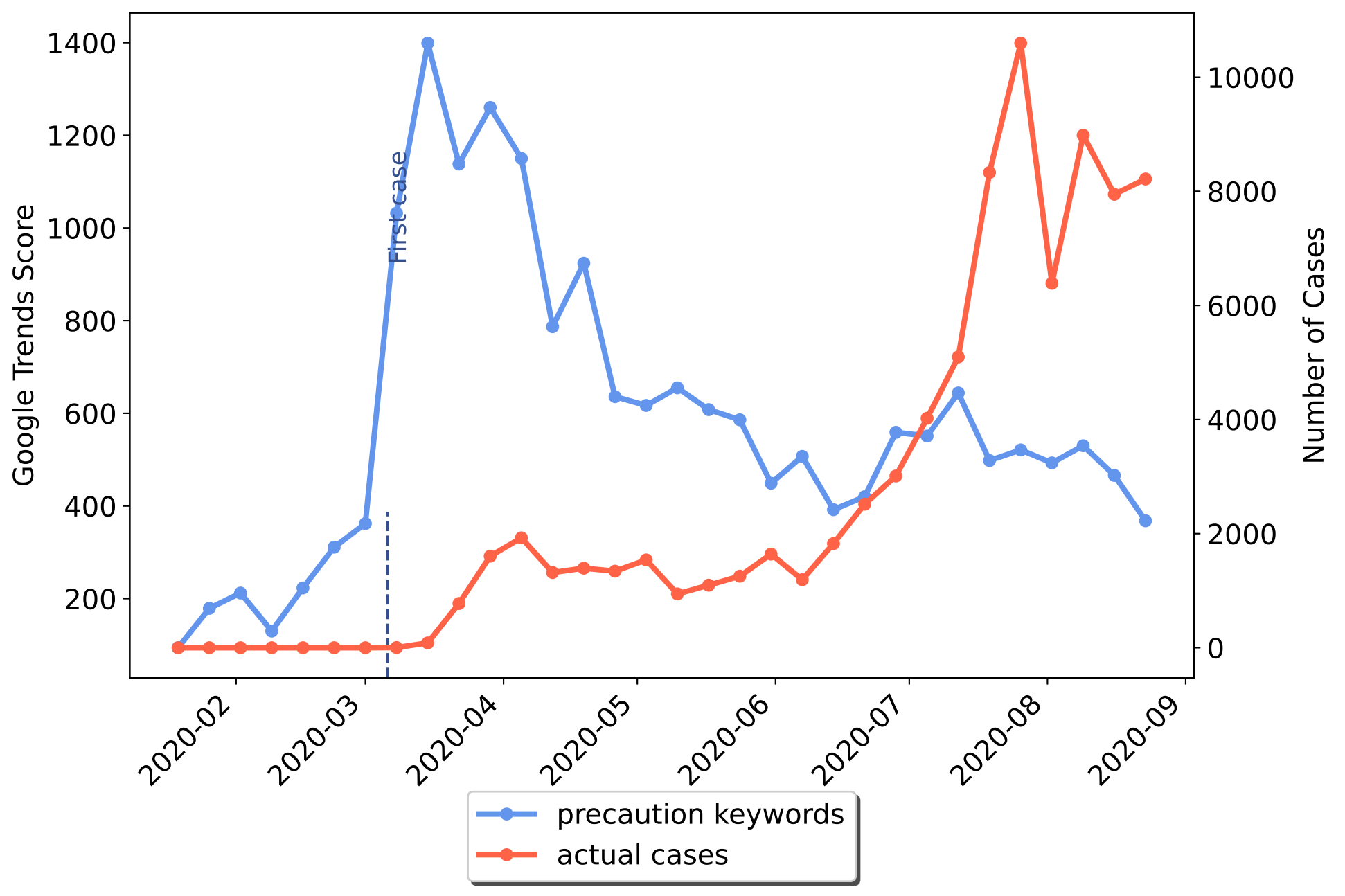

Supplement: Supplementary file 2 [file Data_Sheet_1.ZIP › figures/Missouri_totalprecaution_GT-eps-converted-to.pdf]

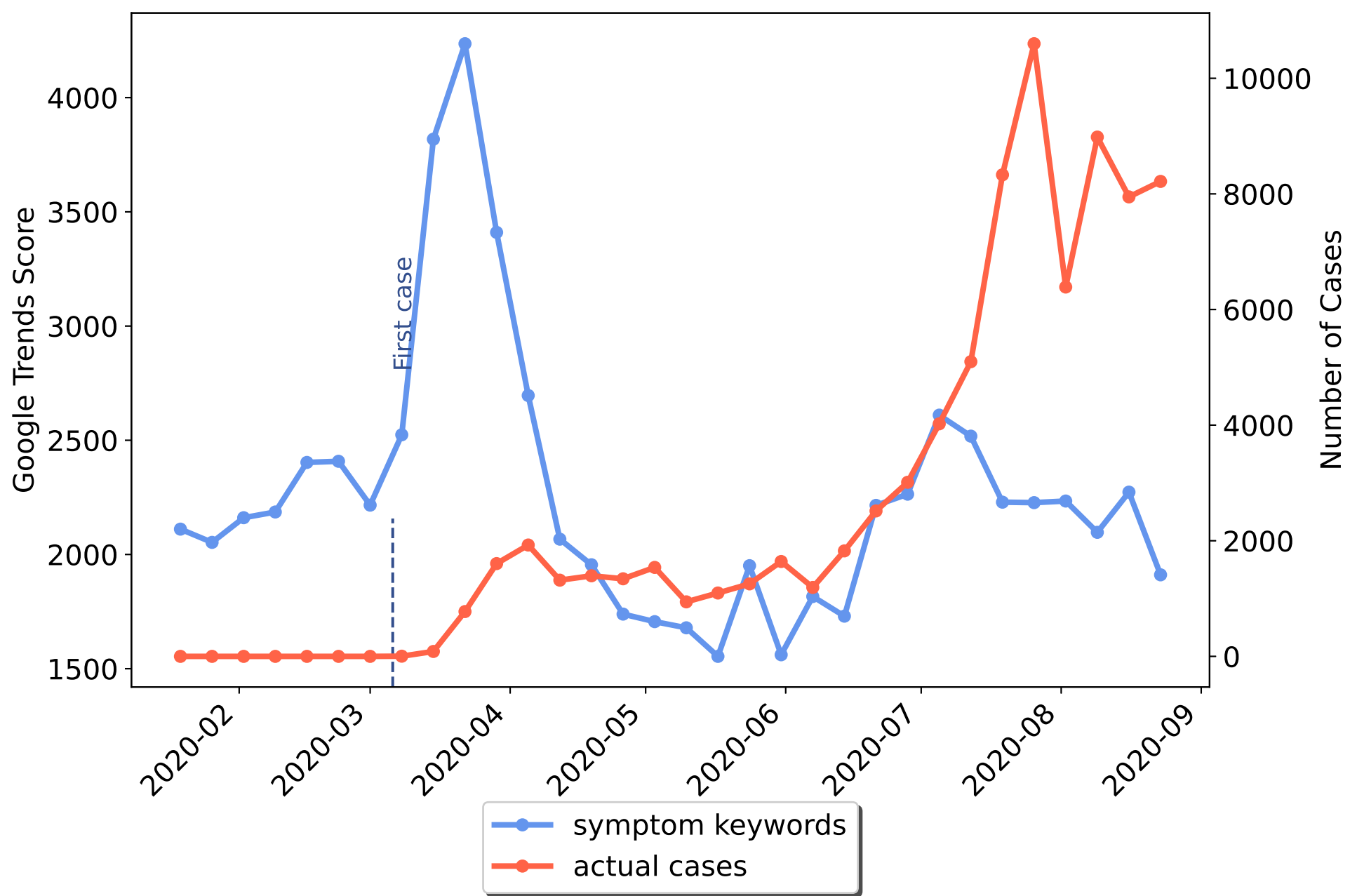

Supplement: Supplementary file 2 [file Data_Sheet_1.ZIP › figures/Missouri_totalsymptom_GT-eps-converted-to.pdf]

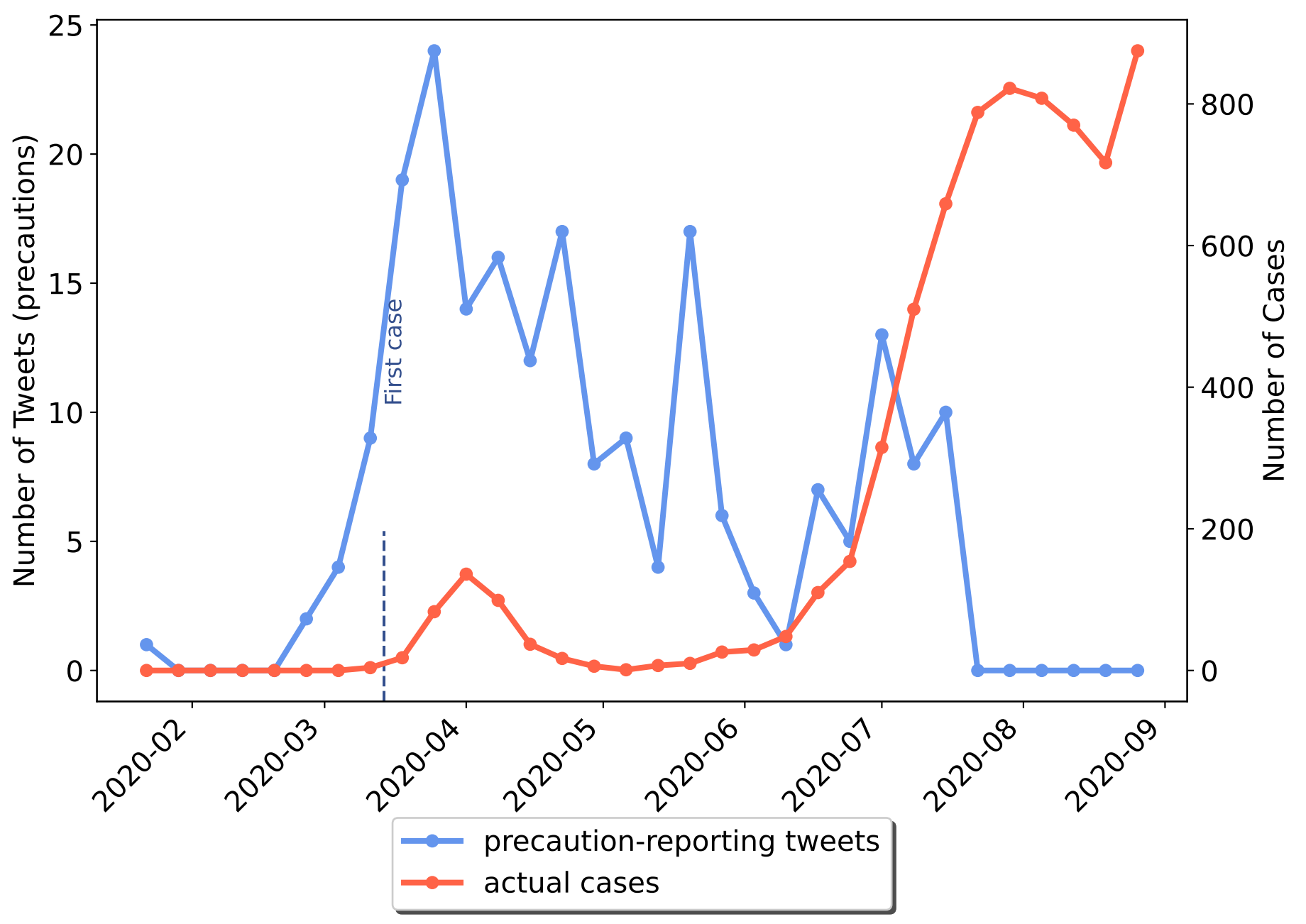

Supplement: Supplementary file 2 [file Data_Sheet_1.ZIP › figures/Montana_precaution_twitter-eps-converted-to.pdf]

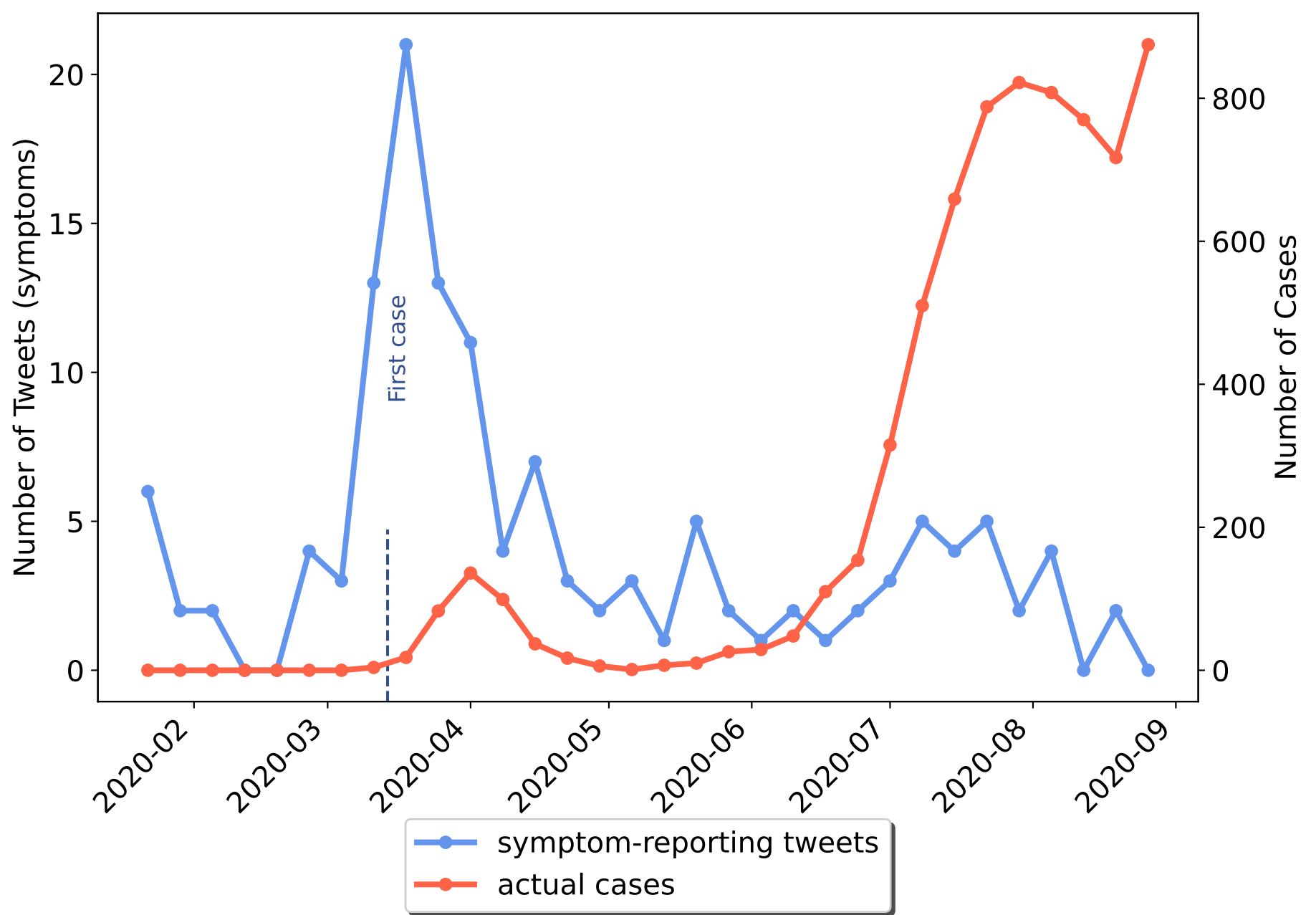

Supplement: Supplementary file 2 [file Data_Sheet_1.ZIP › figures/Montana_symptom_twitter-eps-converted-to.pdf]

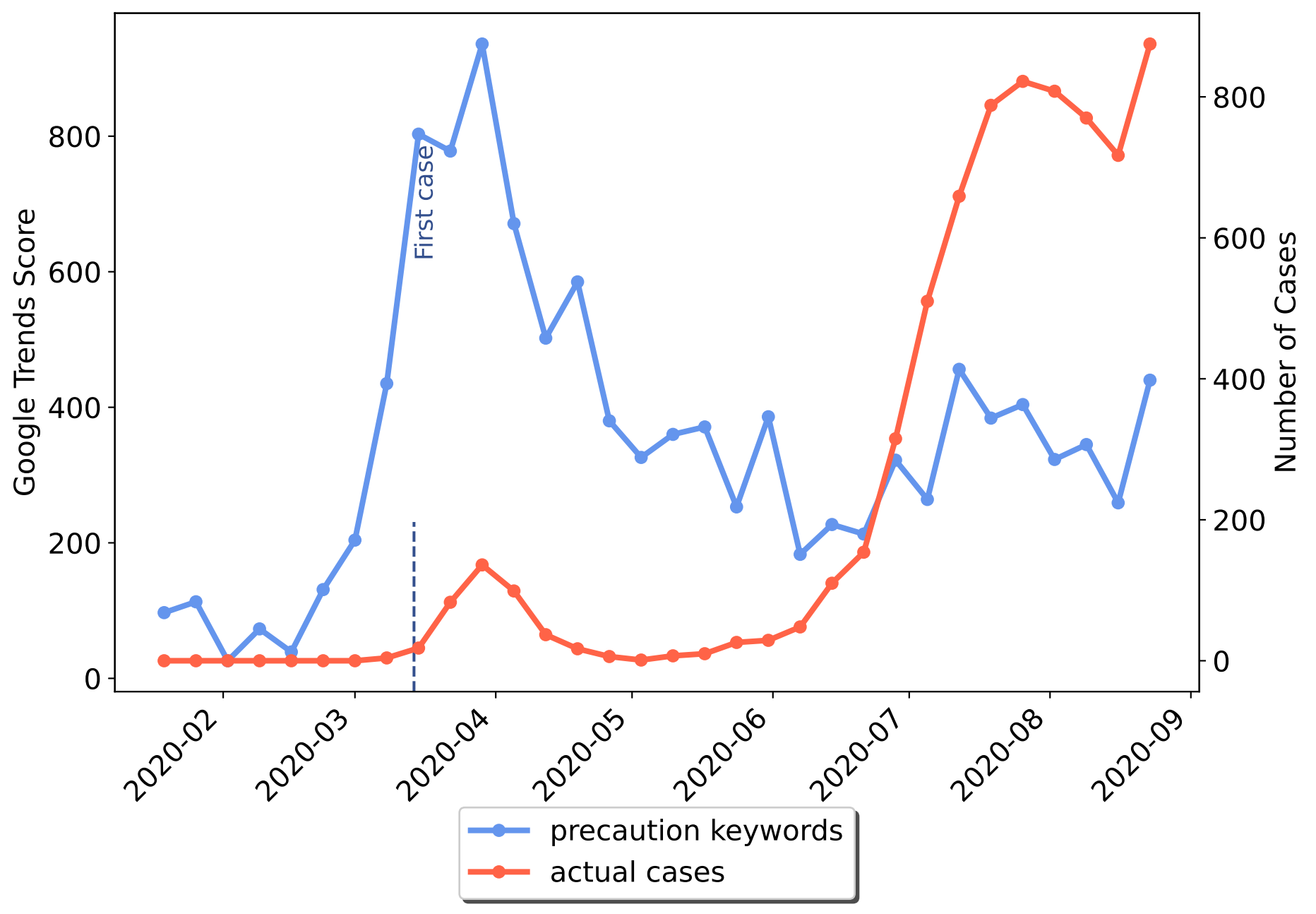

Supplement: Supplementary file 2 [file Data_Sheet_1.ZIP › figures/Montana_totalprecaution_GT-eps-converted-to.pdf]

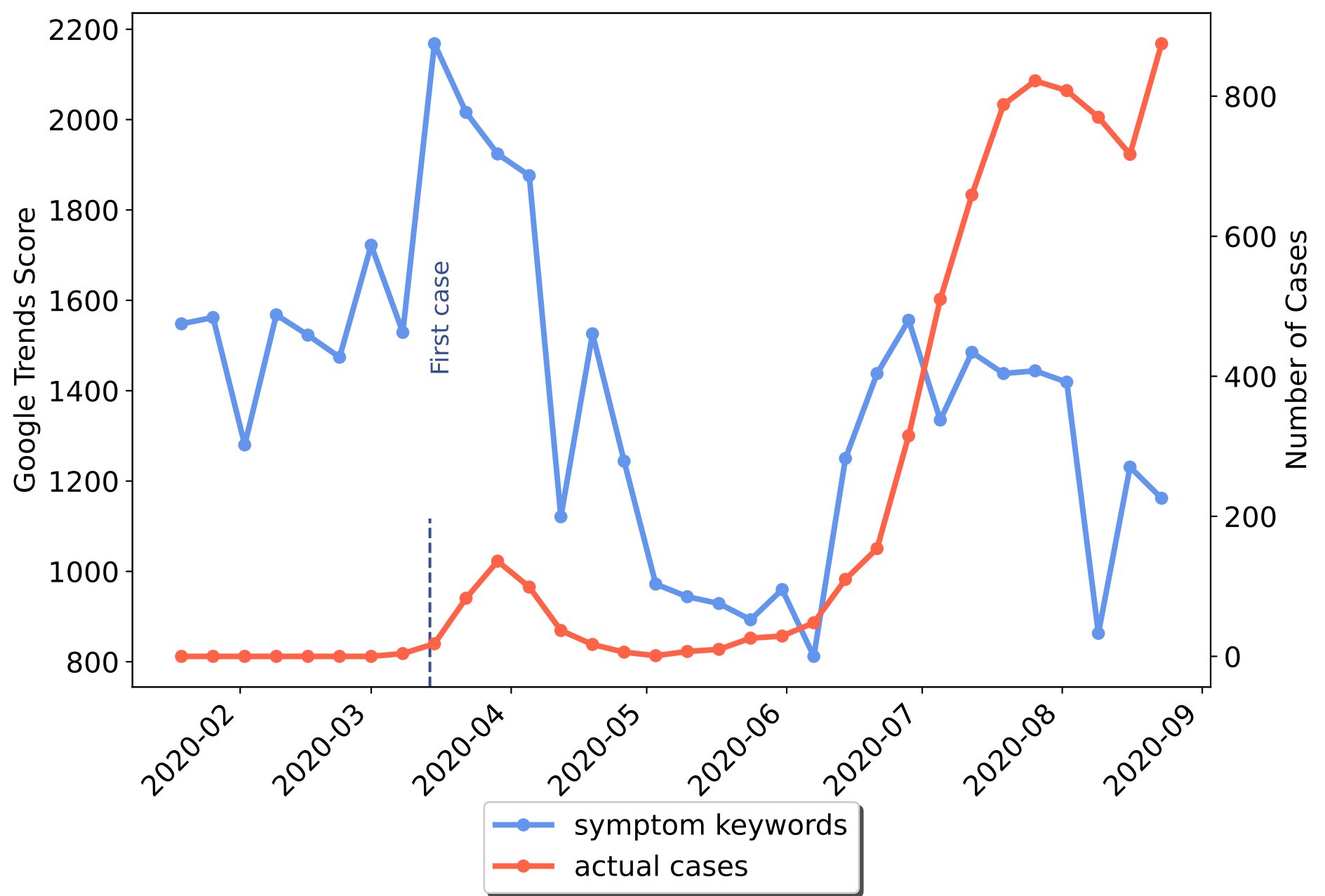

Supplement: Supplementary file 2 [file Data_Sheet_1.ZIP › figures/Montana_totalsymptom_GT-eps-converted-to.pdf]

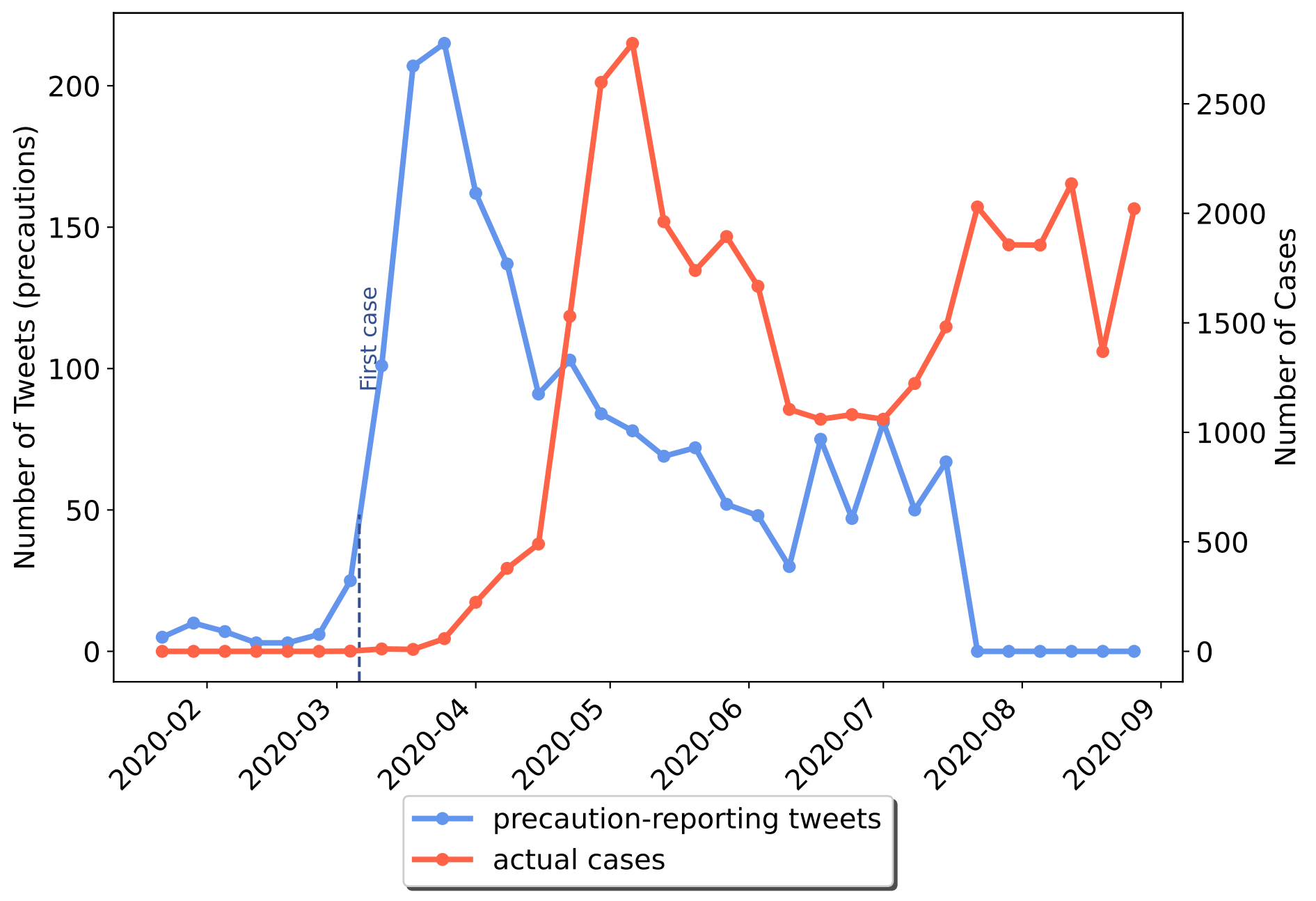

Supplement: Supplementary file 2 [file Data_Sheet_1.ZIP › figures/Nebraska_precaution_twitter-eps-converted-to.pdf]

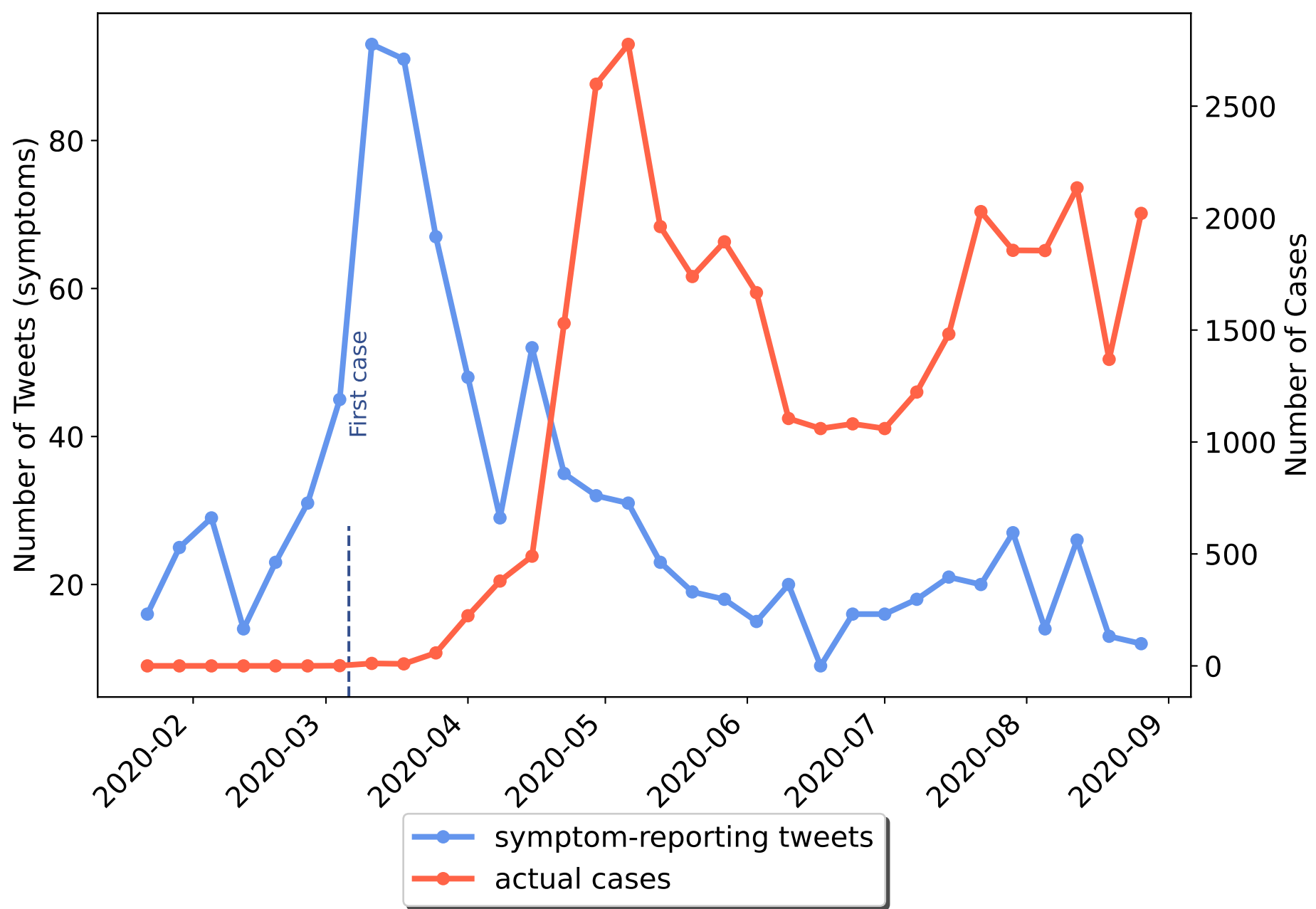

Supplement: Supplementary file 2 [file Data_Sheet_1.ZIP › figures/Nebraska_symptom_twitter-eps-converted-to.pdf]

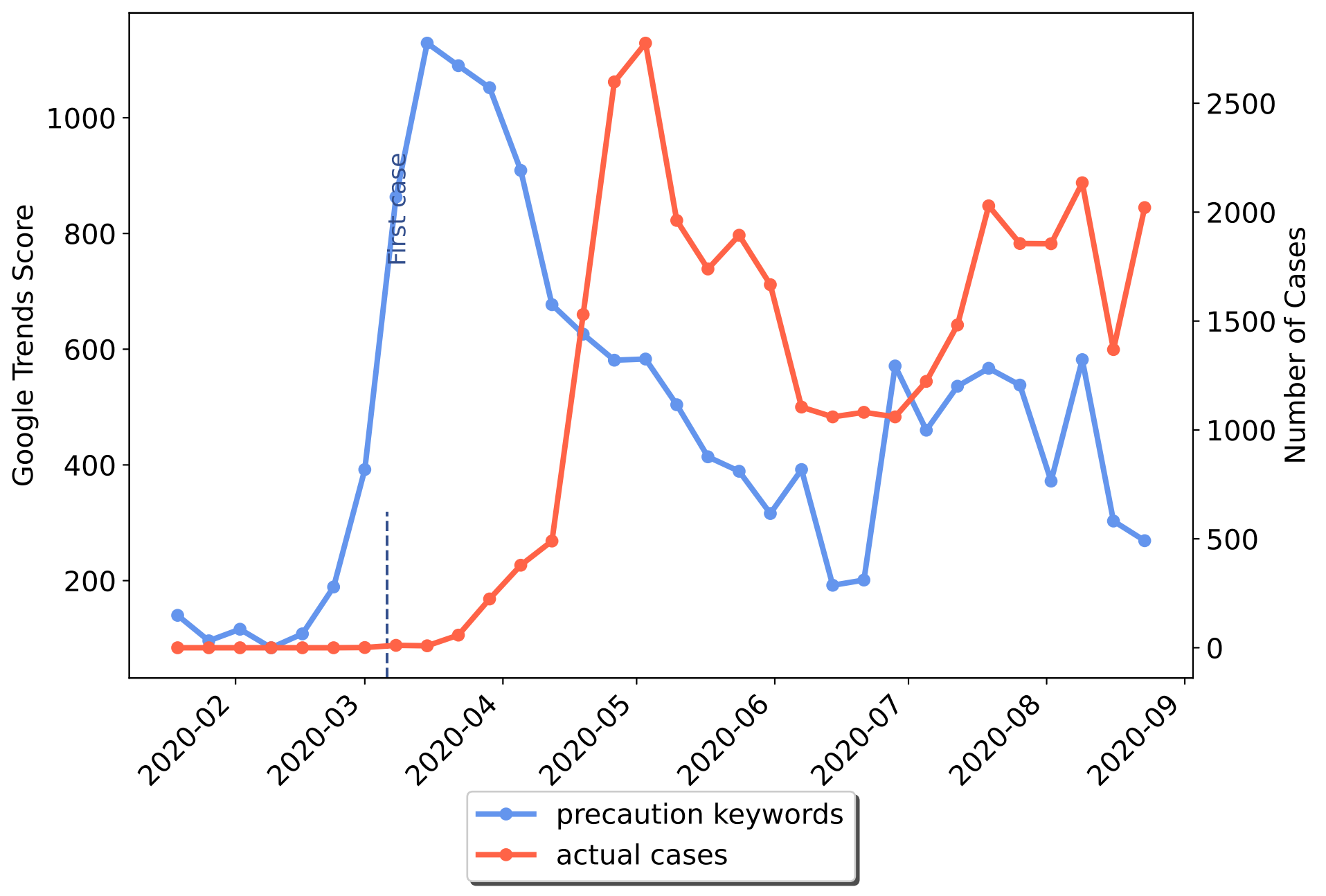

Supplement: Supplementary file 2 [file Data_Sheet_1.ZIP › figures/Nebraska_totalprecaution_GT-eps-converted-to.pdf]

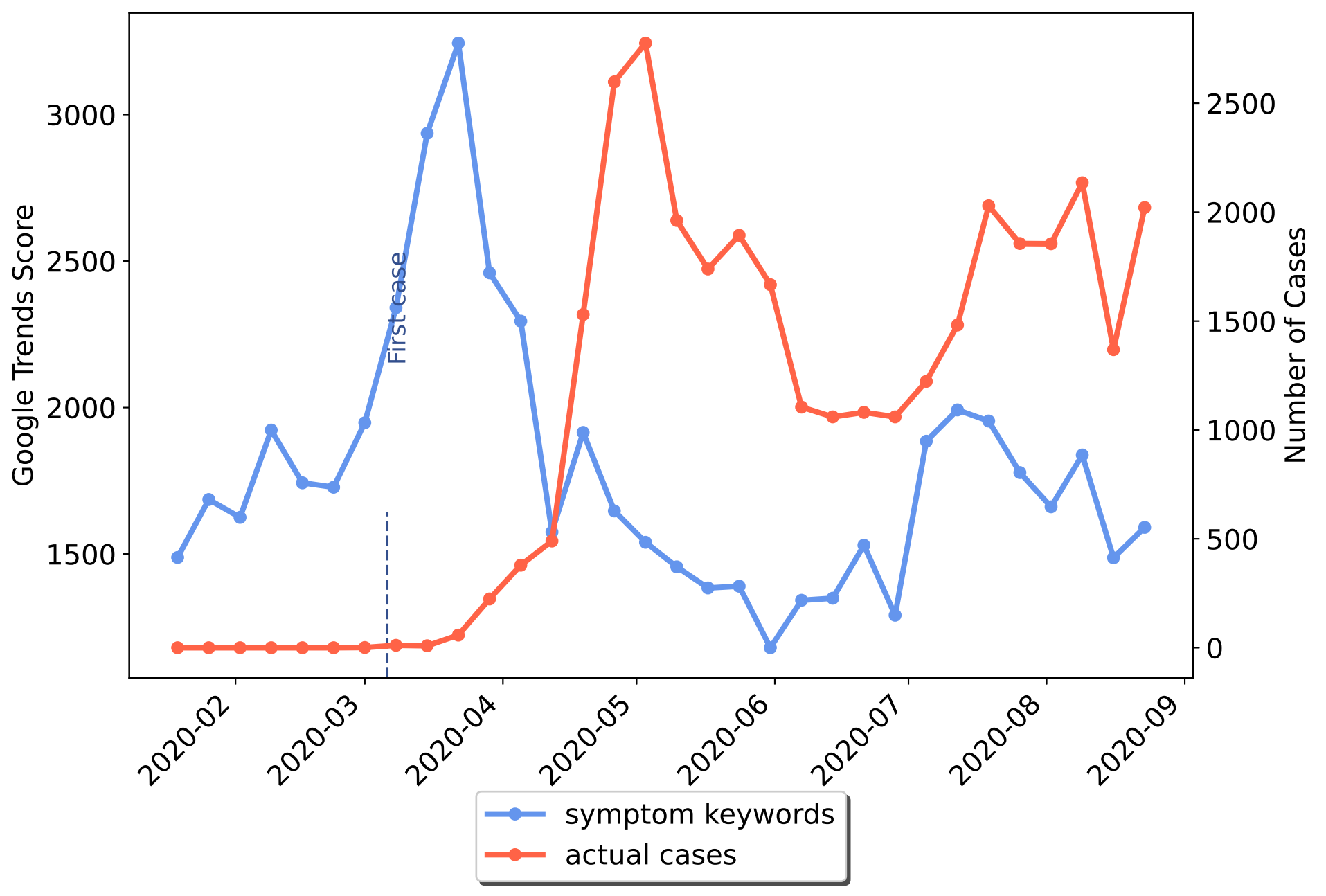

Supplement: Supplementary file 2 [file Data_Sheet_1.ZIP › figures/Nebraska_totalsymptom_GT-eps-converted-to.pdf]

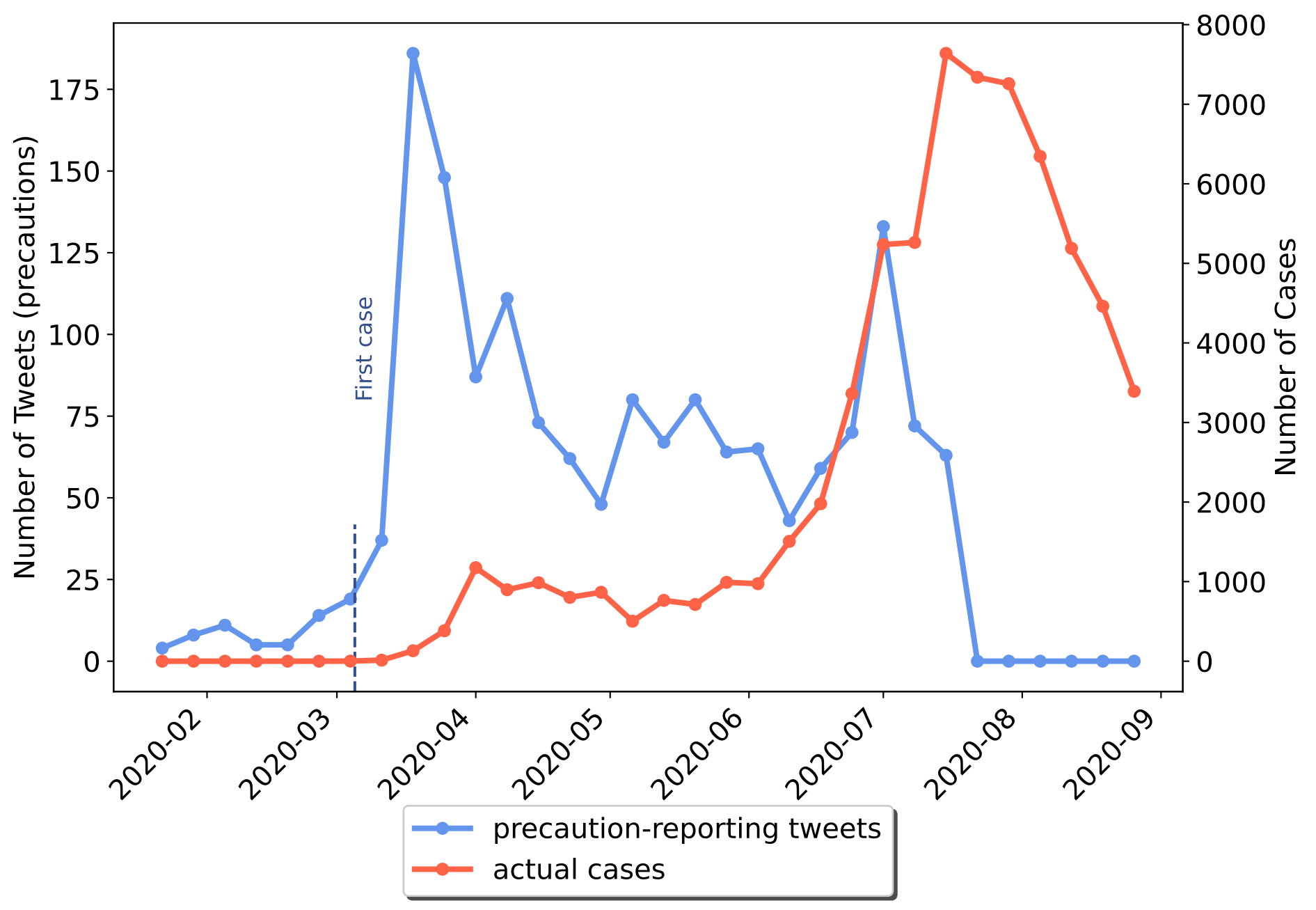

Supplement: Supplementary file 2 [file Data_Sheet_1.ZIP › figures/Nevada_precaution_twitter-eps-converted-to.pdf]

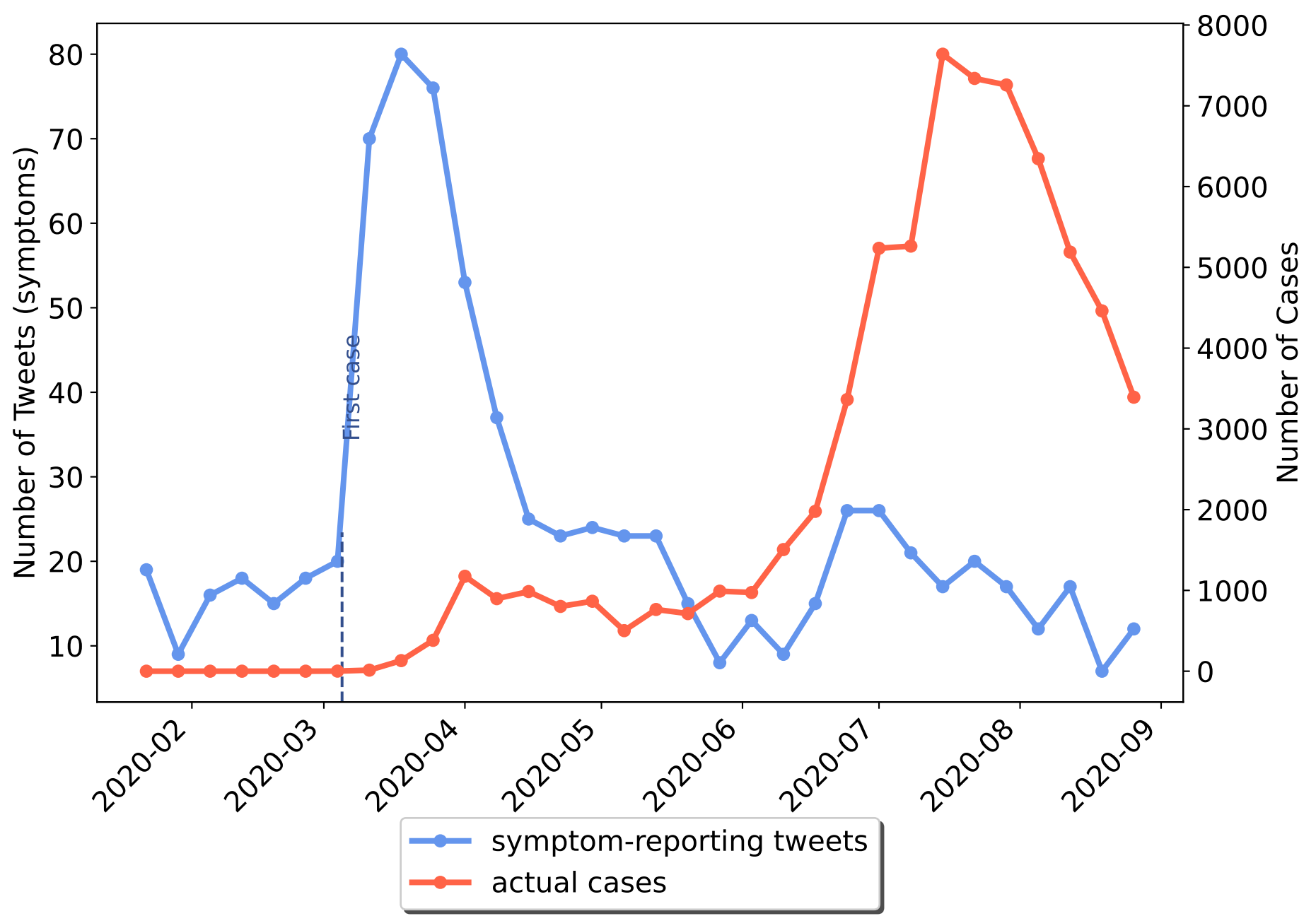

Supplement: Supplementary file 2 [file Data_Sheet_1.ZIP › figures/Nevada_symptom_twitter-eps-converted-to.pdf]

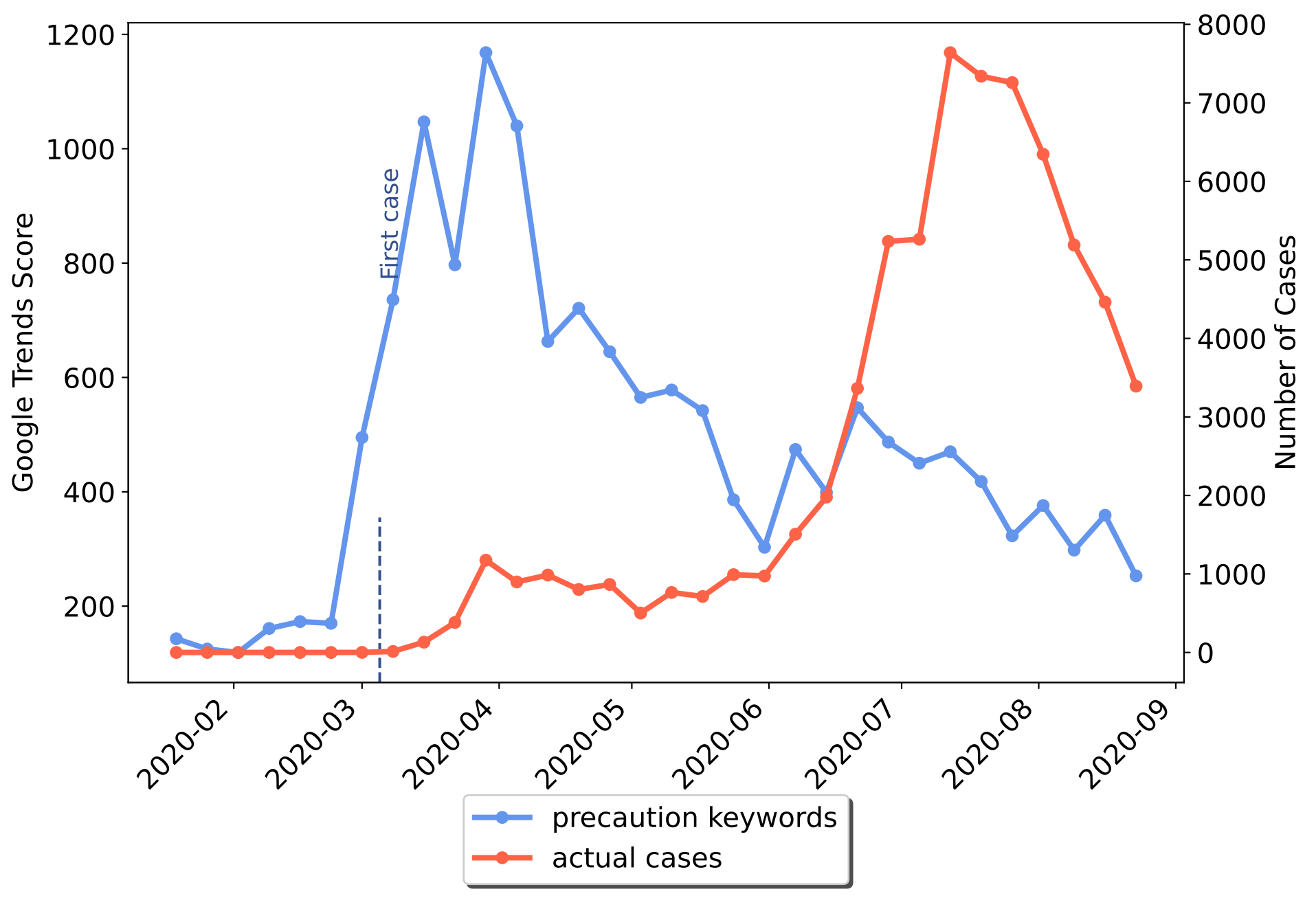

Supplement: Supplementary file 2 [file Data_Sheet_1.ZIP › figures/Nevada_totalprecaution_GT-eps-converted-to.pdf]

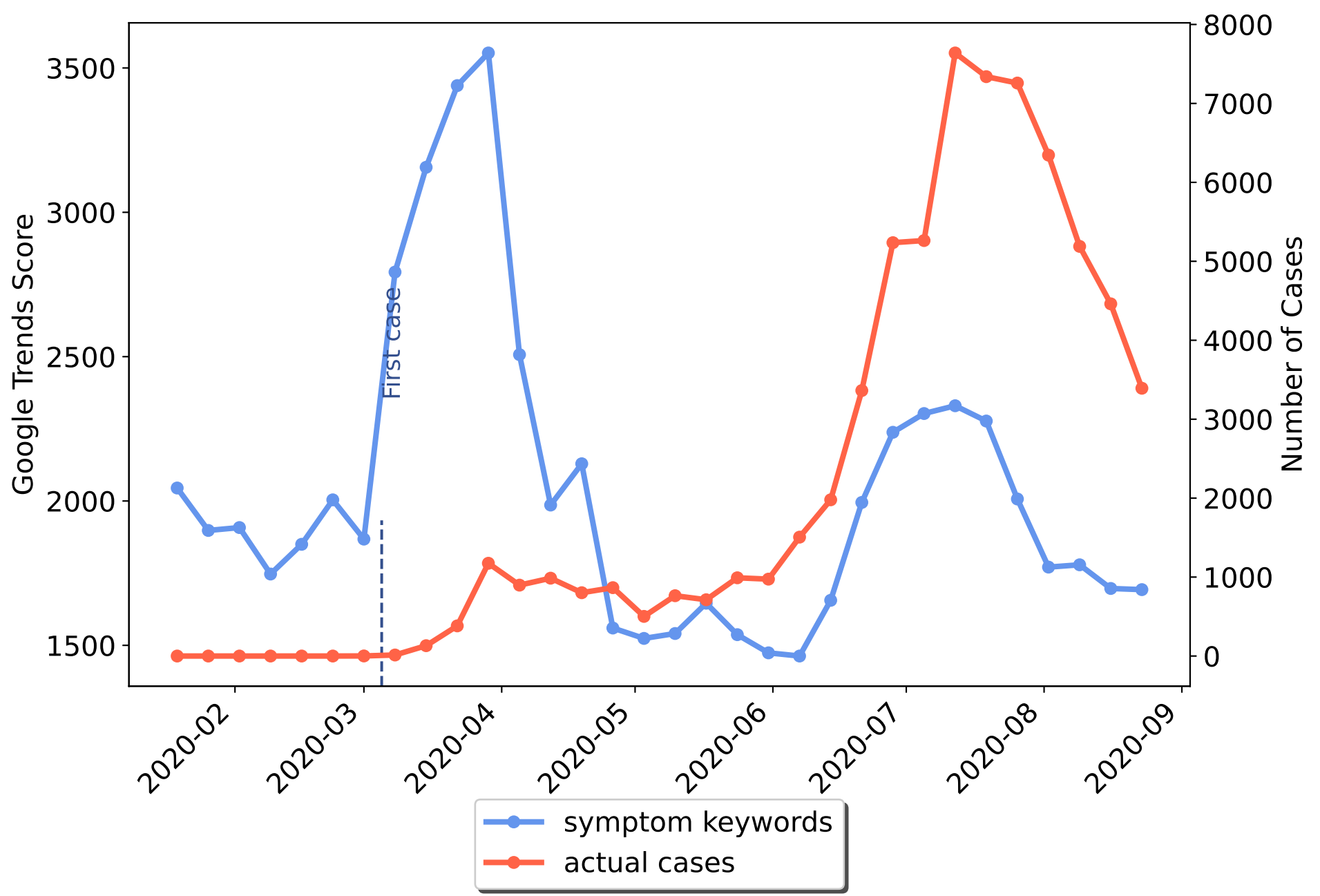

Supplement: Supplementary file 2 [file Data_Sheet_1.ZIP › figures/Nevada_totalsymptom_GT-eps-converted-to.pdf]

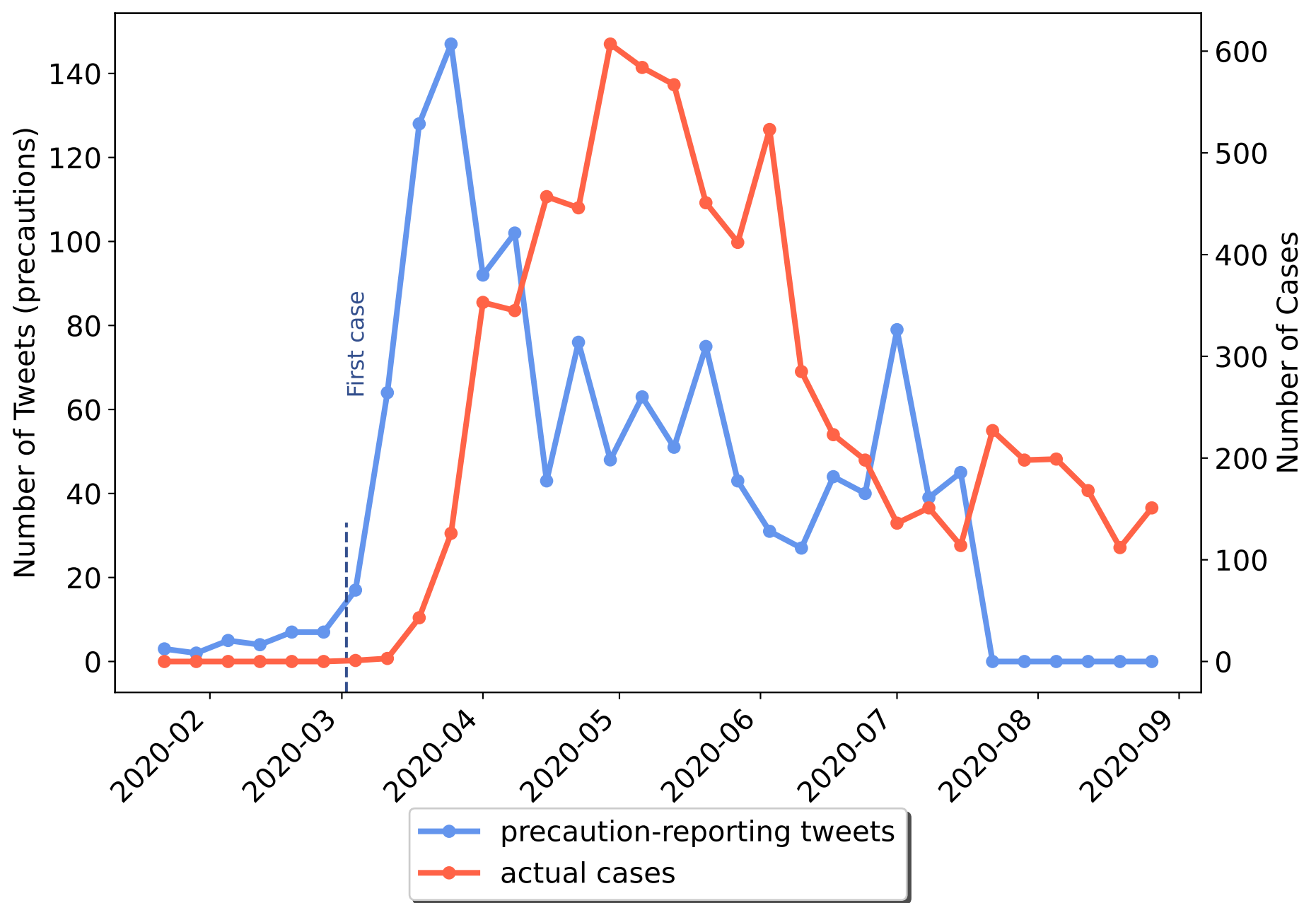

Supplement: Supplementary file 2 [file Data_Sheet_1.ZIP › figures/New_Hampshire_precaution_twitter-eps-converted-to.pdf]

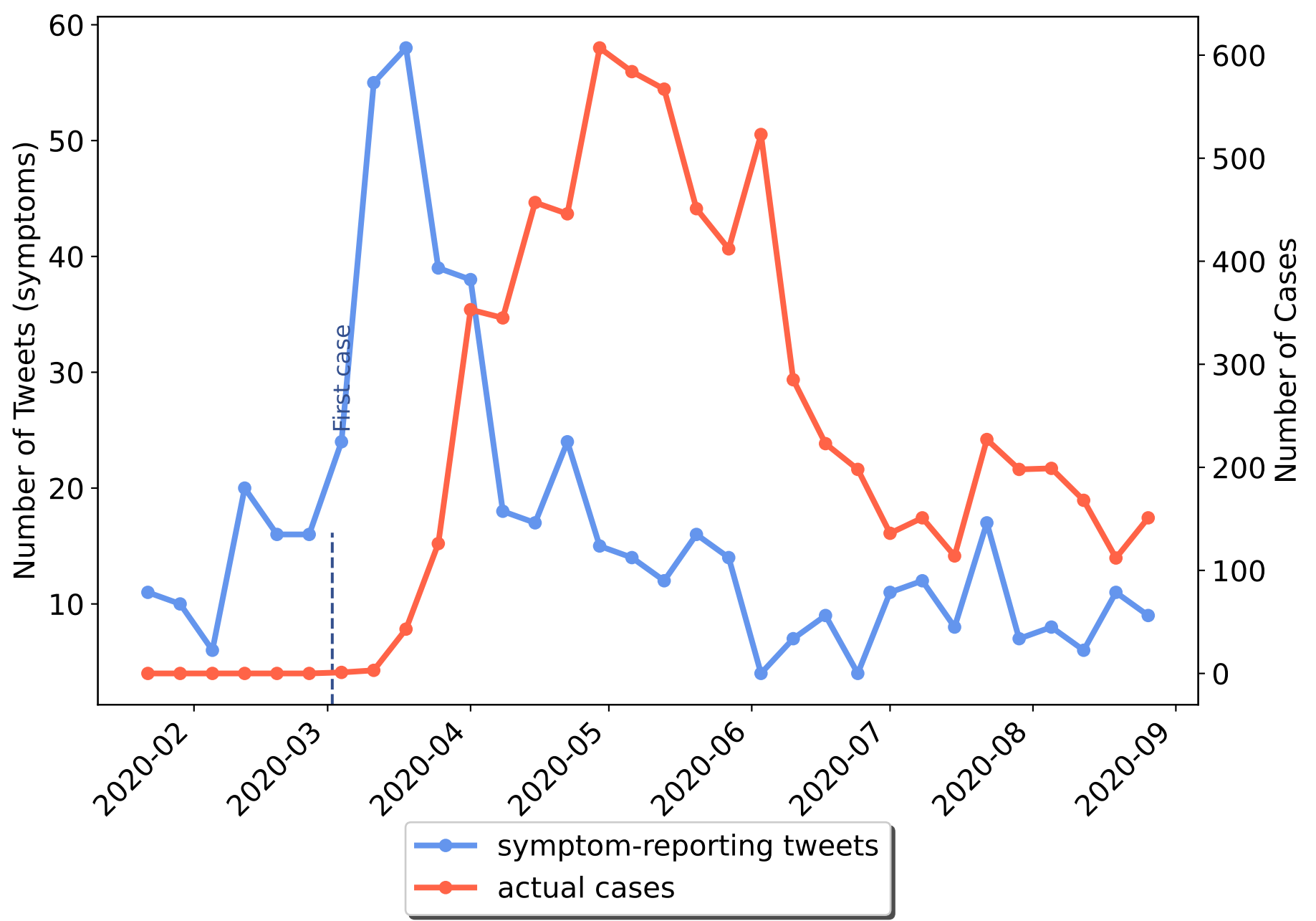

Supplement: Supplementary file 2 [file Data_Sheet_1.ZIP › figures/New_Hampshire_symptom_twitter-eps-converted-to.pdf]

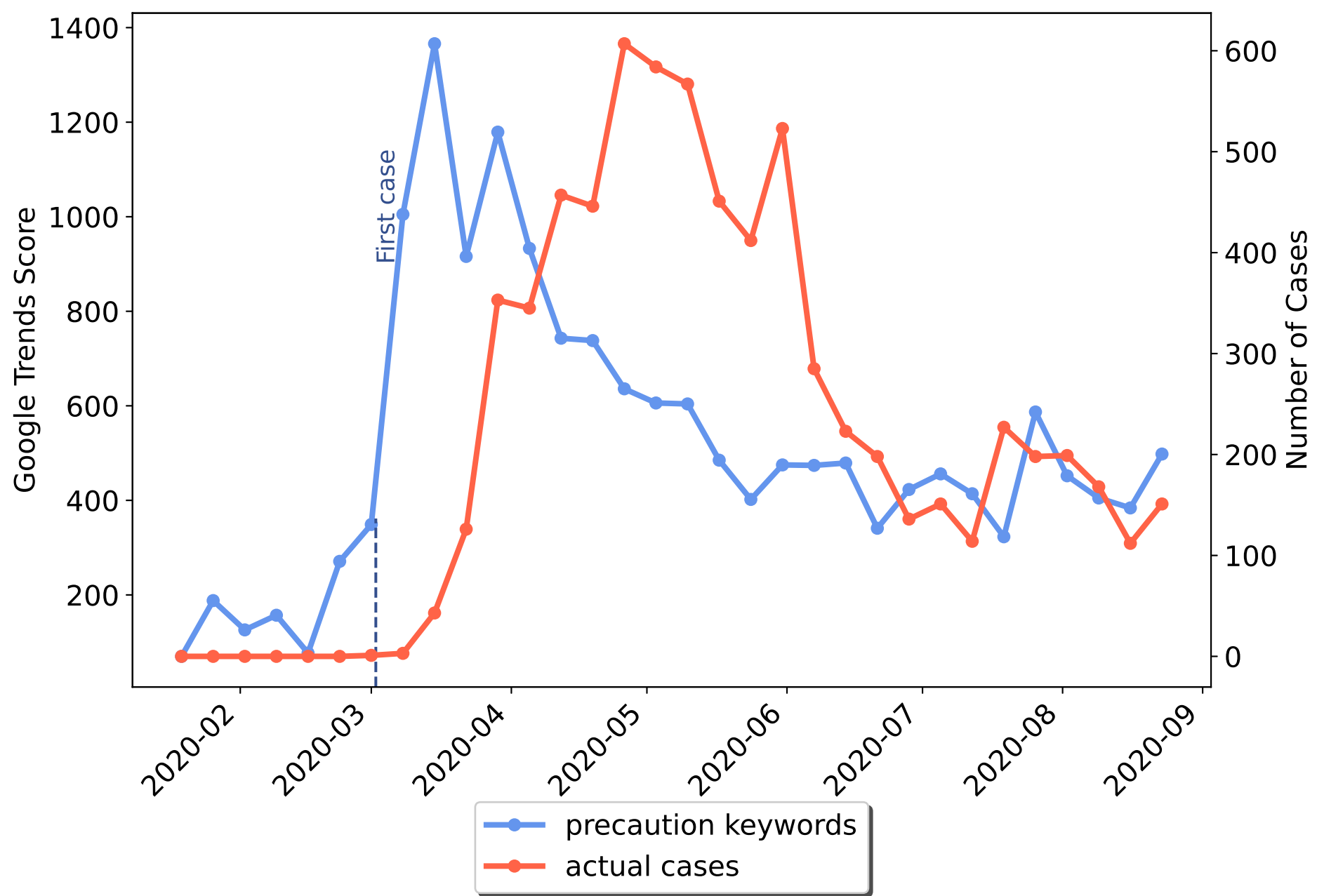

Supplement: Supplementary file 2 [file Data_Sheet_1.ZIP › figures/New_Hampshire_totalprecaution_GT-eps-converted-to.pdf]

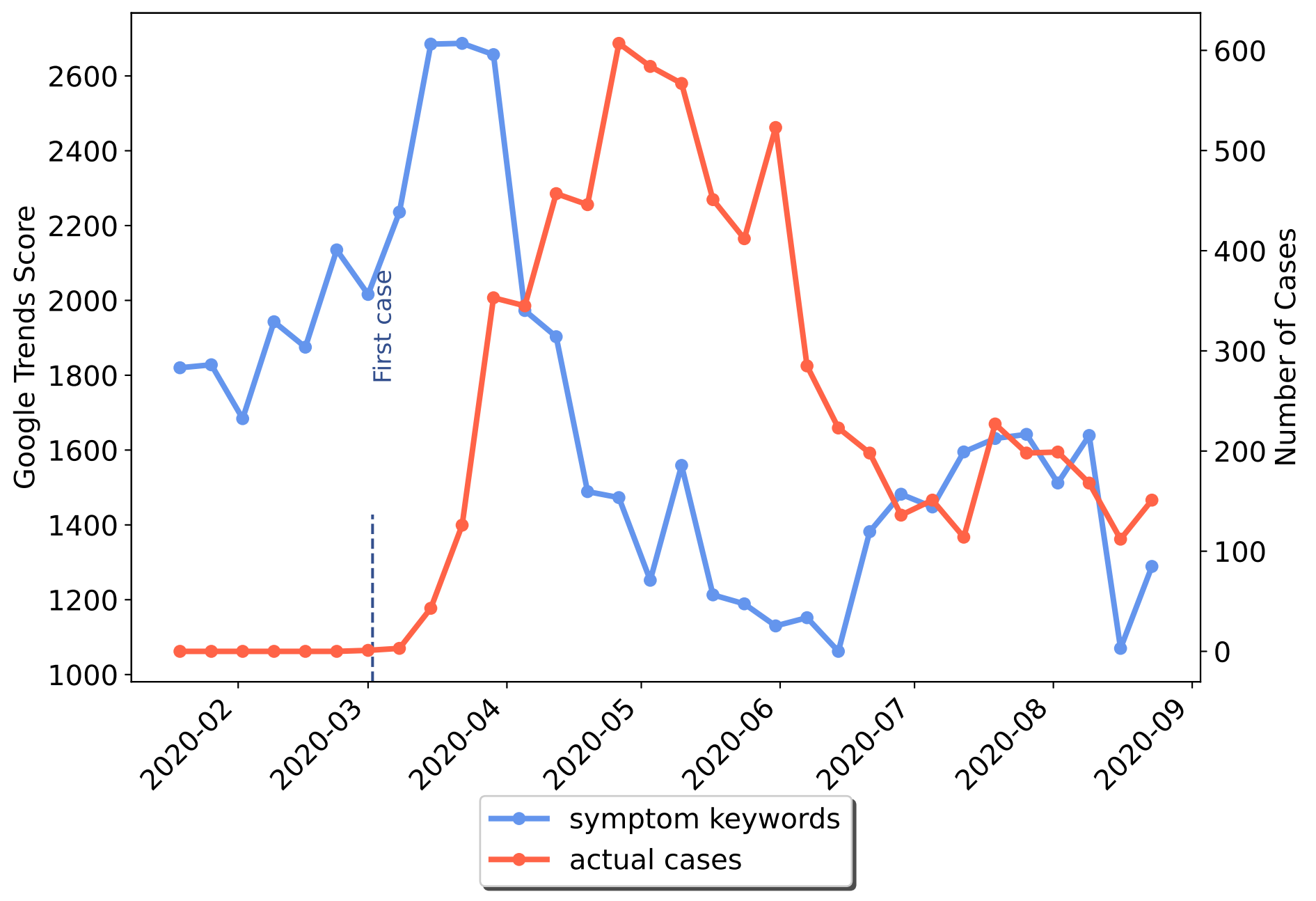

Supplement: Supplementary file 2 [file Data_Sheet_1.ZIP › figures/New_Hampshire_totalsymptom_GT-eps-converted-to.pdf]

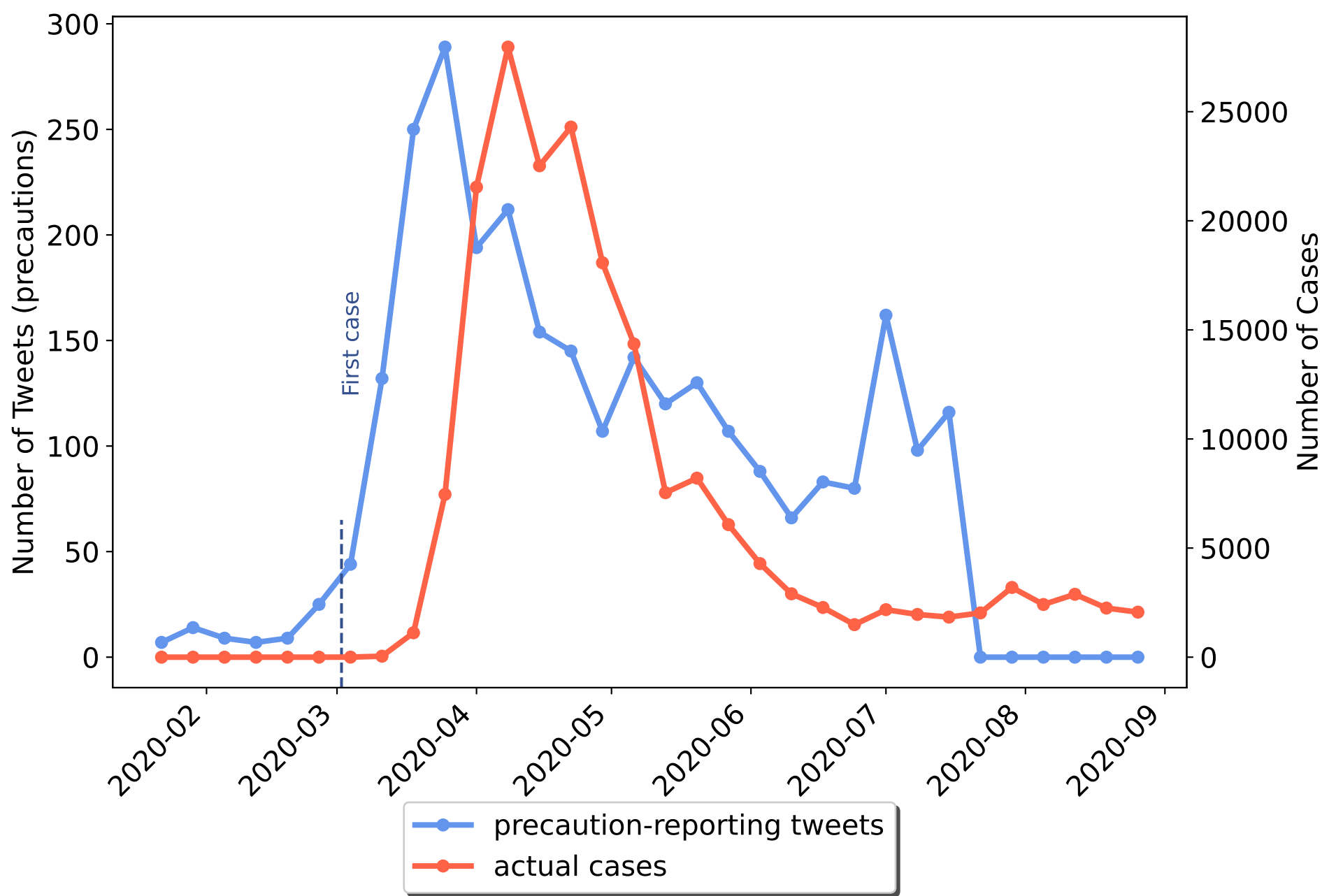

Supplement: Supplementary file 2 [file Data_Sheet_1.ZIP › figures/New_Jersey_precaution_twitter-eps-converted-to.pdf]

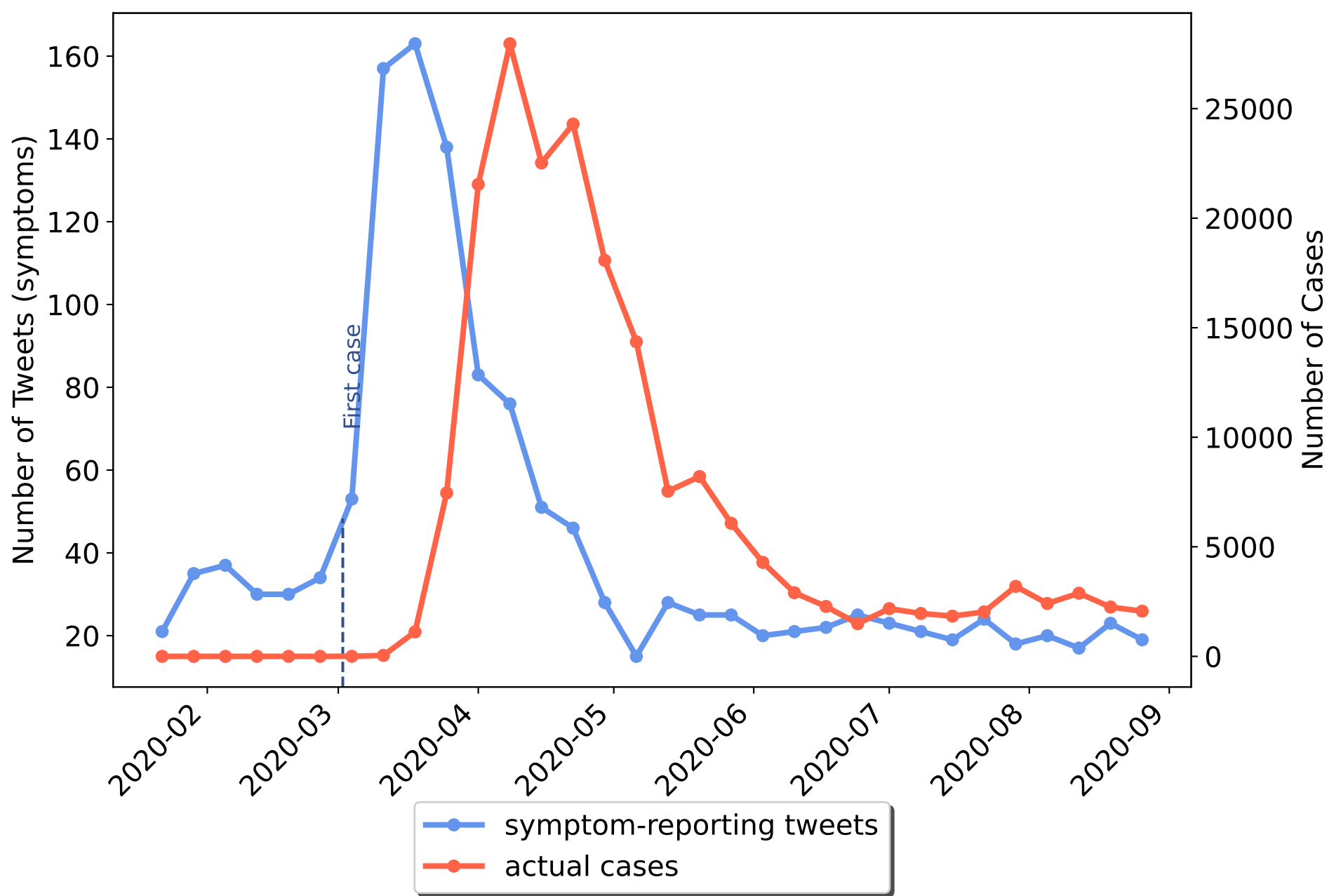

Supplement: Supplementary file 2 [file Data_Sheet_1.ZIP › figures/New_Jersey_symptom_twitter-eps-converted-to.pdf]

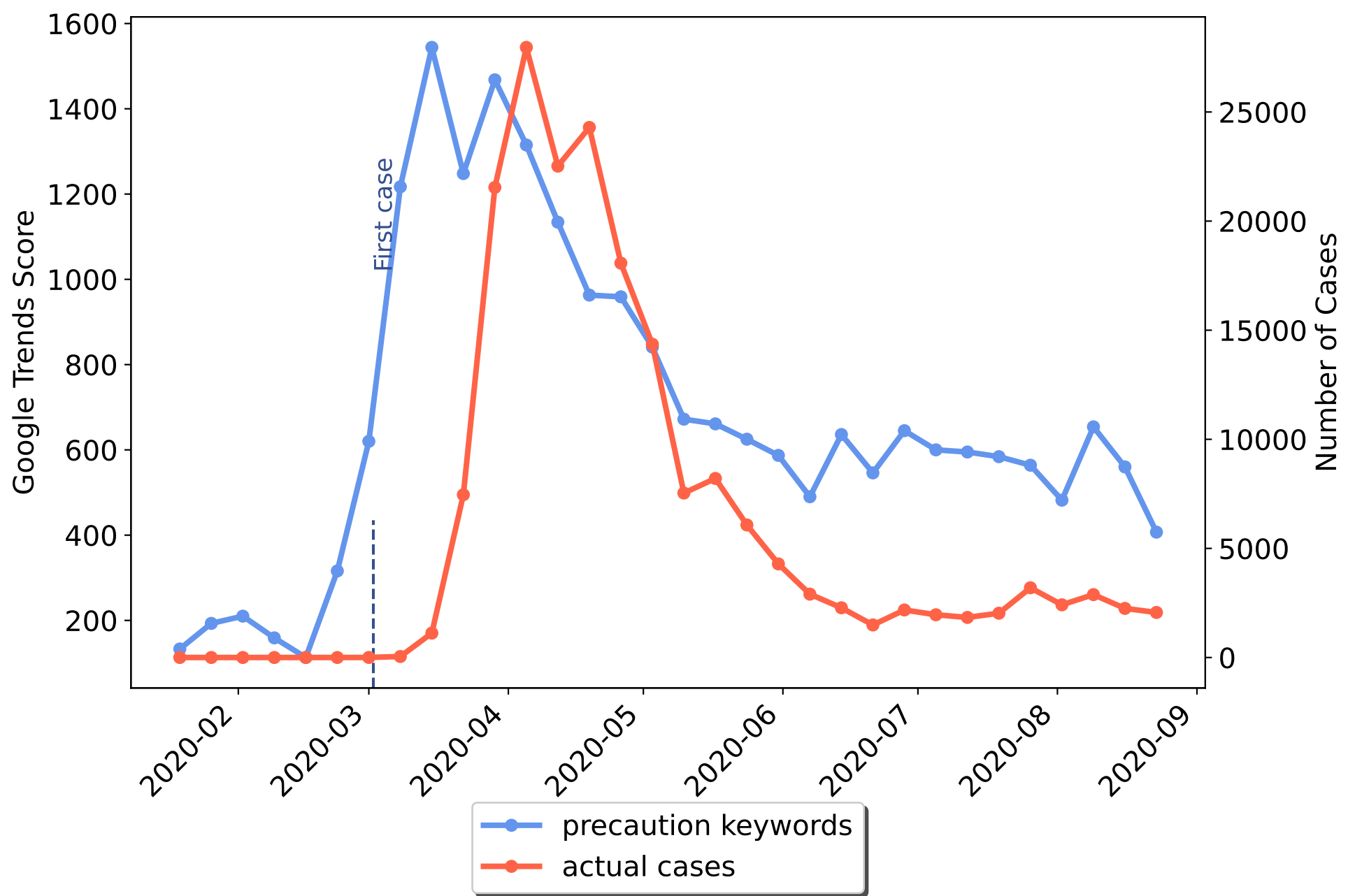

Supplement: Supplementary file 2 [file Data_Sheet_1.ZIP › figures/New_Jersey_totalprecaution_GT-eps-converted-to.pdf]

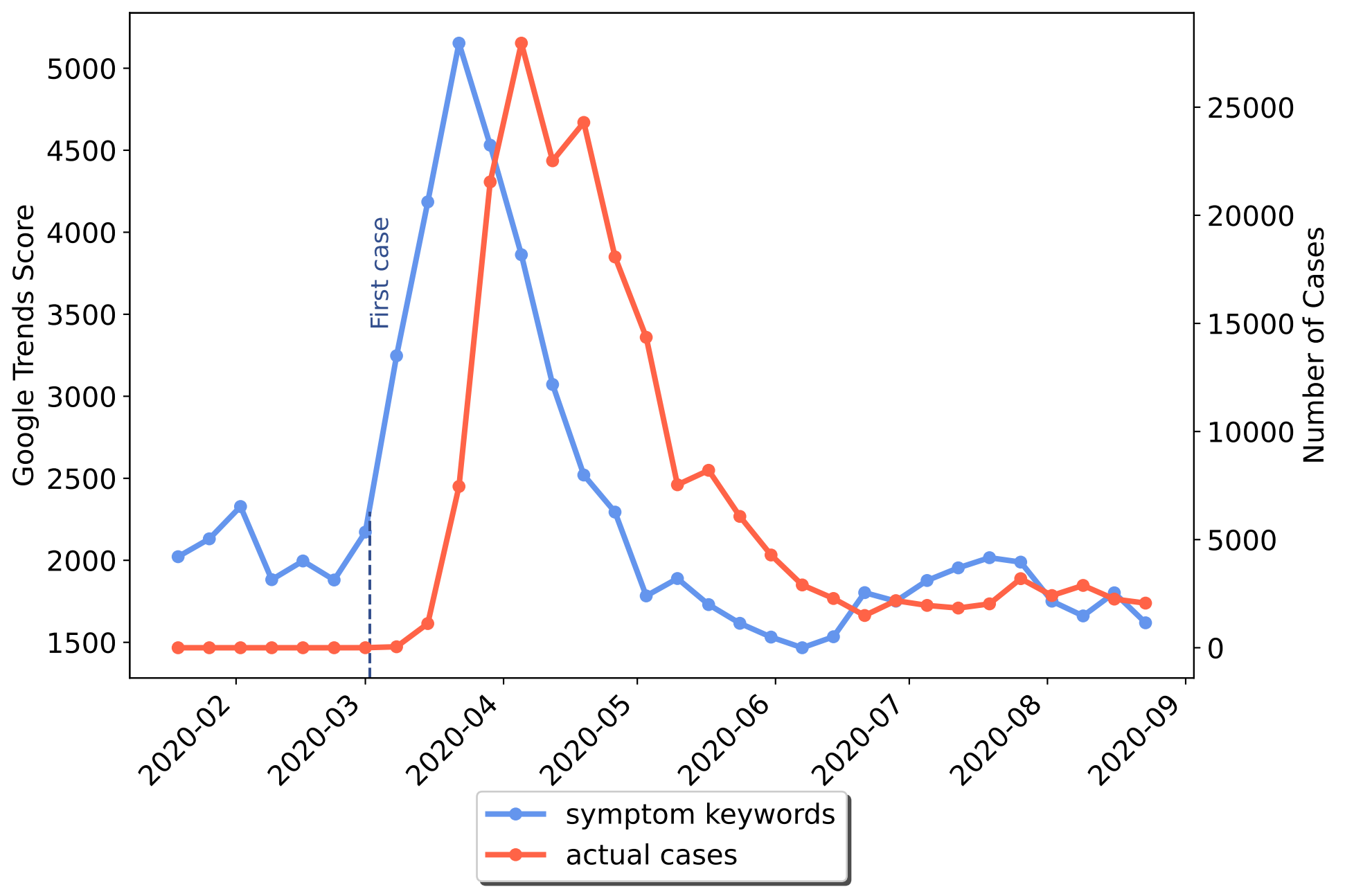

Supplement: Supplementary file 2 [file Data_Sheet_1.ZIP › figures/New_Jersey_totalsymptom_GT-eps-converted-to.pdf]

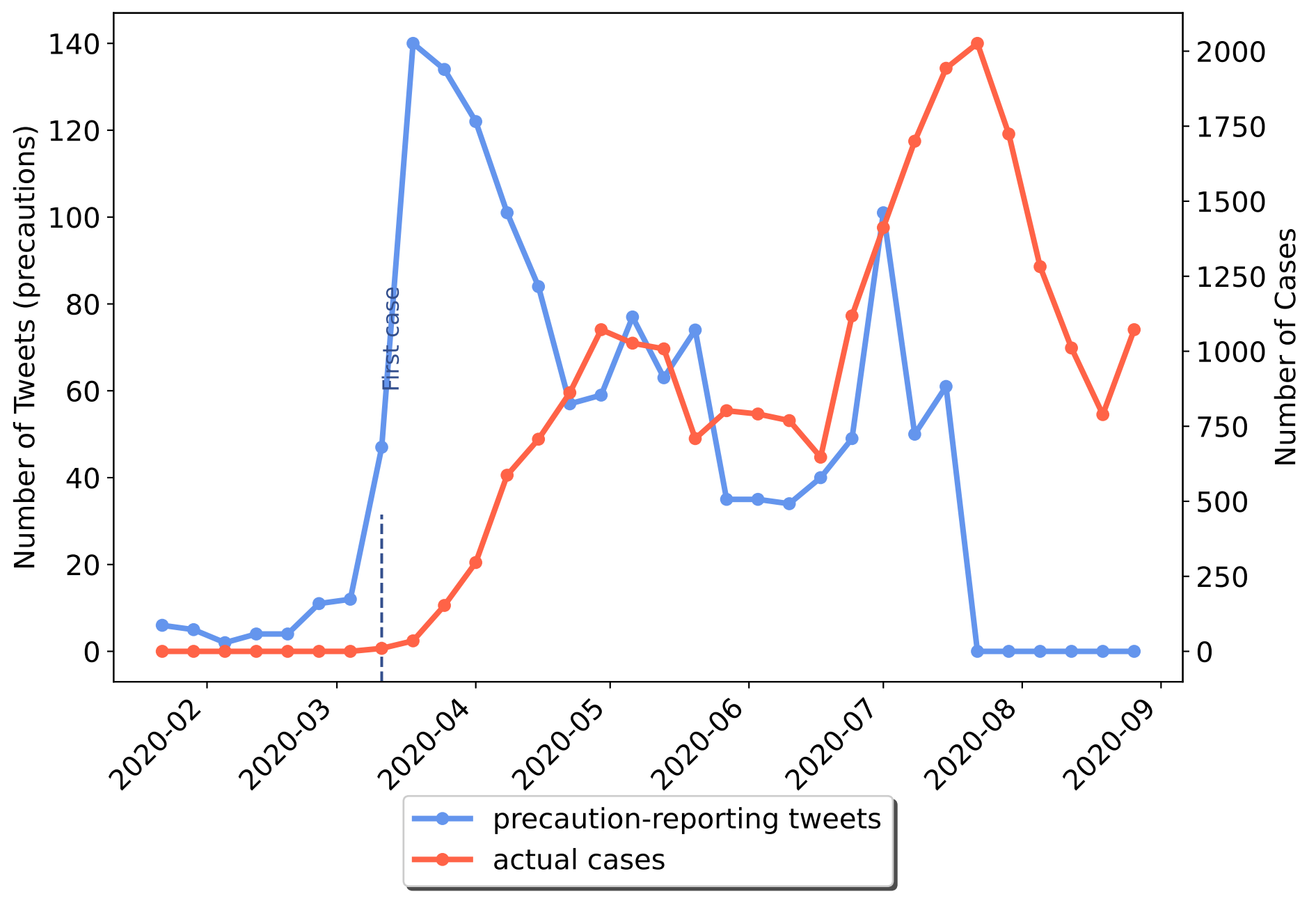

Supplement: Supplementary file 2 [file Data_Sheet_1.ZIP › figures/New_Mexico_precaution_twitter-eps-converted-to.pdf]

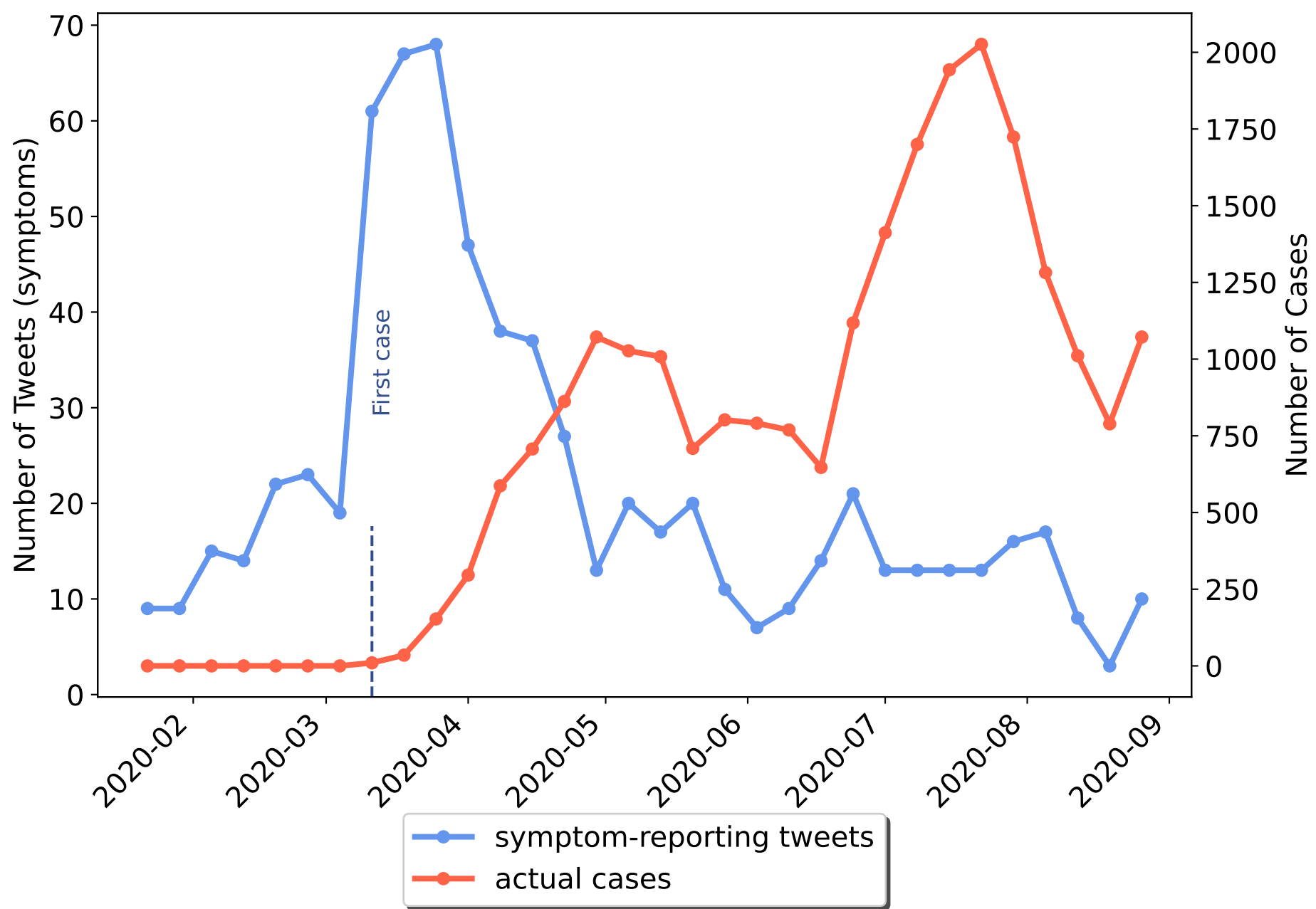

Supplement: Supplementary file 2 [file Data_Sheet_1.ZIP › figures/New_Mexico_symptom_twitter-eps-converted-to.pdf]

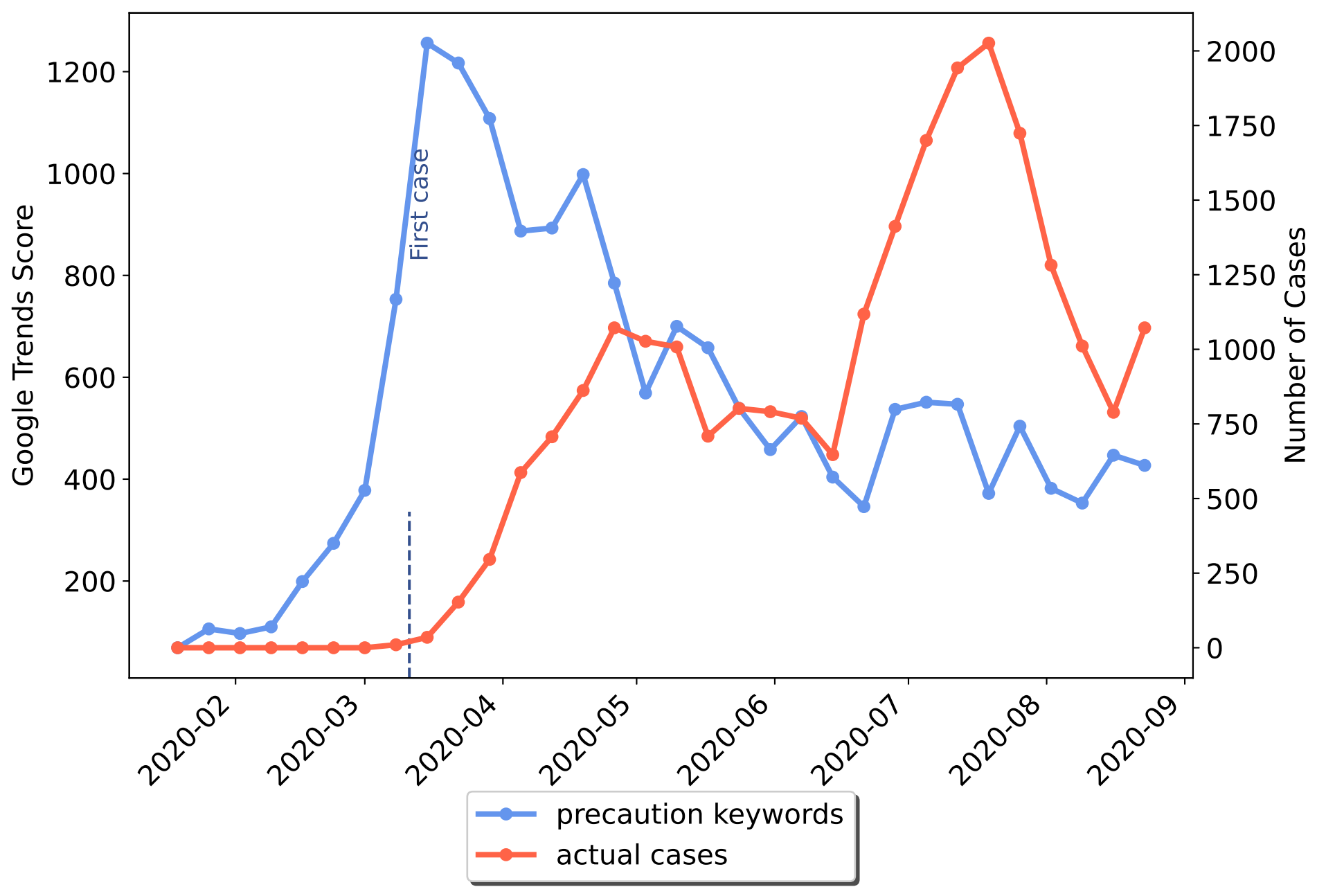

Supplement: Supplementary file 2 [file Data_Sheet_1.ZIP › figures/New_Mexico_totalprecaution_GT-eps-converted-to.pdf]

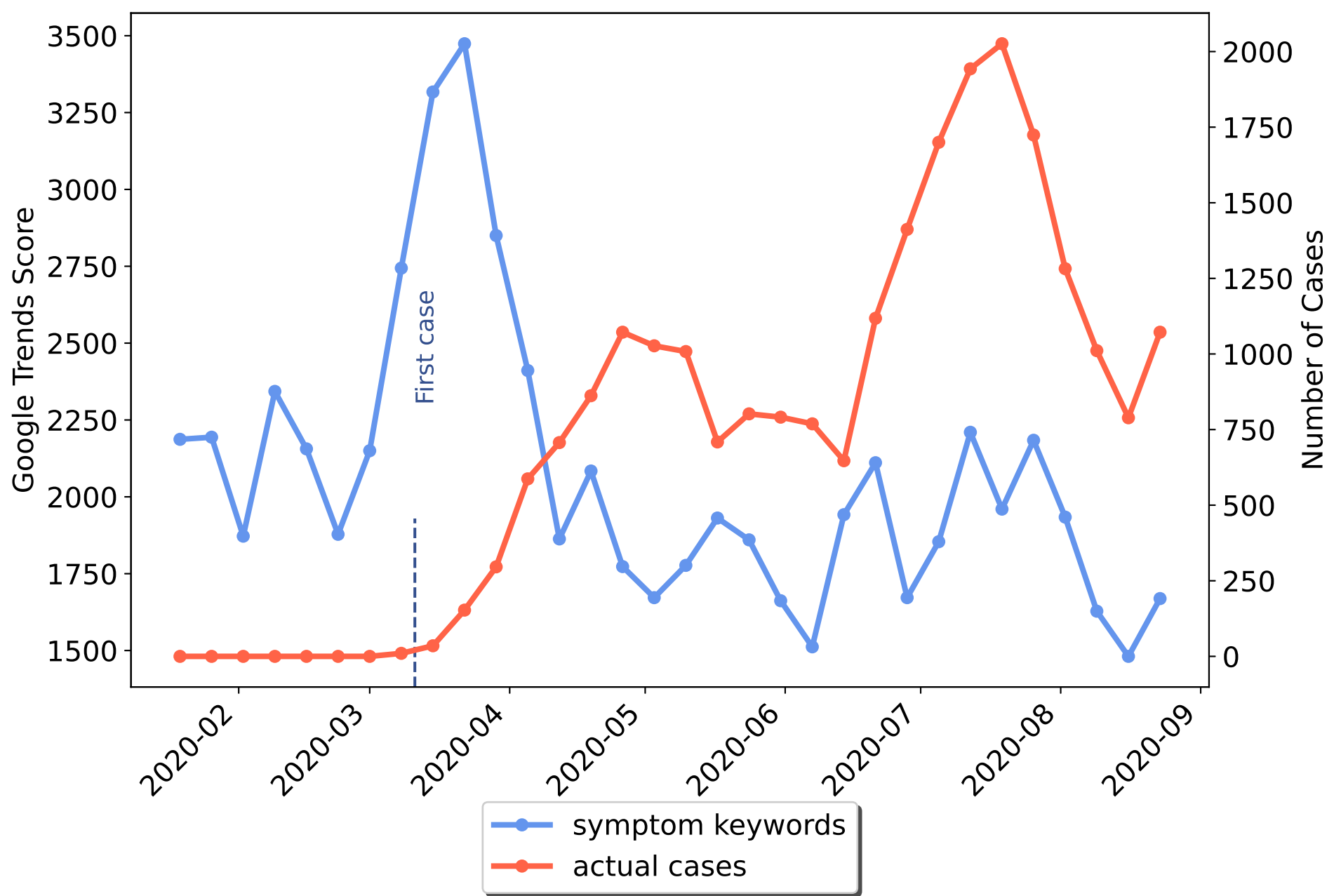

Supplement: Supplementary file 2 [file Data_Sheet_1.ZIP › figures/New_Mexico_totalsymptom_GT-eps-converted-to.pdf]

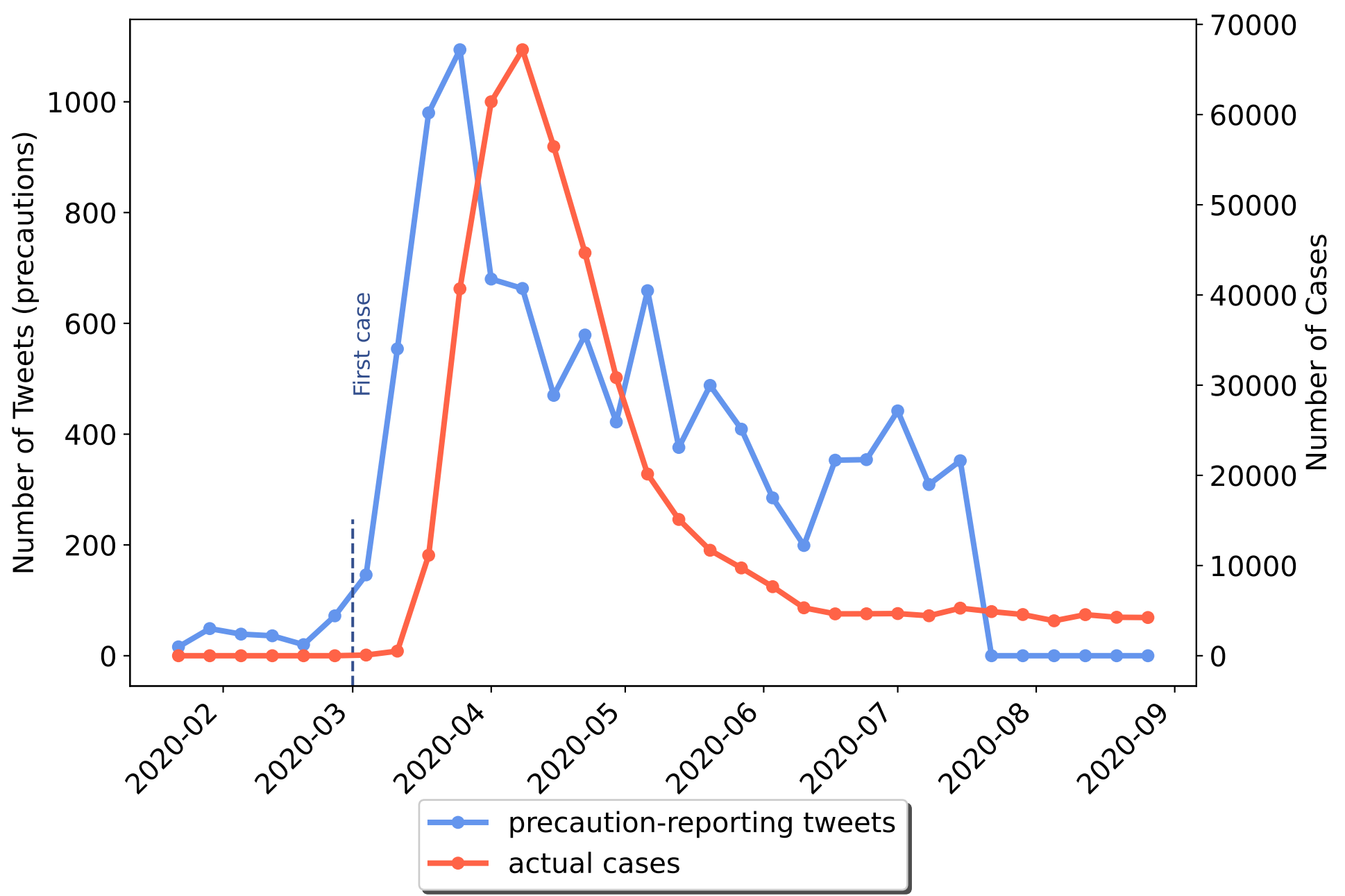

Supplement: Supplementary file 2 [file Data_Sheet_1.ZIP › figures/New_York_precaution_twitter-eps-converted-to.pdf]

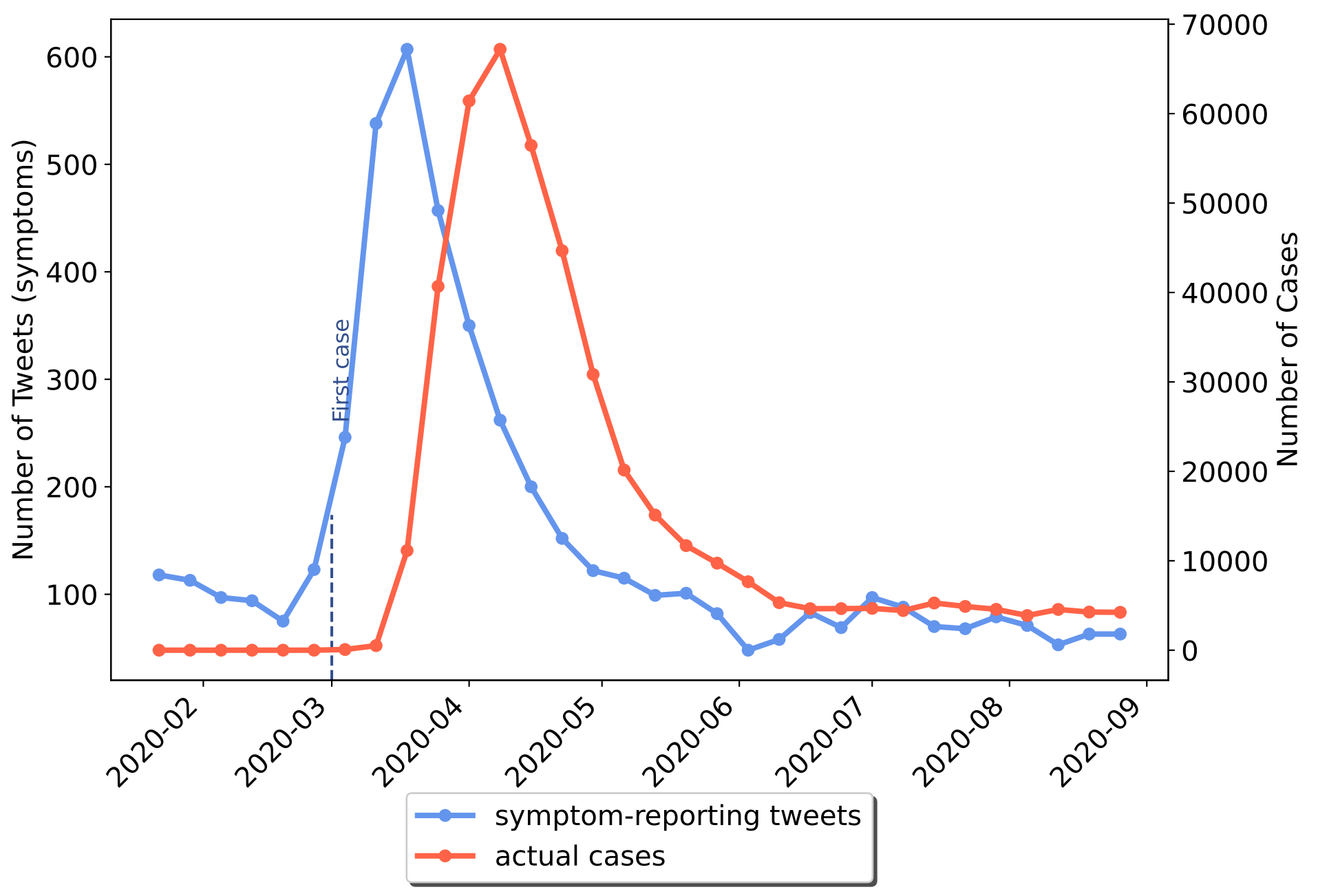

Supplement: Supplementary file 2 [file Data_Sheet_1.ZIP › figures/New_York_symptom_twitter-eps-converted-to.pdf]

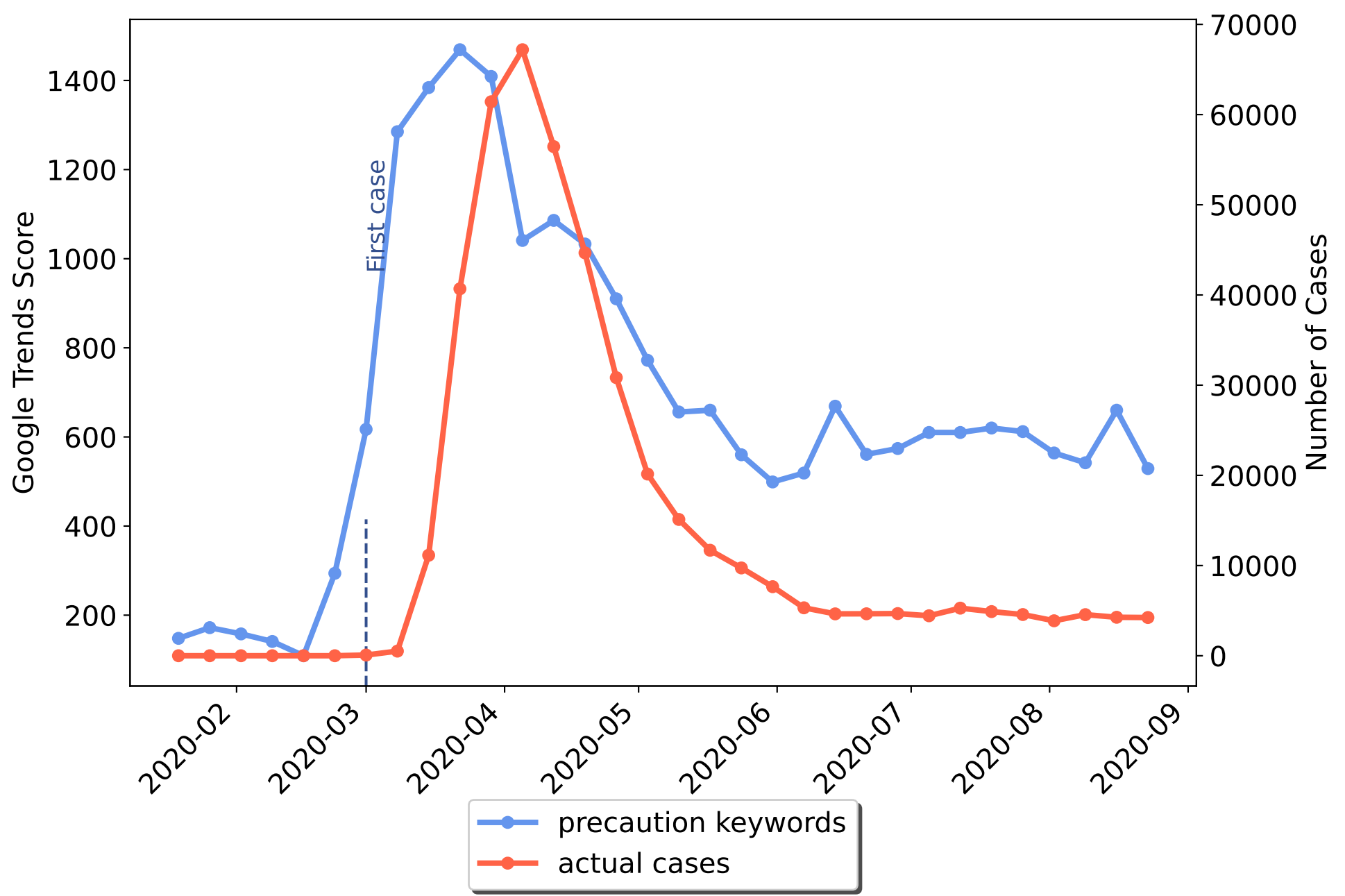

Supplement: Supplementary file 2 [file Data_Sheet_1.ZIP › figures/New_York_totalprecaution_GT-eps-converted-to.pdf]

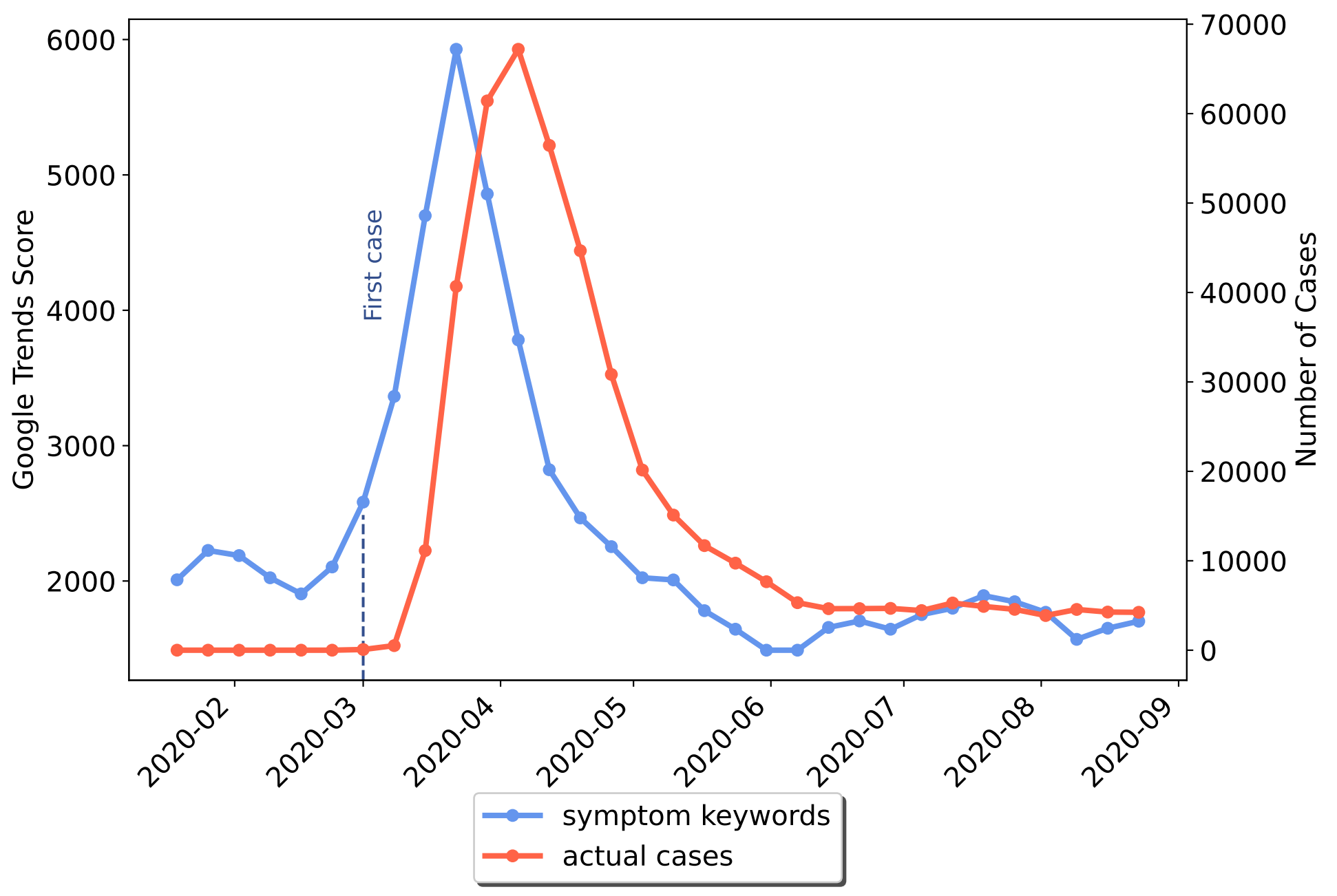

Supplement: Supplementary file 2 [file Data_Sheet_1.ZIP › figures/New_York_totalsymptom_GT-eps-converted-to.pdf]

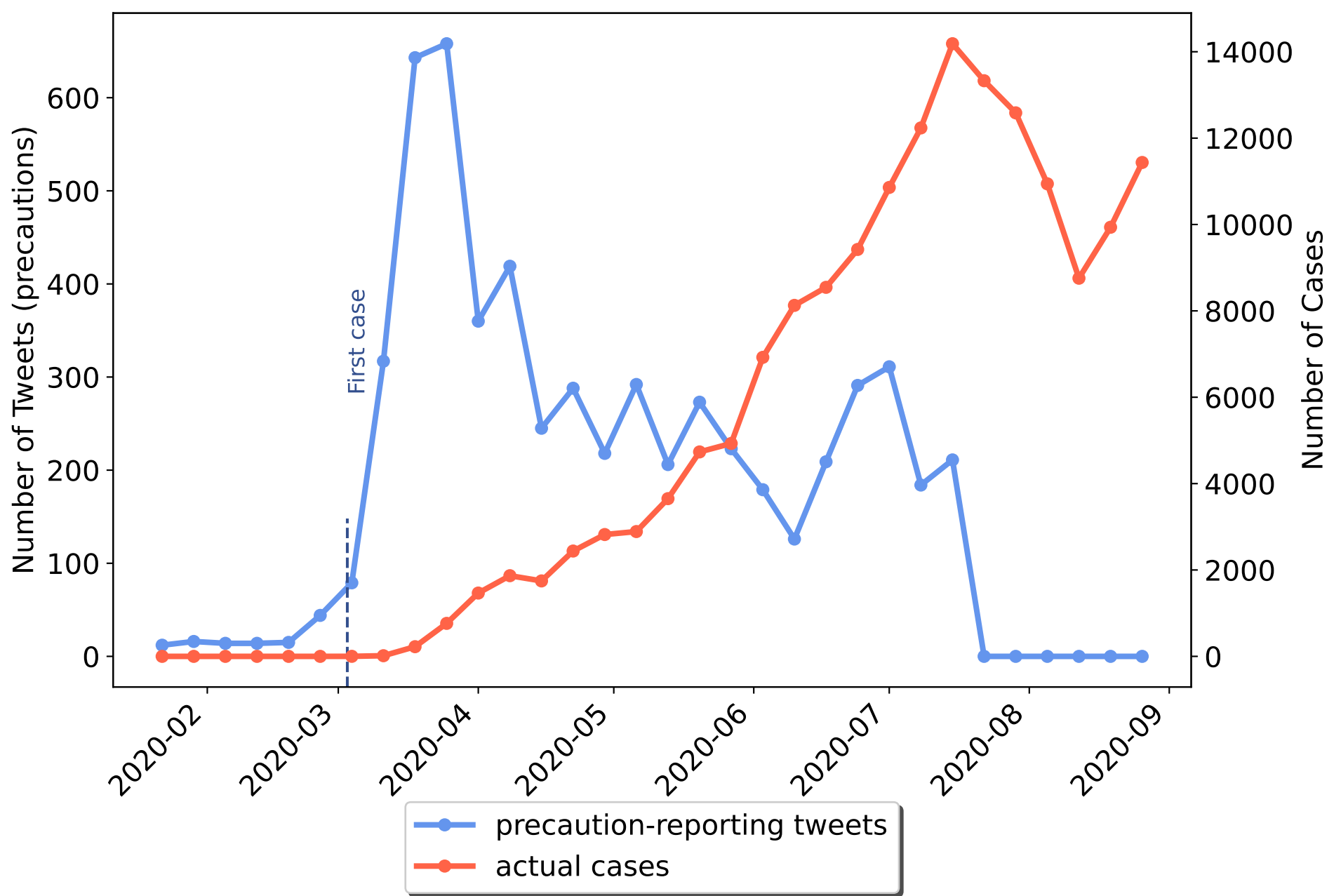

Supplement: Supplementary file 2 [file Data_Sheet_1.ZIP › figures/North_Carolina_precaution_twitter-eps-converted-to.pdf]

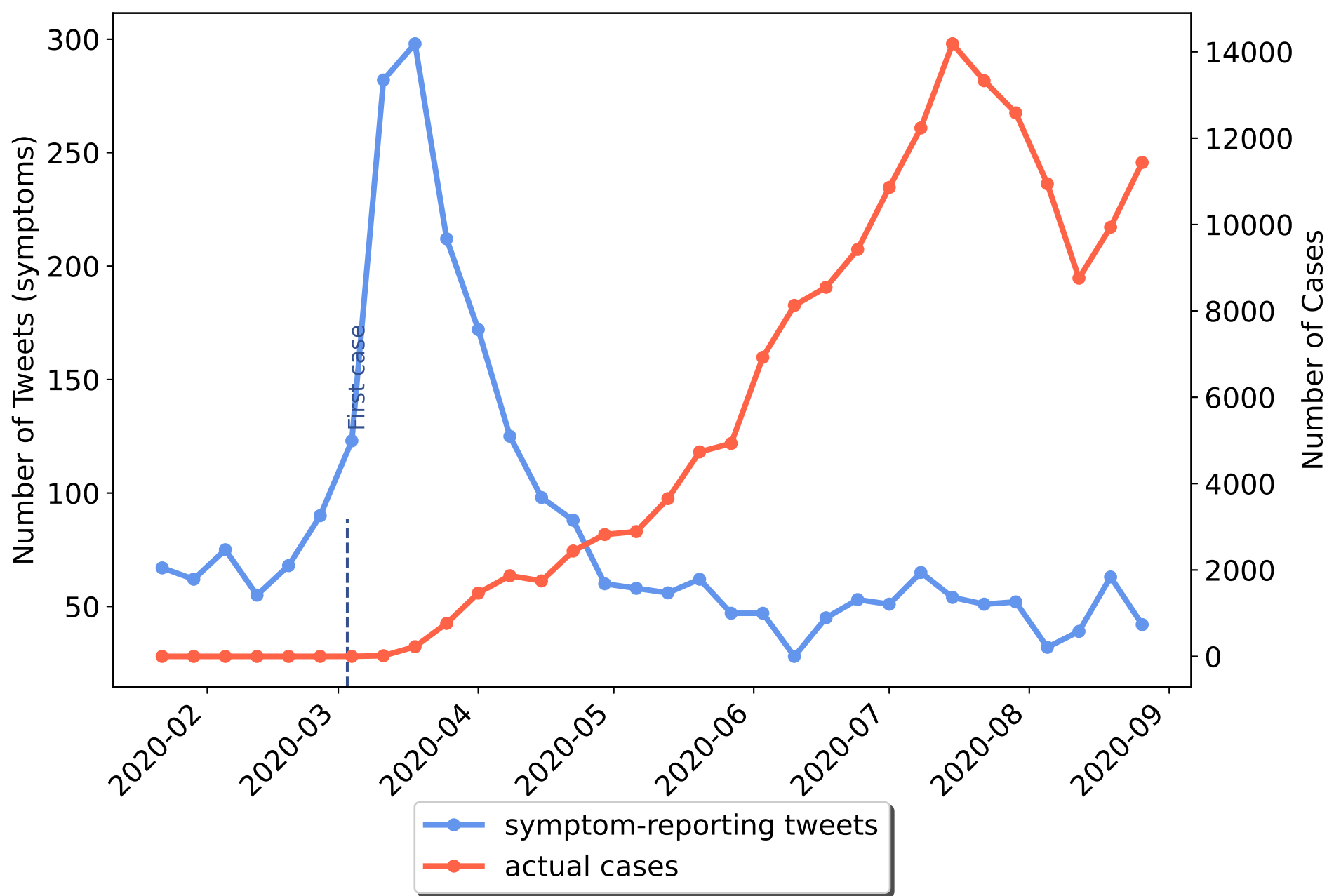

Supplement: Supplementary file 2 [file Data_Sheet_1.ZIP › figures/North_Carolina_symptom_twitter-eps-converted-to.pdf]

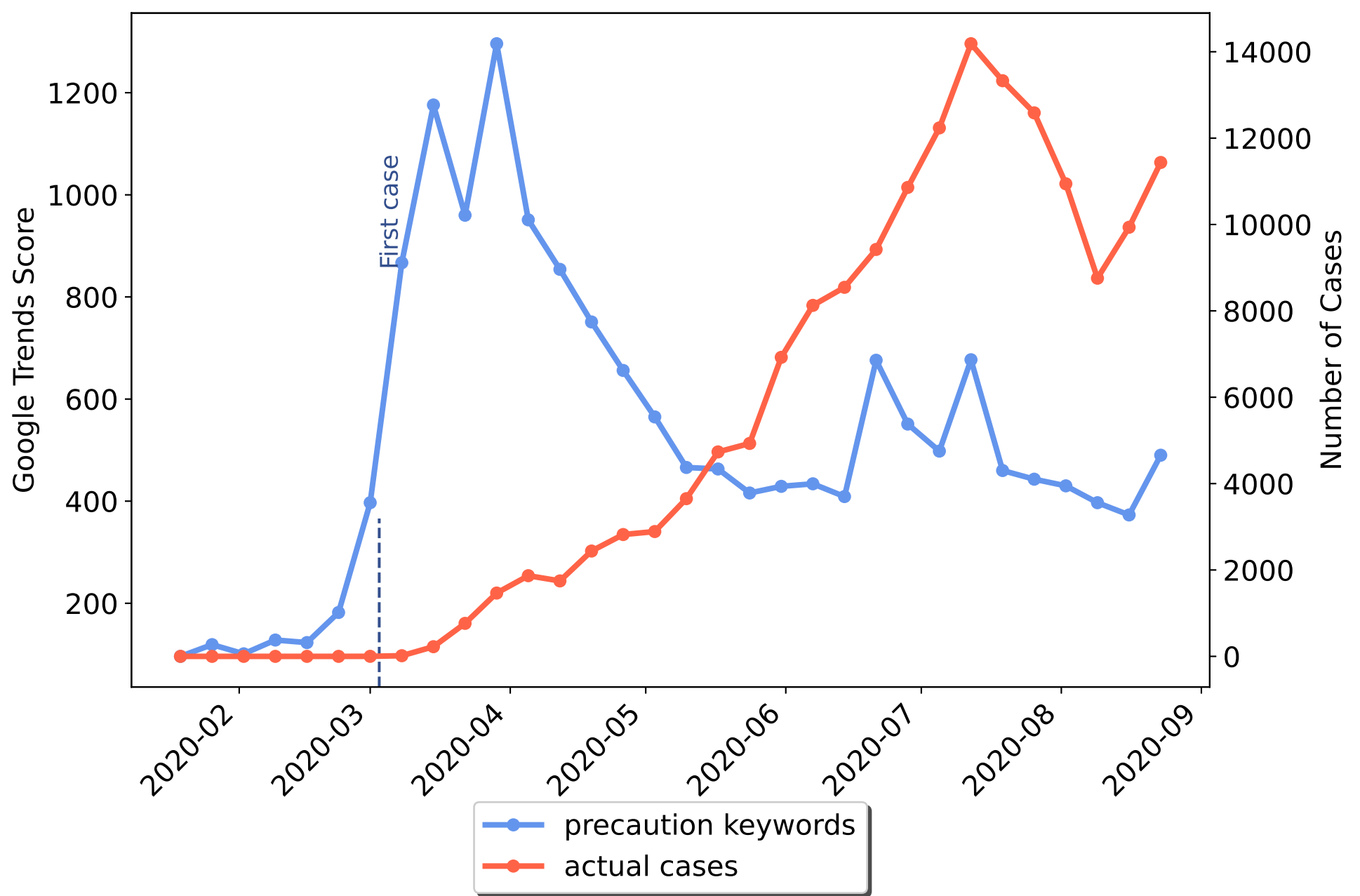

Supplement: Supplementary file 2 [file Data_Sheet_1.ZIP › figures/North_Carolina_totalprecaution_GT-eps-converted-to.pdf]

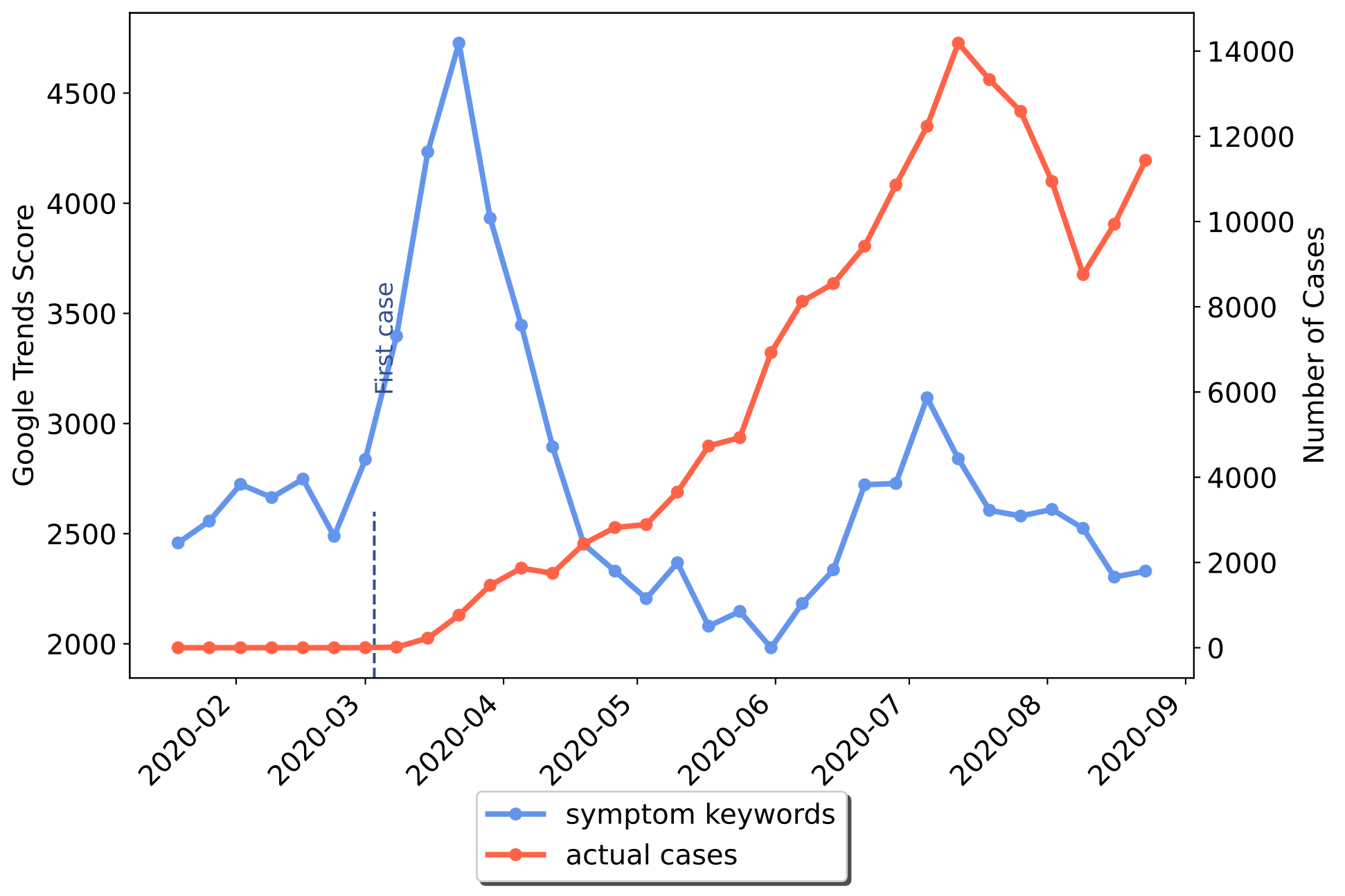

Supplement: Supplementary file 2 [file Data_Sheet_1.ZIP › figures/North_Carolina_totalsymptom_GT-eps-converted-to.pdf]

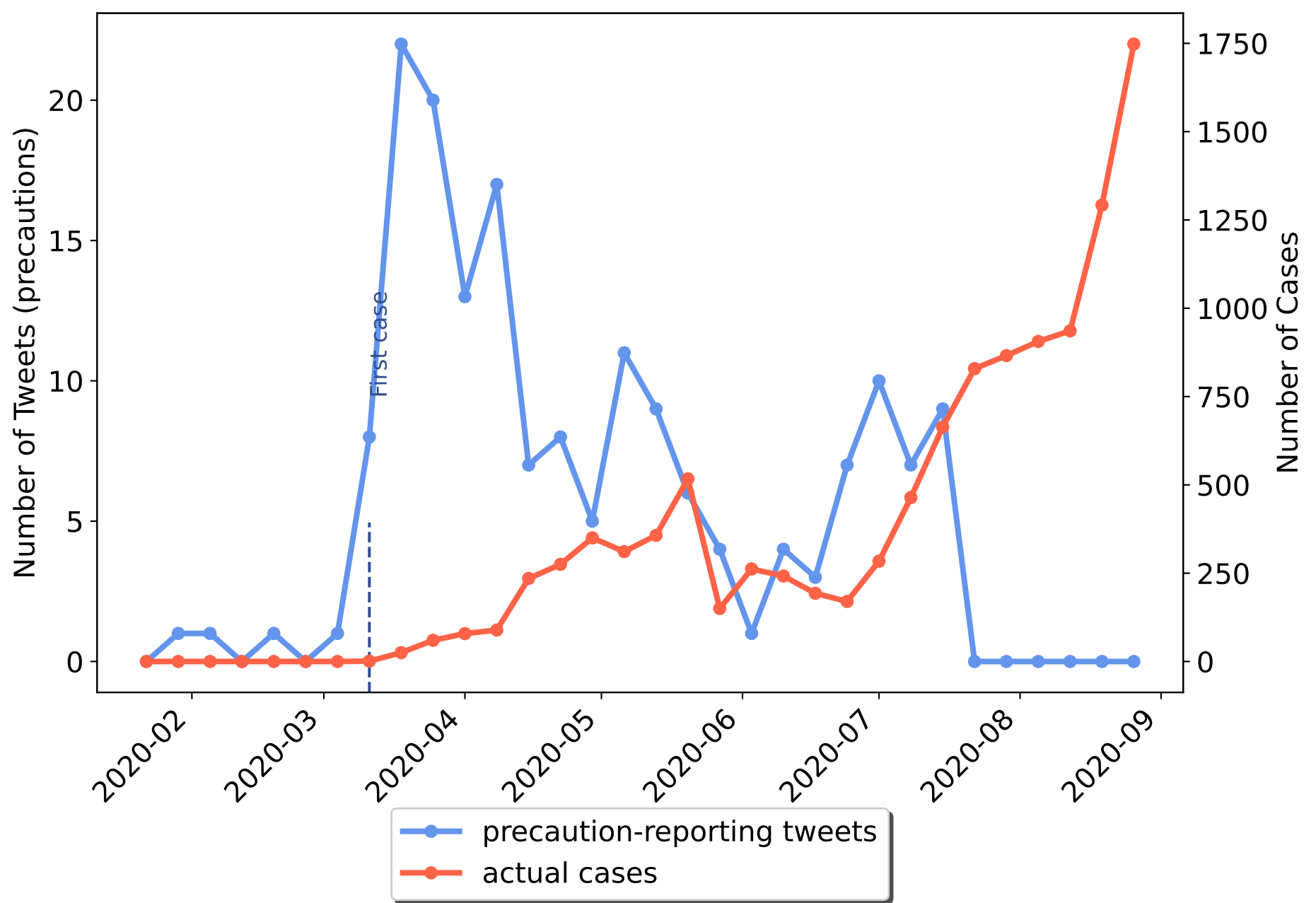

Supplement: Supplementary file 2 [file Data_Sheet_1.ZIP › figures/North_Dakota_precaution_twitter-eps-converted-to.pdf]

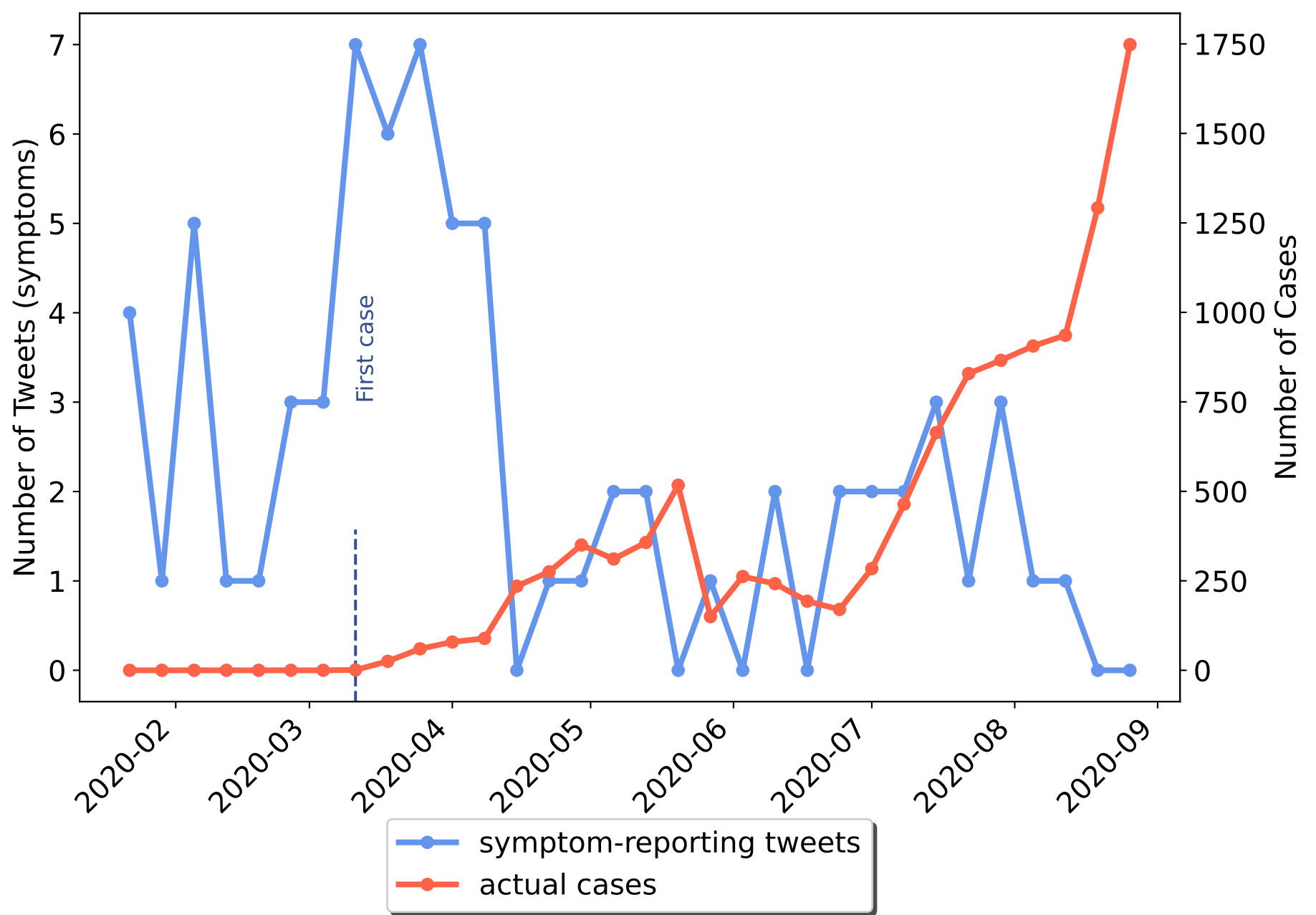

Supplement: Supplementary file 2 [file Data_Sheet_1.ZIP › figures/North_Dakota_symptom_twitter-eps-converted-to.pdf]

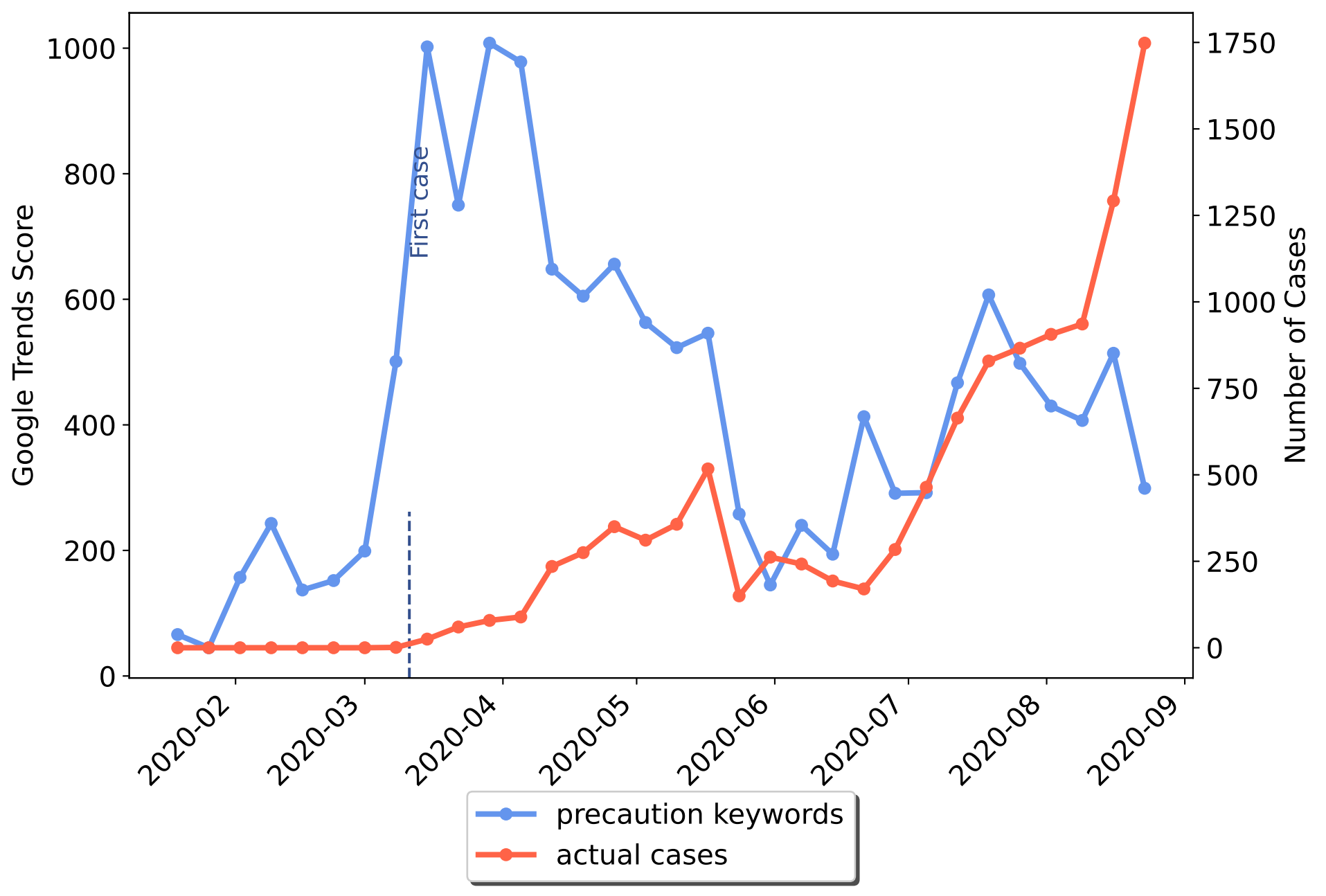

Supplement: Supplementary file 2 [file Data_Sheet_1.ZIP › figures/North_Dakota_totalprecaution_GT-eps-converted-to.pdf]

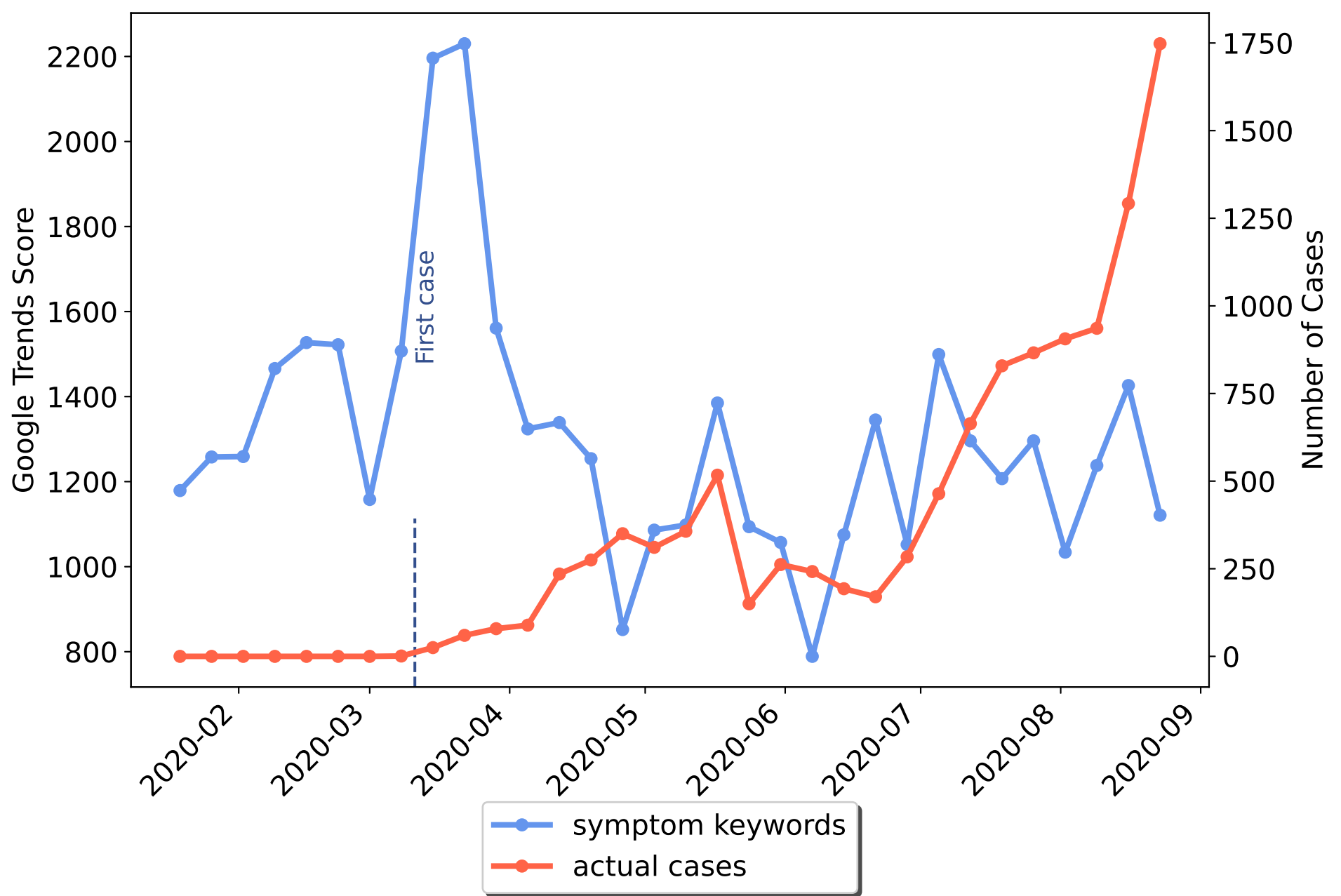

Supplement: Supplementary file 2 [file Data_Sheet_1.ZIP › figures/North_Dakota_totalsymptom_GT-eps-converted-to.pdf]

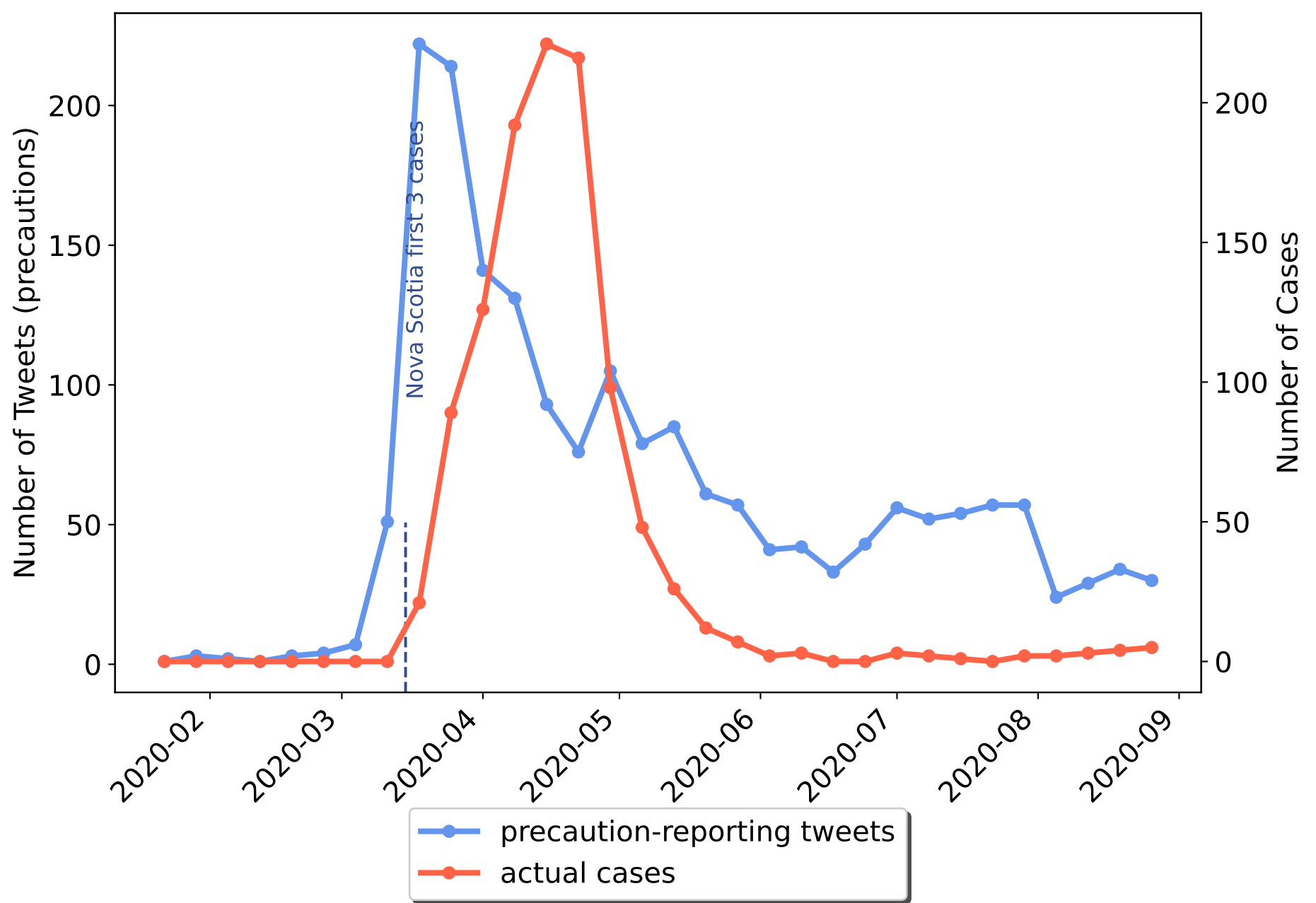

Supplement: Supplementary file 2 [file Data_Sheet_1.ZIP › figures/Nova_Scotia_precaution_twitter-eps-converted-to.pdf]

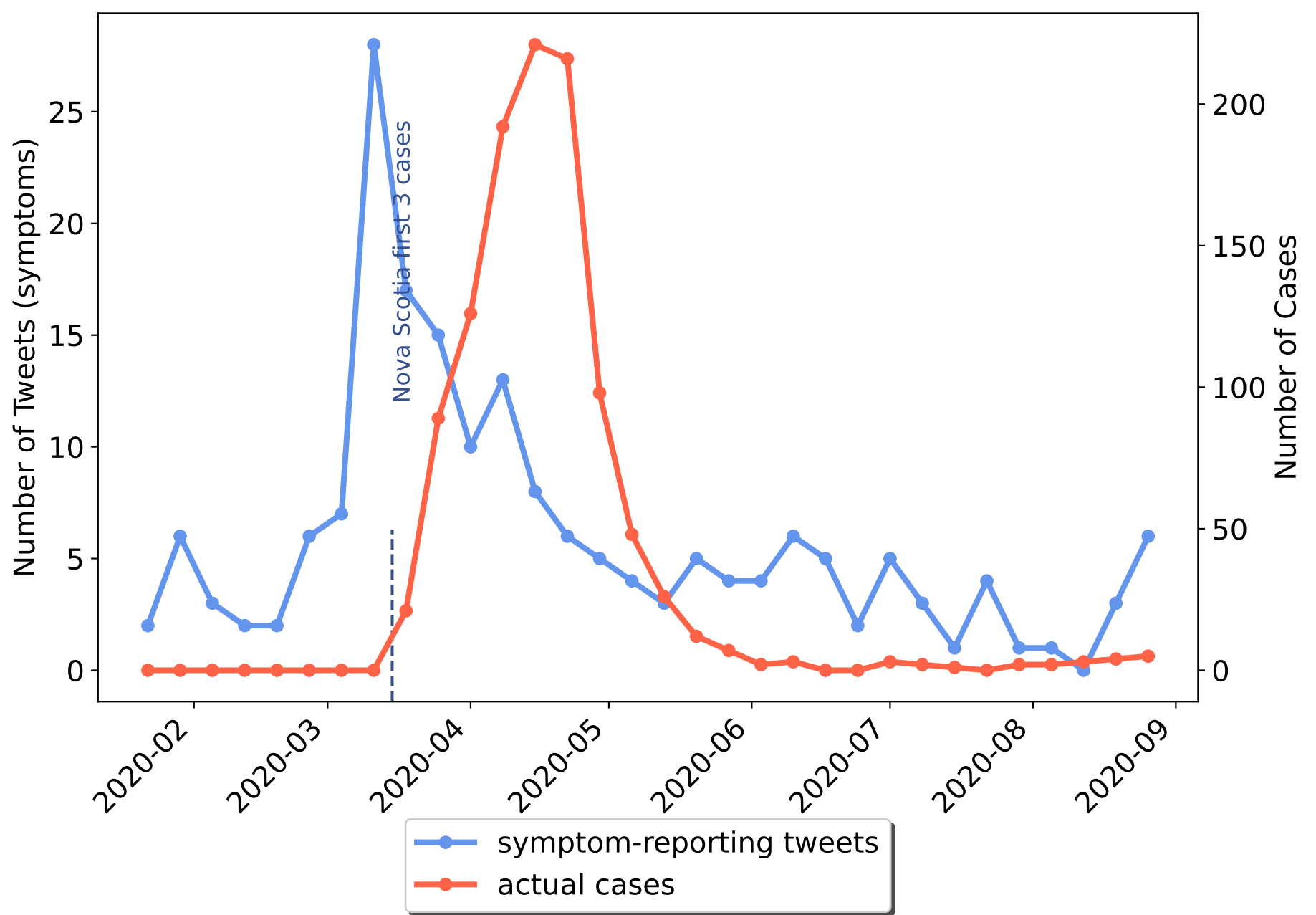

Supplement: Supplementary file 2 [file Data_Sheet_1.ZIP › figures/Nova_Scotia_symptom_twitter-eps-converted-to.pdf]

Google Trends Score

1000  
800  
600  
400  
200

Number of Cases

200  
150  
100  
50  
0

2020-02 2020-03 2020-04 2020-05 2020-06 2020-07 2020-08 2020-09

Nova Scotia first 3 cases

precaution keywords  
actual cases

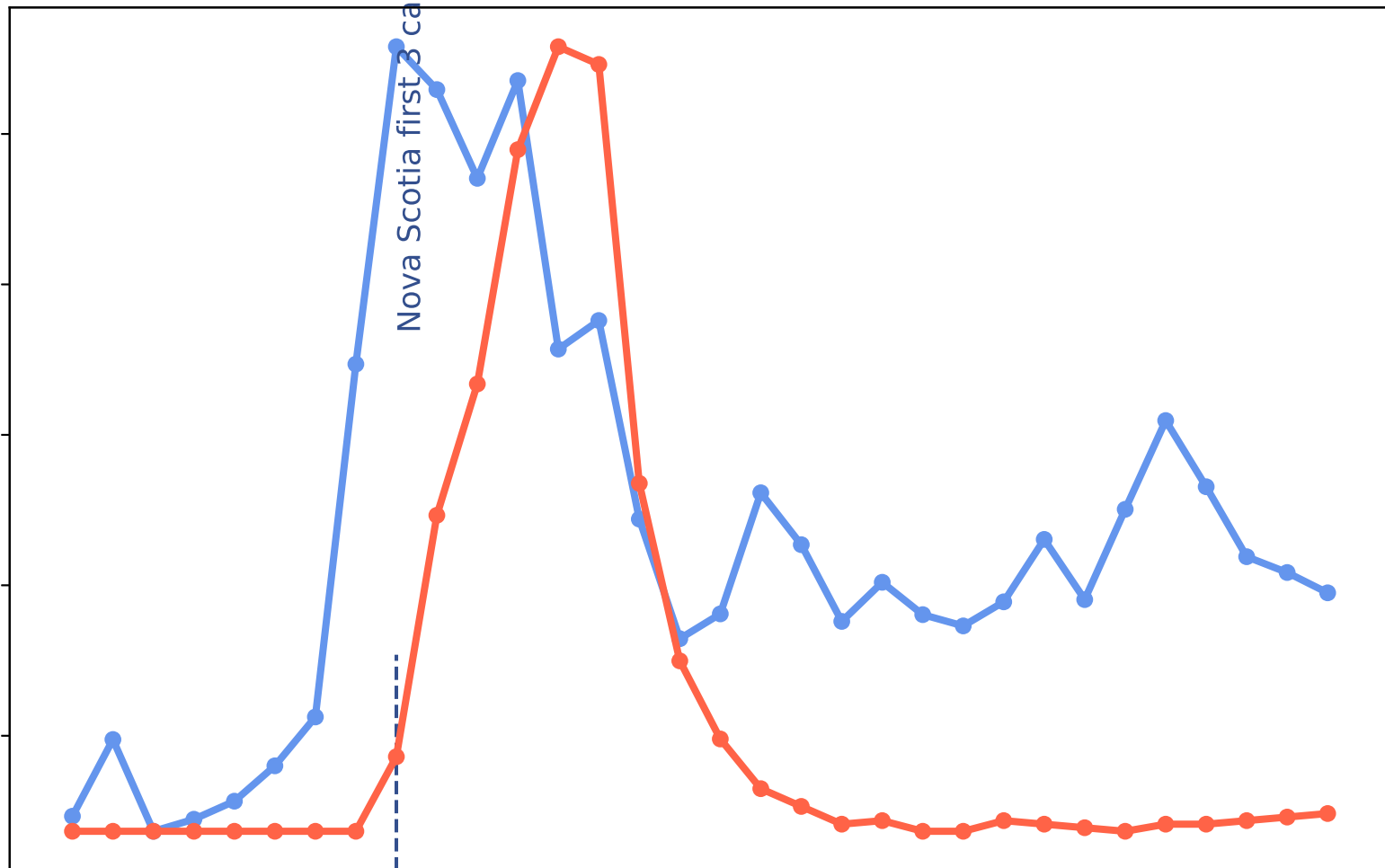

Supplement: Supplementary file 2 [file Data_Sheet_1.ZIP › figures/Nova_Scotia_totalprecaution_GT-eps-converted-to.pdf]

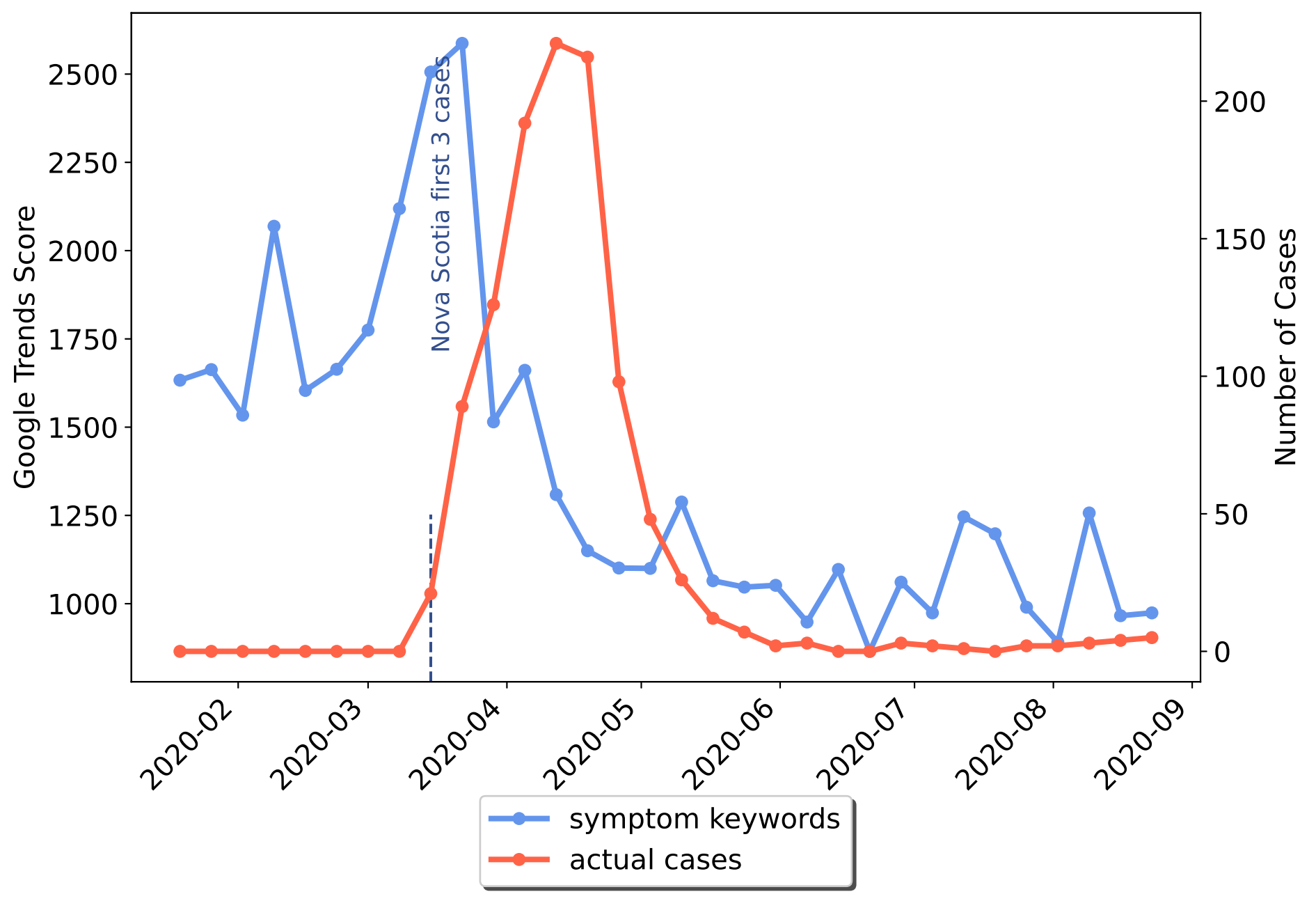

Supplement: Supplementary file 2 [file Data_Sheet_1.ZIP › figures/Nova_Scotia_totalsymptom_GT-eps-converted-to.pdf]

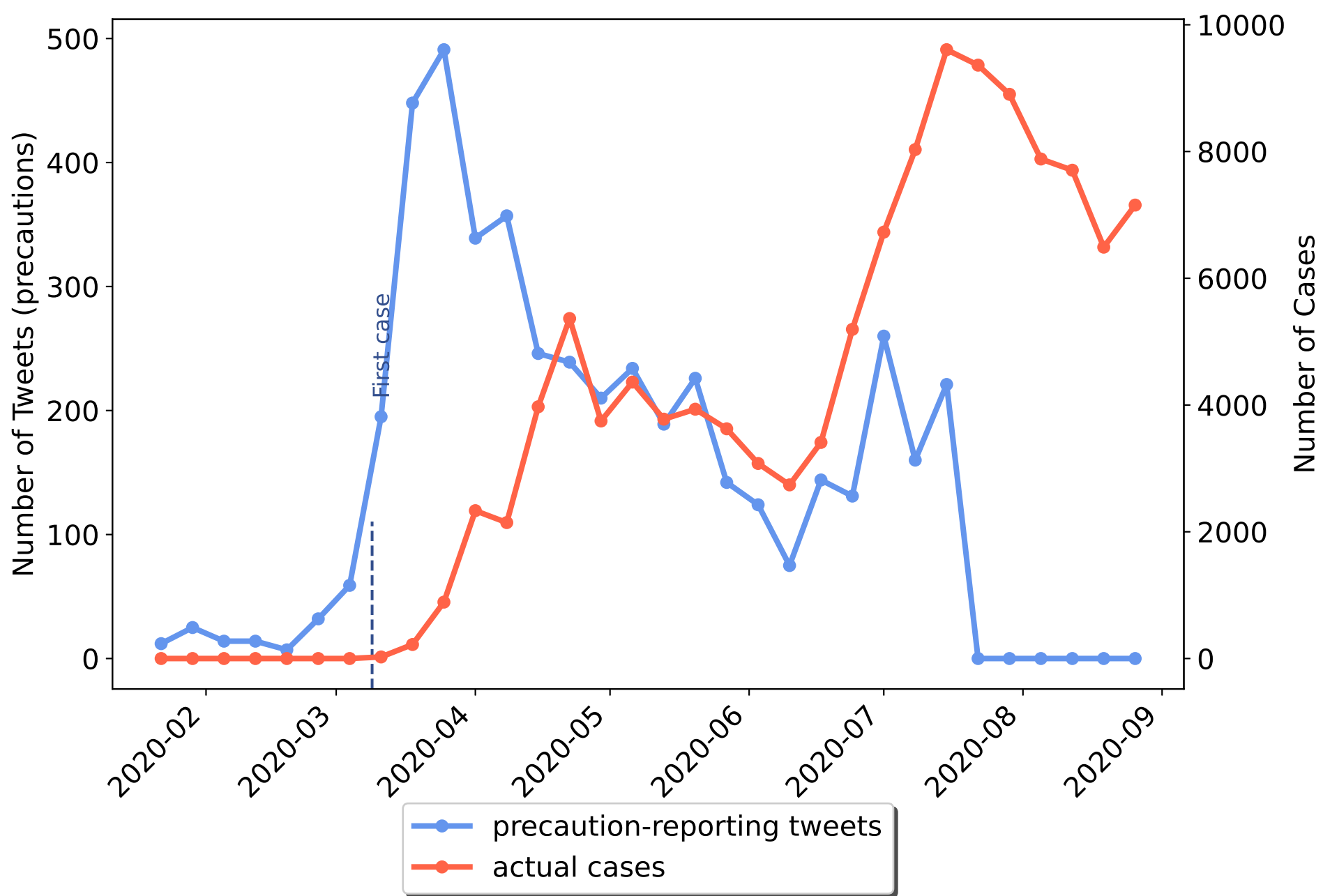

Supplement: Supplementary file 2 [file Data_Sheet_1.ZIP › figures/Ohio_precaution_twitter-eps-converted-to.pdf]

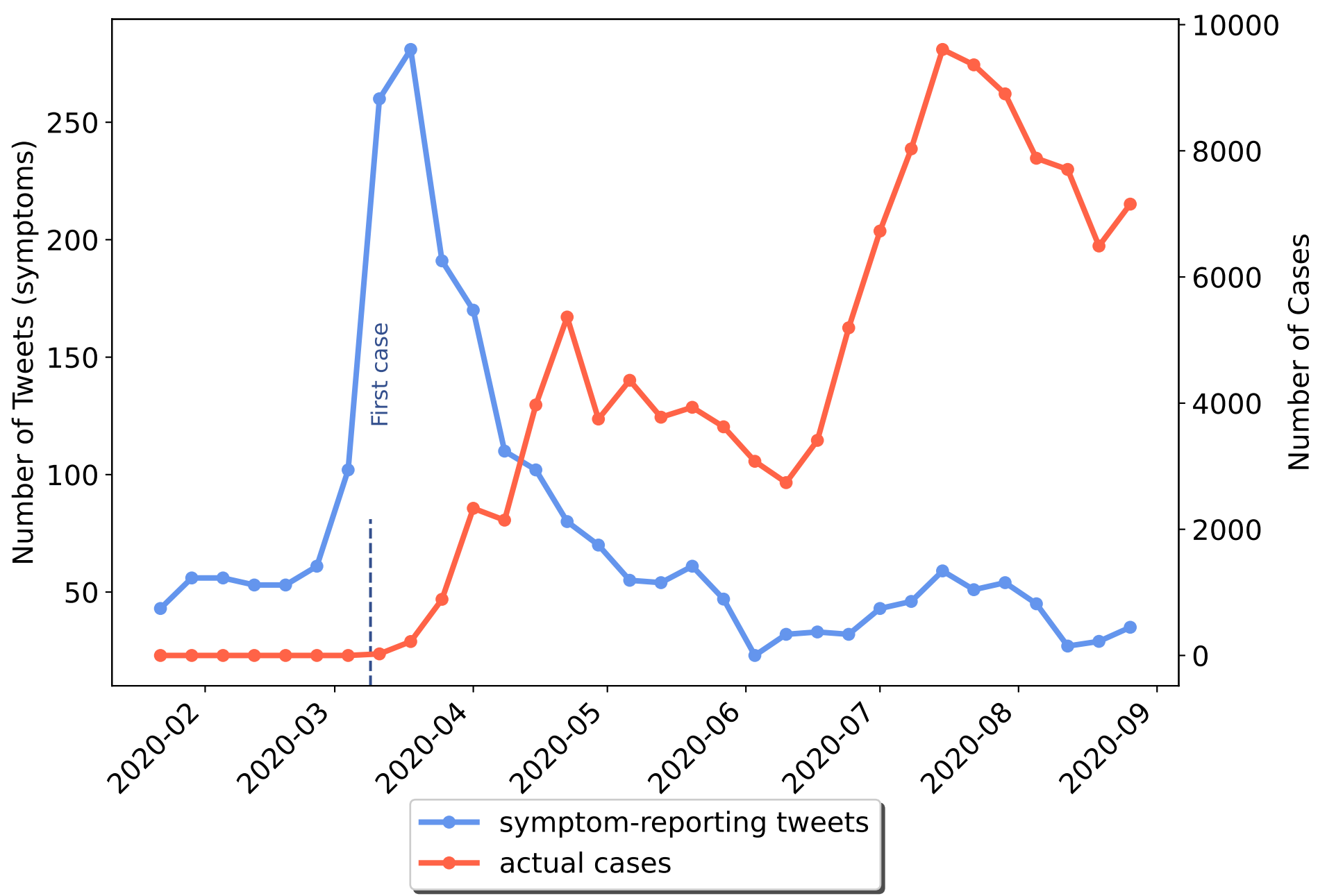

Supplement: Supplementary file 2 [file Data_Sheet_1.ZIP › figures/Ohio_symptom_twitter-eps-converted-to.pdf]

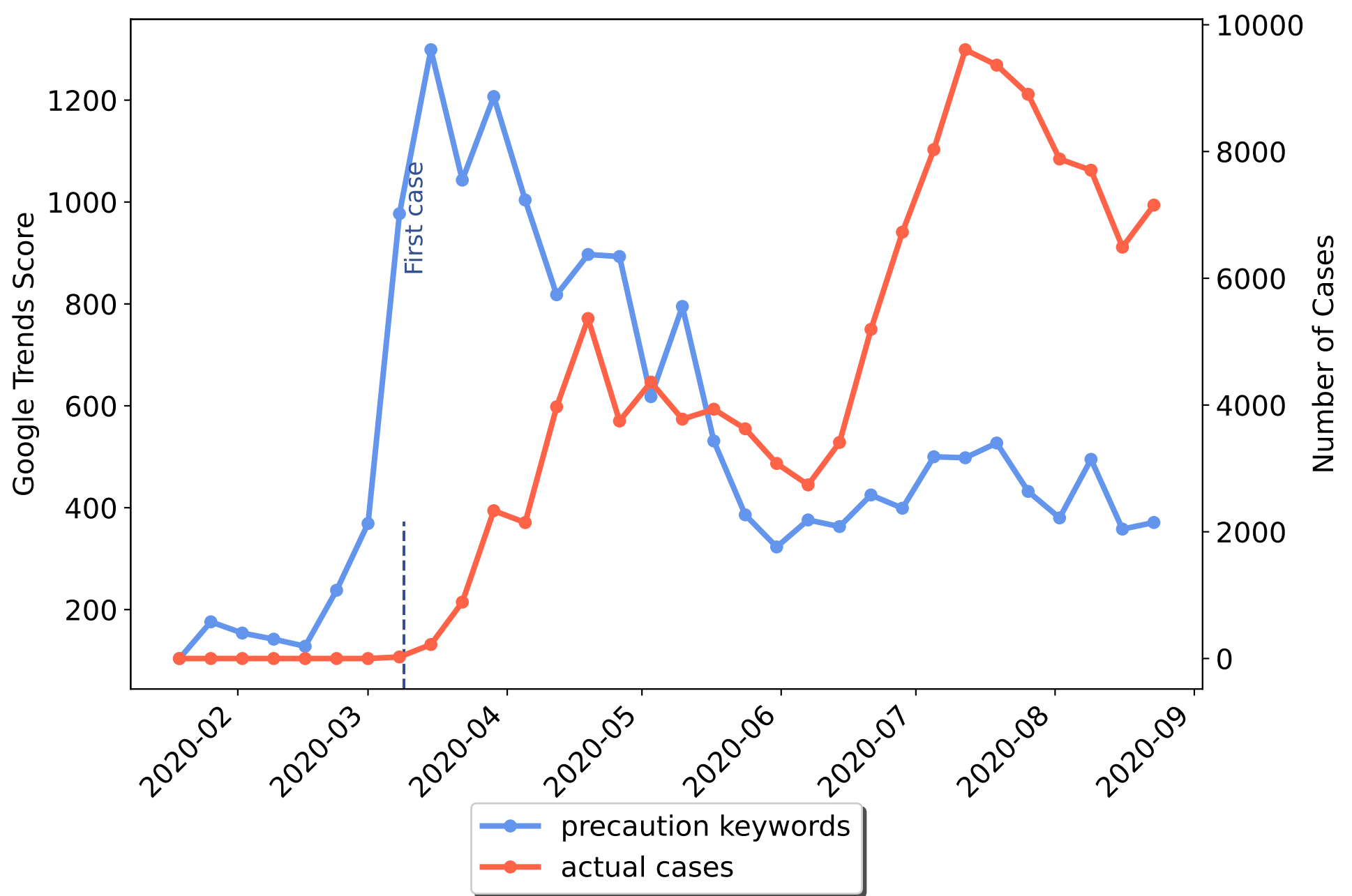

Supplement: Supplementary file 2 [file Data_Sheet_1.ZIP › figures/Ohio_totalprecaution_GT-eps-converted-to.pdf]

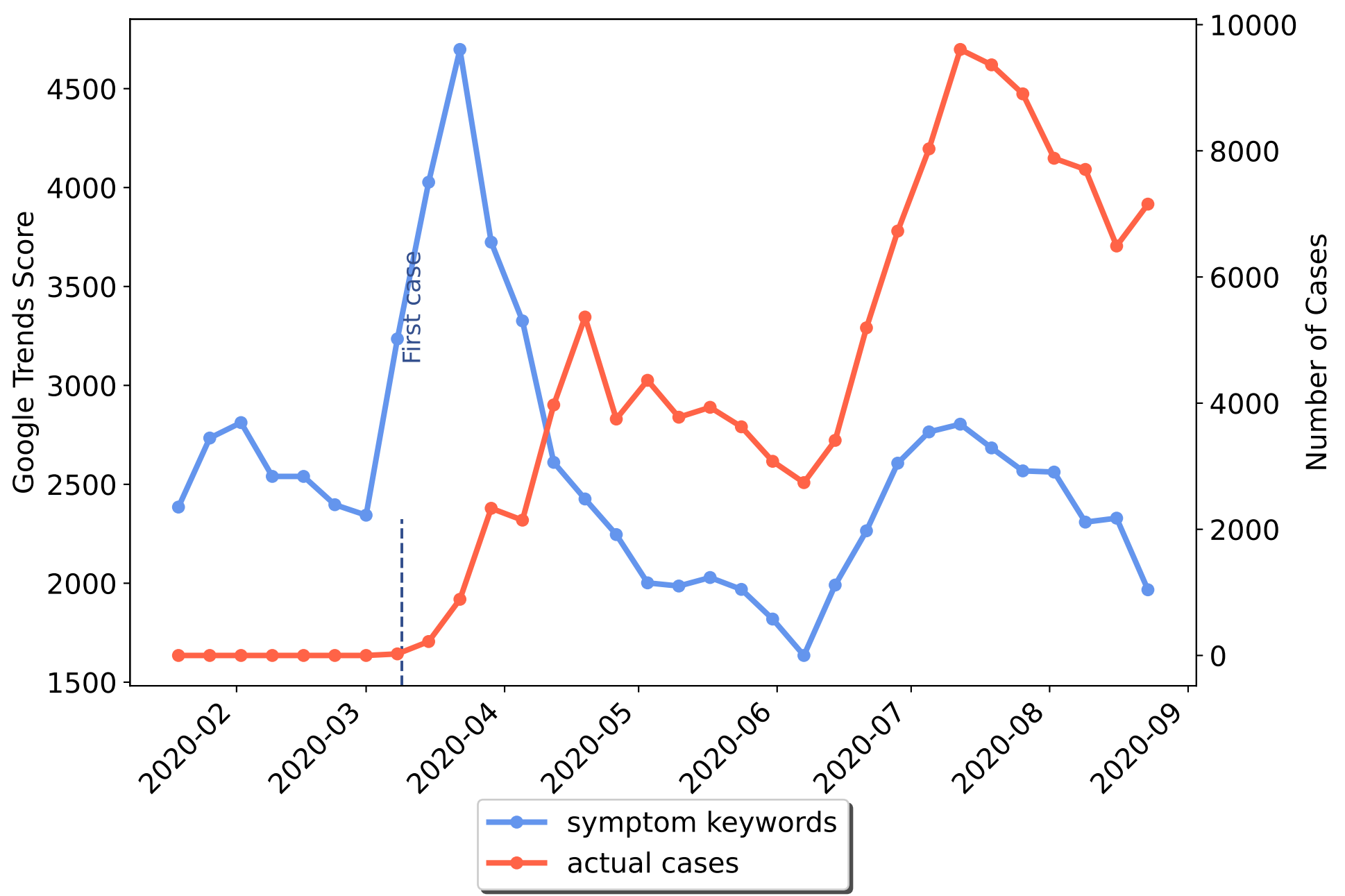

Supplement: Supplementary file 2 [file Data_Sheet_1.ZIP › figures/Ohio_totalsymptom_GT-eps-converted-to.pdf]

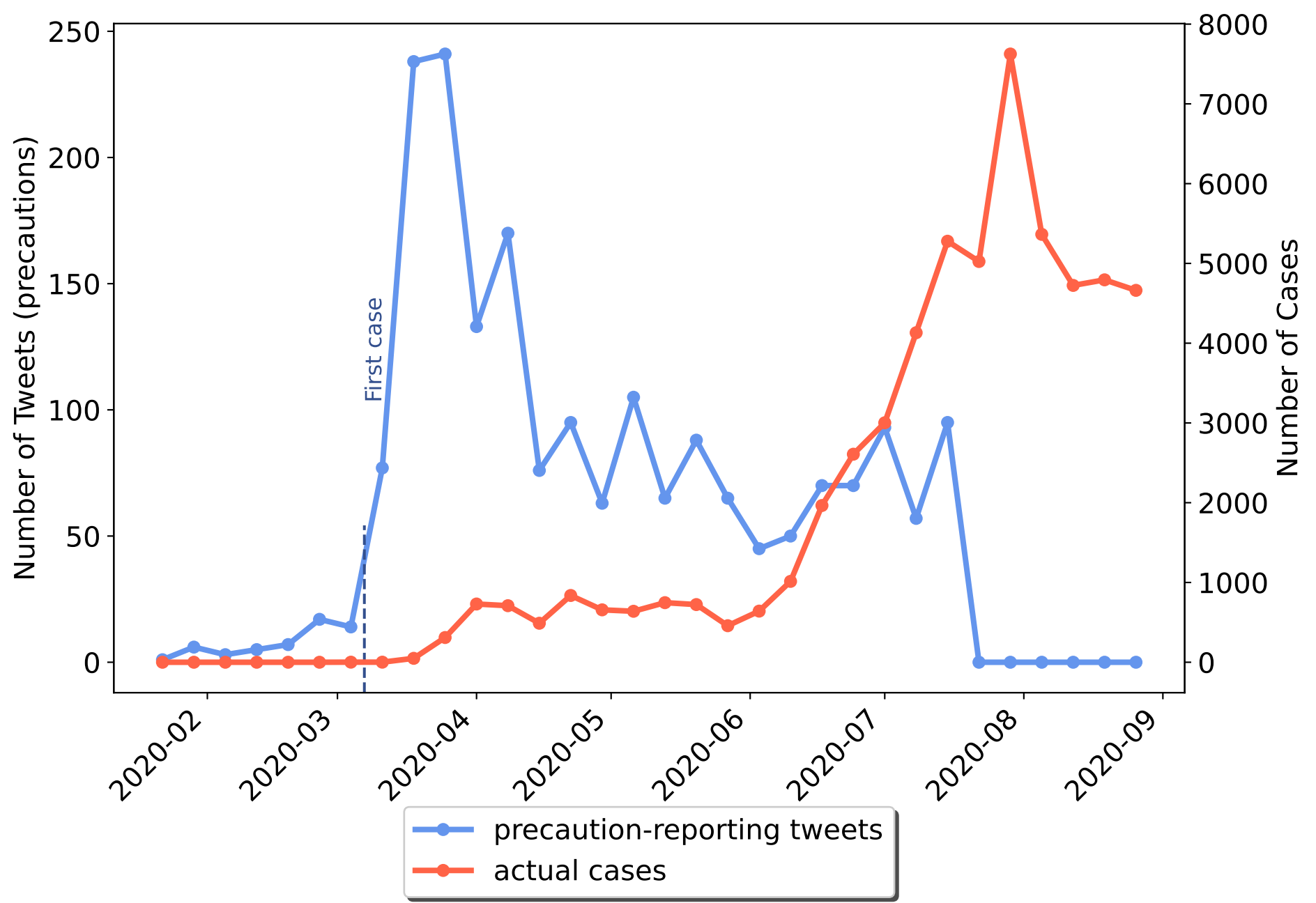

Supplement: Supplementary file 2 [file Data_Sheet_1.ZIP › figures/Oklahoma_precaution_twitter-eps-converted-to.pdf]

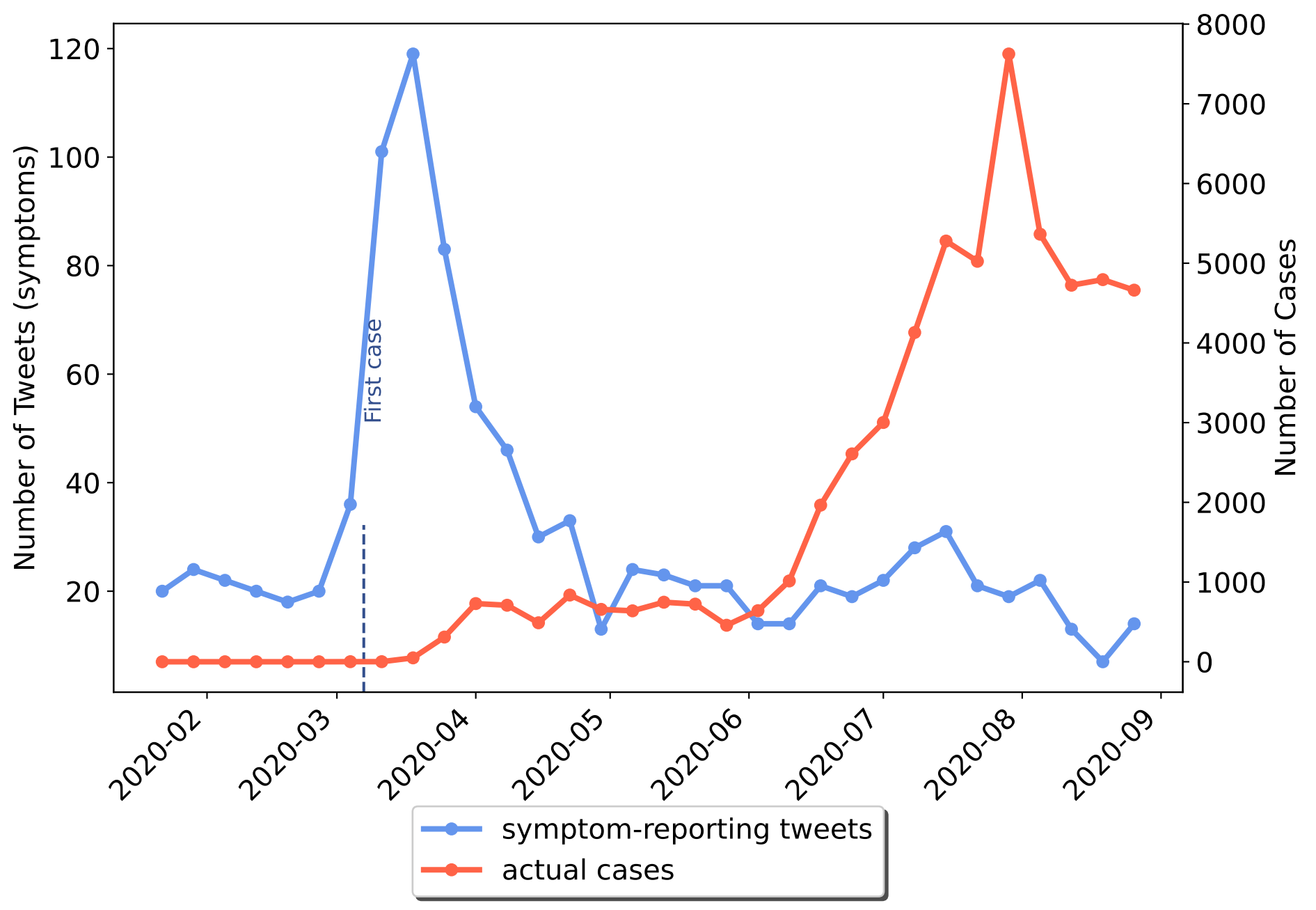

Supplement: Supplementary file 2 [file Data_Sheet_1.ZIP › figures/Oklahoma_symptom_twitter-eps-converted-to.pdf]

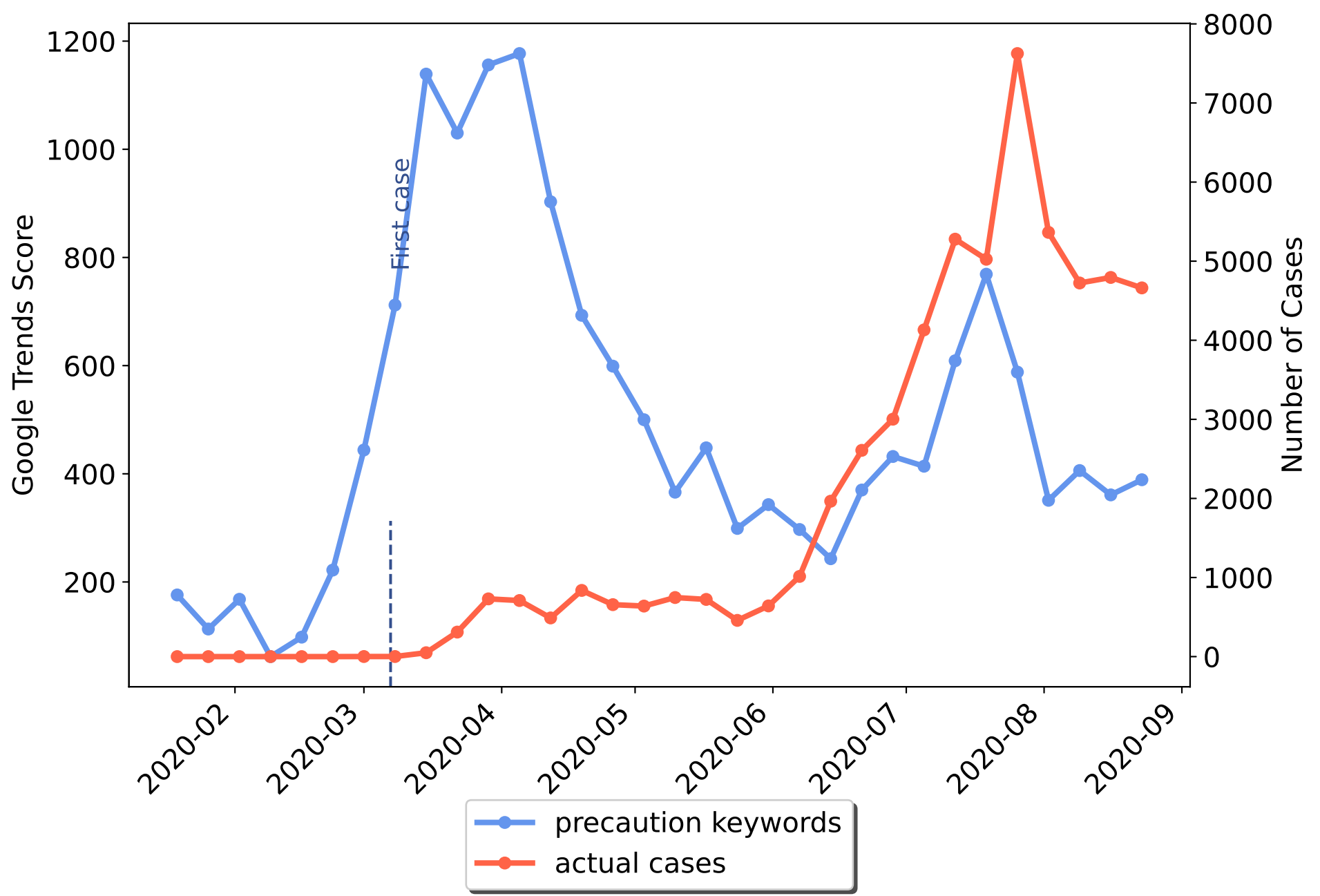

Supplement: Supplementary file 2 [file Data_Sheet_1.ZIP › figures/Oklahoma_totalprecaution_GT-eps-converted-to.pdf]

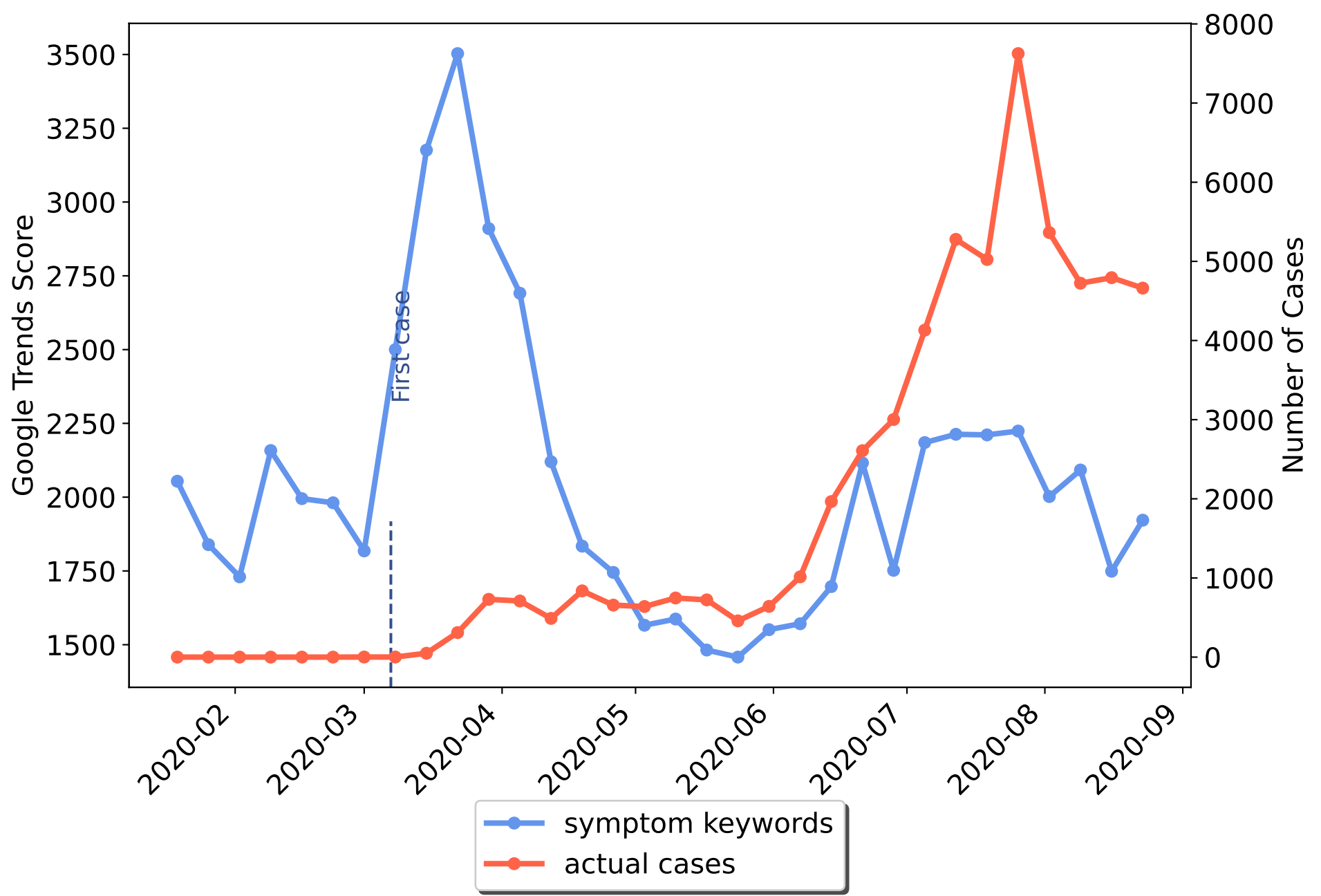

Supplement: Supplementary file 2 [file Data_Sheet_1.ZIP › figures/Oklahoma_totalsymptom_GT-eps-converted-to.pdf]

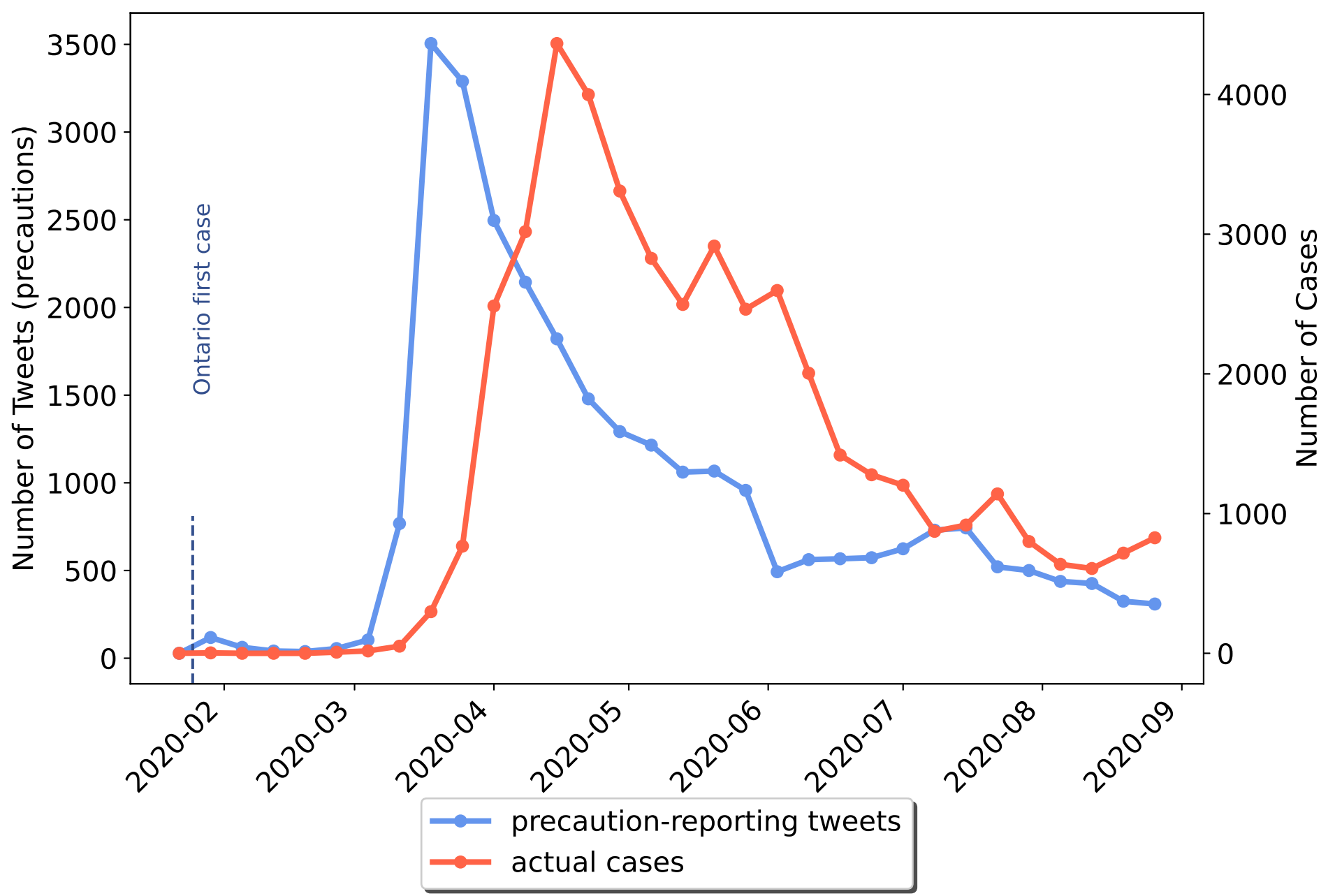

Supplement: Supplementary file 2 [file Data_Sheet_1.ZIP › figures/Ontario_precaution_twitter-eps-converted-to.pdf]

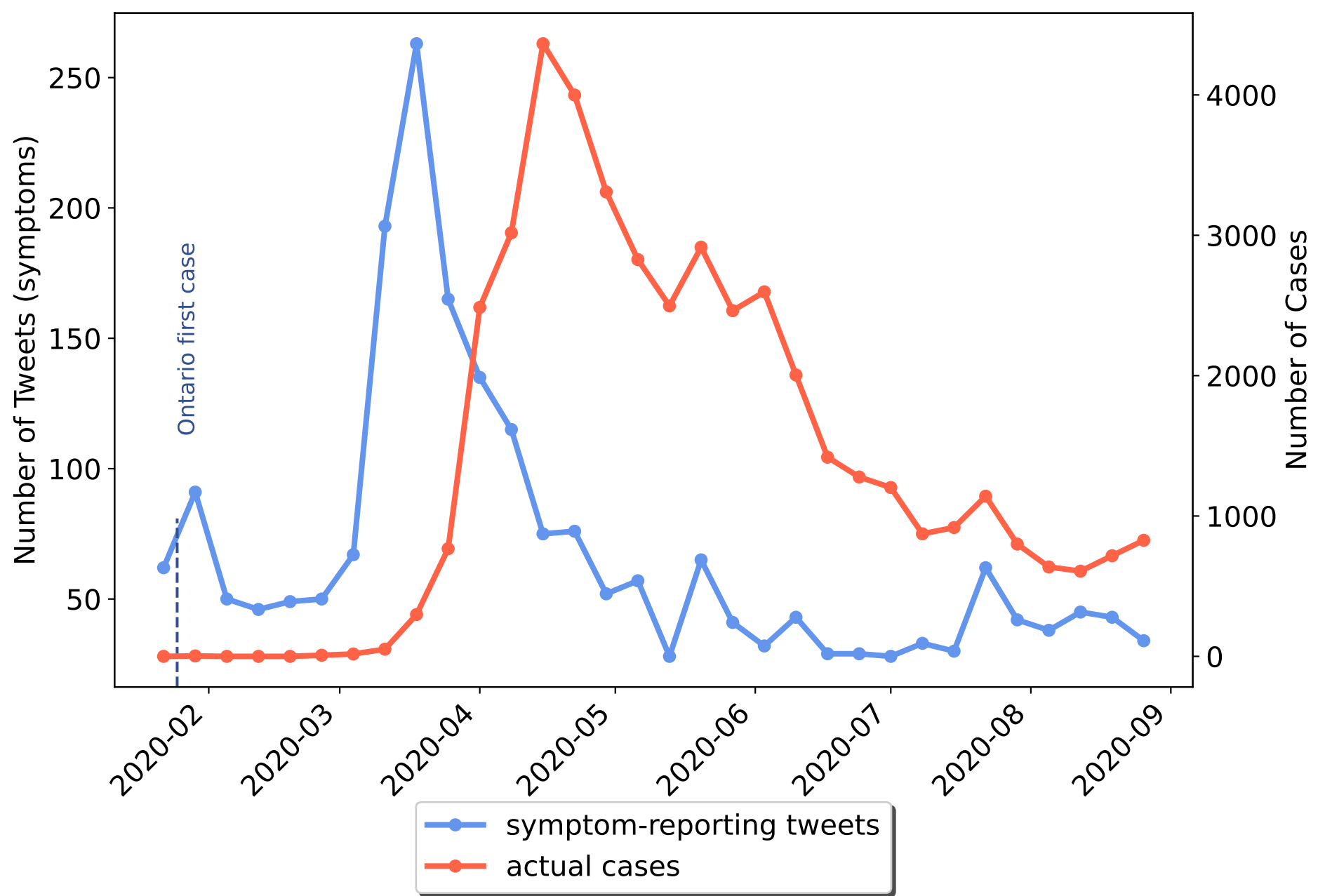

Supplement: Supplementary file 2 [file Data_Sheet_1.ZIP › figures/Ontario_symptom_twitter-eps-converted-to.pdf]

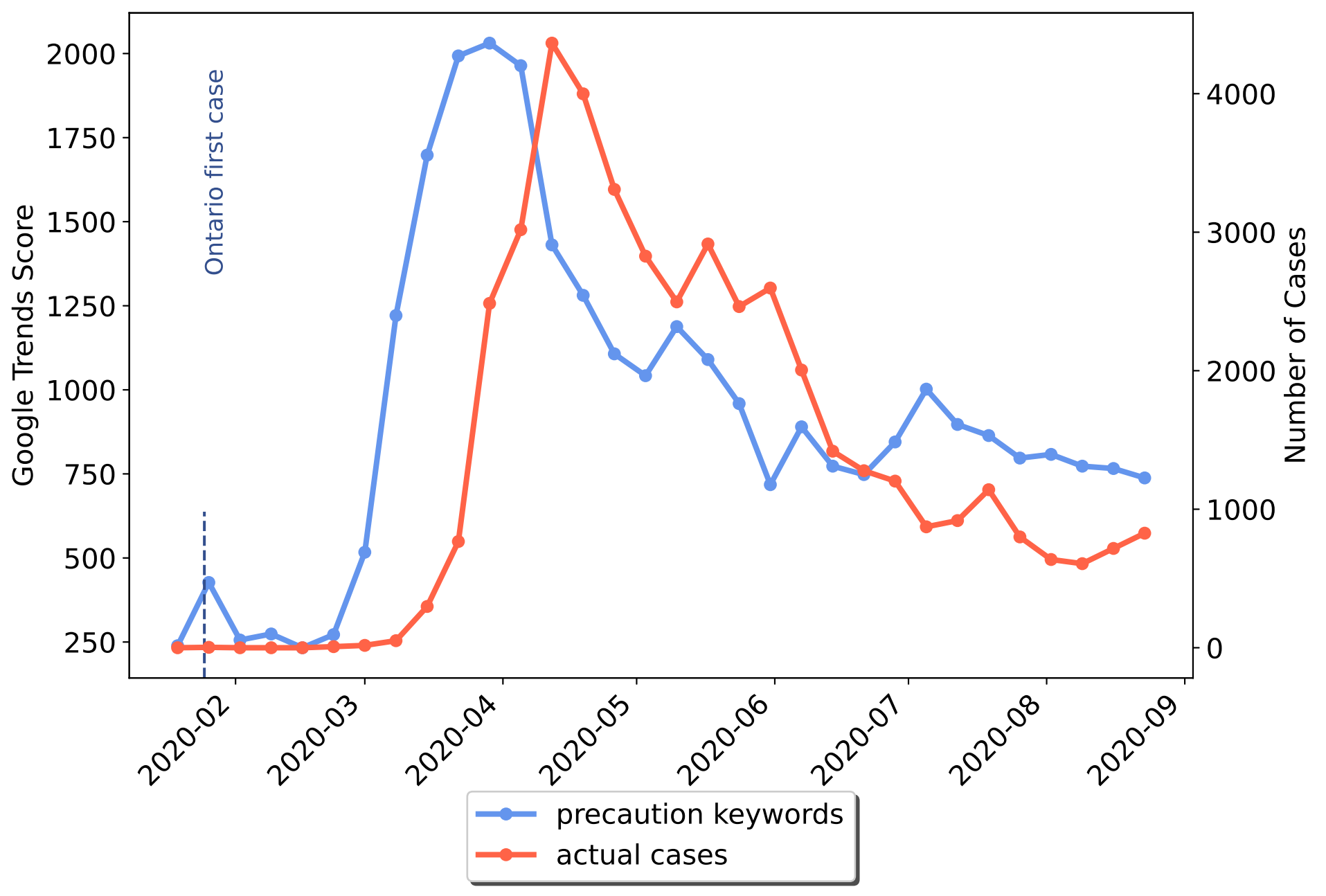

Supplement: Supplementary file 2 [file Data_Sheet_1.ZIP › figures/Ontario_totalprecaution_GT-eps-converted-to.pdf]

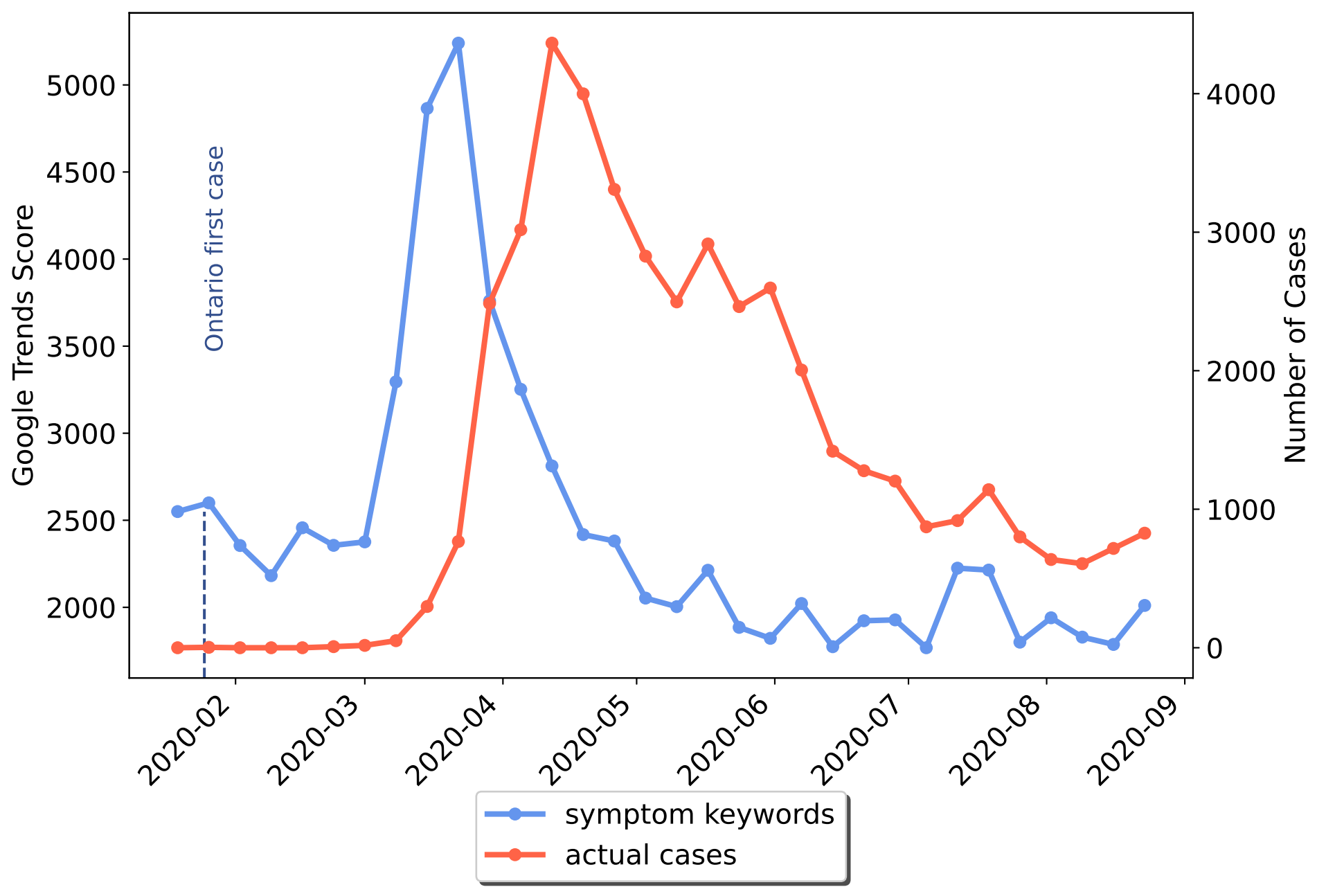

Supplement: Supplementary file 2 [file Data_Sheet_1.ZIP › figures/Ontario_totalsymptom_GT-eps-converted-to.pdf]

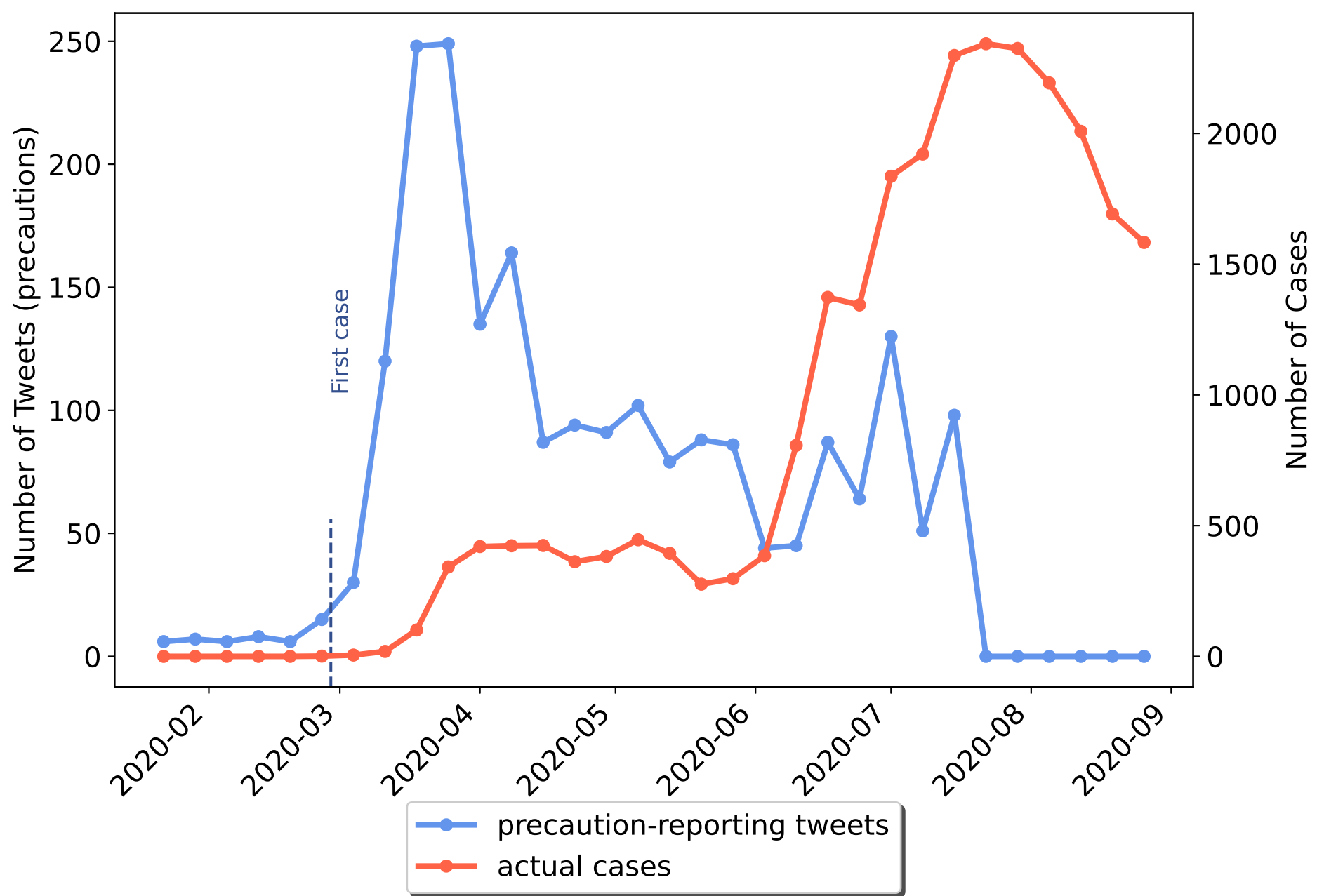

Supplement: Supplementary file 2 [file Data_Sheet_1.ZIP › figures/Oregon_precaution_twitter-eps-converted-to.pdf]

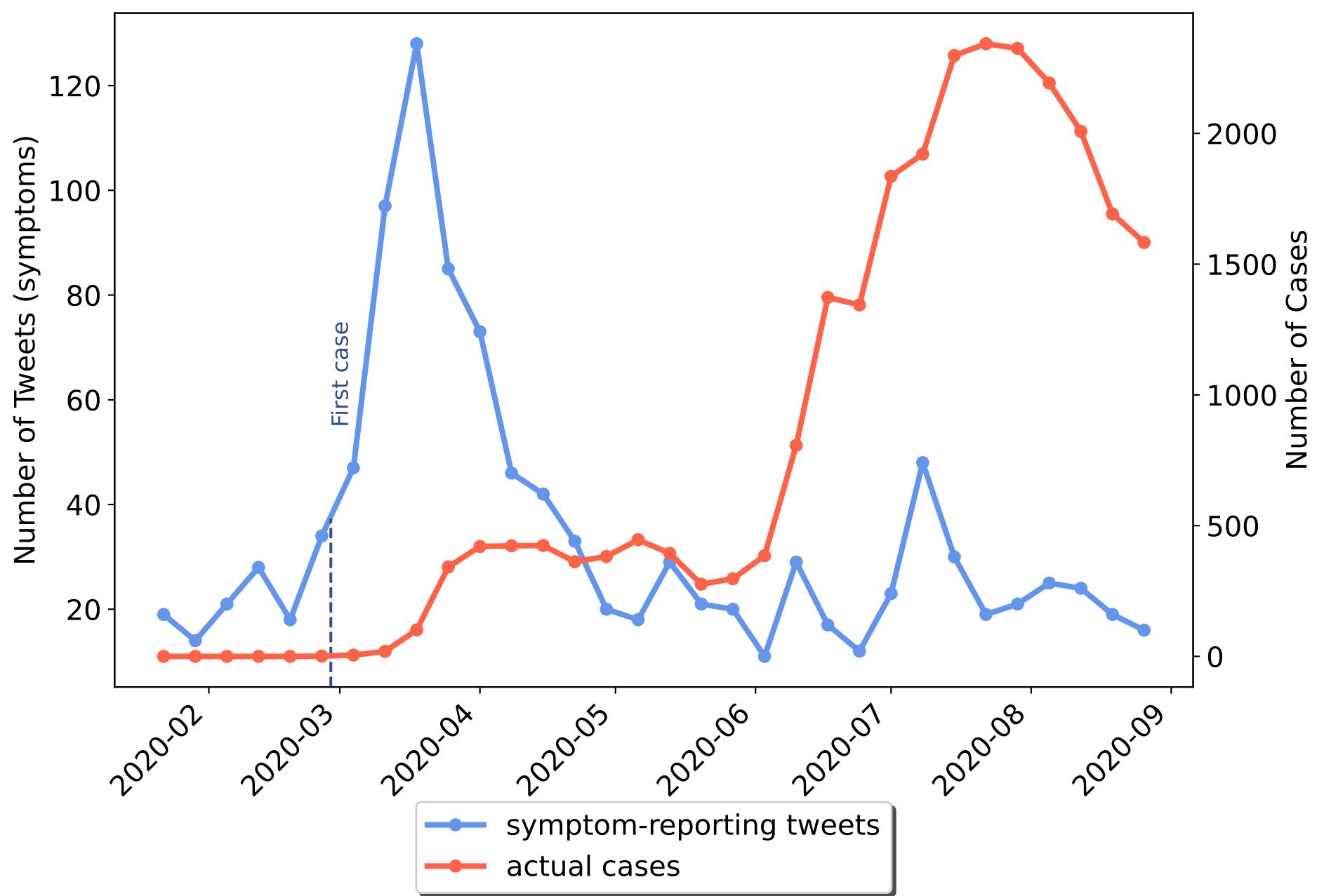

Supplement: Supplementary file 2 [file Data_Sheet_1.ZIP › figures/Oregon_symptom_twitter-eps-converted-to.pdf]

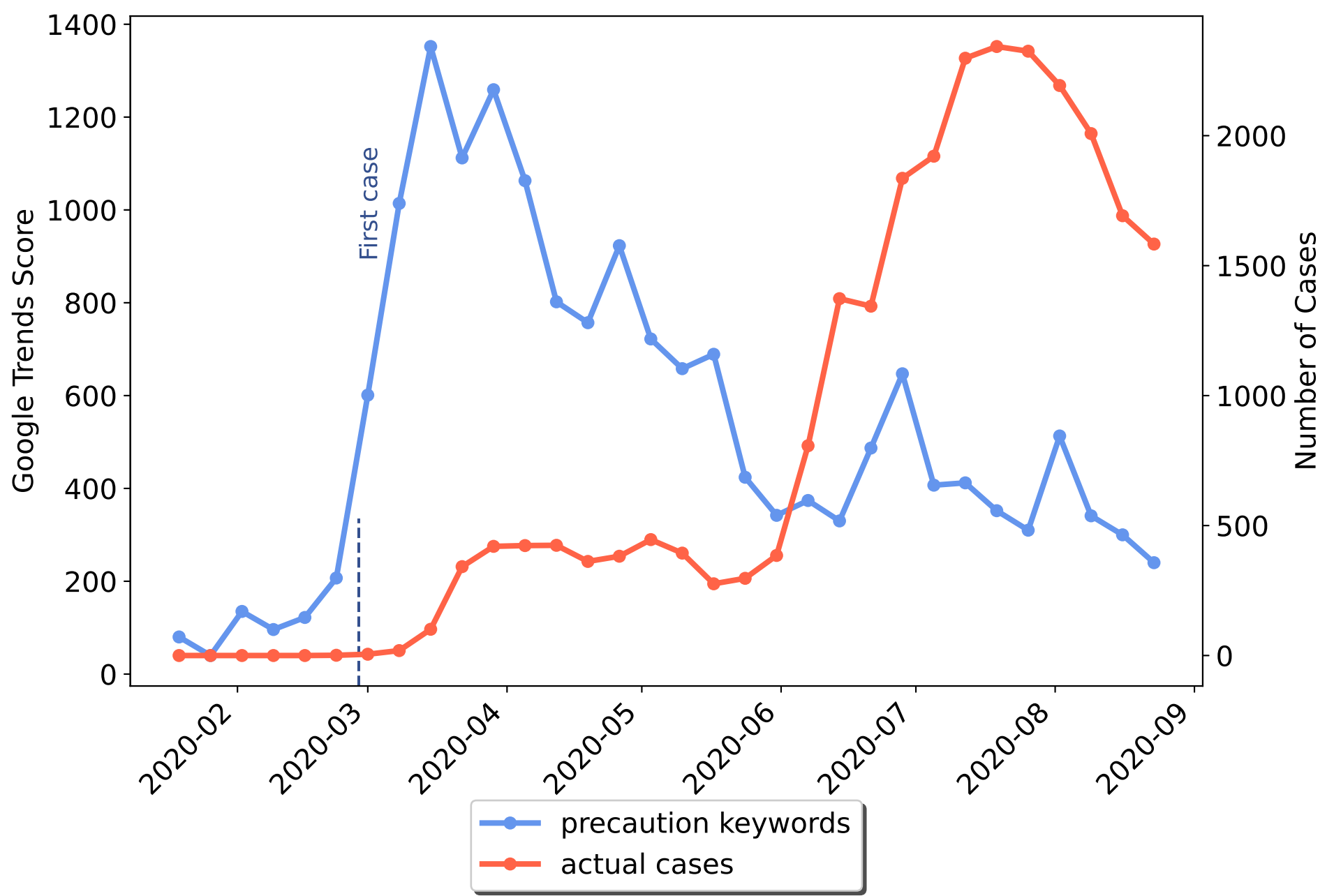

Supplement: Supplementary file 2 [file Data_Sheet_1.ZIP › figures/Oregon_totalprecaution_GT-eps-converted-to.pdf]

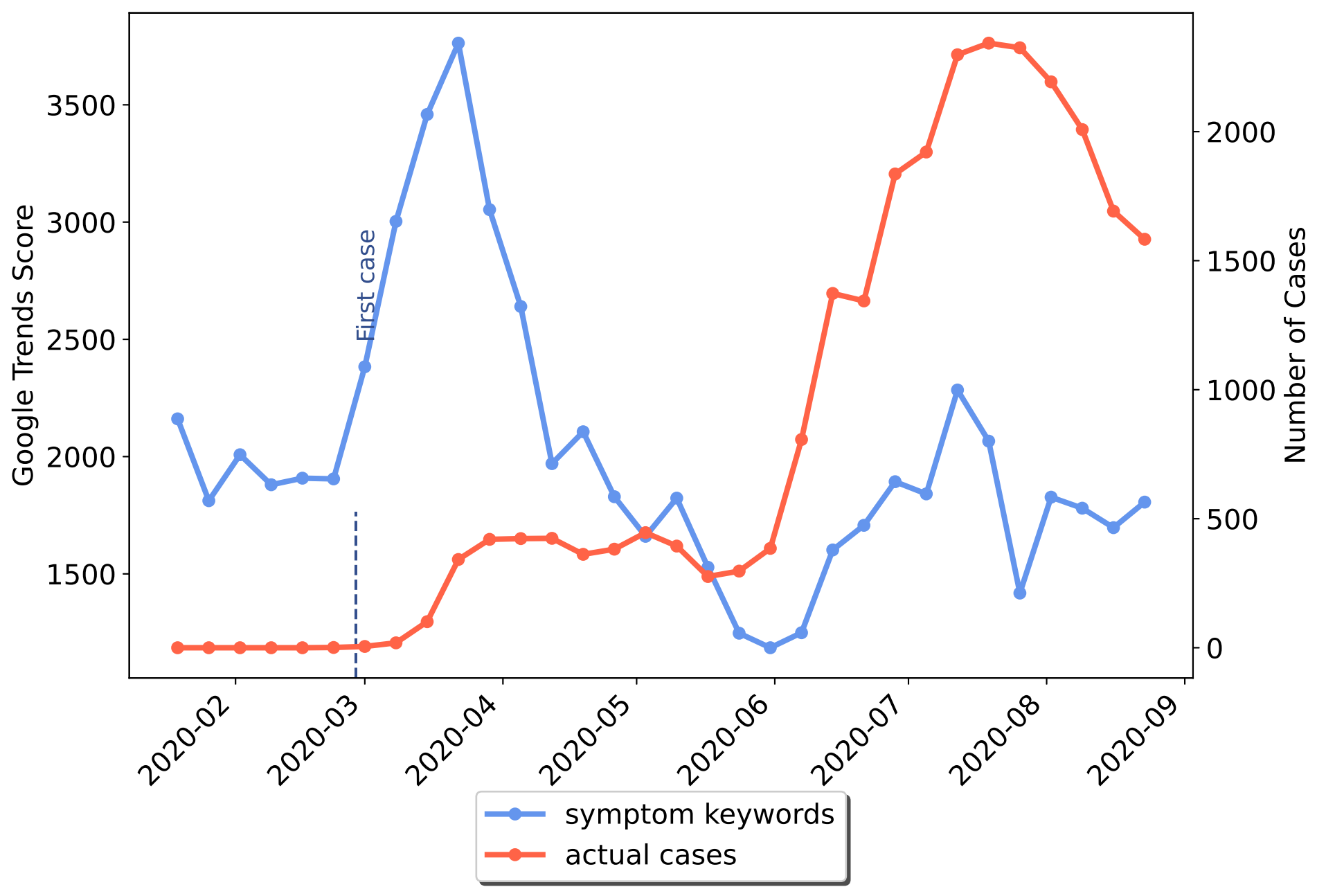

Supplement: Supplementary file 2 [file Data_Sheet_1.ZIP › figures/Oregon_totalsymptom_GT-eps-converted-to.pdf]

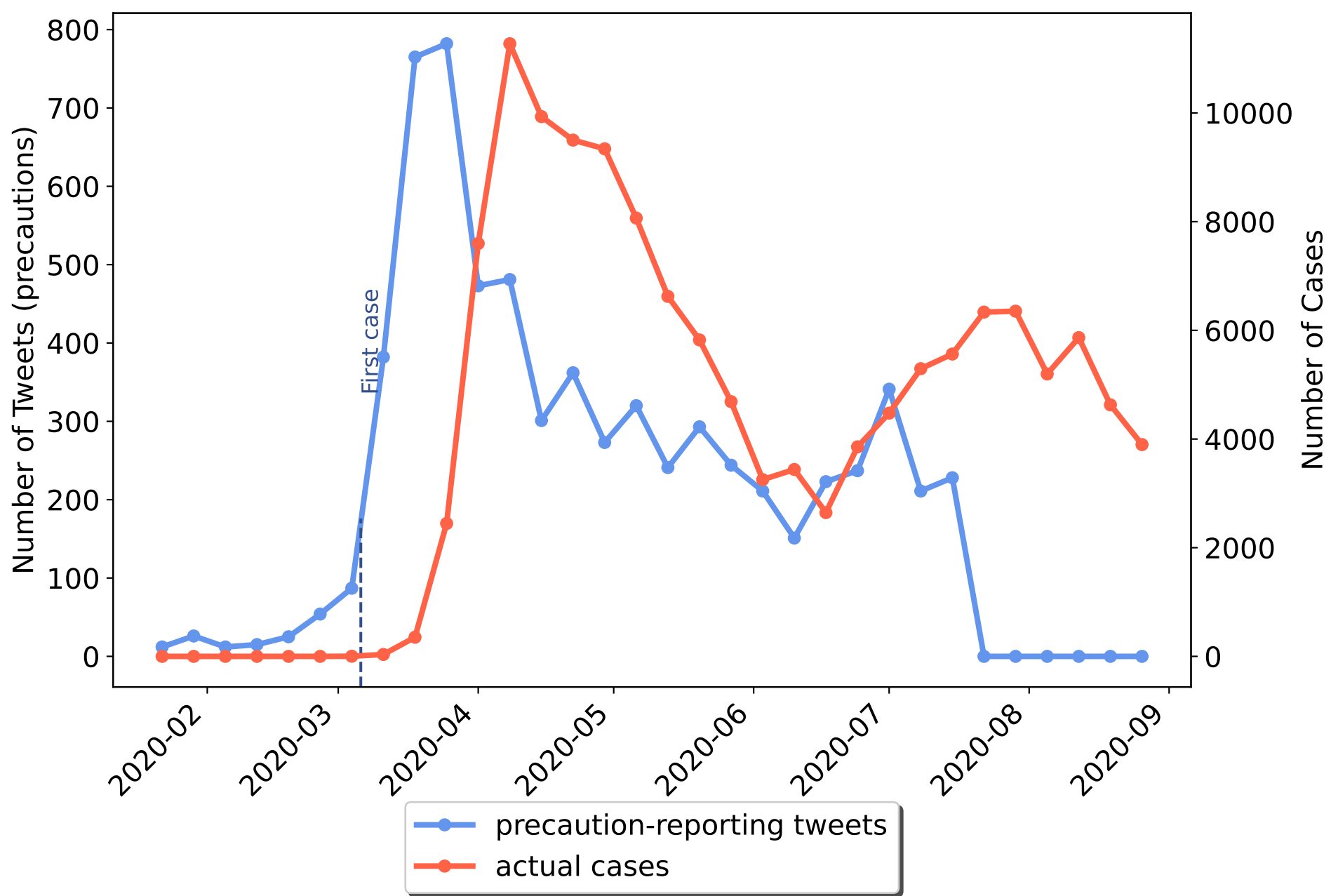

Supplement: Supplementary file 2 [file Data_Sheet_1.ZIP › figures/Pennsylvania_precaution_twitter-eps-converted-to.pdf]

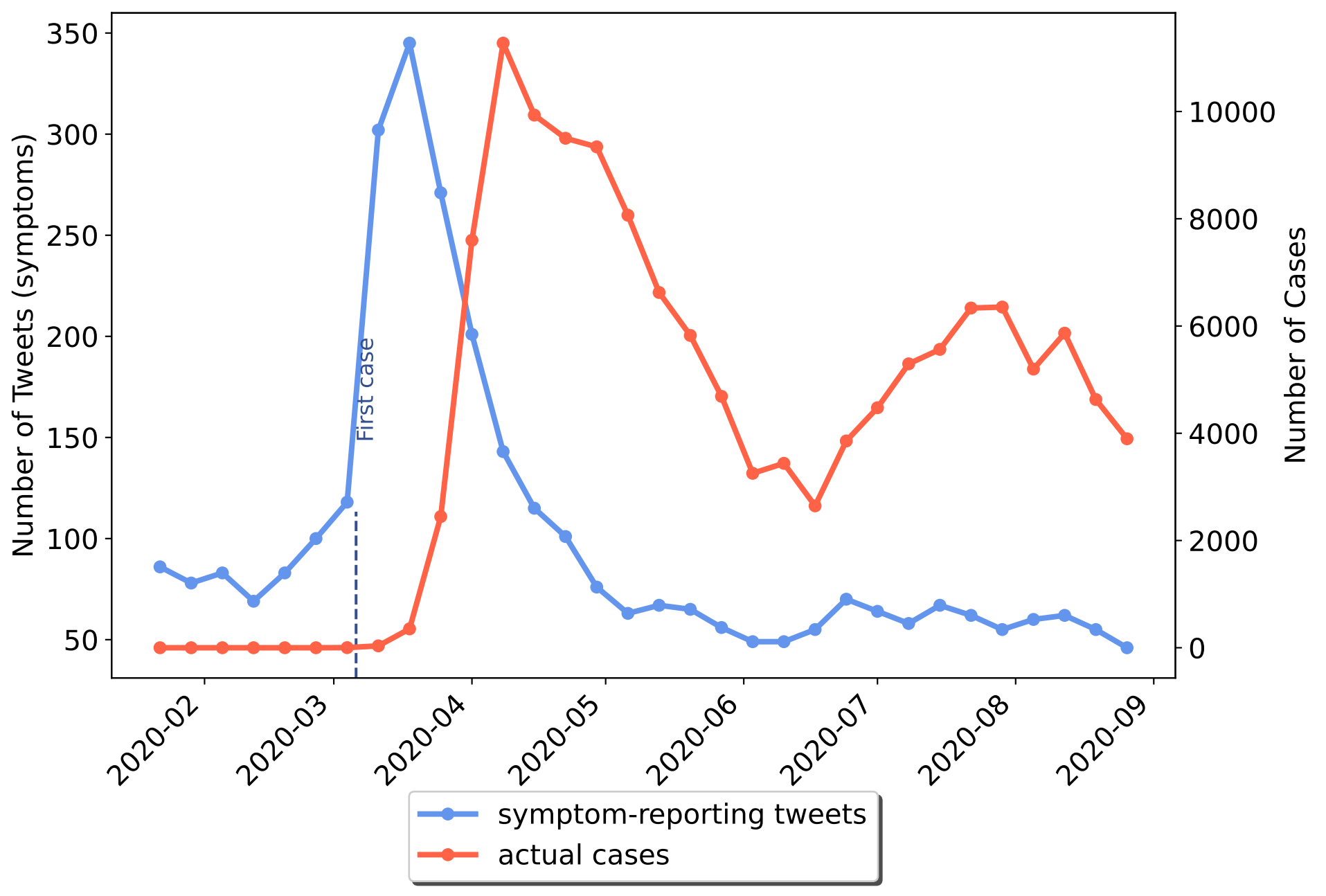

Supplement: Supplementary file 2 [file Data_Sheet_1.ZIP › figures/Pennsylvania_symptom_twitter-eps-converted-to.pdf]

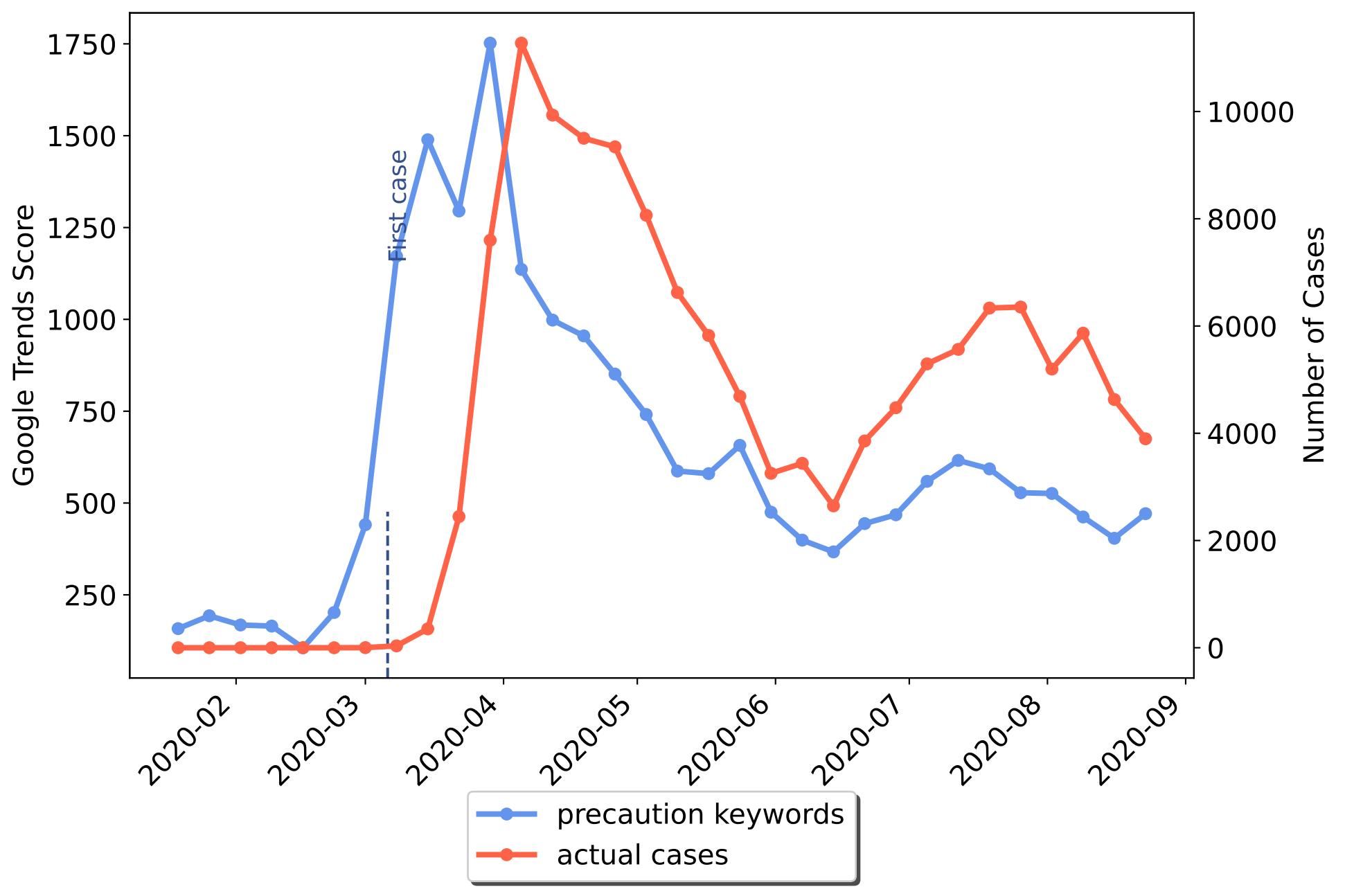

Supplement: Supplementary file 2 [file Data_Sheet_1.ZIP › figures/Pennsylvania_totalprecaution_GT-eps-converted-to.pdf]

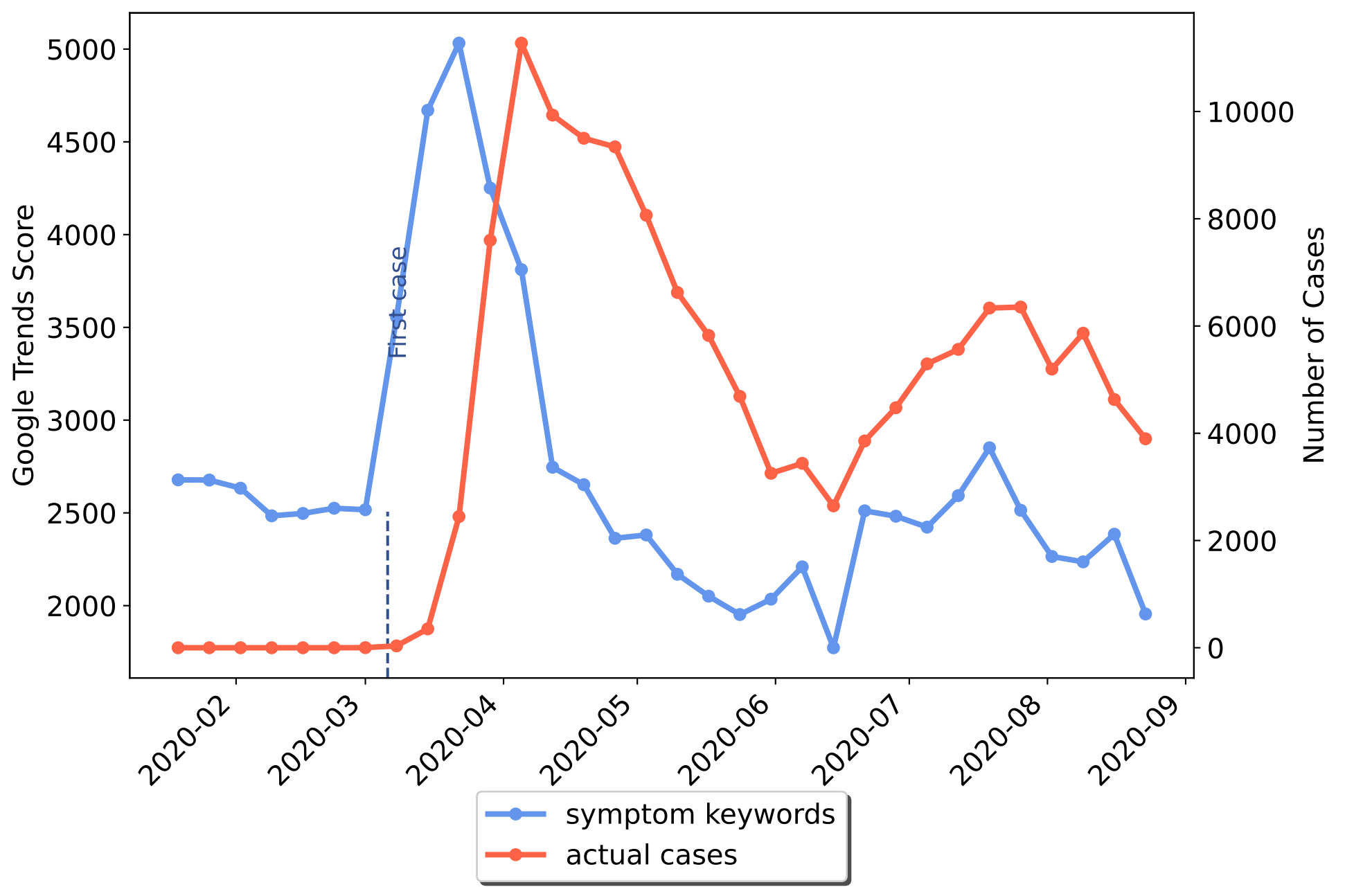

Supplement: Supplementary file 2 [file Data_Sheet_1.ZIP › figures/Pennsylvania_totalsymptom_GT-eps-converted-to.pdf]

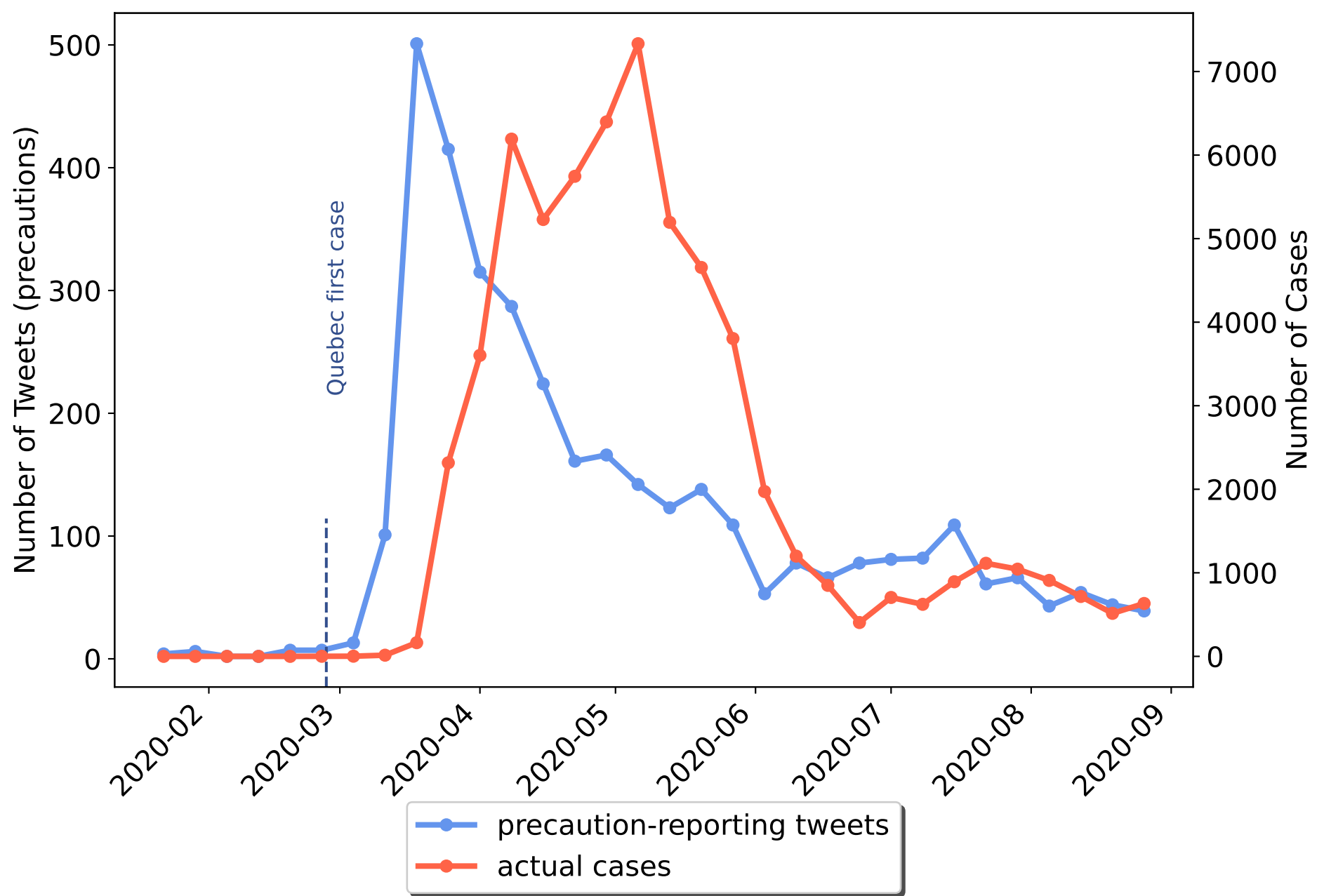

Supplement: Supplementary file 2 [file Data_Sheet_1.ZIP › figures/Quebec_precaution_twitter-eps-converted-to.pdf]

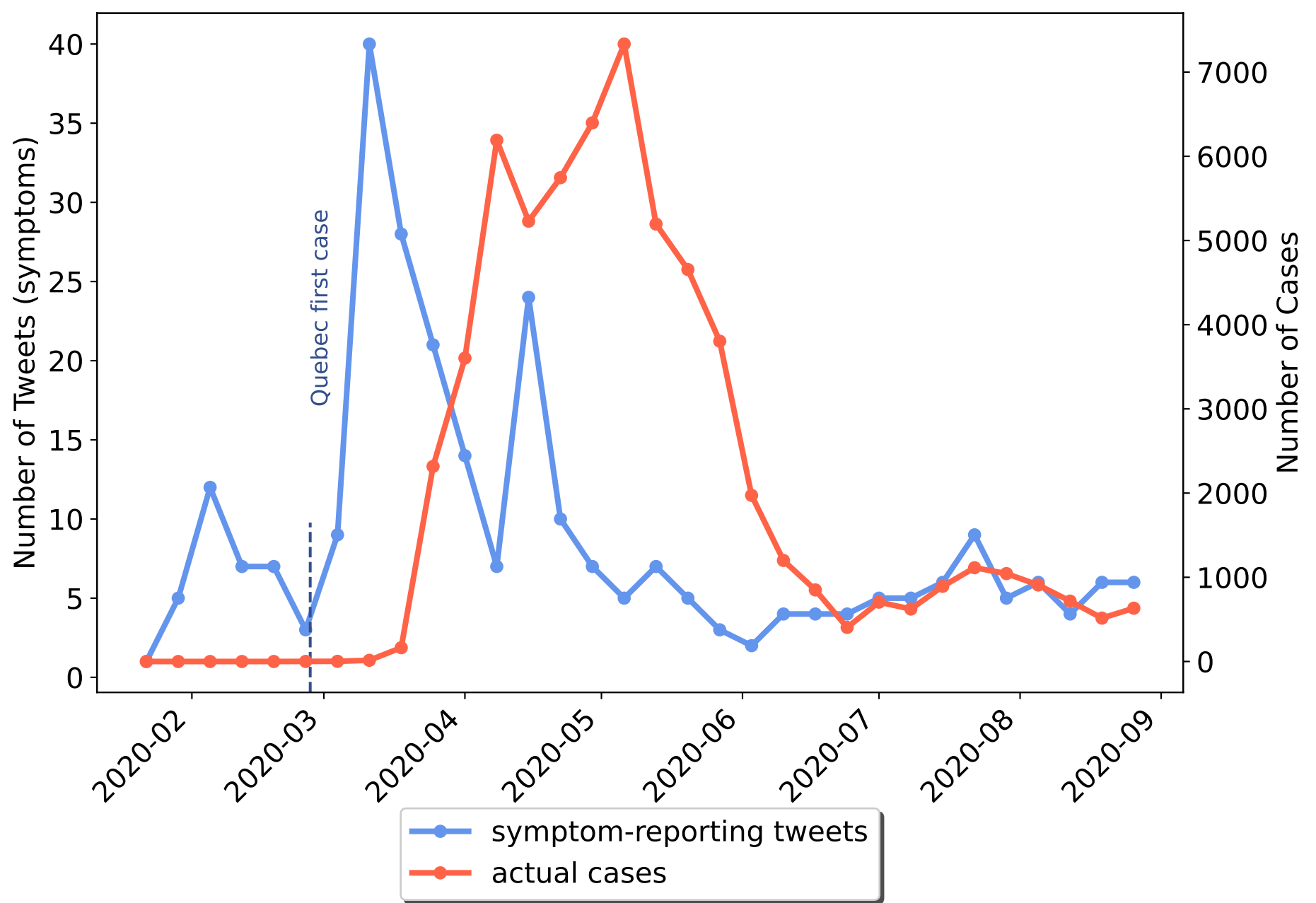

Supplement: Supplementary file 2 [file Data_Sheet_1.ZIP › figures/Quebec_symptom_twitter-eps-converted-to.pdf]

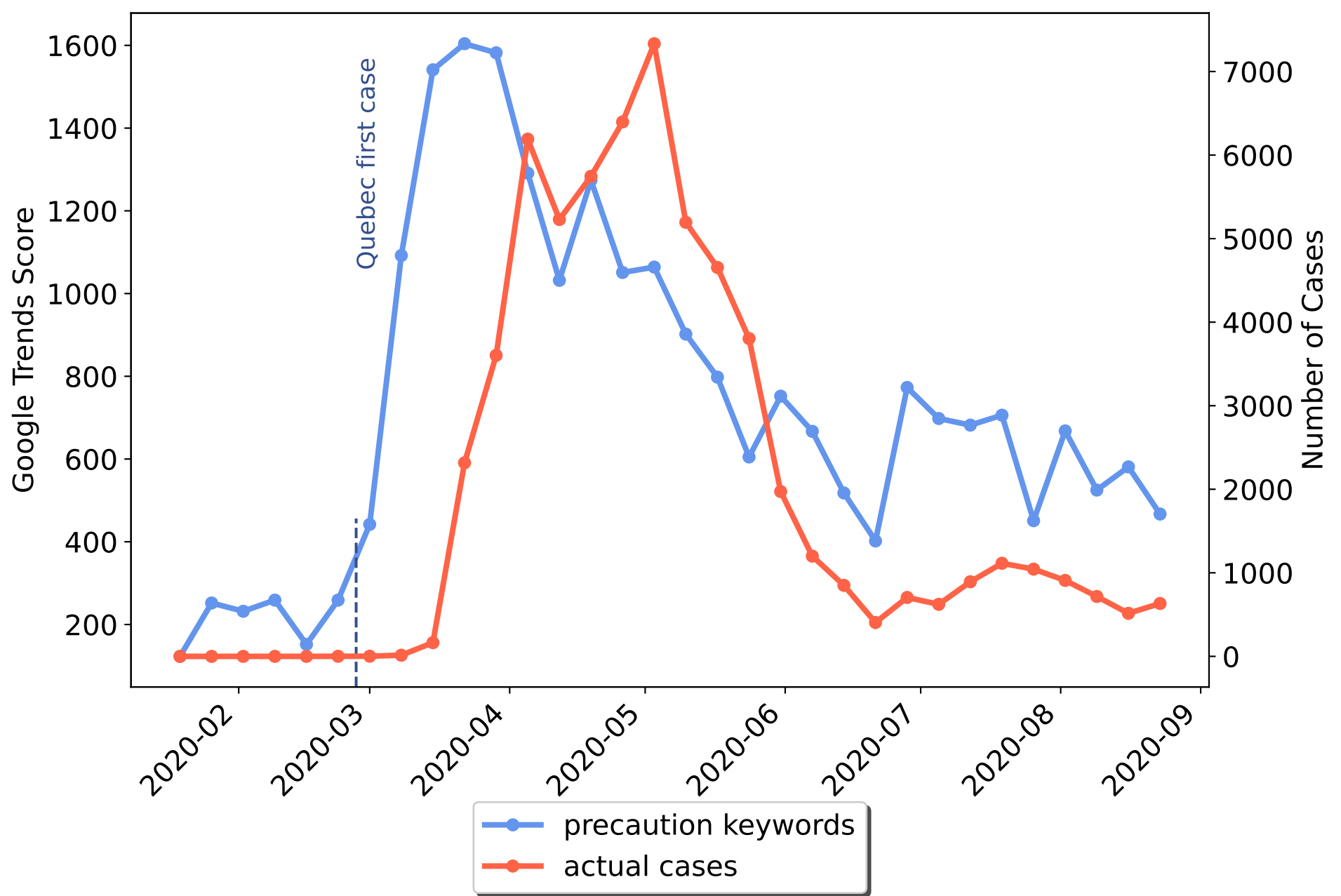

Supplement: Supplementary file 2 [file Data_Sheet_1.ZIP › figures/Quebec_totalprecaution_GT-eps-converted-to.pdf]

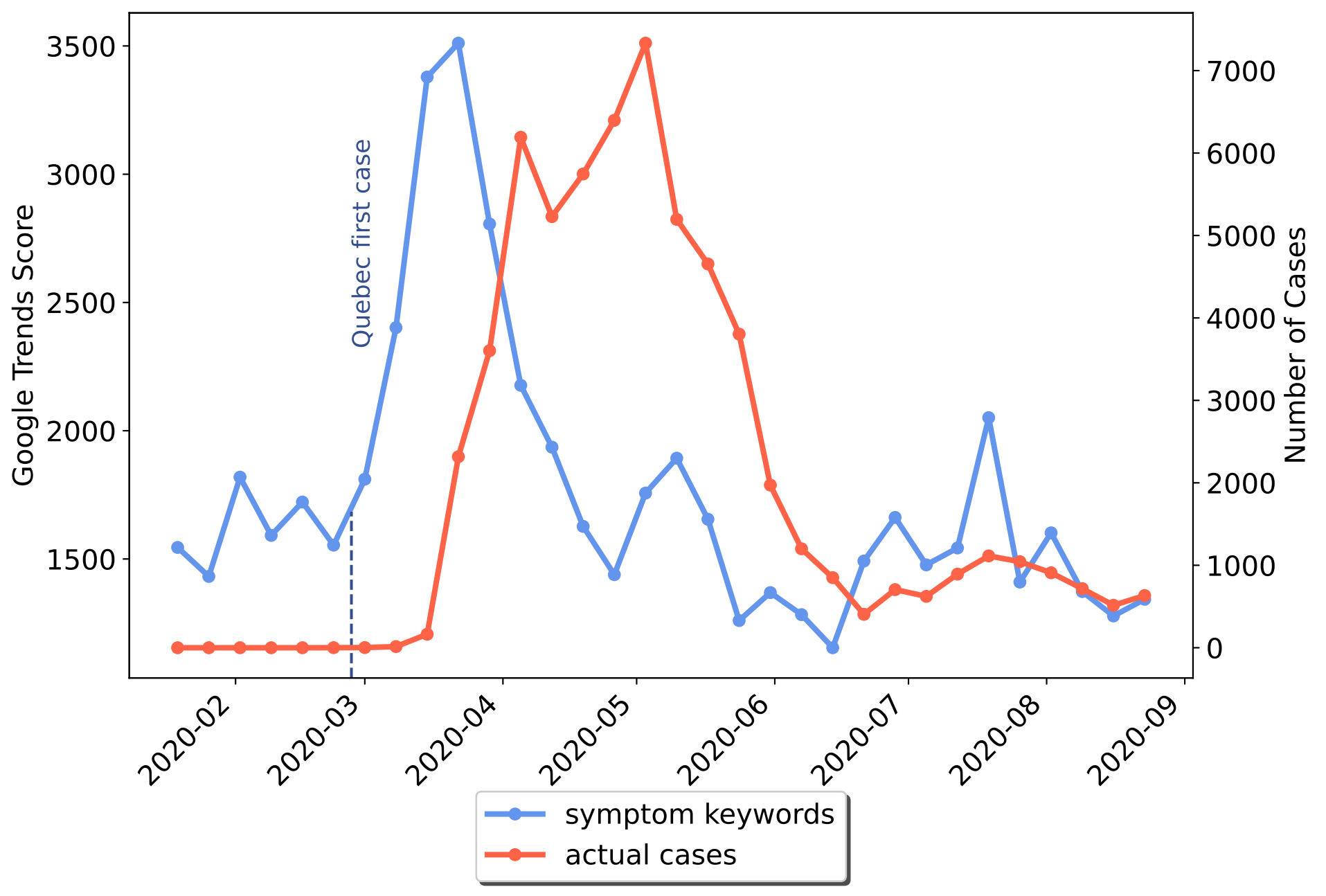

Supplement: Supplementary file 2 [file Data_Sheet_1.ZIP › figures/Quebec_totalsymptom_GT-eps-converted-to.pdf]

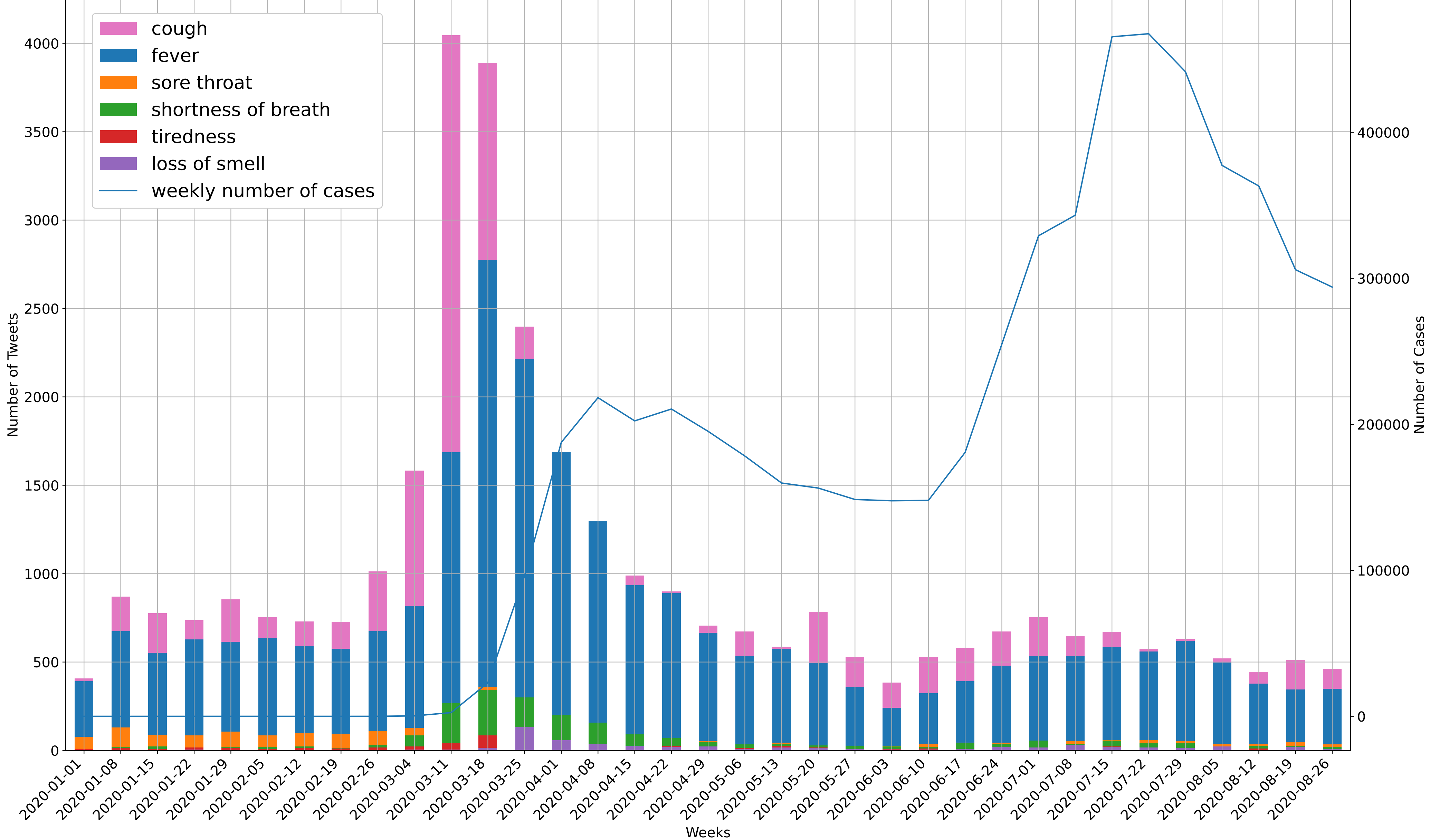

Supplement: Supplementary file 2 [file Data_Sheet_1.ZIP › figures/stacked_bar_chart_symptoms_twitter_united_states-eps-converted-to.pdf]

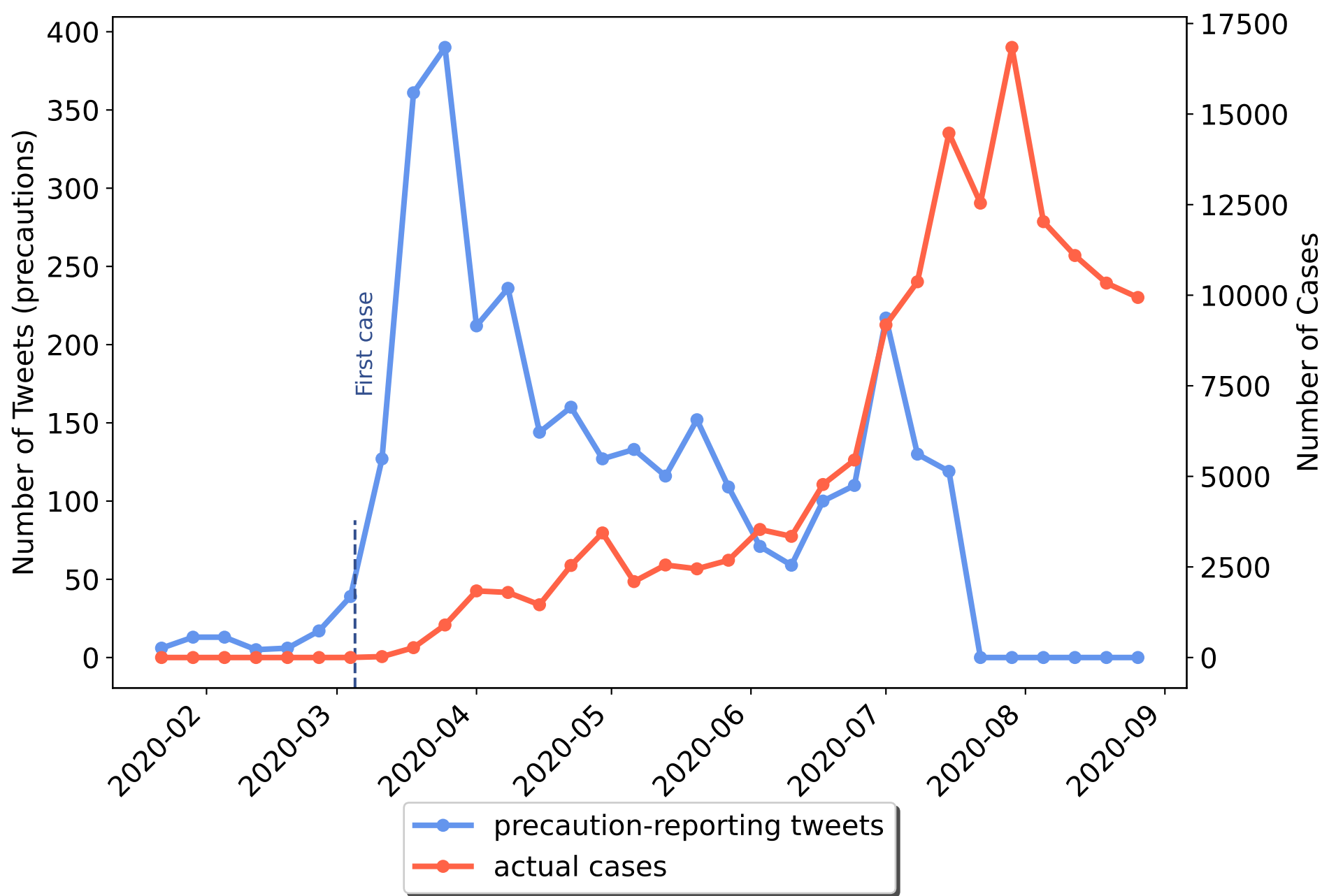

Supplement: Supplementary file 2 [file Data_Sheet_1.ZIP › figures/Tennessee_precaution_twitter-eps-converted-to.pdf]

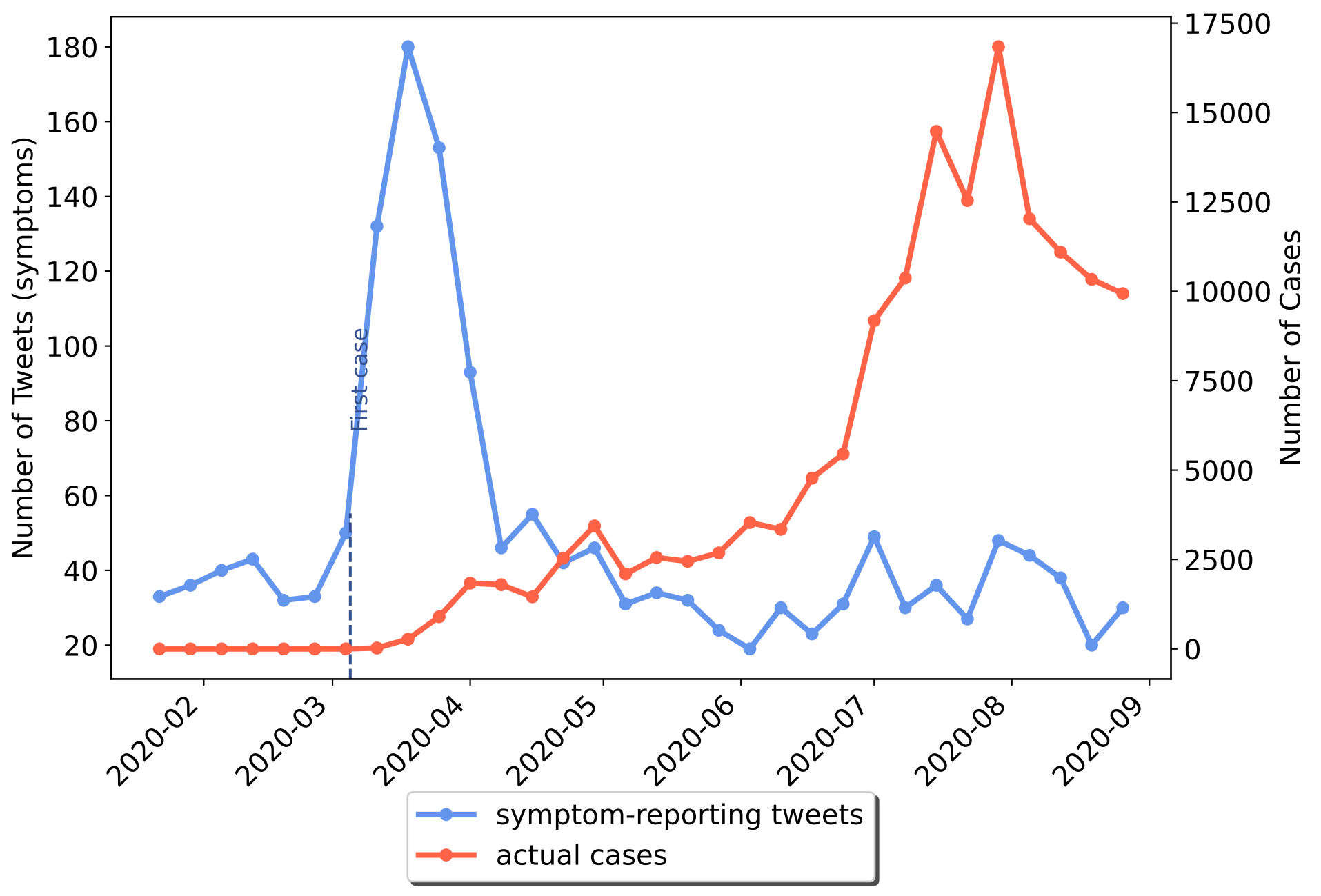

Supplement: Supplementary file 2 [file Data_Sheet_1.ZIP › figures/Tennessee_symptom_twitter-eps-converted-to.pdf]

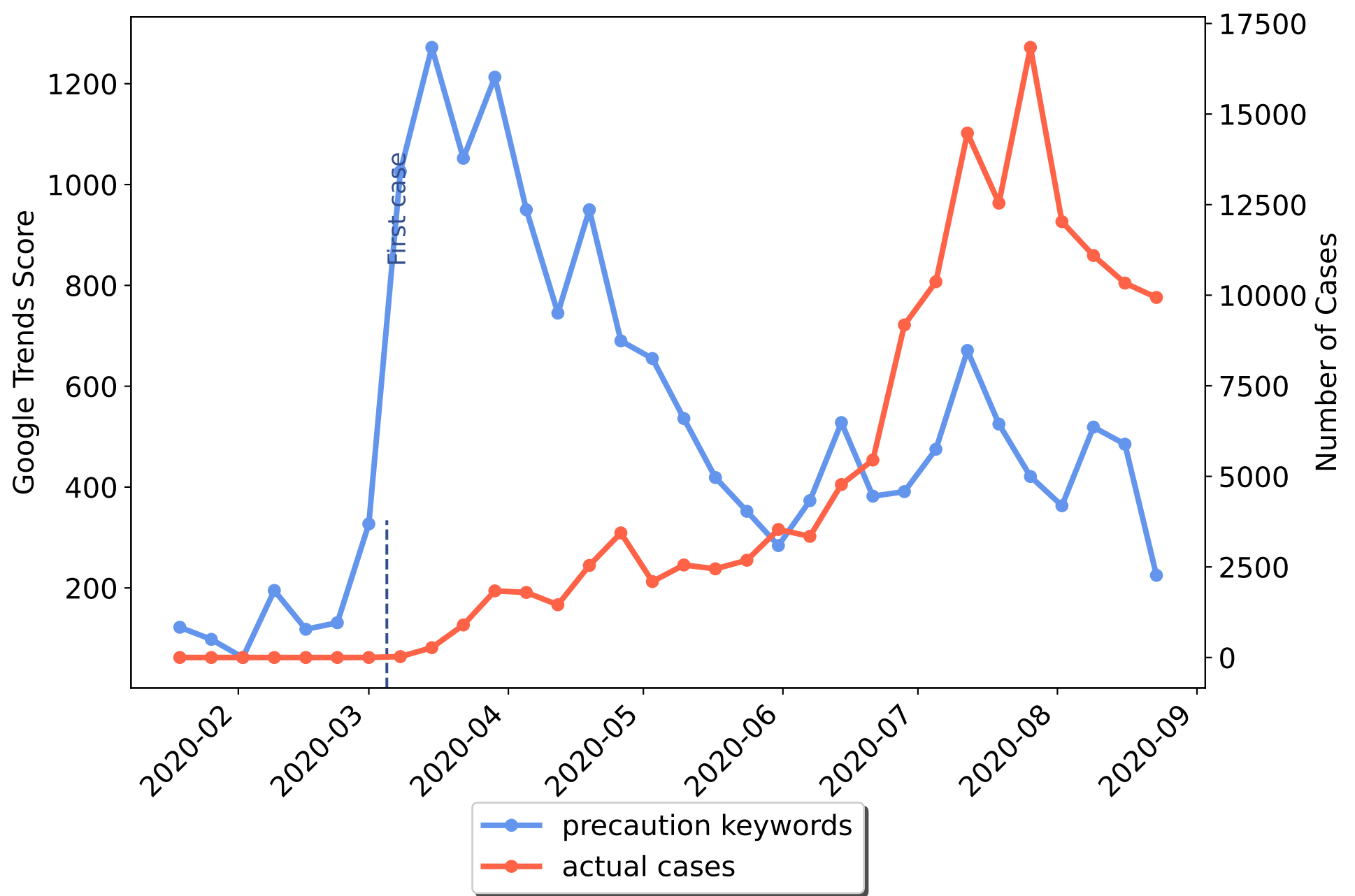

Supplement: Supplementary file 2 [file Data_Sheet_1.ZIP › figures/Tennessee_totalprecaution_GT-eps-converted-to.pdf]

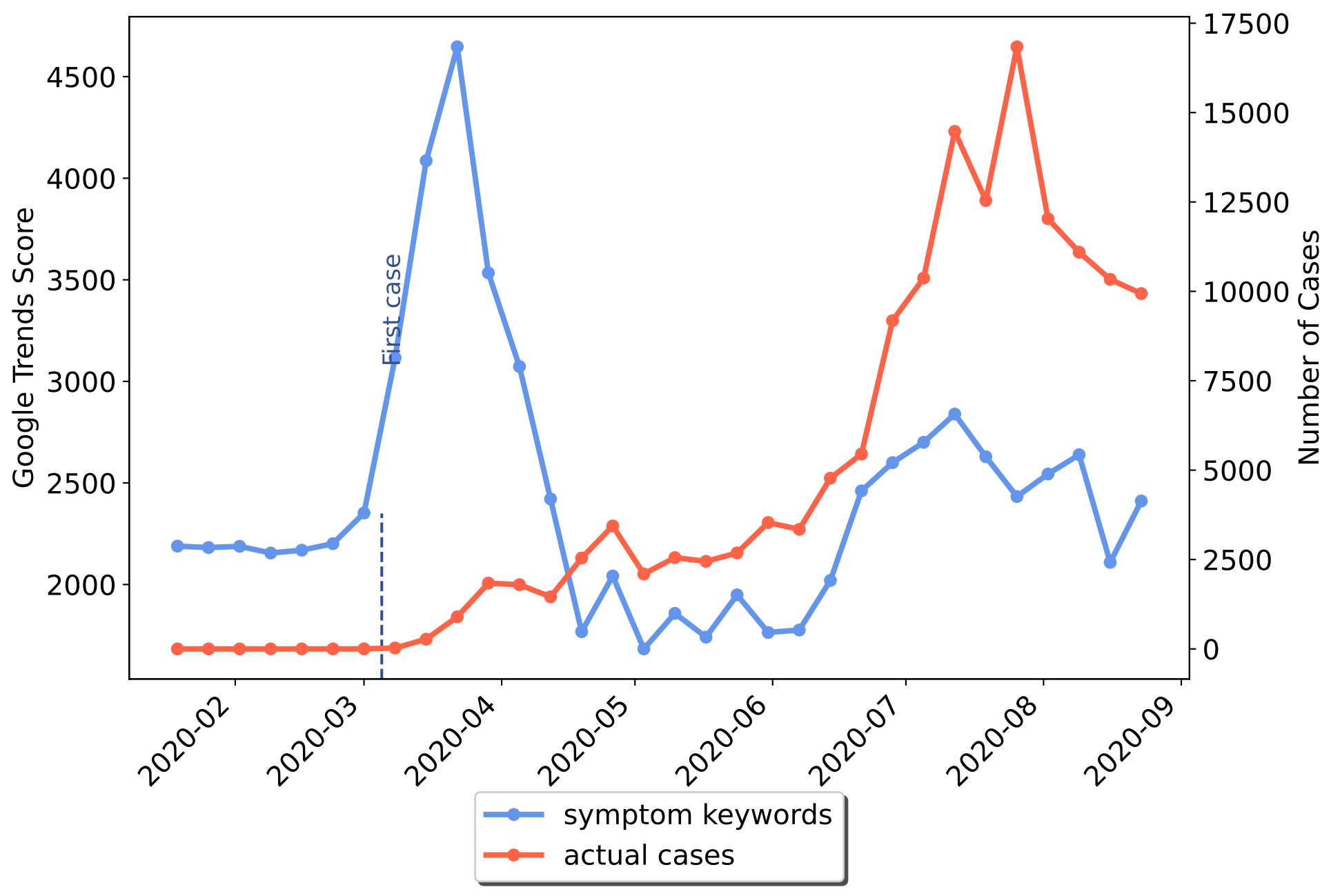

Supplement: Supplementary file 2 [file Data_Sheet_1.ZIP › figures/Tennessee_totalsymptom_GT-eps-converted-to.pdf]

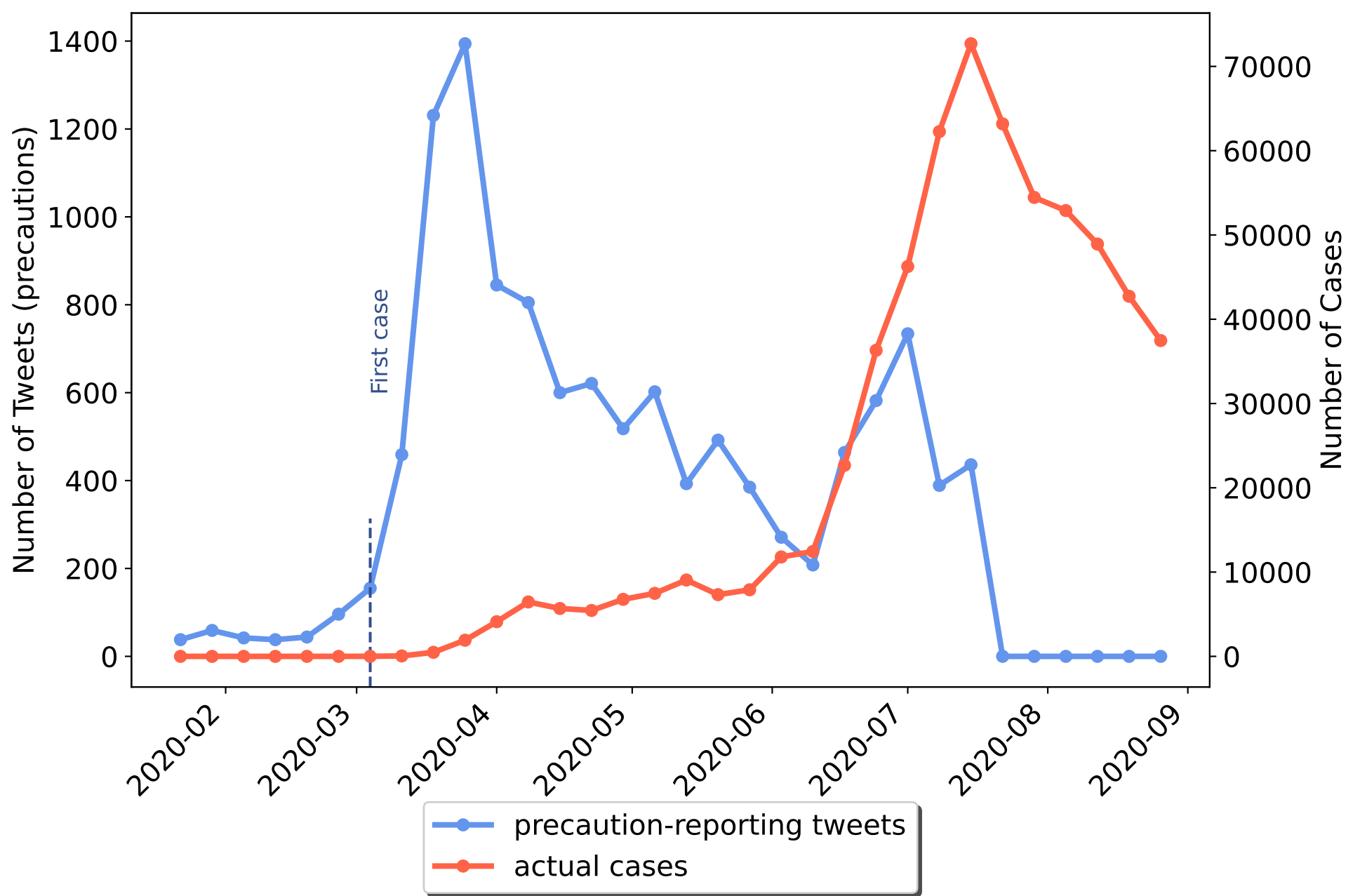

Supplement: Supplementary file 2 [file Data_Sheet_1.ZIP › figures/Texas_precaution_twitter-eps-converted-to.pdf]

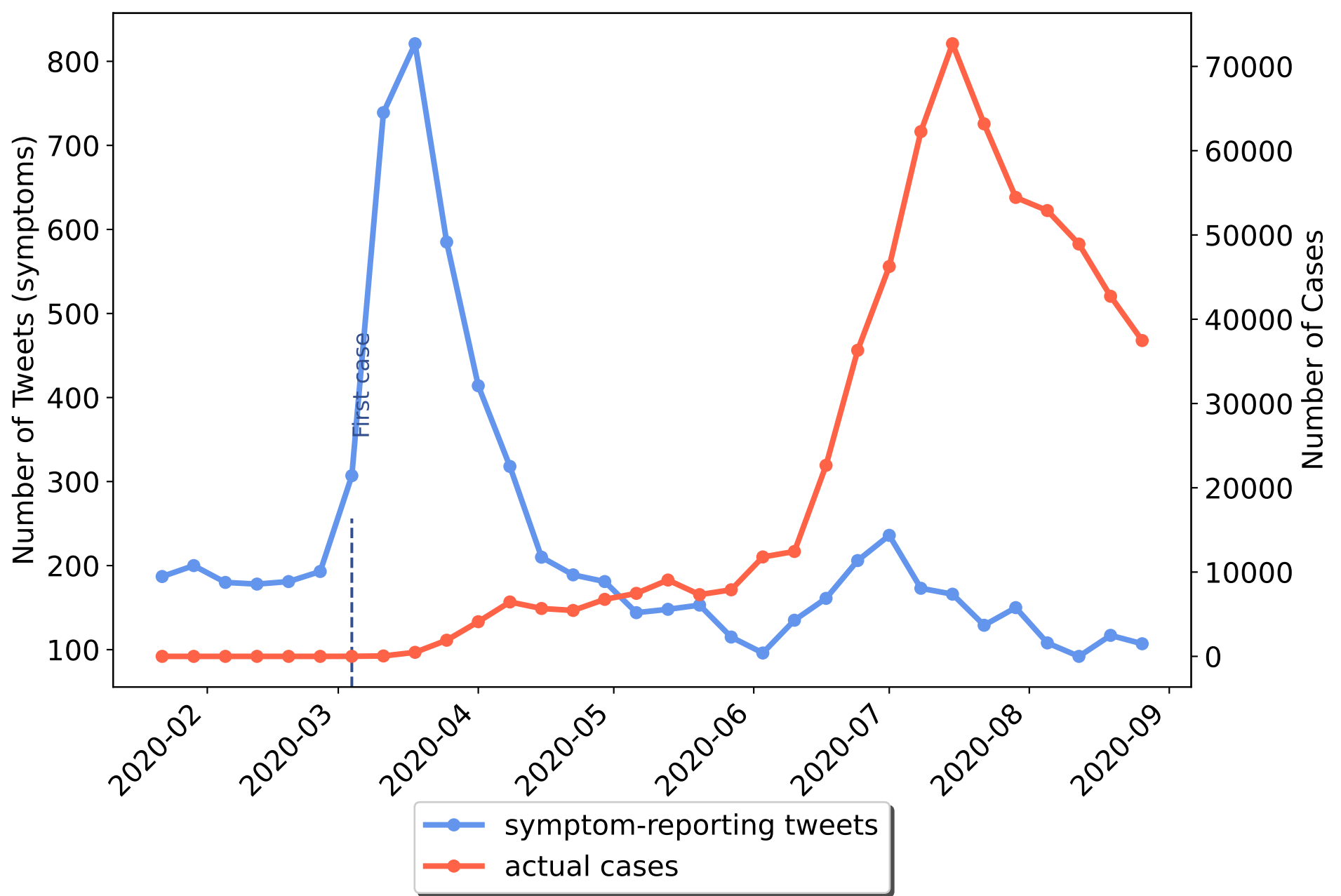

Supplement: Supplementary file 2 [file Data_Sheet_1.ZIP › figures/Texas_symptom_twitter-eps-converted-to.pdf]

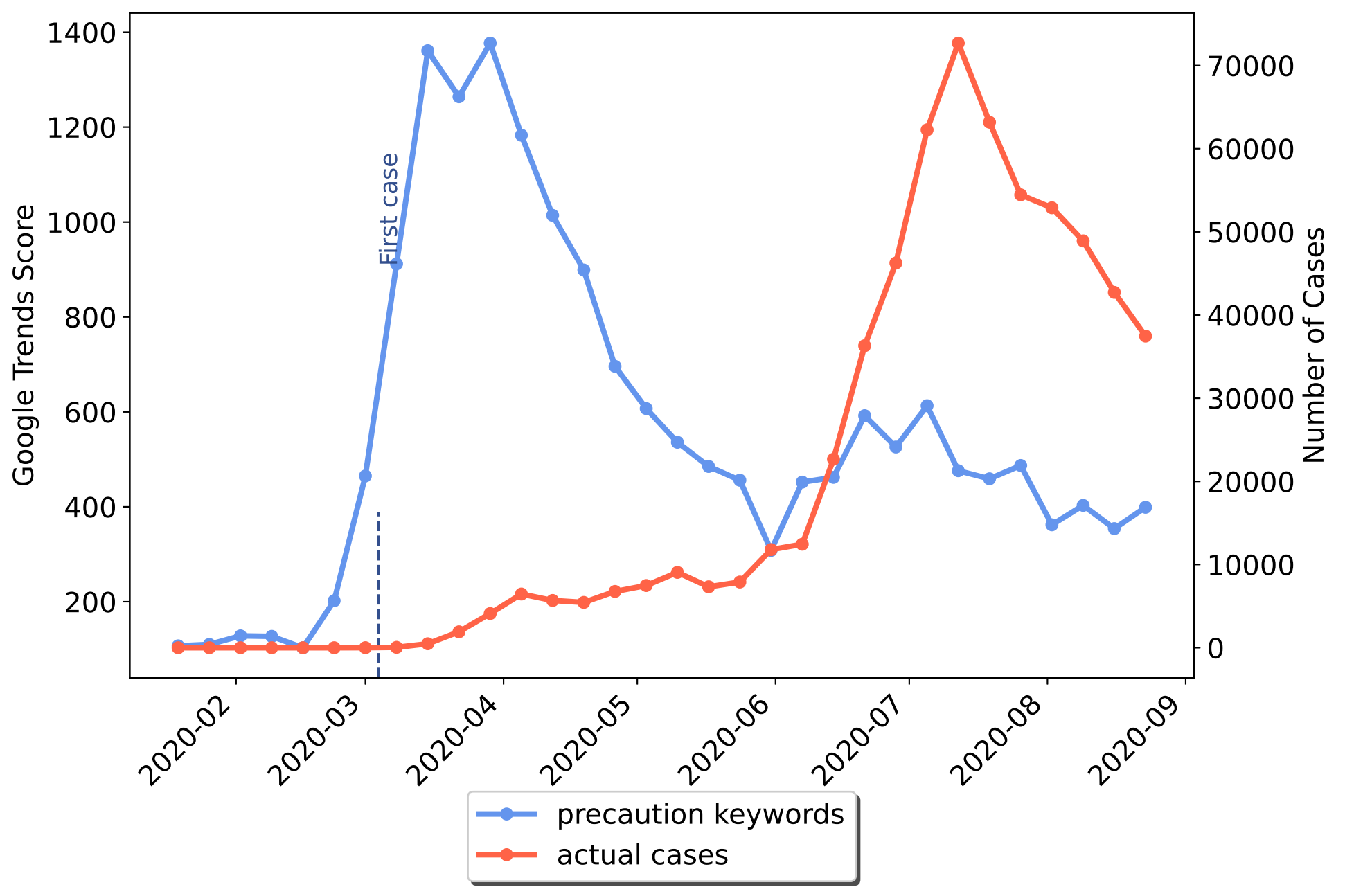

Supplement: Supplementary file 2 [file Data_Sheet_1.ZIP › figures/Texas_totalprecaution_GT-eps-converted-to.pdf]

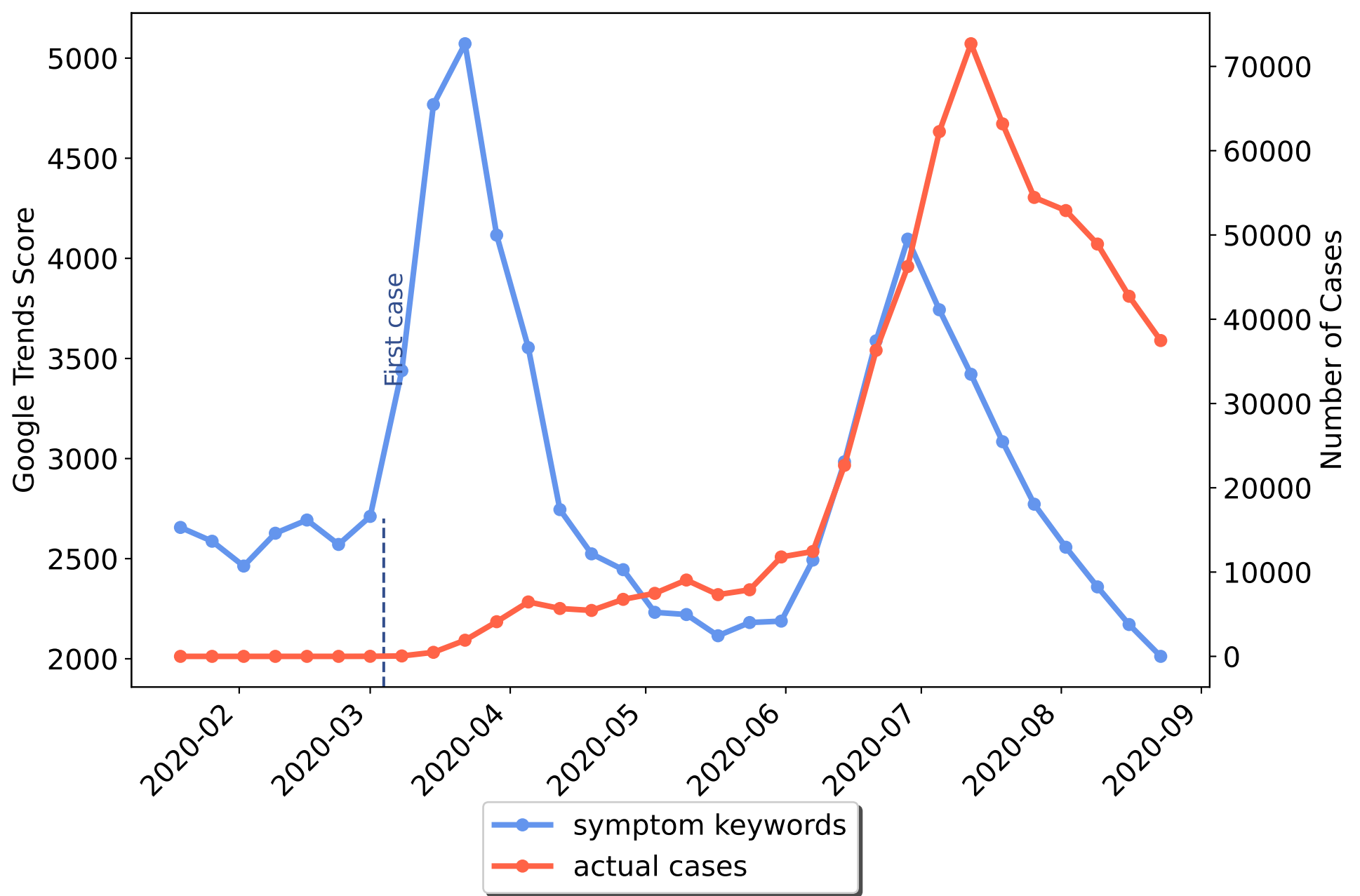

Supplement: Supplementary file 2 [file Data_Sheet_1.ZIP › figures/Texas_totalsymptom_GT-eps-converted-to.pdf]

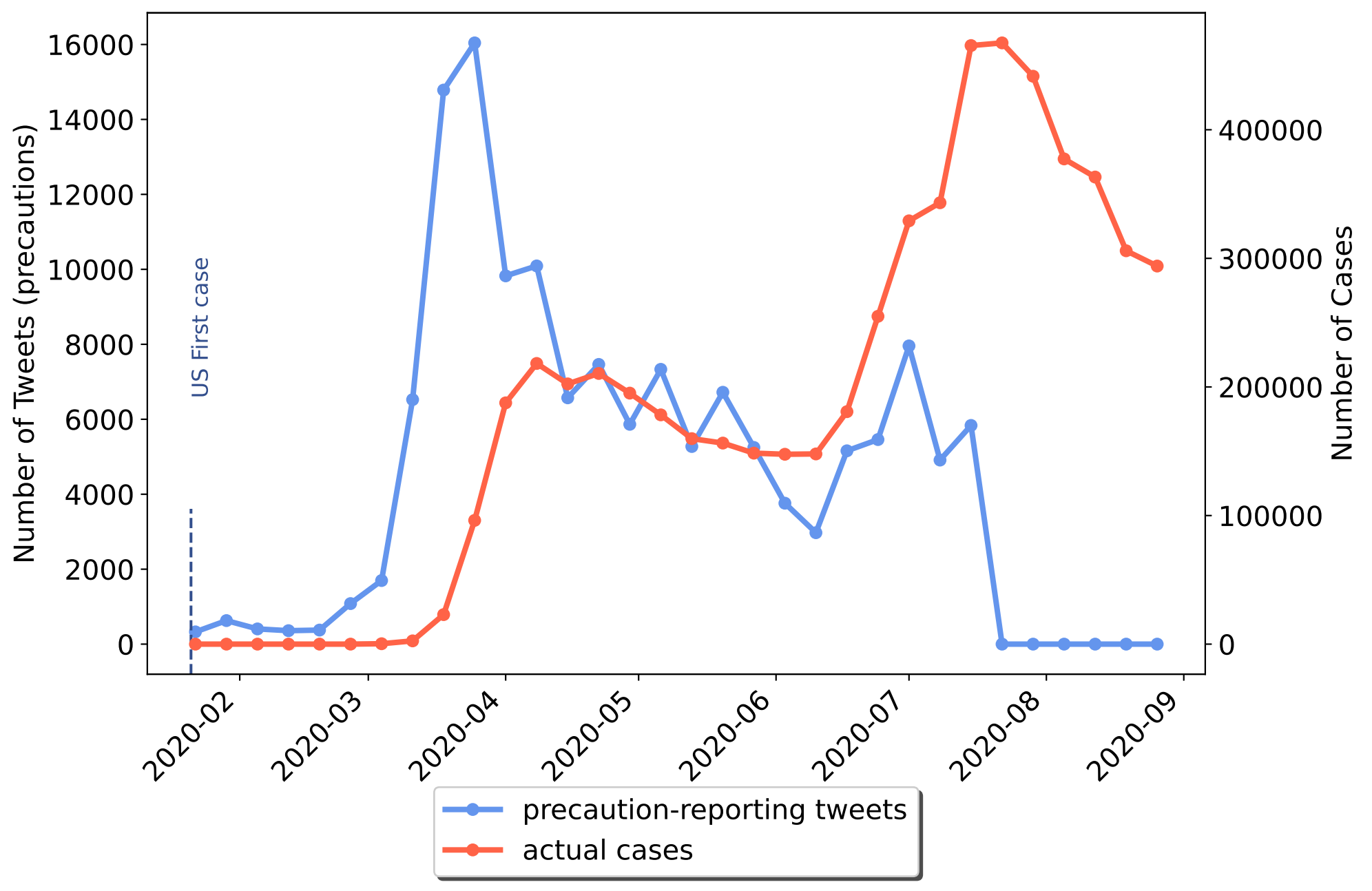

Supplement: Supplementary file 2 [file Data_Sheet_1.ZIP › figures/united_states_precaution_twitter-eps-converted-to.pdf]

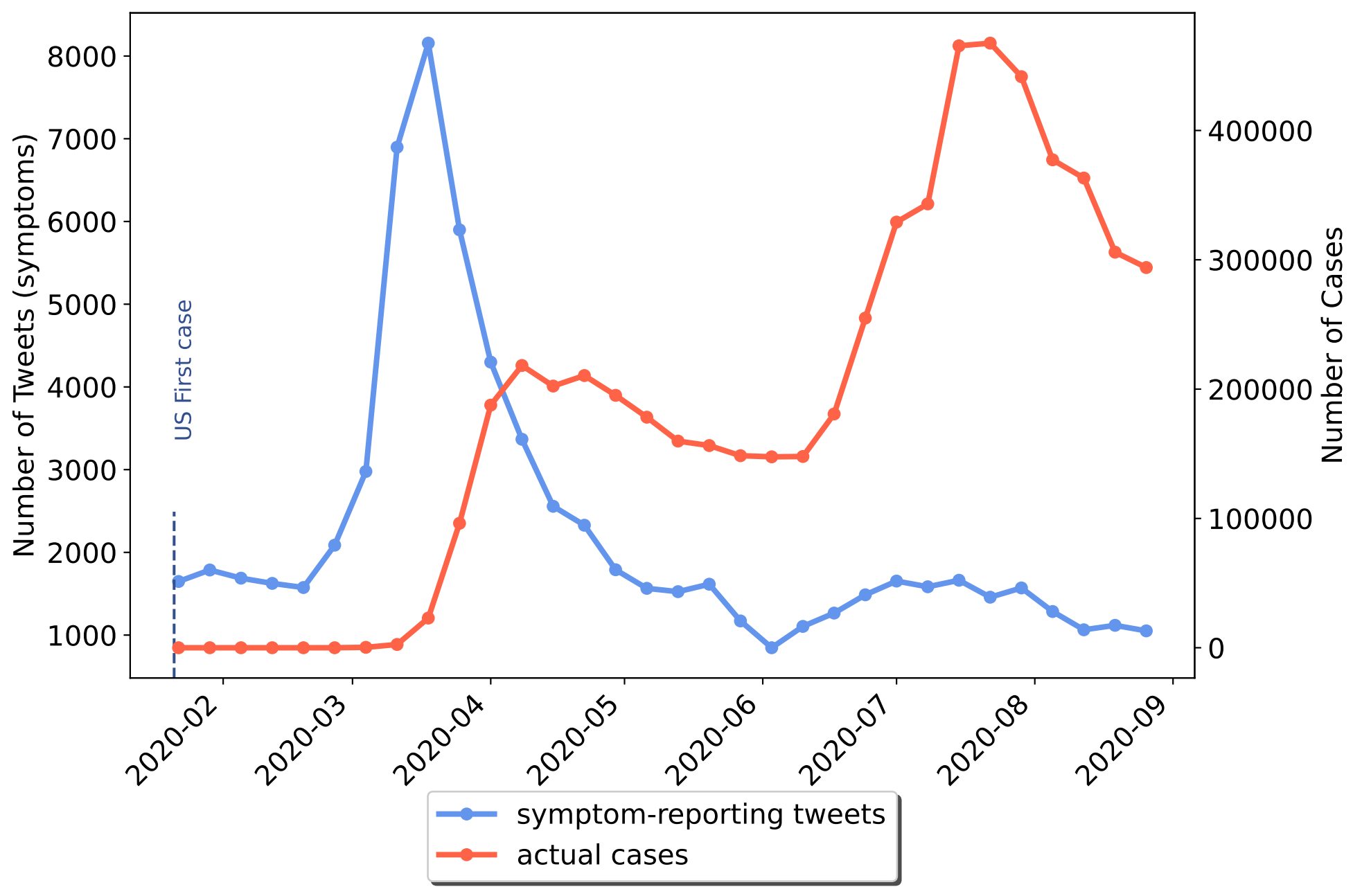

Supplement: Supplementary file 2 [file Data_Sheet_1.ZIP › figures/united_states_symptom_twitter-eps-converted-to.pdf]

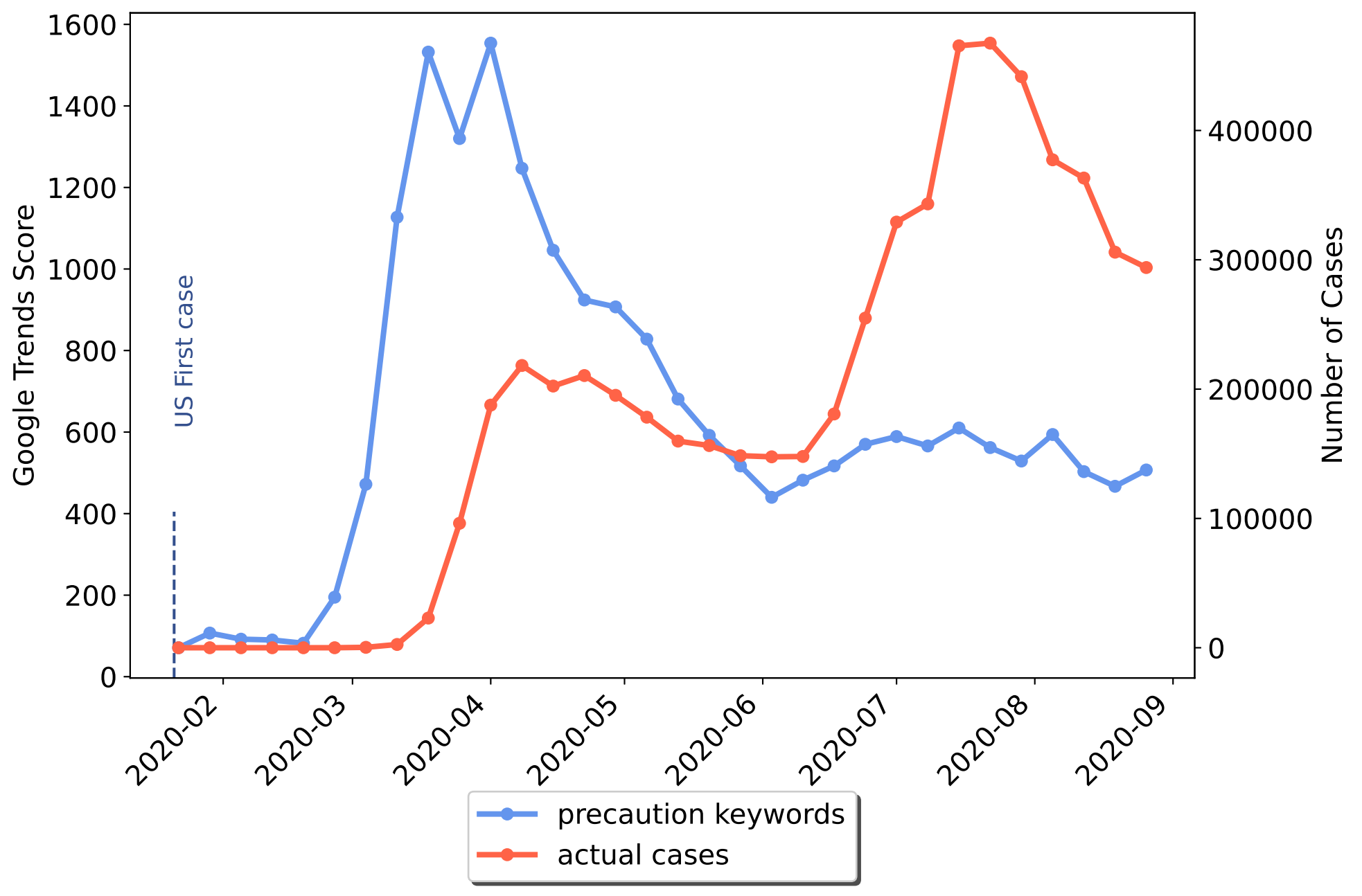

Supplement: Supplementary file 2 [file Data_Sheet_1.ZIP › figures/united_states_totalprecaution_GT-eps-converted-to.pdf]

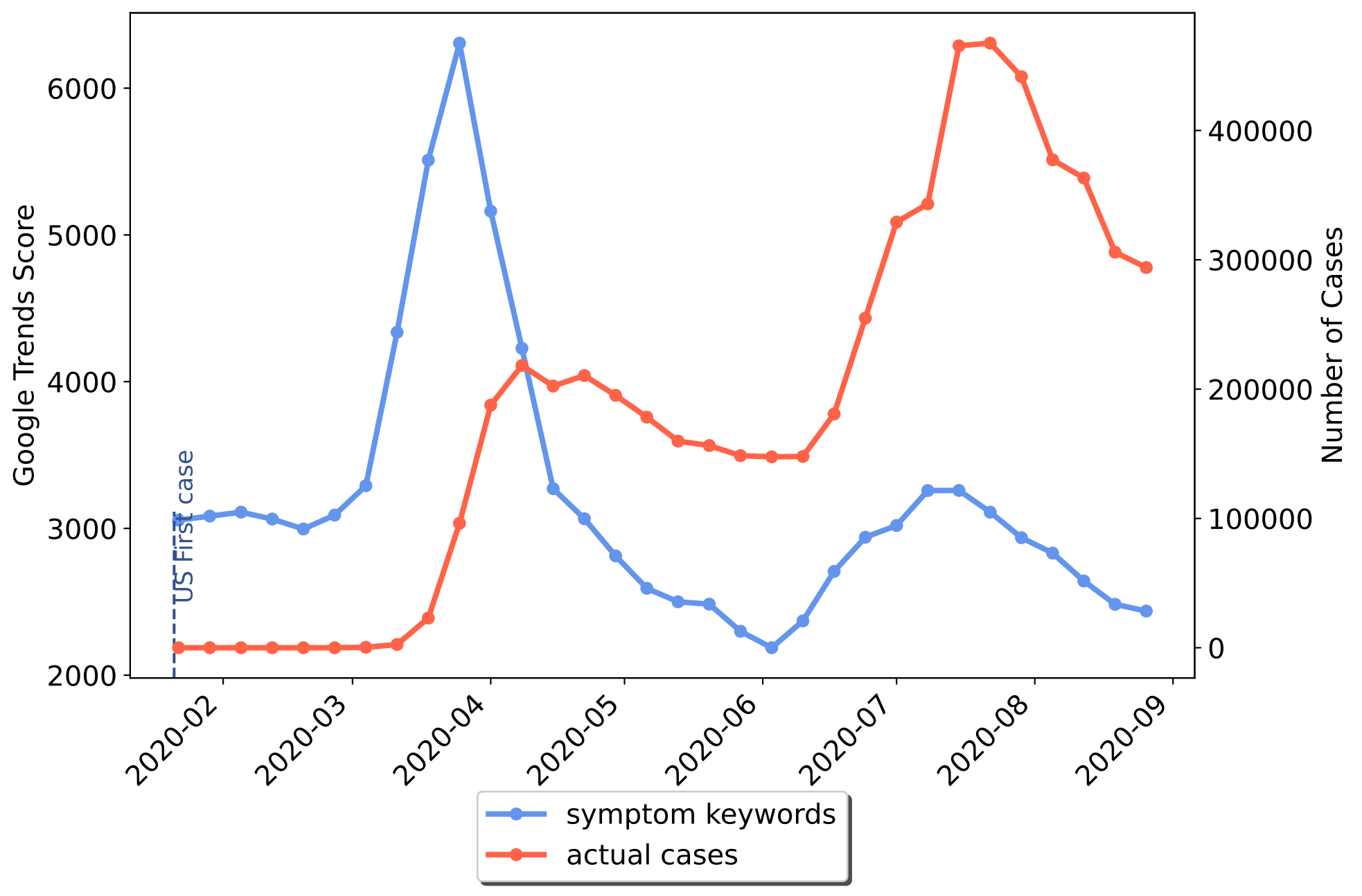

Supplement: Supplementary file 2 [file Data_Sheet_1.ZIP › figures/united_states_totalsymptom_GT-eps-converted-to.pdf]

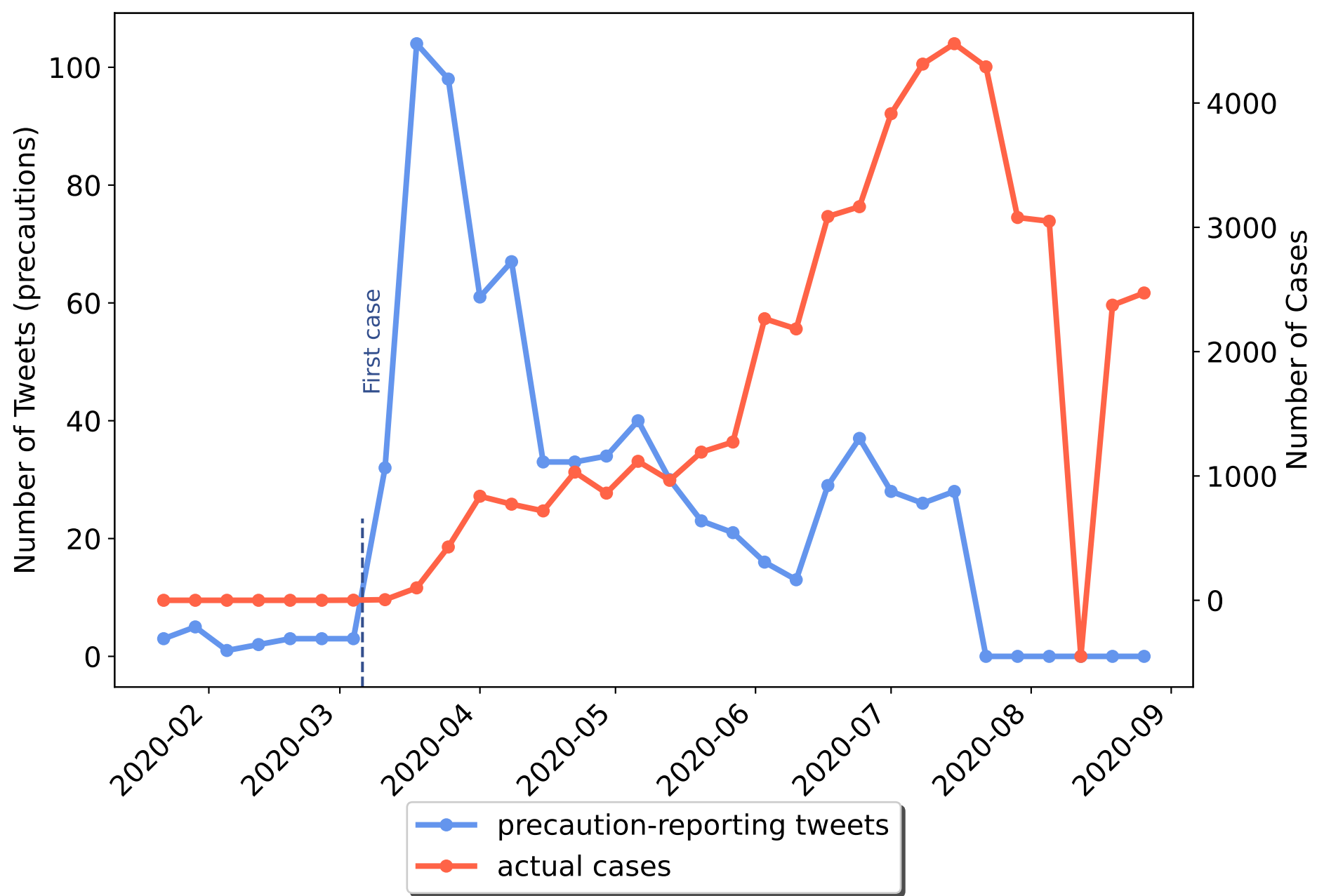

Supplement: Supplementary file 2 [file Data_Sheet_1.ZIP › figures/Utah_precaution_twitter-eps-converted-to.pdf]

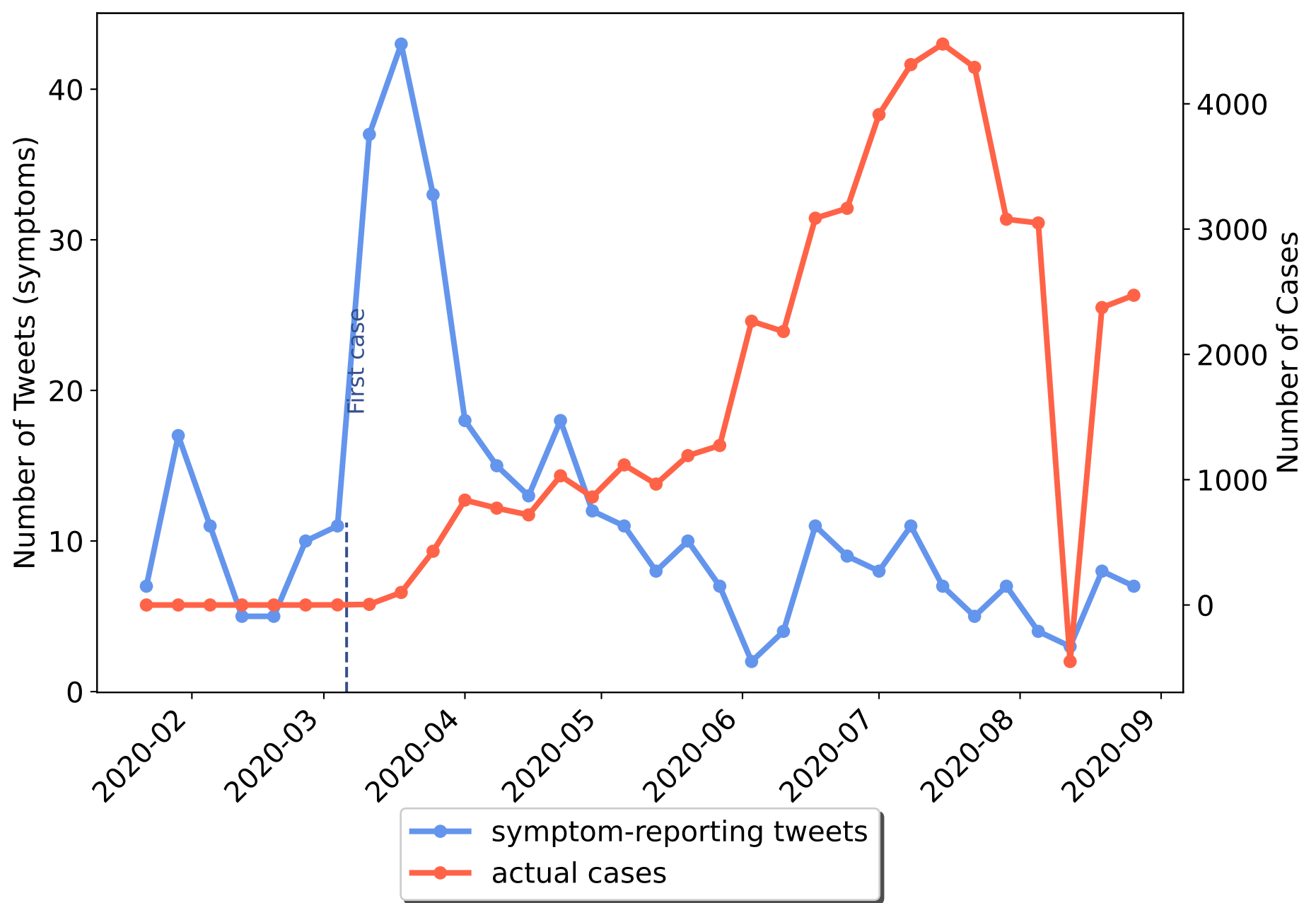

Supplement: Supplementary file 2 [file Data_Sheet_1.ZIP › figures/Utah_symptom_twitter-eps-converted-to.pdf]

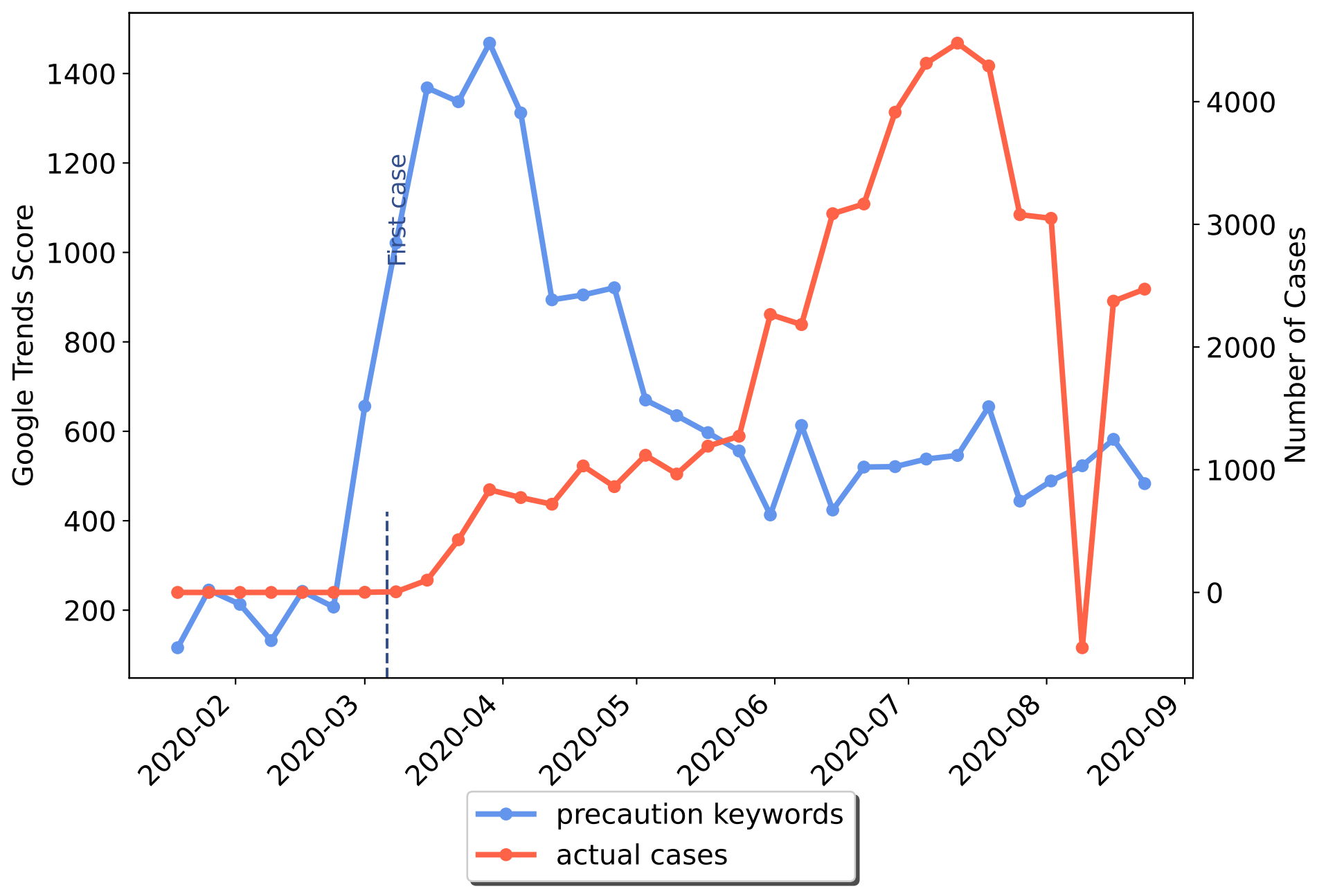

Supplement: Supplementary file 2 [file Data_Sheet_1.ZIP › figures/Utah_totalprecaution_GT-eps-converted-to.pdf]

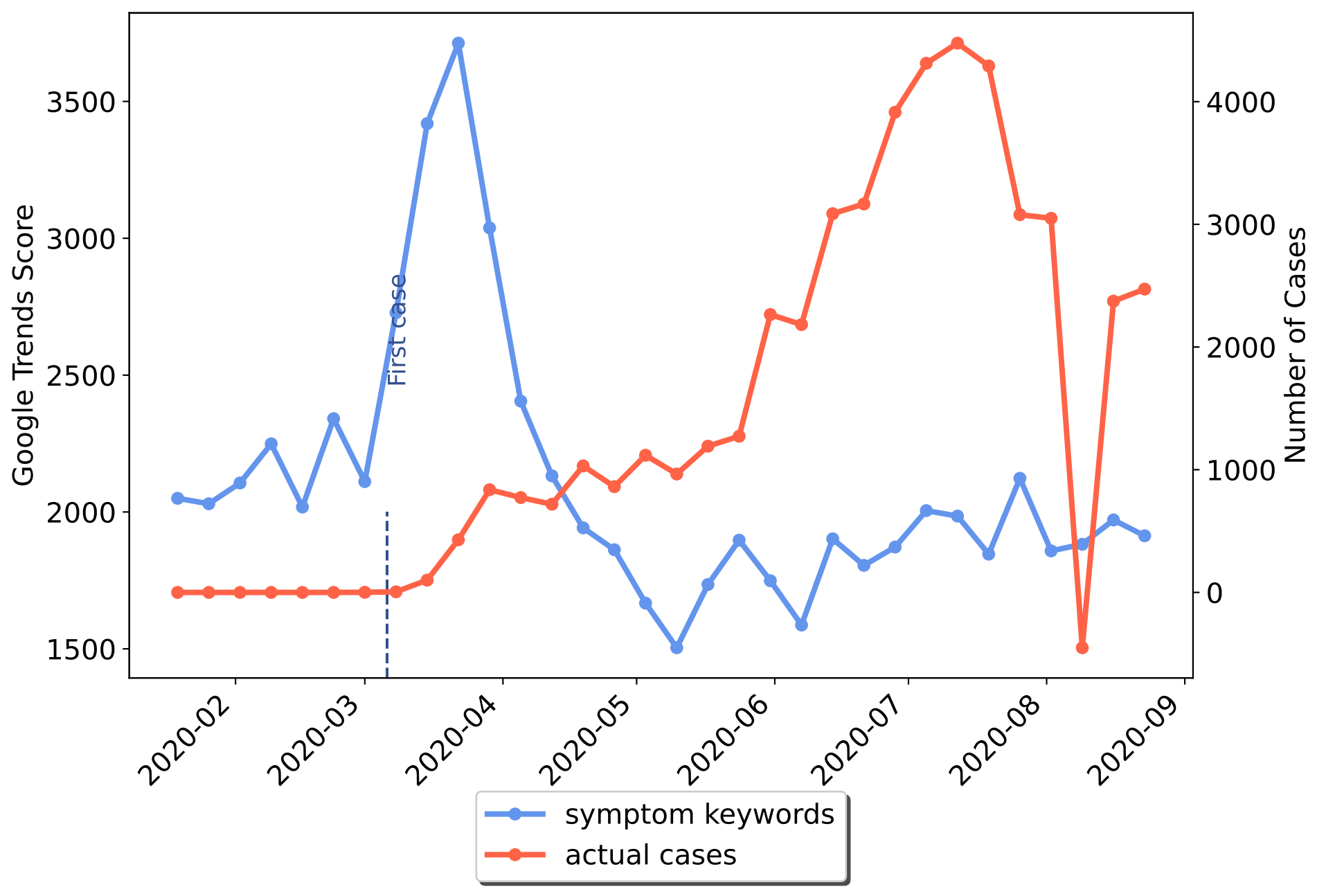

Supplement: Supplementary file 2 [file Data_Sheet_1.ZIP › figures/Utah_totalsymptom_GT-eps-converted-to.pdf]

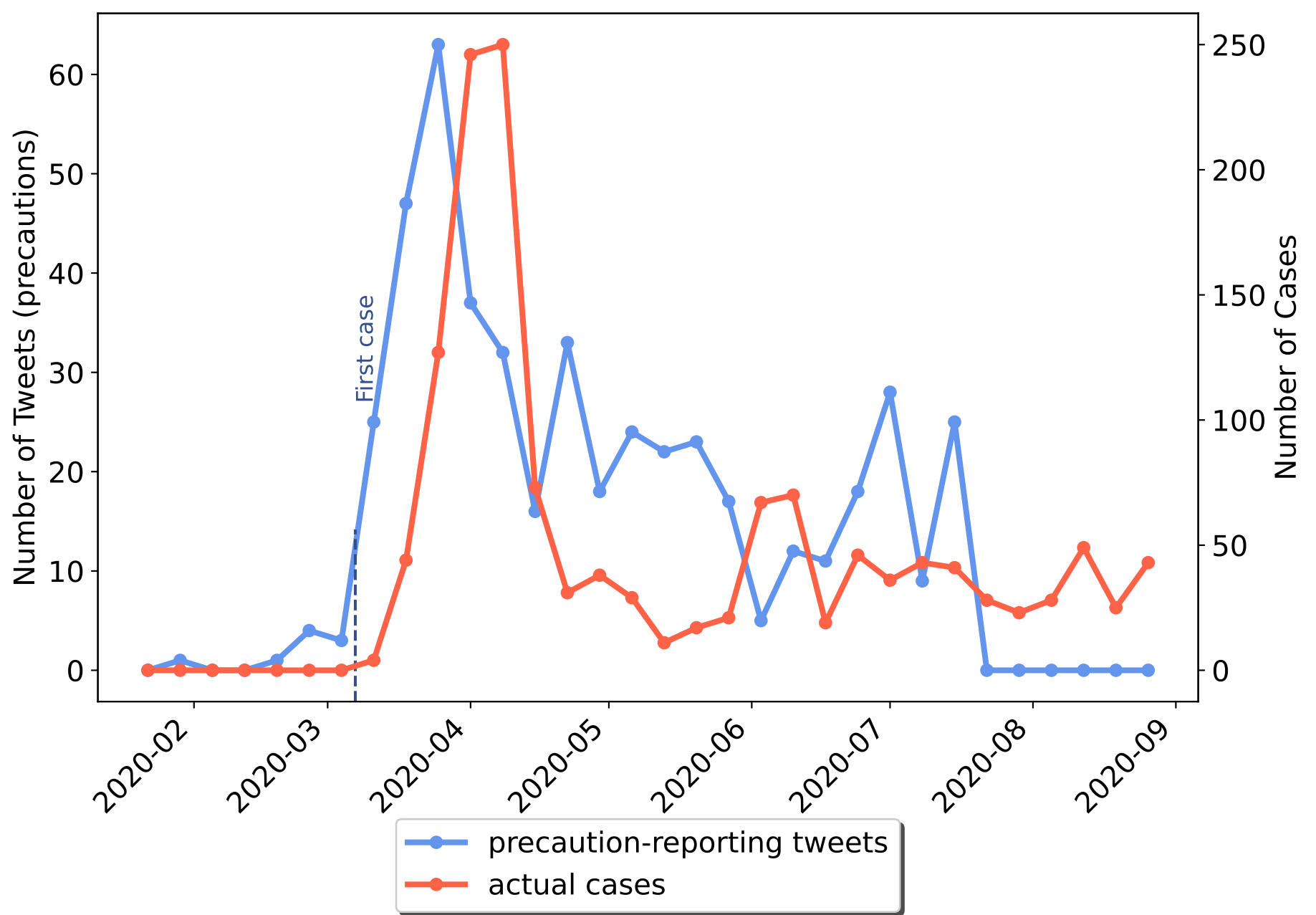

Supplement: Supplementary file 2 [file Data_Sheet_1.ZIP › figures/Vermont_precaution_twitter-eps-converted-to.pdf]

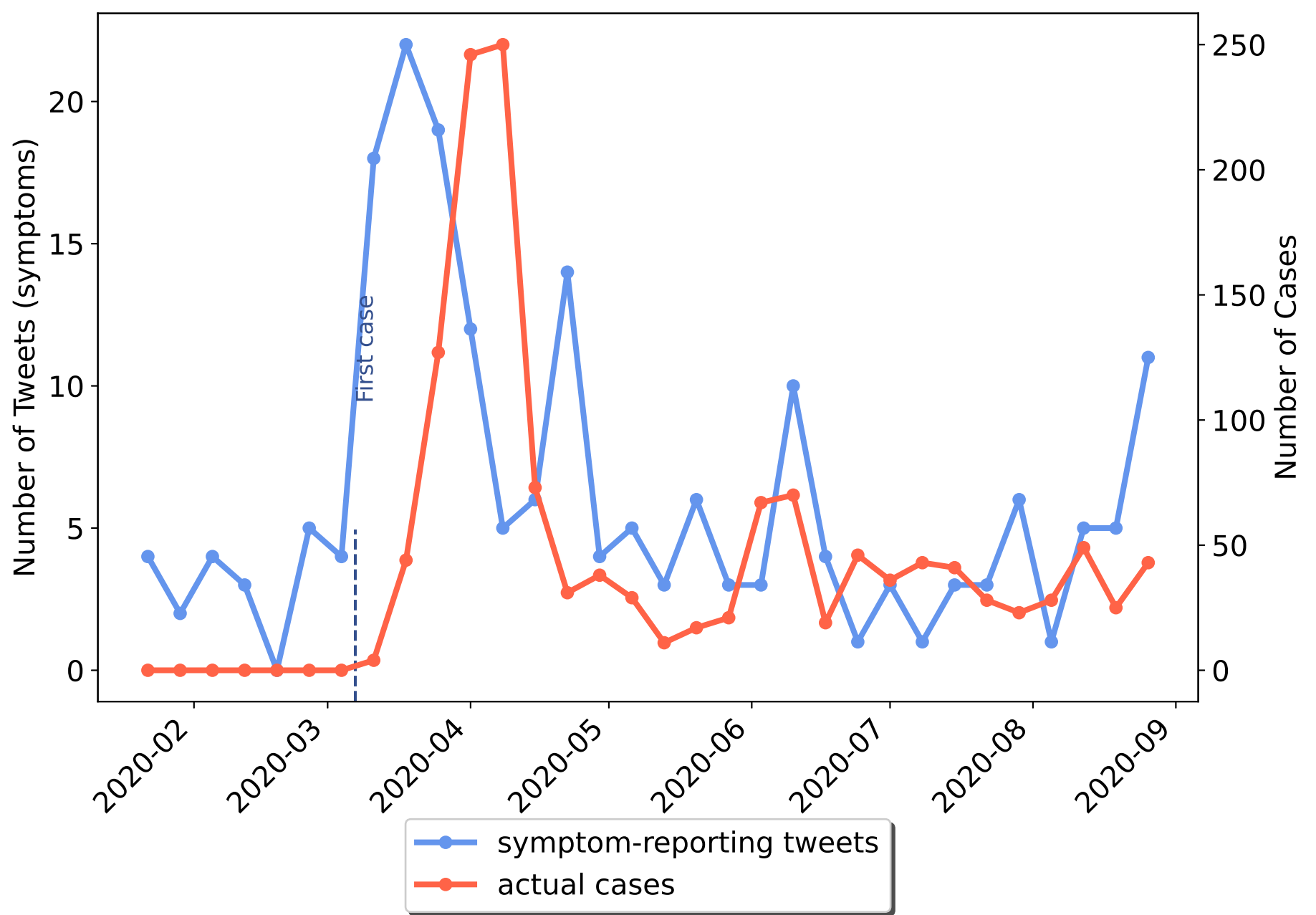

Supplement: Supplementary file 2 [file Data_Sheet_1.ZIP › figures/Vermont_symptom_twitter-eps-converted-to.pdf]

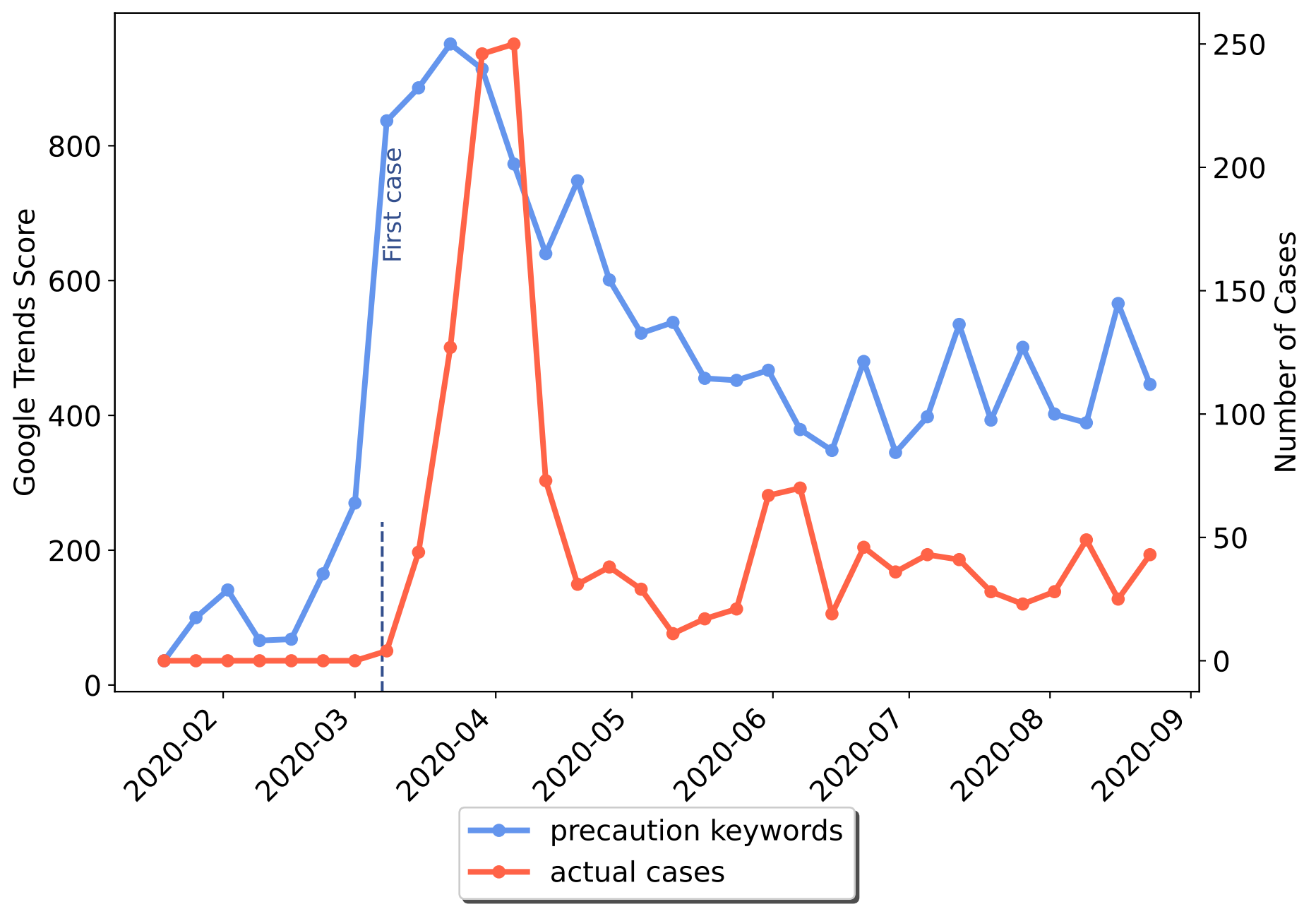

Supplement: Supplementary file 2 [file Data_Sheet_1.ZIP › figures/Vermont_totalprecaution_GT-eps-converted-to.pdf]

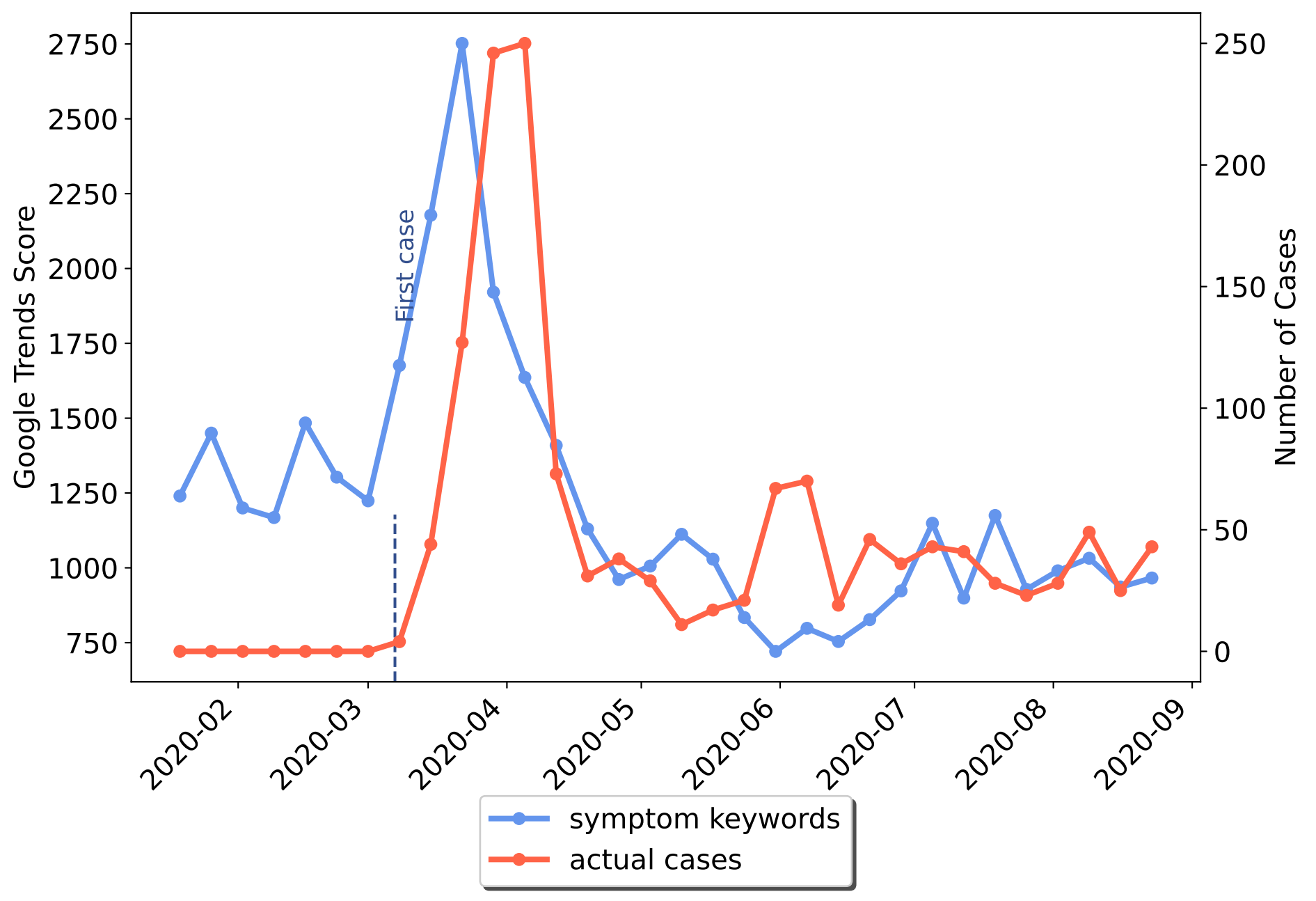

Supplement: Supplementary file 2 [file Data_Sheet_1.ZIP › figures/Vermont_totalsymptom_GT-eps-converted-to.pdf]

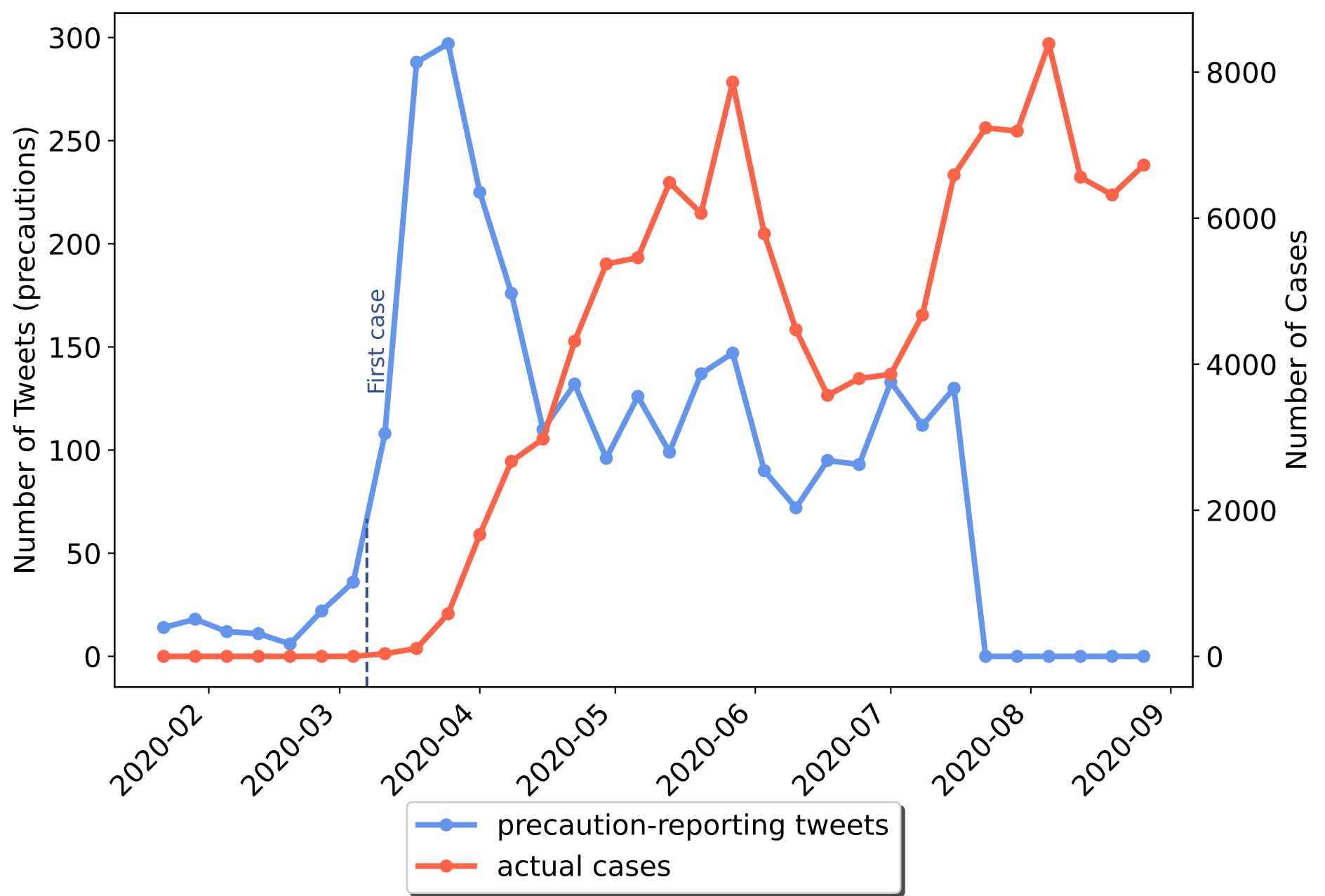

Supplement: Supplementary file 2 [file Data_Sheet_1.ZIP › figures/Virginia_precaution_twitter-eps-converted-to.pdf]

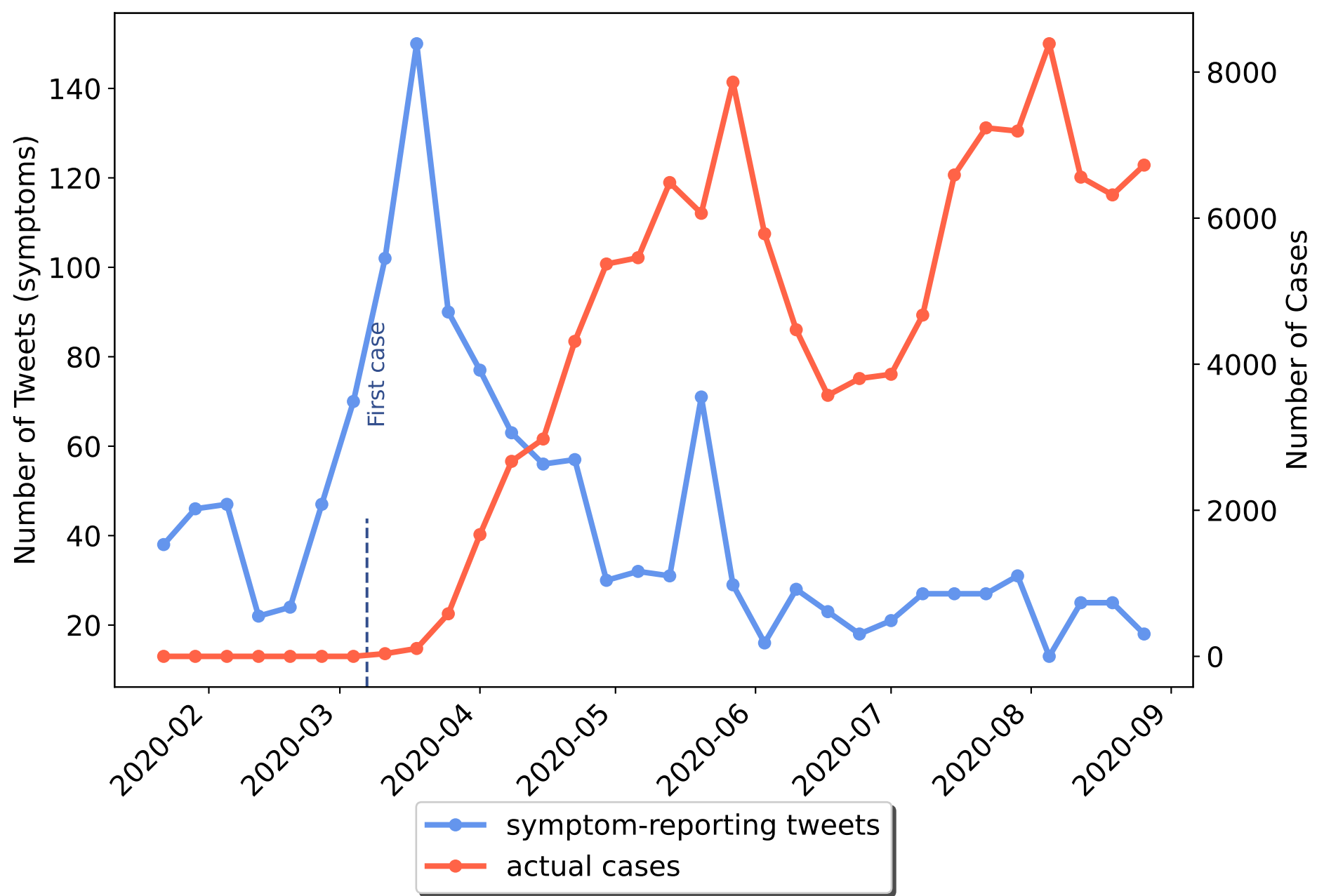

Supplement: Supplementary file 2 [file Data_Sheet_1.ZIP › figures/Virginia_symptom_twitter-eps-converted-to.pdf]
