# Supplementary figures and images for: Prediction of COVID-19 Waves Using Social Media and Google Search: A Case Study of the US and Canada (part 1 of 2)
Source: Front Public Health. 2021 Apr 16;9:656635. doi: 10.3389/fpubh.2021.656635 (PMC8085269; doi:10.3389/fpubh.2021.656635)

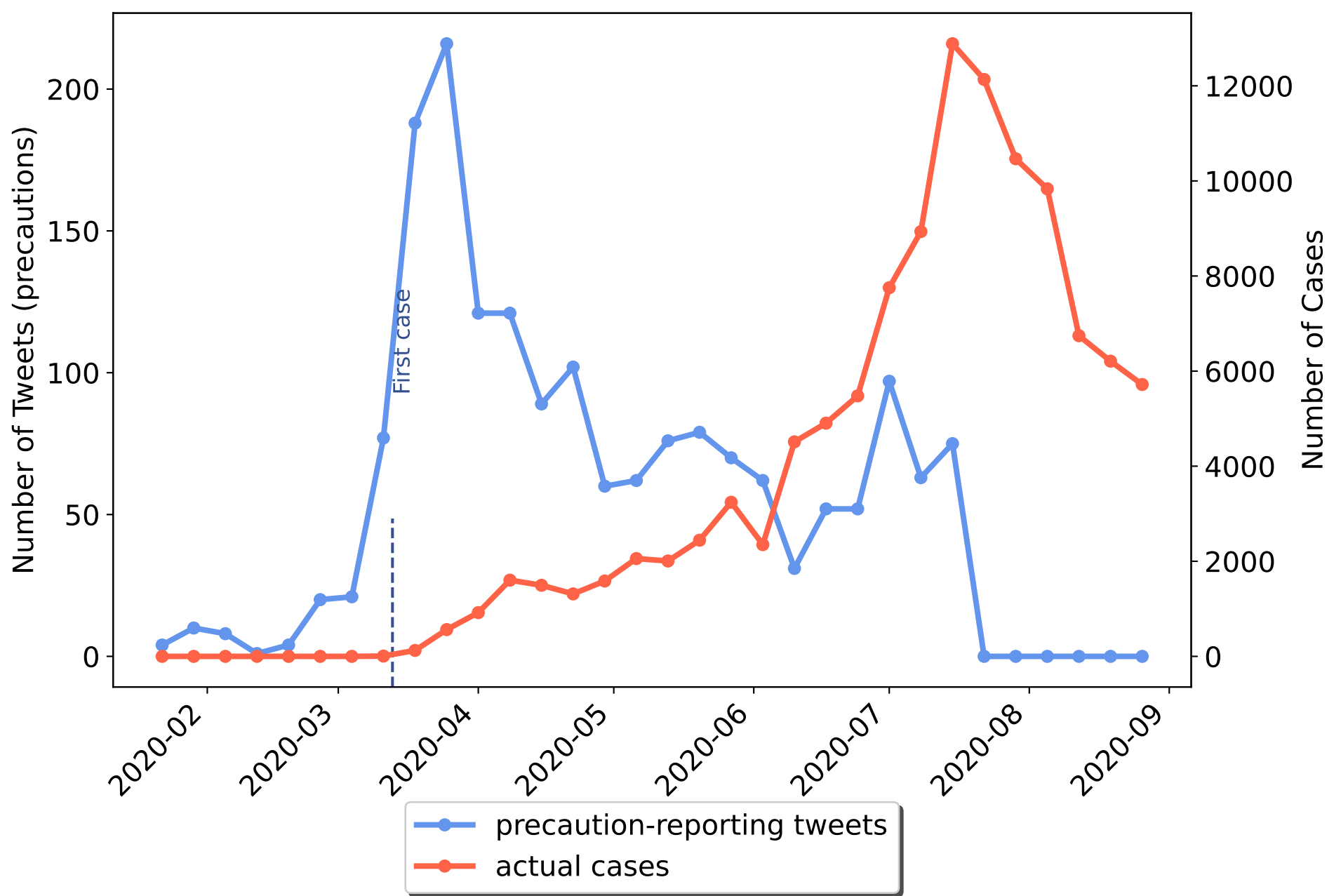

Supplement: Supplementary file 2 [file Data_Sheet_1.ZIP › figures/Alabama_precaution_twitter-eps-converted-to.pdf]

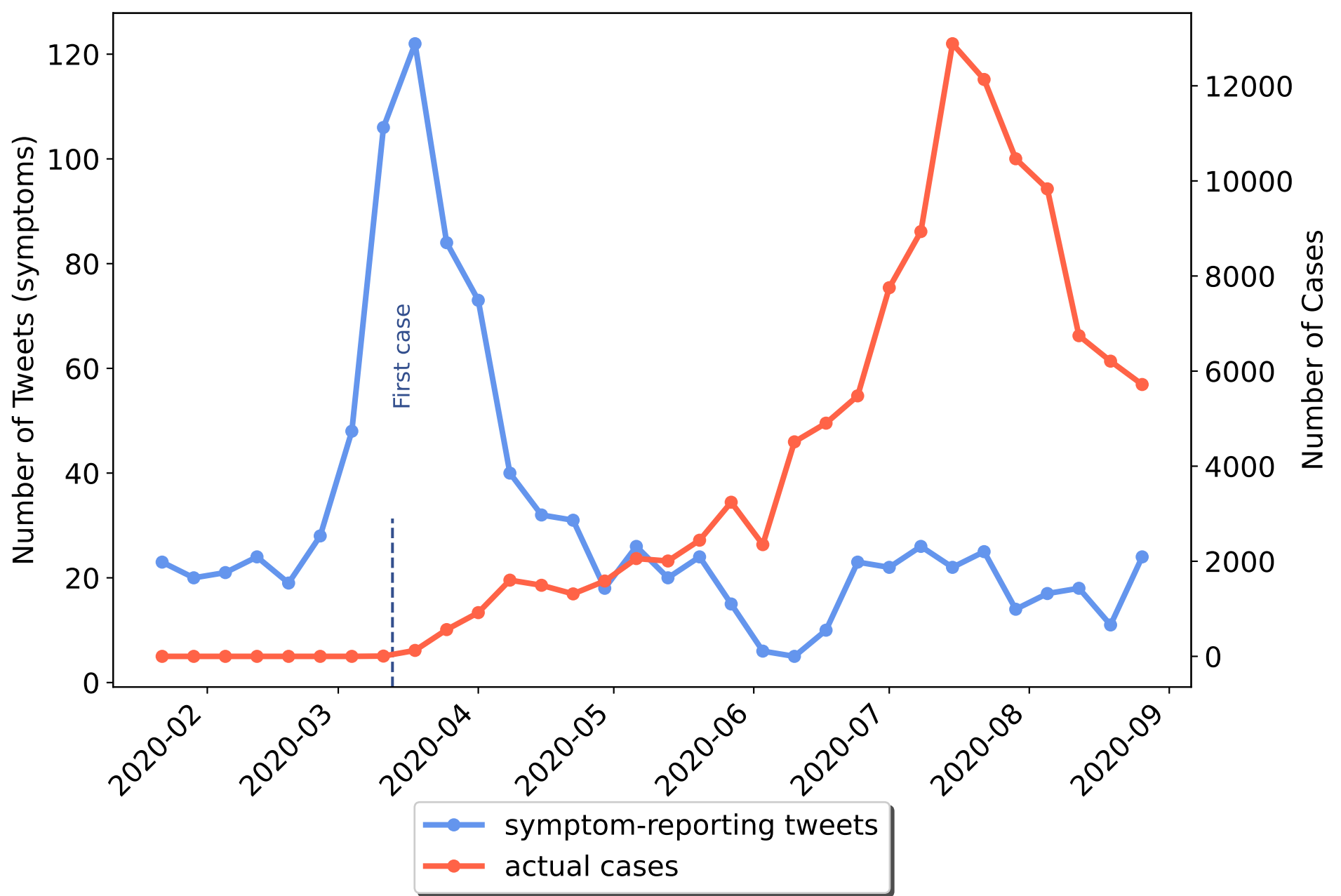

Supplement: Supplementary file 2 [file Data_Sheet_1.ZIP › figures/Alabama_symptom_twitter-eps-converted-to.pdf]

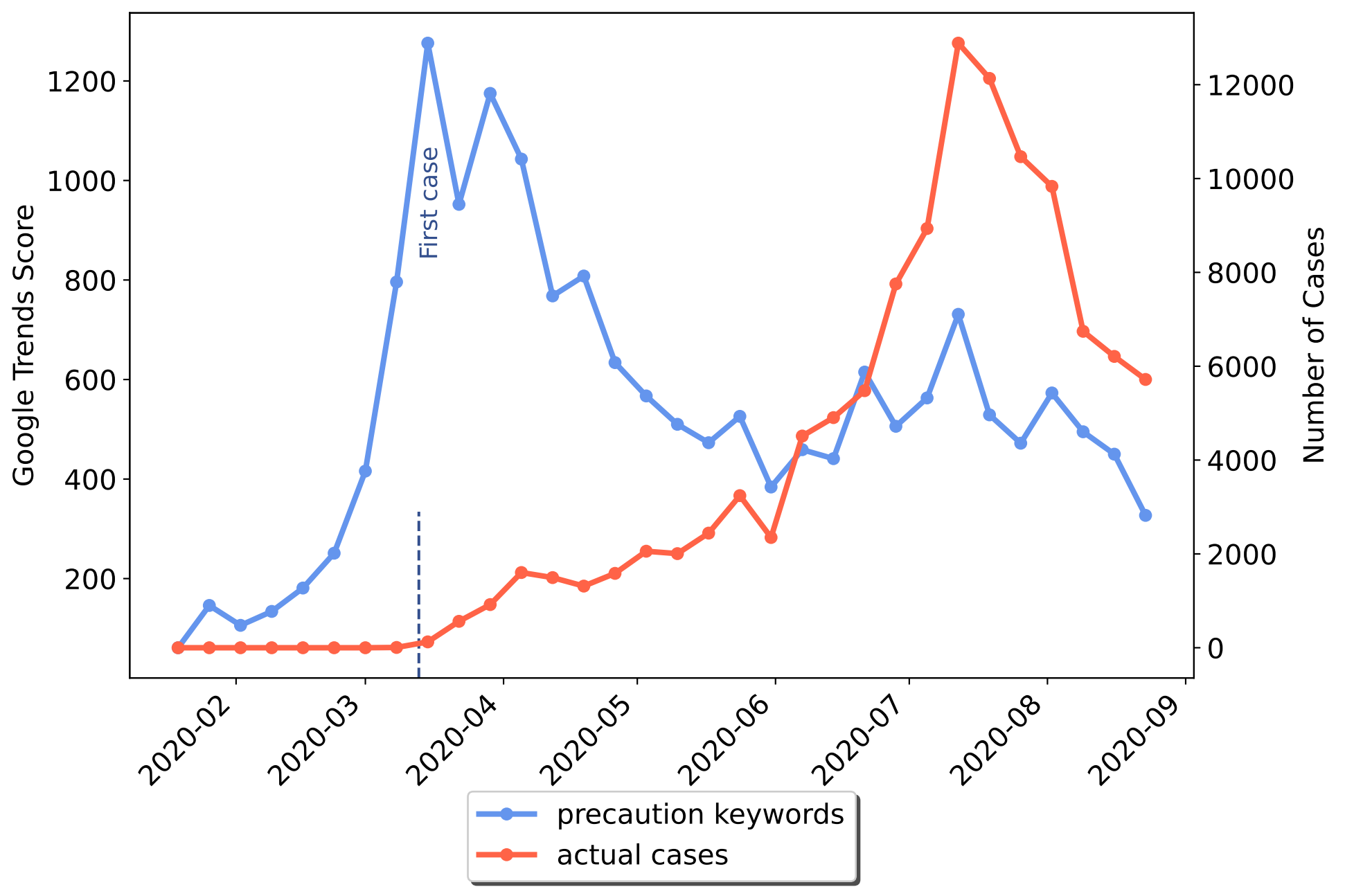

Supplement: Supplementary file 2 [file Data_Sheet_1.ZIP › figures/Alabama_totalprecaution_GT-eps-converted-to.pdf]

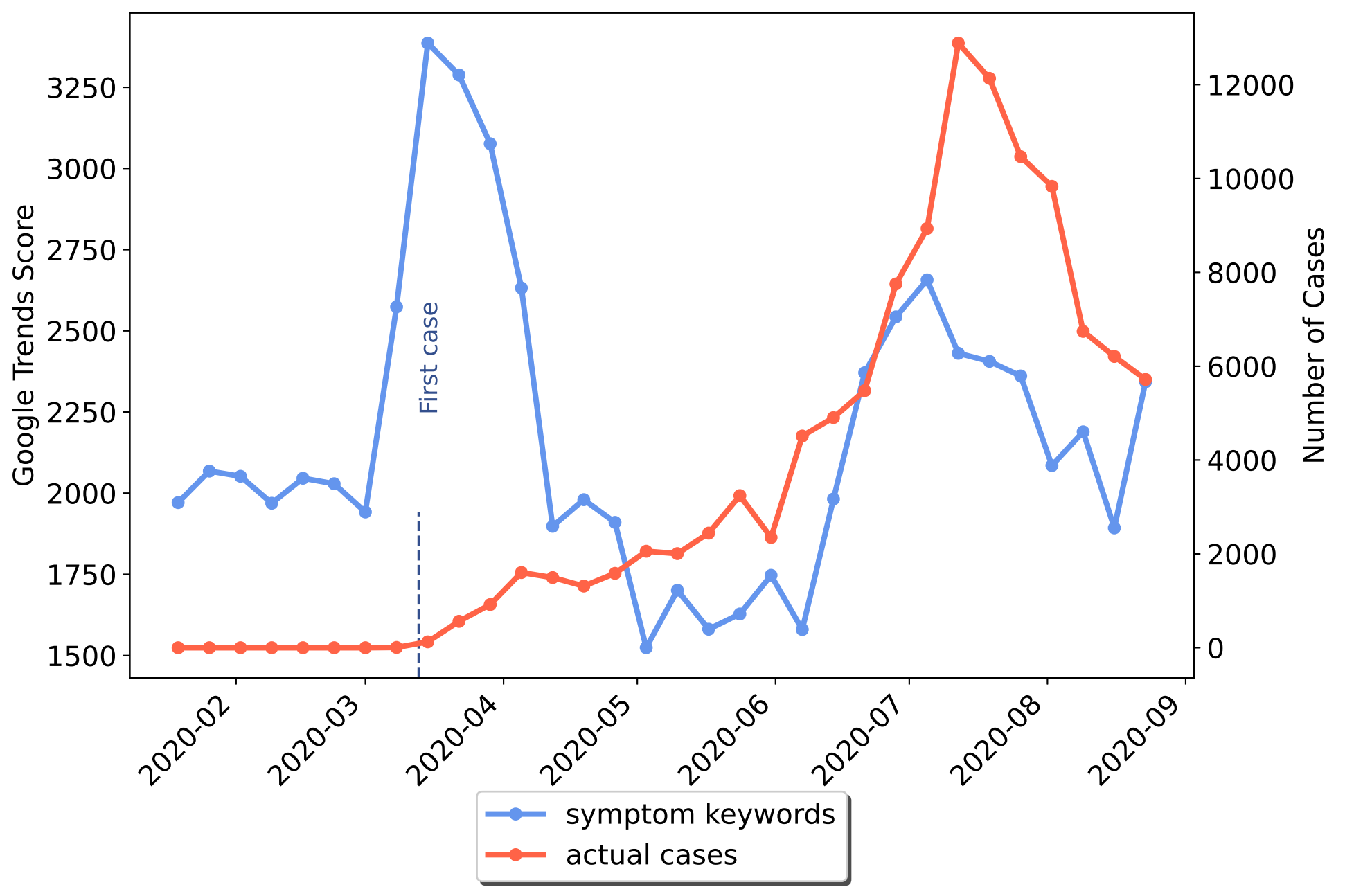

Supplement: Supplementary file 2 [file Data_Sheet_1.ZIP › figures/Alabama_totalsymptom_GT-eps-converted-to.pdf]

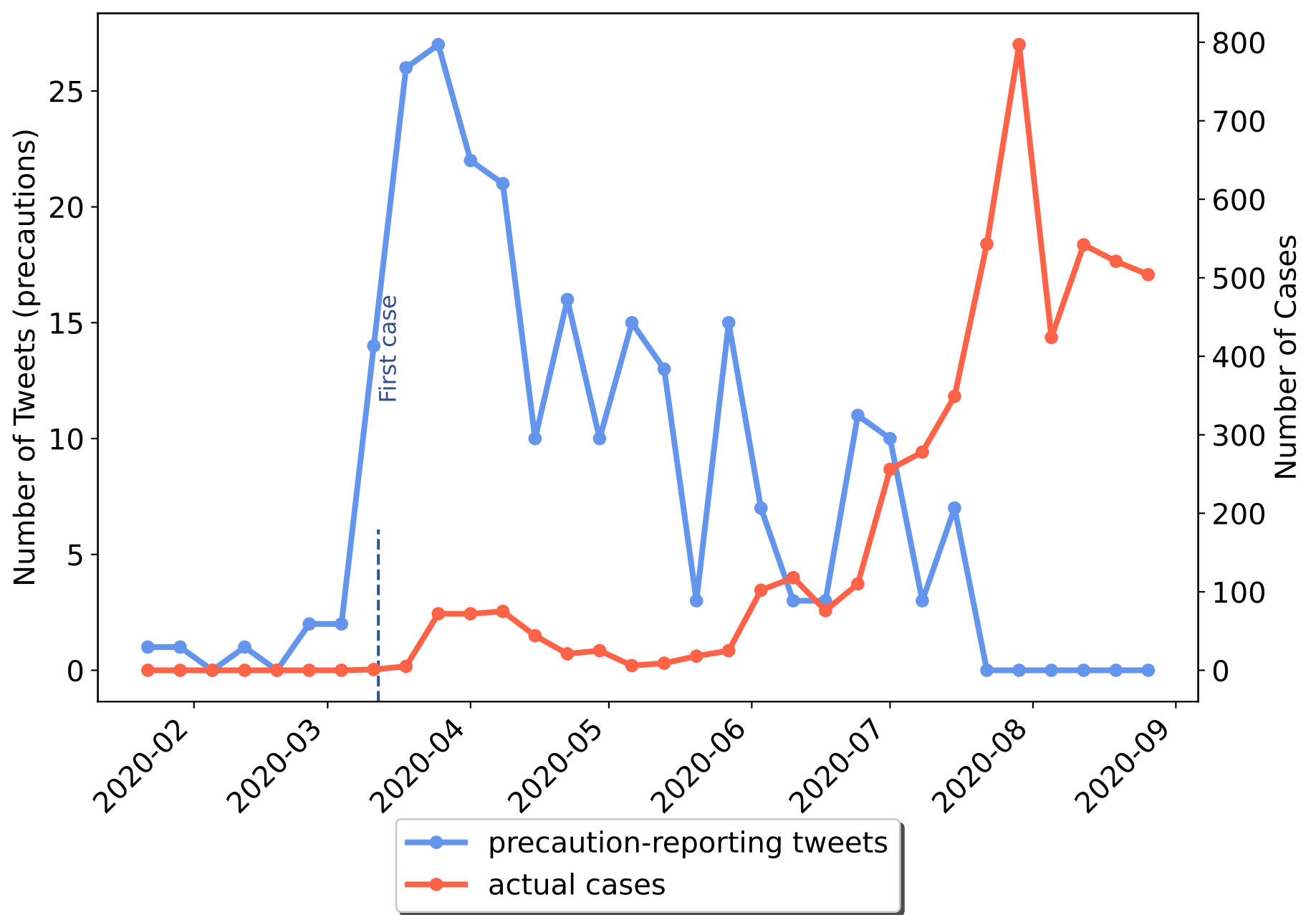

Supplement: Supplementary file 2 [file Data_Sheet_1.ZIP › figures/Alaska_precaution_twitter-eps-converted-to.pdf]

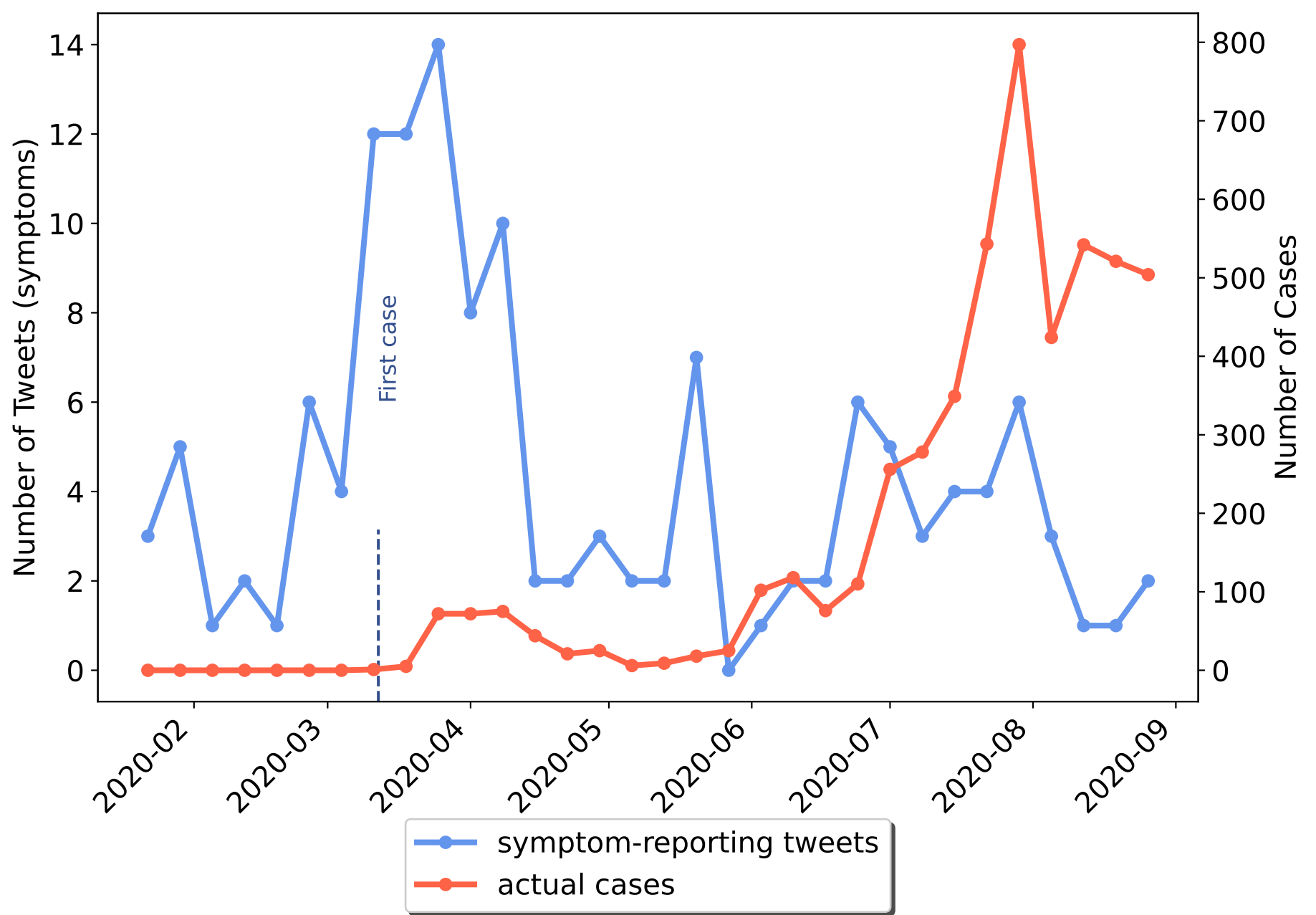

Supplement: Supplementary file 2 [file Data_Sheet_1.ZIP › figures/Alaska_symptom_twitter-eps-converted-to.pdf]

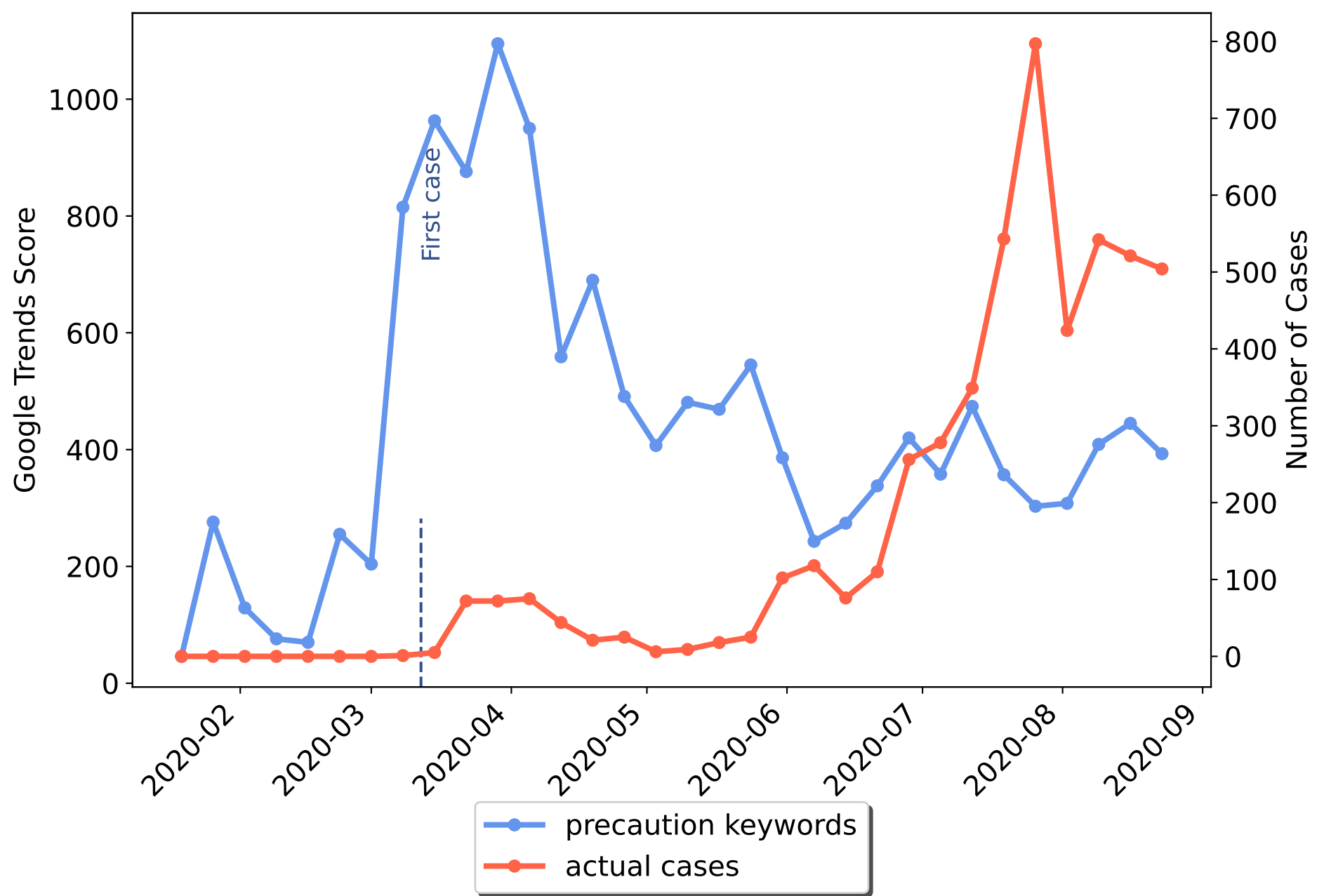

Supplement: Supplementary file 2 [file Data_Sheet_1.ZIP › figures/Alaska_totalprecaution_GT-eps-converted-to.pdf]

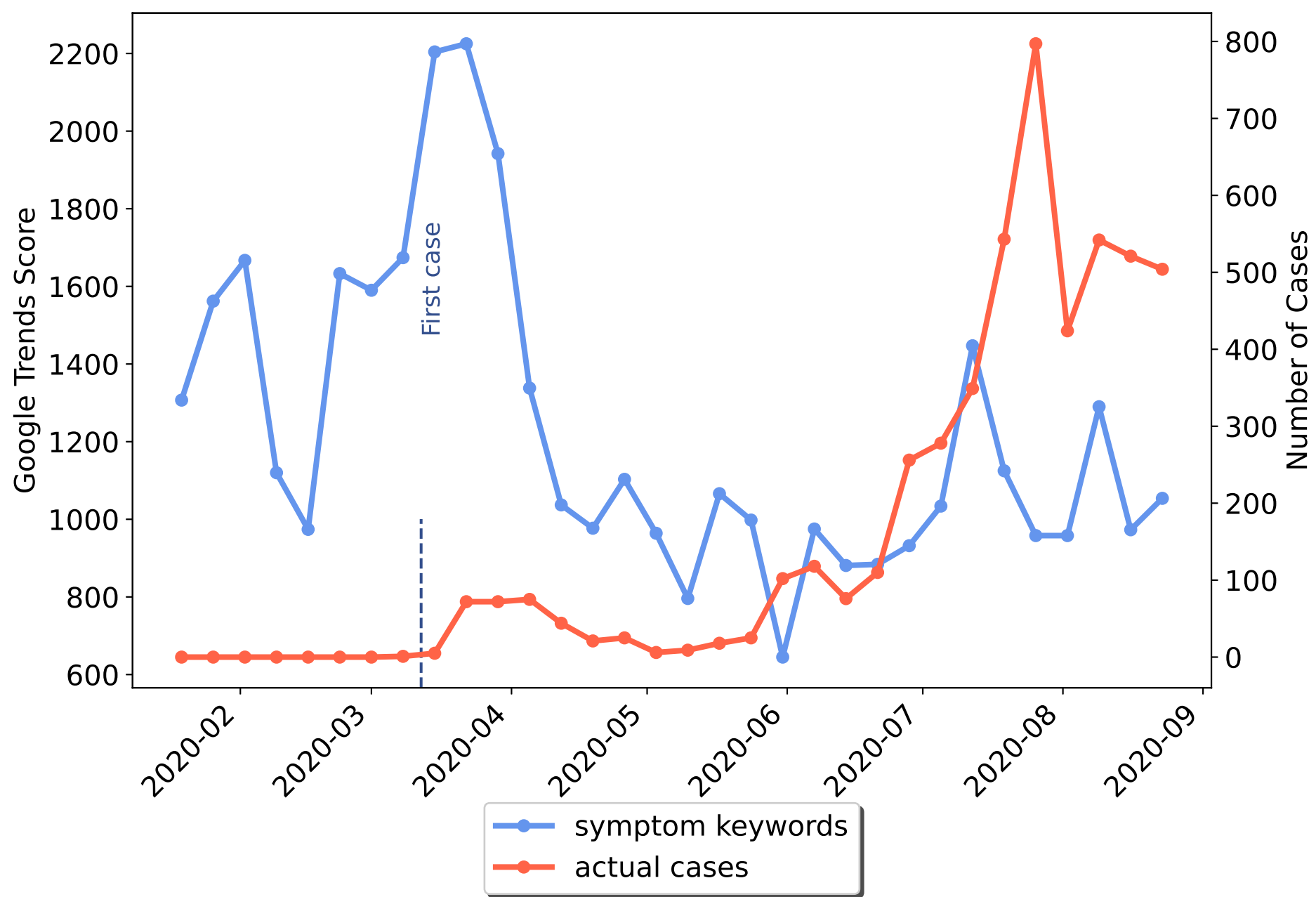

Supplement: Supplementary file 2 [file Data_Sheet_1.ZIP › figures/Alaska_totalsymptom_GT-eps-converted-to.pdf]

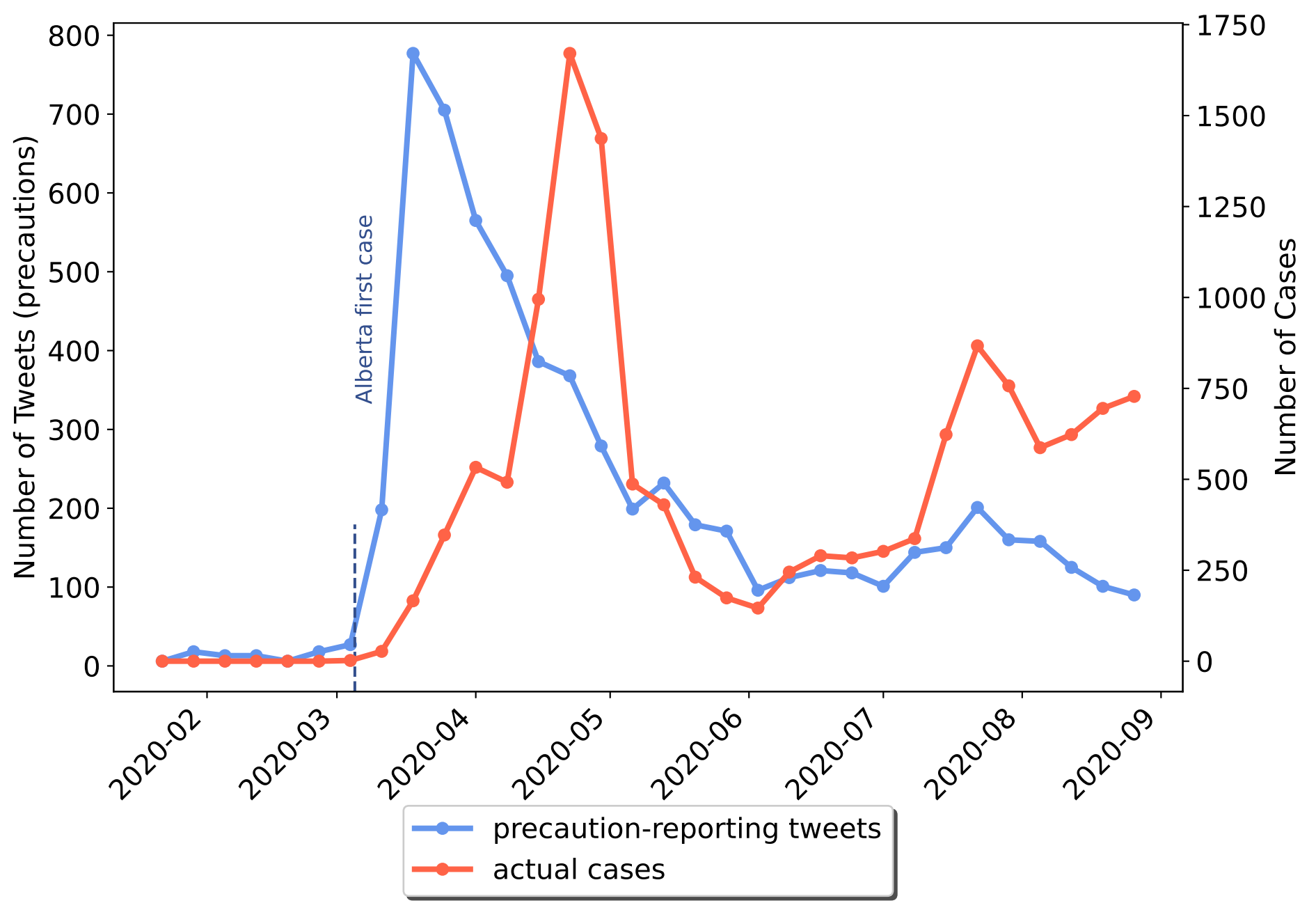

Supplement: Supplementary file 2 [file Data_Sheet_1.ZIP › figures/Alberta_precaution_twitter-eps-converted-to.pdf]

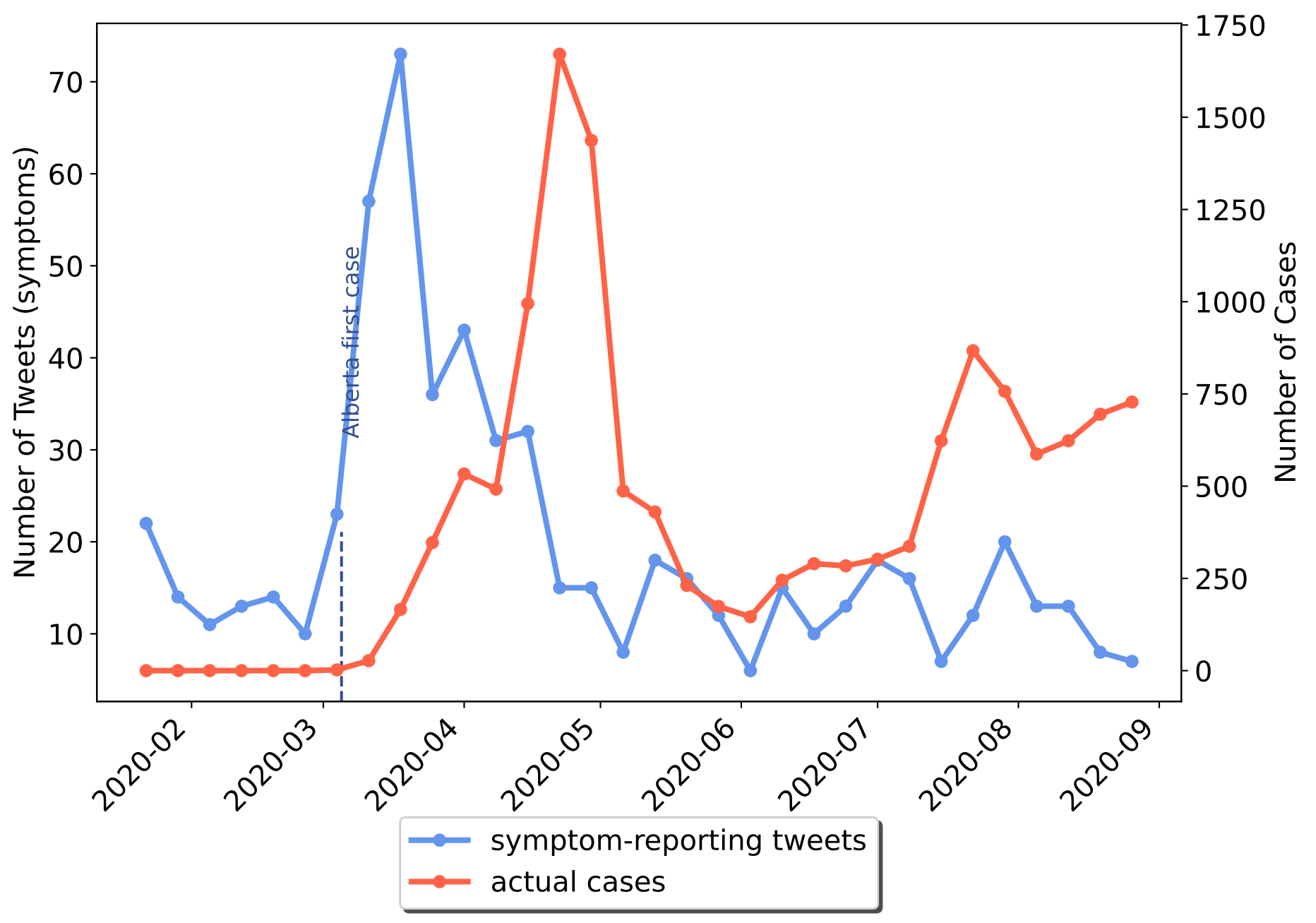

Supplement: Supplementary file 2 [file Data_Sheet_1.ZIP › figures/Alberta_symptom_twitter-eps-converted-to.pdf]

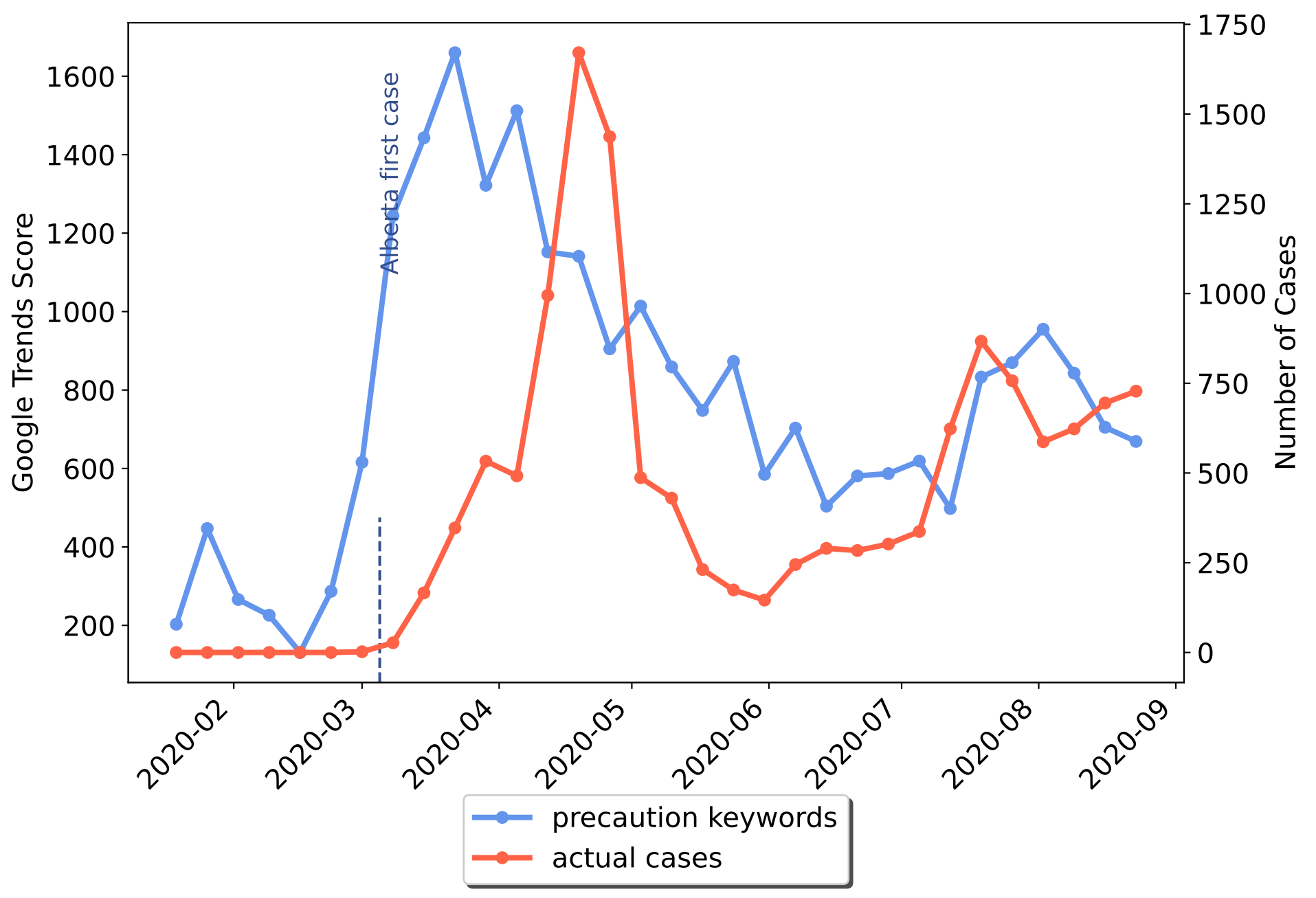

Supplement: Supplementary file 2 [file Data_Sheet_1.ZIP › figures/Alberta_totalprecaution_GT-eps-converted-to.pdf]

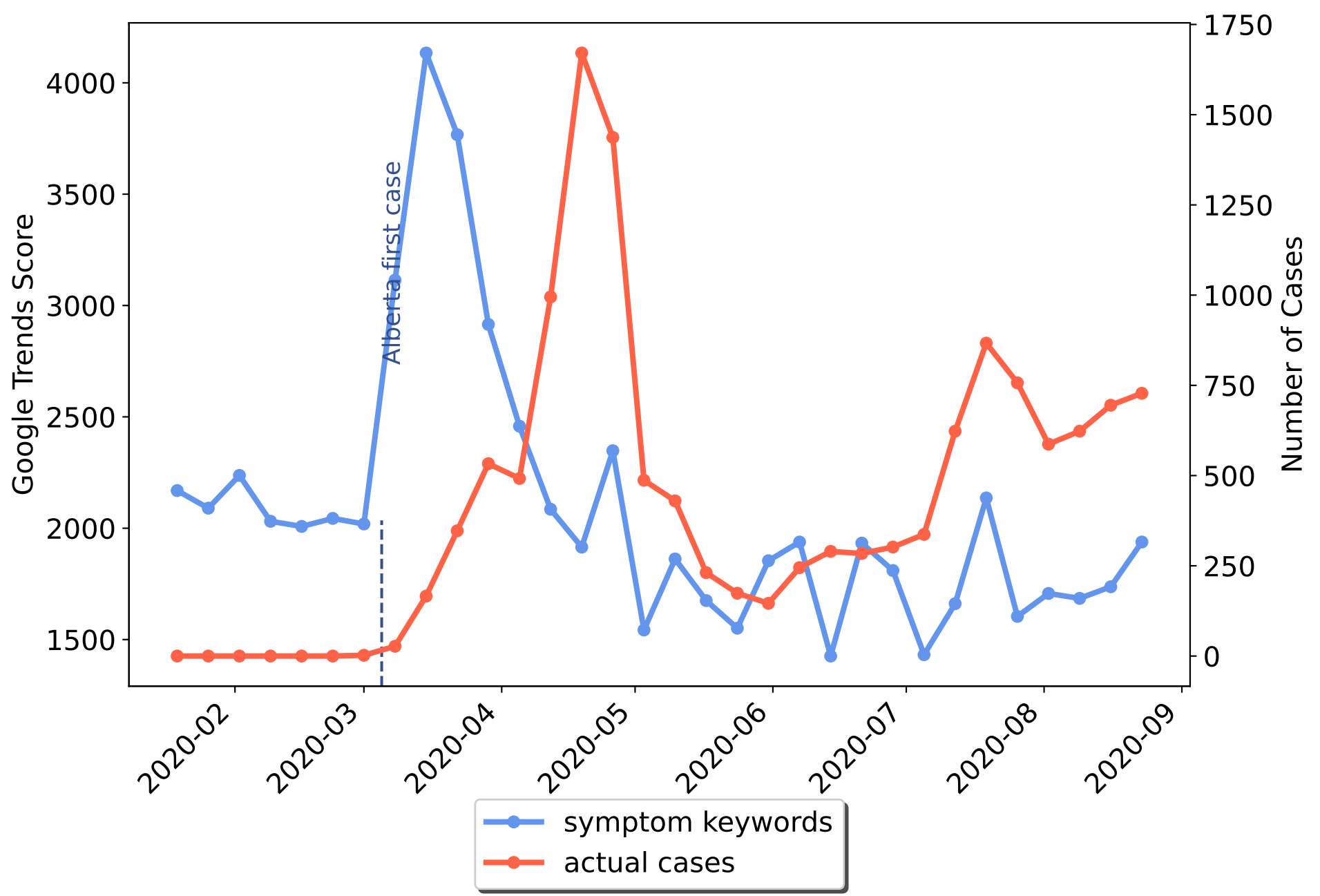

Supplement: Supplementary file 2 [file Data_Sheet_1.ZIP › figures/Alberta_totalsymptom_GT-eps-converted-to.pdf]

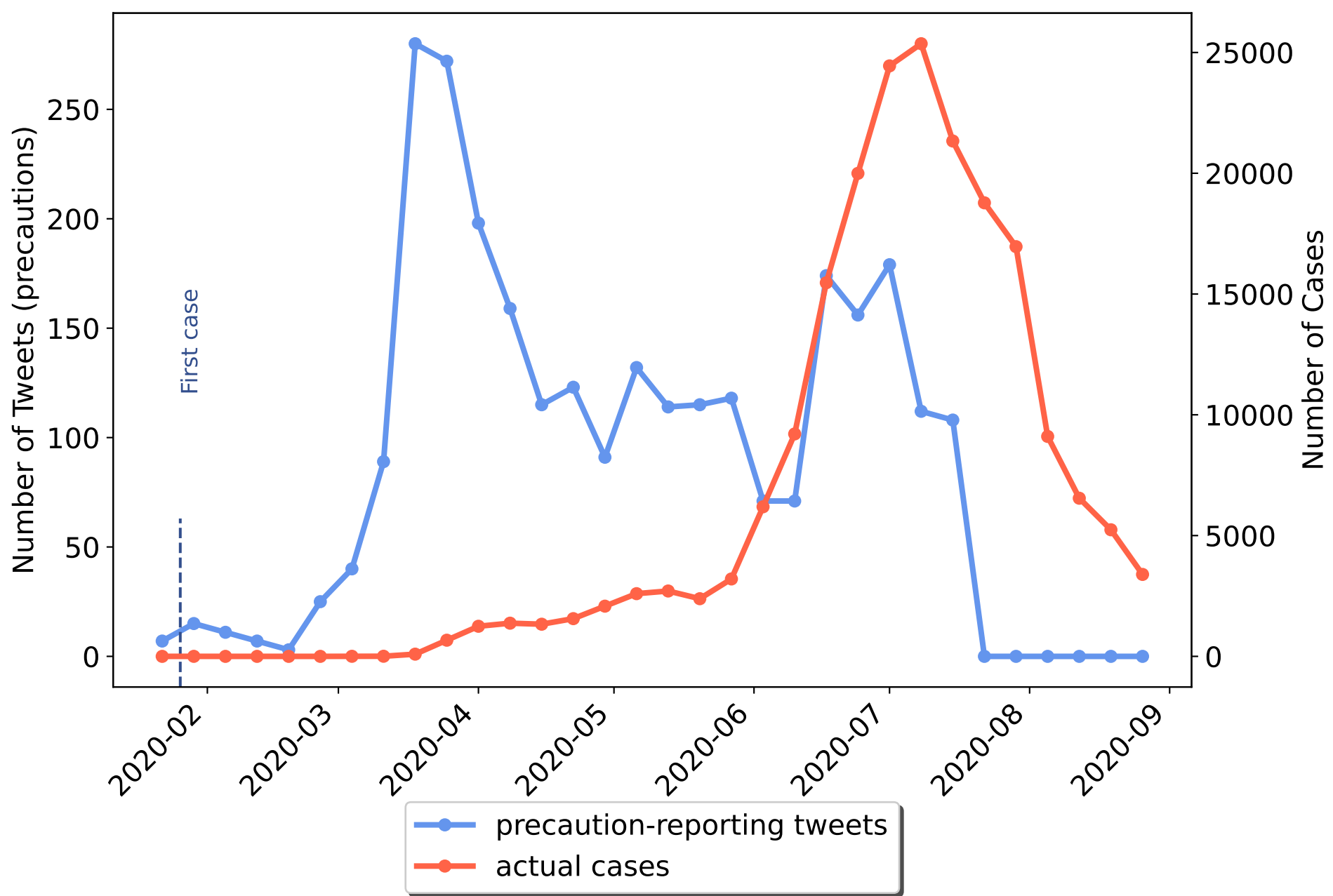

Supplement: Supplementary file 2 [file Data_Sheet_1.ZIP › figures/Arizona_precaution_twitter-eps-converted-to.pdf]

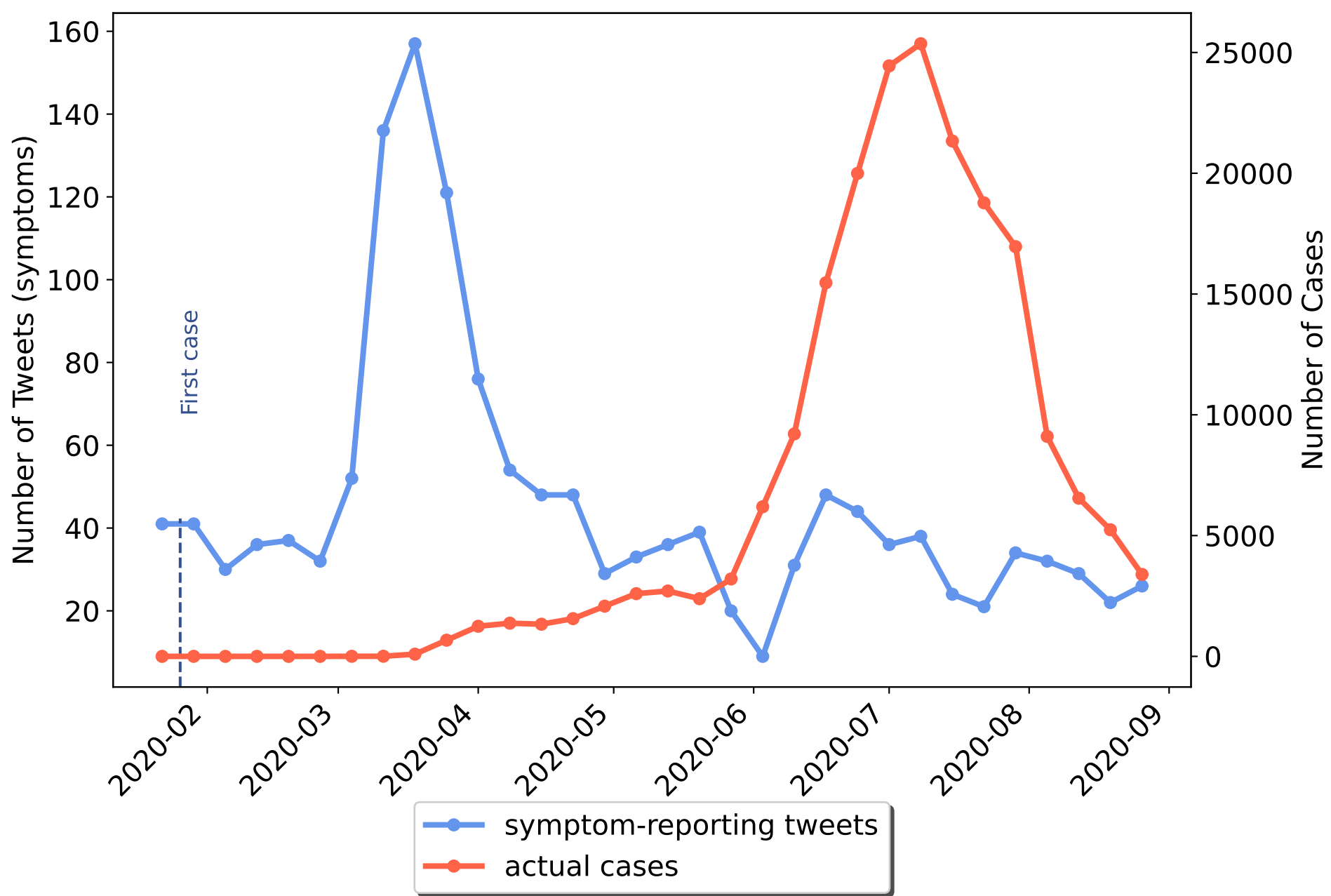

Supplement: Supplementary file 2 [file Data_Sheet_1.ZIP › figures/Arizona_symptom_twitter-eps-converted-to.pdf]

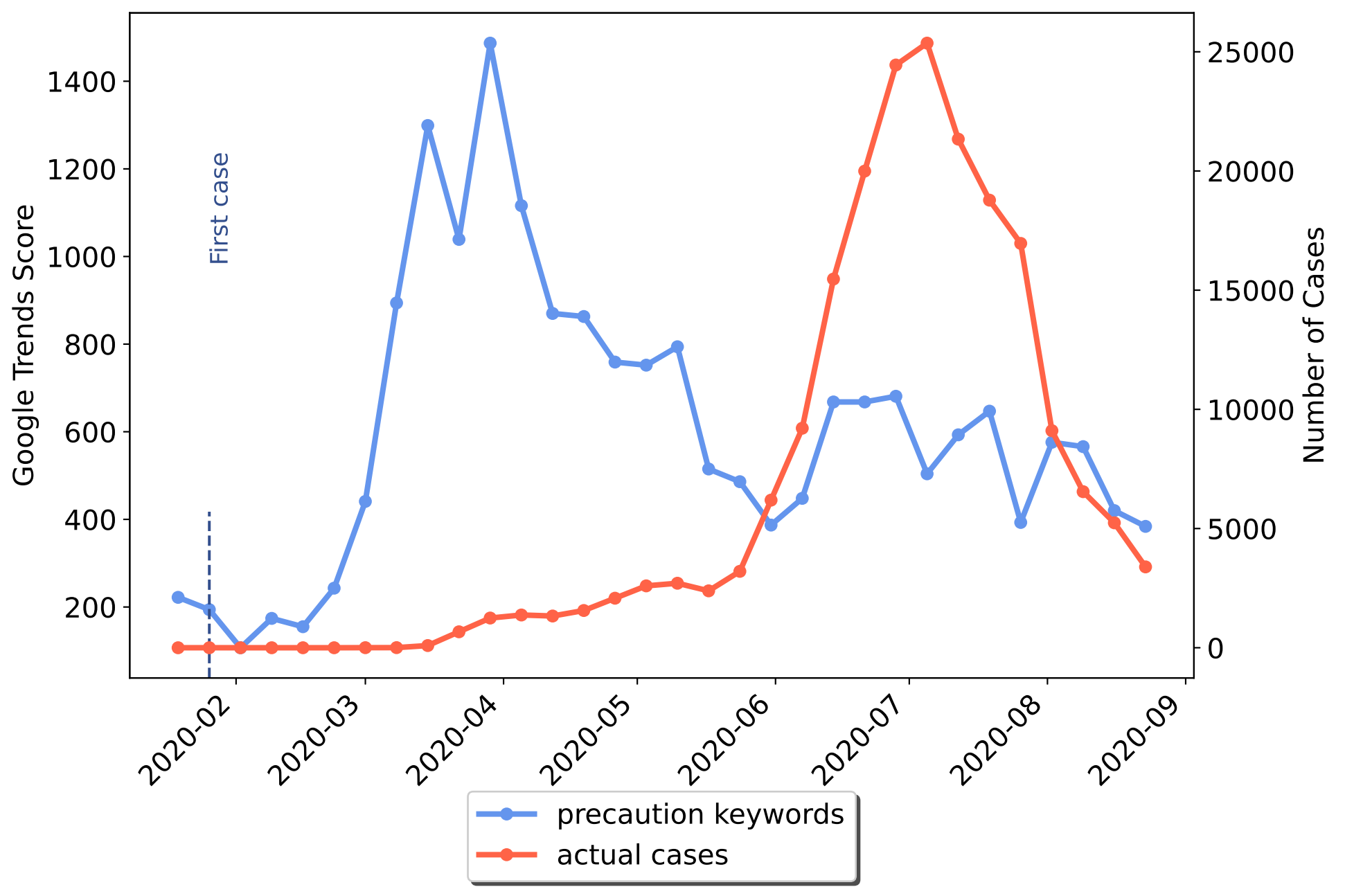

Supplement: Supplementary file 2 [file Data_Sheet_1.ZIP › figures/Arizona_totalprecaution_GT-eps-converted-to.pdf]

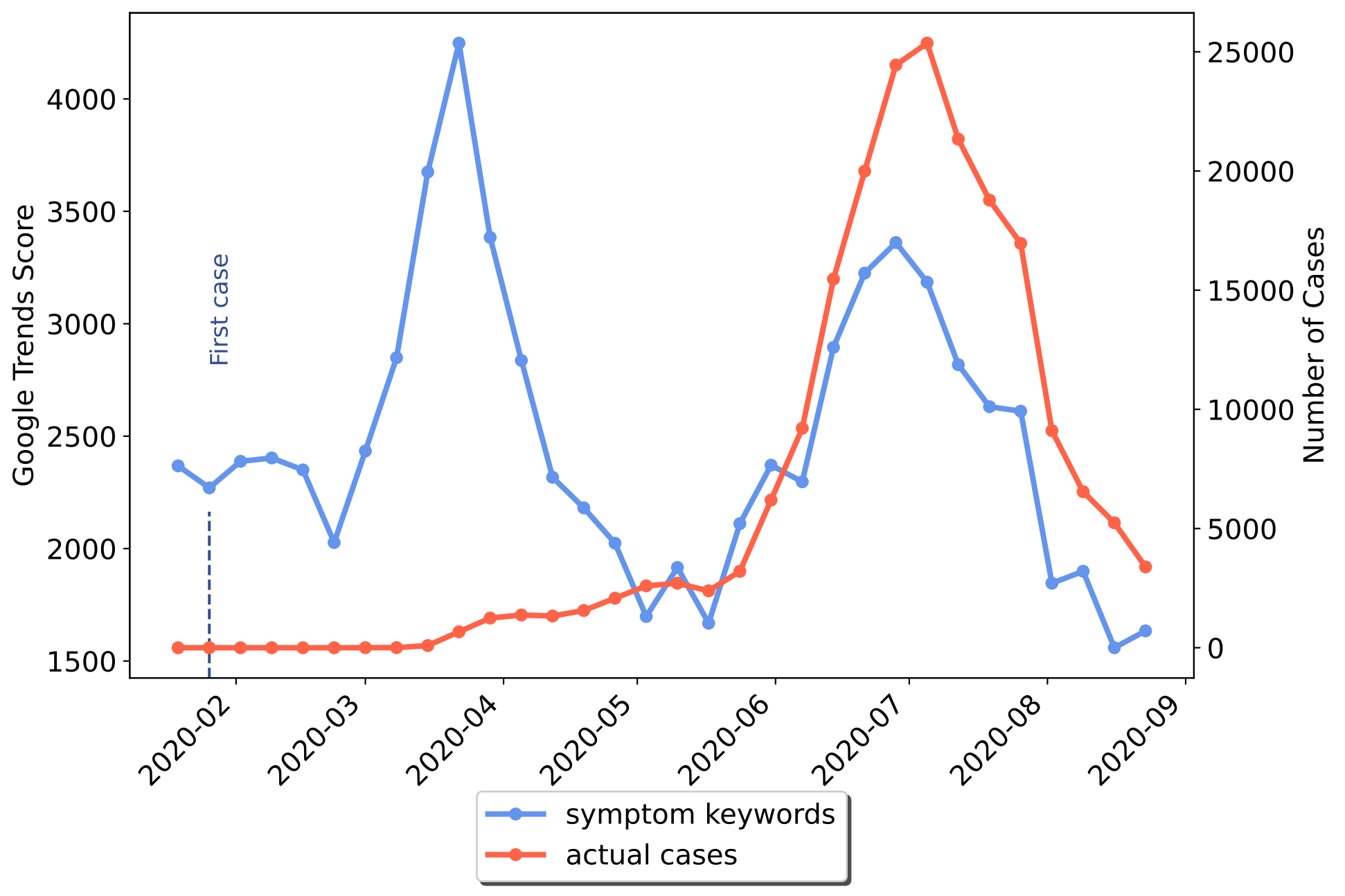

Supplement: Supplementary file 2 [file Data_Sheet_1.ZIP › figures/Arizona_totalsymptom_GT-eps-converted-to.pdf]

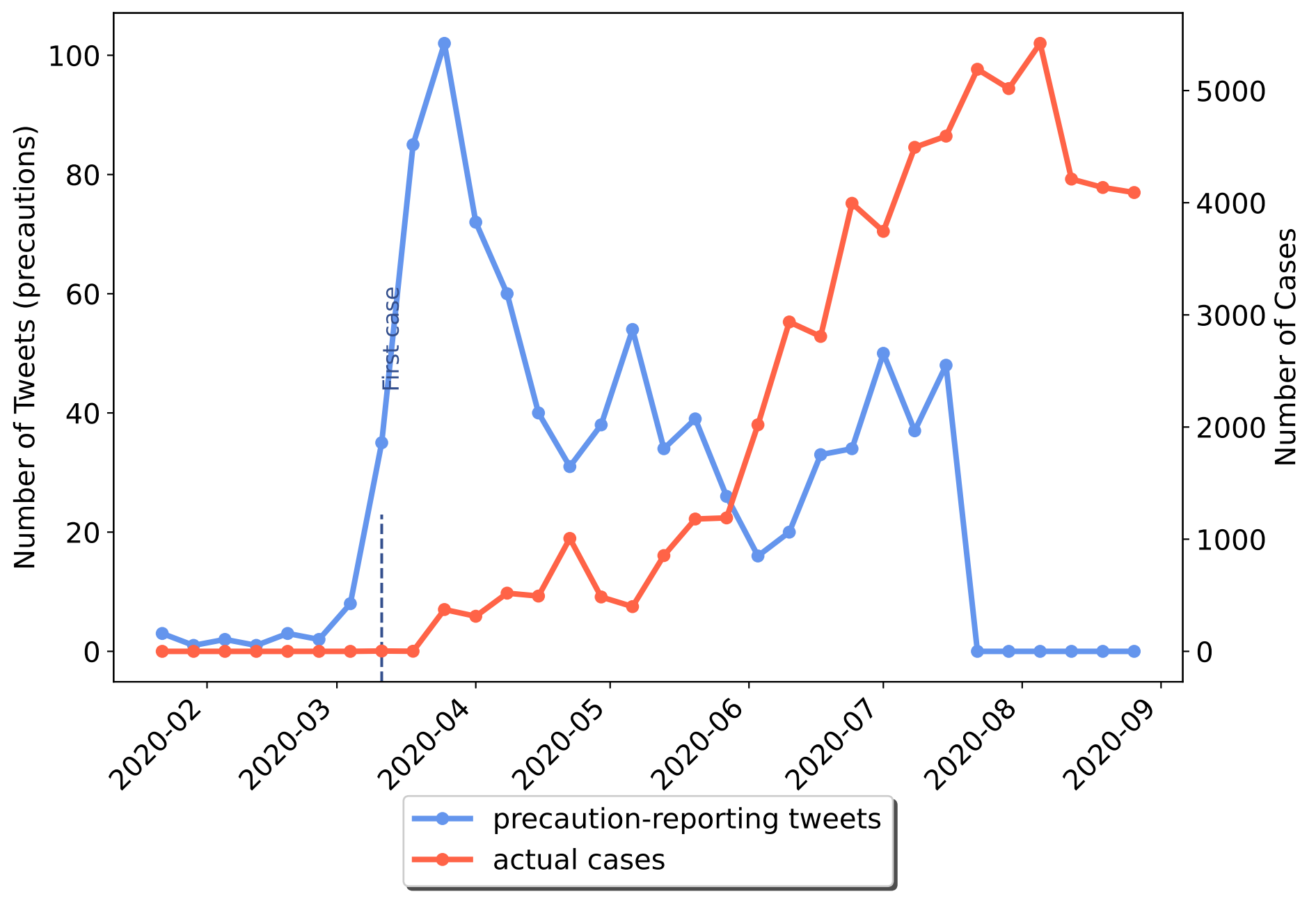

Supplement: Supplementary file 2 [file Data_Sheet_1.ZIP › figures/Arkansas_precaution_twitter-eps-converted-to.pdf]

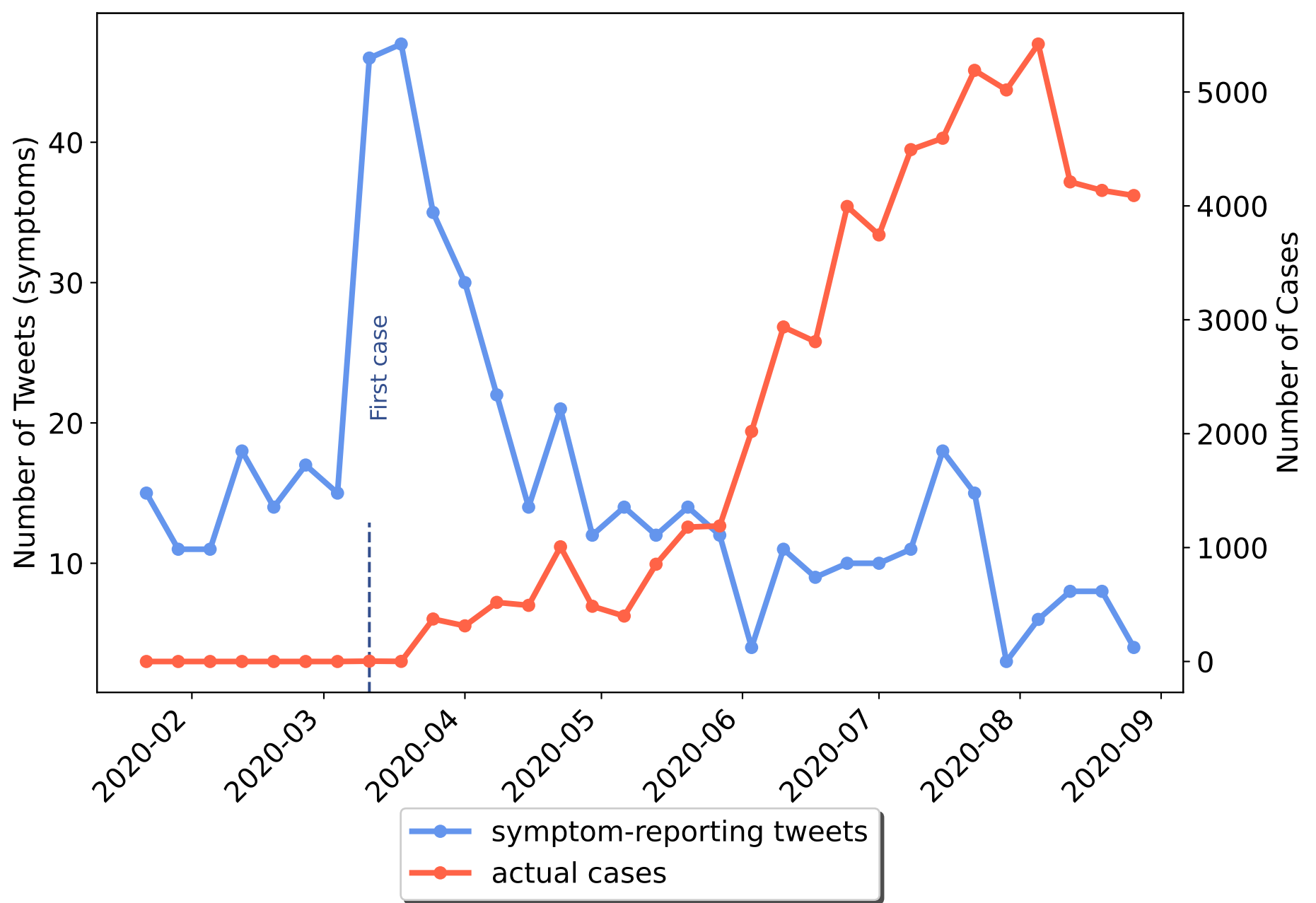

Supplement: Supplementary file 2 [file Data_Sheet_1.ZIP › figures/Arkansas_symptom_twitter-eps-converted-to.pdf]

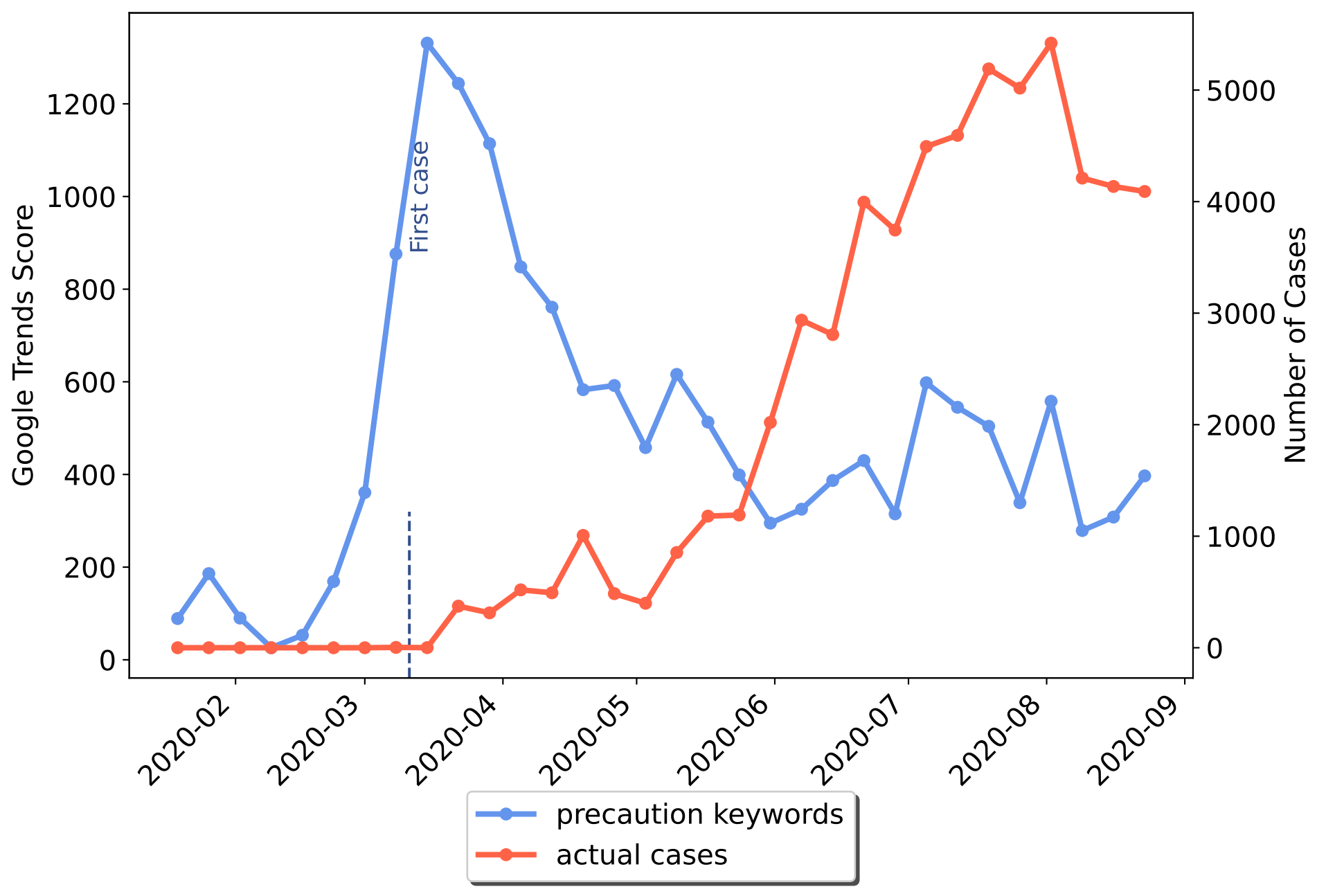

Supplement: Supplementary file 2 [file Data_Sheet_1.ZIP › figures/Arkansas_totalprecaution_GT-eps-converted-to.pdf]

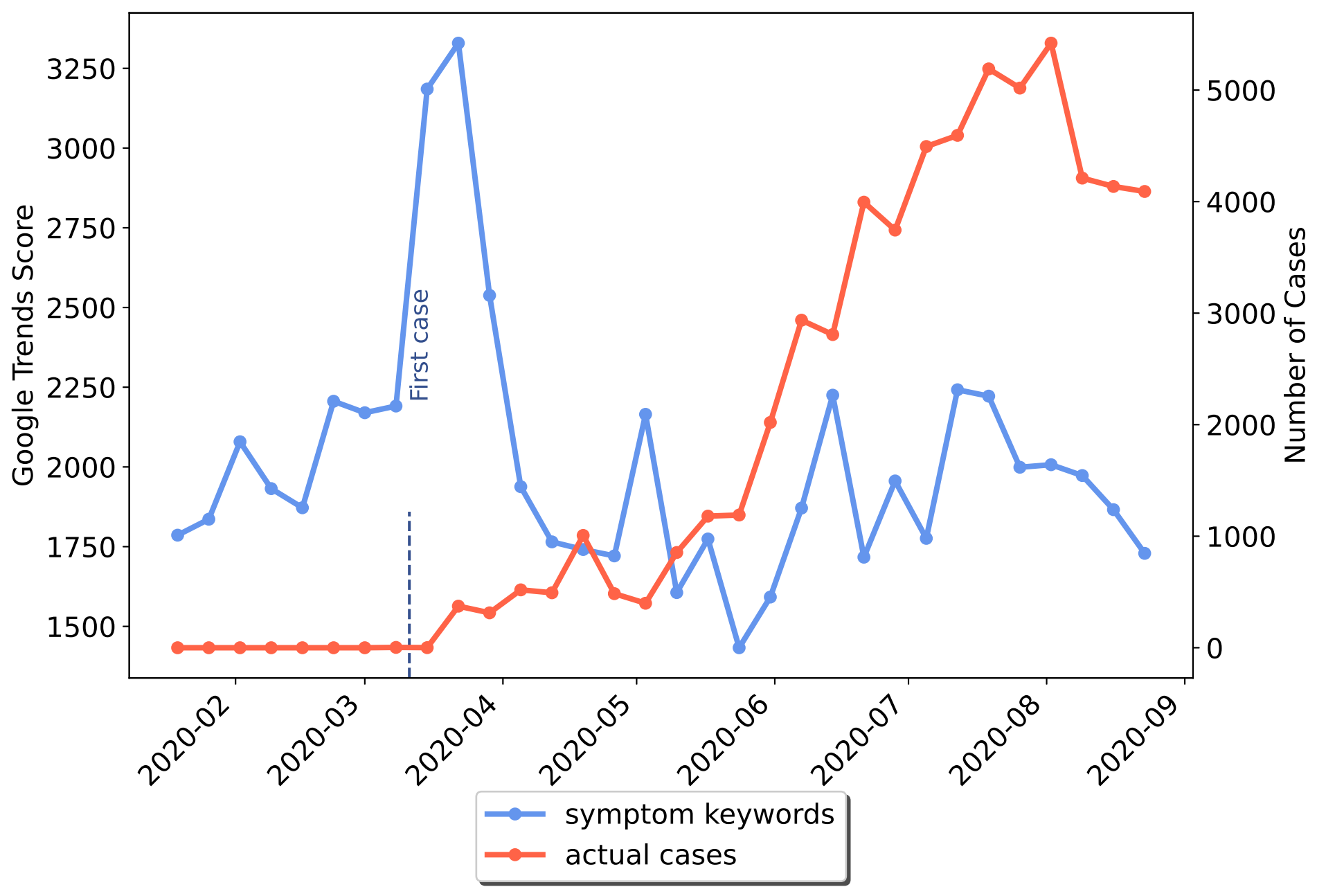

Supplement: Supplementary file 2 [file Data_Sheet_1.ZIP › figures/Arkansas_totalsymptom_GT-eps-converted-to.pdf]

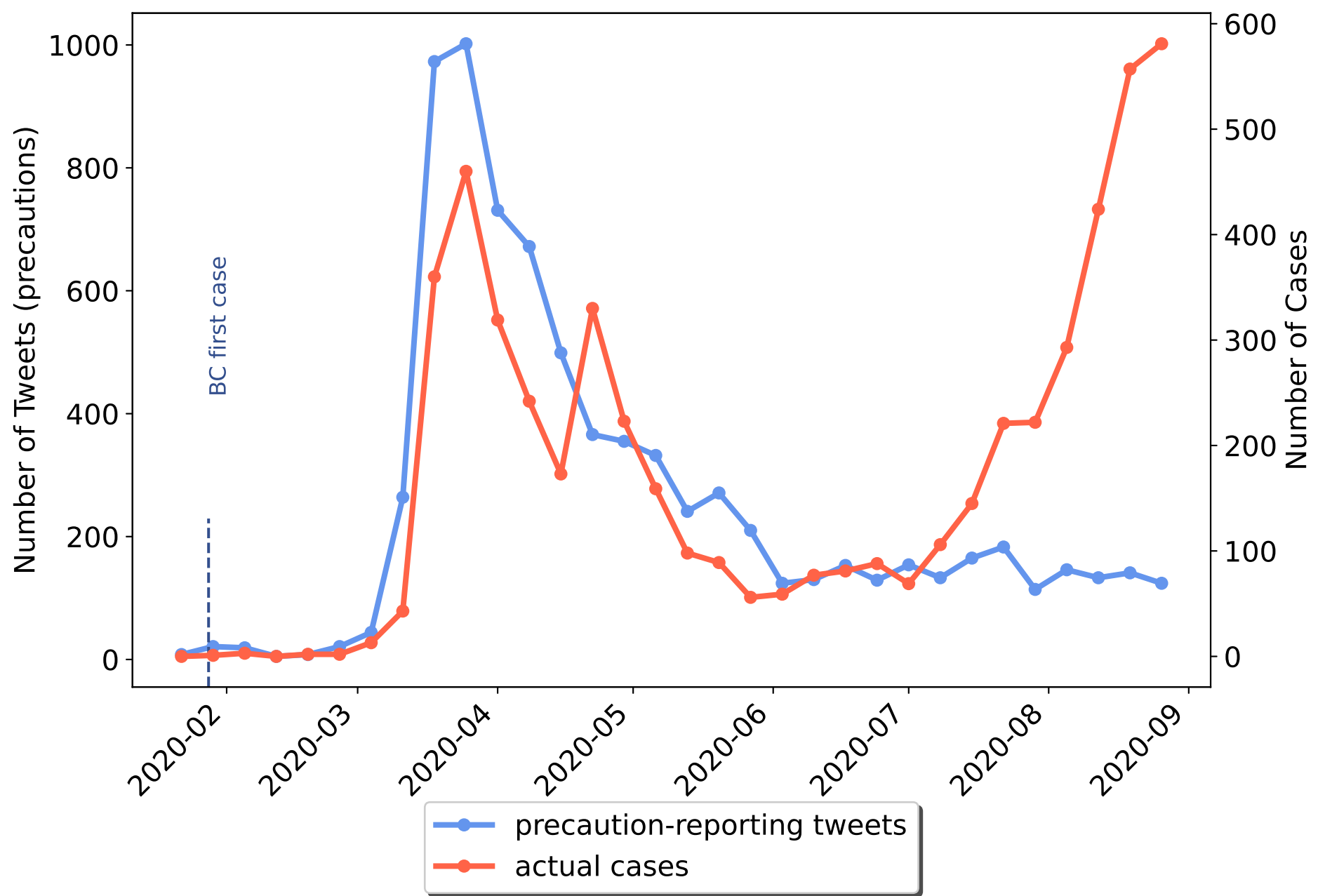

Supplement: Supplementary file 2 [file Data_Sheet_1.ZIP › figures/British_Columbia_precaution_twitter-eps-converted-to.pdf]

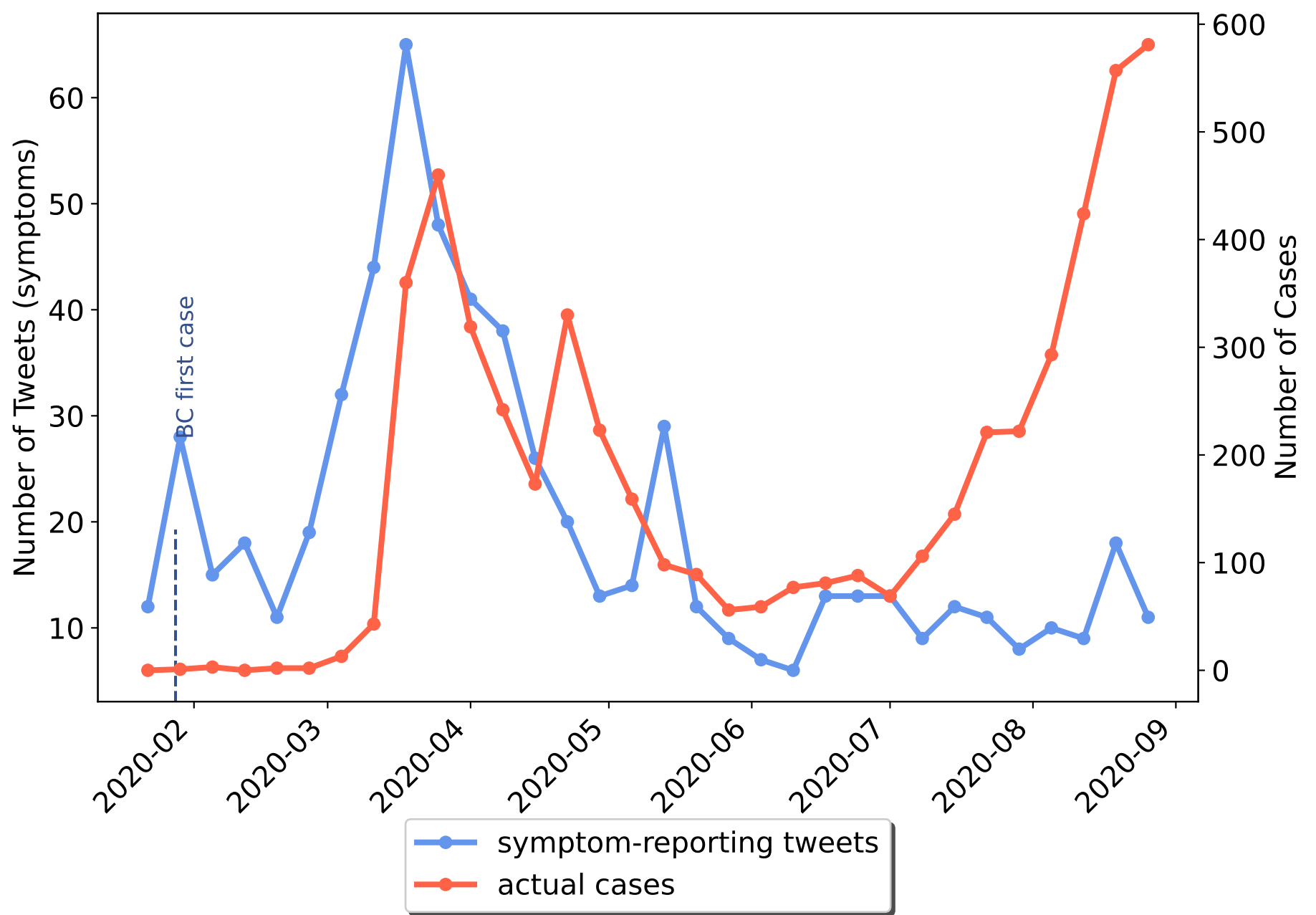

Supplement: Supplementary file 2 [file Data_Sheet_1.ZIP › figures/British_Columbia_symptom_twitter-eps-converted-to.pdf]

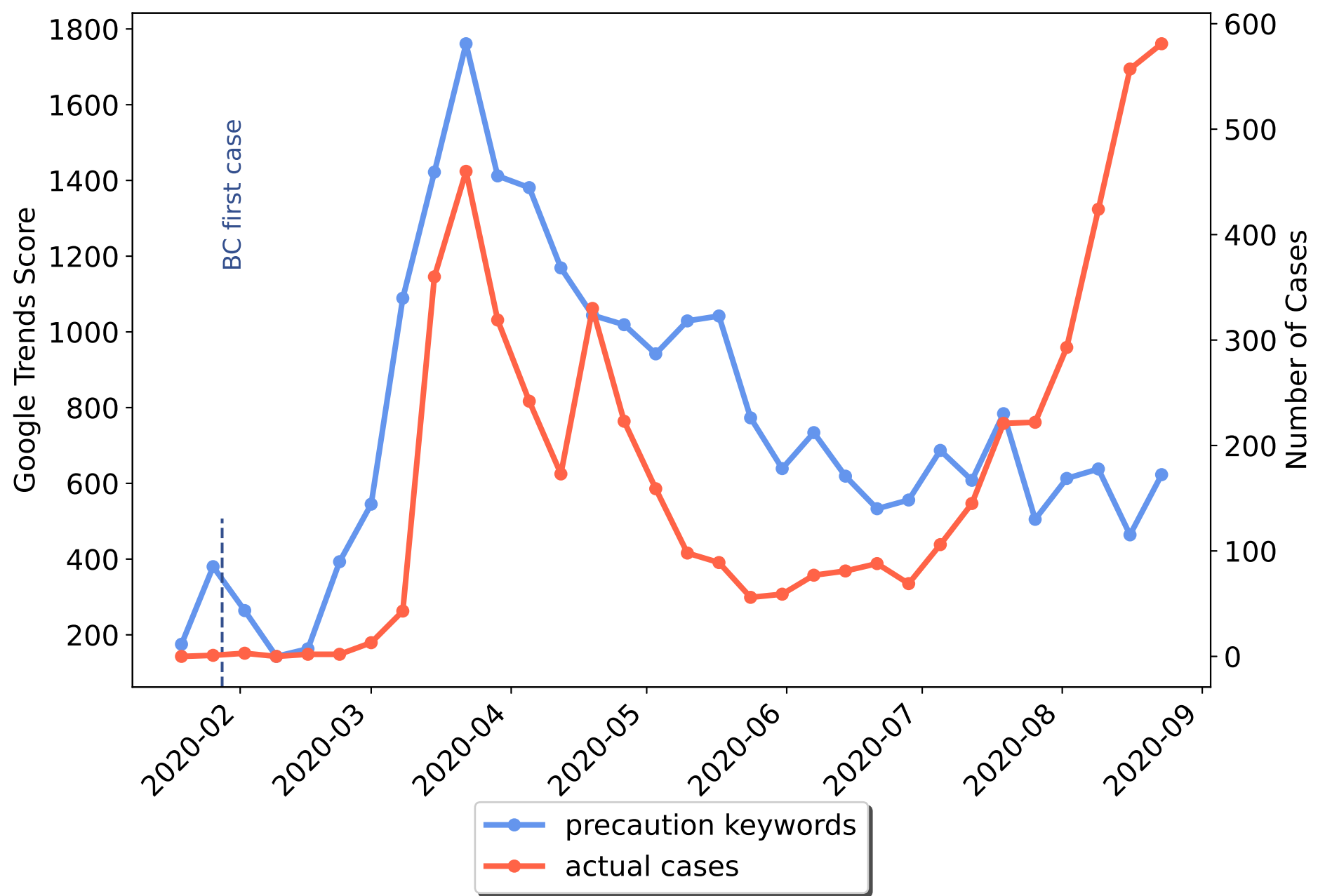

Supplement: Supplementary file 2 [file Data_Sheet_1.ZIP › figures/British_Columbia_totalprecaution_GT-eps-converted-to.pdf]

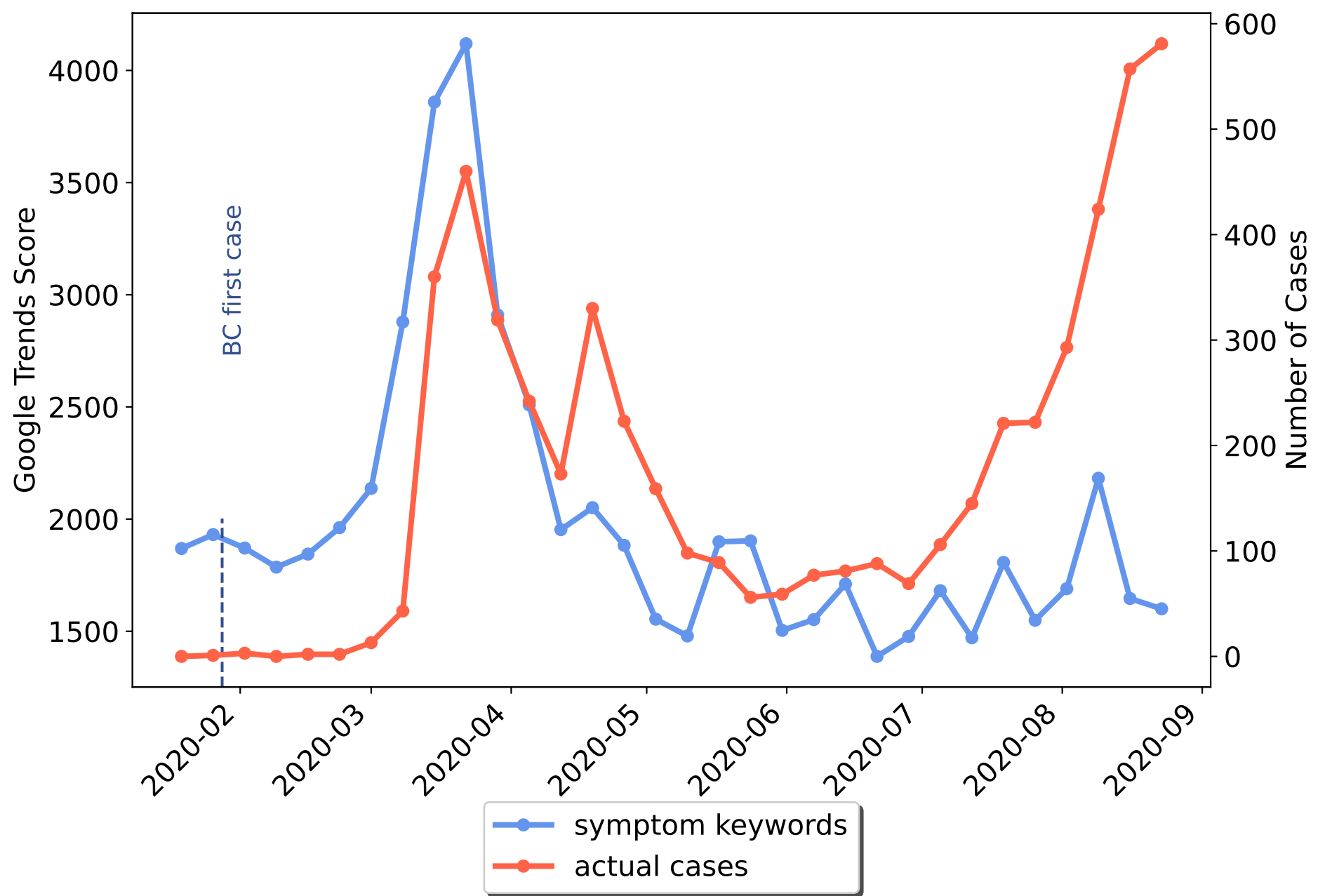

Supplement: Supplementary file 2 [file Data_Sheet_1.ZIP › figures/British_Columbia_totalsymptom_GT-eps-converted-to.pdf]

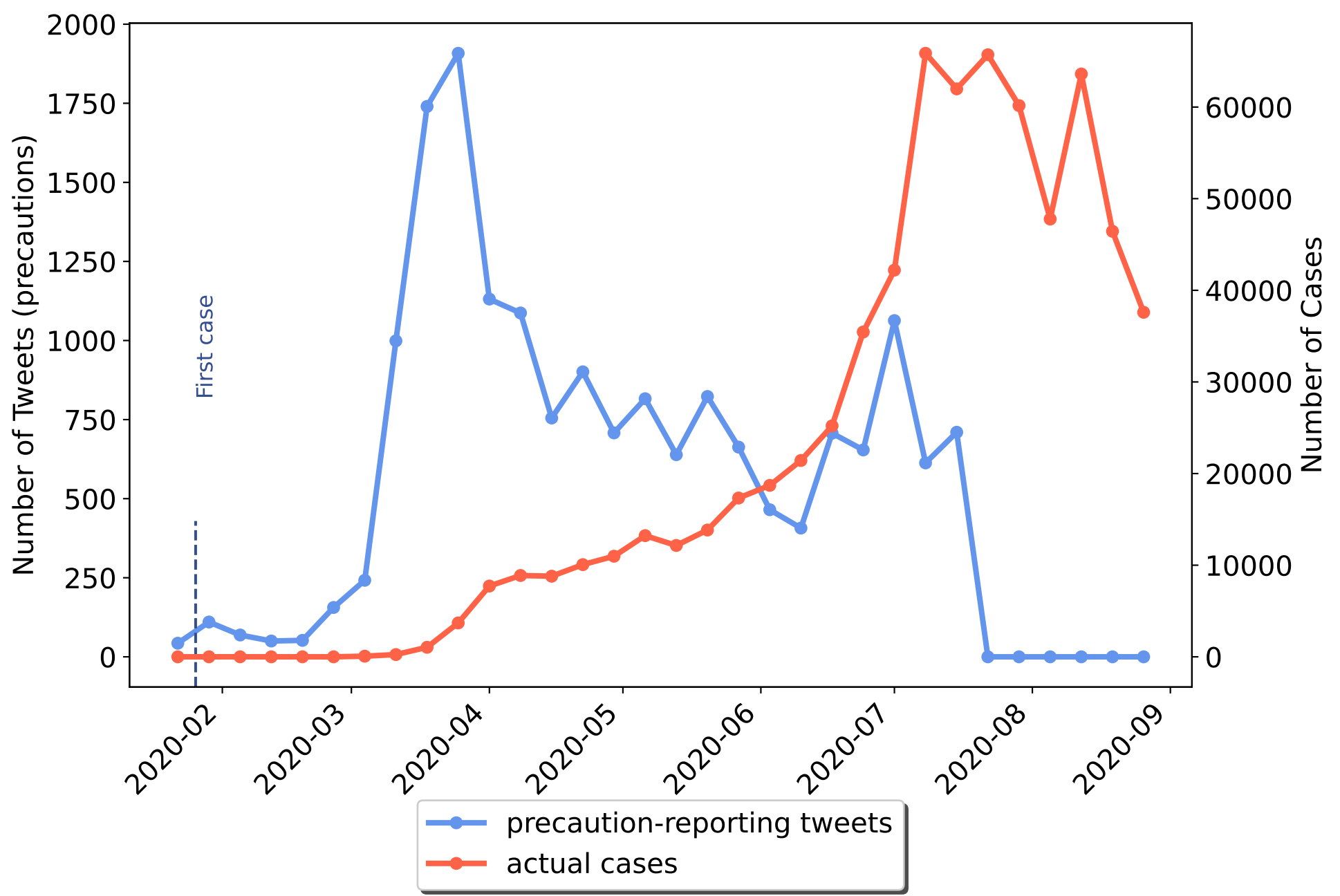

Supplement: Supplementary file 2 [file Data_Sheet_1.ZIP › figures/California_precaution_twitter-eps-converted-to.pdf]

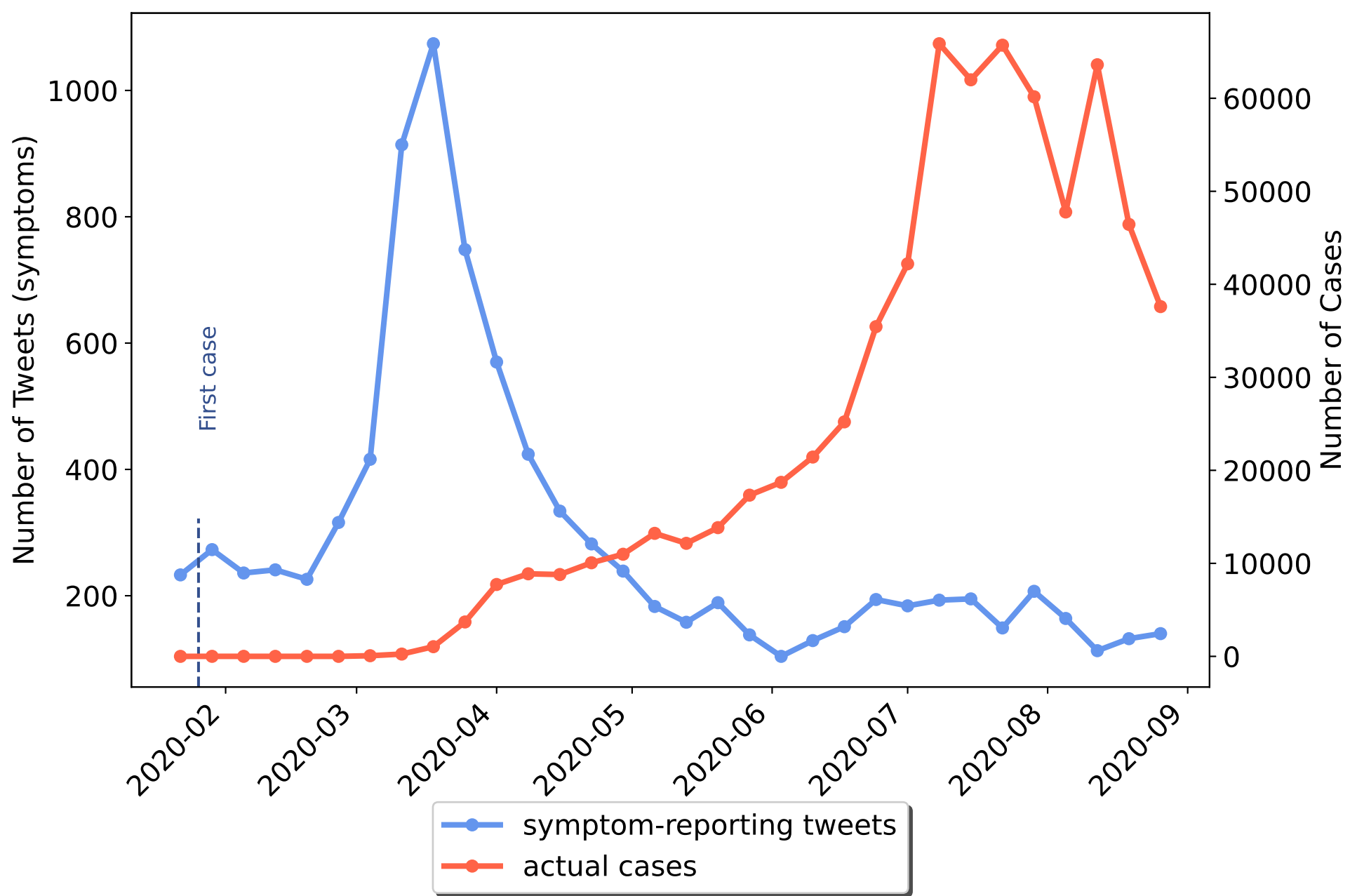

Supplement: Supplementary file 2 [file Data_Sheet_1.ZIP › figures/California_symptom_twitter-eps-converted-to.pdf]

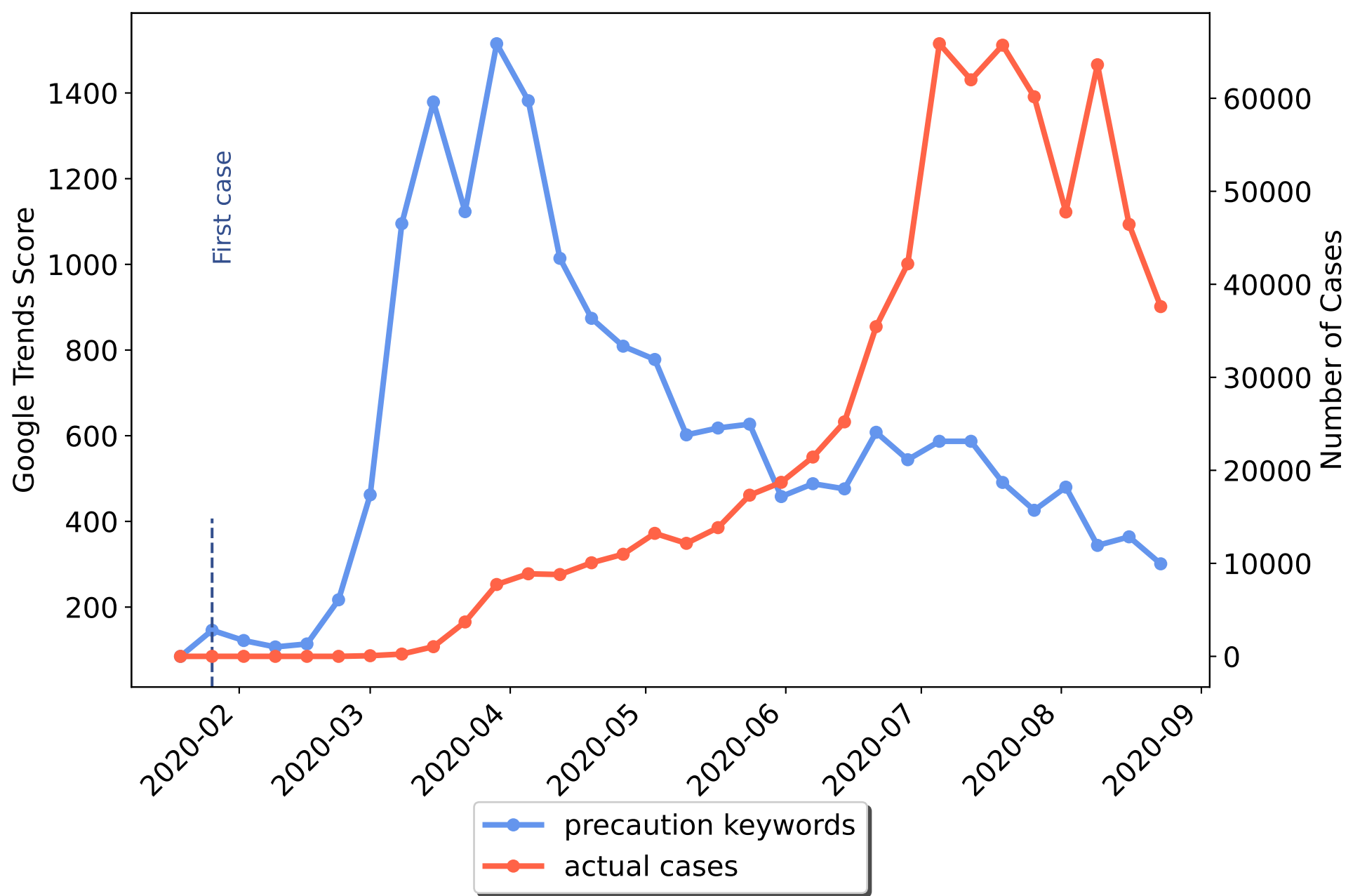

Supplement: Supplementary file 2 [file Data_Sheet_1.ZIP › figures/California_totalprecaution_GT-eps-converted-to.pdf]

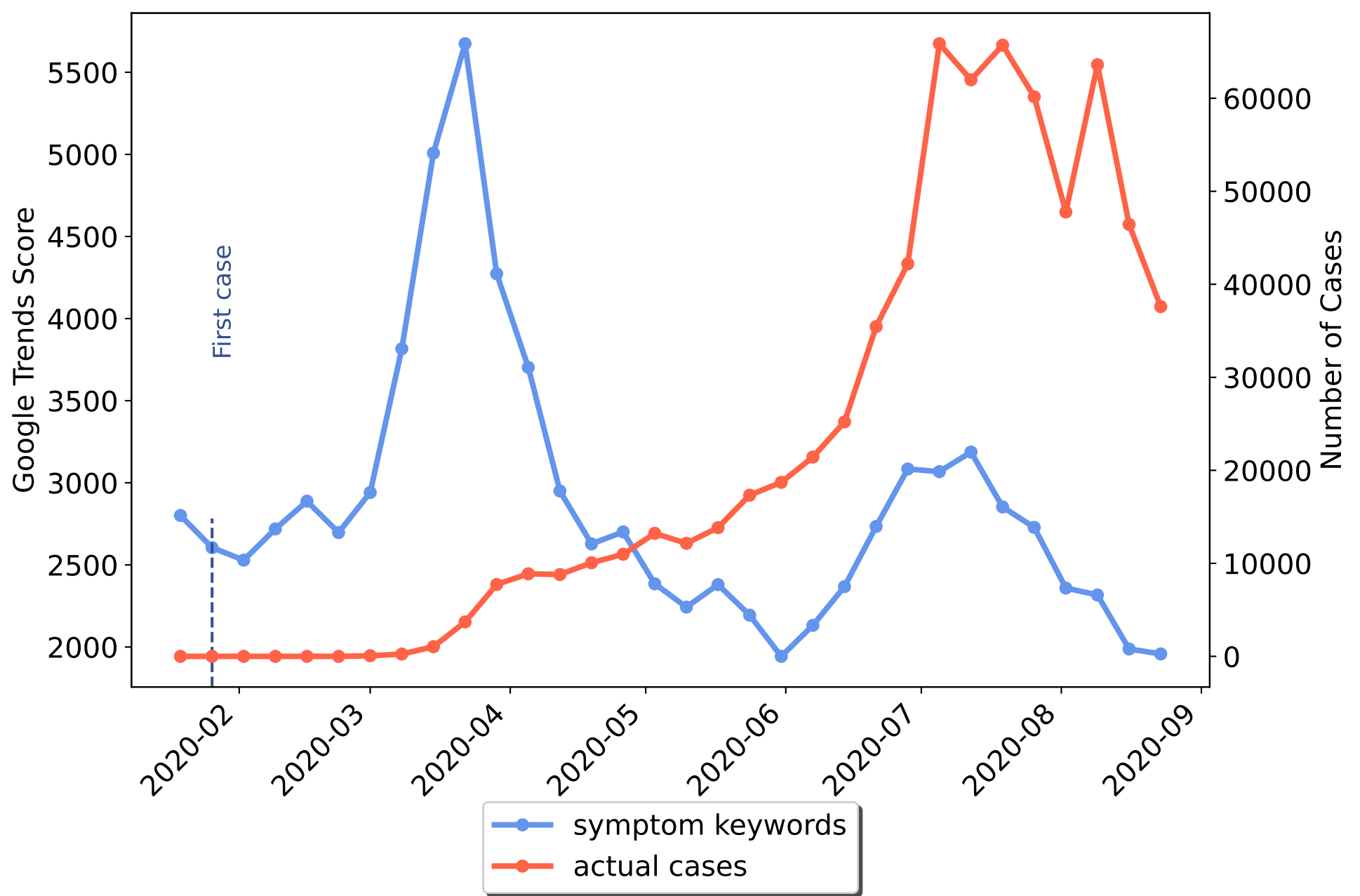

Supplement: Supplementary file 2 [file Data_Sheet_1.ZIP › figures/California_totalsymptom_GT-eps-converted-to.pdf]

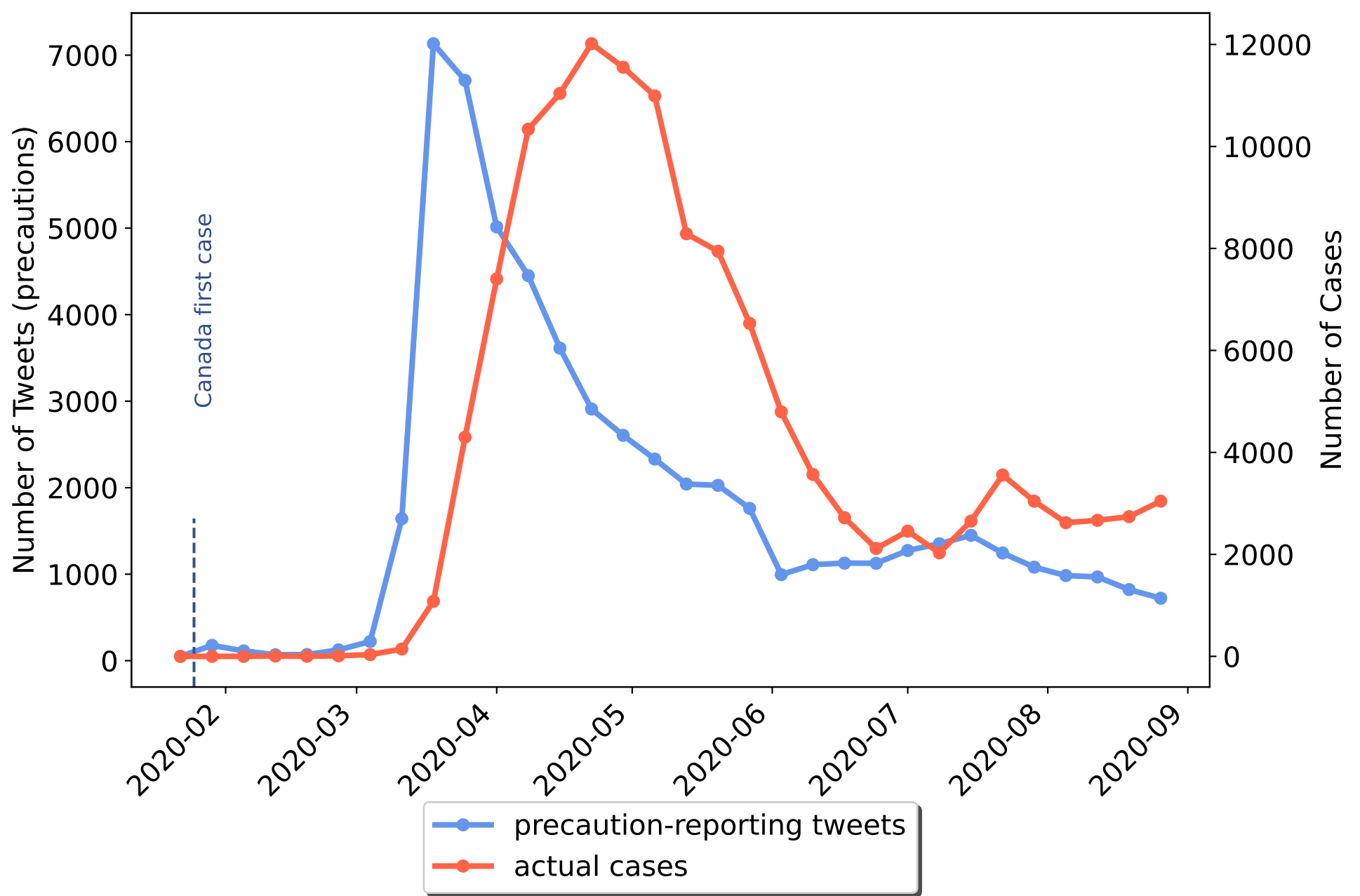

Supplement: Supplementary file 2 [file Data_Sheet_1.ZIP › figures/Canada_precaution_twitter-eps-converted-to.pdf]

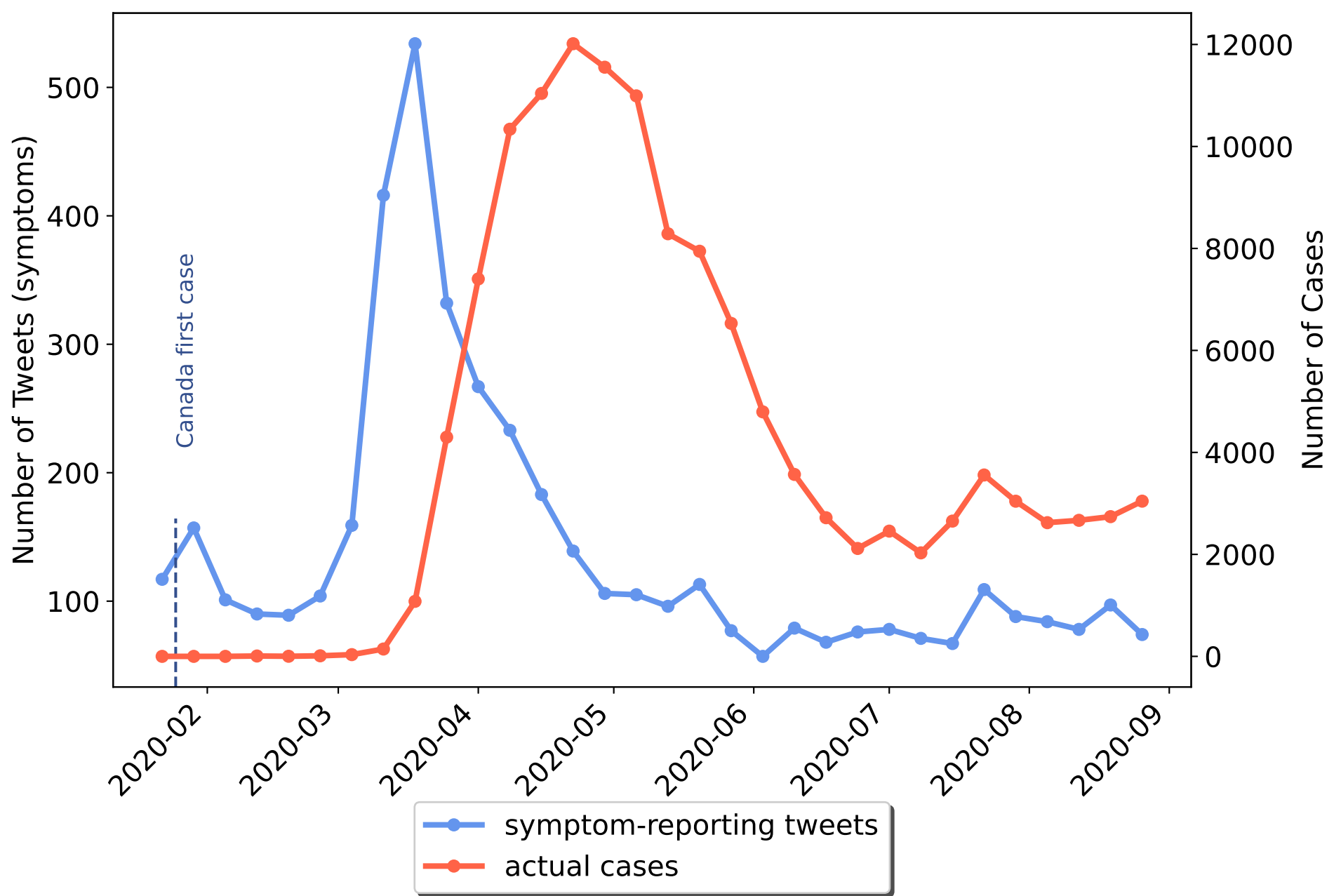

Supplement: Supplementary file 2 [file Data_Sheet_1.ZIP › figures/Canada_symptom_twitter-eps-converted-to.pdf]

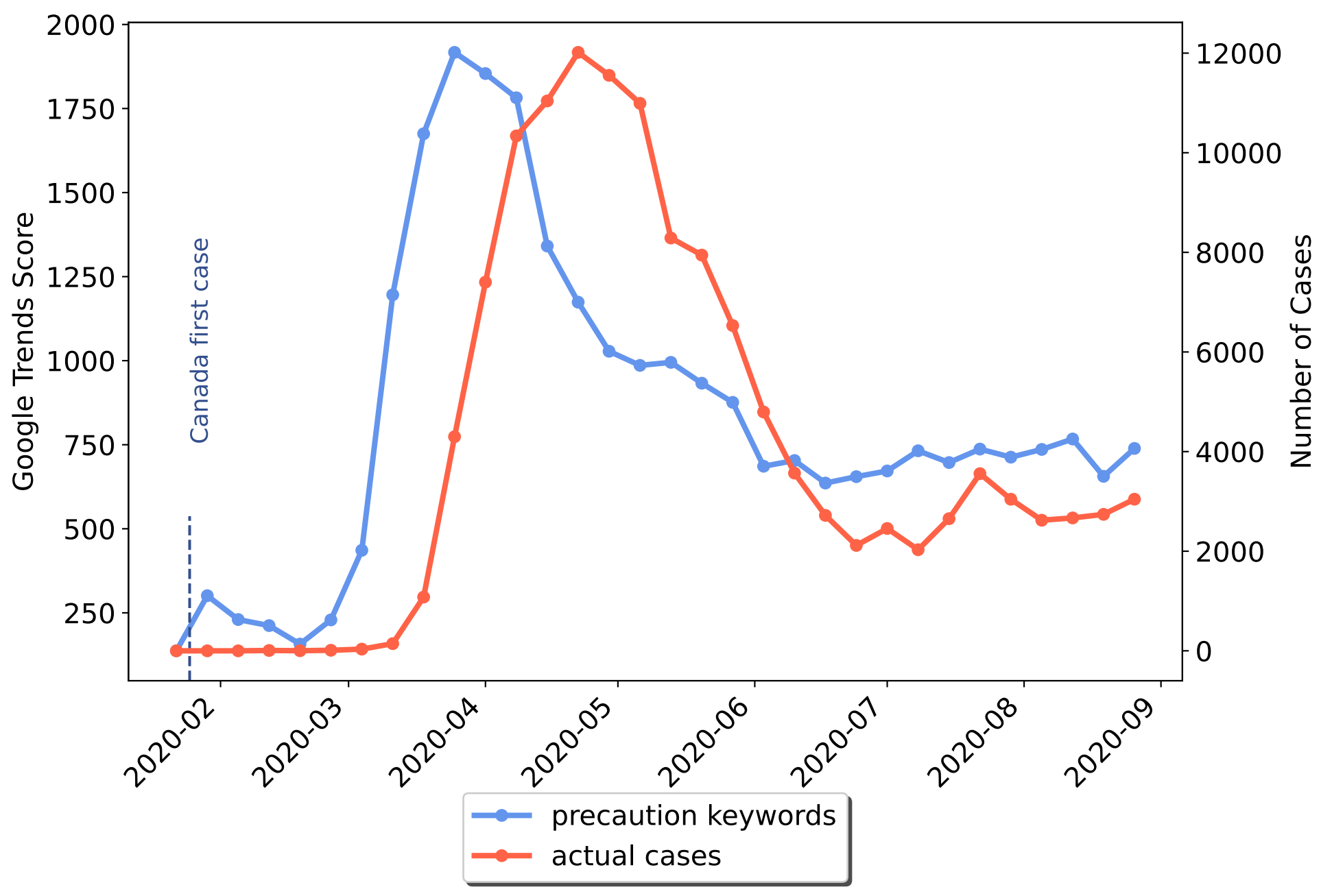

Supplement: Supplementary file 2 [file Data_Sheet_1.ZIP › figures/Canada_totalprecaution_GT-eps-converted-to.pdf]

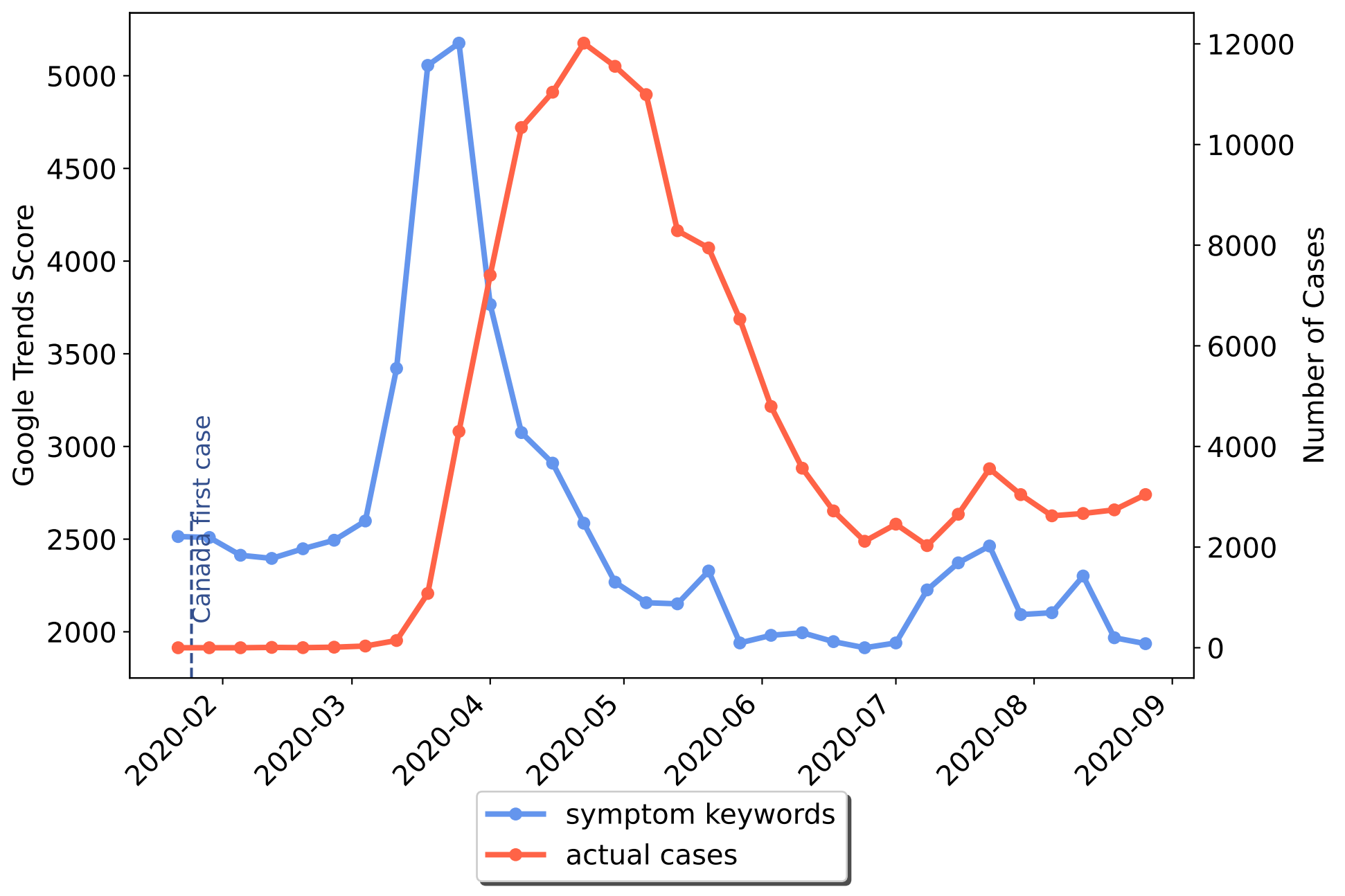

Supplement: Supplementary file 2 [file Data_Sheet_1.ZIP › figures/Canada_totalsymptom_GT-eps-converted-to.pdf]

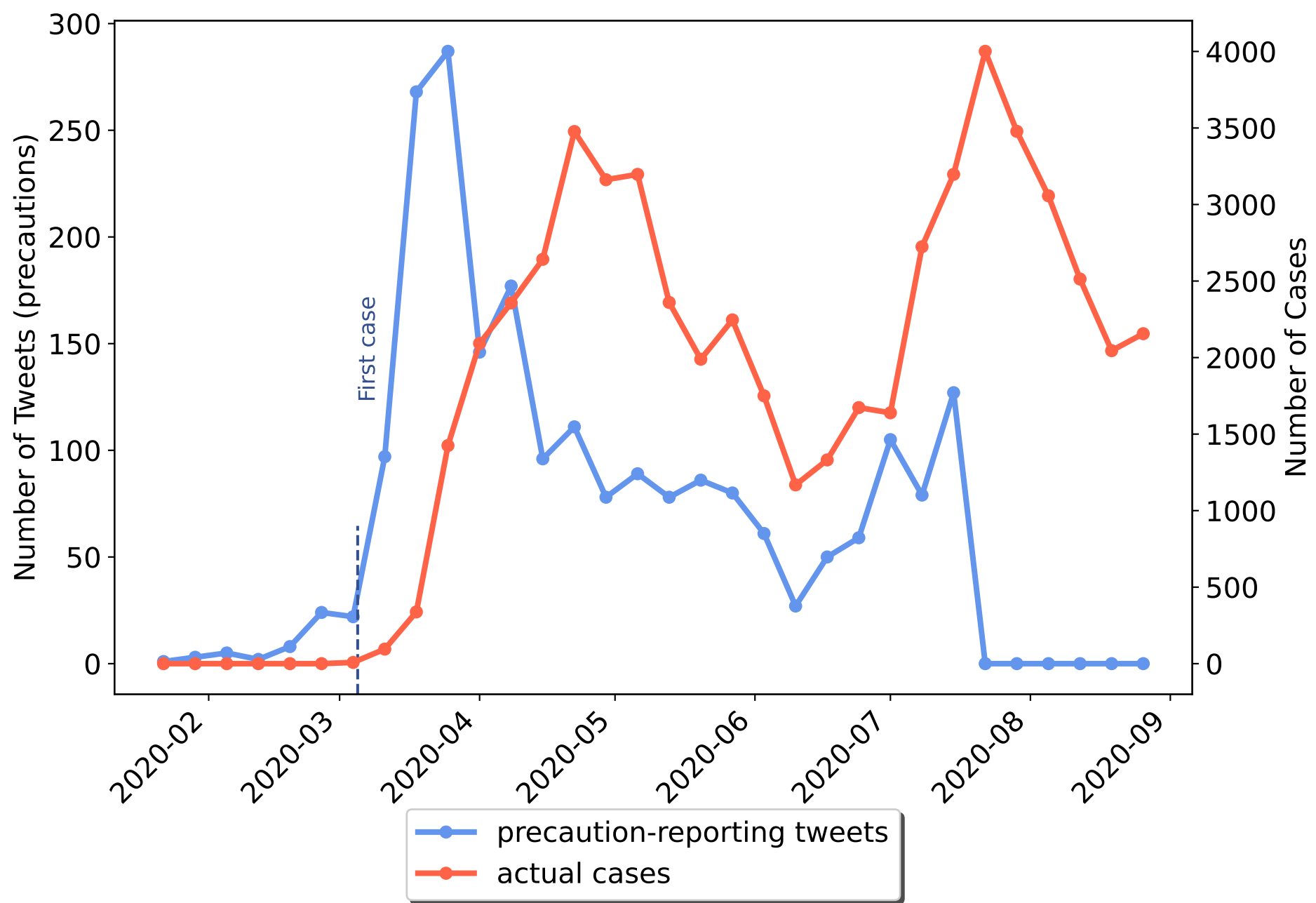

Supplement: Supplementary file 2 [file Data_Sheet_1.ZIP › figures/Colorado_precaution_twitter-eps-converted-to.pdf]

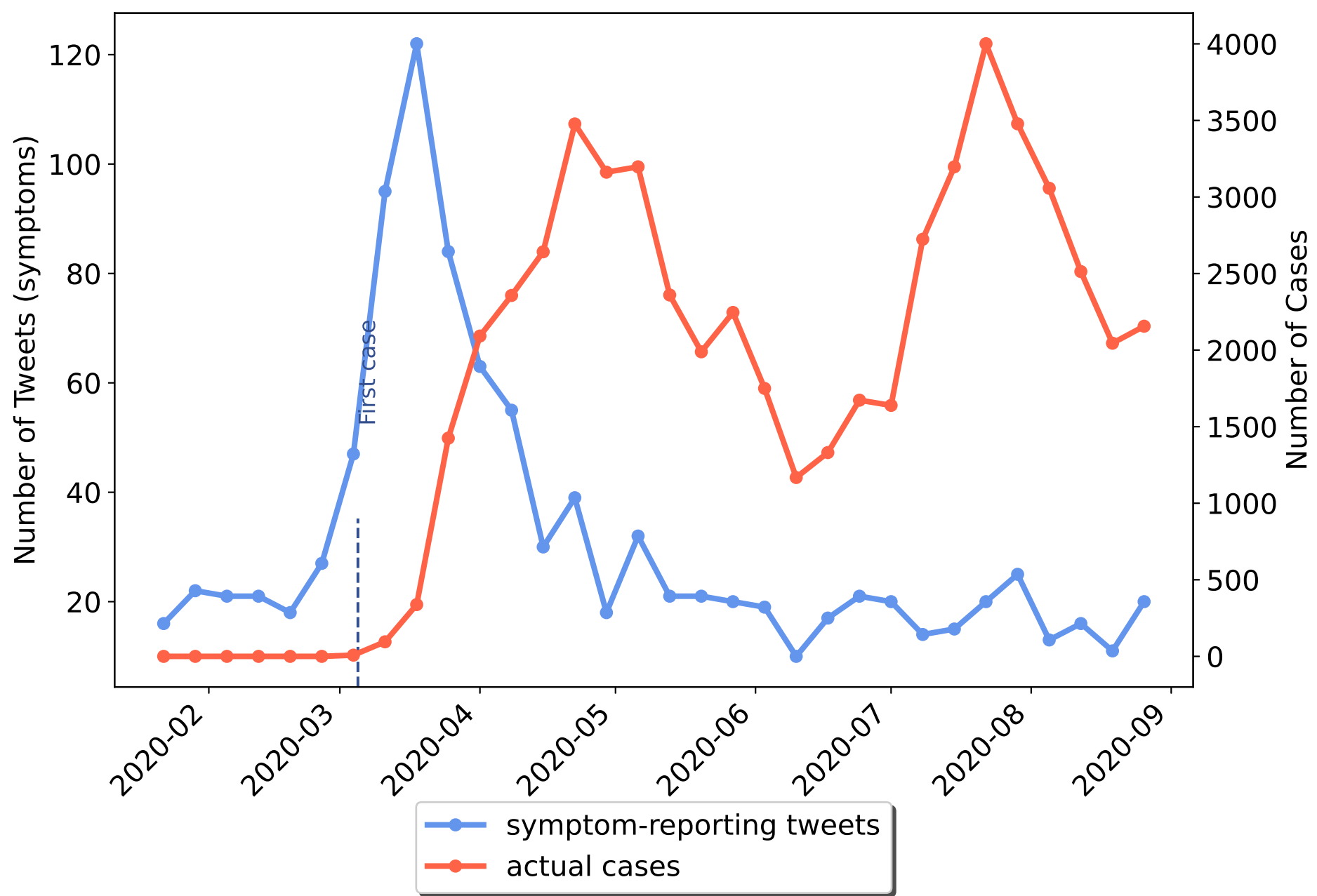

Supplement: Supplementary file 2 [file Data_Sheet_1.ZIP › figures/Colorado_symptom_twitter-eps-converted-to.pdf]

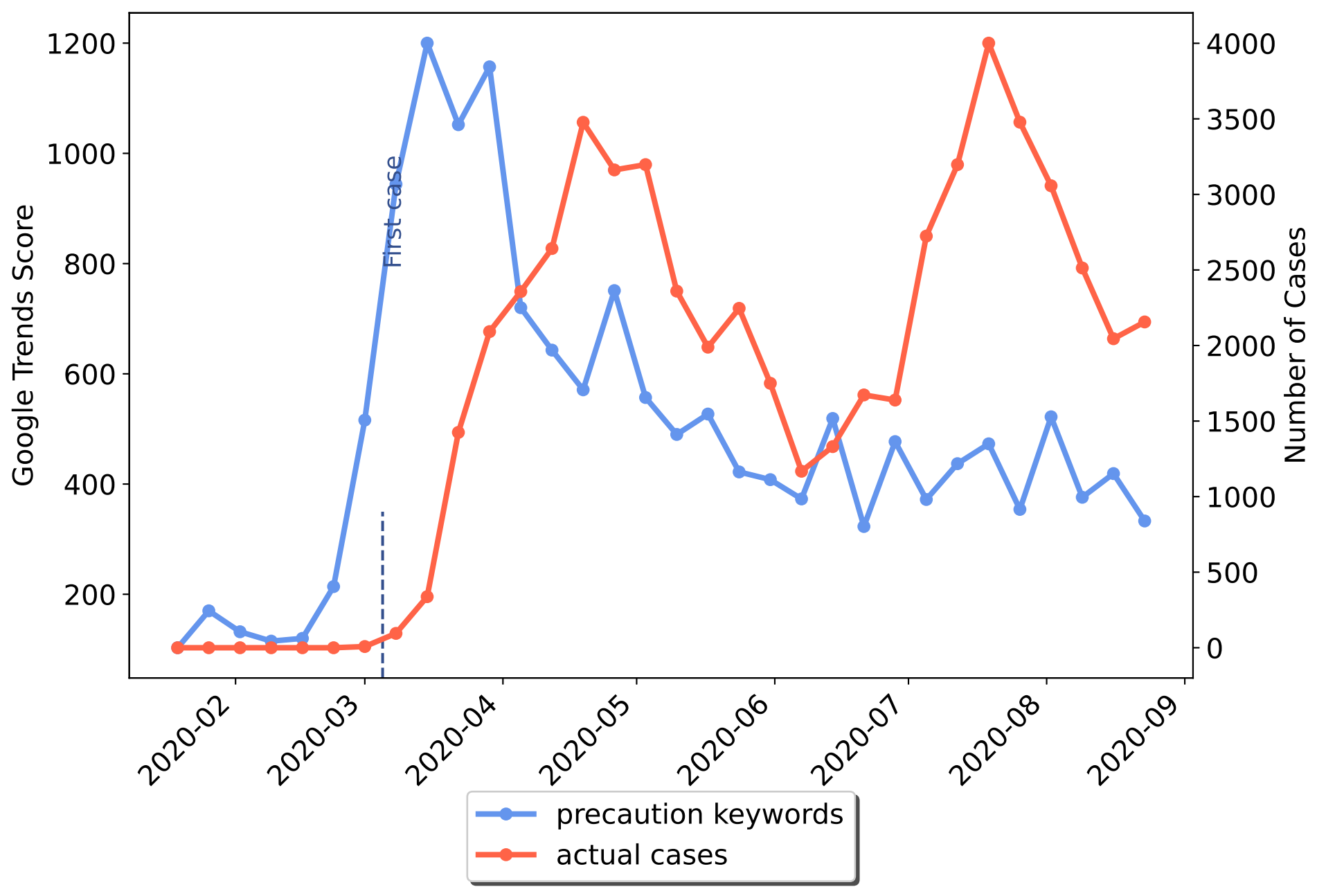

Supplement: Supplementary file 2 [file Data_Sheet_1.ZIP › figures/Colorado_totalprecaution_GT-eps-converted-to.pdf]

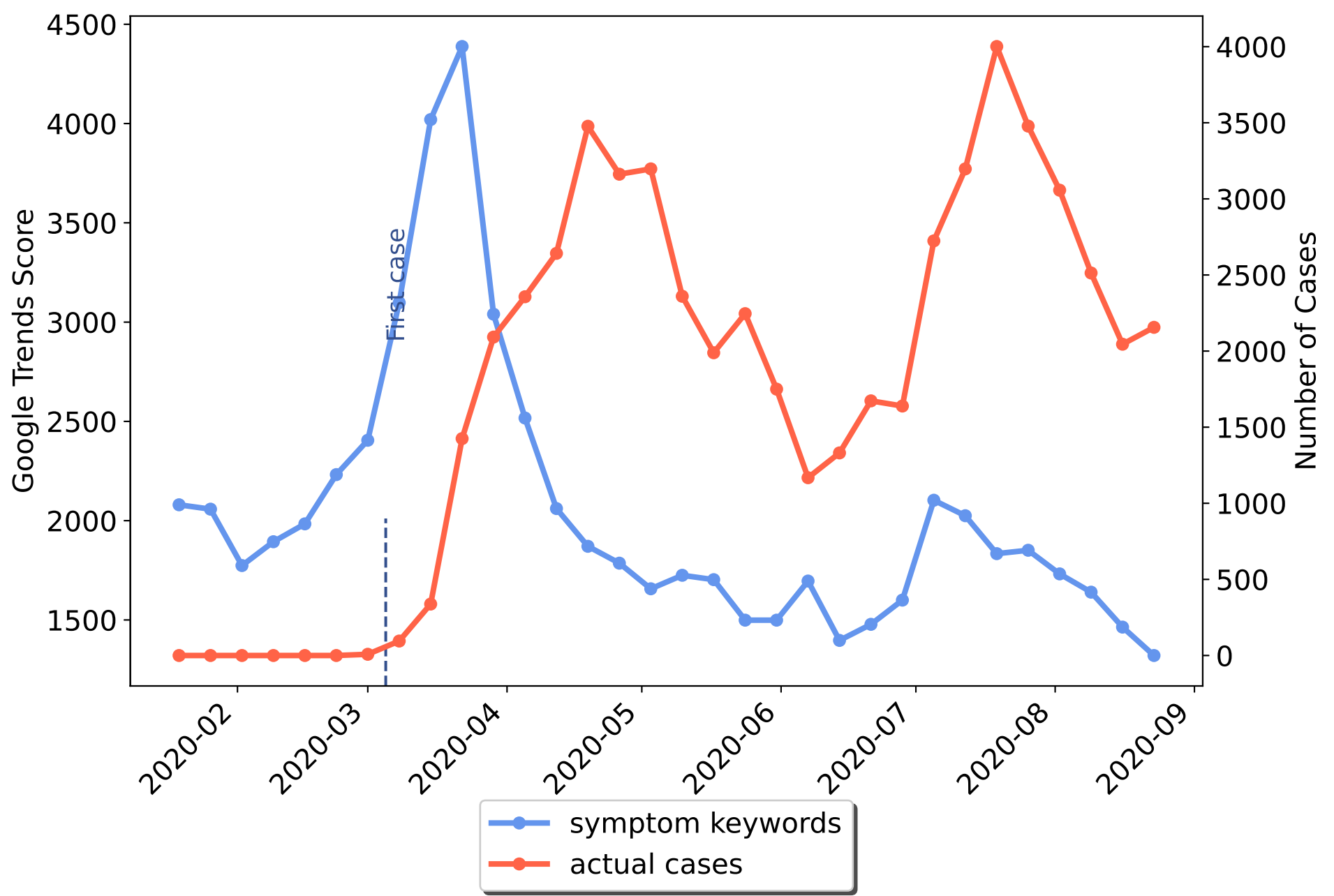

Supplement: Supplementary file 2 [file Data_Sheet_1.ZIP › figures/Colorado_totalsymptom_GT-eps-converted-to.pdf]

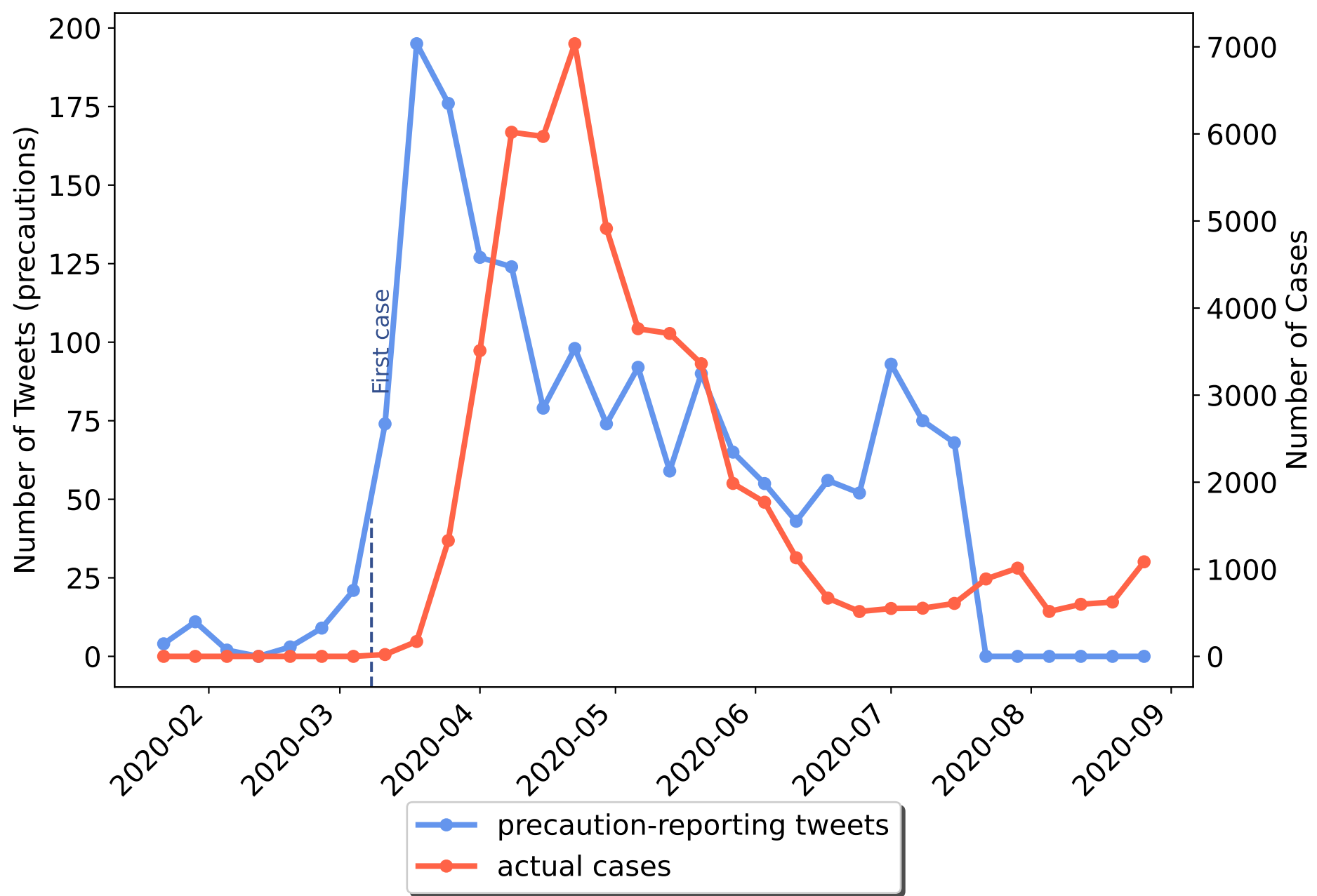

Supplement: Supplementary file 2 [file Data_Sheet_1.ZIP › figures/Connecticut_precaution_twitter-eps-converted-to.pdf]

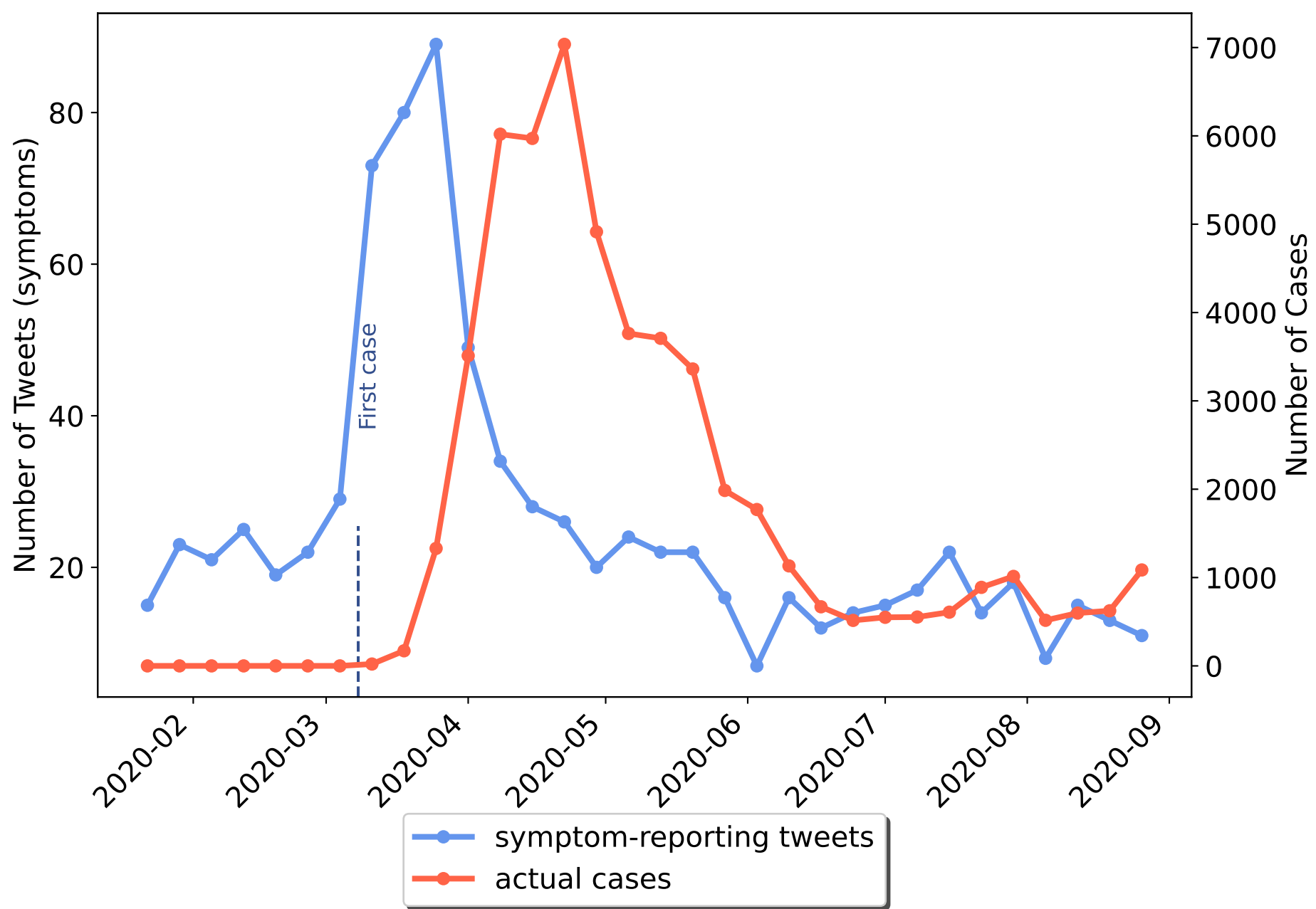

Supplement: Supplementary file 2 [file Data_Sheet_1.ZIP › figures/Connecticut_symptom_twitter-eps-converted-to.pdf]

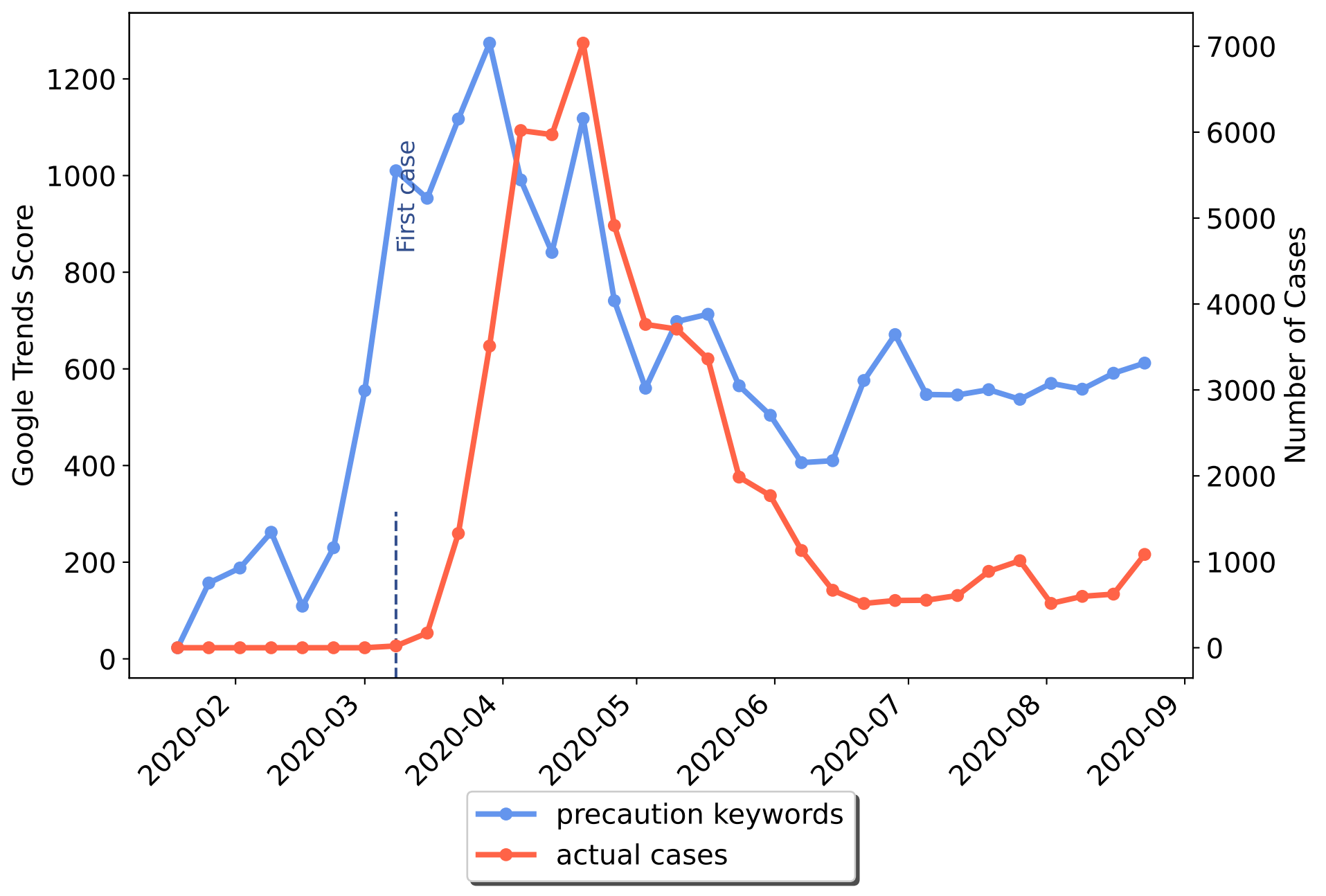

Supplement: Supplementary file 2 [file Data_Sheet_1.ZIP › figures/Connecticut_totalprecaution_GT-eps-converted-to.pdf]

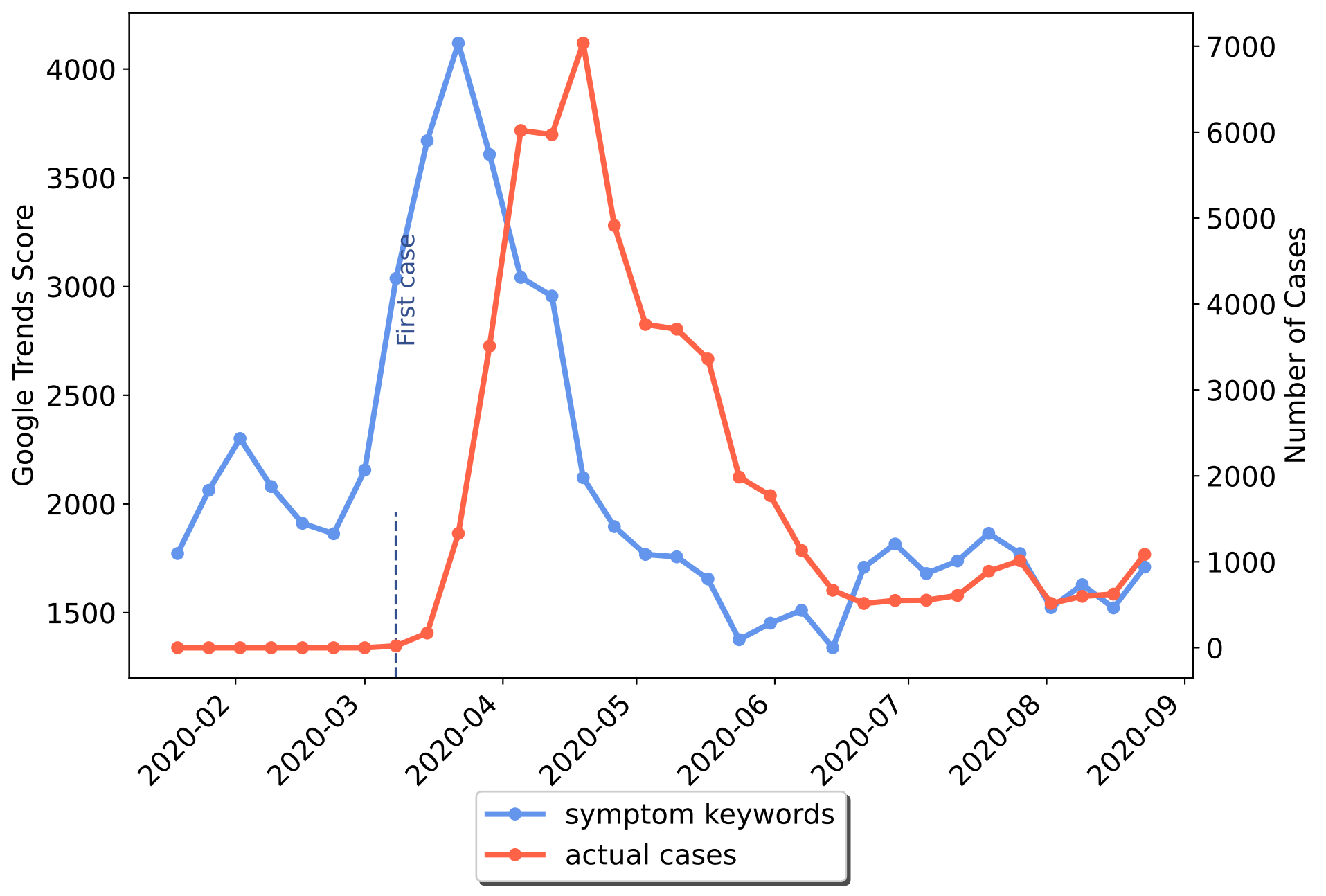

Supplement: Supplementary file 2 [file Data_Sheet_1.ZIP › figures/Connecticut_totalsymptom_GT-eps-converted-to.pdf]

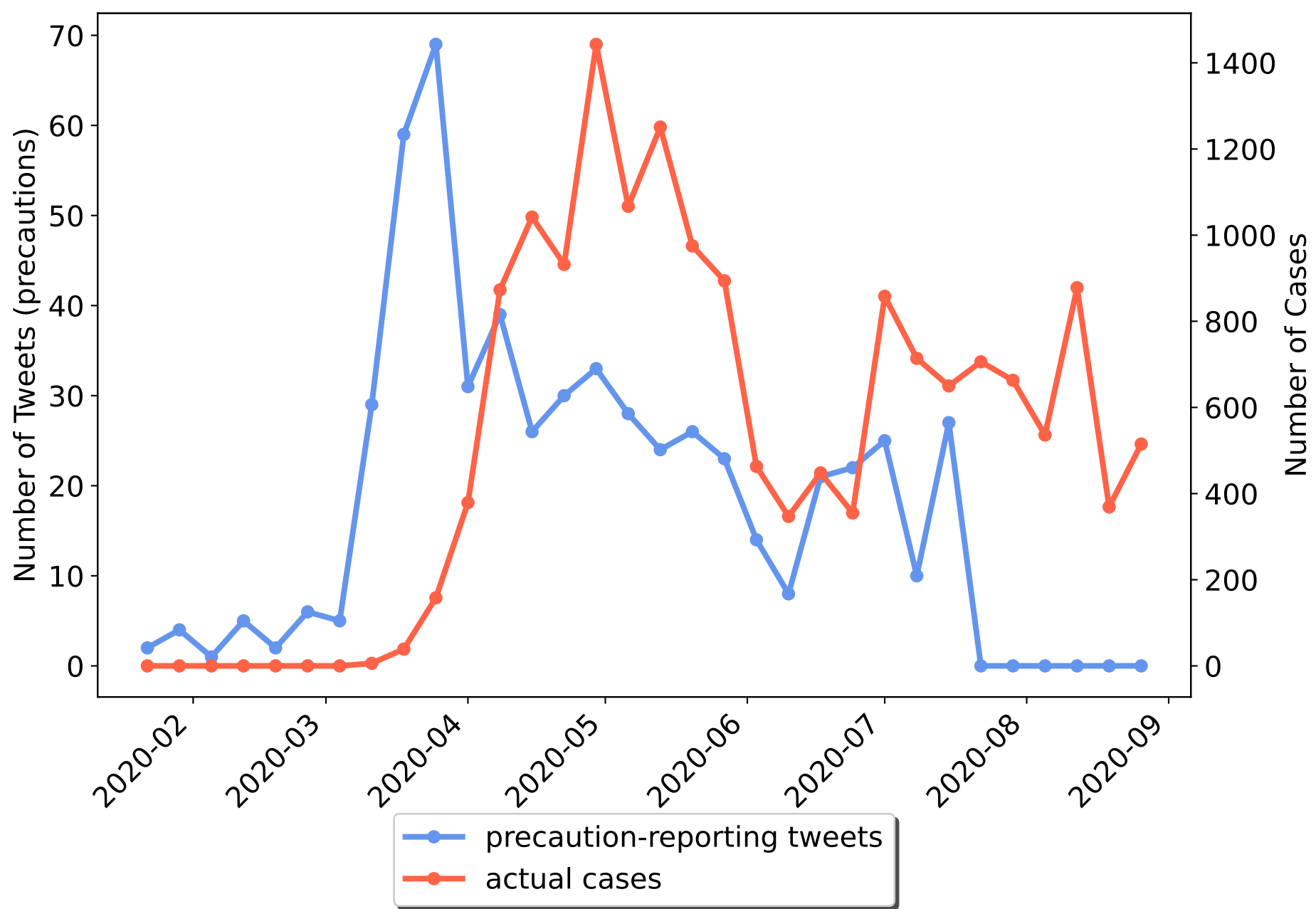

Supplement: Supplementary file 2 [file Data_Sheet_1.ZIP › figures/Delaware_precaution_twitter-eps-converted-to.pdf]

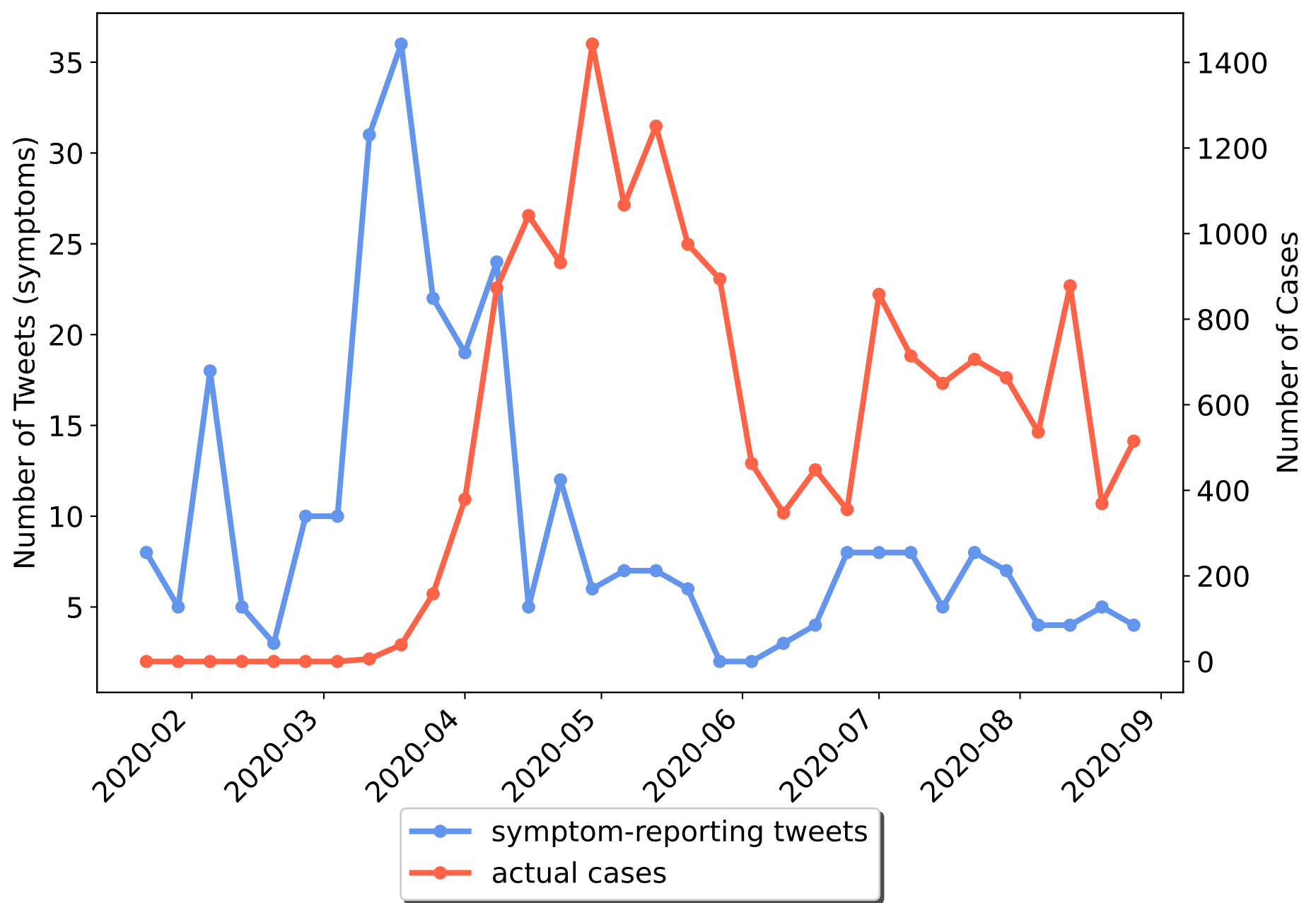

Supplement: Supplementary file 2 [file Data_Sheet_1.ZIP › figures/Delaware_symptom_twitter-eps-converted-to.pdf]

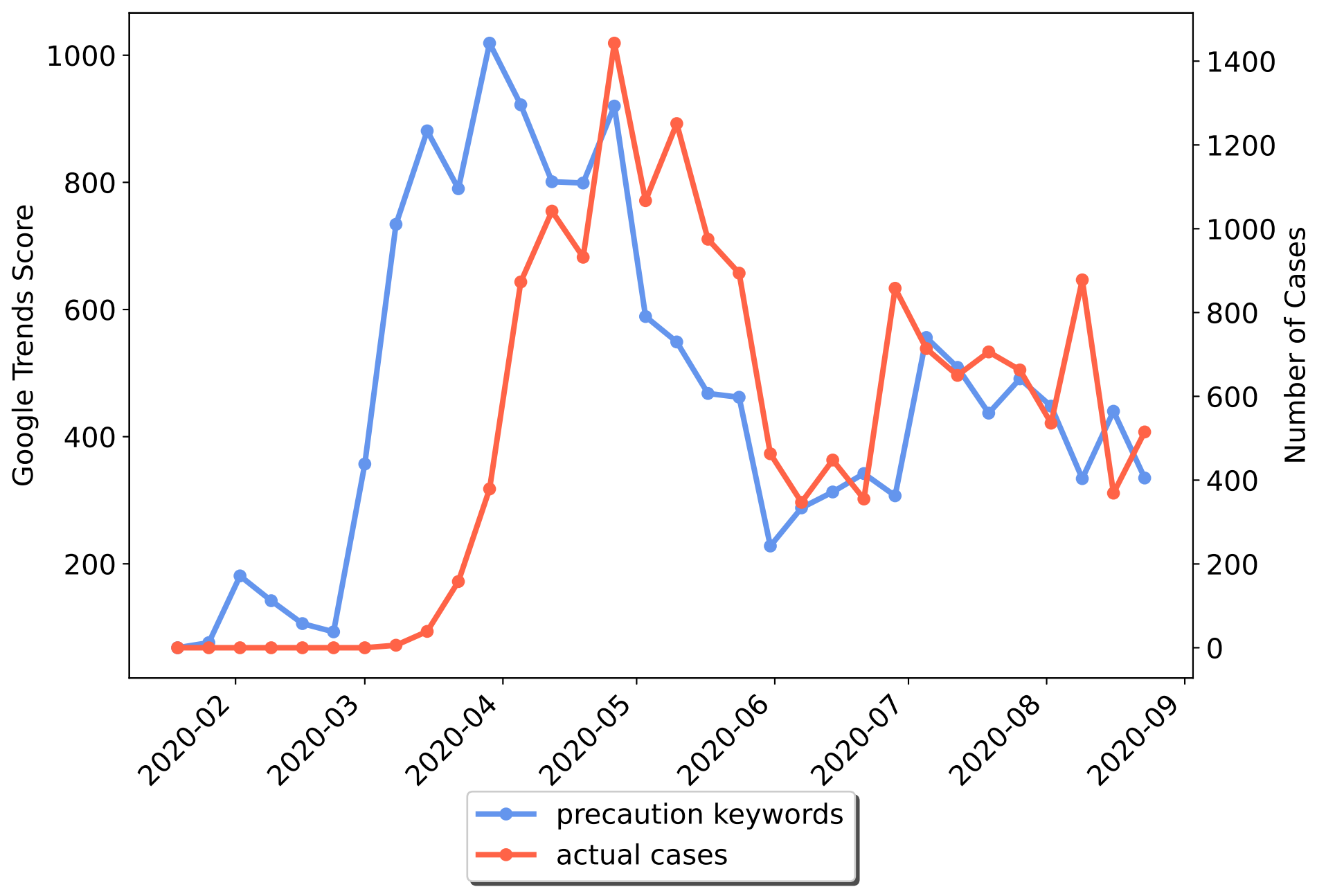

Supplement: Supplementary file 2 [file Data_Sheet_1.ZIP › figures/Delaware_totalprecaution_GT-eps-converted-to.pdf]

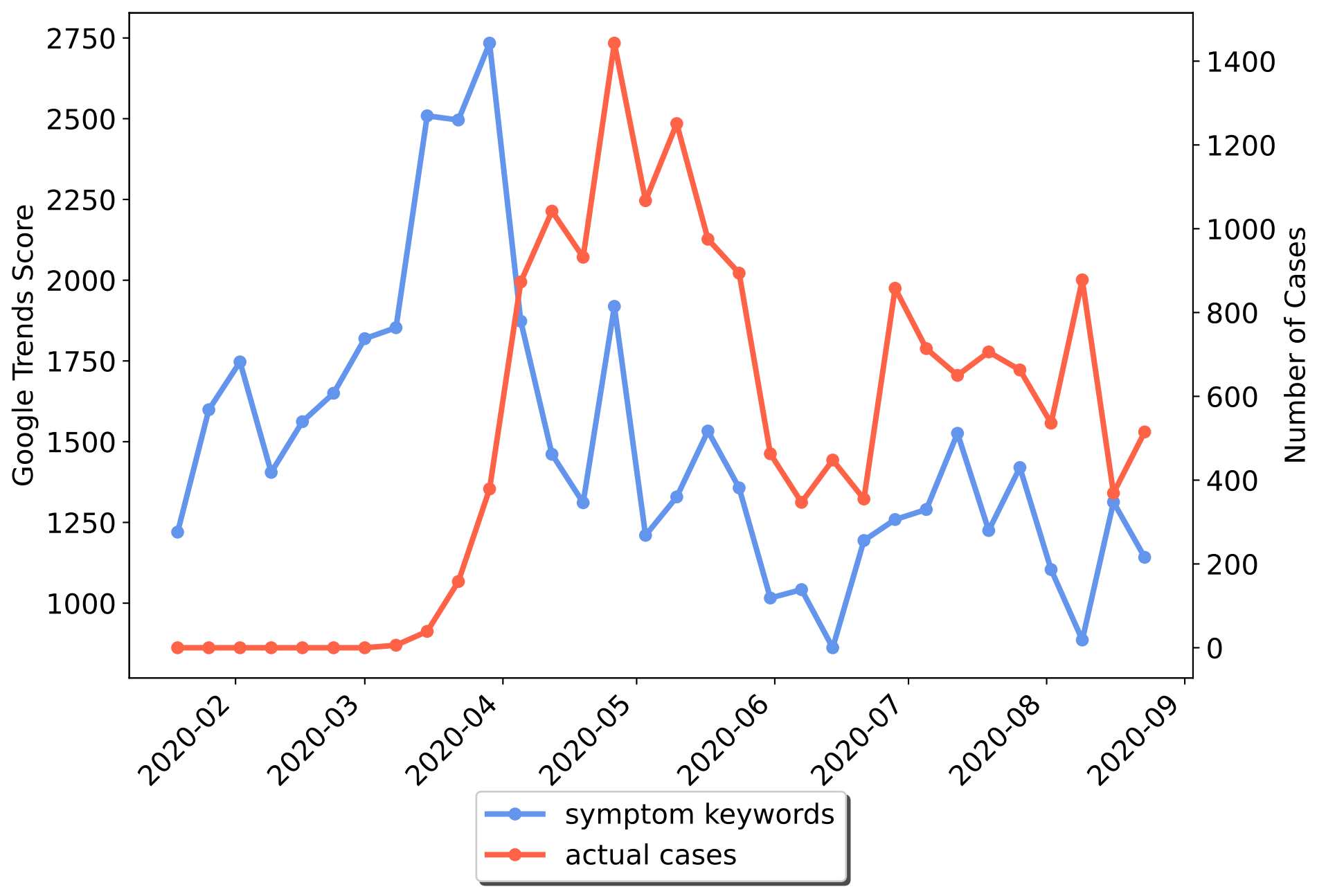

Supplement: Supplementary file 2 [file Data_Sheet_1.ZIP › figures/Delaware_totalsymptom_GT-eps-converted-to.pdf]

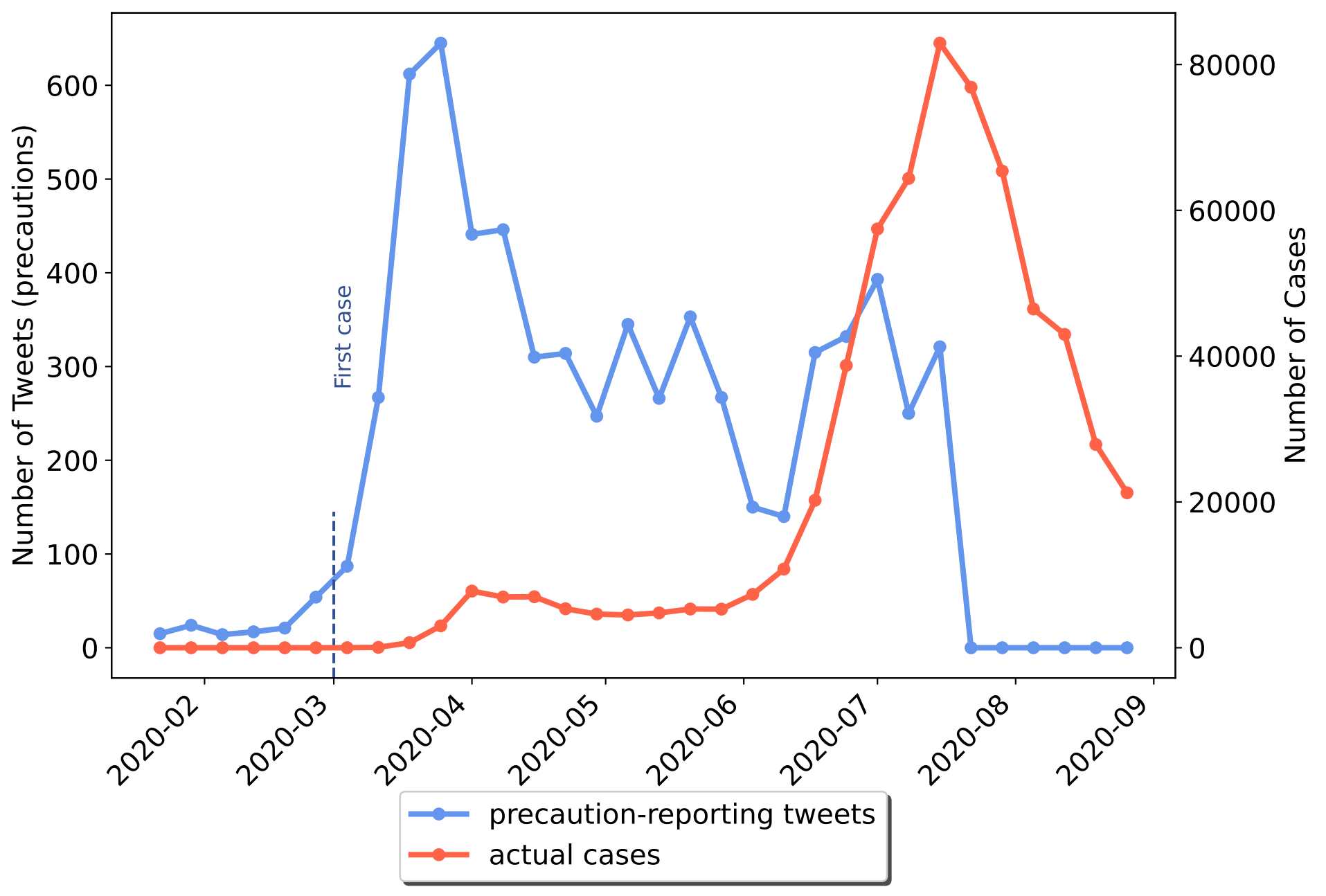

Supplement: Supplementary file 2 [file Data_Sheet_1.ZIP › figures/Florida_precaution_twitter-eps-converted-to.pdf]

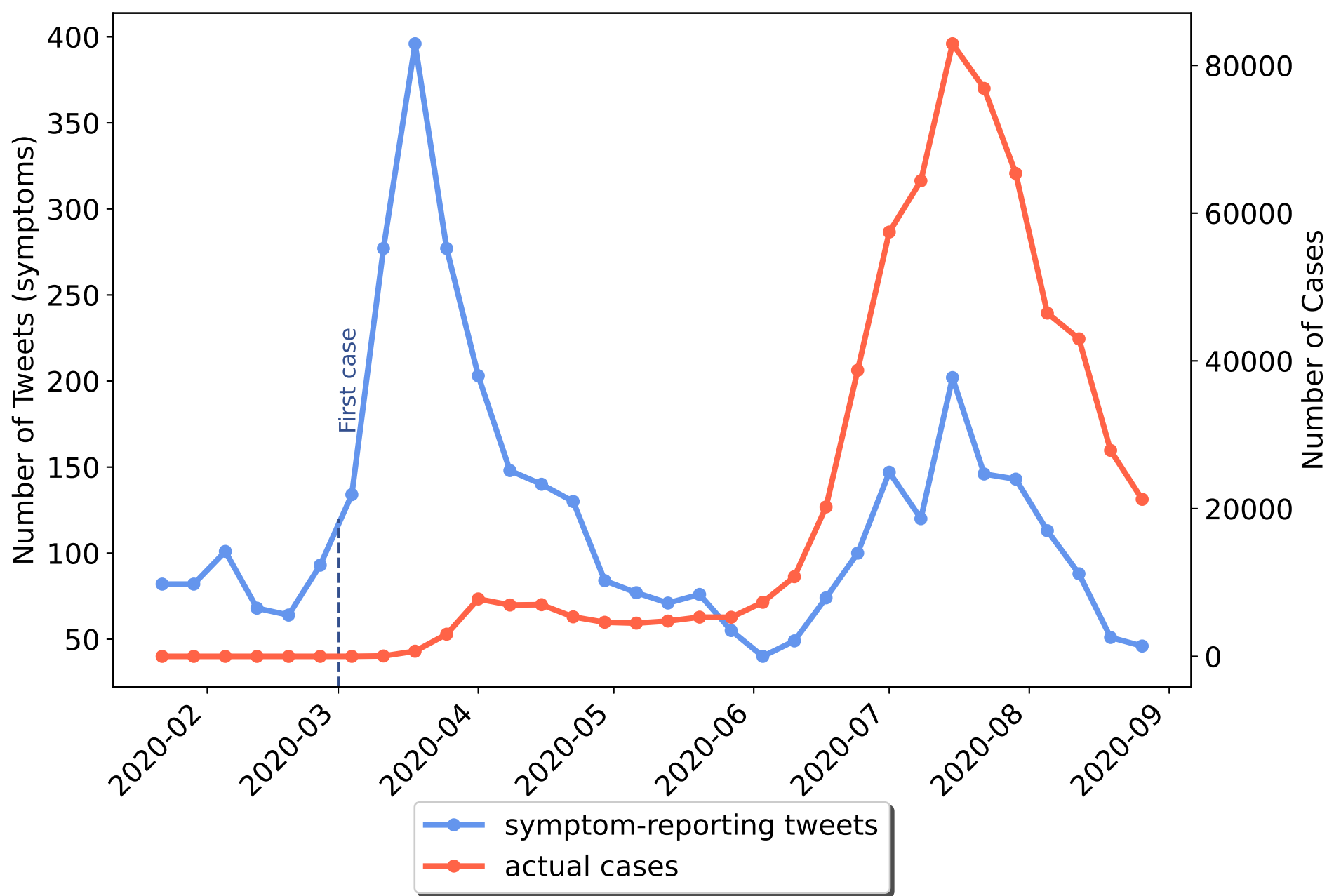

Supplement: Supplementary file 2 [file Data_Sheet_1.ZIP › figures/Florida_symptom_twitter-eps-converted-to.pdf]

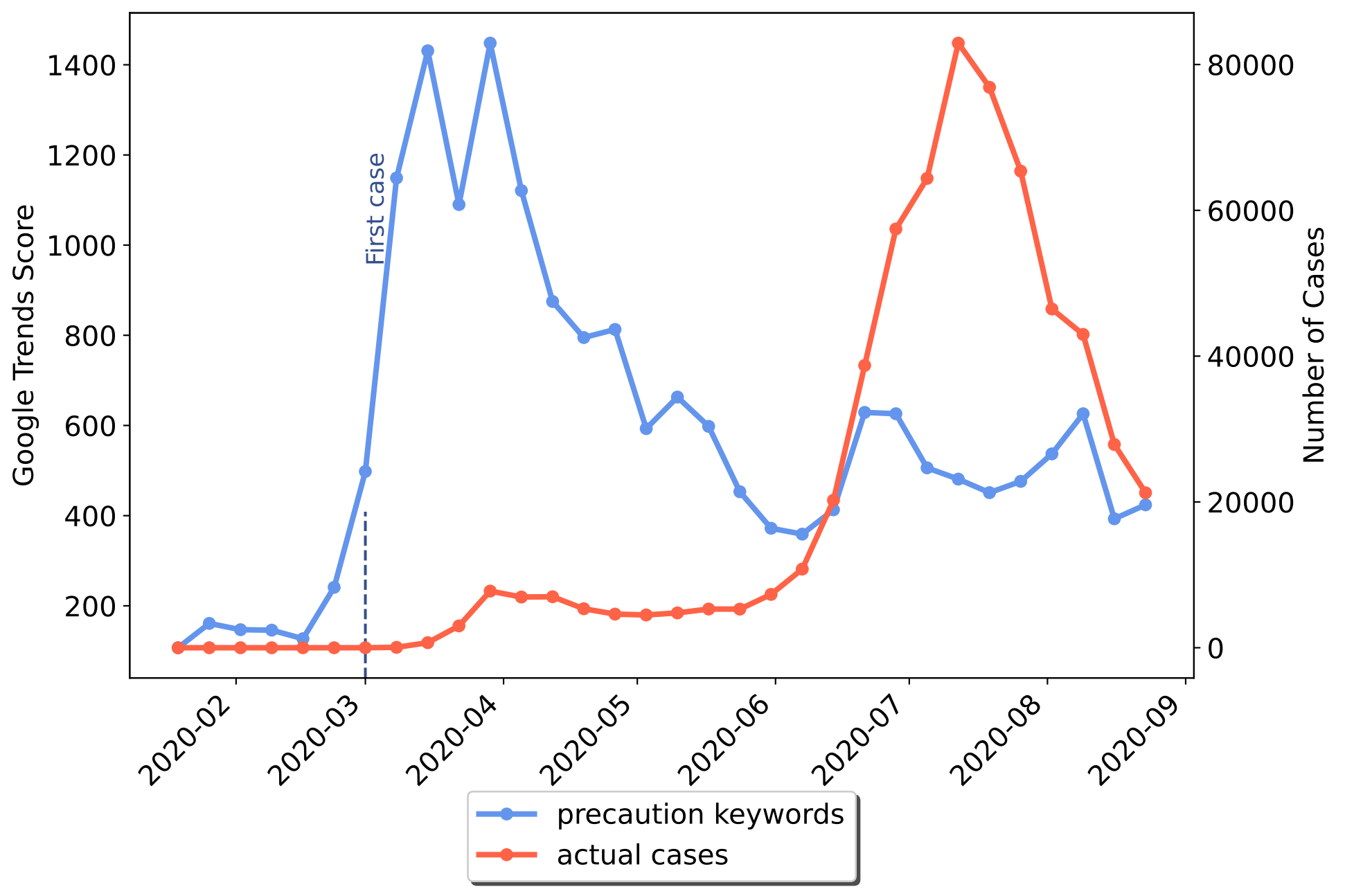

Supplement: Supplementary file 2 [file Data_Sheet_1.ZIP › figures/Florida_totalprecaution_GT-eps-converted-to.pdf]

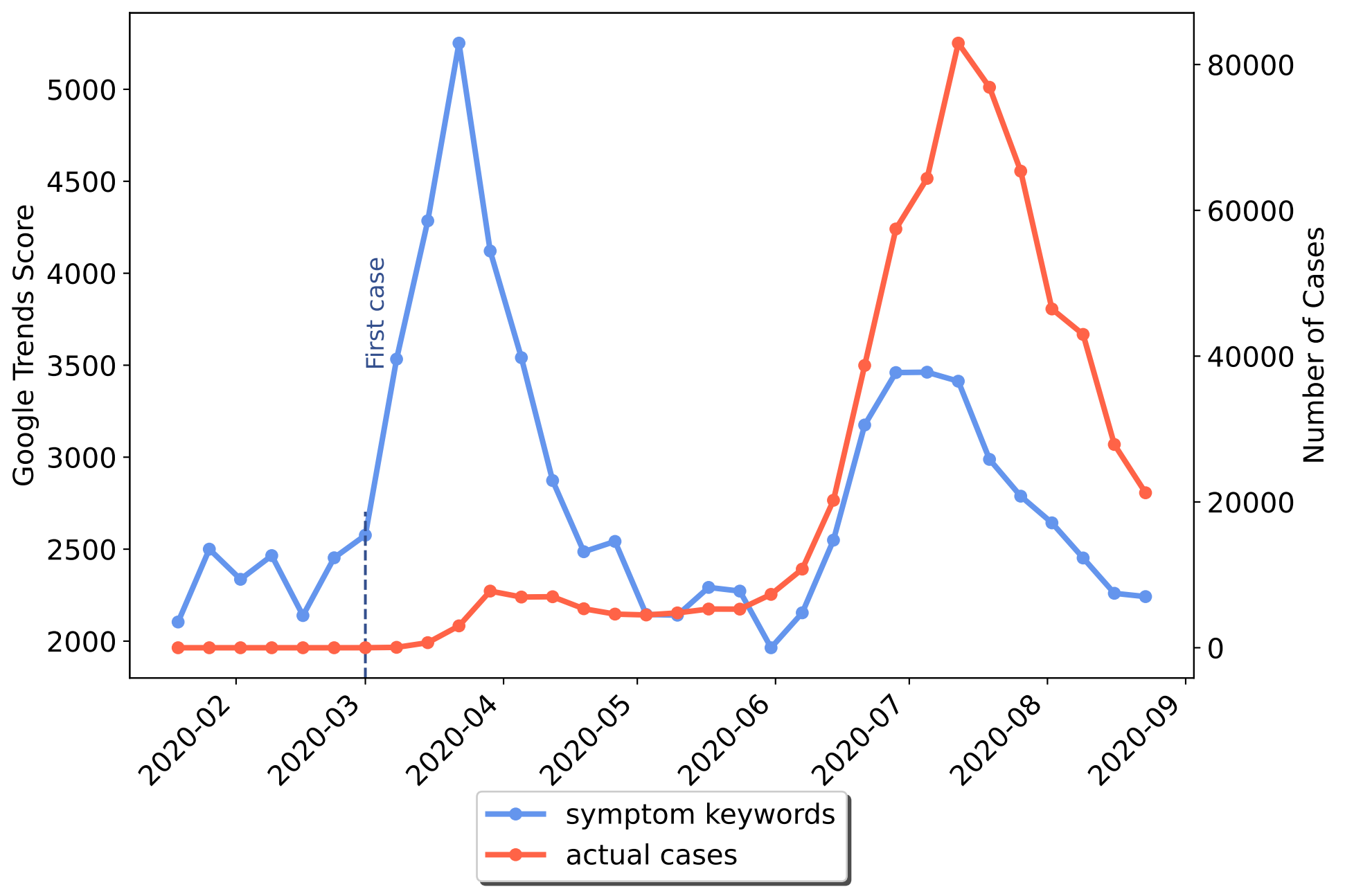

Supplement: Supplementary file 2 [file Data_Sheet_1.ZIP › figures/Florida_totalsymptom_GT-eps-converted-to.pdf]

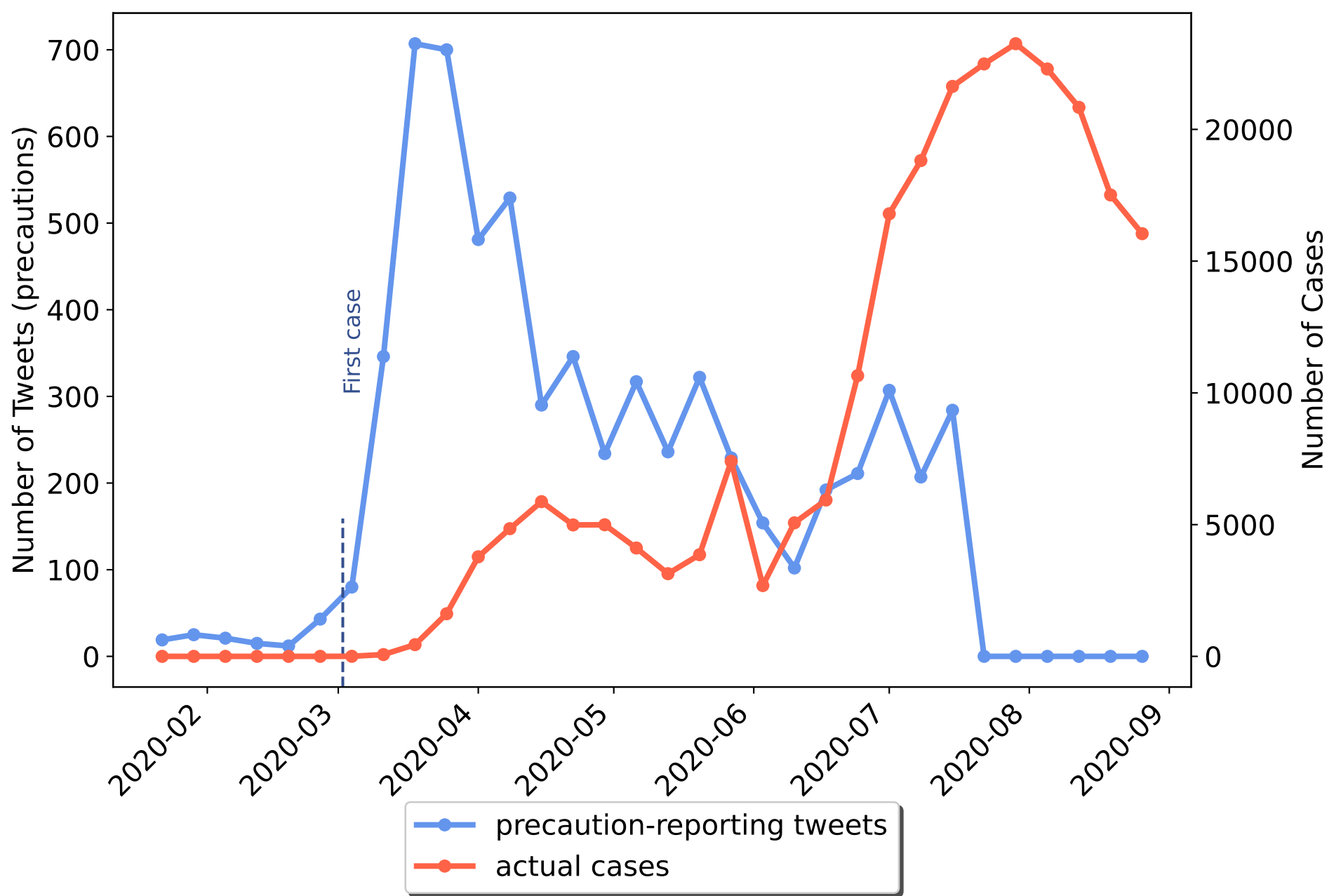

Supplement: Supplementary file 2 [file Data_Sheet_1.ZIP › figures/Georgia_precaution_twitter-eps-converted-to.pdf]

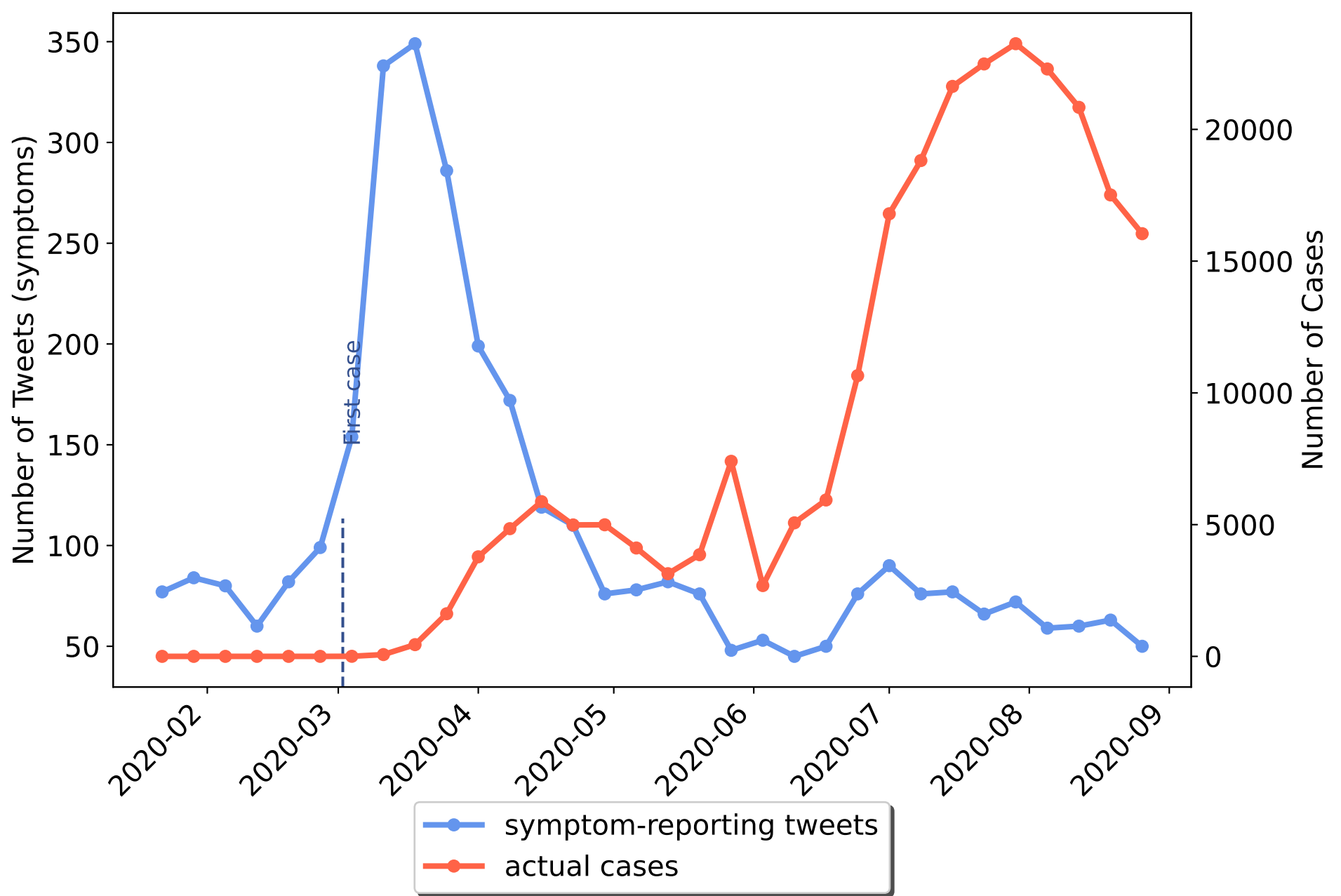

Supplement: Supplementary file 2 [file Data_Sheet_1.ZIP › figures/Georgia_symptom_twitter-eps-converted-to.pdf]

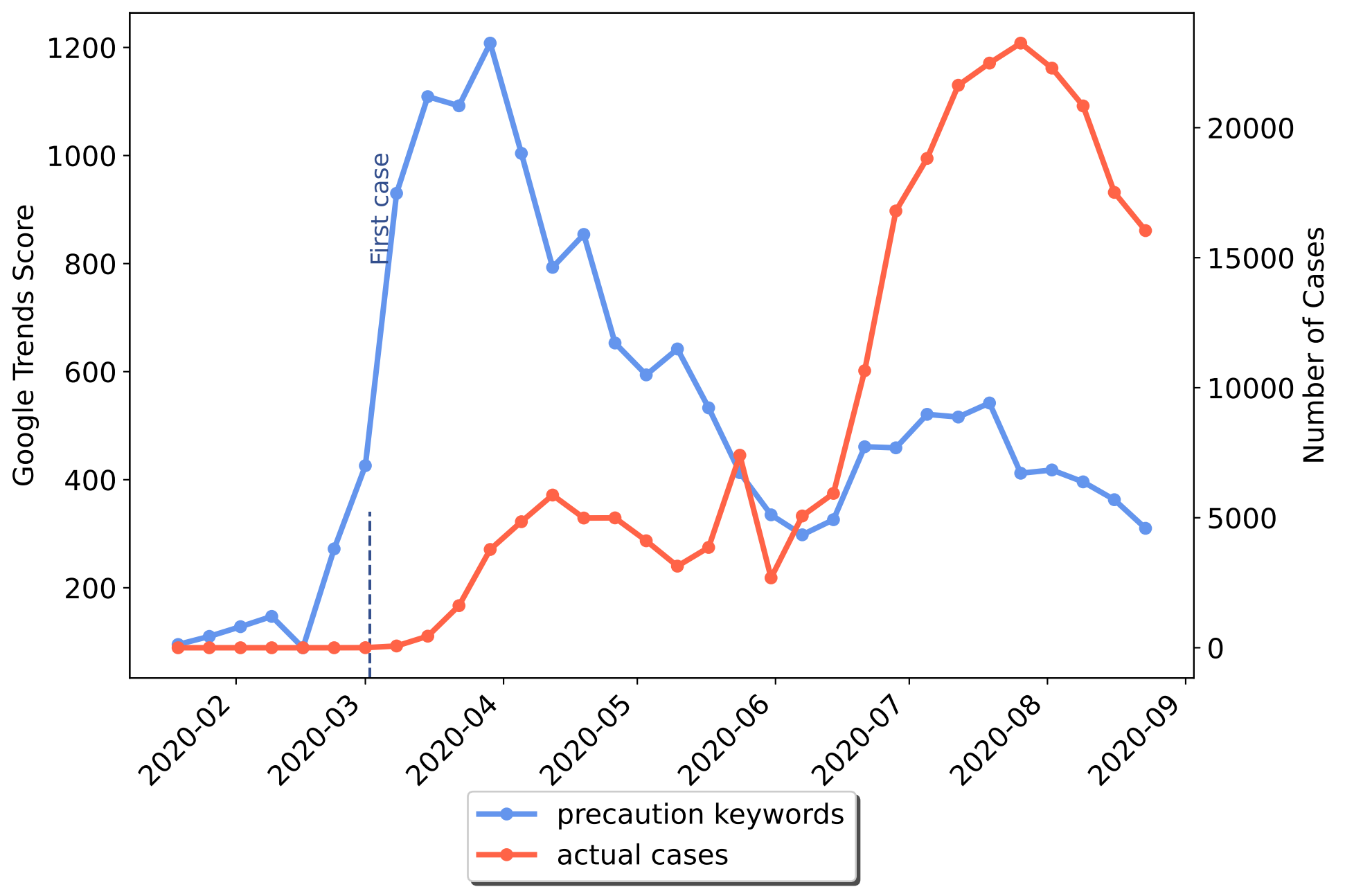

Supplement: Supplementary file 2 [file Data_Sheet_1.ZIP › figures/Georgia_totalprecaution_GT-eps-converted-to.pdf]

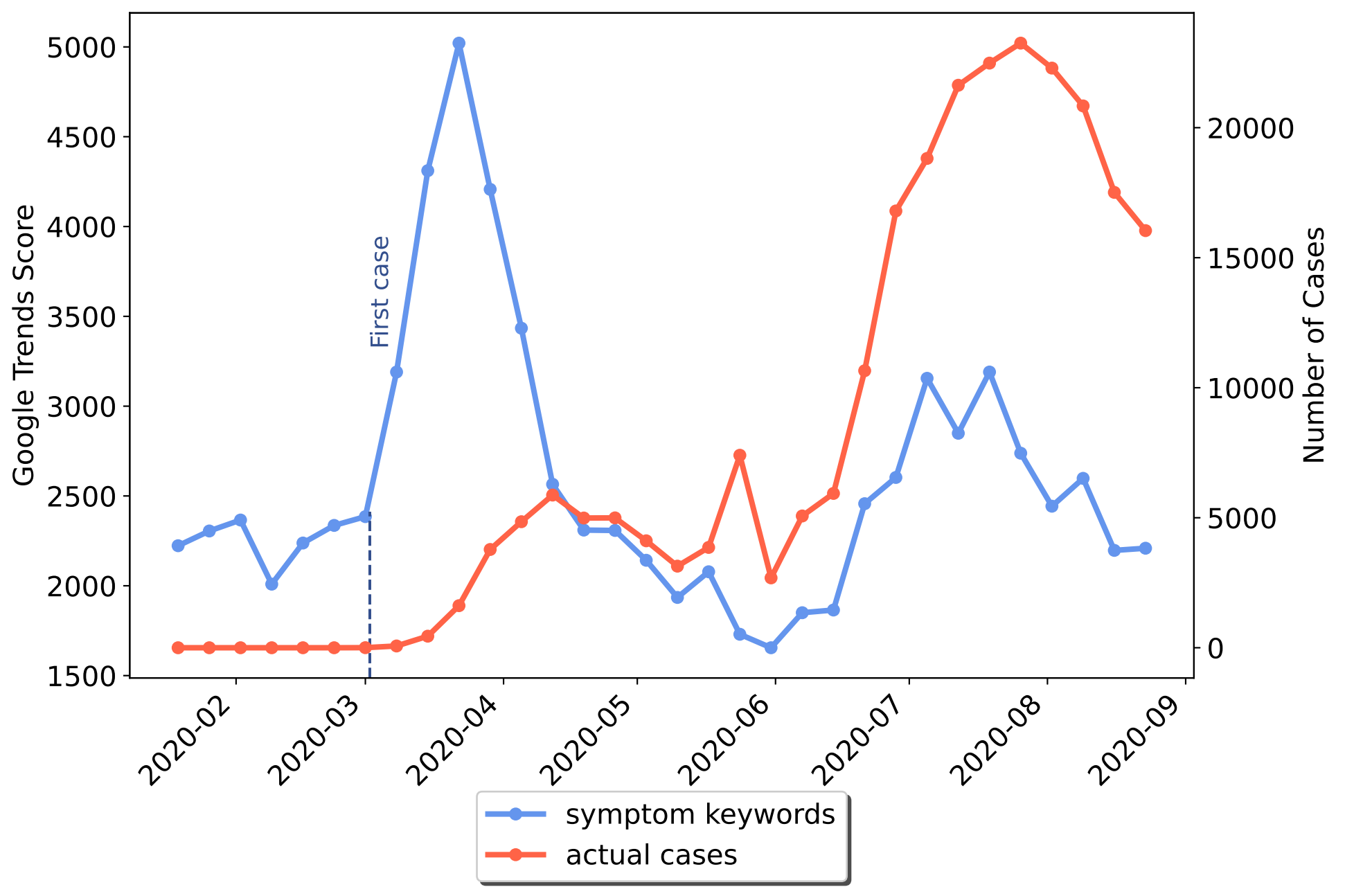

Supplement: Supplementary file 2 [file Data_Sheet_1.ZIP › figures/Georgia_totalsymptom_GT-eps-converted-to.pdf]

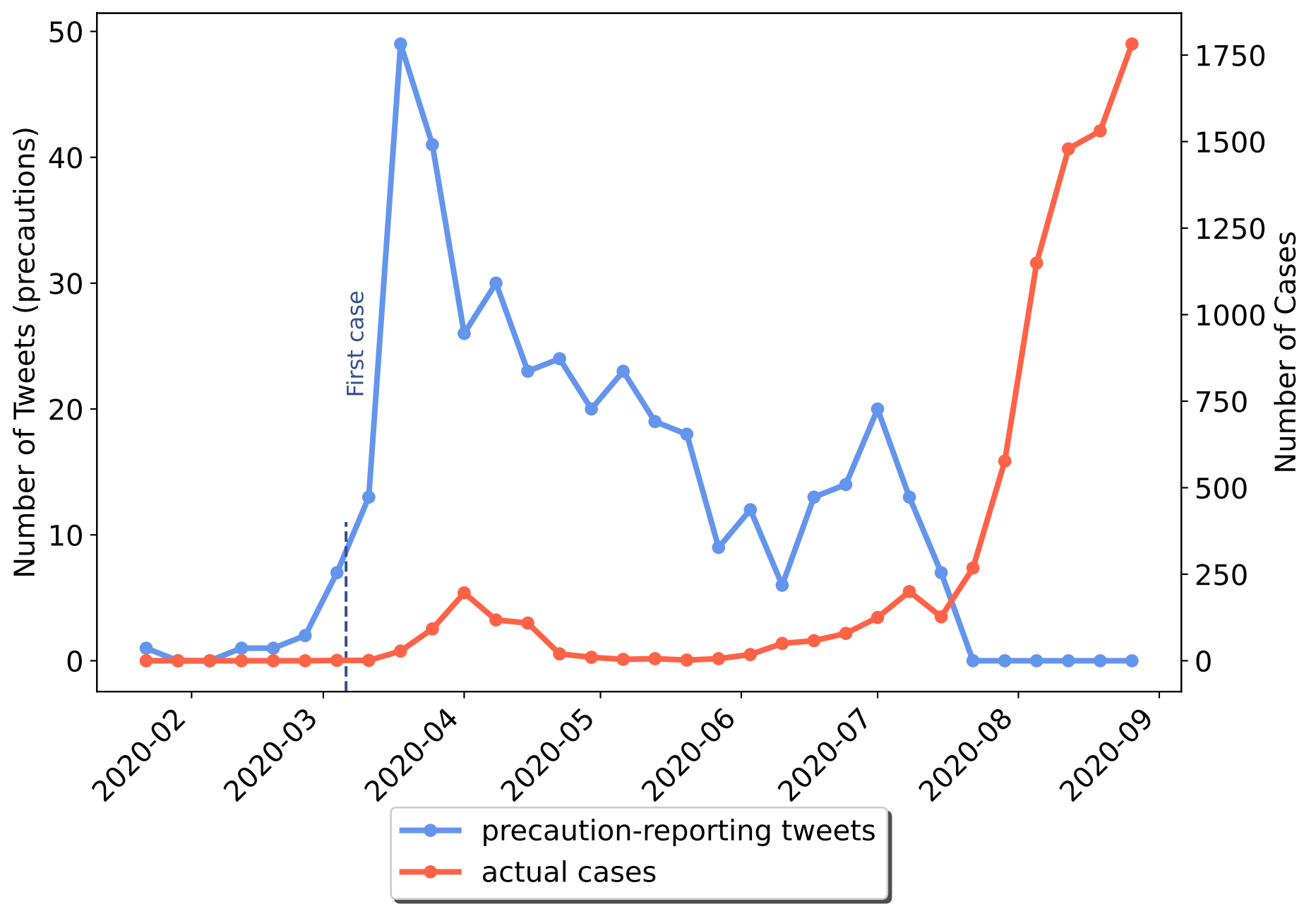

Supplement: Supplementary file 2 [file Data_Sheet_1.ZIP › figures/Hawaii_precaution_twitter-eps-converted-to.pdf]

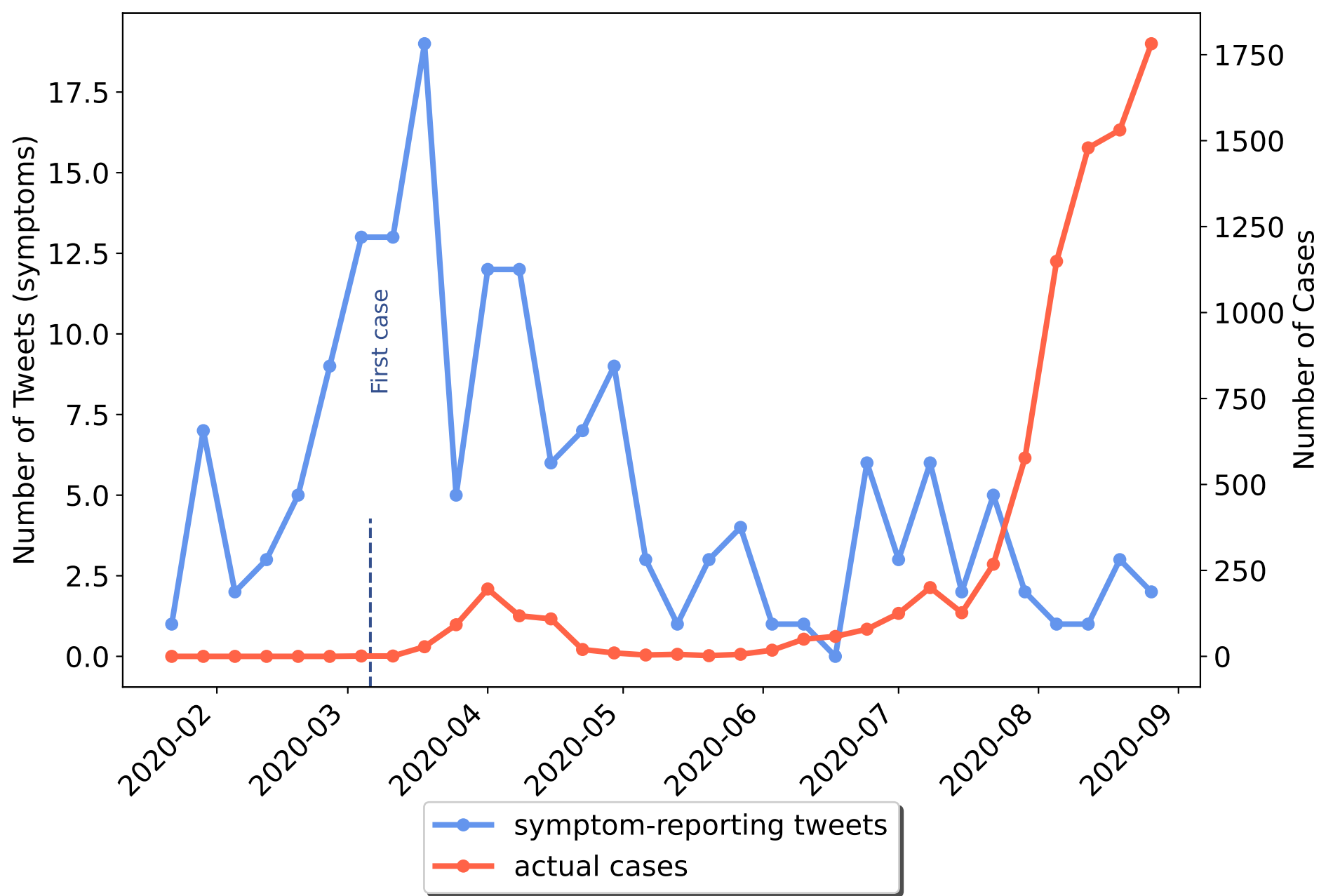

Supplement: Supplementary file 2 [file Data_Sheet_1.ZIP › figures/Hawaii_symptom_twitter-eps-converted-to.pdf]

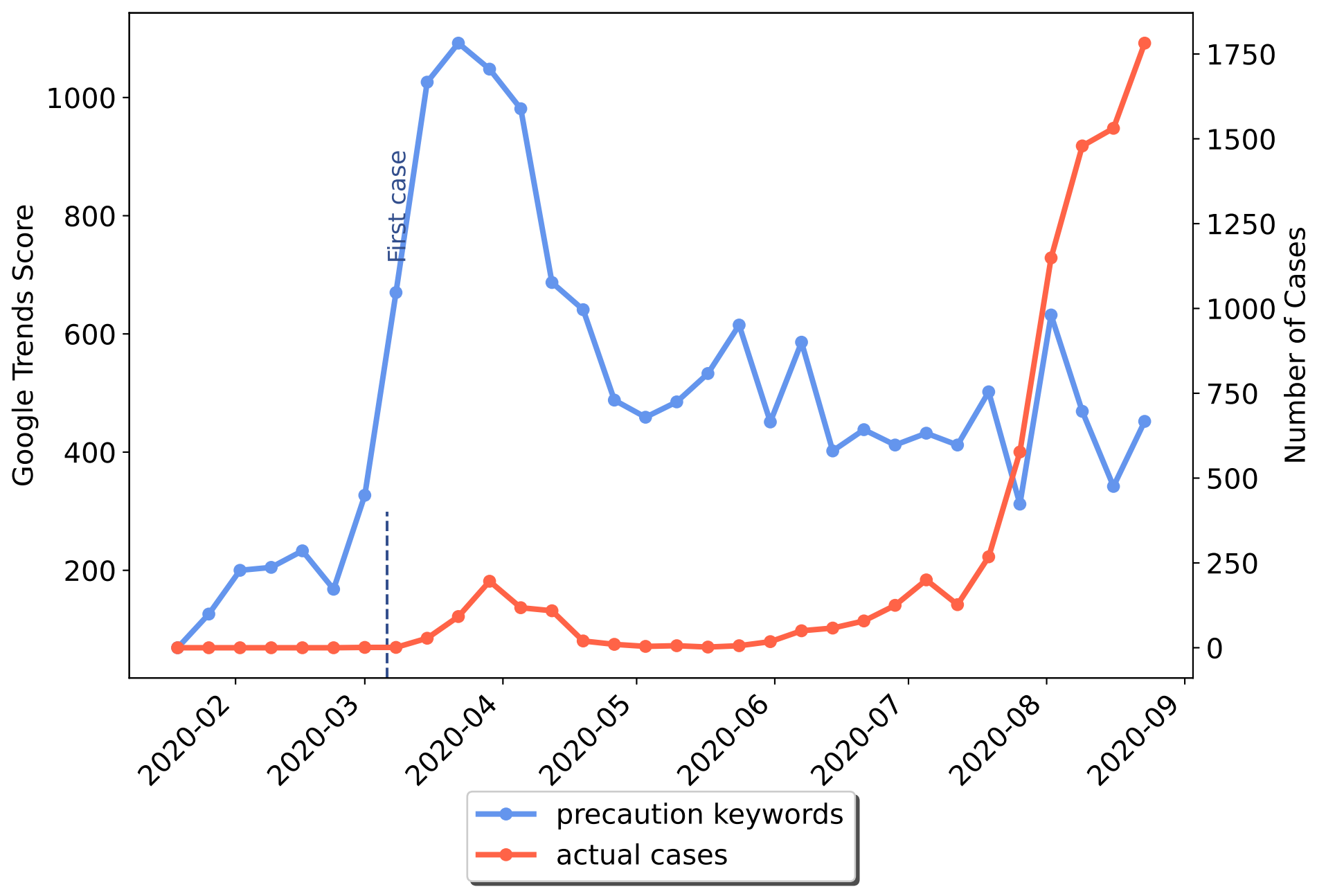

Supplement: Supplementary file 2 [file Data_Sheet_1.ZIP › figures/Hawaii_totalprecaution_GT-eps-converted-to.pdf]

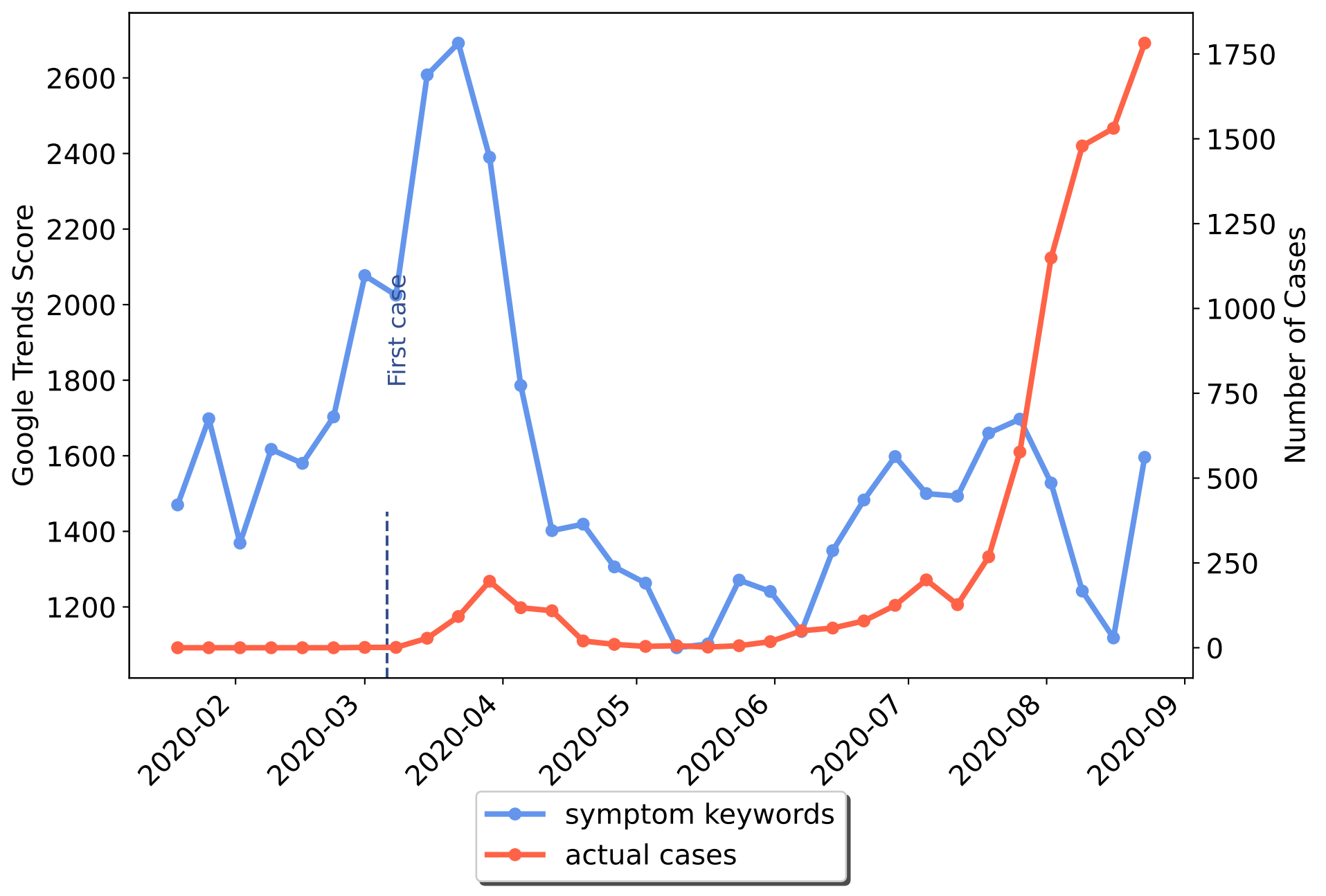

Supplement: Supplementary file 2 [file Data_Sheet_1.ZIP › figures/Hawaii_totalsymptom_GT-eps-converted-to.pdf]

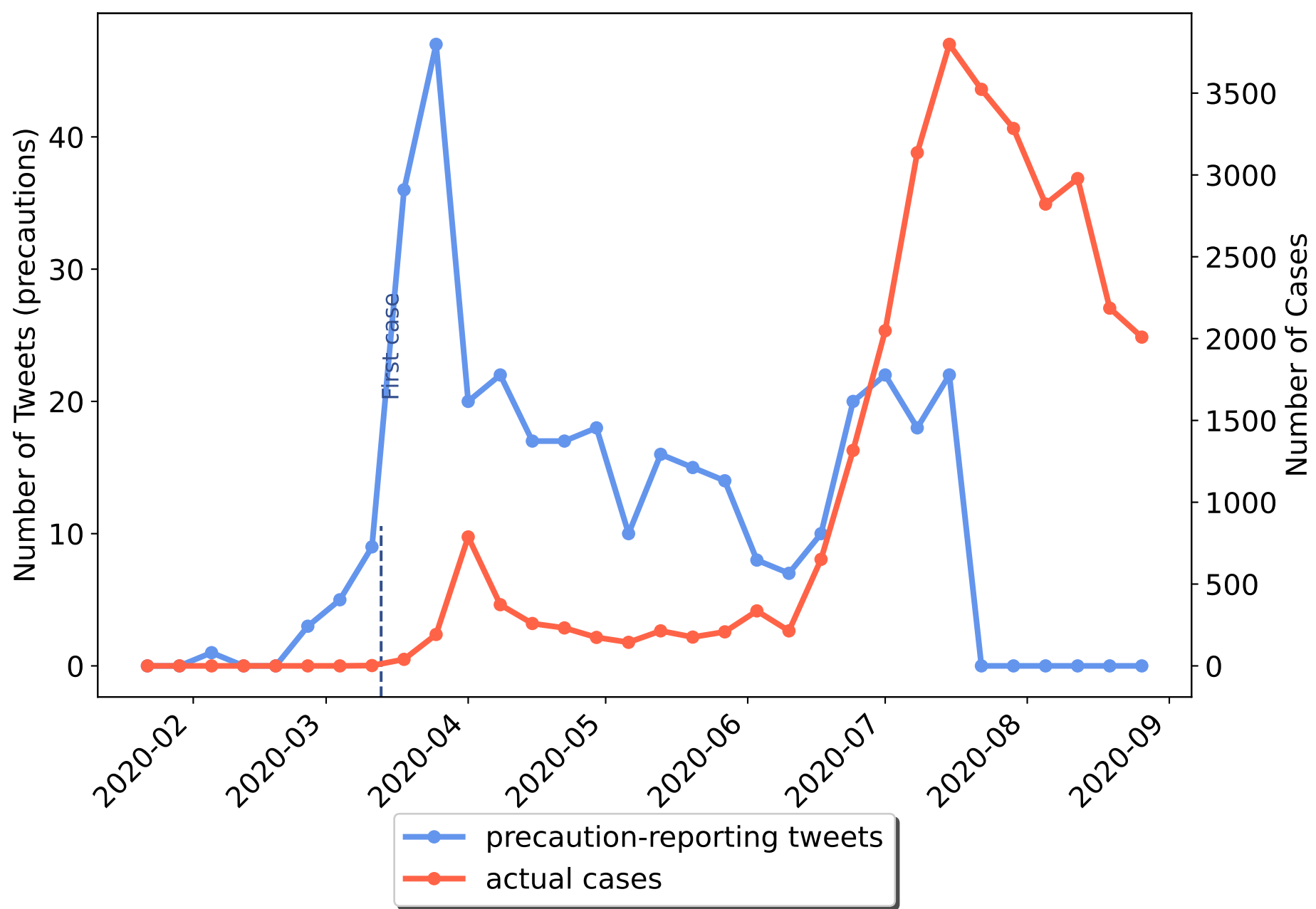

Supplement: Supplementary file 2 [file Data_Sheet_1.ZIP › figures/Idaho_precaution_twitter-eps-converted-to.pdf]

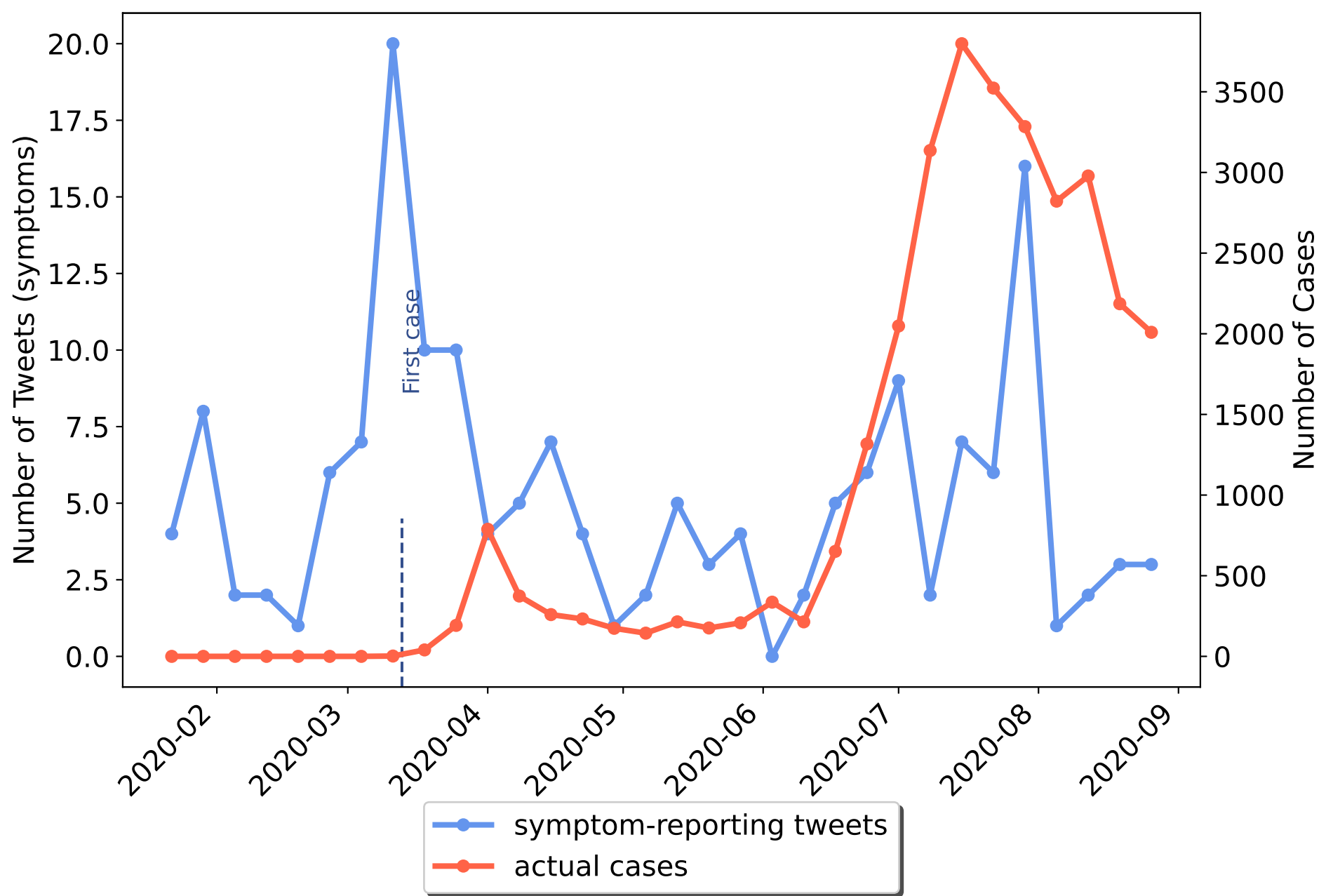

Supplement: Supplementary file 2 [file Data_Sheet_1.ZIP › figures/Idaho_symptom_twitter-eps-converted-to.pdf]

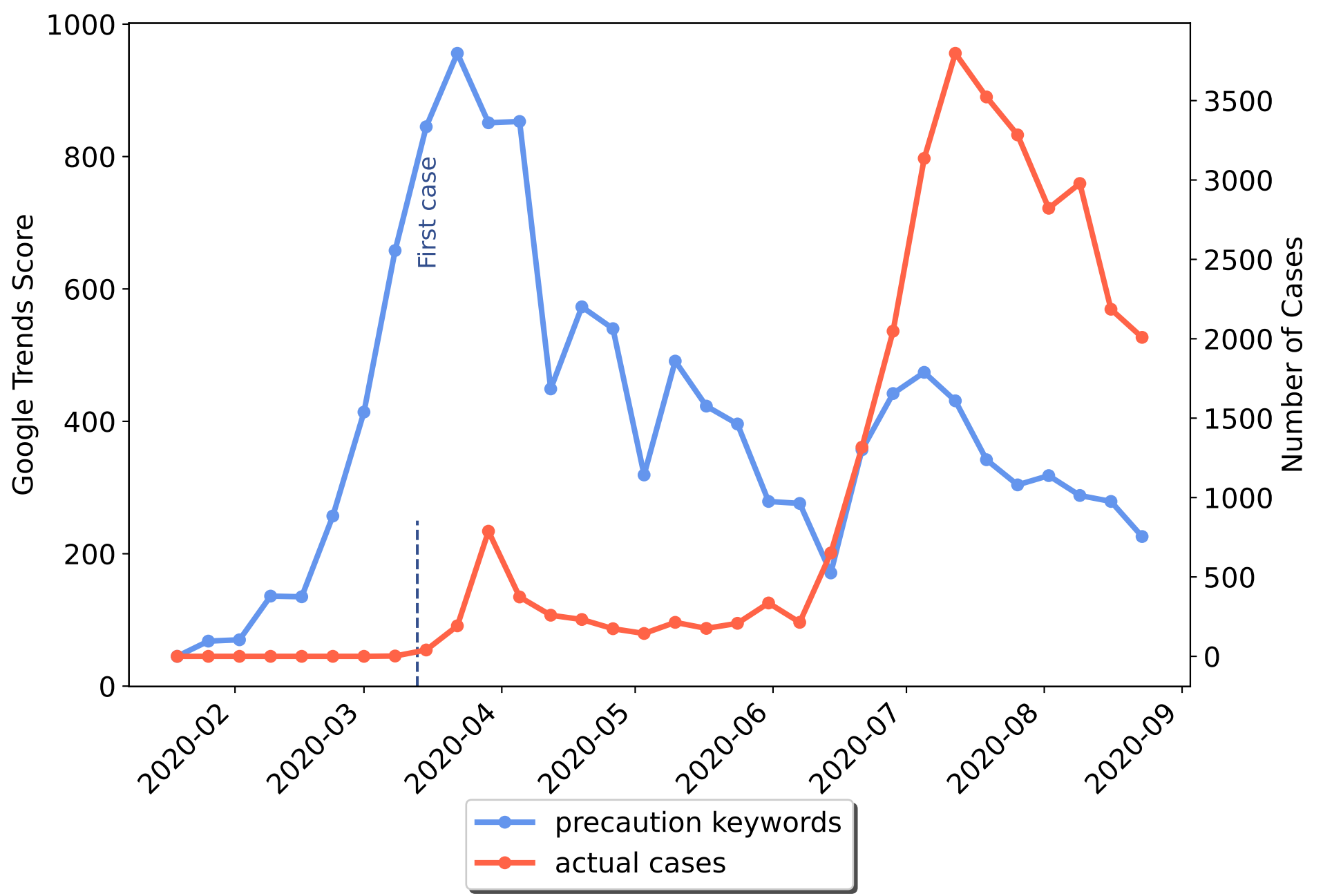

Supplement: Supplementary file 2 [file Data_Sheet_1.ZIP › figures/Idaho_totalprecaution_GT-eps-converted-to.pdf]

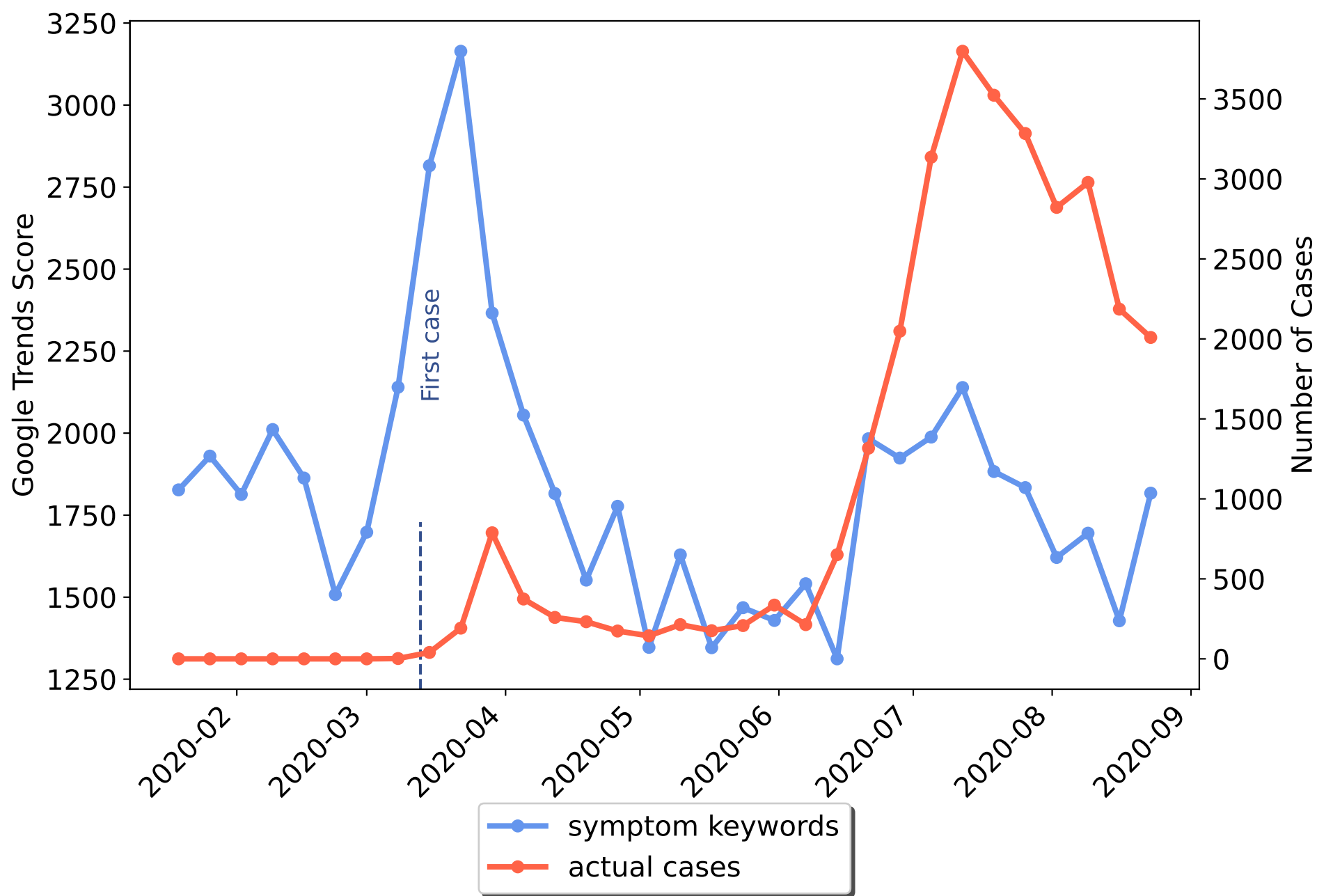

Supplement: Supplementary file 2 [file Data_Sheet_1.ZIP › figures/Idaho_totalsymptom_GT-eps-converted-to.pdf]

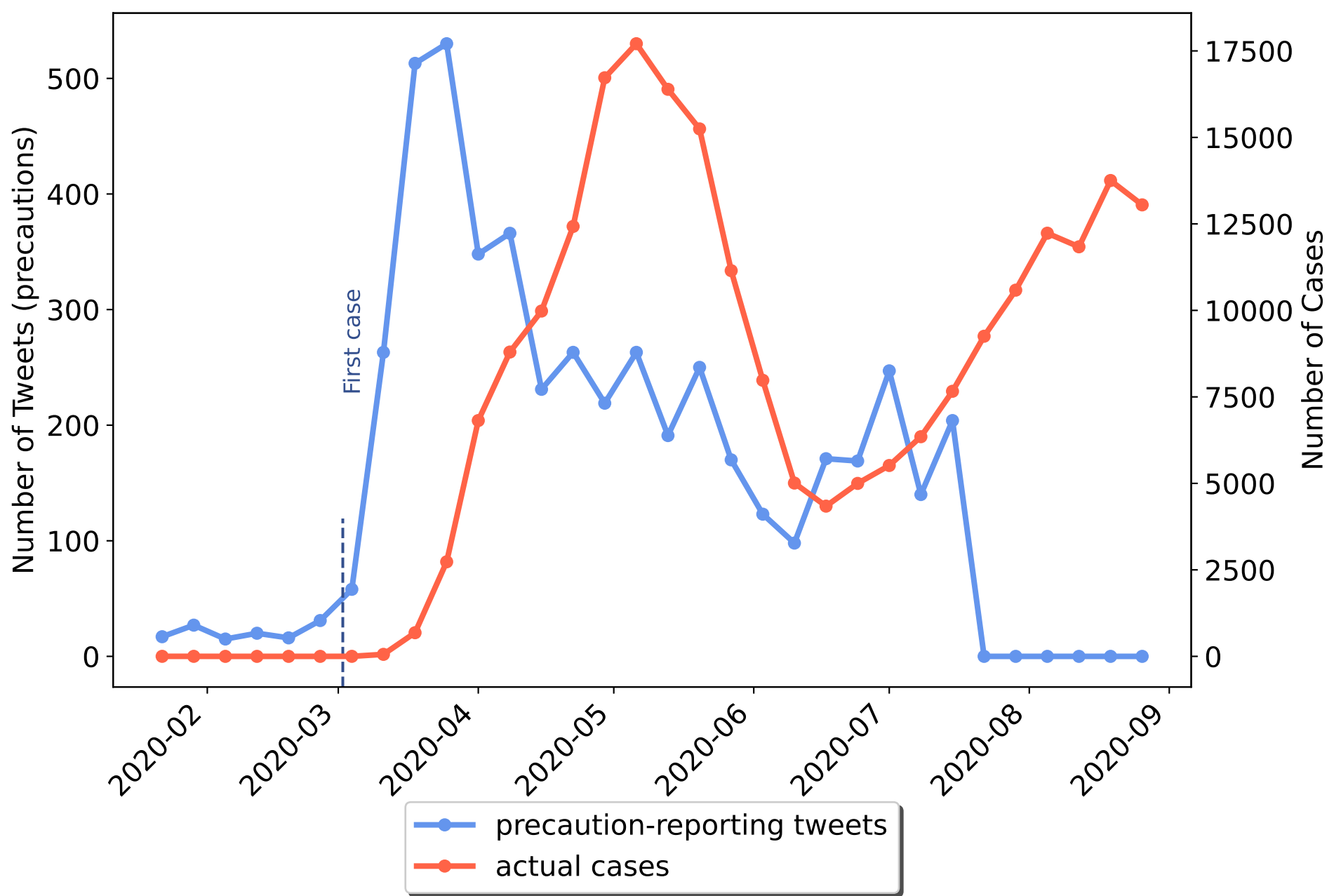

Supplement: Supplementary file 2 [file Data_Sheet_1.ZIP › figures/Illinois_precaution_twitter-eps-converted-to.pdf]

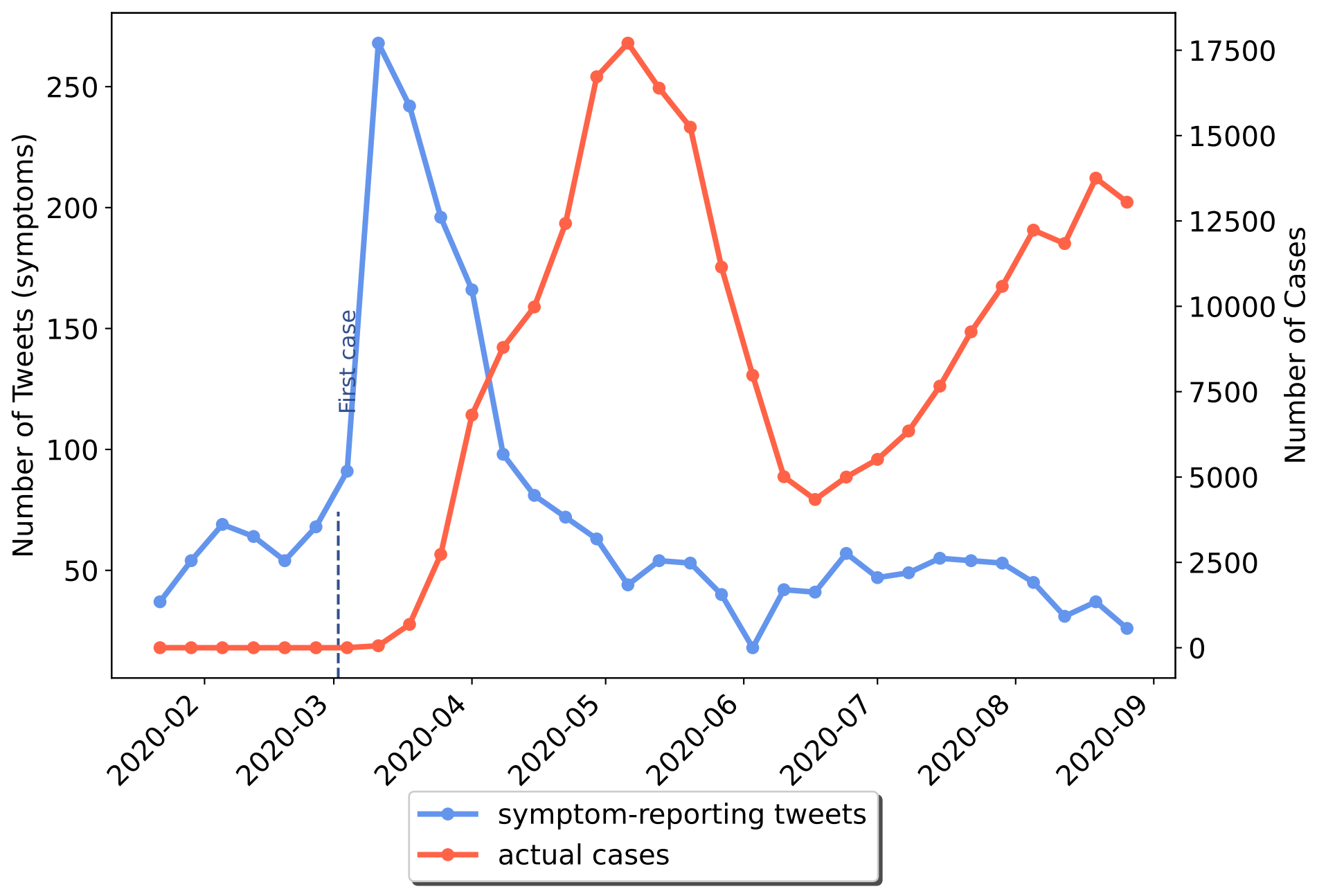

Supplement: Supplementary file 2 [file Data_Sheet_1.ZIP › figures/Illinois_symptom_twitter-eps-converted-to.pdf]

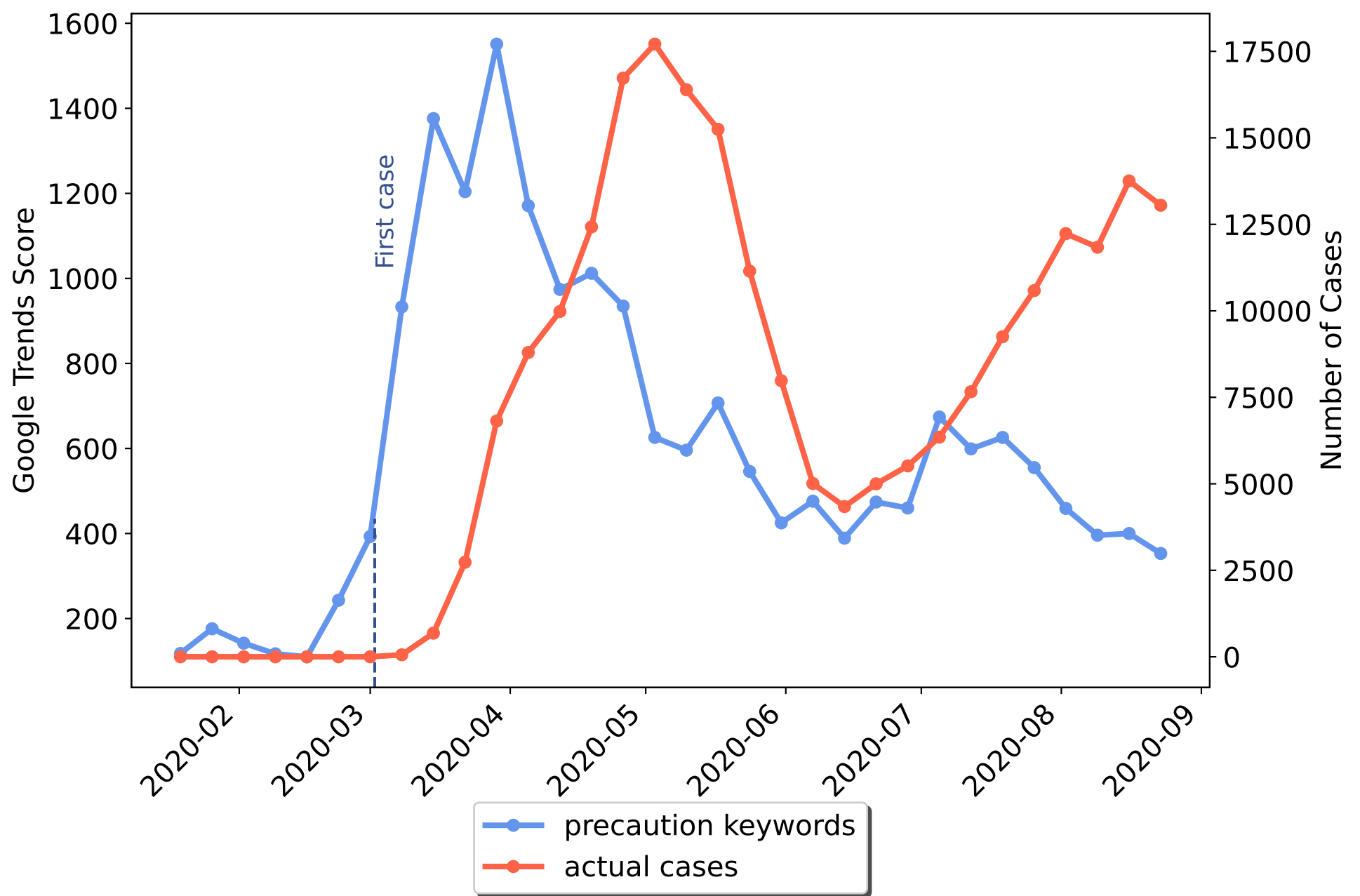

Supplement: Supplementary file 2 [file Data_Sheet_1.ZIP › figures/Illinois_totalprecaution_GT-eps-converted-to.pdf]

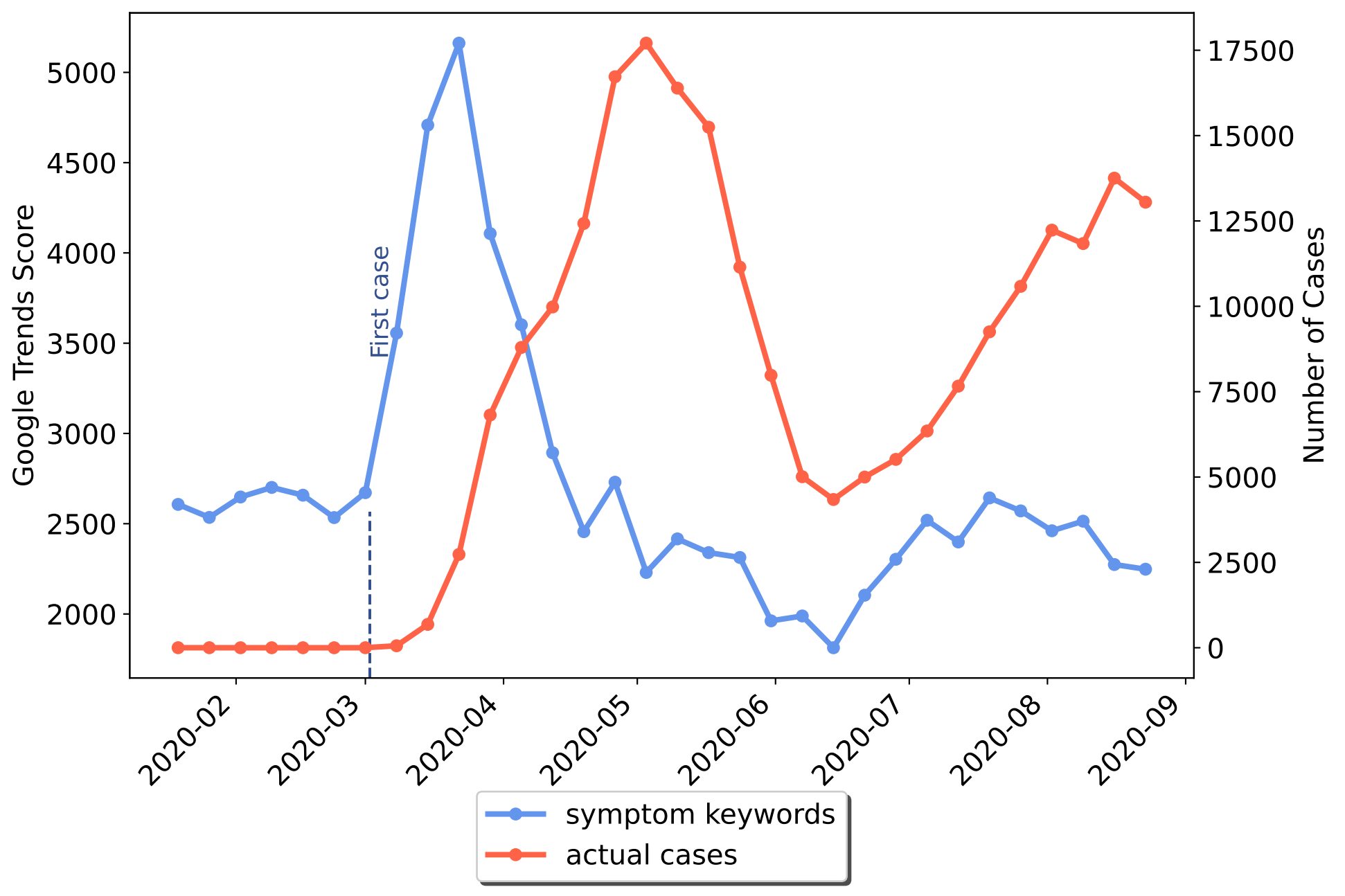

Supplement: Supplementary file 2 [file Data_Sheet_1.ZIP › figures/Illinois_totalsymptom_GT-eps-converted-to.pdf]

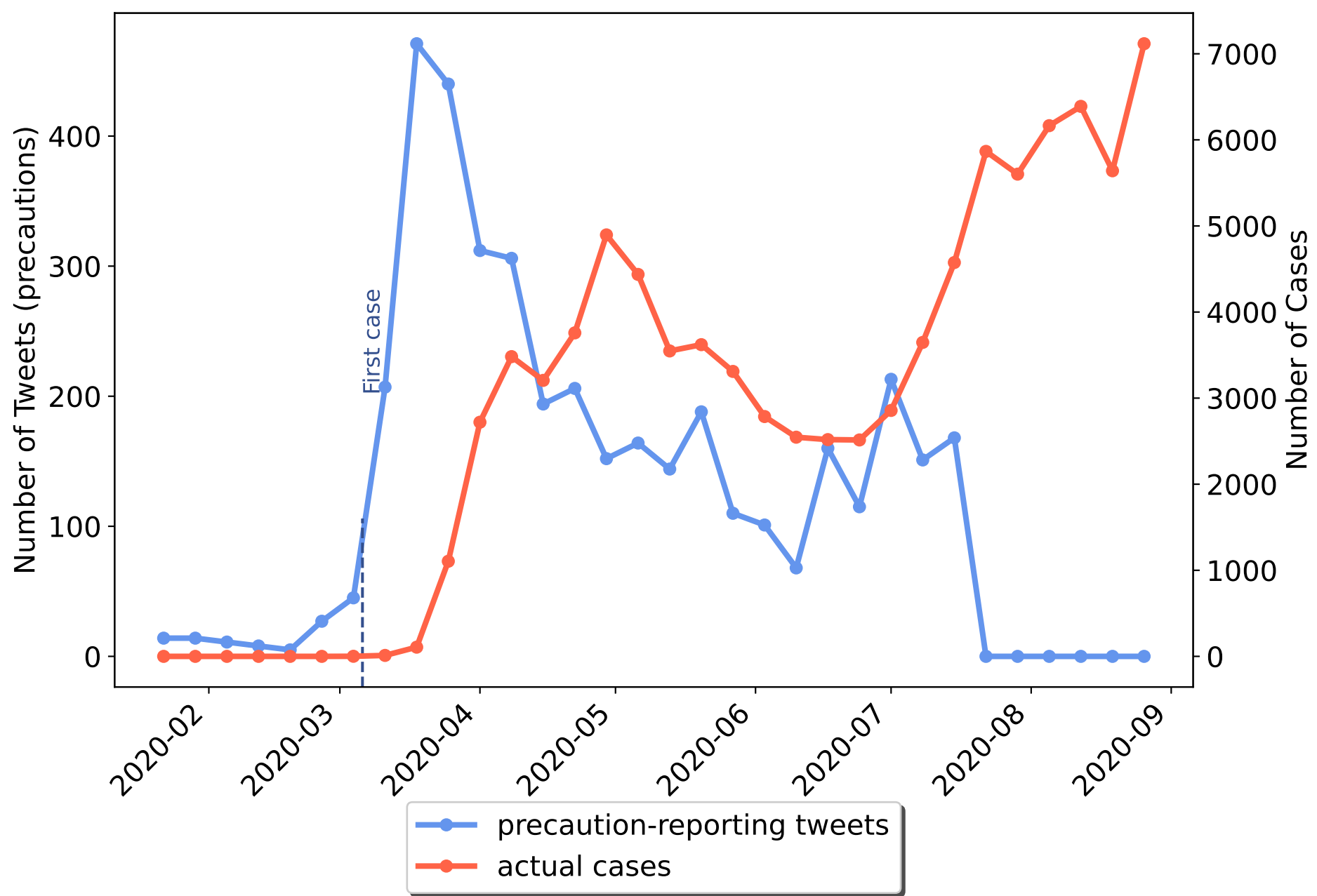

Supplement: Supplementary file 2 [file Data_Sheet_1.ZIP › figures/Indiana_precaution_twitter-eps-converted-to.pdf]

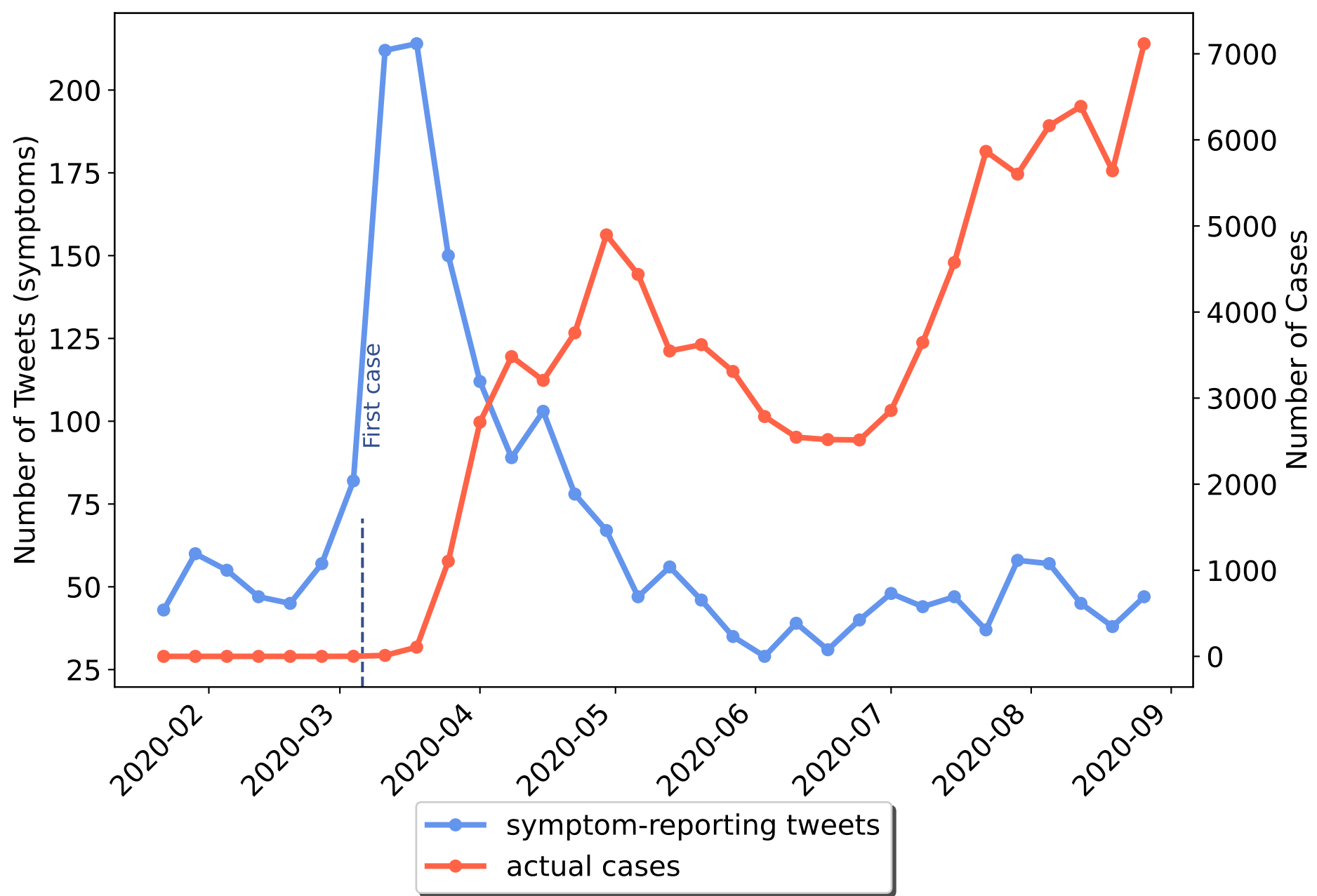

Supplement: Supplementary file 2 [file Data_Sheet_1.ZIP › figures/Indiana_symptom_twitter-eps-converted-to.pdf]

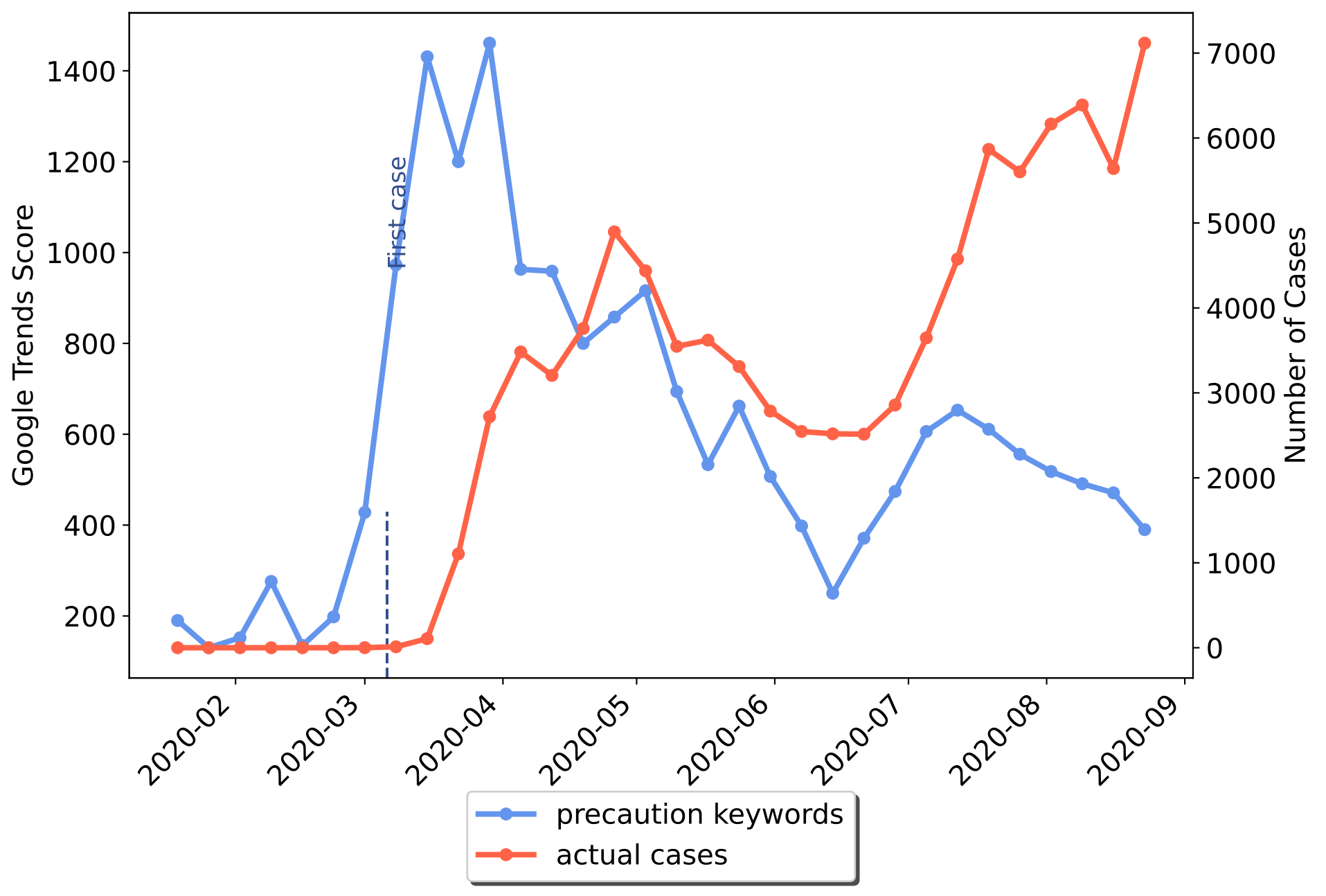

Supplement: Supplementary file 2 [file Data_Sheet_1.ZIP › figures/Indiana_totalprecaution_GT-eps-converted-to.pdf]

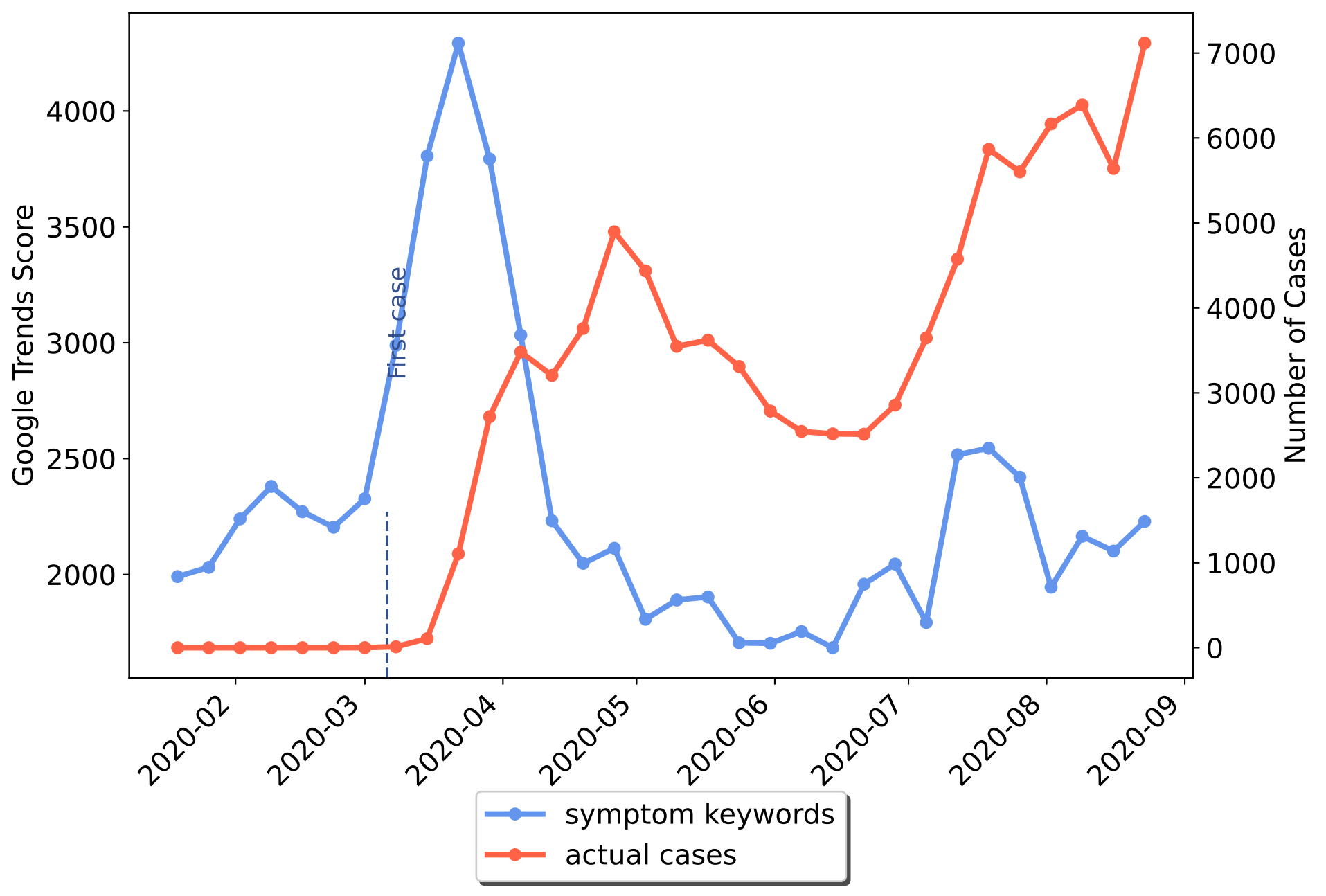

Supplement: Supplementary file 2 [file Data_Sheet_1.ZIP › figures/Indiana_totalsymptom_GT-eps-converted-to.pdf]

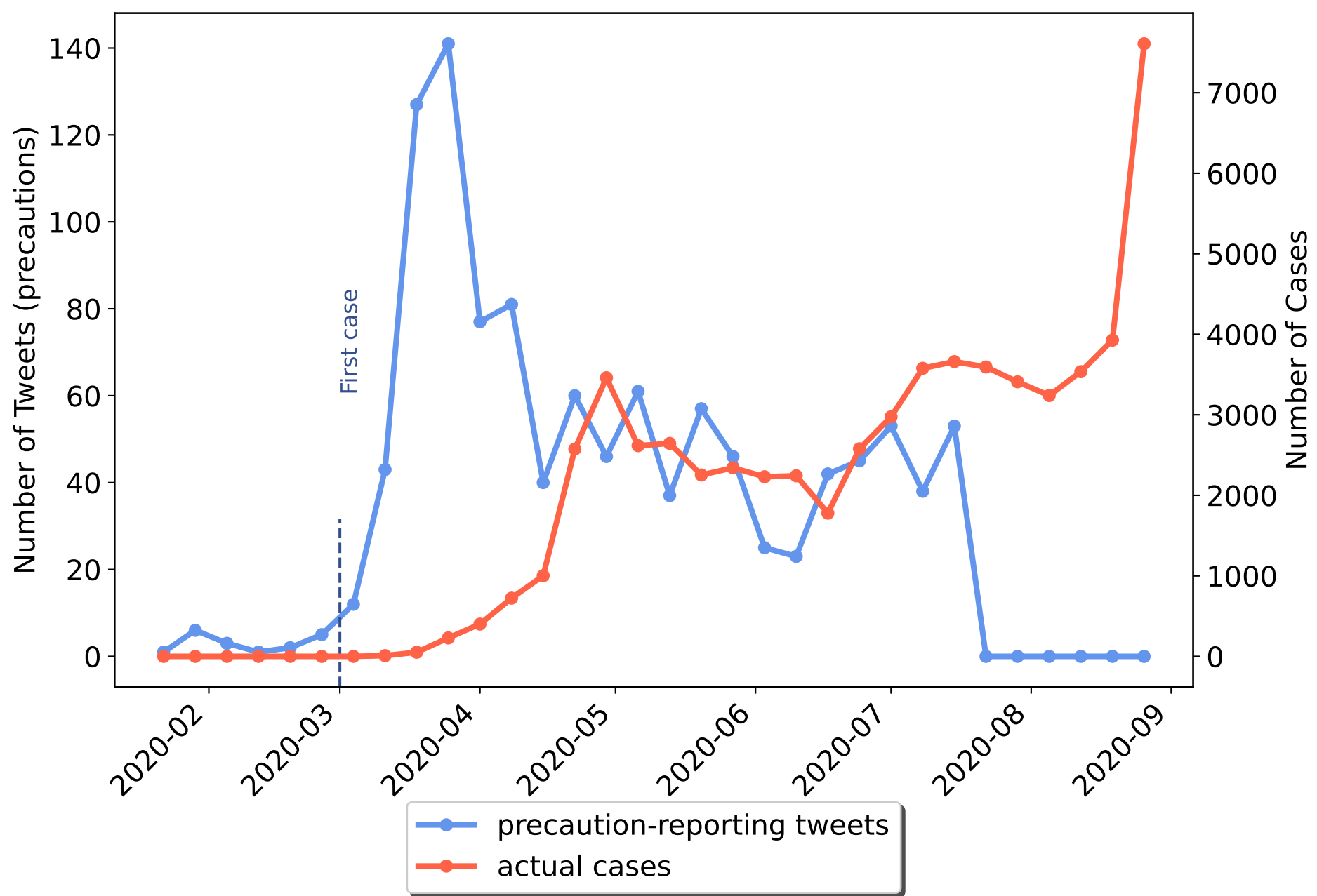

Supplement: Supplementary file 2 [file Data_Sheet_1.ZIP › figures/Iowa_precaution_twitter-eps-converted-to.pdf]

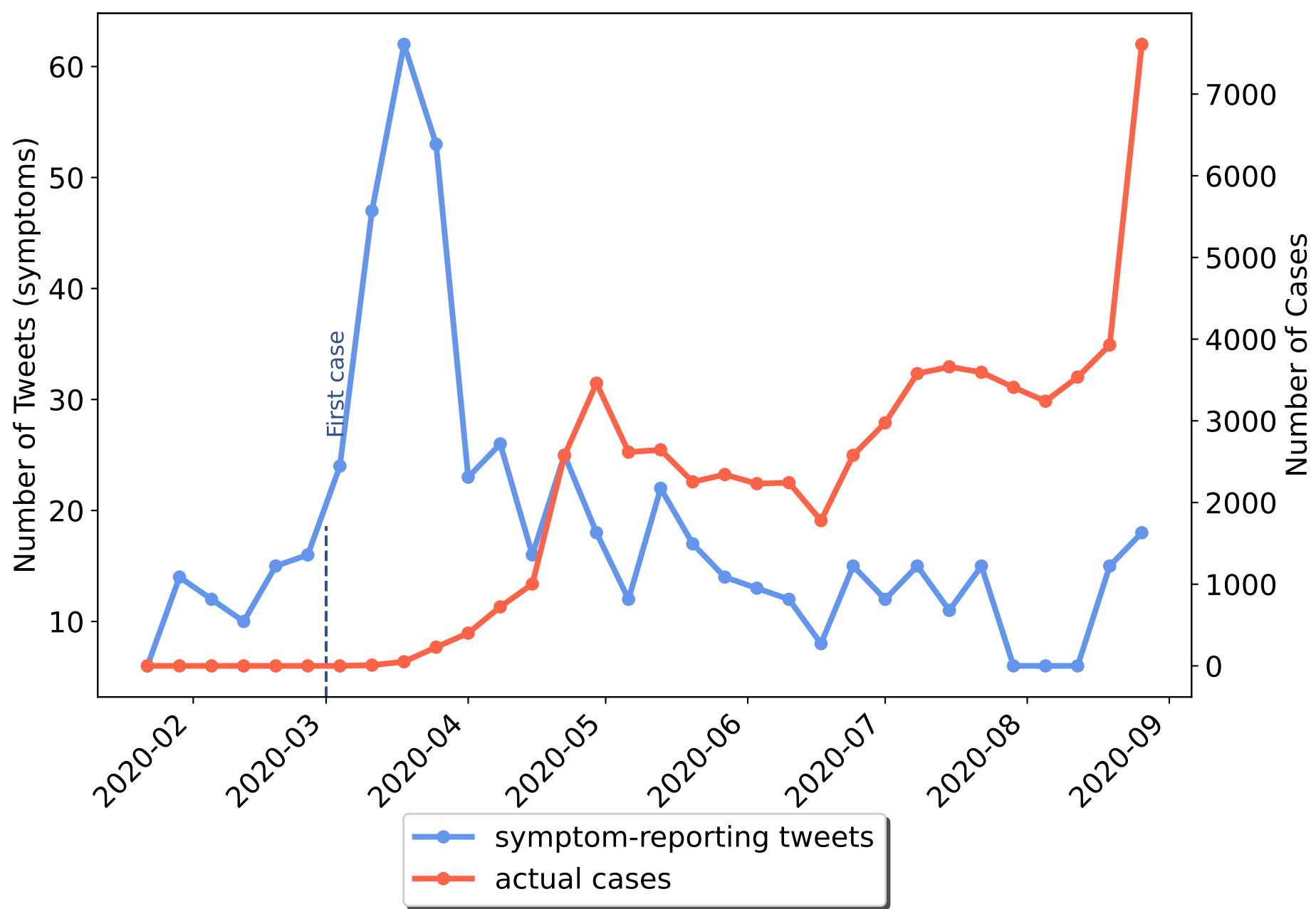

Supplement: Supplementary file 2 [file Data_Sheet_1.ZIP › figures/Iowa_symptom_twitter-eps-converted-to.pdf]

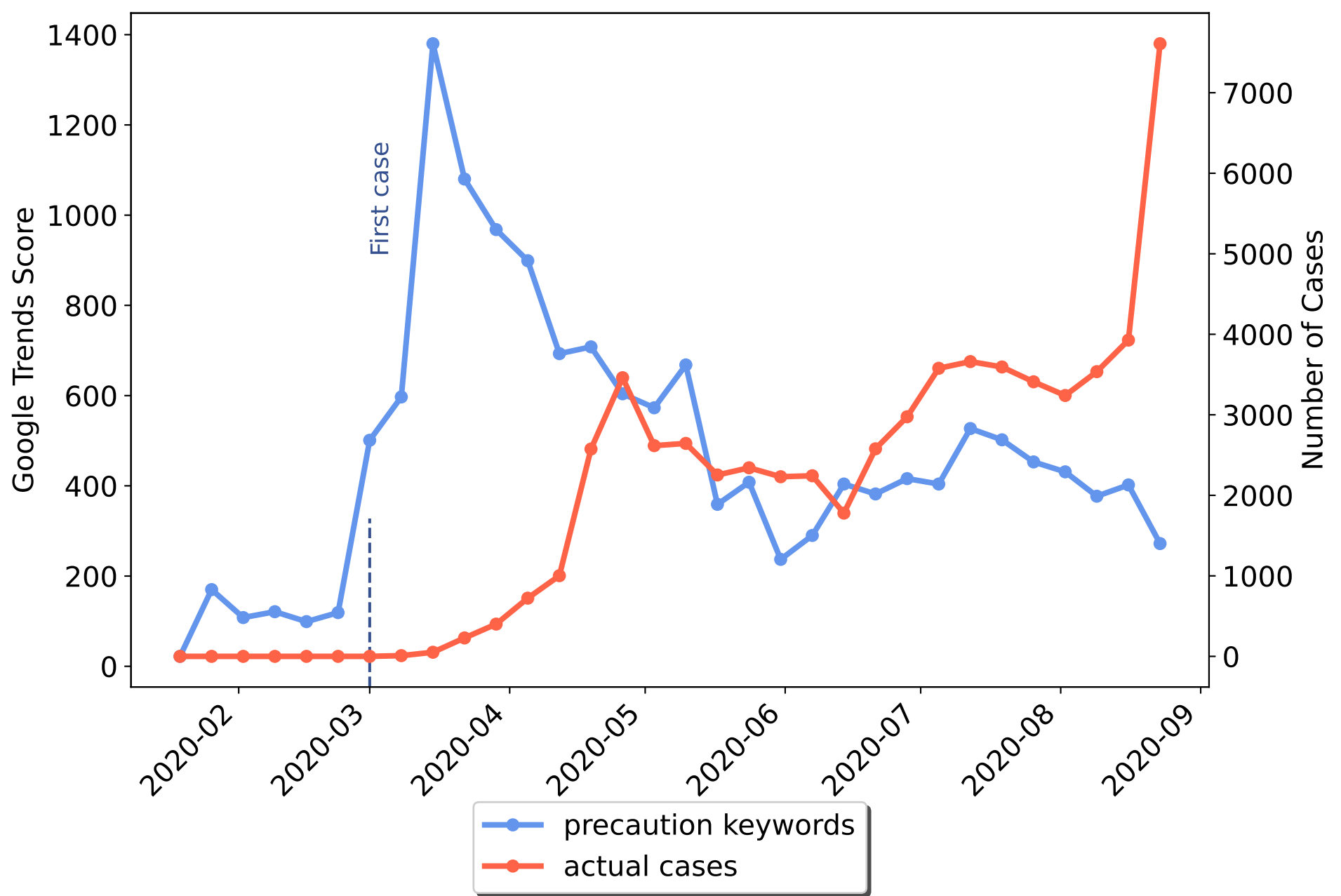

Supplement: Supplementary file 2 [file Data_Sheet_1.ZIP › figures/Iowa_totalprecaution_GT-eps-converted-to.pdf]

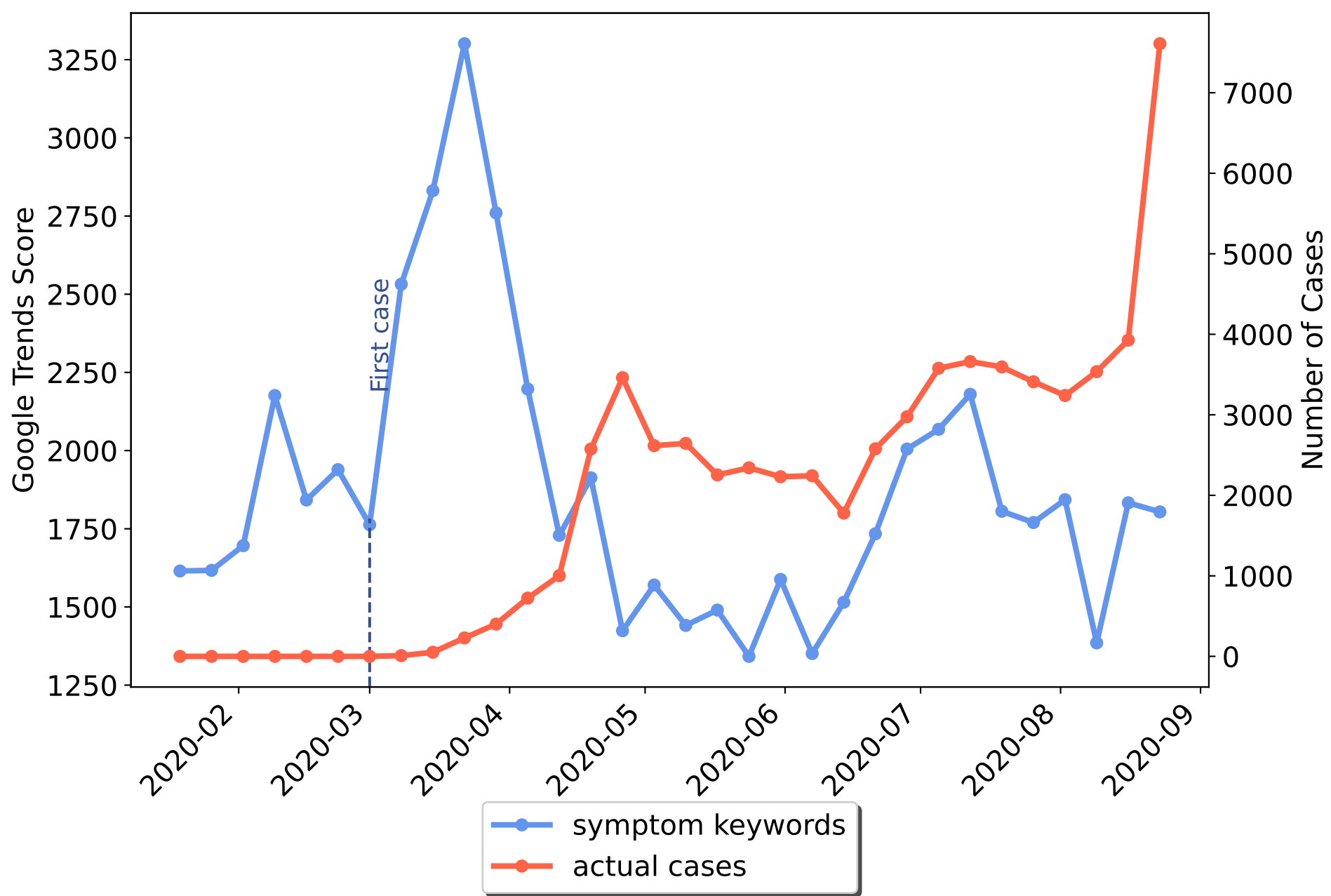

Supplement: Supplementary file 2 [file Data_Sheet_1.ZIP › figures/Iowa_totalsymptom_GT-eps-converted-to.pdf]

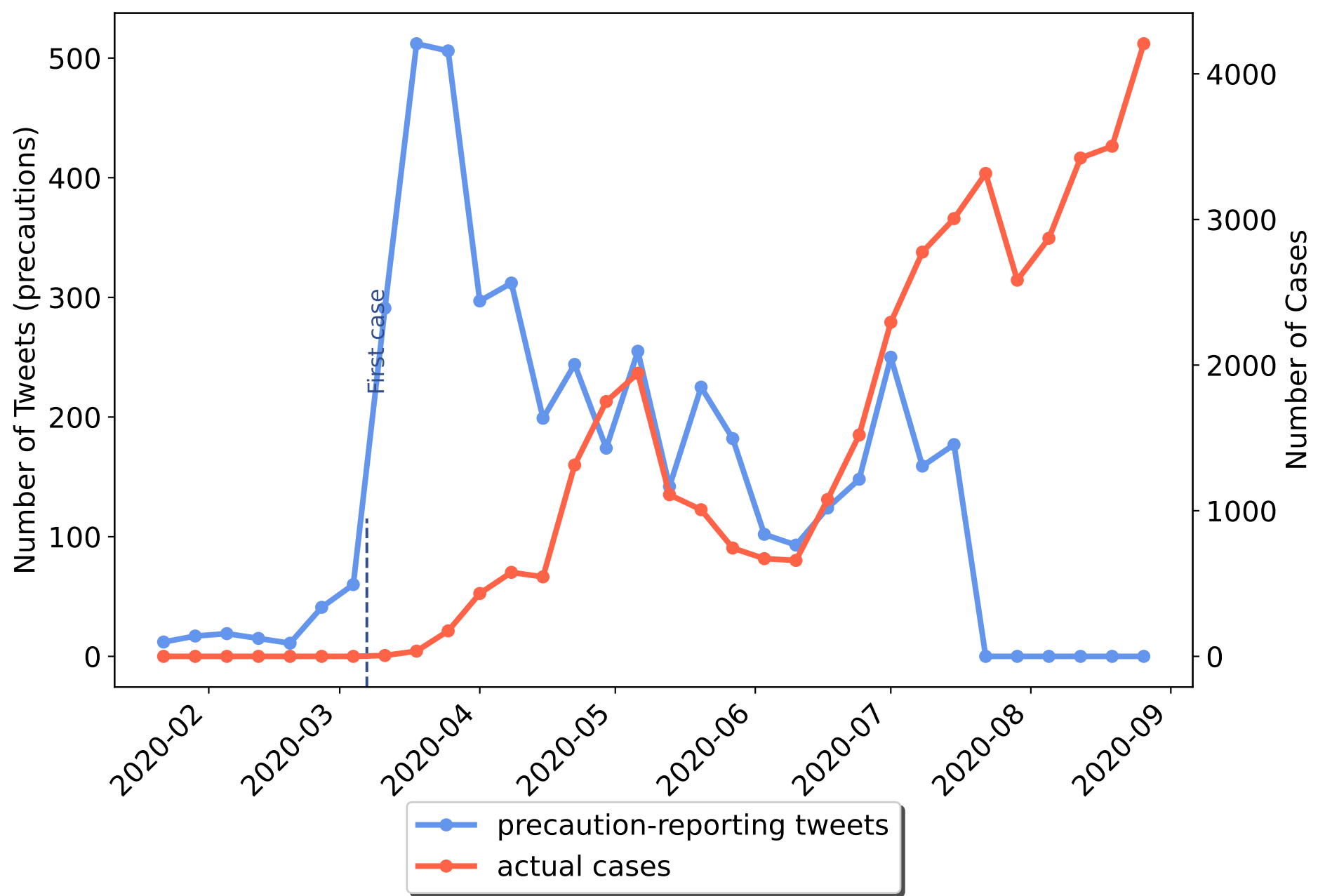

Supplement: Supplementary file 2 [file Data_Sheet_1.ZIP › figures/Kansas_precaution_twitter-eps-converted-to.pdf]

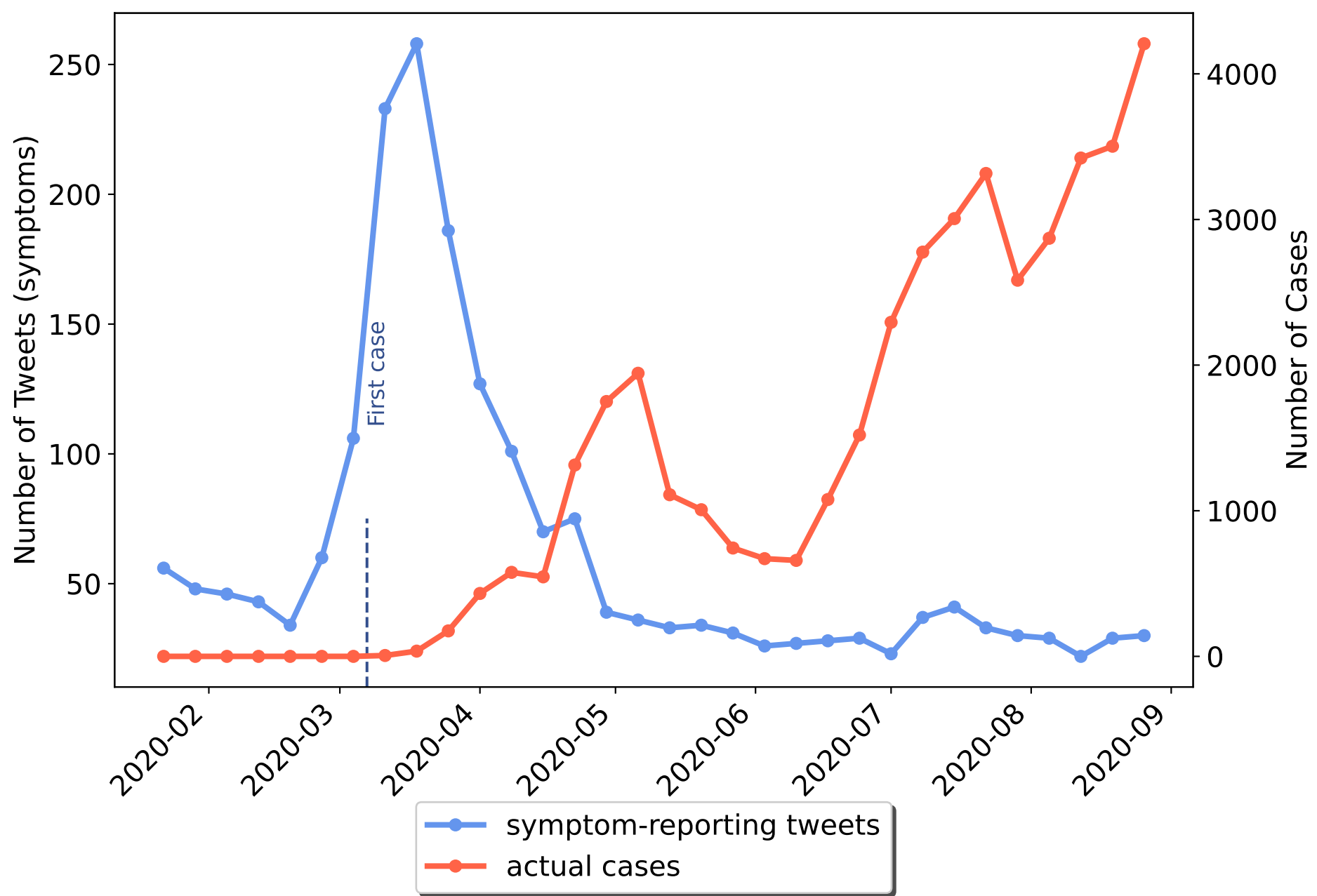

Supplement: Supplementary file 2 [file Data_Sheet_1.ZIP › figures/Kansas_symptom_twitter-eps-converted-to.pdf]

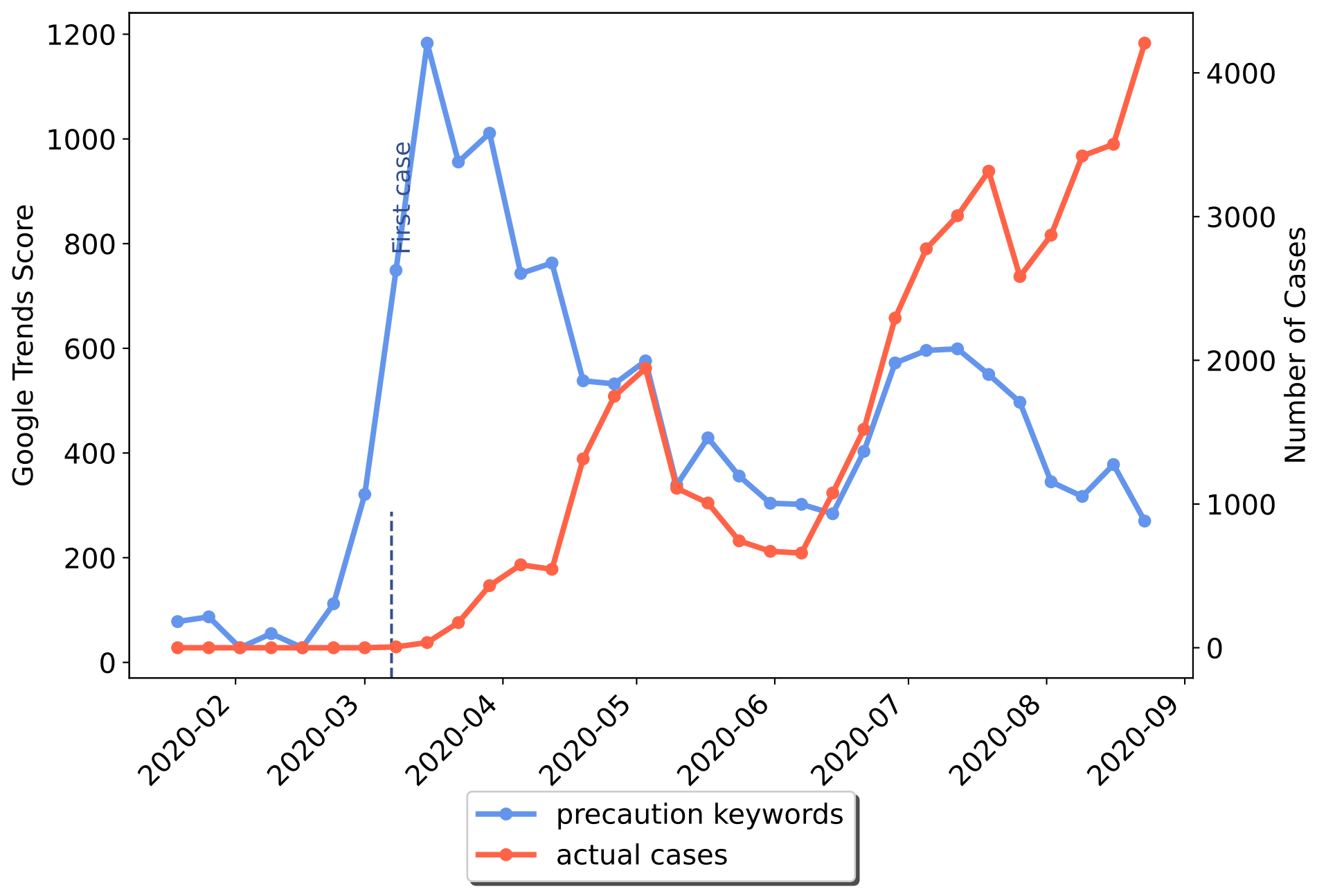

Supplement: Supplementary file 2 [file Data_Sheet_1.ZIP › figures/Kansas_totalprecaution_GT-eps-converted-to.pdf]

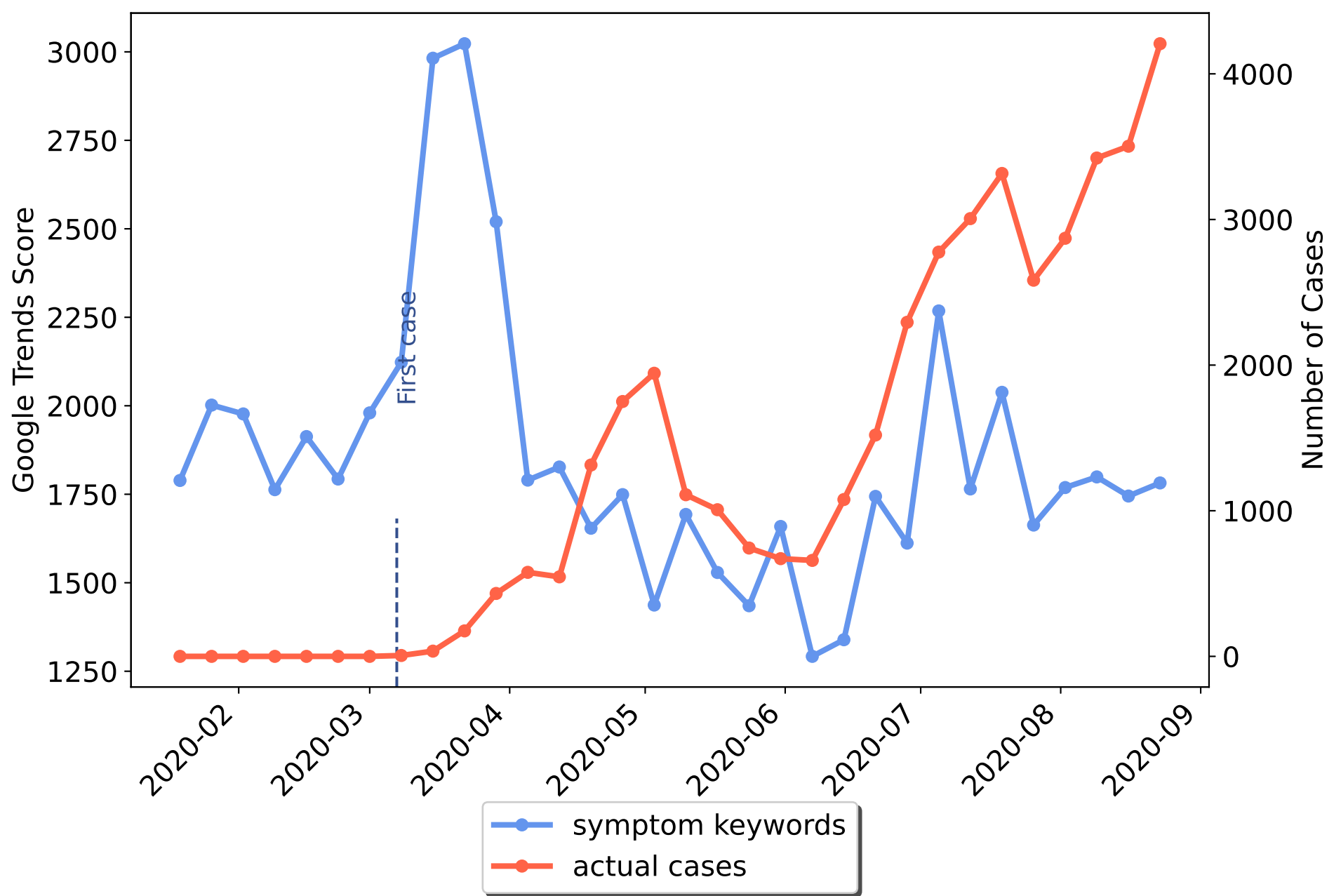

Supplement: Supplementary file 2 [file Data_Sheet_1.ZIP › figures/Kansas_totalsymptom_GT-eps-converted-to.pdf]

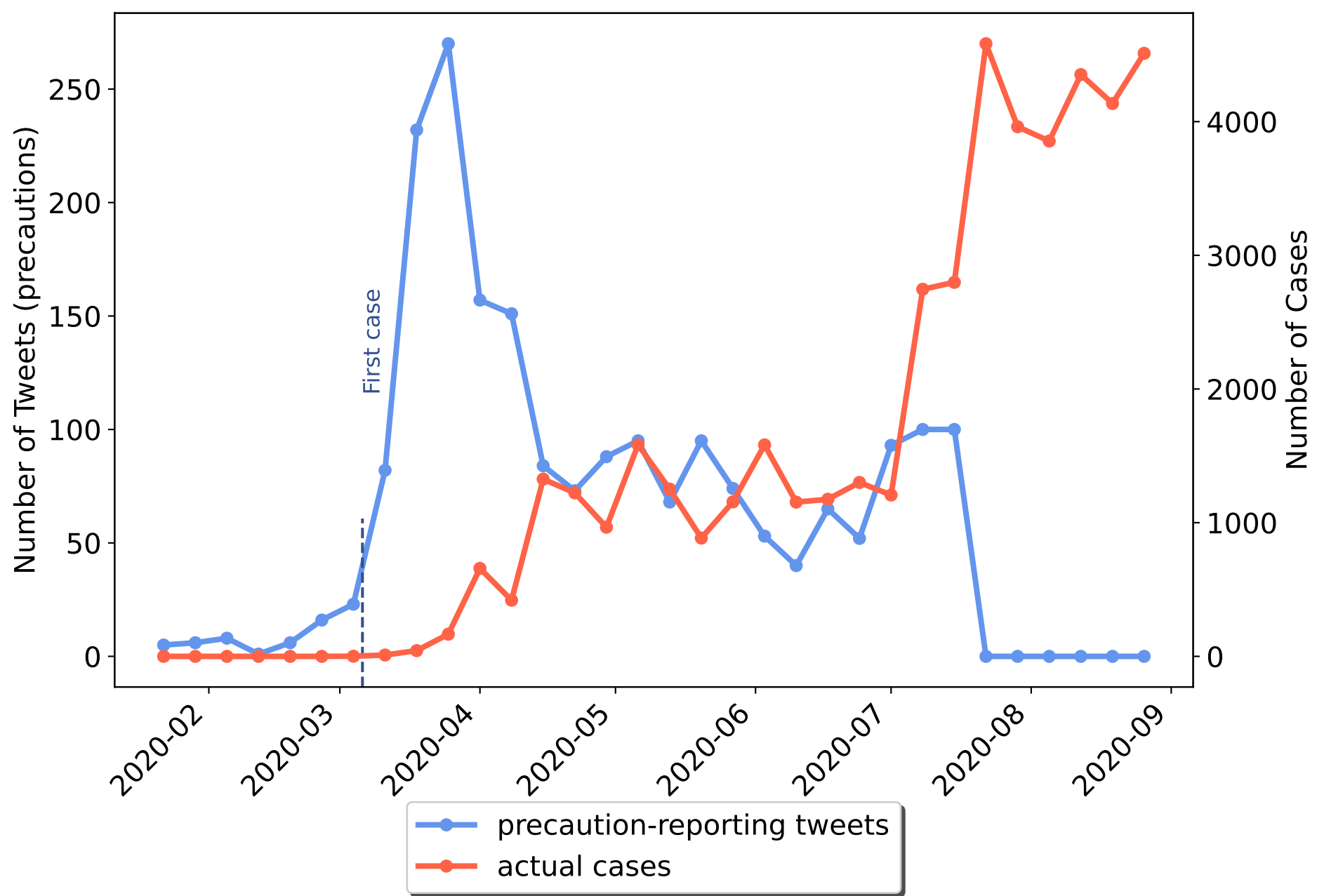

Supplement: Supplementary file 2 [file Data_Sheet_1.ZIP › figures/Kentucky_precaution_twitter-eps-converted-to.pdf]

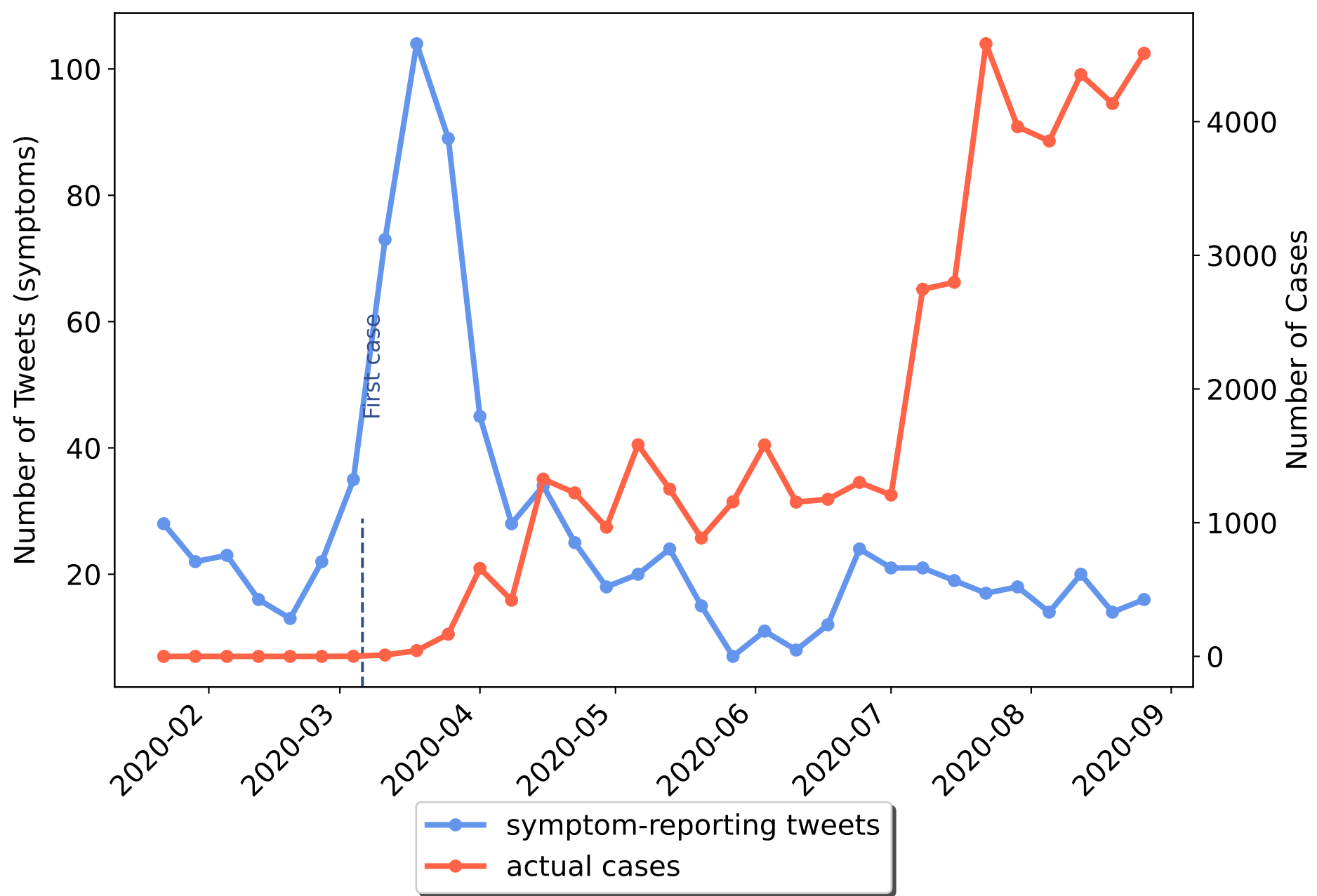

Supplement: Supplementary file 2 [file Data_Sheet_1.ZIP › figures/Kentucky_symptom_twitter-eps-converted-to.pdf]

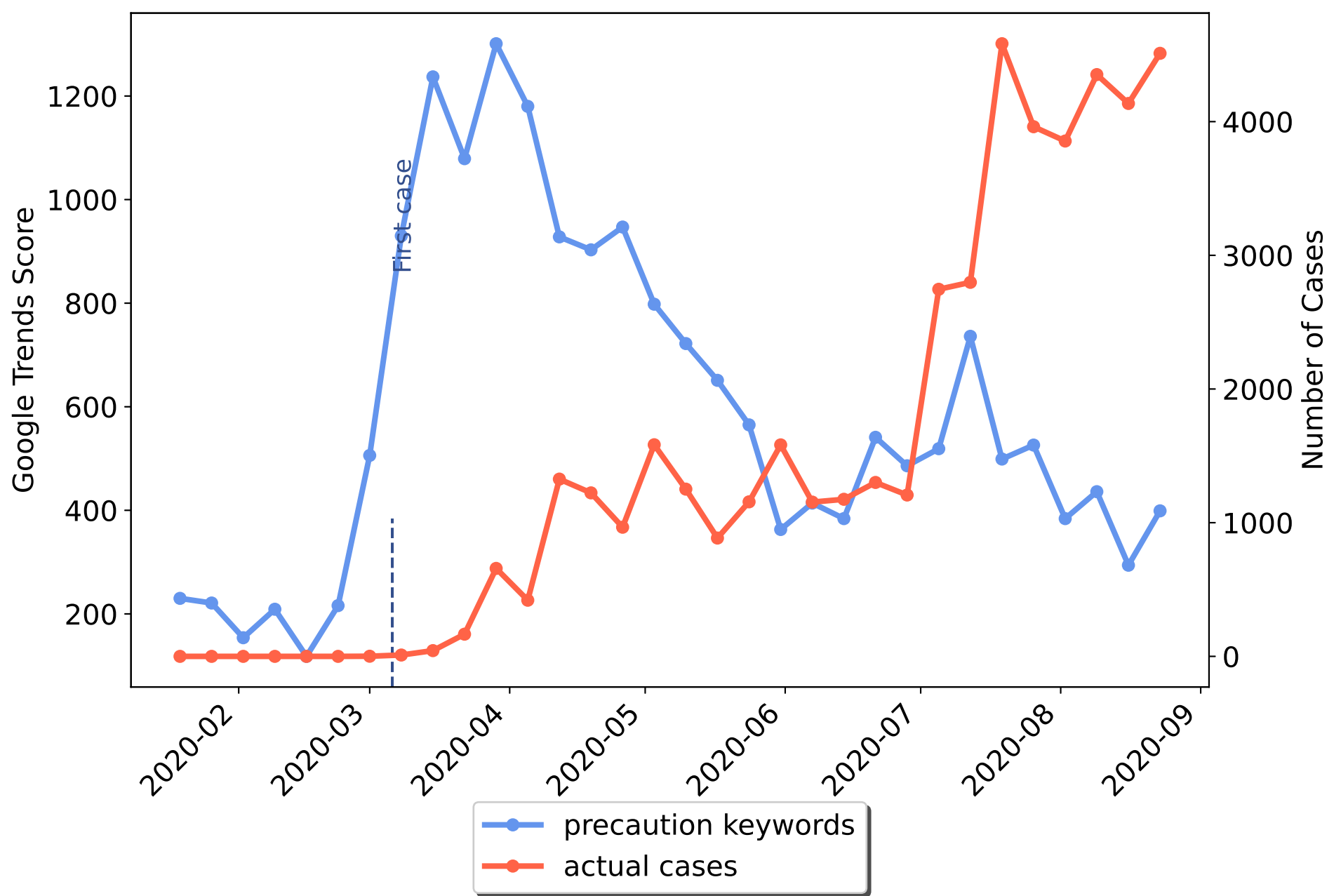

Supplement: Supplementary file 2 [file Data_Sheet_1.ZIP › figures/Kentucky_totalprecaution_GT-eps-converted-to.pdf]

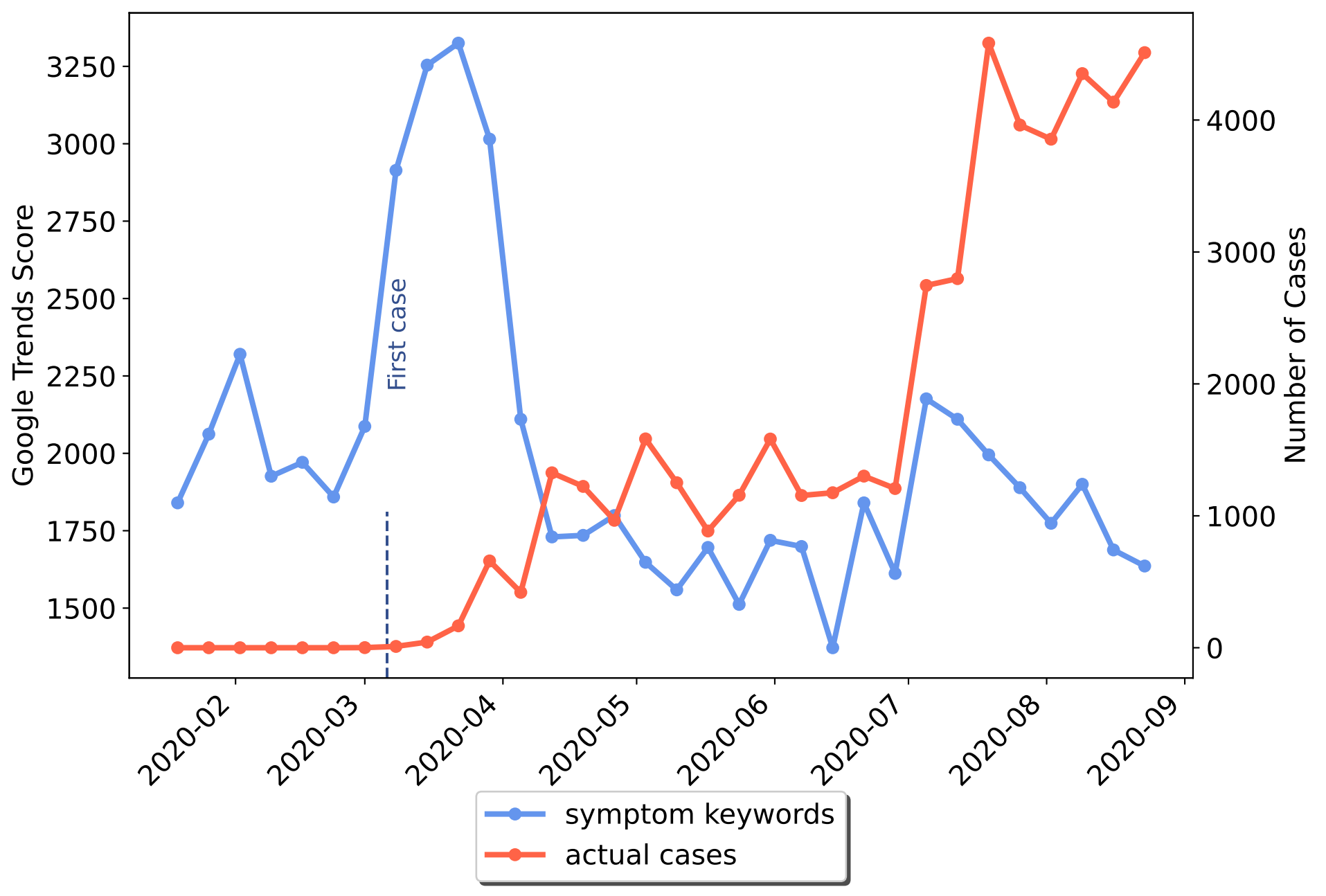

Supplement: Supplementary file 2 [file Data_Sheet_1.ZIP › figures/Kentucky_totalsymptom_GT-eps-converted-to.pdf]

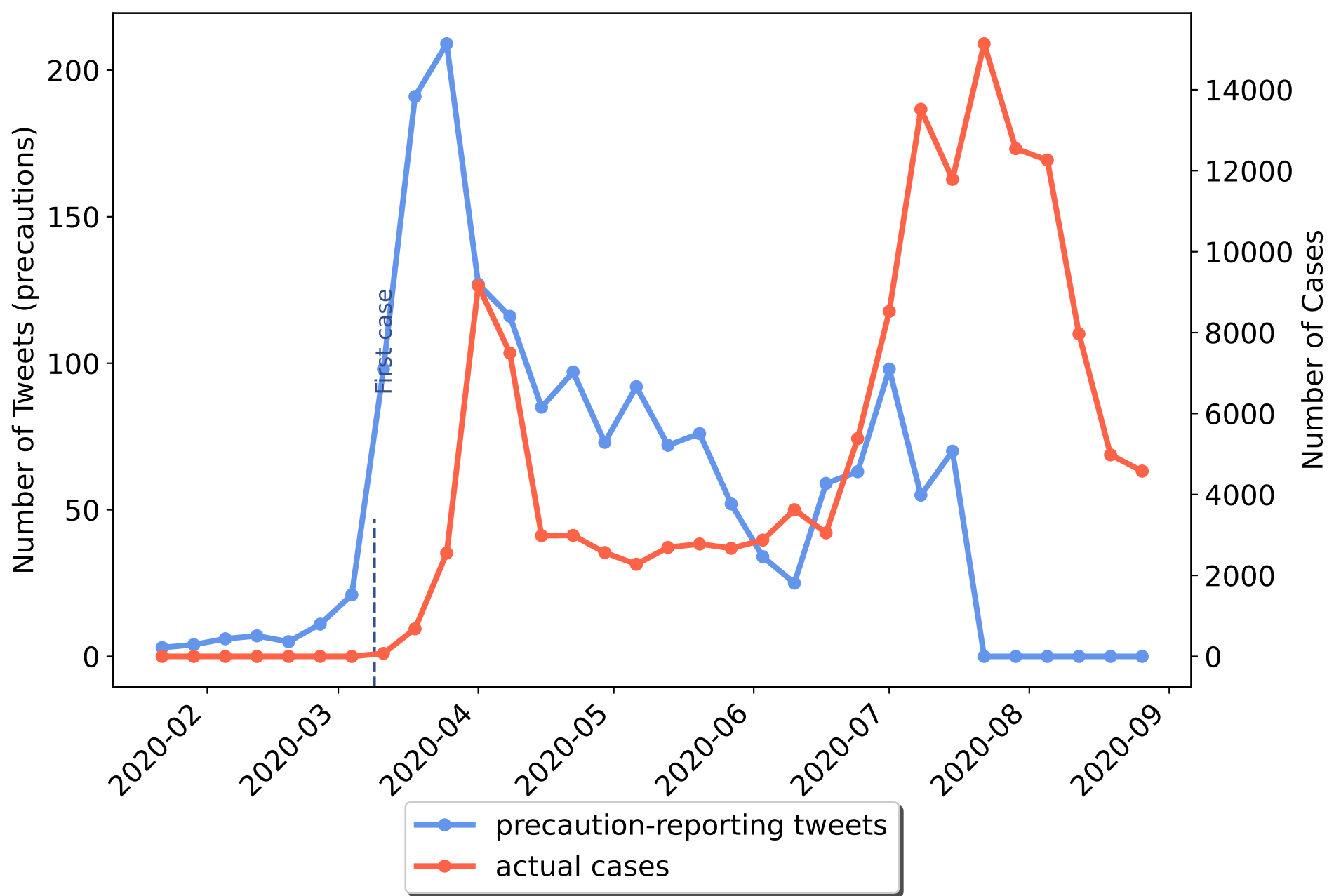

Supplement: Supplementary file 2 [file Data_Sheet_1.ZIP › figures/Louisiana_precaution_twitter-eps-converted-to.pdf]

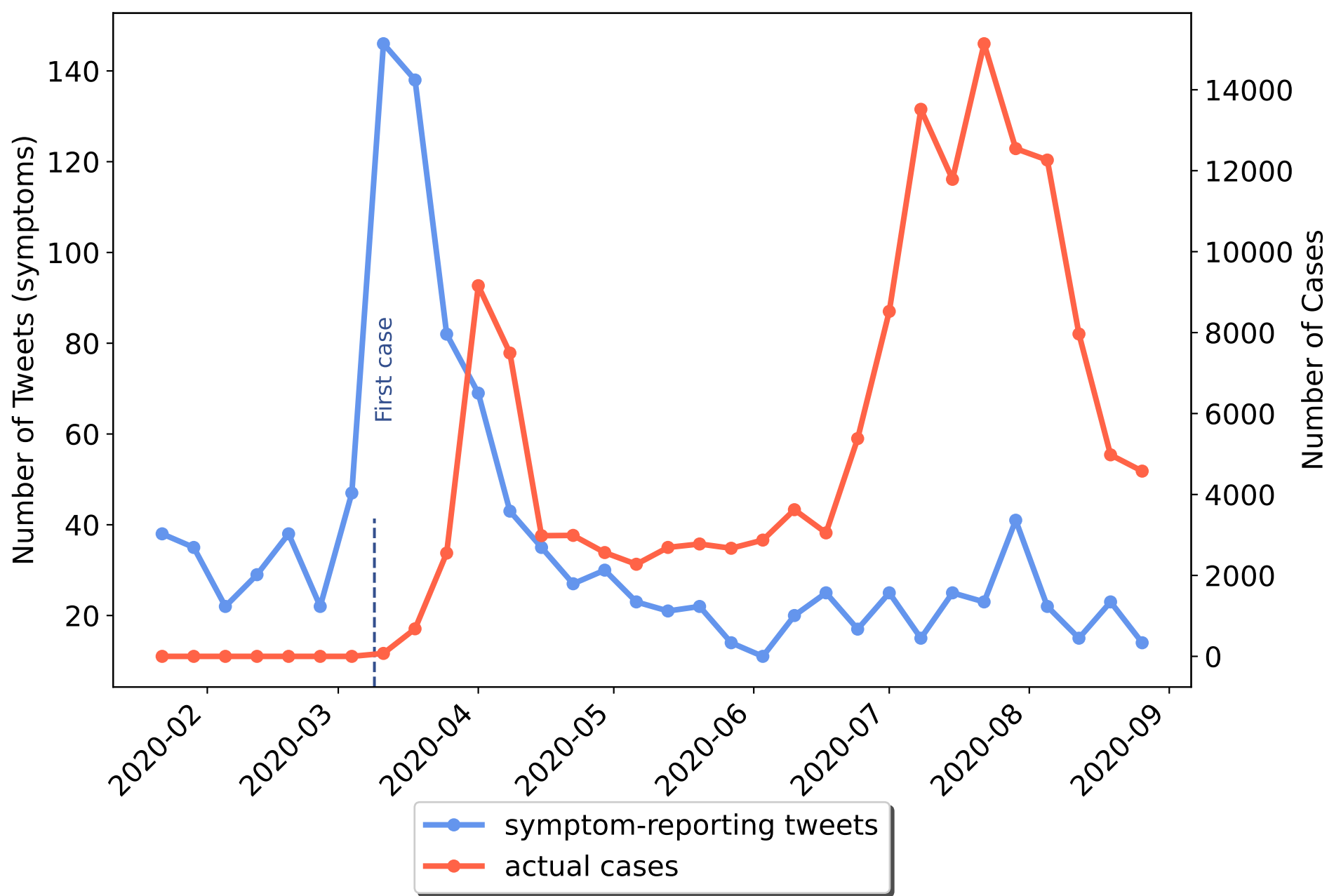

Supplement: Supplementary file 2 [file Data_Sheet_1.ZIP › figures/Louisiana_symptom_twitter-eps-converted-to.pdf]

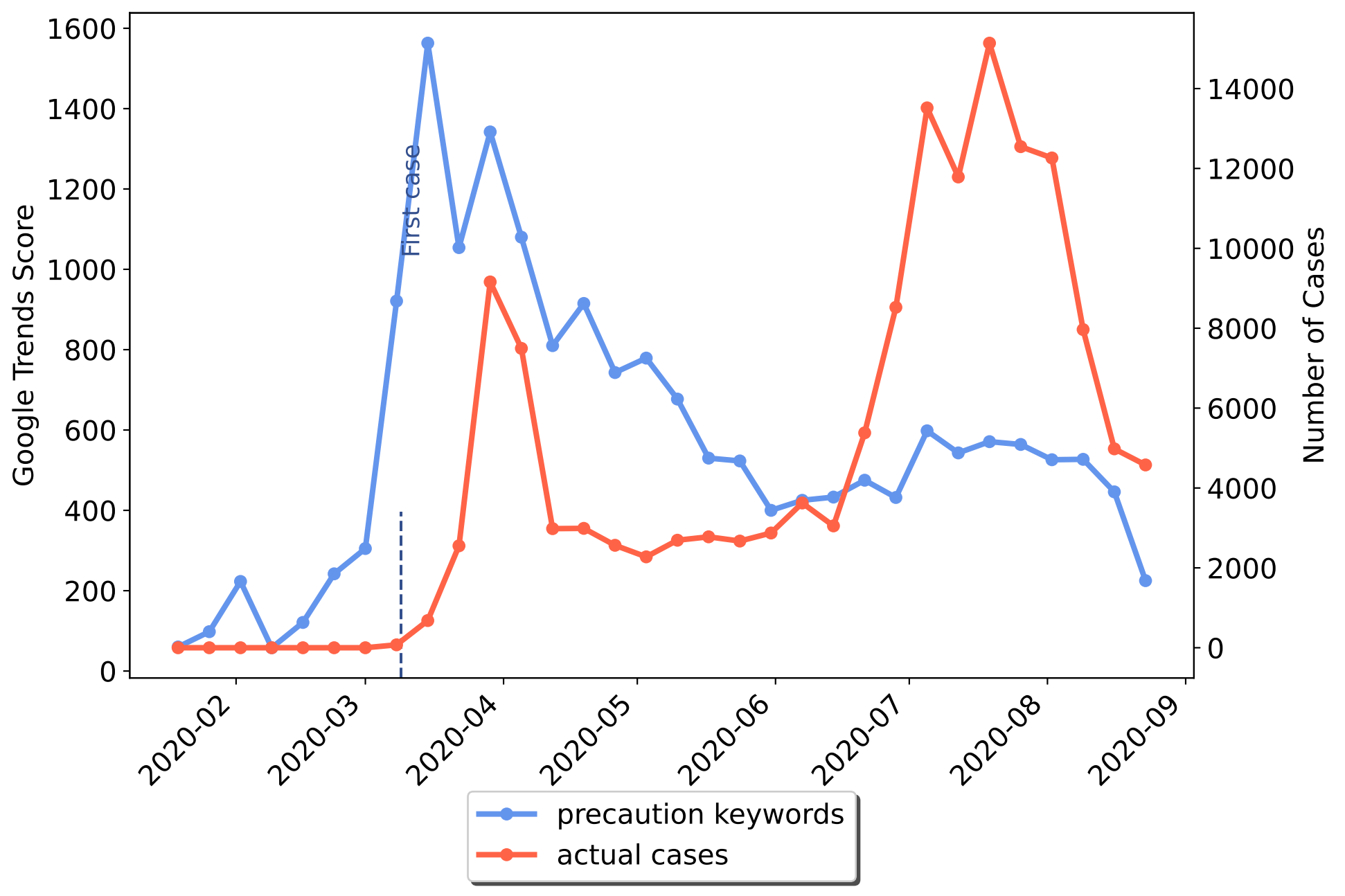

Supplement: Supplementary file 2 [file Data_Sheet_1.ZIP › figures/Louisiana_totalprecaution_GT-eps-converted-to.pdf]

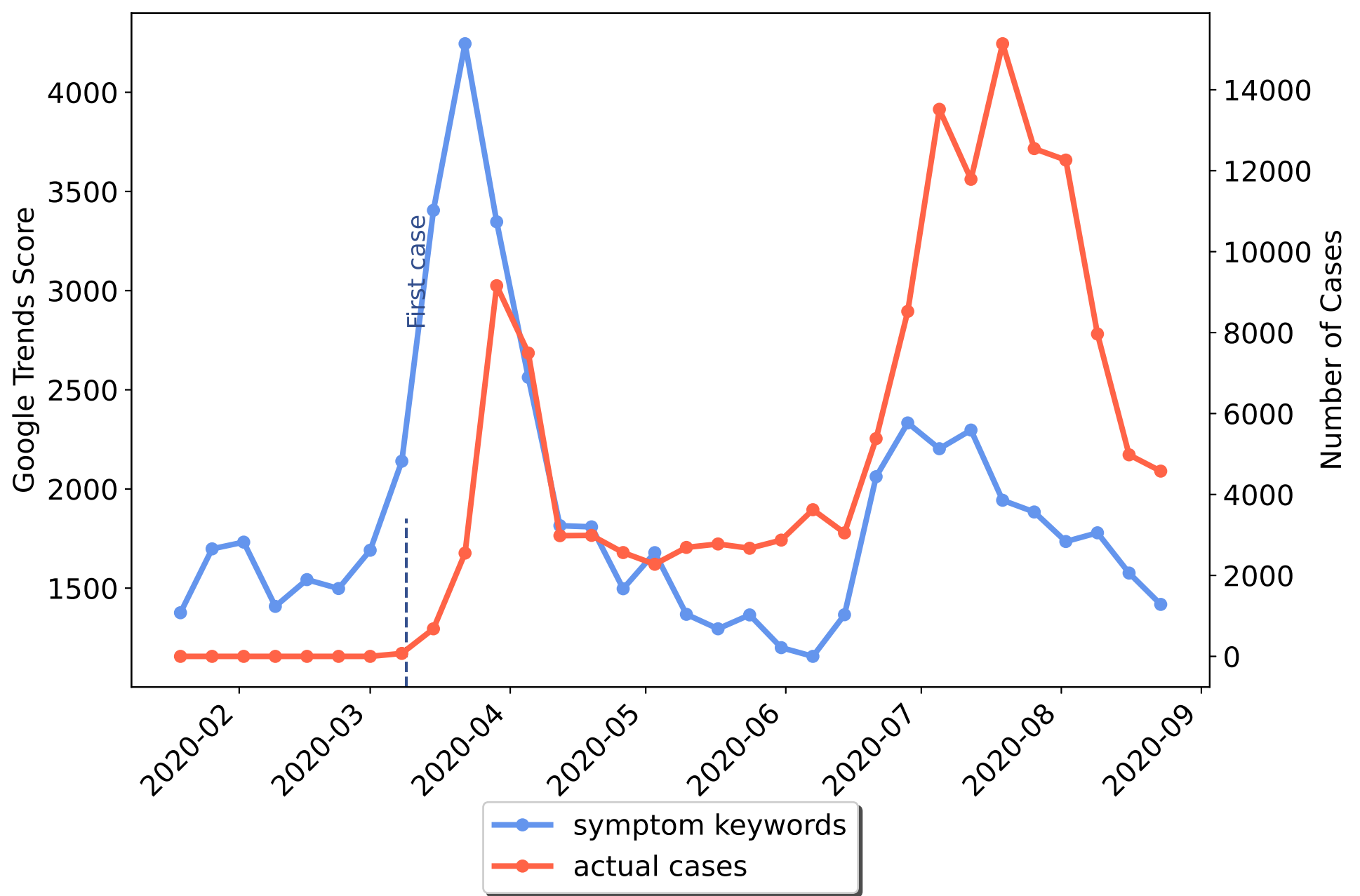

Supplement: Supplementary file 2 [file Data_Sheet_1.ZIP › figures/Louisiana_totalsymptom_GT-eps-converted-to.pdf]

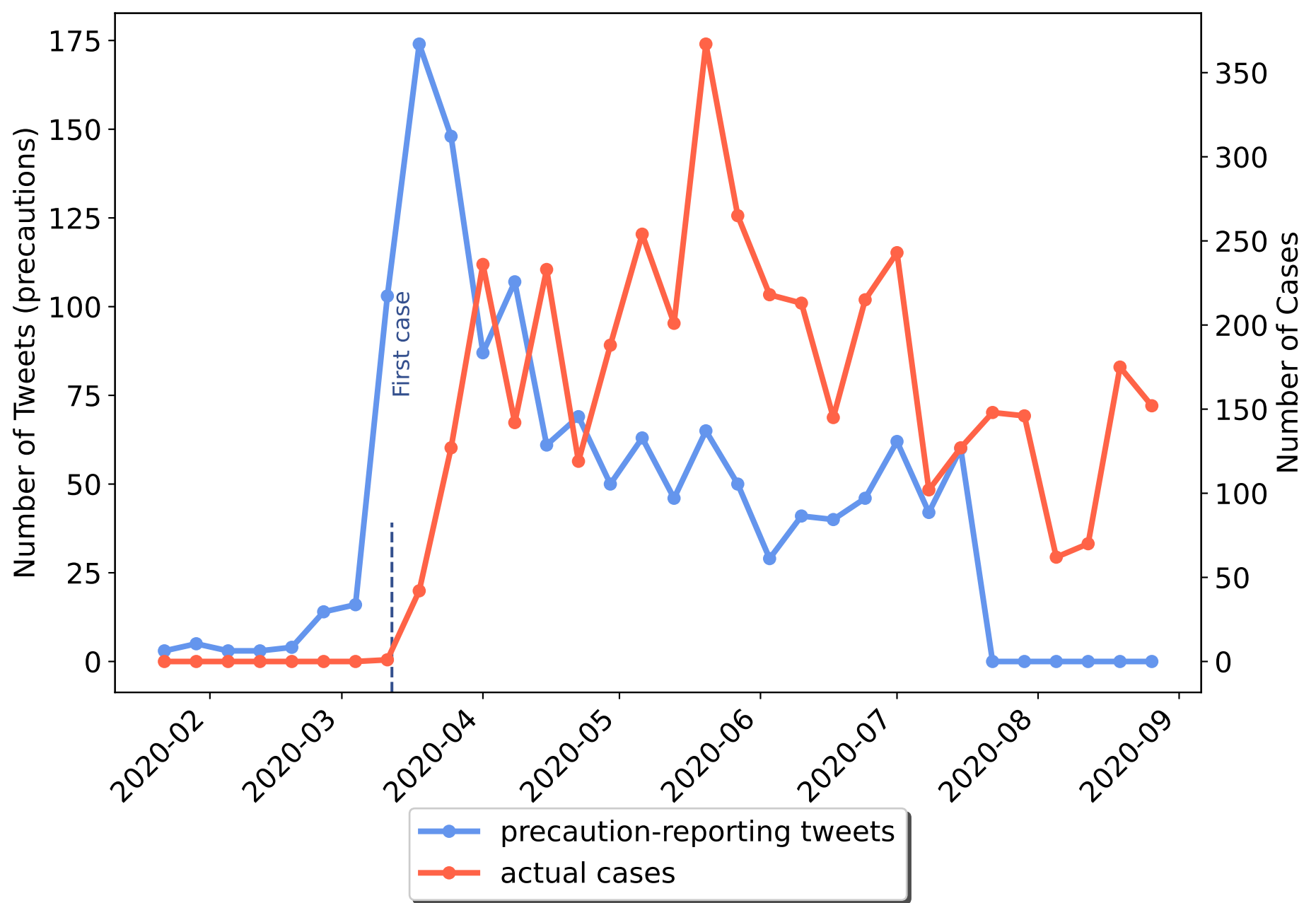

Supplement: Supplementary file 2 [file Data_Sheet_1.ZIP › figures/Maine_precaution_twitter-eps-converted-to.pdf]

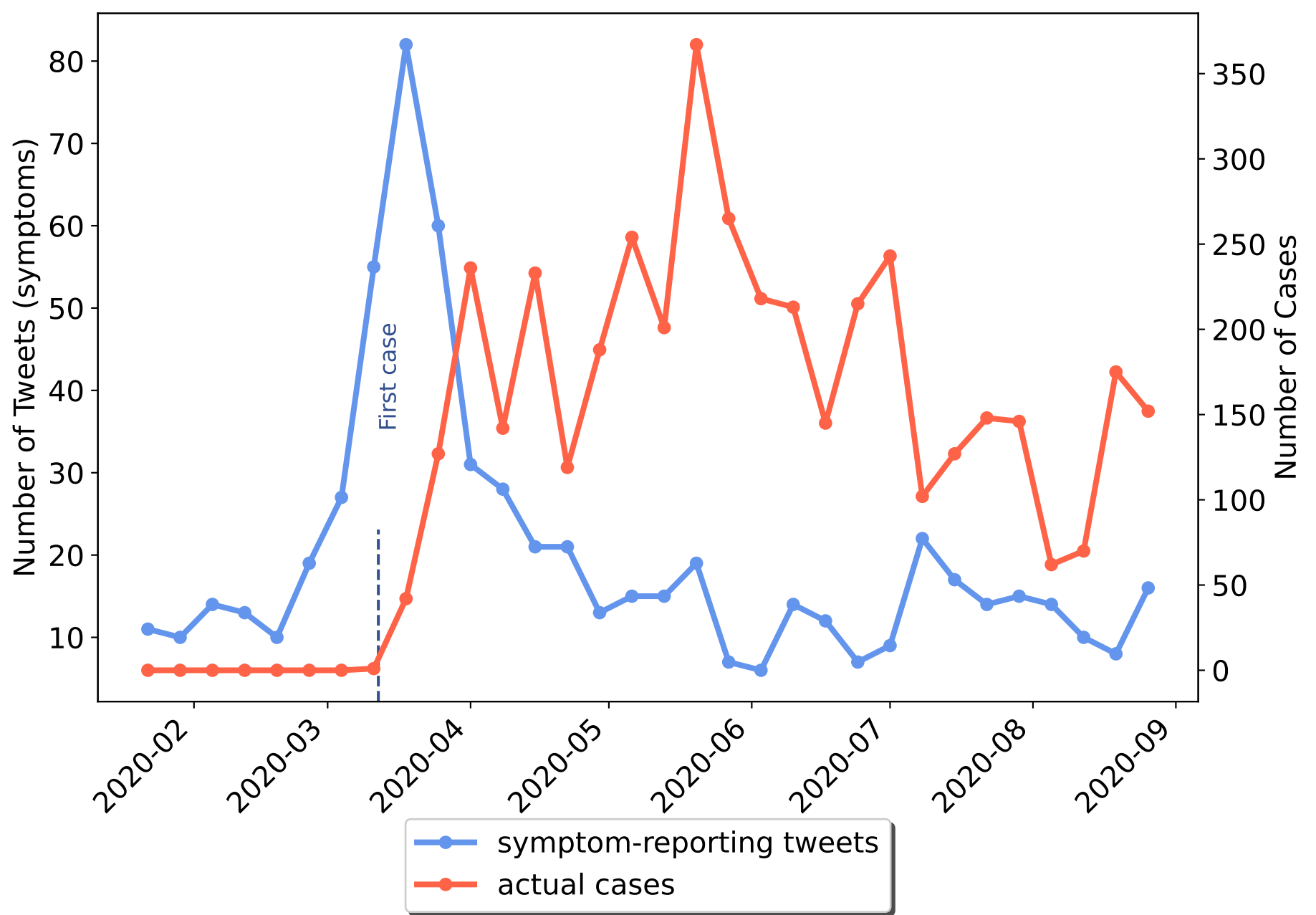

Supplement: Supplementary file 2 [file Data_Sheet_1.ZIP › figures/Maine_symptom_twitter-eps-converted-to.pdf]

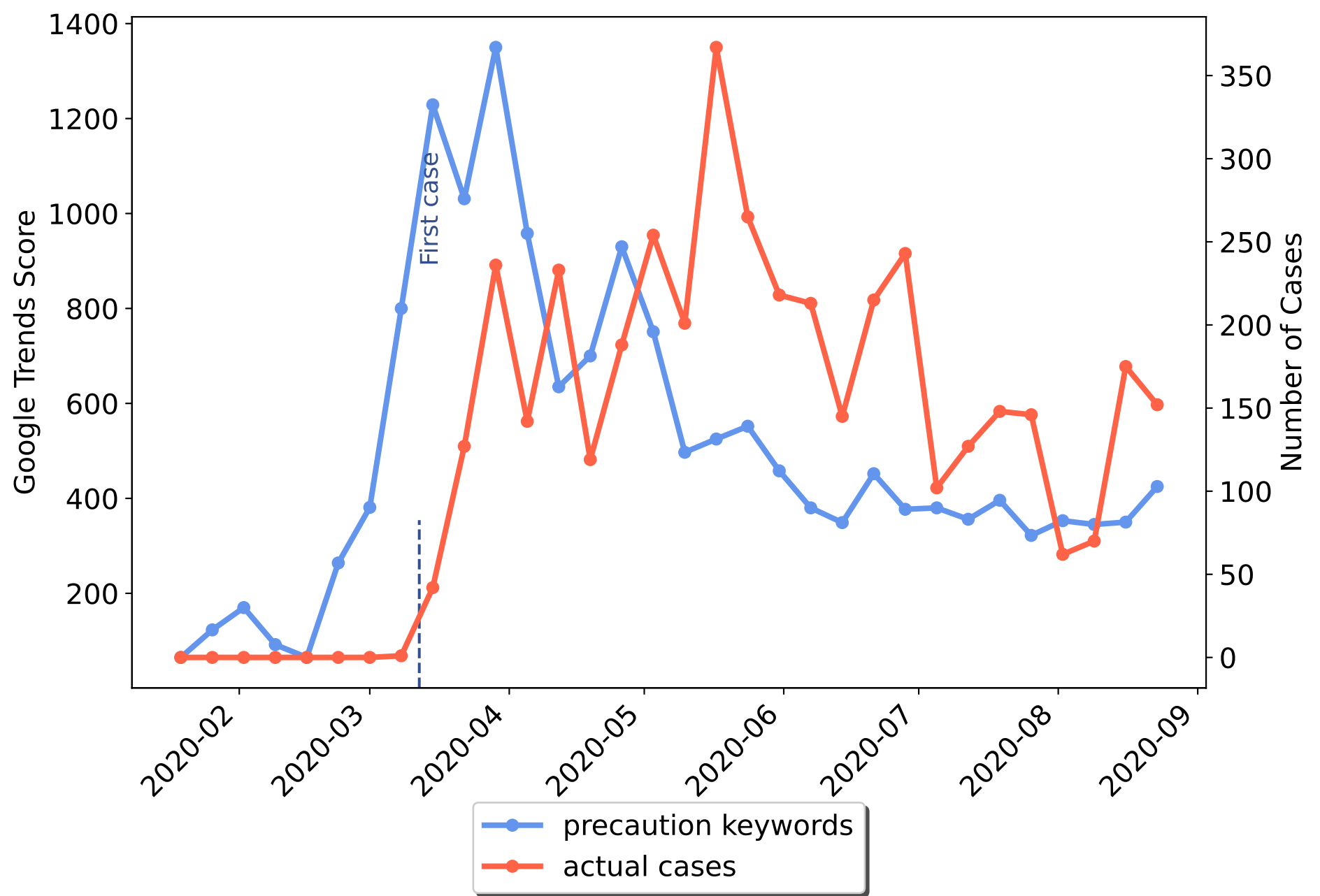

Supplement: Supplementary file 2 [file Data_Sheet_1.ZIP › figures/Maine_totalprecaution_GT-eps-converted-to.pdf]

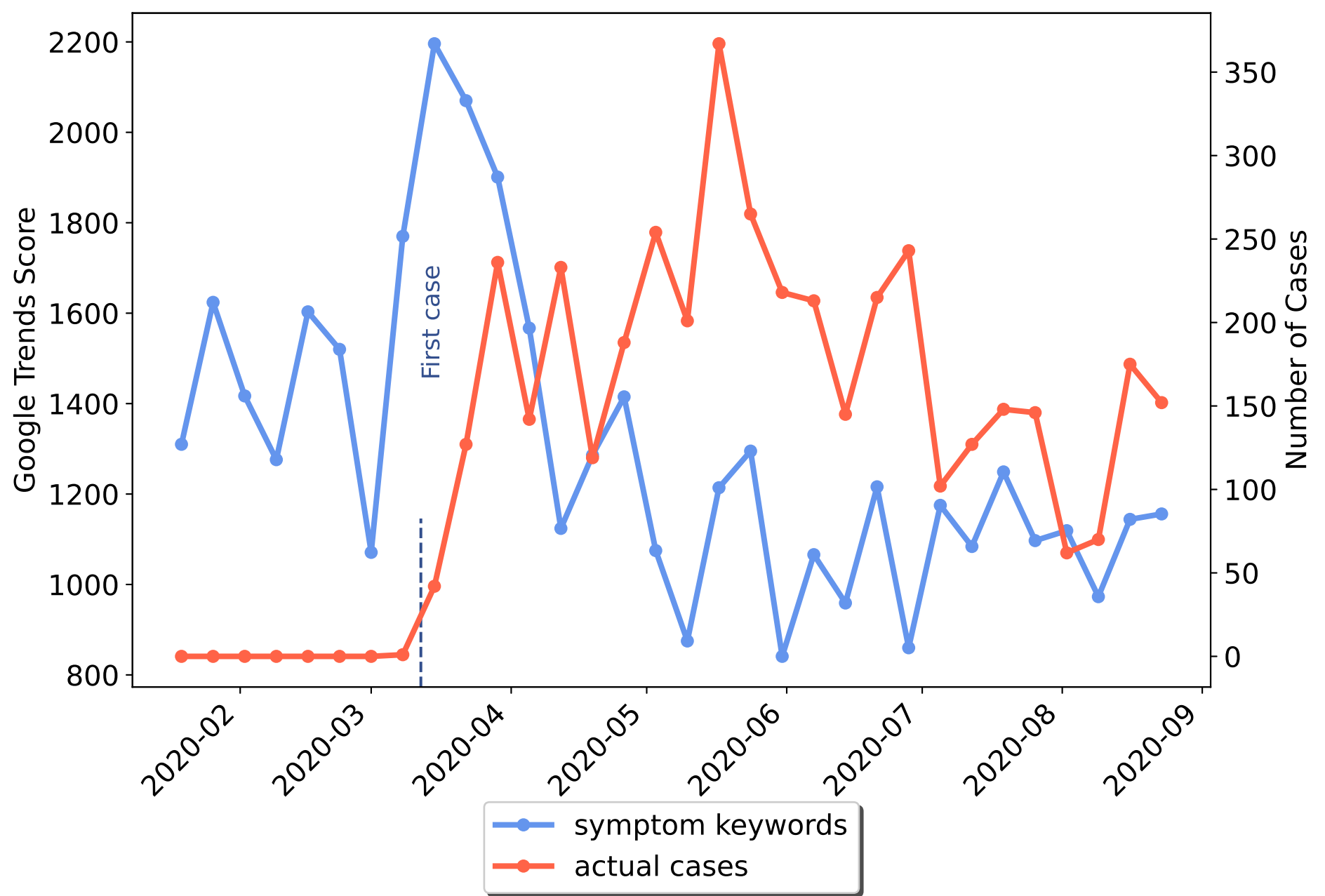

Supplement: Supplementary file 2 [file Data_Sheet_1.ZIP › figures/Maine_totalsymptom_GT-eps-converted-to.pdf]

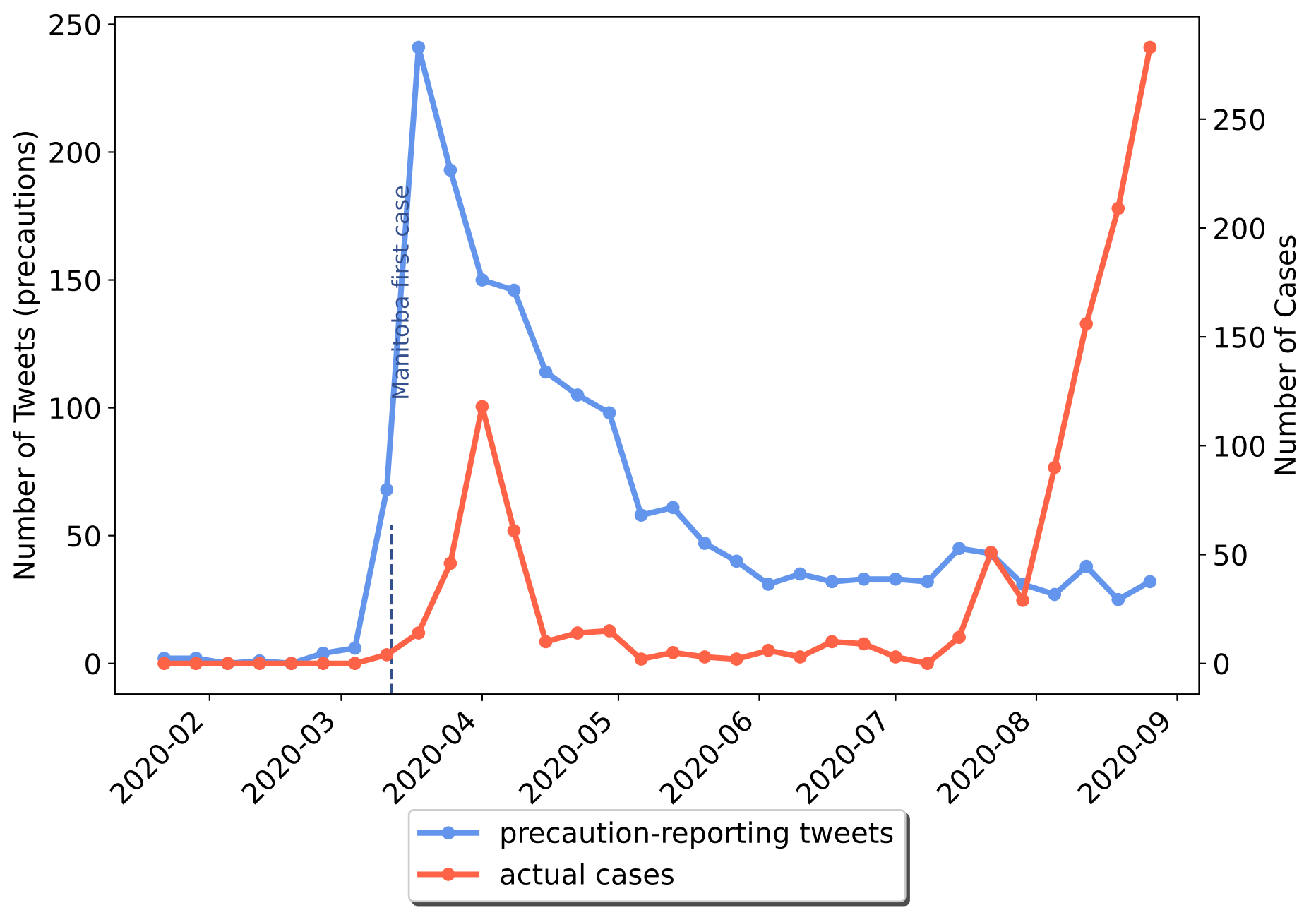

Supplement: Supplementary file 2 [file Data_Sheet_1.ZIP › figures/Manitoba_precaution_twitter-eps-converted-to.pdf]

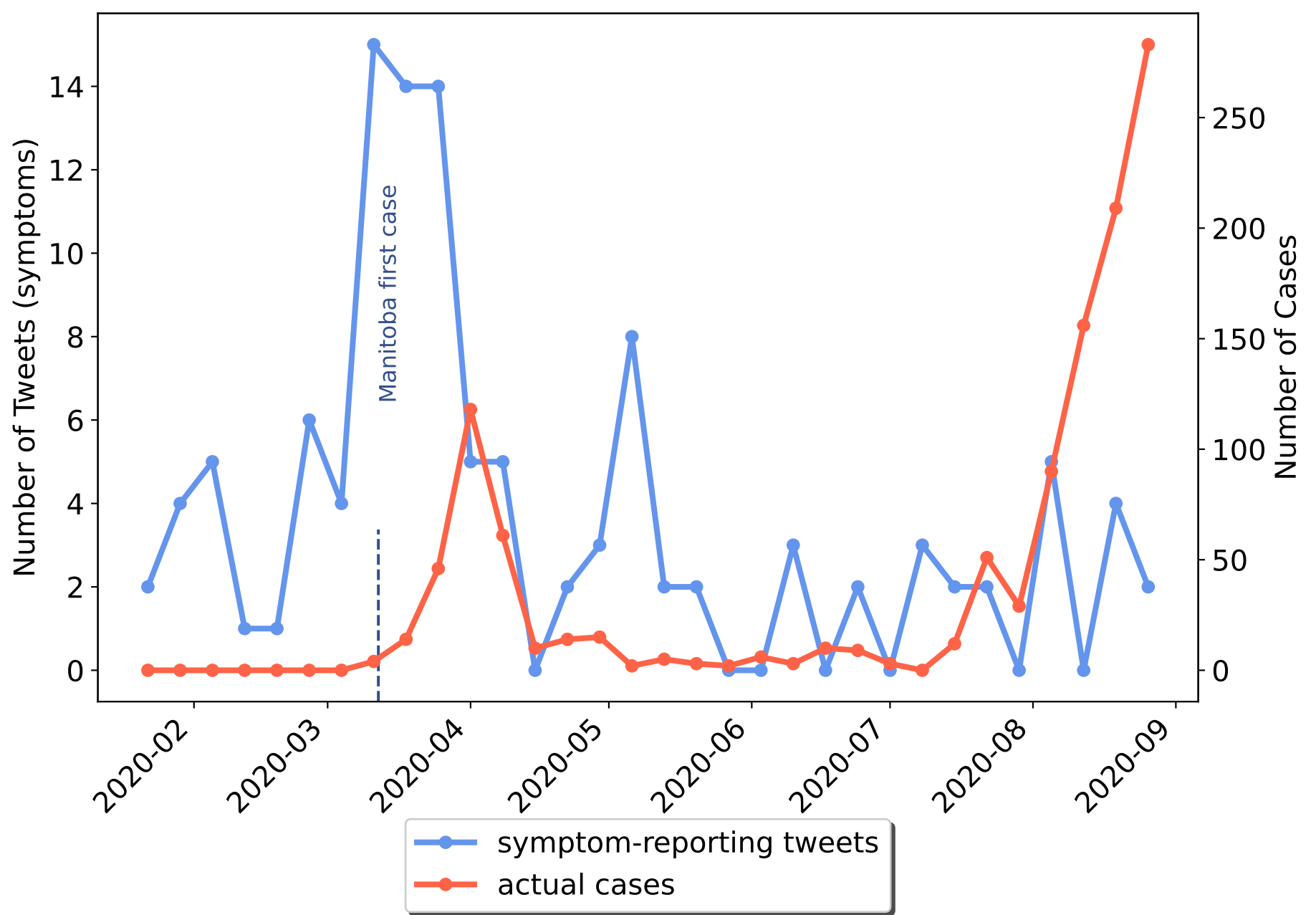

Supplement: Supplementary file 2 [file Data_Sheet_1.ZIP › figures/Manitoba_symptom_twitter-eps-converted-to.pdf]

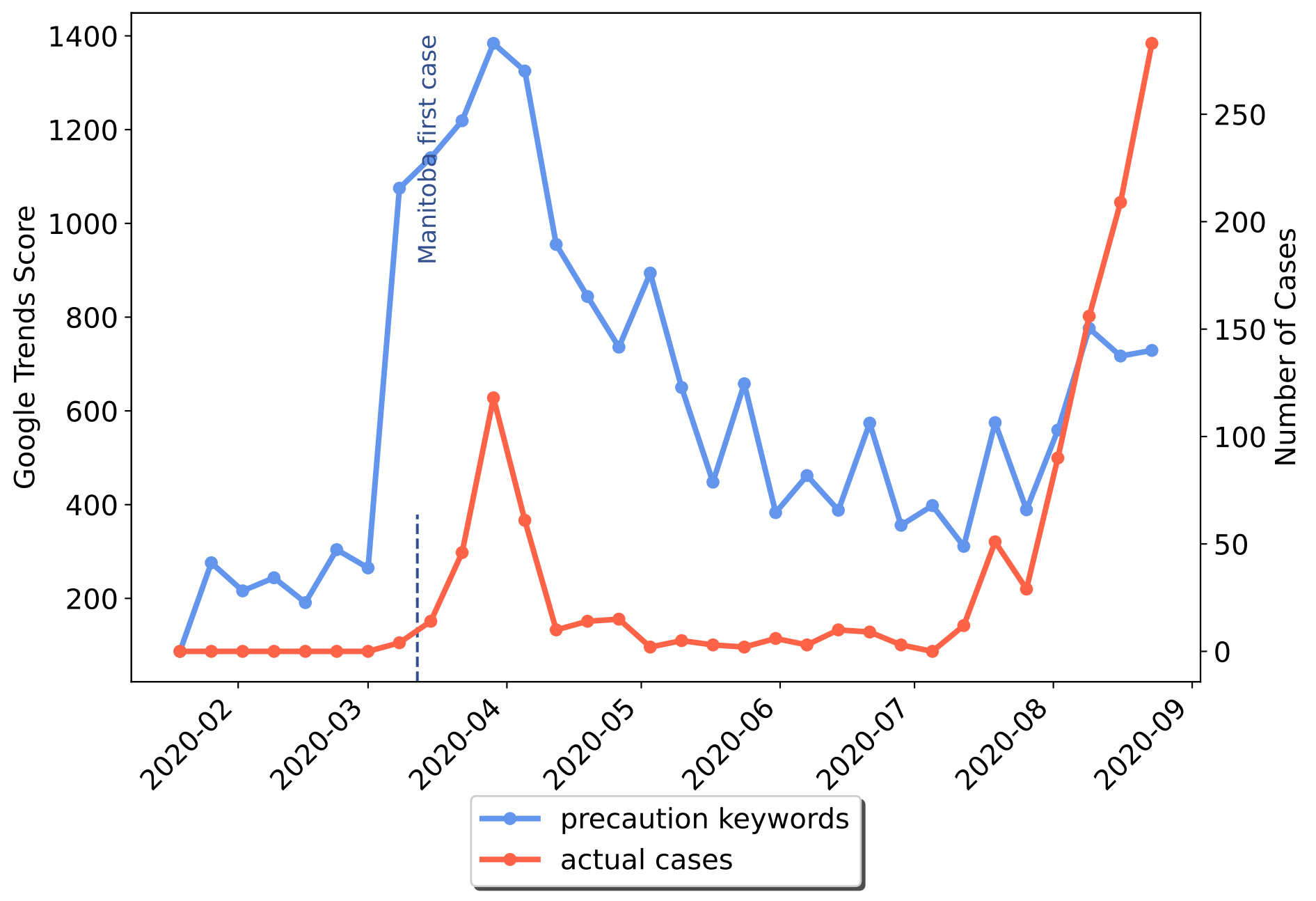

Supplement: Supplementary file 2 [file Data_Sheet_1.ZIP › figures/Manitoba_totalprecaution_GT-eps-converted-to.pdf]

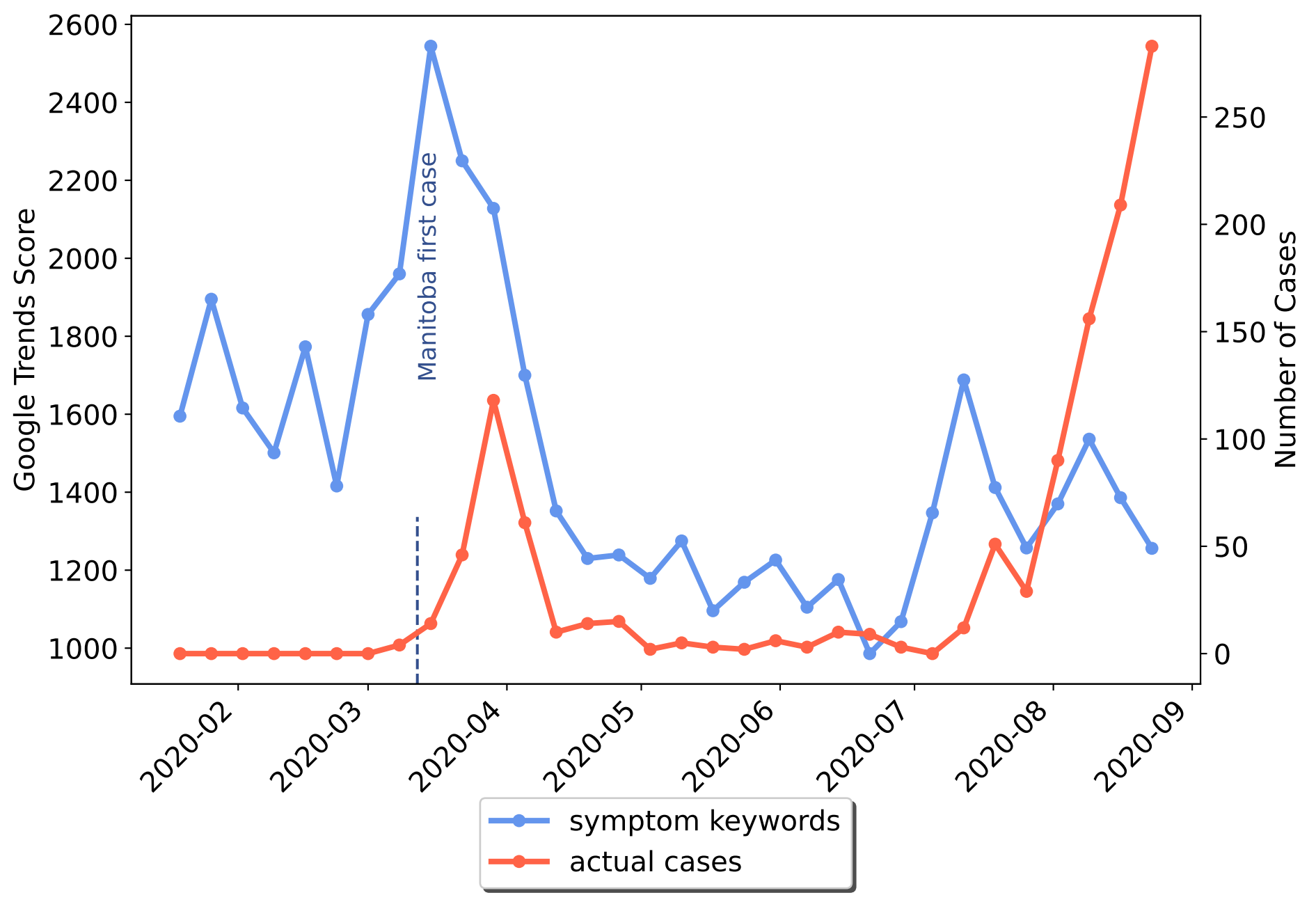

Supplement: Supplementary file 2 [file Data_Sheet_1.ZIP › figures/Manitoba_totalsymptom_GT-eps-converted-to.pdf]

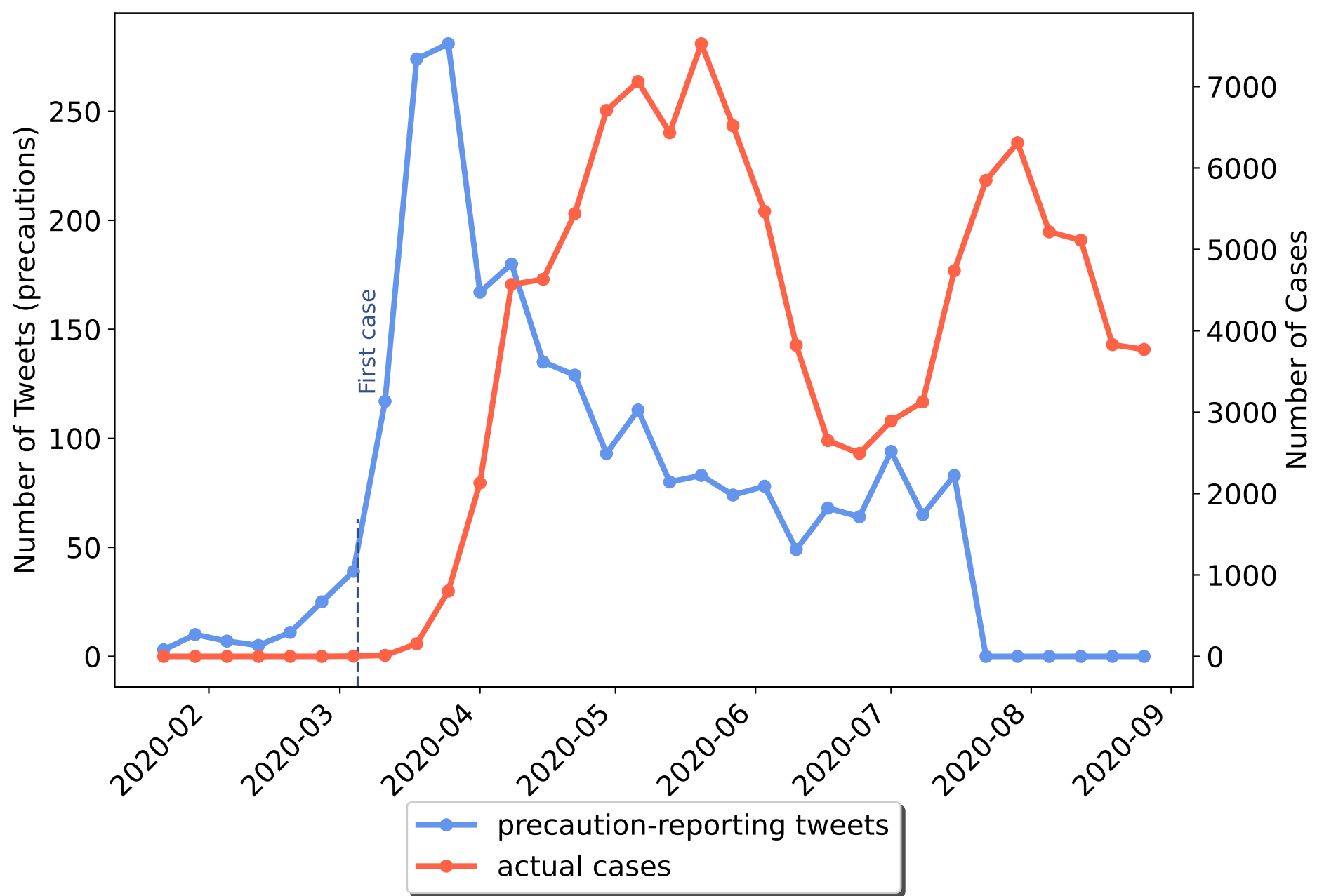

Supplement: Supplementary file 2 [file Data_Sheet_1.ZIP › figures/Maryland_precaution_twitter-eps-converted-to.pdf]

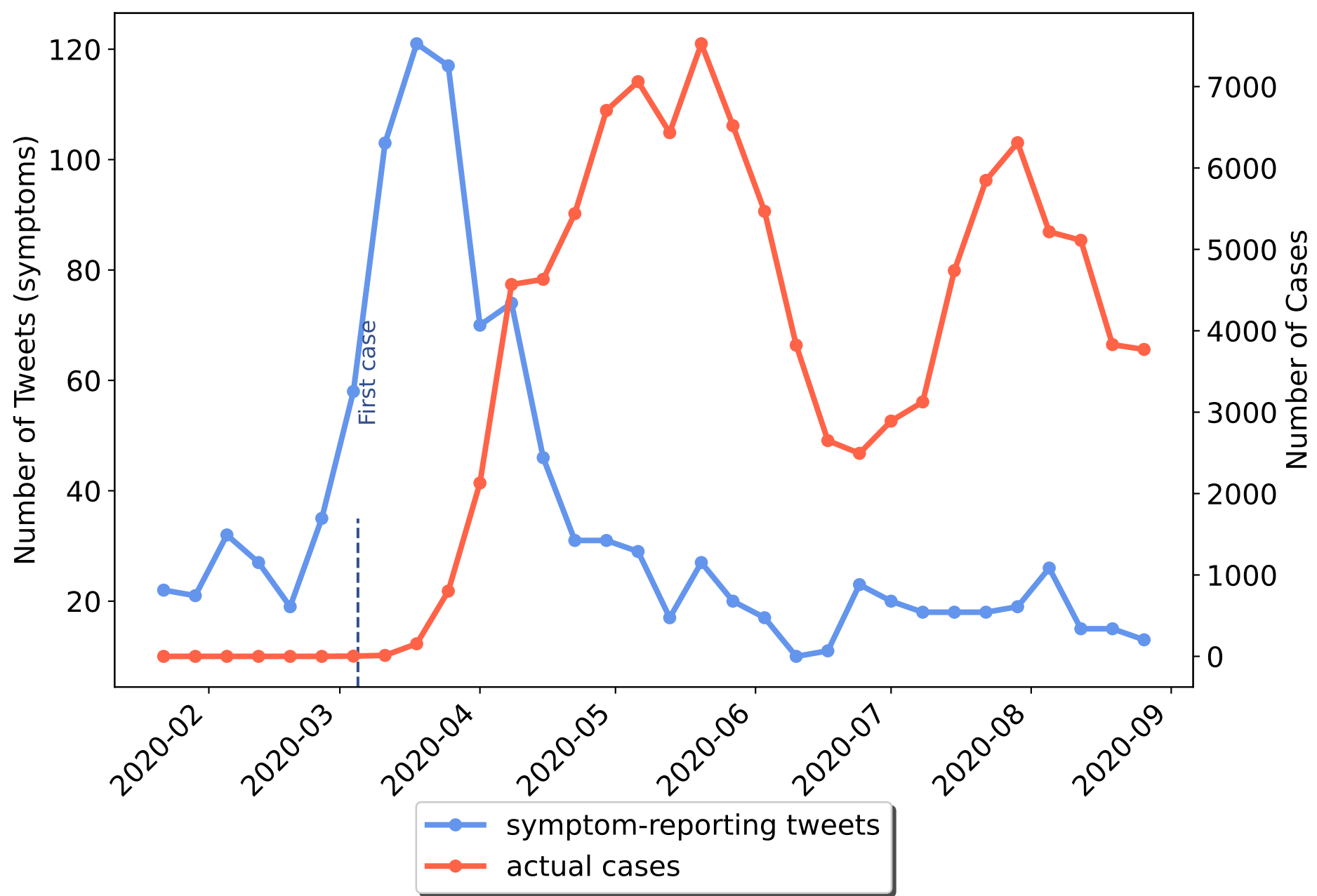

Supplement: Supplementary file 2 [file Data_Sheet_1.ZIP › figures/Maryland_symptom_twitter-eps-converted-to.pdf]

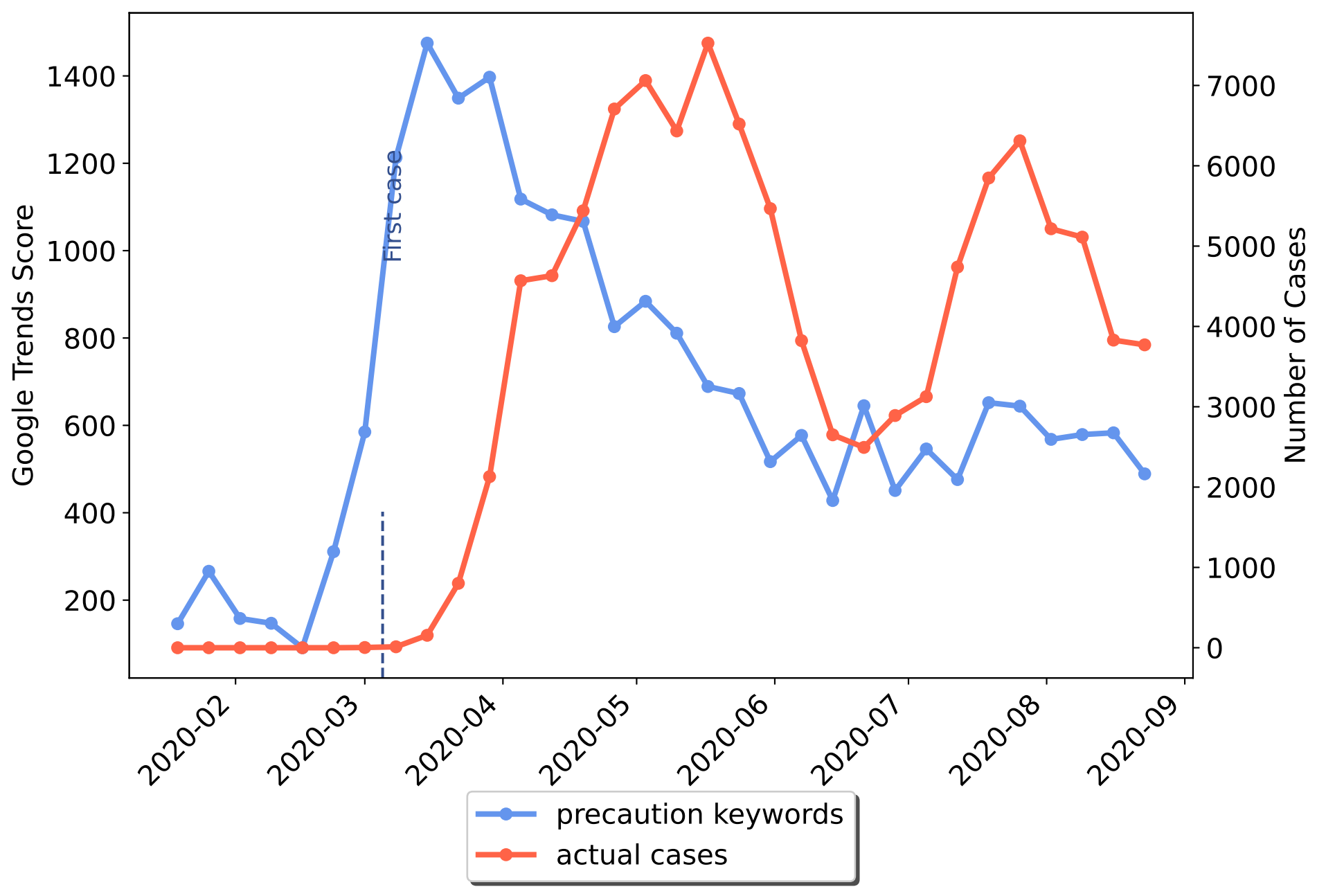

Supplement: Supplementary file 2 [file Data_Sheet_1.ZIP › figures/Maryland_totalprecaution_GT-eps-converted-to.pdf]

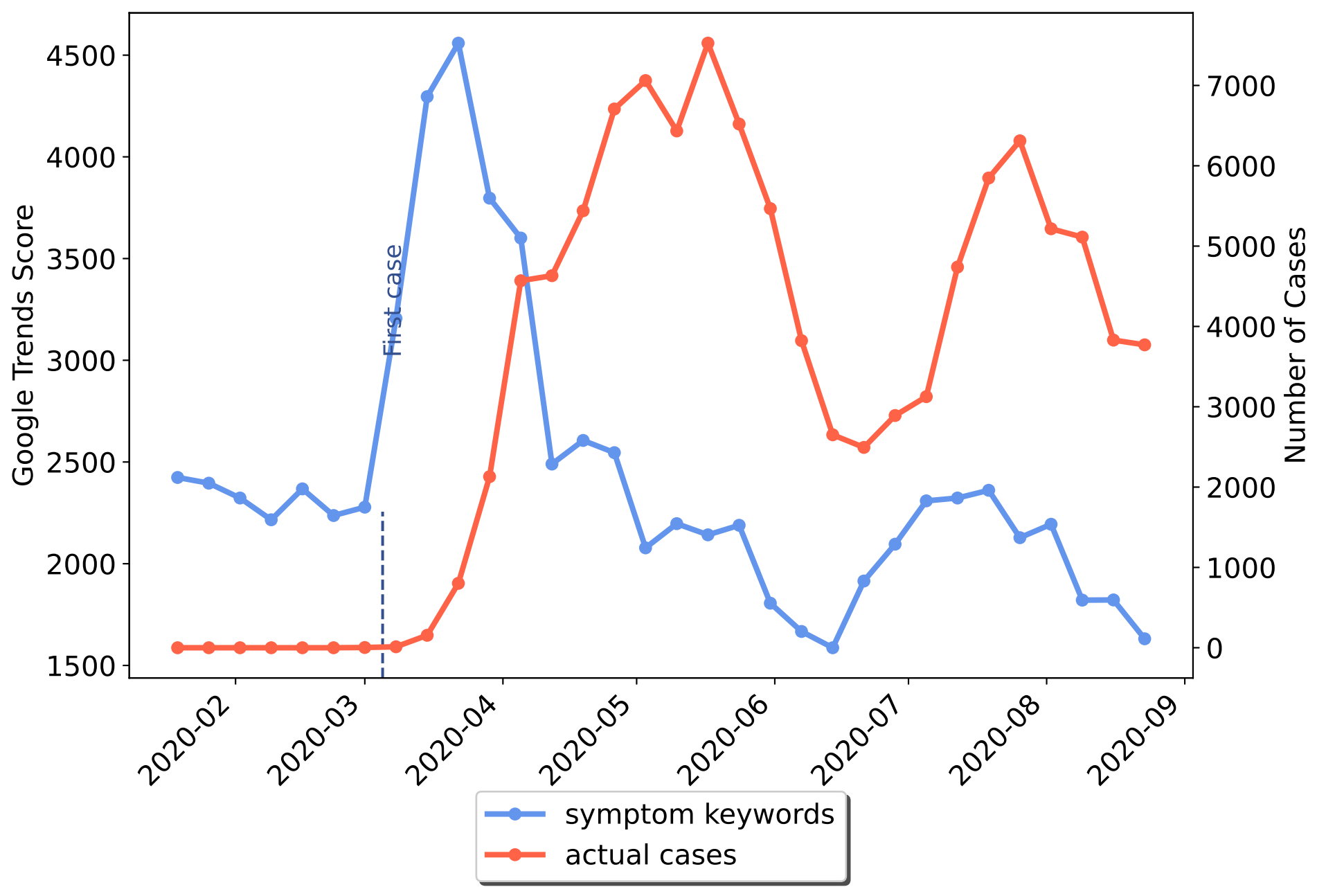

Supplement: Supplementary file 2 [file Data_Sheet_1.ZIP › figures/Maryland_totalsymptom_GT-eps-converted-to.pdf]

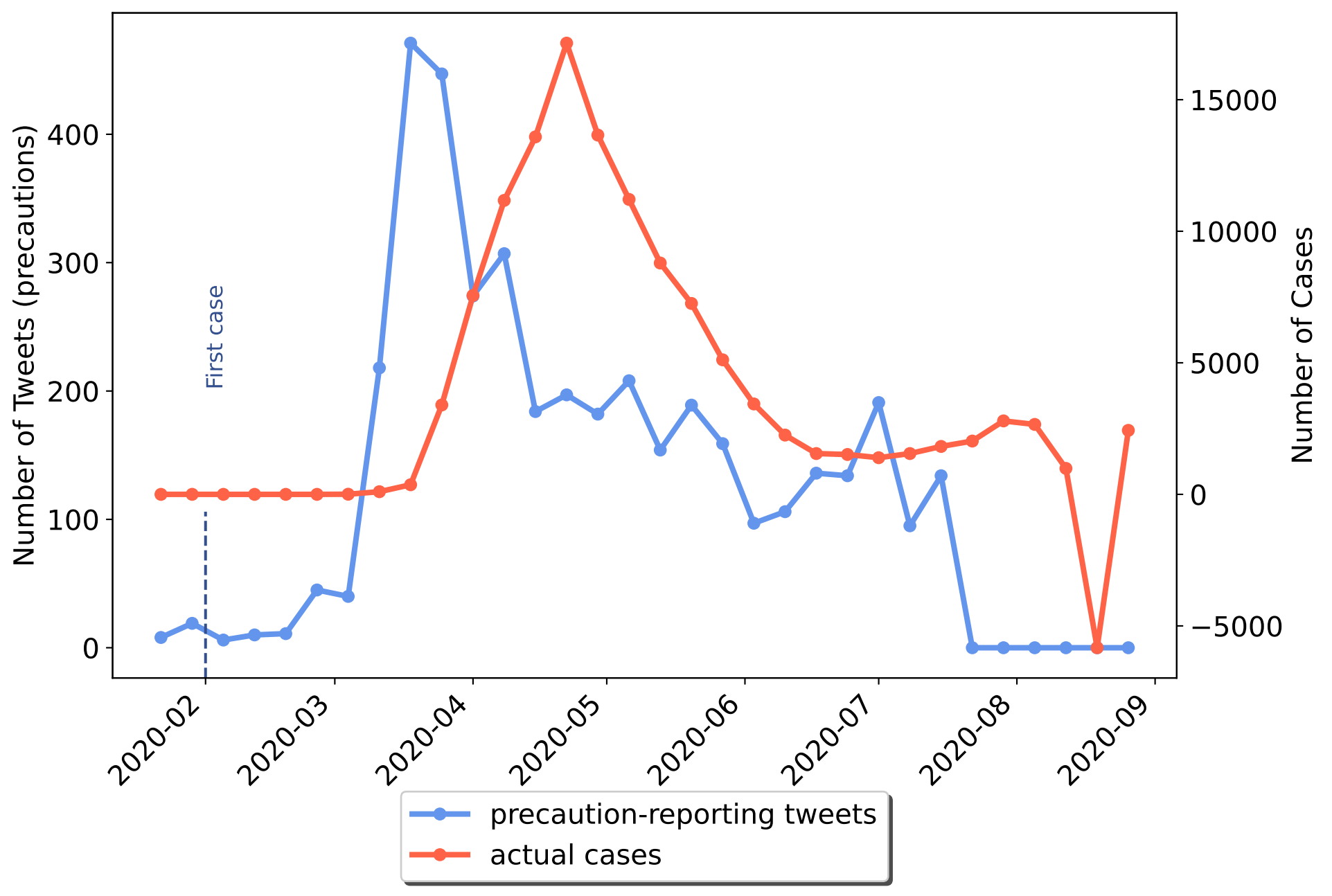

Supplement: Supplementary file 2 [file Data_Sheet_1.ZIP › figures/Massachusetts_precaution_twitter-eps-converted-to.pdf]

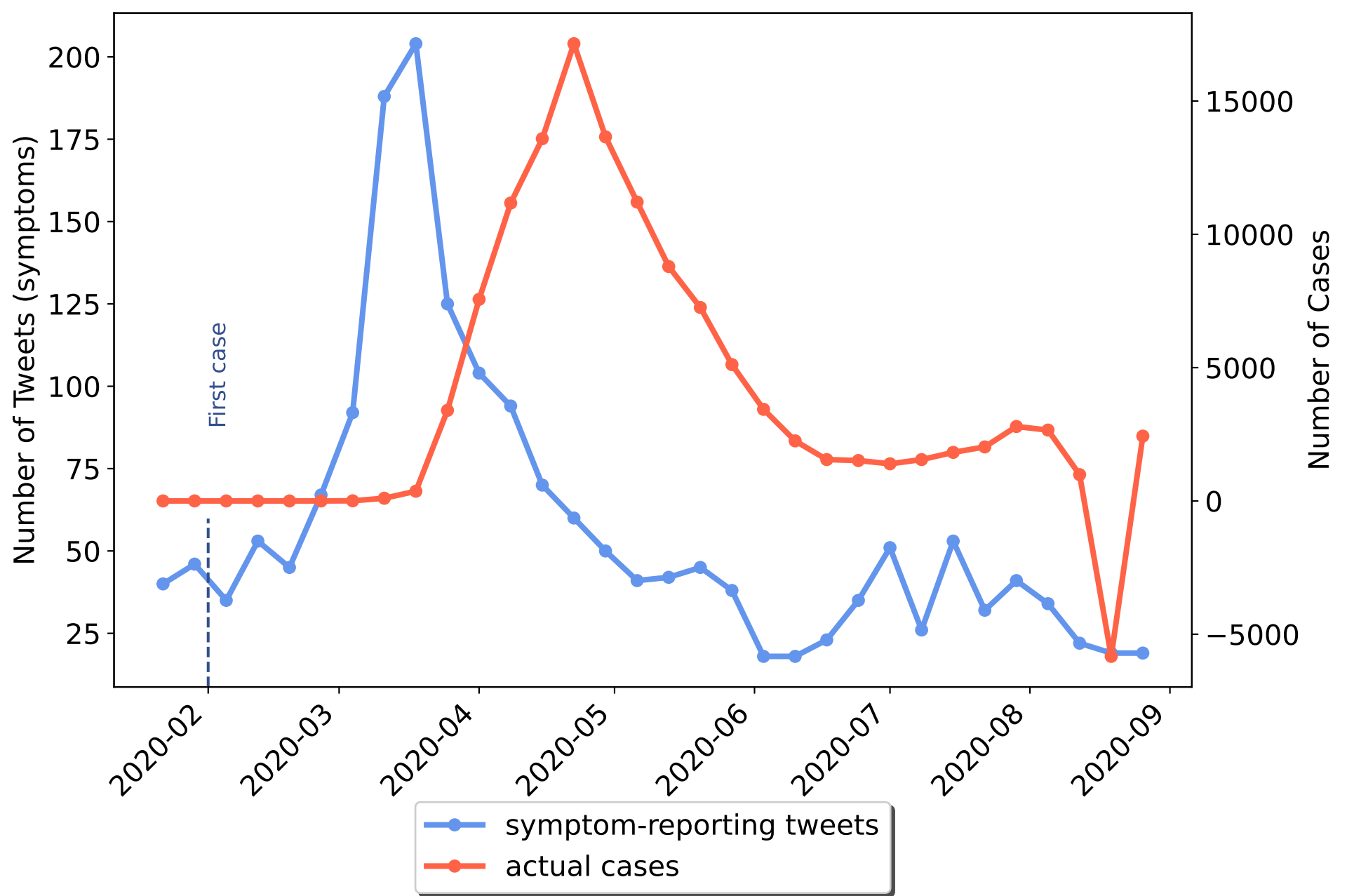

Supplement: Supplementary file 2 [file Data_Sheet_1.ZIP › figures/Massachusetts_symptom_twitter-eps-converted-to.pdf]

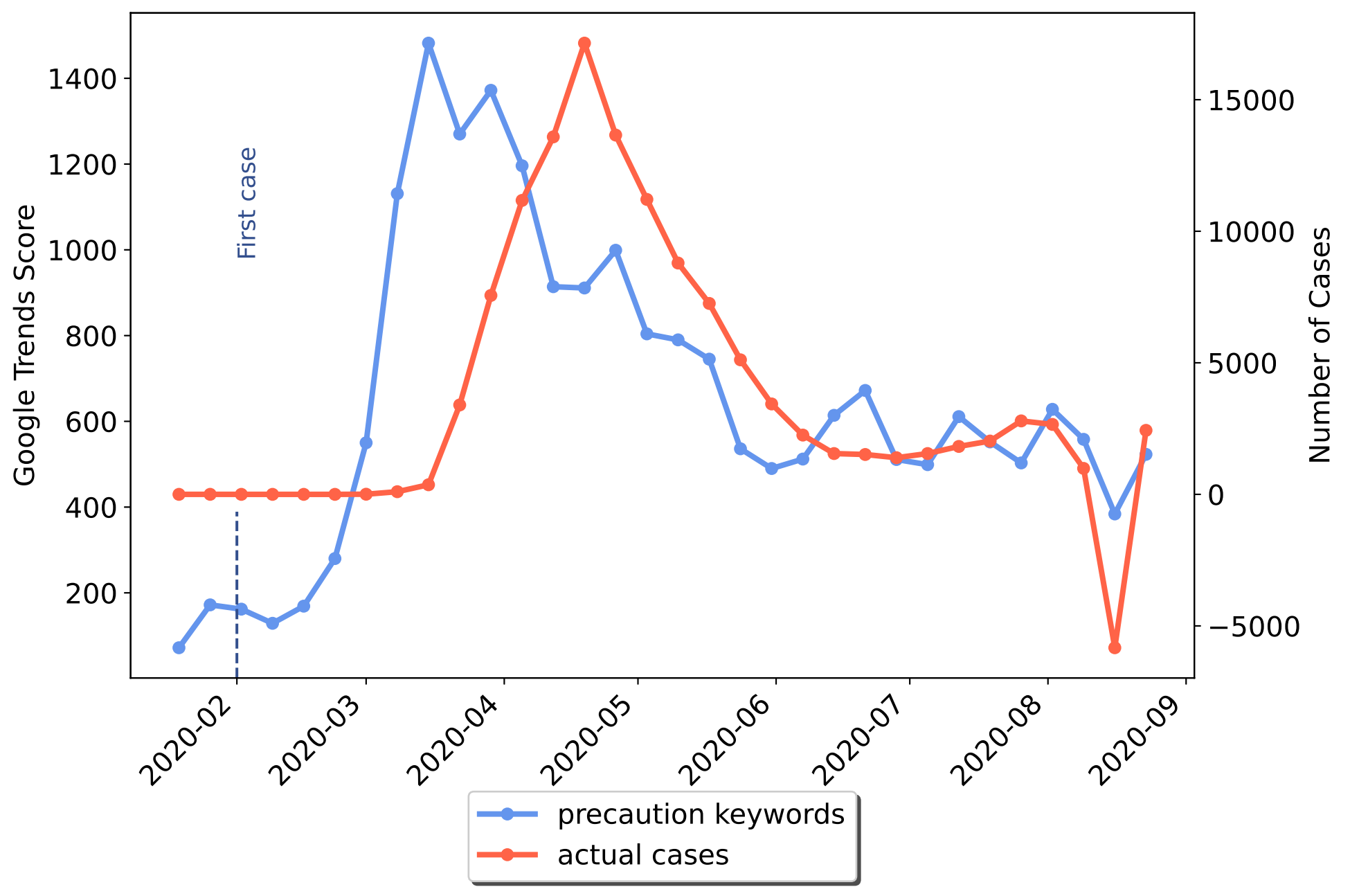

Supplement: Supplementary file 2 [file Data_Sheet_1.ZIP › figures/Massachusetts_totalprecaution_GT-eps-converted-to.pdf]

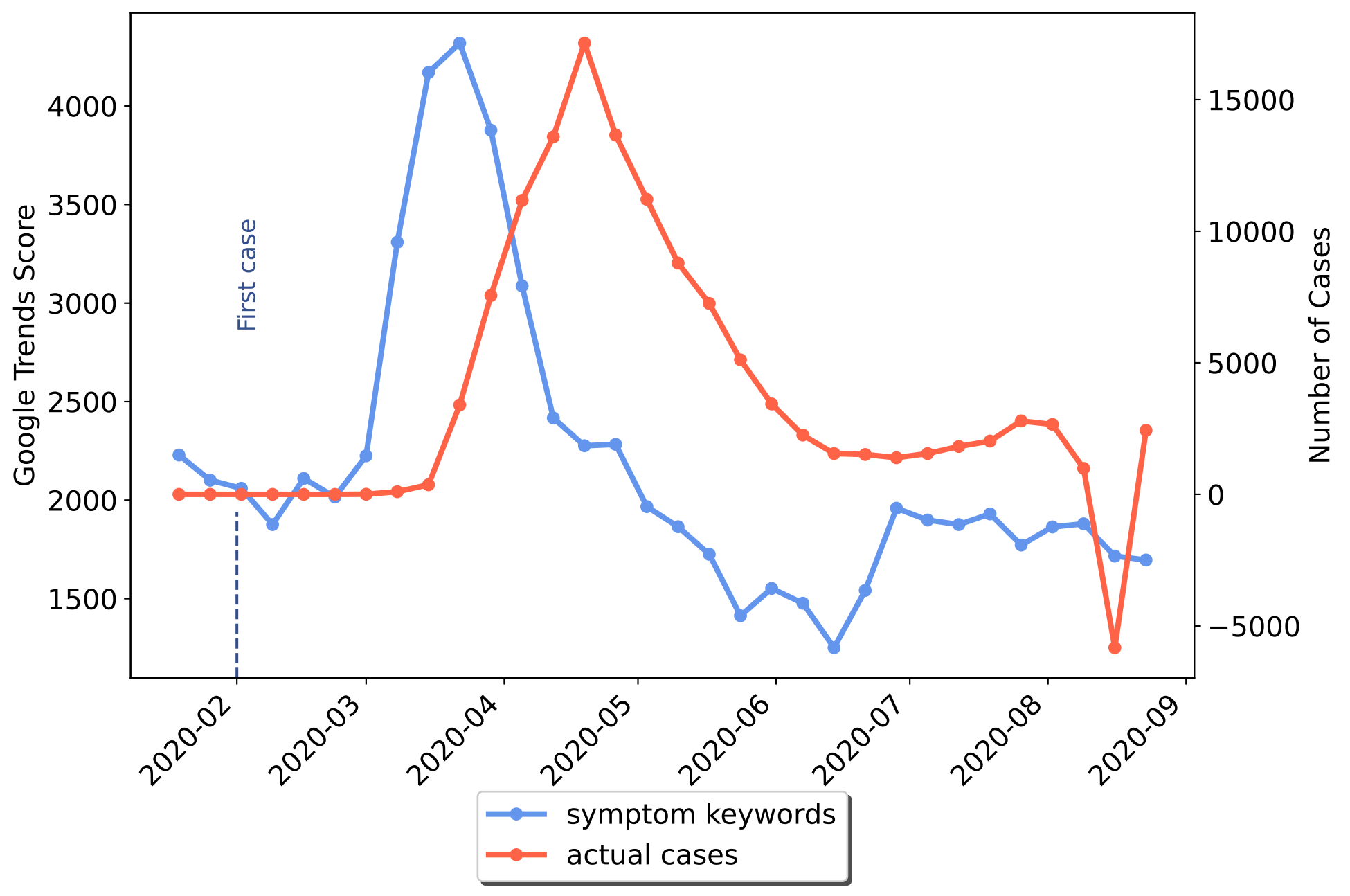

Supplement: Supplementary file 2 [file Data_Sheet_1.ZIP › figures/Massachusetts_totalsymptom_GT-eps-converted-to.pdf]
