# Supplementary material for: Prediction of COVID-19 Waves Using Social Media and Google Search: A Case Study of the US and Canada
Source: Front Public Health. 2021 Apr 16;9:656635. doi: 10.3389/fpubh.2021.656635 (PMC8085269; doi:10.3389/fpubh.2021.656635)
Supplement: Supplementary file 2 [file Data_Sheet_1.ZIP › figures/Virginia_totalsymptom_GT-eps-converted-to.pdf]

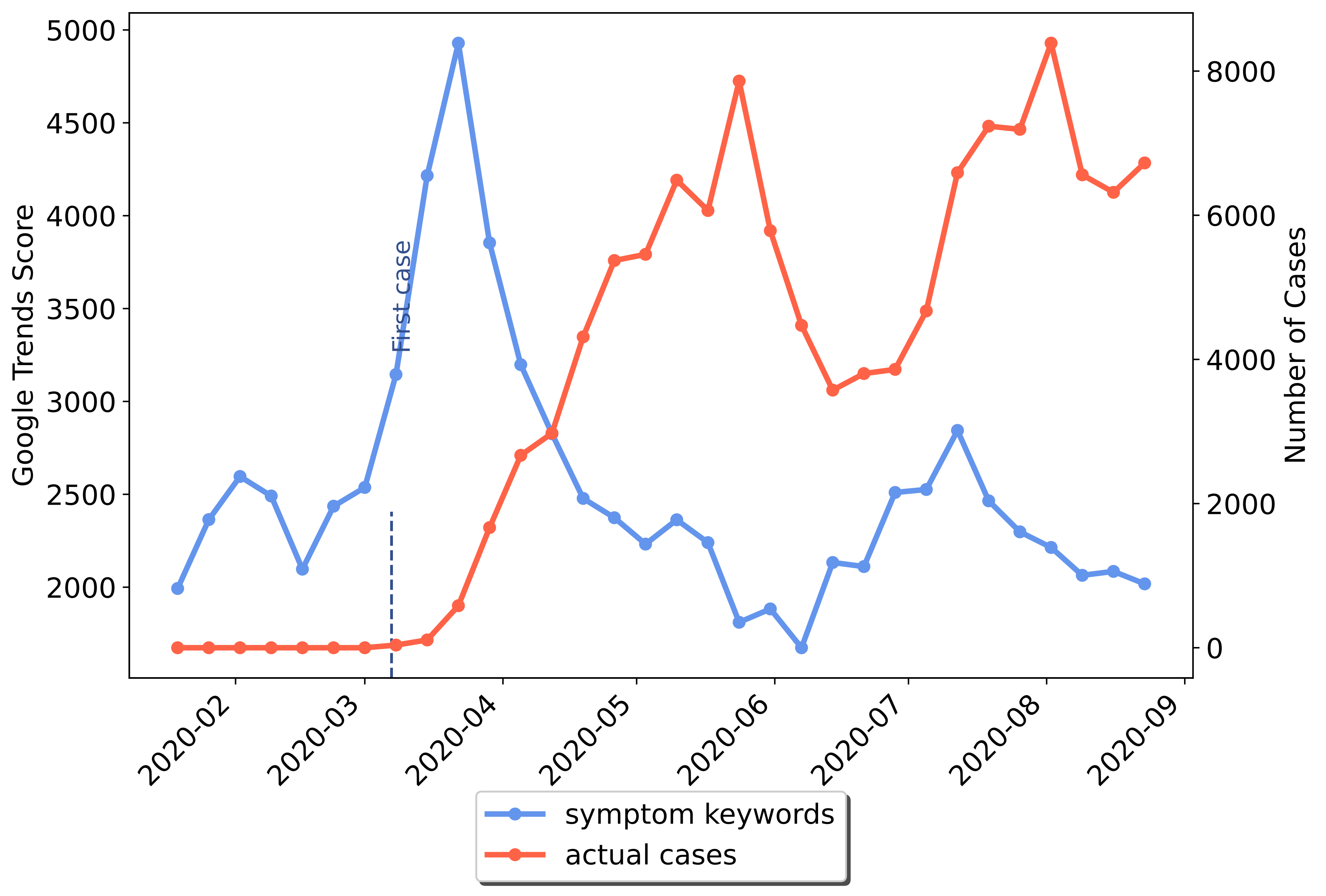

Google Trends Score
Number of Cases
5000
4500
4000
3500
3000
2500
2000
8000
6000
4000
2000
0
First case
2020-02
2020-03
2020-04
2020-05
2020-06
2020-07
2020-08
2020-09
symptom keywords
actual cases
