# Supplementary material for: Prediction of COVID-19 Waves Using Social Media and Google Search: A Case Study of the US and Canada
Source: Front Public Health. 2021 Apr 16;9:656635. doi: 10.3389/fpubh.2021.656635 (PMC8085269; doi:10.3389/fpubh.2021.656635)
Supplement: Supplementary file 2 [file Data_Sheet_1.ZIP › figures/Wisconsin_totalprecaution_GT-eps-converted-to.pdf]

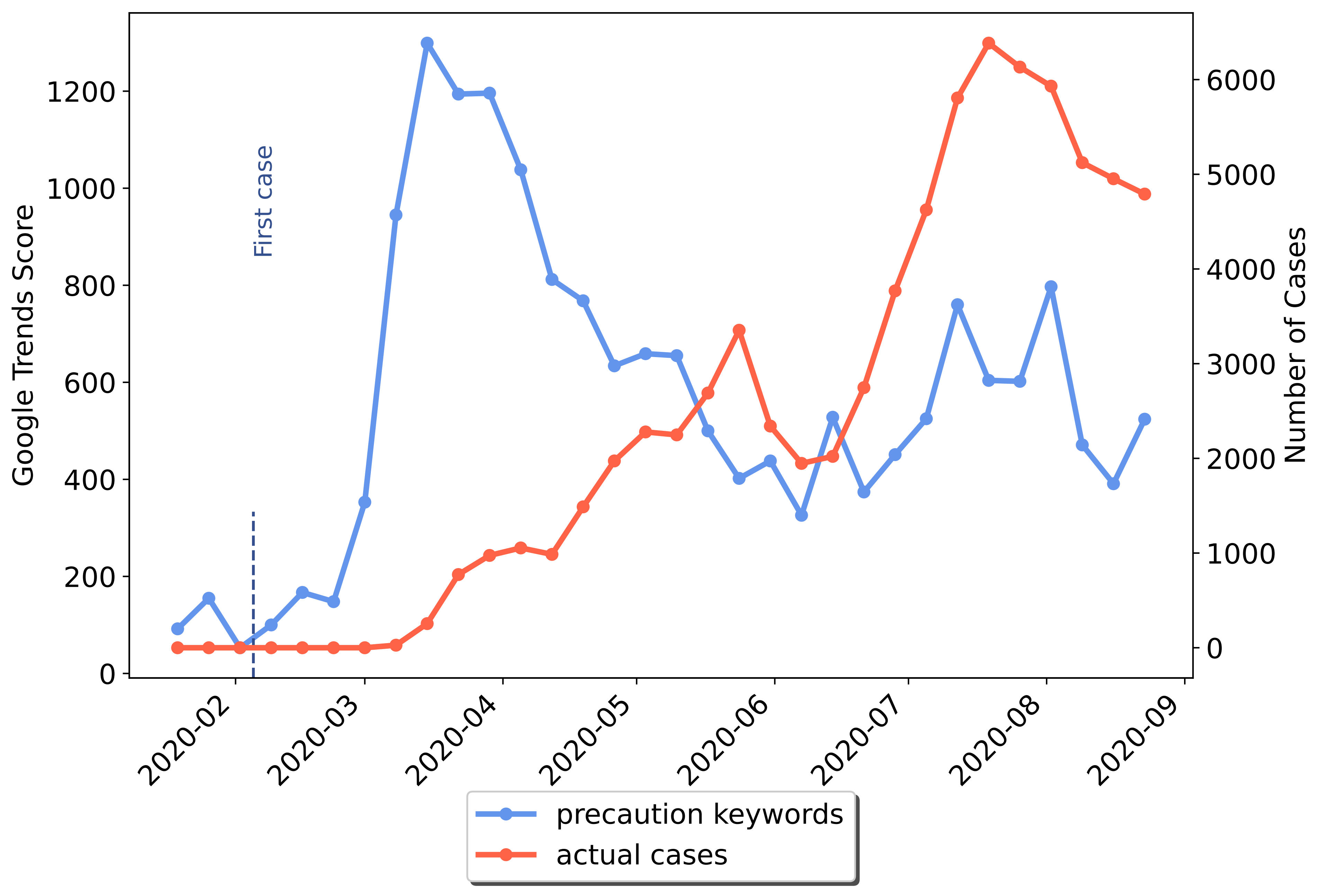

Google Trends Score
Number of Cases
First case
0
200
400
600
800
1000
1200
0
1000
2000
3000
4000
5000
6000
2020-02
2020-03
2020-04
2020-05
2020-06
2020-07
2020-08
2020-09
precaution keywords
actual cases
