# Supplementary material for: Prediction of COVID-19 Waves Using Social Media and Google Search: A Case Study of the US and Canada
Source: Front Public Health. 2021 Apr 16;9:656635. doi: 10.3389/fpubh.2021.656635 (PMC8085269; doi:10.3389/fpubh.2021.656635)
Supplement: Supplementary file 2 [file Data_Sheet_1.ZIP › figures/Wyoming_symptom_twitter-eps-converted-to.pdf]

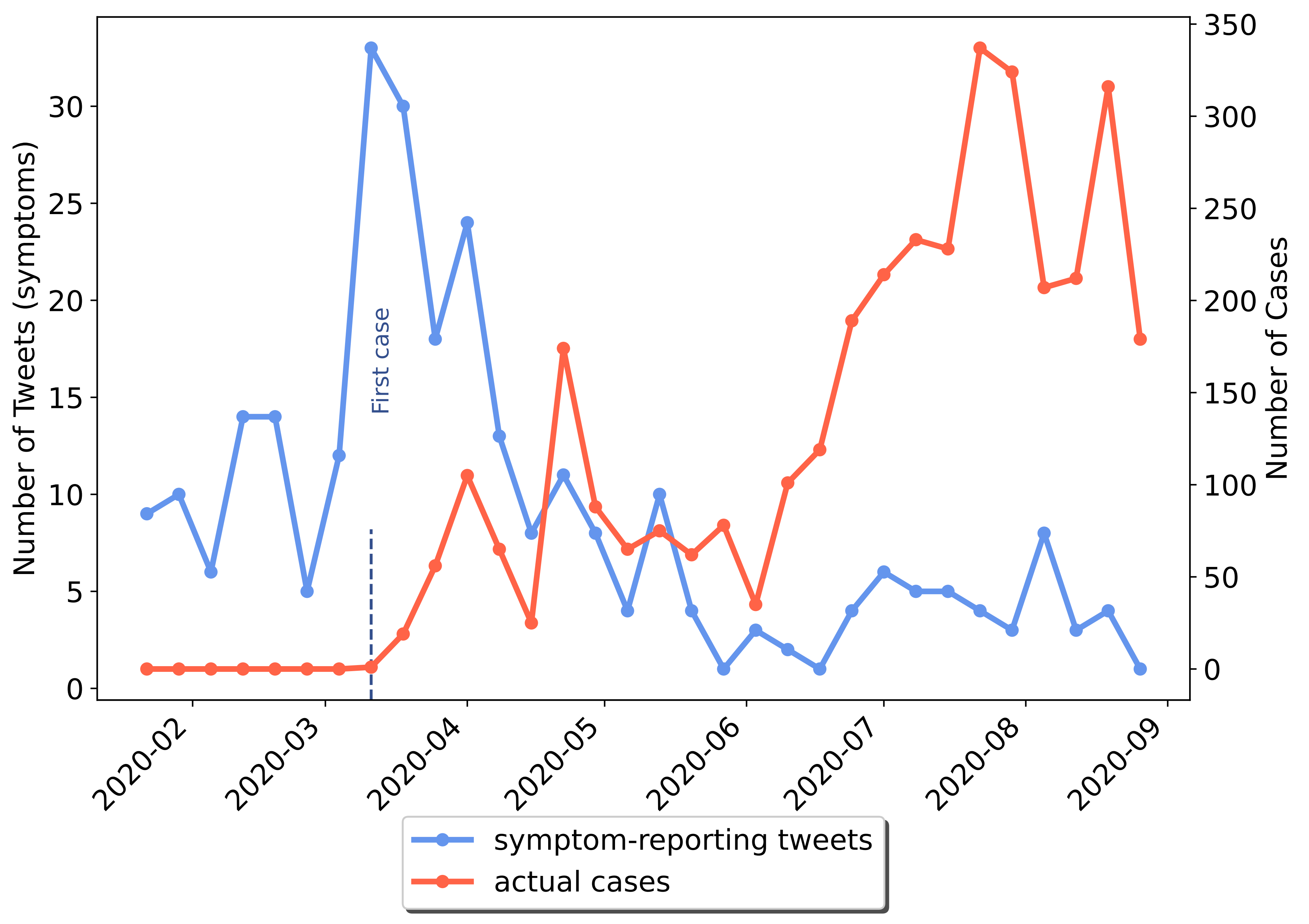

Number of Tweets (symptoms)
Number of Cases
First case
0
5
10
15
20
25
30
0
50
100
150
200
250
300
350
2020-02
2020-03
2020-04
2020-05
2020-06
2020-07
2020-08
2020-09
symptom-reporting tweets
actual cases
